# Supplementary material for: Paramagnetic relaxation enhancement NMR as a tool to probe guest binding and exchange in metallohosts
Source: Nat Commun. 2022 Apr 6;13:1846. doi: 10.1038/s41467-022-29406-1 (PMC8986849; doi:10.1038/s41467-022-29406-1)
Supplement: Supplementary file 1 — Supplementary Information [file 41467_2022_29406_MOESM1_ESM.pdf]

Supplementary Information for

Paramagnetic relaxation enhancement NMR as a tool to probe guest binding and exchange in metallohosts

Anne Swartjes, Paul B. White, Jeroen P. J. Bruekers, Johannes A. A. W. Elemans, Roeland J. M. Nolte

Table of contents

1. Supplementary Methods 4
1.1. Materials and instrumentation 4
1.2. Synthesis of V1 4
1.3. Synthesis of the unmetallated rotaxane (H_2Rot) 5
1.4. Synthesis of the Mn(III)Cl-rotaxane (MnRot) 6
1.5. Theory for PRE exchange studies 7
1.6. General method for PRE exchange studies 10
1.6.1. Sample preparation 11
1.6.2. Measurements 12
1.6.3. Data fitting 12
1.7. General procedure for the NMR binding titrations 13
1.7.1. Sample preparation 13
1.7.2. Measurements 17
1.7.3. Data fitting 17
1.8. Method for UV-Vis binding titrations 20
1.8.1. Sample preparation 20
1.8.2. Measurements 20
1.8.3. Data fitting 20
1.9. Method for sample preparation and measurement of selective EXSY of $H_2$1/V1 20
1.9.1. Sample preparation 20
1.9.2. Measurements 21
1.9.3. Data fitting 21

2. Supplementary figures and tables ... 21
2.1. PRE exchange studies ... 21
2.1.1. Host and guest dependence ... 21
2.1.2. T_1 fits Mn1/V1 ... 24
2.1.3. T_2 PROJECT-CPMG fits Mn1/V1 ... 26
2.1.4. T_1 fits Mn1/VP ... 29
2.1.5. T_2 fits Mn1/VP ... 31
2.1.6. T_1 fits Mn1/V2 ... 35
2.1.7. Determining dissociation parameters of Mn1/V2 ... 35
2.1.8. Extracting $T_{1,M}$ ... 38
2.1.9. All Eyring plots (Mn1/V1, Mn1/V2, Mn1/VP) ... 41
2.2. NMR binding titrations ... 42
2.2.1. Binding curves ... 42
2.2.2. Temperature dependence ... 43
2.3. UV-Vis binding titrations ($H_2$1/V1) ... 43
2.4. 1D EXSY studies ($H_2$1/V1) ... 46
3. NMR spectra of compounds ... 48
3.1. V1 ... 48
3.2. Mn1 ... 53
3.3. H_2Rot ... 55
3.4. MnRot ... 57
3.5. Mn1/V1 ... 61
3.6. Mn1/V2 ... 62
3.7. Mn1/VP ... 63
3.8. MnRot/V1 ... 64
3.9. MnRot/VP ... 65
4. Exchange: T_1 (inverse recovery) and T_2 (CPMG and PROJECT-CPMG) spectra ... 66
4.1. V1 ($T_{1,0}$ and $T_{2,0}$) ... 66
4.2. Mn1/V1 ($T_{1,obs}$ and $T_{2,obs}$) ... 84
4.3. MnRot/V1 ($T_{1,os}$ and $T_{2,os}$) ... 111
4.4. VP ($T_{1,0}$ and $T_{2,0}$) ... 137
4.5. Mn1/VP ($T_{1,obs}$ and $T_{2,obs}$) ... 156
4.6. MnRot/VP ($T_{1,os}$ and $T_{2,os}$) ... 174

4.7. V2 ($T_{1,0}$) 191
4.8. Mn1/V2 ($T_{1,obs}$) 200
5. Binding: T_1 (inverse recovery) spectra 207
5.1. Mn1/V1 207
5.2. Mn1/VP 218
5.3. Mn1/V2 227
5. How-to guide for utilizing PRE relaxometry studies to determine association and dissociation constants in paramagnetic host-guest complexes 237
Determination of association constants using relaxometry in PRE NMR 237
Determination of dissociation rate constants using relaxometry in PRE NMR 242
6. References 246

1. Supplementary Methods

1.1. Materials and instrumentation

Reagents and solvents used were of commercially available reagent quality unless indicated otherwise. Longitudinal relaxation rates were measured through inverse recovery experiments and spin-lattice relaxation rates were measured via (PROJECT)-CPMG experiments on a Bruker 500 MHz Avance III spectrometer equipped with a Prodigy BB cryoprobe, a Bruker 400 MHz Avance III HD nanobay spectrometer equipped with a BFFO probe, or a Bruker 300 MHz Avance III HD nanobay spectrometer equipped with a BBFO probe. For all VT experiments, the temperature was calibrated using pure ethylene glycol for temperatures of 20 °C and higher. Methanol was used to calibrate the temperatures lower than 20 °C.

Fluorescence quenching titrations were performed on a JASCO FP-8300ST Spectrofluorometer in a quartz cuvette with 1 cm path length. Samples for titrations consisted of 1:1 anhydrous chloroform and anhydrous acetonitrile. Anhydrous chloroform (>99%) and acetonitrile were obtained from Sigma-Aldrich. Acetonitrile was distilled and dried over CaCl2. Before use, anhydrous chloroform was filtered over dry K_2CO_3. The solution was irradiated with 419 nm for all studies at 22°C.

1.2. Synthesis of V1

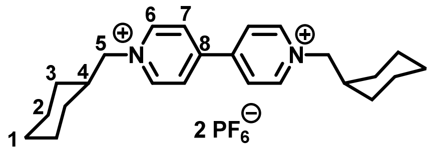

A solution was prepared containing 4,4'-bipyridine (300 mg, 1.92 mmol) and (bromomethyl)cyclohexane (1.36 g, 7.68 mmol) in anhydrous DMF (3 mL). The reaction mixture was stirred for 3 days at 90 °C. The solution was cooled to room temperature and the product was precipitated in diethyl ether. The crude

product was filtered and purified over silica (DCM : $MeNO_2$: MeOH, 6:1:1 v/v/v). Afterwards, the product was mixed with acetone (2 mL). The resulting suspension was added dropwise to a saturated solution of NH_4PF_6. The suspension was filtered and the precipitate was washed with water and dichloromethane. Compound **V1** was obtained as a white solid in a yield of 33%.

^{1}H NMR (500 MHz, acetonitrile-d_3): δ (ppm) = 8.85 (d, J = 5.84 Hz, 4H, **6**), 8.42 (d, J = 5.39 Hz, 4H, **7**), 4.47 (d, J = 7.28 Hz, 4H, **5**), 2.05 – 1.98 (m, 2H, **4**), 1.84 – 1.75 (m, 4H, $\mathbf{2_a}$), 1.74 – 1.68 (m, 4H, 1_a), 1.67 – 1.58 (m, 4H, 3_a), 1.35 – 1.19 (m, 6H, $\mathbf{1_b}$ & $\mathbf{2_b}$), 1.19 – 1.05 (m, 4H, 3_b).

^{13}C{^{1}H} NMR (125 MHz, acetonitrile-d_3): δ (ppm) = 149.94 (**8**), 145.71 (**6**), 127.57 (**7**), 67.32 (**5**), 39.40 (**4**), 29.35 (**3**), 25.55 (**1**), 25.15 (**2**).

MALDI-TOF: m/z: 350.095 $(M+H)^+$ calculated for $C_{24}H_{34}N_2 + H^+$ m/z: 351.550.

1.3. Synthesis of the unmetallated rotaxane (H_2Rot)

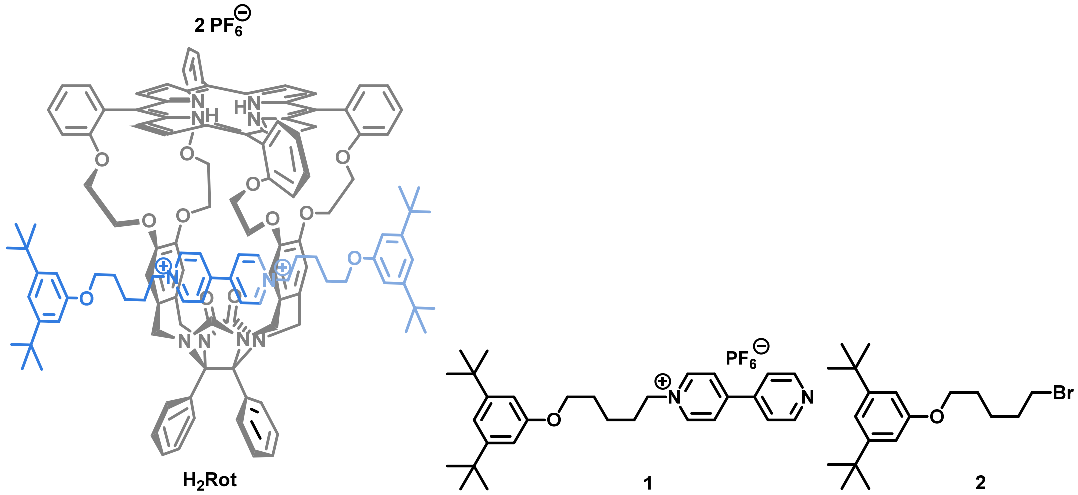

H_2Rot 1 2

Rotaxane **H_2Rot** was synthesized according to a previously reported procedure.[1] A Schlenk finger was evacuated and charged with **$H_2$1** (20 mg, 0.015 mmol)[2] and **1** (42.67 mg, 0.074 mmol)[3]. The starting materials were dissolved in anhydrous DMF (3 mL) and the resulting mixture was stirred after which **2** (211.44 mg, 0.595 mmol, synthesis previously reported[3]) was added and the mixture was heated to 90 °C and left stirring for 4 days. After allowing the reaction mixture to cool to room temperature, the solvent

was evaporated and the resulting crude solid was dissolved in acetonitrile. To this, a saturated solution of NH_4PF_6 was added. Water and chloroform were added and the organic layer was separated and evaporated. The crude product was purified over silica by flash column chromatography (10% acetonitrile, 90% dichloromethane) to give the product as a purple powder in a yield of 55%.

^{1}H NMR (400 MHz, chloroform-*d* : acetonitrile-d_3, 1:1, v/v): δ (ppm) = 9.15 (s, 4H), 8.82 (s, 4H), 8.13 (dd, *J* = 7.46, 1.74 Hz, 4H), 7.84 (td, *J* = 8.00, 1.63 Hz, 4H), 7.48 (t, *J* = 7.16 Hz, 4H), 7.44 (d, *J* = 8.30 Hz, 4H), 7.08 – 6.99 (m, 8H), 6.99 – 6.92 (m, 4H), 6.80 (d, *J* = 1.66 Hz, 4H), 6.30 (d, *J* = 6.00 Hz, 4H), 6.02 (s, 4H), 4.33 – 4.24 (m, 8H), 4.20 (d, *J* = 15.80 Hz, 4H), 3.66 – 3.52 (m, 4H), 3.48 – 3.29 (m, 4H), 2.79 – 2.60 (m, 4H), 1.84 – 1.66 (m, 4H), 1.33 (s, 36 H), 1.16 – 1.03 (m, 4H), −2.85 (s, 2H).

^{19}F NMR (376 MHz, chloroform-*d* : acetonitrile-d_3, 1:1, v/v): δ (ppm) = −73.73 (d, *J* = 710.76 Hz, 12H, PF_6).

MALDI-TOF: m/z: 2051.043 $(M+H)^+$ calculated for $C_{132}H_{133}N_{10}O_{12}$ + H^+ m/z: 2050.851.

1.4. Synthesis of the Mn(III)Cl-rotaxane (MnRot)

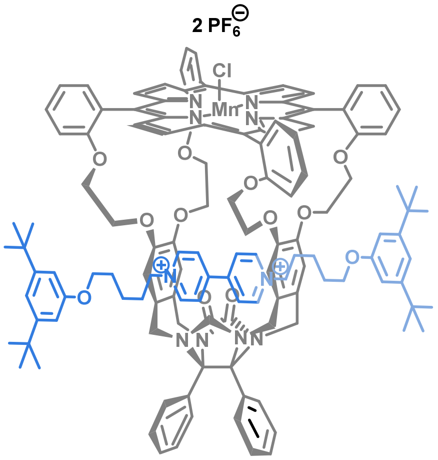

MnRot

A Schlenk finger was evacuated and charged with **H₂Rot** (50 mg, 0.0213 mmol), $MnCl_2$ (40 mg, 0.3195 mmol) and the starting materials were dissolved in a chloroform : methanol (2:1, v/v) mixture after which

1,2-lutidine (0.15 ml, 1.30 mmol) was added. The resulting mixture was heated to reflux and stirred for 2 days. Subsequently, more $MnCl_2$ (40 mg, 0.3195 mmol) and 1,2-lutidine (0.15 mL, 1.30 mmol) were added after which the mixture was stirred at reflux temperature for another day. Chloroform and water were added and the organic layer was extracted and washed with brine (2x) and water (2x) and evaporated *in vacuo*. The crude product was purified over silica using flash chromatography (20% acetonitrile, 80% chloroform) and gave **MnRot** as a green powder in a yield of 52%.

^{19}F NMR (376 MHz, chloroform-*d* : acetonitrile-d_3, 1:1): δ (ppm) = −73.95 (d, *J* = 707.56 Hz, 12H, PF_6).

MALDI-TOF: m/z: 2103.965 $(M+H)^+$ calculated for $C_{132}H_{131}MnN_{10}O_{12}$ + H^+ m/z: 2104.482.

1.5. Theory for PRE exchange studies

The relaxation of a nucleus after excitation is governed by two time constants: T_1 (spin-lattice) and T_2 (spin-spin). If the magnetization contains only longitudinal elements, then T_1 relaxation is the operative mode, which serves to return the magnetization to its equilibrium position along the Z-axis. If the magnetization contains transverse elements, then T_2 relaxation governs relaxation in the XY plane and T_1 relaxation returns the bulk magnetization along the Z-axis. The rates of these processes are determined by taking the inverses of the respective time constants (Supplementary Equation. 1).

$$\mathrm{R_{1,obs}} = \frac{1}{\mathrm{T_{1,obs}}} \quad \text{and} \quad \mathrm{R_{2,obs}} = \frac{1}{\mathrm{T_{2,obs}}}$$ Supplementary Equation 1

In the presence of a paramagnetic center, an increase in the longitudinal ($R_{1,obs}$) and transverse ($R_{2,obs}$) relaxation rate of a given nucleus can often be observed. This observed relaxation rate is defined as the sum of the diamagnetic (R_0) and paramagnetic (R_p) relaxation rates (Supplementary Equation. 2). The diamagnetic relaxation rate can be considered the natural relaxation rate in the absence of the

paramagnetic component, whereas the paramagnetic relaxation rate is the rate enhancement due to the presence of the paramagnetic center.

$$R_{1,obs} = R_{1,0} + R_{1,p}$$ Supplementary Equation 2

There are three regimes of exchange (fast, intermediate and slow) that provide information about the interaction of a guest with a paramagnetic center. In the fast-exchange regime, the rate constant of exchange is much greater than the difference in relaxation rate between the ligand in the free state and in the (bound) state near a paramagnetic center (i.e. $k_{ex} >> |R_{1,p} – R_{1,0}|$). When this regime is present, $R_{1,obs}$ becomes the population-weighted sum of the diamagnetic and paramagnetic relaxation components (Supplementary Equation 3).

$$R_{1,obs} = \chi_f \cdot R_{1,0} + \chi_b \cdot R_{1,p}$$ Supplementary Equation 3

Where χ_f and χ_b are the mole-fractions of the free and bound ligand, respectively. By varying the total ligand or host concentration and measuring how the observed relaxation rate changes, the binding constant for the given ligand can be calculated.

In the opposite extreme case, where $k_{ex} << |R_{1,p} – R_{1,0}|$), the exchange process is considered to be in the slow regime. Here, the observed relaxation enhancement effect on the ligand becomes dependent on the exchange rate constant. The $R_{1,p}$ term in Supplementary Equation 2 can be further broken up into inner-sphere ($\frac{f}{\tau_M + T_{1M}}$) and outer-sphere terms ($R_{1,os}$), which represents long-range, non-exchanging dipolar relaxation effects of the paramagnetic center on free ligands in solution (Supplementary Equation 4).

$$R_{1,p} = \frac{f}{\tau_M + T_{1M}} + R_{1,os}$$ Supplementary Equation 4

The inner-sphere term contains information about the number of binding sites in the host and the mole-fraction of the ligand bound to the host, which is indicated by the term ''f''. Under saturation conditions, this is simply the ratio of total host to total guest, however the fraction of the host bound by guest must be determined for any given ligand/host sample. In the denominator lies the mean-residence time (τ_M) of the guest bound to the host as well as the relaxation time constant (T_{1M}) it experiences while bound. The inverse of the mean-residence time represents the observed rate constant for the exchange process ($\tau_M^{-1} = k_{d,obs}$). If the order of the ligand and host concentration are found to be zero- and first-order, respectively, then $k_{d,obs} = k_d$. Otherwise, different reaction orders must be accounted for when calculating k_d. For the inner-sphere term, three possible scenarios exist which set boundary conditions for the range of measureable rate constants. First, if $\tau_M >> T_{1M}$, then $R_{1,p}$ can be written simply as:

$$R_{1,p} = f \cdot k_{d,obs} + R_{1,os}$$ Supplementary Equation 5

In this case, $R_{1,p}$ is dominated by $k_{d,obs}$ and the dissociation constant can be easily determined. Second, if $\tau_M << T_{1M}$, then the fast-exchange regime as described by Supplementary Equation 3 is dominant and rate information can no longer be obtained. Lastly, as τ_M approaches infinity due to very slow dissociation, then $R_{1,p}$ is dominated by the outer-sphere term as the inner-sphere term approaches zero and rate information is again lost.

Therefore, in order for a given paramagnetic host-guest system to be suitable for measuring the kinetics of ligand dissociation, $R_{1,p}$ must be dominated by the residence time, τ_M. If this condition holds, then three measureable observations result, as outlined by Mildvan[4]:

1. since $\tau_M > T_{1M}$ and $T_{1M} \geq T_{2M}$, it must also hold true that $\tau_M > T_{2M}$ and dominates $R_{2,p}$. Therefore, $R_{1,p} \approx R_{2,p}$.
2. since τ_M decreases with increasing temperature, $R_{1,p}$ and $R_{2,p}$ should also increase with temperature ($\frac{\partial R_{1/2,p}}{\partial T} > 0$) until the point where $\tau_M \approx T_{1M}$ or T_{2M}. On the other hand, if T_{1M} or T_{2M}, were dominant, then $R_{1,p}$ and $R_{2,p}$ would decrease with increasing temperature ($\frac{\partial R_{1/2,p}}{\partial T} < 0$).
3. as τ_M is a residence time and by its nature is independent of field frequency, $R_{1,p}$ and $R_{2,p}$ should also be independent of frequency ($\frac{\partial R_{1/2,p}}{\partial \omega} = 0$).

While these criteria hold true, the simplification for $R_{1,p}$ in Eq. 5 is valid, and rate constants for dissociation can be readily extracted. However, as the residence time approaches the very slow ($\tau_M > T_{1/2,os}$) or intermediate-fast ($\tau_M \approx T_{1/2M}$) exchange limit, the analysis becomes complicated and is no longer straightforward.

1.6. General method for PRE exchange studies

Below, the general procedure for the PRE measurements used for extraction of the dissociation rate constants and activation energies for dissociation of the guest molecule from the cavity of **Mn1** is described. The PRE exchange studies are performed for systems that are in the slow, intermediate and fast exchange regimes in which different parameters dominate.

As mentioned before, in the intermediate exchange regime the inner-sphere interactions dominate and the dissociation rate constant can be determined by measuring the diamagnetic relaxation rates ($R_{1/2,d}$) and the observed relaxation rates ($R_{1/2,obs}$). The $R_{1/2,d}$ values are measured by NMR samples containing the

viologen guest compound in absence of the paramagnetic host (**Mn1**) and the $R_{1/2,obs}$ values are measured by samples containing the viologen guest compound in presence of **Mn1**.

In the slow exchange regime, the outer-sphere interactions dominate and the dissociation rate constant can be determined by measuring $R_{1/2,d}$, $R_{1/2,obs}$ and the outer-sphere relaxation rates $R_{1/2,os}$. The $R_{1/2,os}$ values are measured by NMR samples containing the viologen guest compound in presence of the paramagnetic rotaxane (**MnRot**).

In the fast exchange regime, the inner-sphere interactions dominate and the dissociation rate constant can be determined by measuring $R_{1/2,d}$ and $R_{1/2,obs}$. Furthermore, before extracting the dissociation rate constants the inner-sphere relaxation rates ($R_{1/2,M}$) should be calculated. This is further explained in chapter **2.1.7** and **2.1.8.** (p36-41).

1.6.1. Sample preparation

$R_{1,obs}$ and $R_{2,obs}$

Mn1 (0.35 µmol for **Mn1**/**V1** and **Mn1**/**VP** and 0.035 µmol for **Mn1**/**V2**) was dissolved in 0.35 mL chloroform-*d*. To the solution, the guest (**V1**, **V2**, or **VP**) (3.50 µmol) in acetonitrile-d_3 (0.35 mL) was added and the resulting host/guest mixture was transferred to a 5 mm NMR tube. The sample was capped with a 1.5 mm × 3.9 mm sleeve stopper septum and sealed with parafilm to keep the concentration constant. The samples were stored at −8 °C.

$R_{1,0}$ and $R_{2,0}$

Guest compound (**V1**, **V2** or **VP**) (3.50 µmol) was dissolved in acetonitrile-d_3 (0.35 mL) and chloroform-*d* (0.35 mL) was added and the resulting mixture was transferred to a 5 mm NMR tube. The sample was capped with a 1.5 mm × 3.9 mm sleeve stopper septum and sealed with parafilm to keep the concentration constant. The samples were stored at −8 °C.

$R_{1,os}$ and $R_{2,os}$

MnRot (0.35 µmol) was dissolved in 0.35 mL chloroform-*d*. To the solution, the guest (**V1** or **VP**) (3.50 µmol) in acetonitrile-*d₃* (0.35 mL) was added and the resulting host/guest mixture was transferred to a 5 mm NMR tube. The sample was capped with a 1.5 mm × 3.9 mm sleeve stopper septum and sealed with parafilm to keep the concentration constant. The samples were stored at −8 °C.

1.6.2. Measurements

All PRE NMR measurements for the extraction of dissociation rate constants were performed on a Bruker 500 MHz Avance III spectrometer equipped with a prodigy BB cryoprobe. All temperatures included in the studies were calibrated with ethylene glycol (> 20 °C) and methanol (≤ 20 °C). At each temperature, the 90-degree pulse was measured. For T_1 measurements the inverse recovery pulse sequence was utilized. For T_2 measurements, CPMG[5] and PROJECT-CPMG[6] pulse sequences were used.

1.6.3. Data fitting

T_1 data

The T_1 (and R_1) values were determined by fitting the obtained data in MNOVA with a three-parameter exponential fit (Supplementary Equation 6)

$$y = B + F \cdot e^{-\frac{t}{T_1}} \qquad \text{Supplementary Equation 6}$$

T_2 data

The T_2 (and R_2) values were determined by fitting the obtained data in a custom expression in Origin 2020:

```
Independent: t
Dependent variables: Mt
Parameters: R2, M0
Expression: Mt = M0*exp(-R2*t)
```

In which t is the time, Mt is the magnetization at time t, M0 is the initial magnetization and R2 is the spin-lattice relaxation rate (R_2). In the CPMG and PROJECT-CPMG pulse sequences the magnetization is measured at different loop counts. The time can be calculated from the number of loops in CPMG experiments following Supplementary Equation 7 and in PROJECT-CPMG experiments following Supplementary Equation 8.

$$t = n(2\boldsymbol{d20} + 2\boldsymbol{pw})$$ Supplementary Equation 7

$$t = n(\boldsymbol{d2} + 5\boldsymbol{pw})$$ Supplementary Equation 8

in which n is the number of loops, d20 and d2 are delay times (s) for CPMG and PROJECT-CPMG experiments, respectively, and pw is the 90-degree pulse width (s). A schematic depiction of the CPMG and PROJECT-CPMG experiments is given in Supplementary Figure 1.

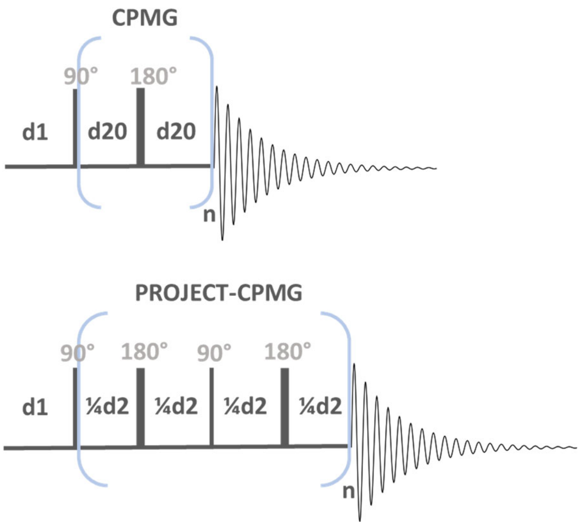

Supplementary Figure 1. Schematic depictions of spin-lattice relaxation rate experiments. top: CPMG, bottom: PROJECT-CPMG

1.7. General procedure for the NMR binding titrations

1.7.1. Sample preparation

Deuterated solvents (chloroform-*d* and acetonitrile-*d*$_3$) were obtained commercially. Chloroform was filtered over K_2CO_3 and Na_2SO_4 prior to making the samples.

Mn1/V1

A stock solution of host was prepared by dissolving **Mn1** (3.01 mg, 2.1 µmol) in a 4 : 1 mixture of $CDCl_3$: $CHCl_3$ (3.5 mL) and a guest stock solution was prepared by dissolving **V1** (4.61 mg, 7.20 µmol) in a separate vial in CD_3CN. From the host stock solution 300 µL was transferred to 10 NMR tubes (5 mm). From the guest stock solution (**V1** stock 1) a 7.15-fold dilution was prepared (**V1** stock 2) by transferring 140 µL of the stock solution to a separate vial and 850 µL CD_3CN was added. For each NMR tube an increasing amount of the guest stock solution was added and CD_3CN until the total sample volume was 600 µL (See Supplementary Tables 1 and 2). The samples were stored at −8 °C.

Supplementary Table 1. Overview of equivalents V1 added to each sample.

Sample	[Mn1] mM	[V1] mM	V1 eq.
**1**	0.30	0	0
**2**	0.30	0.07	0.23
**3**	0.30	0.14	0.47
**4**	0.30	0.25	0.84
**5**	0.30	0.42	1.40
**6**	0.30	0.63	2.10
**7**	0.30	0.90	3.00
**8**	0.30	1.35	4.50
**9**	0.30	2.10	7.00
**10**	0.30	3.00	10.00

Supplementary Table 2. Overview of volumes of stock solutions added to each sample.

Sample	Mn1 stock (µL)	V1 stock 1 (µL)	V1 stock 2 (µL)	CD_3CN (µL)
**1**	300		0	300
**2**	300		50	250
**3**	300		100	200
**4**	300		180	120
**5**	300		300	0
**6**	300	63		237
**7**	300	90		210
**8**	300	135		165
**9**	300	210		90
**10**	300	300		0

Mn1/VP

A stock solution of host was prepared by dissolving **Mn1** (0.43 mg, 0.3 µmol) in a 4 : 1 mixture of $CDCl_3$: $CHCl_3$ (0.5 mL) and a guest stock solution was prepared by dissolving **VP** (28.60 mg, 6.58 µmol) in a separate vial in CD_3CN. From the host stock solution 40 µL was transferred to 9 capillary (100 µL reference capacity) NMR tubes. From the guest stock solution (**VP** stock 1) a 5.35-fold dilution was prepared (**VP** stock 2) by transferring 28.2 µL of the stock solution to a separate vial and 121.8 µL CD_3CN was added. For each NMR tube an increasing amount of the guest stock solution was added and CD_3CN until the total sample volume was 80 µL (See Supplementary Tables 3 and 4). The samples were left at room temperature for one day to allow threading and afterwards the samples were stored at −8 °C.

Supplementary Table 3. Overview of equivalents VP added to each sample.

Sample	[Mn1] mM	[VP] mM	VP eq.
1	0.30	0	0
2	0.30	0.68	2.26
3	0.30	1.47	4.89
4	0.30	2.71	9.02
5	0.30	4.51	15.00
6	0.30	7.20	24.00
7	0.30	11.40	37.90
8	0.30	16.80	55.90
9	0.30	24.00	80.00

Supplementary Table 4. Overview of volumes of stock solutions added to each sample.

Sample	Mn1 stock (µL)	VP stock 1 (µL)	VP stock 2 (µL)	CD_3CN (µL)
**1**	40		0	40
**2**	40		6	34
**3**	40		13	27
**4**	40		24	16
**5**	40		40	0
**6**	40	12		28
**7**	40	19		21
**8**	40	28		12
**9**	40	40		0

Mn1/V2

A stock solution of host was prepared by dissolving **Mn1** (3.01 mg, 2.1 µmol) in a 4 : 1 mixture of $CDCl_3$: $CHCl_3$ (3.5 mL) and a guest stock solution was prepared by dissolving **V2** (25.71 mg, 54.00 µmol) in a separate vial in CD_3CN. From the host stock solution 300 µL was transferred to 10 NMR tubes (5 mm). From the guest stock solution (**V2** stock 1) a 10-fold dilution was prepared (**V2** stock 2) by transferring 100 µL of the stock solution to a separate vial and 900 µL CD_3CN was added. For each NMR tube an increasing amount of the guest stock solution was added and CD_3CN until the total sample volume was 600 µL (See Supplementary Tables 5 and 6). The samples were stored at −8 °C.

Supplementary Table 5. Overview of equivalents V2 added to each sample.

Sample	[Mn1] mM	[V2] mM	V2 eq.
**1**	0.30	0	0
**2**	0.30	0.30	1.00
**3**	0.30	0.60	2.00
**4**	0.30	1.05	3.50
**5**	0.30	1.73	5.75
**6**	0.30	2.93	9.75
**7**	0.30	4.88	16.25
**8**	0.30	8.10	27.00
**9**	0.30	13.50	45.00
**10**	0.30	22.50	75.00

Supplementary Table 6. Overview of volumes of stock solutions added to each sample.

Sample	Mn1 stock (µL)	V2 stock 1 (µL)	V2 stock 2 (µL)	CD_3CN (µL)
**1**	300		0	300
**2**	300		40	260
**3**	300		80	220
**4**	300		140	160
**5**	300		230	70
**6**	300	39		261
**7**	300	65		235
**8**	300	108		192
**9**	300	180		120
**10**	300	300		0

1.7.2. Measurements

Inverse recovery experiments for determining T_1 values were performed on a Bruker 300 MHz Avance III HD nanobay spectrometer equipped with a BBFO probe at 293 K.

1.7.3. Data fitting

T_1 values were obtained by fitting the data from the inverse recovery experiments in MNOVA using the three-parameter estimation, described in **1.5.3.**.Binding constants (K_a) were determined by fitting the obtained relaxation data in a custom equation in Origin 2020:

```
a = Ka;

b = 1 - Ka*Gt + Ka*Ht;

c = -Gt;

G = (-b + sqrt(b^2 - 4*a*c))/(2*a);

Robs = ((Rb - Rf)*(Gt - G))/Ht + Rf;

Parameters:

Ka, Rb

Constants:

Ht, Rf
```

In which:

Gt = $[G]_{total}$ = the total guest concentration

Ht = $[H]_{total}$ = the total host concentration

Rb = R_{bound} = the relaxation rate of the bound species

Rf = R_{free} = the relaxation rate of the unbound species

The derivation of the used equation is presented below:

The total relaxation rate (R_{obs}) consists of a component originating from unbound (R_{free}) host and a component originating from bound host (R_{bound}) (Supplementary Equation 9). Each of these components contribute proportional to the population of the respective species (Supplementary Equation 10 and Supplementary Equation 11).

$$R_{obs} = \chi_{free} R_{free} + \chi_{bound} R_{bound}$$ Supplementary Equation 9

$$\chi_{free} = \frac{[H]_{free}}{[H]_{total}}$$ Supplementary Equation 10

$$\chi_{bound} = \frac{[HG]}{[H]_{total}}$$ Supplementary Equation 11

In which [H] is the host concentration, [H] the total host concentration and [HG] concentration of the host-guest complex. Combining Supplementary Equations 9-11 gives Supplementary Equation 12.

$$R_{obs} = \frac{[H]_{free}}{[H]_{total}} R_{free} + \frac{[HG]}{[H]_{total}} R_{bound}$$ Supplementary Equation 12

Multiplying Supplementary Equation 12 by $[H]_{total}$ gives:

$$R_{obs}[H]_{total} = [H]R_{free} + [HG]R_{bound}$$ Supplementary Equation 13

The concentration of free host $[H]_{free}$ can be rewritten as the subtraction of the bound host concentration (concentration of the host-guest complex, [HG]):

$$[H]_{free} = [H]_{total} - [HG]$$ Supplementary Equation 14

After which Supplementary Equation 13 can be rewritten as Supplementary Equations 15-17

$$R_{obs}[H]_{total} = ([H]_{total} - [HG])R_{free} + [HG]R_{bound} \qquad \text{Supplementary Equation 15}$$

$$R_{obs}[H]_{total} = [H]_{total}R_{free} + [HG](R_{bound} - R_{free}) \qquad \text{Supplementary Equation 16}$$

$$R_{obs} = R_{free} + \frac{[HG](R_{bound} - R_{free})}{[H]_{total}} \qquad \text{Supplementary Equation 17}$$

Lastly, the concentration of the host-guest complex [HG] can be rewritten according to Supplementary Equation 18 giving the final Supplementary Equation 19.

$$[HG] = [G]_{total} - [G]_{free} \qquad \text{Supplementary Equation 18}$$

$$R_{obs} = R_{free} + ([G]_{total} - [G]_{free})\frac{R_{bound} - R_{free}}{[H]_{total}} \qquad \text{Supplementary Equation 19}$$

The concentration of free guest ($[G]_{free}$) can be calculated from the equilibrium law

$$H_{free} + G_{free} \rightleftharpoons HG \qquad \text{Supplementary Equation 20}$$

$$K_a = \frac{[HG]}{[H]_{free}[G]_{free}} \qquad \text{Supplementary Equation 21}$$

Using Supplementary Equations 14 and 18, Supplementary Equation 21 can be rewritten to Supplementary Equations 22 and 23.

$$K_a = \frac{([G]_{total} - [G]_{free})}{([H]_{total} - [G]_{total} + [G]_{free})[G]_{free}} \qquad \text{Supplementary Equation 22}$$

$$K_a[G]_{free}^2 + (1 - K_a[G]_{total} + K_a[H]_{total})[G]_{free} - [G]_{total} = 0 \qquad \text{Supplementary Equation 23}$$

Supplementary Equation 23 can be solved using the quadratic formula Supplementary Equation 24.

$$[G]_{free} = \frac{-b \pm \sqrt{b^2 - 4ac}}{2a} \qquad \text{Supplementary Equation 24}$$

In which:

$a = K_a$

$b = 1 - K_a[G]_{total} + K_a[H]_{total}$

$c = -[G]_{total}$

1.8. Method for UV-Vis binding titrations

1.8.1. Sample preparation

MeCN was freshly distilled over CaH_2 and $CHCl_3$ freshly distilled over Sicapent followed by a filtration over anhydrous K_2CO_3. A 1:1 v/v mix of MeCN and $CHCl_3$ was prepared from which all the solutions described below were prepared. Stock solutions of **$H_2$1** (3.83 mg in 10.00 mL; 0.285 mM) and **V1** (7.29 mg in 10.00 mL; 1.14 mM) in 1:1 v/v mix of MeCN and $CHCl_3$ in 10.00 mL volumetric flasks. From these stock solutions three solutions were prepared for the titration. All three solutions contained **$H_2$1** (1.99 µM) and only two contained **V1** (80.0 µM and 800 µM).

1.8.2. Measurements

UV-Vis spectra were recorded on a Varian Cary 50 UV-Vis spectrophotometer from 700 nm to 300 nm with a scan rate of 4800 nm/min. All spectra were baseline corrected by measuring a blank of only the solvent mixture in the same cuvette as the samples. UV-Vis titrations were performed by first measuring the pure host solution (2.000 mL) followed by small additions of either **V1** solutions (5 µL – 250 µL) and measuring the UV-Vis spectrum after each addition.

1.8.3. Data fitting

The data from the titrations in Supplementary Tables 15 – 17, chapter **2.3.** (p44-46) was fitted using an online fitting tool: *http://app.supramolecular.org/bindfit/*[7–9] to provide the binding parameters shown in Supplementary Table 14 and the fits depicted in Supplementary Tables 18 – 20.

1.9. Method for sample preparation and measurement of selective EXSY of $H_2$1/V1

1.9.1. Sample preparation

A stock solution of internal standard pentafluorobenzaldehyde (0.68 mg, 3.5 µmol) was prepared in $CDCl_3$ (1 mL) of which 100 µL was transferred to a solution of **$H_2$1** (0.47 mg, 0.35 µmol) dissolved in $CDCl_3$ (250 µl). Consequently, **V1** (2.24 mg, 3.5 µmol) was dissolved in CD_3CN (350 µL) and the host and guest mixture

were mixed and transferred to an NMR tube (5 mm) capped with a 1.5 mm × 3.9 mm sleeve stopper septum and sealed with parafilm to keep the concentration constant. The sample was stored at −8 °C.

1.9.2. Measurements

Selective 1D NOESY experiments were performed on a Bruker 500 MHz Avance III spectrometer equipped with a prodigy BB cryoprobe. All temperatures were calibrated using The pulse sequence used for the 1D NOESY experiments is described in previous studies[3]. The selected signal from the ^{1}H-NMR spectrum is bound **V1** that exchanges with free **V1**.

1.9.3. Data fitting

The mix times were carefully chosen so that the maximum conversion of bound **V1** to free **V1** did not exceed ~20%, making it possible to fit the obtained data linearly and calculate the dissociation rate constants from the initial rates by dividing the slope of the fits by the concentration of bound **V1**.

2. Supplementary figures and tables

2.1. PRE exchange studies

2.1.1. Host and guest dependence

It was found that increasing guest concentration for complex **Mn1**/**V1** promoted the dissociation of the guest from the cavity of the host. The host and guest dependence were determined by preparing 5 samples with varying host concentration and varying guest concentration (Supplementary Table 7). The results show that varying the concentration of **Mn1** does not influence the residence time of **V1** near or on the metal center of the host. In contrast, Supplementary Table 7 shows that if the concentration of **V1** is decreased, the residence time is increased. This means that a higher concentration of **V1** promotes faster exchange. Depicted in Supplementary Figure 2 is the log of the concentration of guest plotted against the log of the inverse τ_M giving the order of the guest dependence as the slope.

To determine whether the guest dependence is a result of the structure of the host or the guest, the order of several different complexes was measured: manganese-inserted porphyrin cage with symmetric

bis(methylcyclohexyl) viologen (**Mn1**/**V1**, Supplementary Figure 2a), manganese-inserted porphyrin cage with a non-symmetric viologen guest that contains a bis(tertbutyl)phenyl group connected to the 4,4'-bipyridyl moiety via a C5-linker equal to **VP** and a methylcyclohexyl group as head group (**Mn1**/**V3**, Supplementary Figure 2b)). This complex is measured as a comparable substitute complex for **Mn1**/**VP** due to solubility complications and the requirement of a significant amount of guest. As shown in Figs. S2a and S2b, both complexes display a ligand dependence order of >0. However, no distinct value for the order can be given as values between ~0.3 and ~0.4 for the slopes are obtained for **Mn1**/**V1** and **Mn1**/**V3**, respectively. From this, we can conclude that the guest concentration promotes exchange, but no distinct order of the exchange can be determined. Because dissociation activation energy barriers determined after correcting for either a 0.3 and 0.4 order ligand dependence do not differ significantly, we can state that structural changes from a symmetric guest to a non-symmetric guest and differences in steric factors of the guest compound have little influence the order of exchange.

Consequently, the influence of the structure of host was investigated by determining the ligand dependence on the non-metallated porphyrin cage compound (**$H_2$1**) and **V1** (Supplementary Figure 2c) and a non-metallated porphyrin cage, of which the xylylene-sidewall phenyl is substituted with a single nitro-group (**$H_2$2**) changing the electronic and steric properties of the host slightly from **$H_2$1**, with **V3** (Supplementary Figure 2d). For both **$H_2$1/V1** and **$H_2$2/V3** a zero-order guest dependence was found, indicating that the Mn(III)Cl insertion into the porphyrin roof is the crucial factor to introducing the non-integer order guest dependence.

Supplementary Table 7. Host and guest dependence on the residence time.

[Mn1] (mM)	[V1] (mM)	f	Residence time (τ_M, ms)
**0.50**	5.0	0.096	2.0
**0.25**	5.0	0.048	2.1
**0.10**	5.0	0.019	2.1
**0.10**	2.5	0.037	2.8
**0.10**	1.0	0.082	3.6

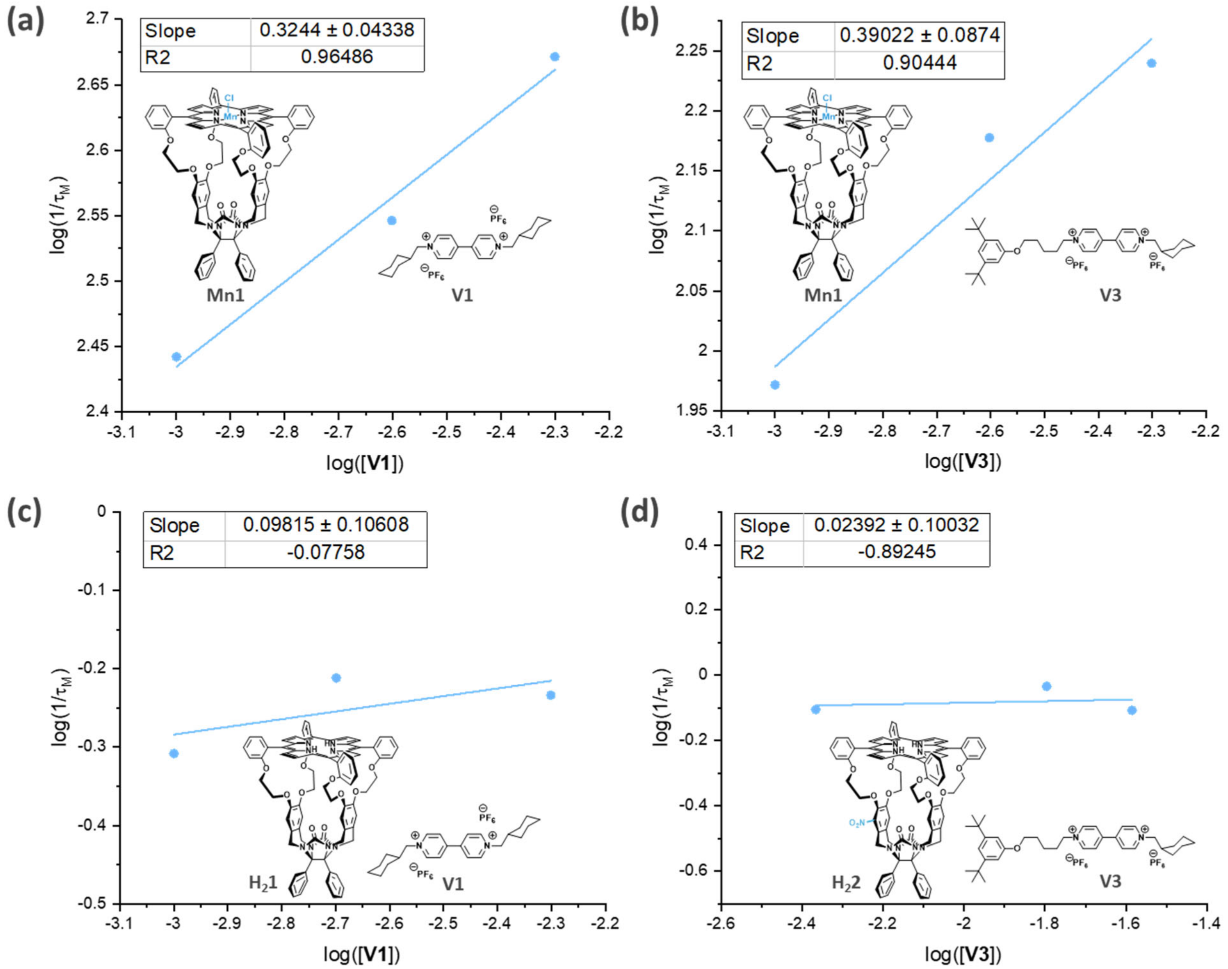

Supplementary Figure 2. Host and guest dependence on the residence time. a. Mn1/**V1** displaying a non-integer exchange-promoting dependence on increasing guest concentration; **b. Mn1**/**V3** displaying a non-integer exchange-promoting dependence on increasing guest concentration; **c. $H_2$1**/**V1** displaying a zero-order dependence; **d. $H_2$2**/**V3** displaying a zero-order dependence.

2.1.2. T_1 fits Mn1/V1

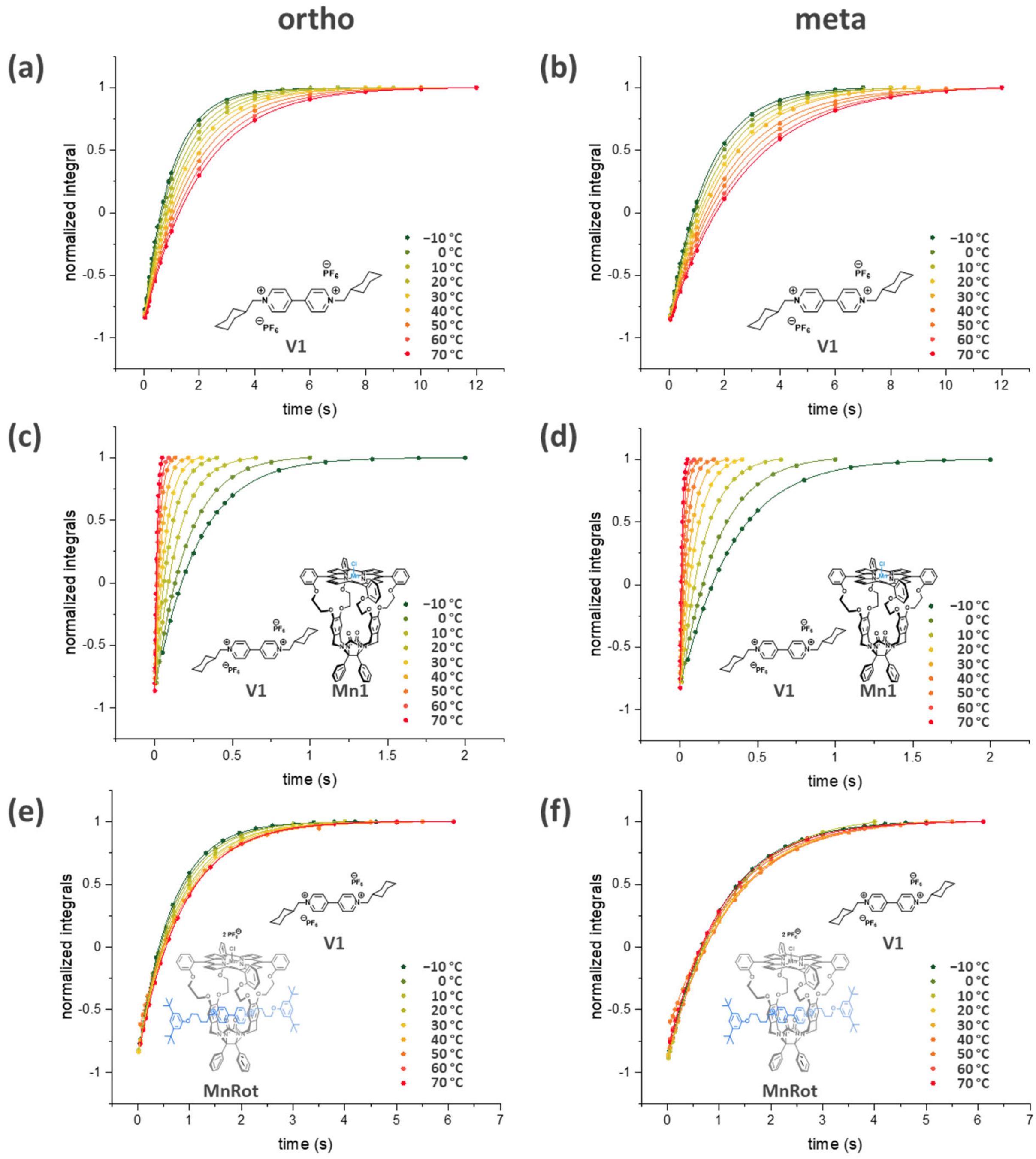

Supplementary Figure 3. Mono-exponential T_1 fits obtained through inverse recovery experiments (chloroform-*d* : acetonitrile-*d₃*,1:1.) a. ortho protons of 4,4'-bipyridyl moiety of **V1** (5 mM), giving $R_{1,0}$, **b.** meta protons of 4,4'-bipyridyl moiety of **V1** (5 mM), giving $R_{1,0}$, **c.** ortho protons of 4,4'-bipyridyl moiety of **V1** (5 mM) and **Mn1** (0.5 mM), giving $R_{1,obs}$, **d.** meta protons of 4,4'-bipyridyl moiety of **V1** (5 mM) and **Mn1** (0.5 mM), giving $R_{1,obs}$, **e.** ortho protons of 4,4'-bipyridyl moiety of **V1** (5 mM) and **MnRot** (0.5 mM), giving $R_{1,os}$, **f.** ortho protons of 4,4'-bipyridyl moiety of **V1** (5 mM) and **MnRot** (0.5 mM), giving $R_{1,os}$.

Supplementary Table 8. Tabulated values of the observed longitudinal rate constants ($R_{1,obs}$), the natural longitudinal rate constants ($R_{1,0}$), the outer-sphere longitudinal rate constants ($R_{1,os}$) and the rate constants of dissociation based off the R_1 experiments (k_d) after correcting for the mol fraction of manganese, the population of bound species and the exchange-promoting ligand dependence for which the non-integer order dependence of 0.32 was used (see Supplementary Figure 2a).

**Temp. (°C)**	**$R_{1,obs}$ (s^{-1}) ortho**	**$R_{1,obs}$ (s^{-1}) meta**	**$R_{1,0}$ (s^{-1}) ortho**	**$R_{1,0}$ (s^{-1}) meta**	**$R_{1,os}$ (s^{-1}) ortho**	**$R_{1,os}$ (s^{-1}) meta**	**k_d (s^{-1}) ortho**	**k_d (s^{-1}) meta**
**−10**	3.62	3.00	1.01	0.72	1.53	0.97	$6.9 \cdot 10^1$	$8.5 \cdot 10^1$
**0**	4.82	4.19	0.95	0.67	1.45	0.93	$1.6 \cdot 10^2$	$1.7 \cdot 10^2$
**10**	7.13	6.60	0.84	0.61	1.37	0.89	$3.1 \cdot 10^2$	$3.3 \cdot 10^2$
**20**	10.8	10.3	0.77	0.55	1.30	0.86	$5.6 \cdot 10^2$	$5.7 \cdot 10^2$
**30**	15.3	15.3	0.71	0.52	1.26	0.85	$8.5 \cdot 10^2$	$8.9 \cdot 10^2$
**40**	23.5	23.9	0.65	0.48	1.20	0.83	$1.4 \cdot 10^3$	$1.4 \cdot 10^3$
**50**	35.3	37.2	0.59	0.43	1.11	0.78	$2.2 \cdot 10^3$	$2.3 \cdot 10^3$
**60**	50.7	54.9	0.53	0.40	1.18	0.89	$3.1 \cdot 10^3$	$3.4 \cdot 10^3$
**70**	68.3	80.7	0.49	0.37	1.16	0.94	$4.3 \cdot 10^3$	$5.1 \cdot 10^3$

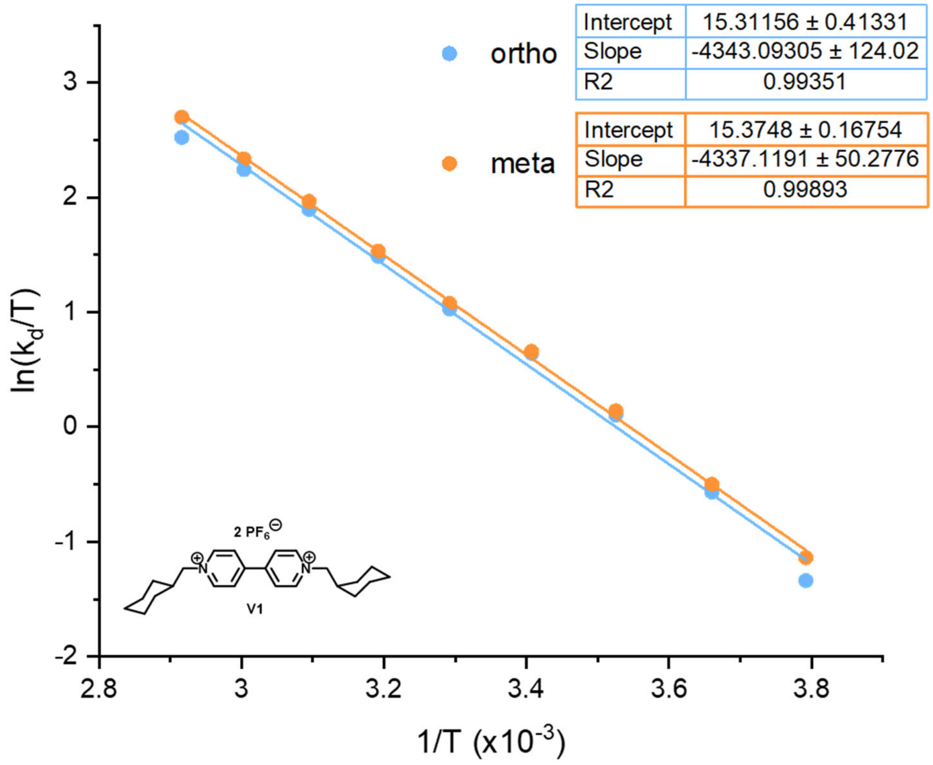

Supplementary Figure 4: Eyring plot from T_1, in which $\ln(k_d/T)$ is plotted against the inverse temperature for host-guest complex Mn1/V1.

2.1.3. T_2 PROJECT-CPMG fits Mn1/V1

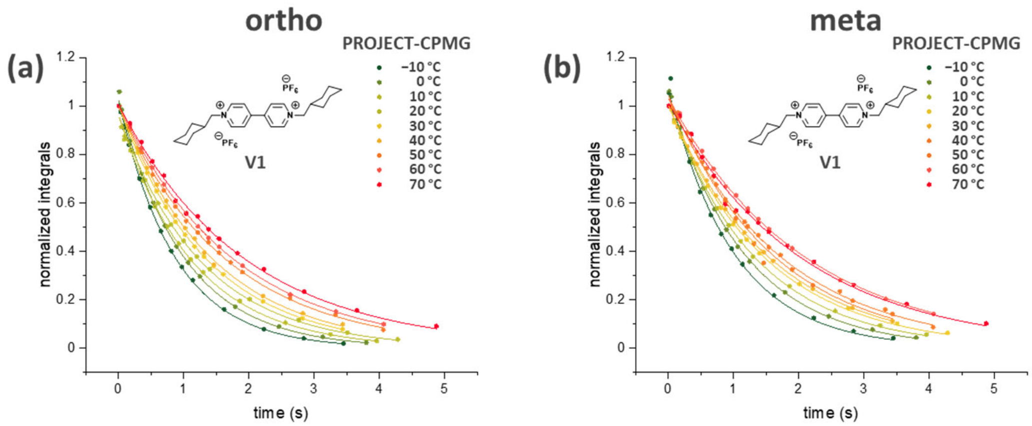

Supplementary Figure 5. T_2 fits obtained through PROJECT-CPMG experiments. Solvent chloroform-*d* : acetonitrile-*d_3* (1:1, v/v) **a.** Ortho protons of 4,4'-bipyridyl moiety of **V1** (5 mM), giving $R_{2,0}$, **b.** meta protons of 4,4'-bipyridyl moiety of **V1** (5 mM), giving $R_{2,0}$.

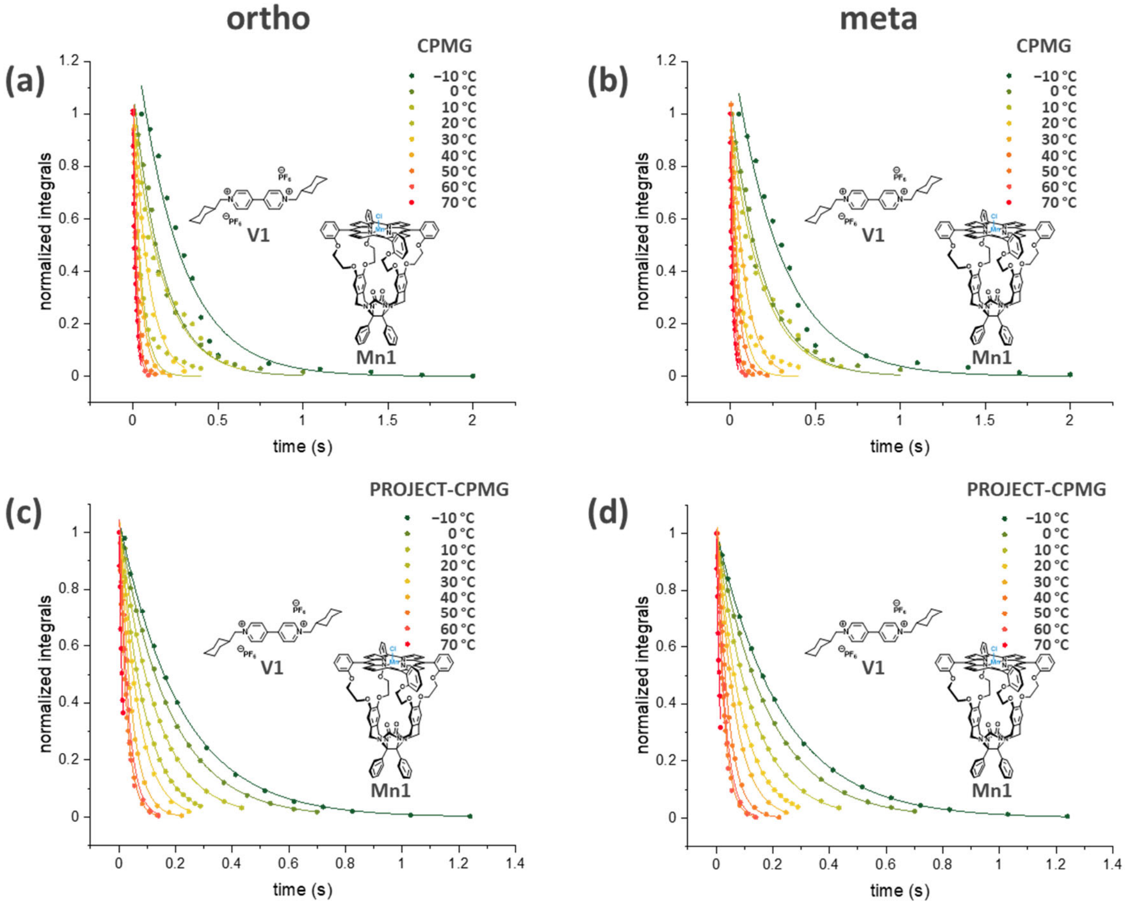

Supplementary Figure 6. T_2 fits obtained through CPMG and PROJECT-CPMG experiments. Solvent chloroform-*d* : acetonitrile-*d*$_3$ (1:1, v/v). **a.** Fits from CPMG experiments of ortho protons of 4,4'-bipyridyl moiety of **V1** (5 mM) and **Mn1** (0.5 mM), giving $R_{2,obs}$, **b.** fits from CPMG experiments of meta protons of 4,4'-bipyridyl moiety of **V1** (5 mM) and **Mn1** (0.5 mM), giving $R_{2,obs}$, **c.** fits from PROJECT-CPMG experiments of ortho protons of 4,4'-bipyridyl moiety of **V1** (5 mM) and **Mn1** (0.5 mM), giving $R_{2,obs}$, **d.** fits from PROJECT-CPMG experiments of meta protons of 4,4'-bipyridyl moiety of **V1** (5 mM) and **Mn1** (0.5 mM), giving $R_{2,obs}$.

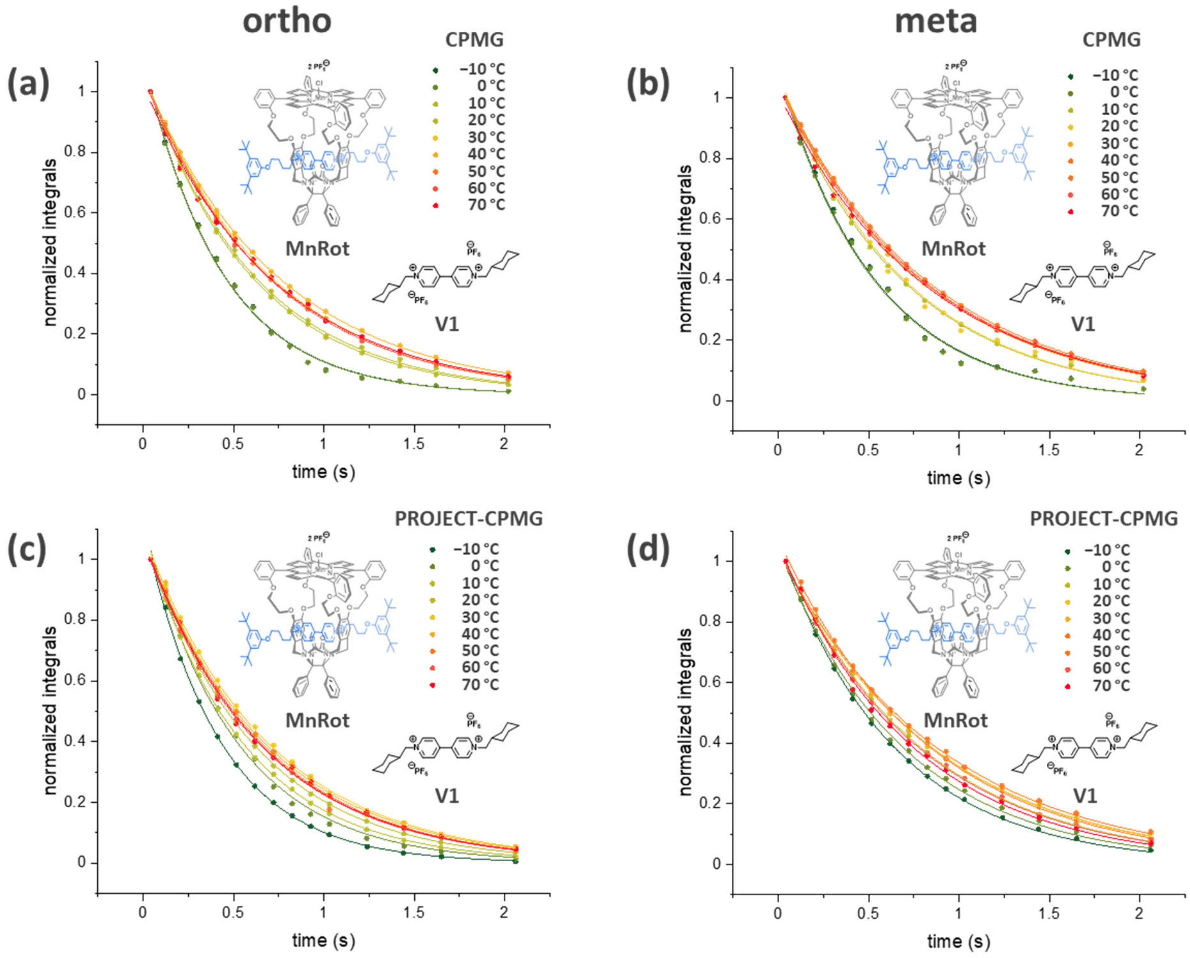

Supplementary Figure 7. T_2 fits obtained through CPMG and PROJECT-CPMG experiments. Solvent chloroform-*d* : acetonitrile-*d*$_3$ (1:1, v/v). **a.** Fits from CPMG experiments of ortho protons of 4,4'-bipyridyl moiety of **V1** (5 mM) and **MnRot** (0.5 mM), giving $R_{2,obs}$, **b.** fits from CPMG experiments of meta protons of 4,4'-bipyridyl moiety of **V1** (5 mM) and **MnRot** (0.5 mM), giving $R_{2,obs}$, **c.** fits from PROJECT-CPMG experiments of ortho protons of 4,4'-bipyridyl moiety of **V1** (5 mM) and **MnRot** (0.5 mM), giving $R_{2,obs}$, **d.** fits from PROJECT-CPMG experiments of meta protons of 4,4'-bipyridyl moiety of **V1** (5 mM) and **MnRot** (0.5 mM), giving $R_{2,obs}$.

Supplementary Table 9. Tabulated values of the observed longitudinal rate constants ($R_{2,obs}$), the natural longitudinal rate constants ($R_{2,0}$), the outer-sphere longitudinal rate constants ($R_{2,os}$) and the rate constants of dissociation based off the R_2 experiments (k_d) after correcting for the mol fraction of manganese, the population of bound species and the exchange-promoting ligand dependence for which the non-integer order dependence of 0.32 was used (see Supplementary Figure 2a).

**Temp. (°C)**	**$R_{2,obs}$ (s^{-1}) ortho**	**$R_{2,obs}$ (s^{-1}) meta**	**$R_{2,0}$ (s^{-1}) ortho**	**$R_{2,0}$ (s^{-1}) meta**	**$R_{2,os}$ (s^{-1}) ortho**	**$R_{2,os}$ (s^{-1}) meta**	**k_d (s^{-1}) ortho**	**k_d (s^{-1}) meta**
**−10**	4.86	4.31	1.07	0.98	2.32	1.78	$9.4 \cdot 10^1$	$9.9 \cdot 10^1$
**0**	5.89	5.42	0.97	0.86	2.07	1.75	$1.8 \cdot 10^2$	$1.8 \cdot 10^2$
**10**	8.15	7.86	0.82	0.75	1.75	1.41	$3.6 \cdot 10^2$	$3.6 \cdot 10^2$
**20**	11.85	11.67	0.76	0.68	1.69	1.39	$6.0 \cdot 10^2$	$6.2 \cdot 10^2$
**30**	16.07	16.58	0.75	0.64	1.38	1.28	$8.9 \cdot 10^2$	$9.4 \cdot 10^2$
**40**	24.74	25.15	0.67	0.61	1.46	1.22	$1.4 \cdot 10^3$	$1.5 \cdot 10^3$
**50**	36.58	36.63	0.60	0.57	1.48	1.20	$2.2 \cdot 10^3$	$2.2 \cdot 10^3$
**60**	52.45	55.06	0.53	0.47	1.54	1.30	$3.2 \cdot 10^3$	$3.4 \cdot 10^3$
**70**	66.43	74.88	0.51	0.49	1.53	1.32	$4.1 \cdot 10^3$	$4.7 \cdot 10^3$

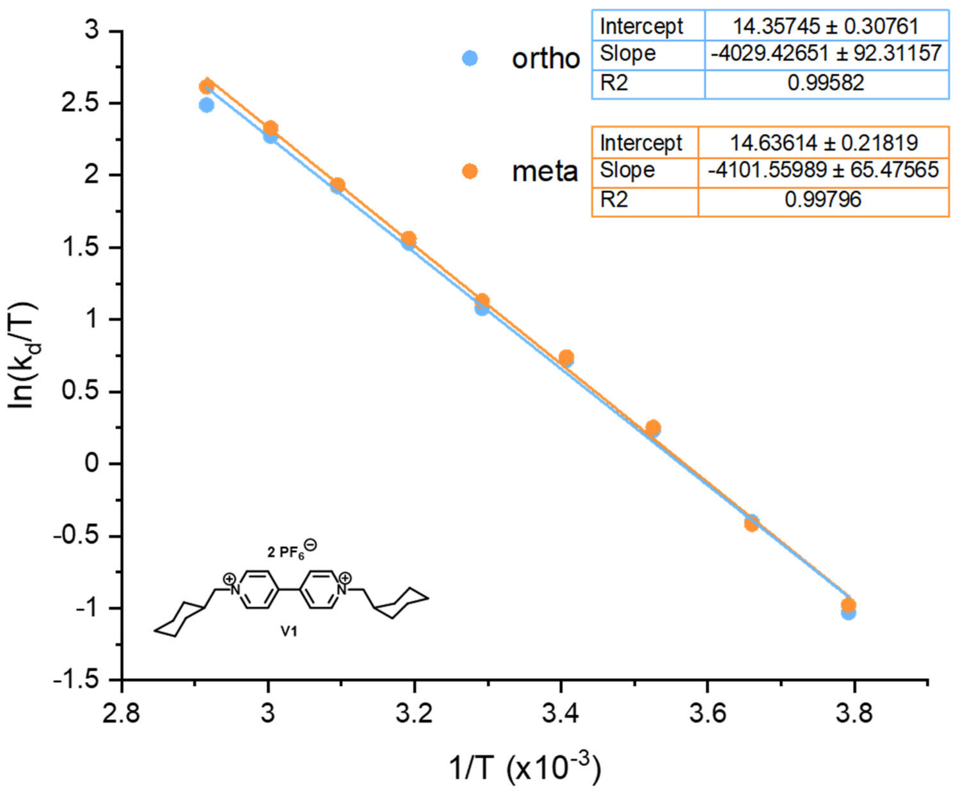

Supplementary Figure 8. Eyring plot from T_2 in which $\ln(k_d/T)$ is plotted against the inverse temperature for host-guest complex Mn1/V1.

2.1.4. T_1 fits Mn1/VP

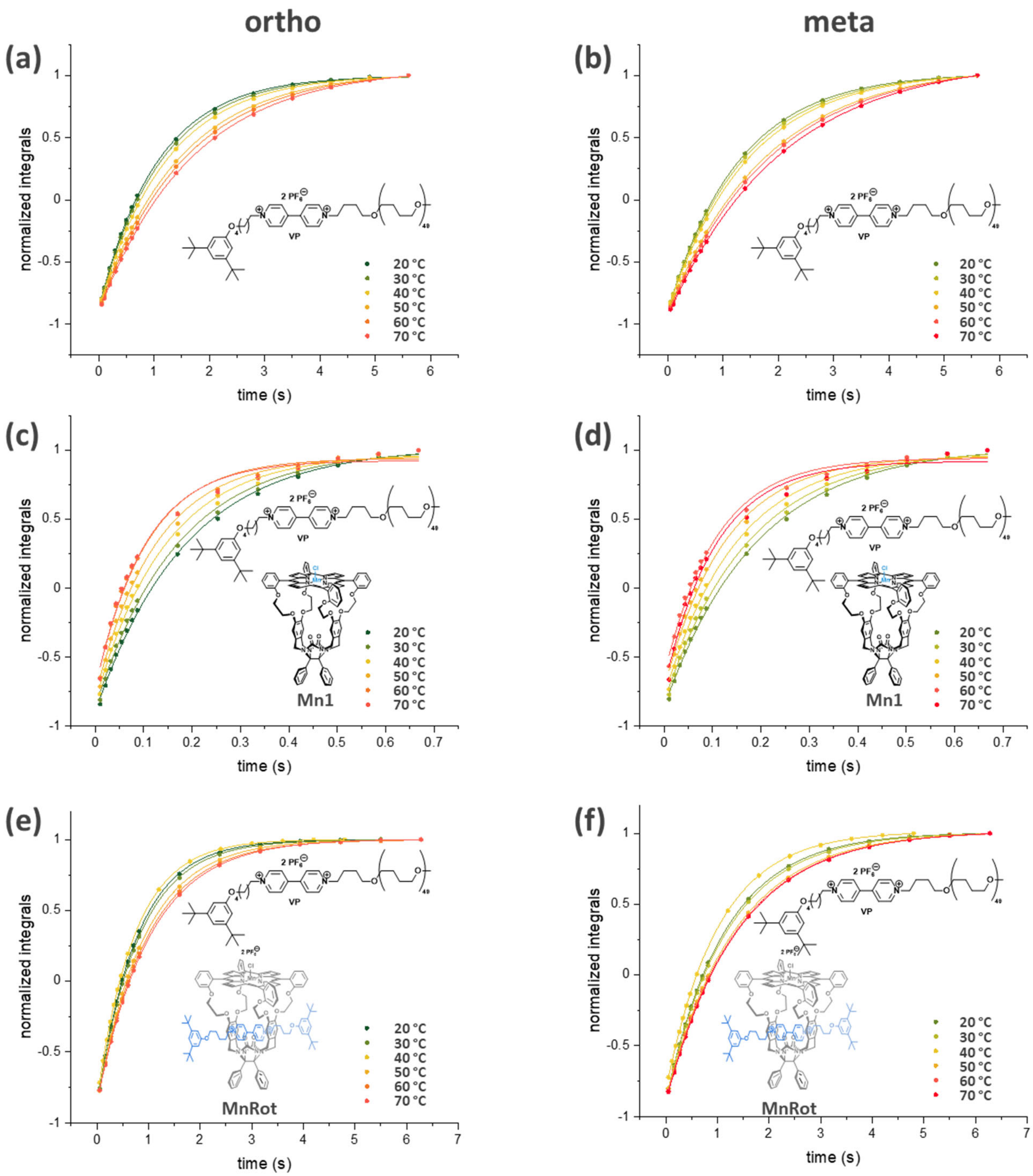

Supplementary Figure 9. Mono-exponential T_1 fits obtained through inverse recovery experiments. Solvent chloroform-d : acetonitrile-d$_3$ (1:1, v/v). **a.** Ortho protons of 4,4'-bipyridyl moiety of **VP** (5 mM), giving $R_{1,0}$, **b.** meta protons of 4,4'-bipyridyl moiety of **VP** (5 mM), giving $R_{1,0}$, **c.** ortho protons of 4,4'-bipyridyl moiety of **VP** (5 mM) and **Mn1** (0.5 mM), giving $R_{1,obs}$, **d.** meta

protons of 4,4'-bipyridyl moiety of **VP** (5 mM) and **Mn1** (0.5 mM), giving $R_{1,obs}$, **e.** ortho protons of 4,4'-bipyridyl moiety of **VP** (5 mM) and **MnRot** (0.5 mM), giving $R_{1,os}$, **f.** ortho protons of 4,4'-bipyridyl moiety of **VP** (5 mM) and **MnRot** (0.5 mM), giving $R_{1,os}$.

Supplementary Table 10. Tabulated values of the observed longitudinal rate constants ($R_{1,obs}$), the natural longitudinal rate constants ($R_{1,0}$), the outer-sphere longitudinal rate constants ($R_{1,os}$) and the rate constants of dissociation based off the R_1 experiments (k_d) after correcting for the mol fraction of manganese, the population of bound species and the exchange-promoting ligand dependence for which the non-integer order dependence of 0.32 was used (see Supplementary Figure 2a).

**Temp. (°C)**	**$R_{1,obs}$ (s^{-1}) ortho**	**$R_{1,obs}$ (s^{-1}) meta**	**$R_{1,0}$ (s^{-1}) ortho**	**$R_{1,0}$ (s^{-1}) meta**	**$R_{1,os}$ (s^{-1}) ortho**	**$R_{1,os}$ (s^{-1}) meta**	**k_d (s^{-1}) ortho**	**k_d (s^{-1}) meta**
**20**	5.36	5.23	0.94	0.77	0.77	1.15	$2.7{\cdot}10^2$	$2.4{\cdot}10^2$
**30**	5.91	5.79	0.89	0.73	0.82	1.19	$3.1{\cdot}10^2$	$2.8{\cdot}10^2$
**40**	6.94	6.76	0.82	0.69	0.86	1.24	$3.8{\cdot}10^2$	$3.5{\cdot}10^2$
**50**	8.03	7.97	0.69	0.56	0.92	1.35	$4.7{\cdot}10^2$	$4.4{\cdot}10^2$
**60**	9.37	9.35	0.65	0.53	0.98	1.38	$5.6{\cdot}10^2$	$5.4{\cdot}10^2$
**70**	11.94	12.13	0.59	0.49	1.03	1.40	$7.5{\cdot}10^2$	$7.5{\cdot}10^2$

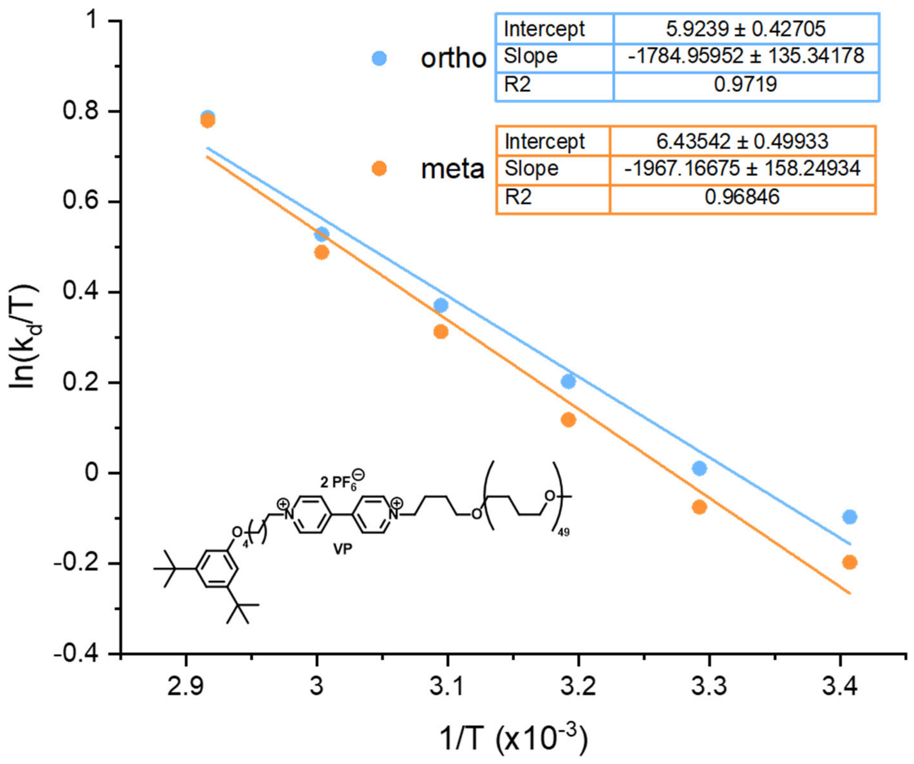

Supplementary Figure 10. Eyring plot from T_1 in which $\ln(k_d/T)$ is plotted against the inverse temperature for host-guest complex Mn1/VP.

2.1.5. T_2 fits Mn1/VP

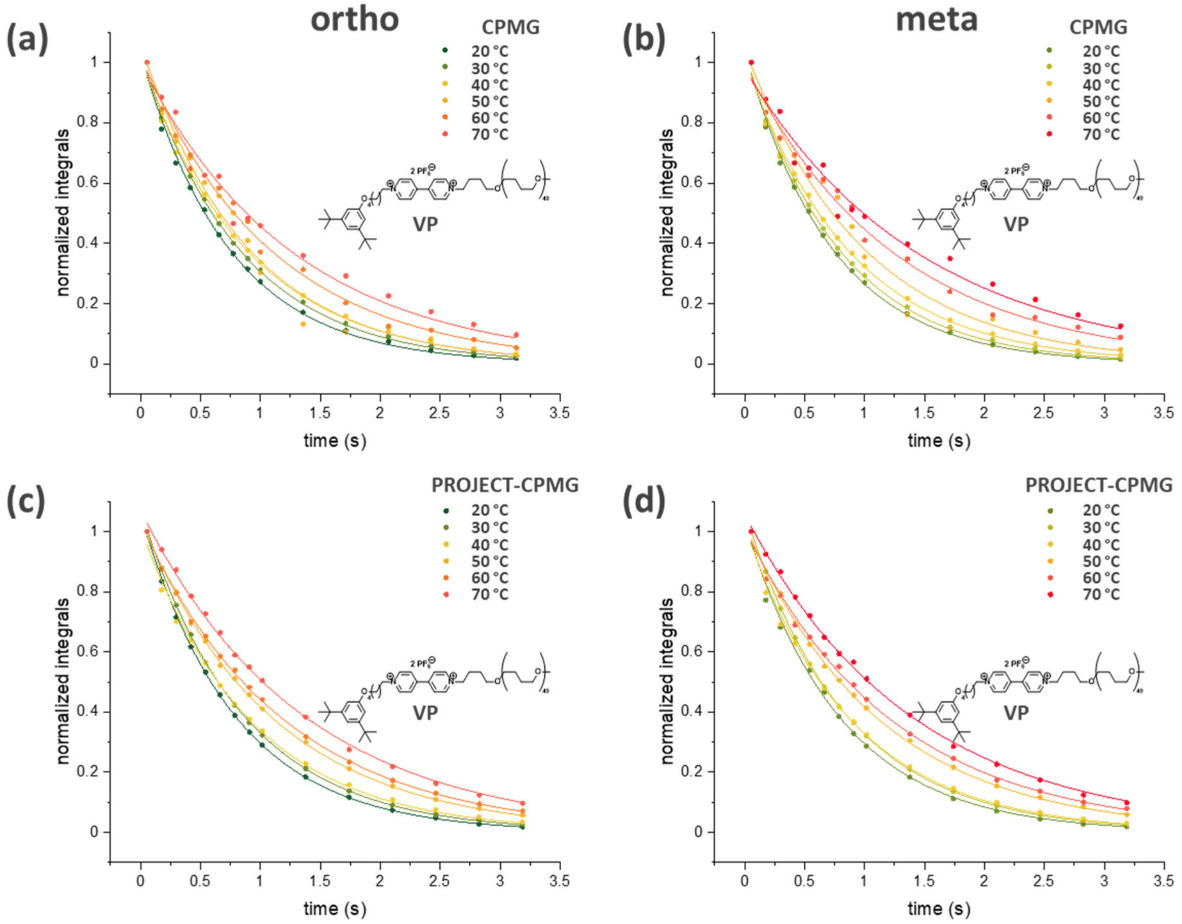

Supplementary Figure 11. T_2 fits obtained through CPMG and PROJECT-CPMG experiments. Solvent chloroform-*d* : acetonitrile-*d*$_3$ (1:1, v/v). **a.** Fits from CPMG experiments of ortho protons of 4,4’-bipyridyl moiety of **VP** (5 mM), giving $R_{2,0}$, **b.** fits from CPMG experiments of meta protons of 4,4’-bipyridyl moiety of **VP** (5 mM), giving $R_{2,0}$, **c.** fits from PROJECT-CPMG experiments of ortho protons of 4,4’-bipyridyl moiety of **VP** (5 mM), giving $R_{2,0}$, **d.** fits from PROJECT-CPMG experiments of meta protons of 4,4’-bipyridyl moiety of **V1** (5 mM), giving $R_{2,0}$.

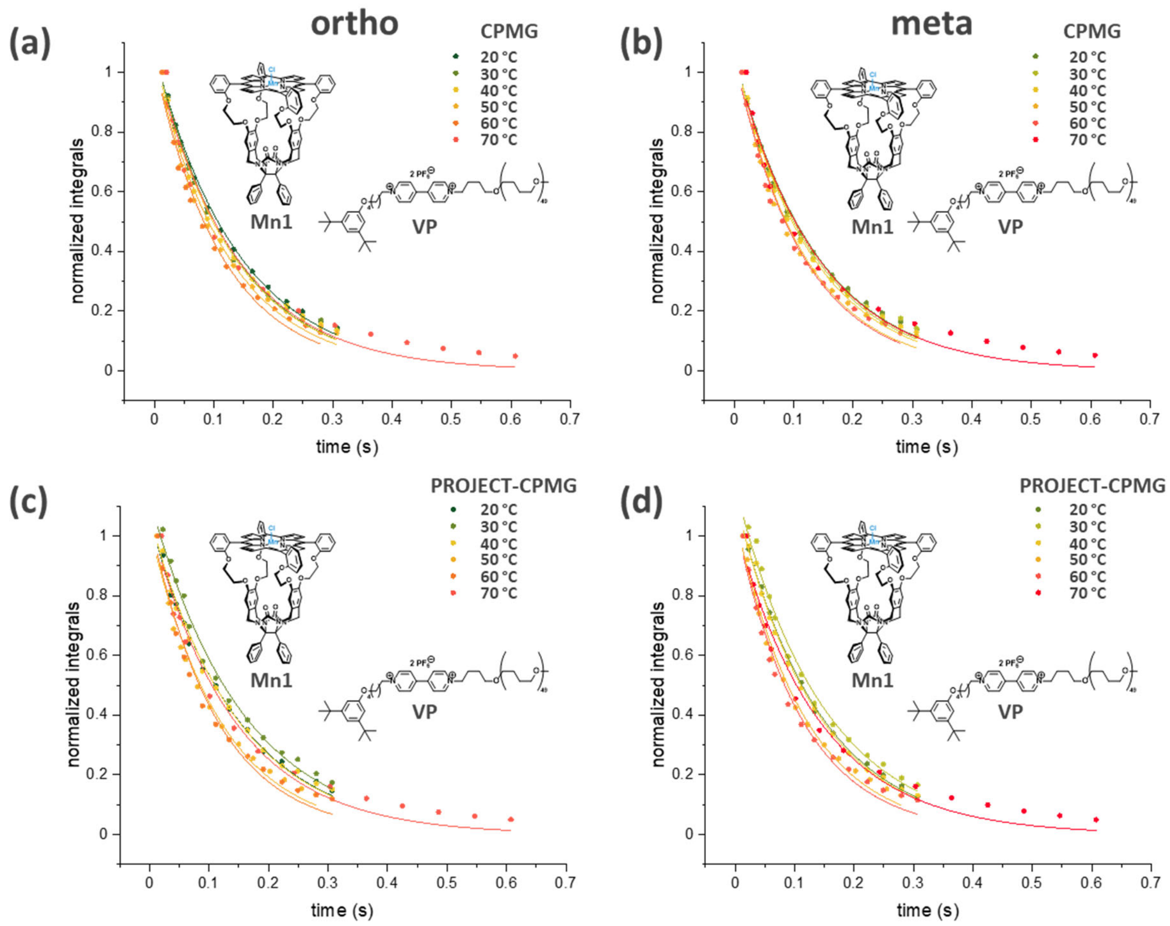

Supplementary Figure 12. T_2 fits obtained through CPMG and PROJECT-CPMG experiments. Solvent chloroform-*d* : acetonitrile-d_3 (1:1, v/v). **a.** Fits from CPMG experiments of ortho protons of 4,4'-bipyridyl moiety of **VP** (5 mM) and **Mn1** (0.5 mM), giving $R_{2,obs}$, **b.** fits from CPMG experiments of meta protons of 4,4'-bipyridyl moiety of **VP** (5 mM) and **Mn1** (0.5 mM), giving $R_{2,obs}$, **c.** fits from PROJECT-CPMG experiments of ortho protons of 4,4'-bipyridyl moiety of **VP** (5 mM) and **Mn1** (0.5 mM), giving $R_{2,obs}$, **d.** fits from PROJECT-CPMG experiments of meta protons of 4,4'-bipyridyl moiety of **VP** (5 mM) and **Mn1** (0.5 mM), giving $R_{2,obs}$.

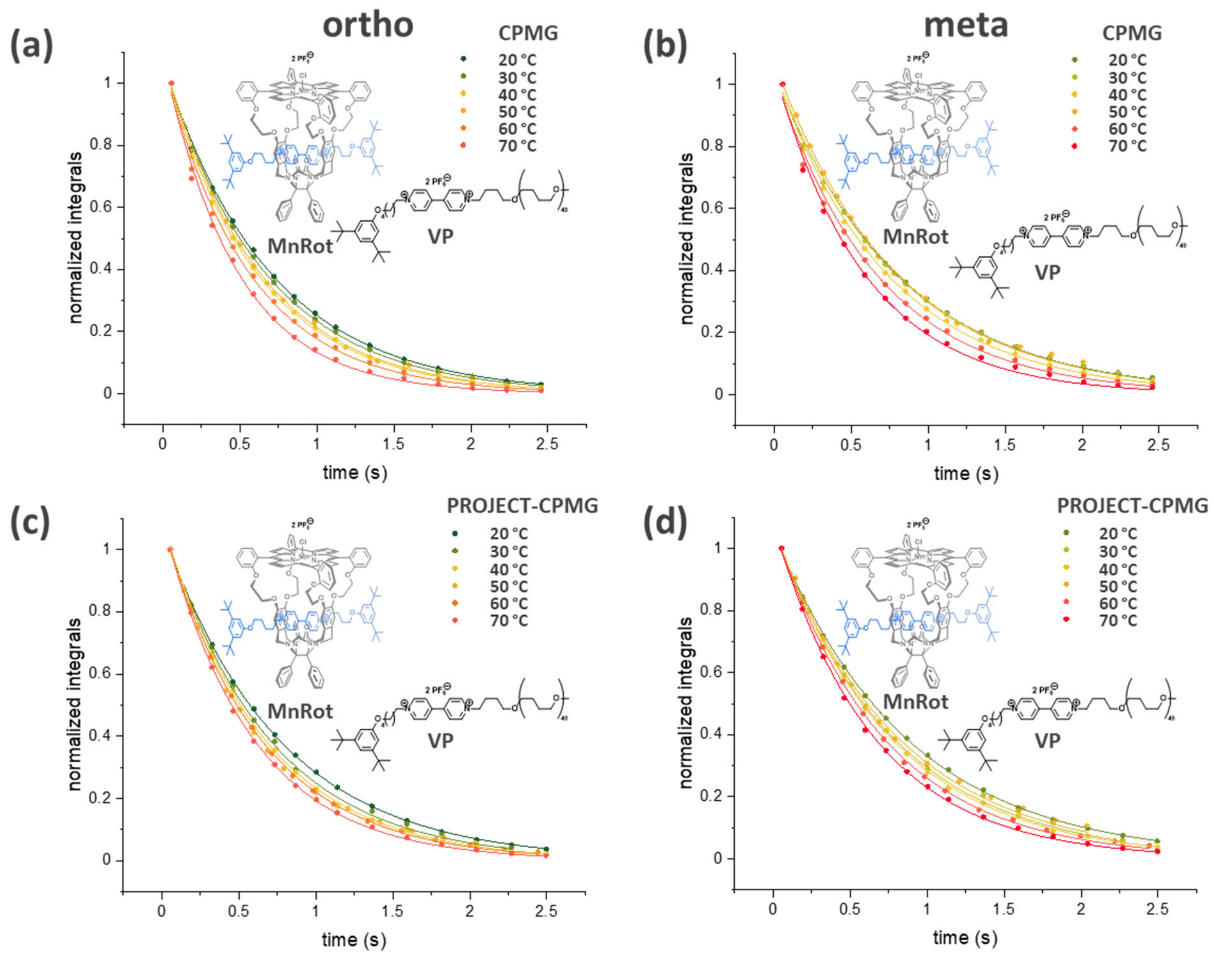

Supplementary Figure 13. T_2 fits obtained through CPMG and PROJECT-CPMG experiments. Solvent chloroform-*d* : acetonitrile-*d*$_3$ (1:1, v/v)**. a.** Fits from CPMG experiments of ortho protons of 4,4'-bipyridyl moiety of **VP** (5 mM) and **MnRot** (0.5 mM), giving $R_{2,obs}$, **b.** fits from CPMG experiments of meta protons of 4,4'-bipyridyl moiety of **VP** (5 mM) and **MnRot** (0.5 mM), giving $R_{2,obs}$, **c.** fits from PROJECT-CPMG experiments of ortho protons of 4,4'-bipyridyl moiety of **VP** (5 mM) and **MnRot** (0.5 mM), giving $R_{2,obs}$, **d.** fits from PROJECT-CPMG experiments of meta protons of 4,4'-bipyridyl moiety of **VP** (5 mM) and **MnRot** (0.5 mM), giving $R_{2,obs}$.

Supplementary Table 11. Tabulated values of the observed longitudinal rate constants ($R_{2,obs}$), the natural longitudinal rate constants ($R_{2,0}$), the outer-sphere longitudinal rate constants ($R_{2,os}$) and the rate constants of dissociation based off the R_2 experiments (k_d) after correcting for the mol fraction of manganese and the population of bound species.

**Temp. (°C)**	**$R_{2,obs}$ (s^{-1}) ortho**	**$R_{2,obs}$ (s^{-1}) meta**	**$R_{2,0}$ (s^{-1}) ortho**	**$R_{2,0}$ (s^{-1}) meta**	**$R_{2,os}$ (s^{-1}) ortho**	**$R_{2,os}$ (s^{-1}) meta**	**k_d (s^{-1}) ortho**	**k_d (s^{-1}) meta**
**20**	6.95	7.21	1.31	1.31	2.01	1.67	$2.6 \cdot 10^2$	$3.1 \cdot 10^2$

**30**	7.04	7.12	1.20	1.23	1.92	1.62	$2.9 \cdot 10^2$	$3.1 \cdot 10^2$
**40**	7.24	7.54	1.11	1.14	1.70	1.45	$3.2 \cdot 10^2$	$3.6 \cdot 10^2$
**50**	8.21	8.52	1.02	0.95	1.61	1.34	$4.1 \cdot 10^2$	$4.5 \cdot 10^2$
**60**	8.84	8.82	0.88	0.81	1.49	1.27	$4.7 \cdot 10^2$	$4.9 \cdot 10^2$
**70**	10.02	10.83	0.76	0.70	1.38	1.20	$5.7 \cdot 10^2$	$6.5 \cdot 10^2$

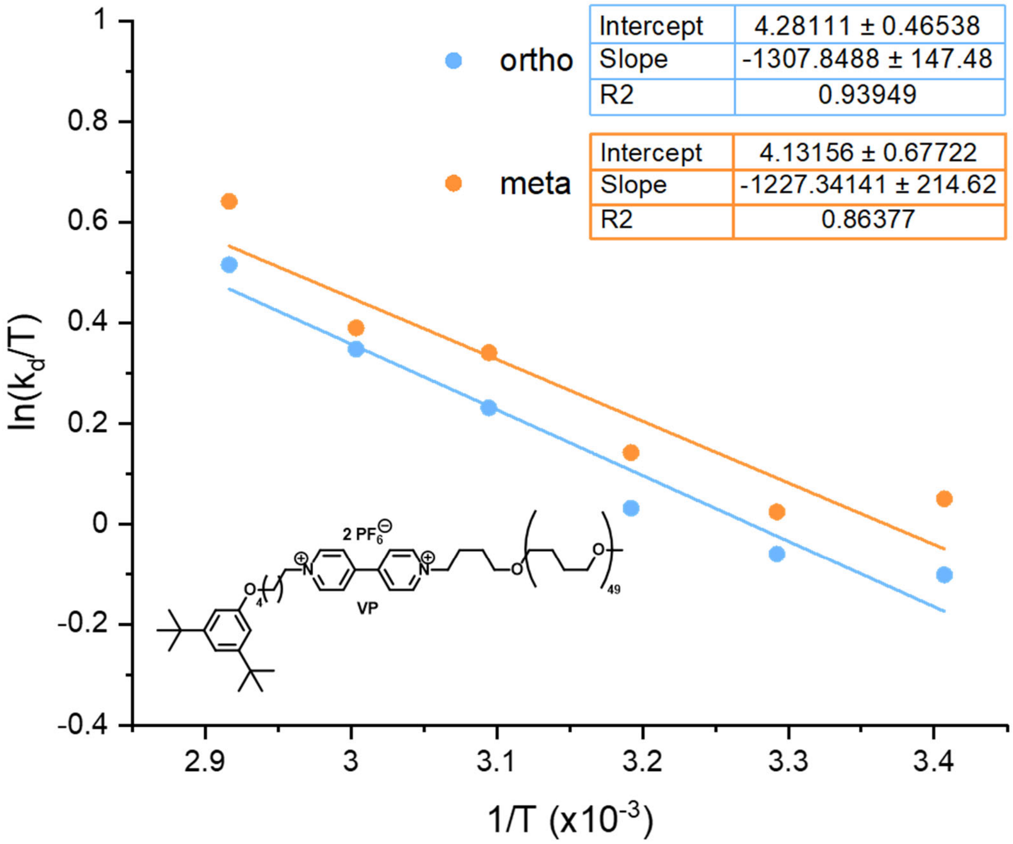

Supplementary Figure 14. Eyring plot from T_2 in which $\ln(k_d/T)$ is plotted against the inverse temperature for host-guest complex Mn1/VP.

2.1.6. T_1 fits Mn1/V2

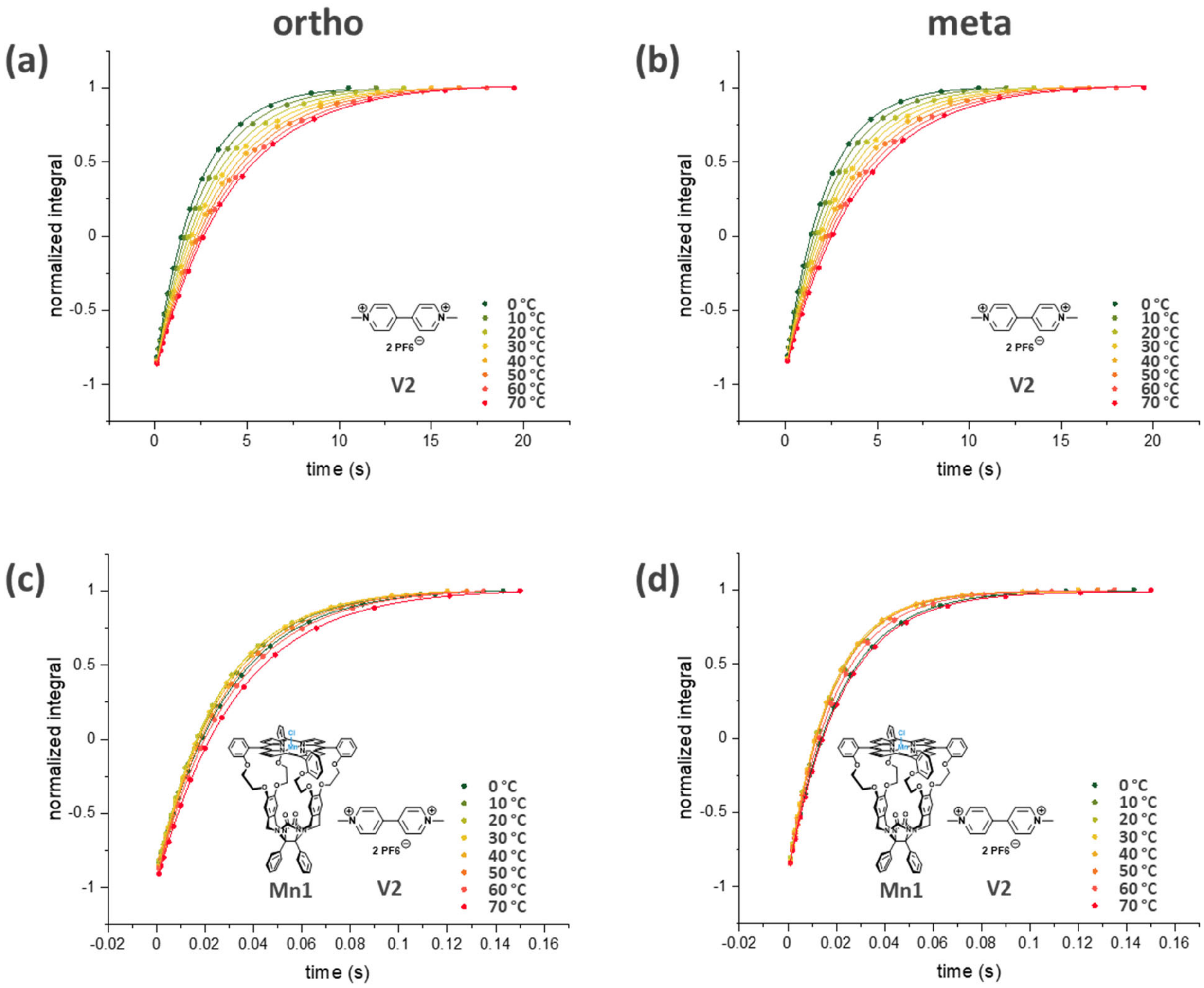

Supplementary Figure 15. Mono-exponential T_1 fits obtained through inverse recovery experiments. Solvent chloroform-d : acetonitrile-d_3 (1:1, v/v). **a.** Ortho protons of 4,4’-bipyridyl moiety of **V2** (5 mM), giving $R_{1,0}$, **b.** meta protons of 4,4’-bipyridyl moiety of **V2** (5 mM), giving $R_{1,0}$, **c.** ortho protons of 4,4’-bipyridyl moiety of **V2** (5 mM) and **Mn1** (0.05 mM), giving $R_{1,obs}$, **d.** meta protons of 4,4’-bipyridyl moiety of **V2** (5 mM) and **Mn1** (0.5 mM), giving $R_{1,obs}$.

2.1.7. Determining dissociation parameters of Mn1/V2

When measuring $R_{1,p}$ of **Mn1/V2** and plotting these parameters against the measurable temperature range, it was evident that this system followed the dynamics of the fast exchange regime (Supplementary Figure 16c). When the exchange regime transitions from slow to intermediate to fast, the curve resulting from plotting R_p against temperature starts flattening ($\tau_M \approx T_{1M}$) before the slope becomes

negative ($\tau_M < T_{1M}$). This is in stark contrast to the slow-exchange curvature observed for **Mn1**/**V1** and **Mn1**/**VP** where $k_{d,obs}$ could be easily determined (Supplementary Figure 16a-b). As T_{1M} begins to play a larger role, a noticeable difference between the $R_{1,p}$ for the *ortho* and *meta* ^{1}Hs is also observed, resulting from the closer proximity of the *meta* protons to the metal center giving shorter $T_{1,M}$ values. As mentioned before, $T_{1,M}$ dominates in this regime and therefore Eq. S5 cannot be used to calculate $k_{d,obs}$. However, by measuring T_1 values for the **Mn1**/**V2** complex in the $T_{1,M}$-dominating temperature window (50 – 70 °C), the exponential nature of $T_{1,M}$ was utilized to calculate $T_{1,M}$ values where $\tau_M \geq T_M$ (Supplementary Table 12 and S13), allowing the construction of an Eyring plot to extract the exchange parameters (p40-41). Similar $\Delta H^{\ddagger}$ values for the dissociation of **Mn1**/**V2** (7.99 ± 1.08 kcal·mol^{-1}) compared to that of **Mn1**/**V1** (8.35 ± 0.17 kcal·mol^{-1}) were found, rejecting the hypothesis that less bulky substituents on the viologen guest result in lower activation enthalpies. Hence, the appreciable difference in dissociation $\Delta H^{\ddagger}$ values measured for **Mn1**/**V1** and **Mn1**/**VP** is possibly caused by an increase in statistical host positions on the polymer chain, as opposed to **Mn1**/**V1** where the viologen moiety is deeply bound inside the cavity of its host.

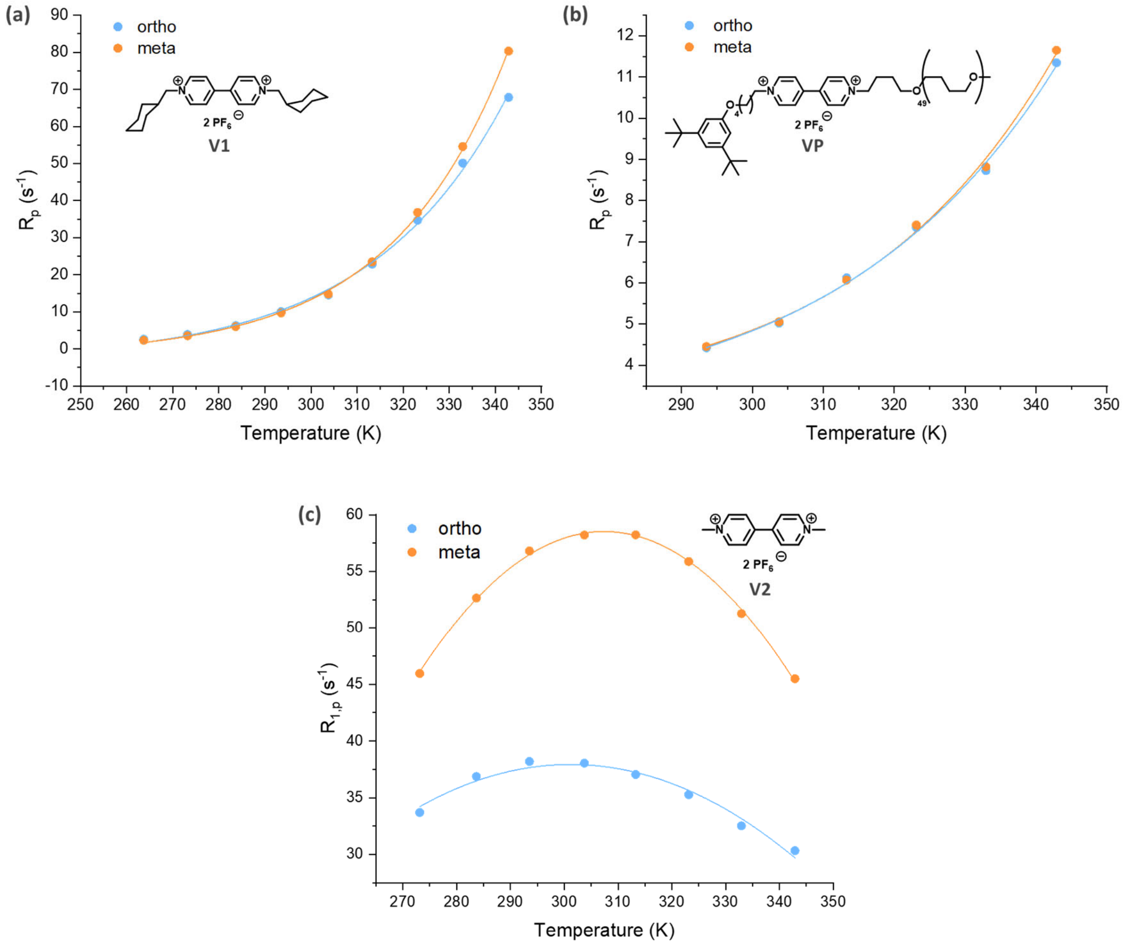

Supplementary Figure 16: **Exchange regimes for Mn1/V1, Mn1/VP, and Mn1/V2. a,** $R_{1,p}$ values of the ortho and meta protons of **V1** (s^{-1}) plotted against the temperature, showing the presence of a slow to intermediate exchange regime. **b**, $R_{1,p}$ values of the ortho and meta protons of **VP** (s^{-1}) plotted against the temperature, showing a slow to medium exchange regime. **c**, $R_{1,p}$ values of the ortho and meta protons of **V2** (s^{-1}) plotted against the temperature, showing a fast exchange regime.

A much lower dissociation entropy cost was found for **Mn1/V2** ($\Delta S^{\ddagger}$ = −5.60 ± 0.35 cal·K^{-1}·mol−1) than for **Mn1/V1** ($\Delta S^{\ddagger}$ = −17.57 ± 0.34 cal·K^{-1}·mol^{-1}). This difference in $\Delta S^{\ddagger}$ may be explained by an increase in steric effects induced by the bulkier methylcyclohexyl side groups of **V1**, thus requiring a higher order of organization of the complex for dissociation than in the case of the complex between **Mn1** and **V2**. In previous studies, the same trend was observed for the dissociation of similar guest compounds in which those with less bulky side groups gave less negative $\Delta S^{\ddagger}$ values[3].

2.1.8. Extracting $T_{1,M}$

When the change in $R_{1,p}$ over an increasing temperature range is negative, the relaxation of **V2** situated closely to the metal center is dominant ($R_{1,M}$) (Supplementary Figure 17). When $R_{1,M}$ is dominating, the value can be calculated by utilizing Supplementary Equation 25.

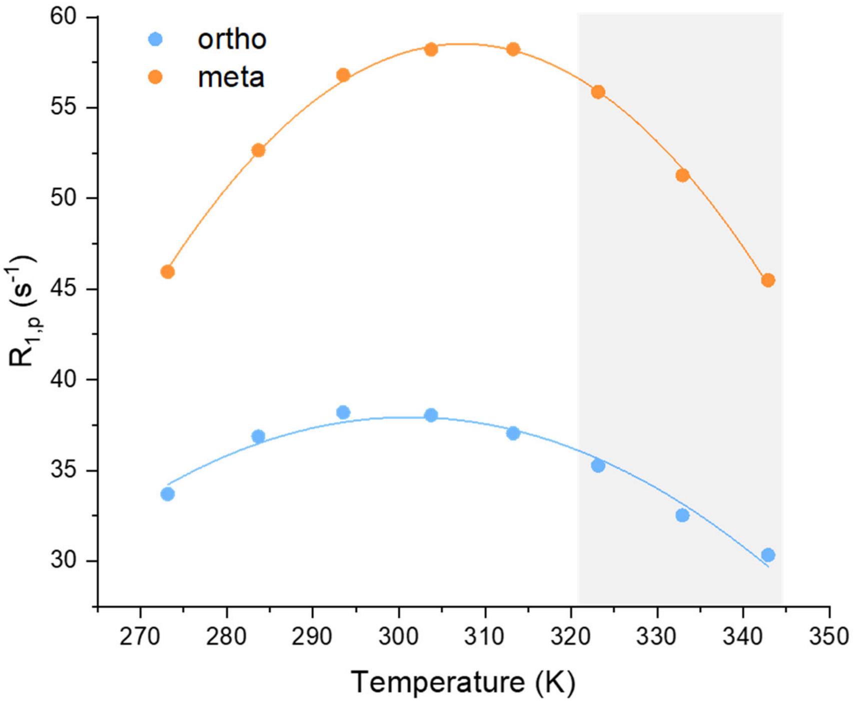

Supplementary Figure 17. The $R_{1,p}$ (s^{-1}) plotted against the temperature (K) for the ortho-protons (blue) and meta-protons (orange). The temperature range which yielded a negative slope for the change in $R_{1,p}$ is indicated in grey.

$$R_{1,obs} = \chi_{bound\ guest} \cdot R_{1,M} + \chi_{free\ guest} \cdot R_{1,0}$$ Supplementary Equation 25

In which $\chi_{bound\ ligand}$ and $\chi_{free\ guest}$ are the mol fractions of bound and free guest, respectively. Because of the exponential relationship between $R_{1,M}$ and temperature, the values for $R_{1,M}$ could be extrapolated for the temperature points in which $R_{1,M}$ is not dominating (0 – 30 °C) and used in the calculation of the dissociation rate constant. Supplementary Figure 18 shows the natural log of the determined $T_{1,M}$ values plotted against temperature and fitted linearly.

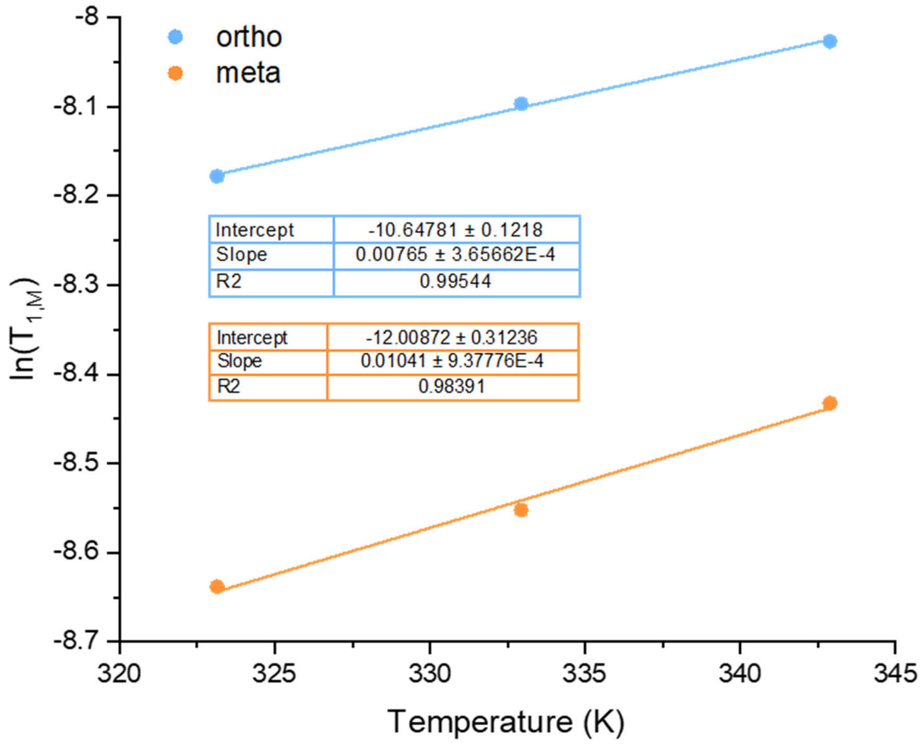

Supplementary Figure 18. Fits of the natural log of $T_{1,M}$ (s) plotted against the temperature (K).

Supplementary Table 12. Tabulated $T_{1,M}$ values extrapolated from the fits obtained in Supplementary Figure 18.

**Temp. (°C)**	**$T_{1,M}$ (μs) ortho**	**$T_{1,M}$ (μs) meta**
**0**	102	111
**10**	60	71
**20**	35	45
**30**	18	26

The calculated values for $T_{1,M}$ are consequently used to calculate the residence time of **V2** near or on the metal center (τ_M) using Supplementary Equations 26 and 27 and correcting for the percentage of host that is bound.

$$R_{1,p} = \frac{qp}{\tau_M + T_{1,M}} \quad \text{Supplementary Equation 26}$$

$$\tau_M = \frac{qp}{R_{1,p}} - T_{1,M}$$ Supplementary Equation 27

Supplementary Table 13. Tabulated values of the observed longitudinal rate constants ($R_{2,obs}$), the natural longitudinal rate constants ($R_{2,0}$), the outer-sphere longitudinal rate constants ($R_{2,os}$) and the rate constants of dissociation based off the R_2 experiments (k_d) after correcting for the mol fraction of manganese, the population of bound species and the exchange-promoting ligand dependence for which the non-integer order dependence of 0.32 was used (see Supplementary Figure 2a).

Temp. (°C)	$R_{1,obs}$ (s^{-1}) ortho	$R_{1,obs}$ (s^{-1}) meta	$R_{1,0}$ (s^{-1}) ortho	$R_{1,0}$ (s^{-1}) meta	$R_{1,os}$ (s^{-1}) ortho	$R_{1,os}$ (s^{-1}) meta	$k_{d,obs}$ (s^{-1}) ortho	$k_{d,obs}$ (s^{-1}) meta	k_d (s^{-1}) ortho	k_d (s^{-1}) meta
**0**	34.13	46.41	0.44	0.46	n.d.	n.d.	$9.8 \cdot 10^3$	$9.0 \cdot 10^3$	$5.5 \cdot 10^4$	$5.0 \cdot 10^4$
**10**	37.25	53.06	0.39	0.41	n.d.	n.d.	$1.7 \cdot 10^4$	$1.4 \cdot 10^4$	$9.2 \cdot 10^4$	$7.8 \cdot 10^4$
**20**	38.54	57.16	0.35	0.36	n.d.	n.d.	$2.9 \cdot 10^4$	$2.2 \cdot 10^4$	$1.6 \cdot 10^5$	$1.2 \cdot 10^5$
**30**	38.36	58.54	0.32	0.34	n.d.	n.d.	$5.7 \cdot 10^4$	$3.8 \cdot 10^4$	$3.1 \cdot 10^5$	$2.1 \cdot 10^5$
**40**	37.33	58.53	0.29	0.31	n.d.	n.d.				
**50**	35.54	56.16	0.27	0.28	n.d.	n.d.				
**60**	32.77	51.53	0.26	0.27	n.d.	n.d.				
**70**	30.56	45.74	0.24	0.25	n.d.	n.d.				

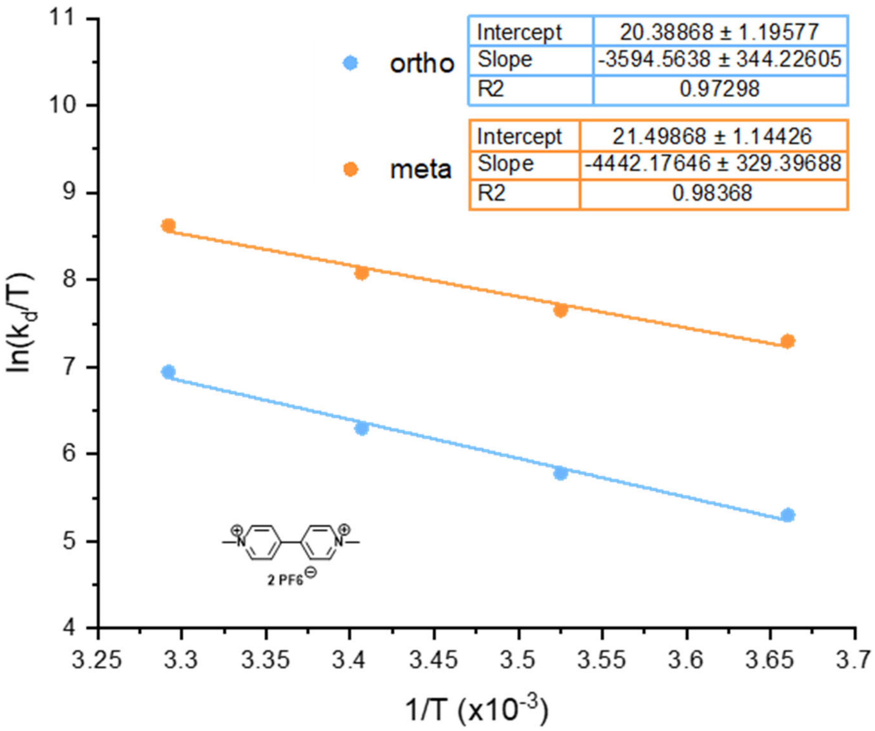

Supplementary Figure 19. Eyring plot from T_1 in which $\ln(k_d/T)$ is plotted against the inverse temperature for host-guest complex Mn1/V2.

2.1.9. All Eyring plots (Mn1/V1, Mn1/V2, Mn1/VP)

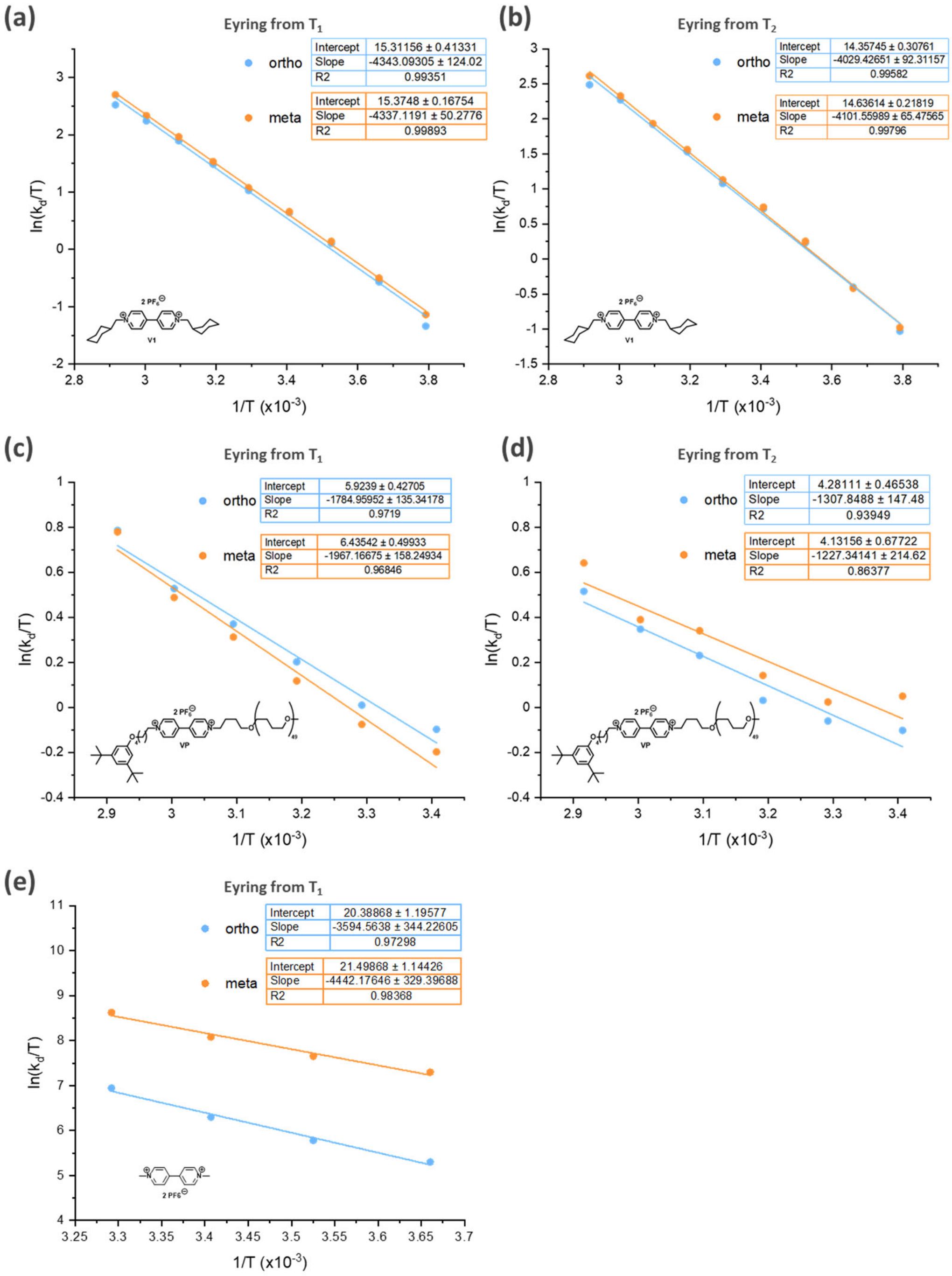

Supplementary Figure 20: **Eyring plots in which $\ln(k_d)$ is plotted against the inverse temperature. a,** Eyring plot for host-guest complex **Mn1**/**V1** determined from k_d obtained from T_1 measurements. **b,** Eyring plot for host-guest complex **Mn1**/**V1** determined

from k_d obtained from T_2 measurements. **c,** Eyring plot for host-guest complex **Mn1**/**VP** determined from k_d obtained from T_1 measurements. **d,** Eyring plot for host-guest complex **Mn1**/**VP** determined from k_d obtained from T_2 measurements. **e**, Eyring plot for host-guest complex **Mn1**/**V2** determined from k_d obtained from T_1 measurements.

2.2. NMR binding titrations

2.2.1. Binding curves

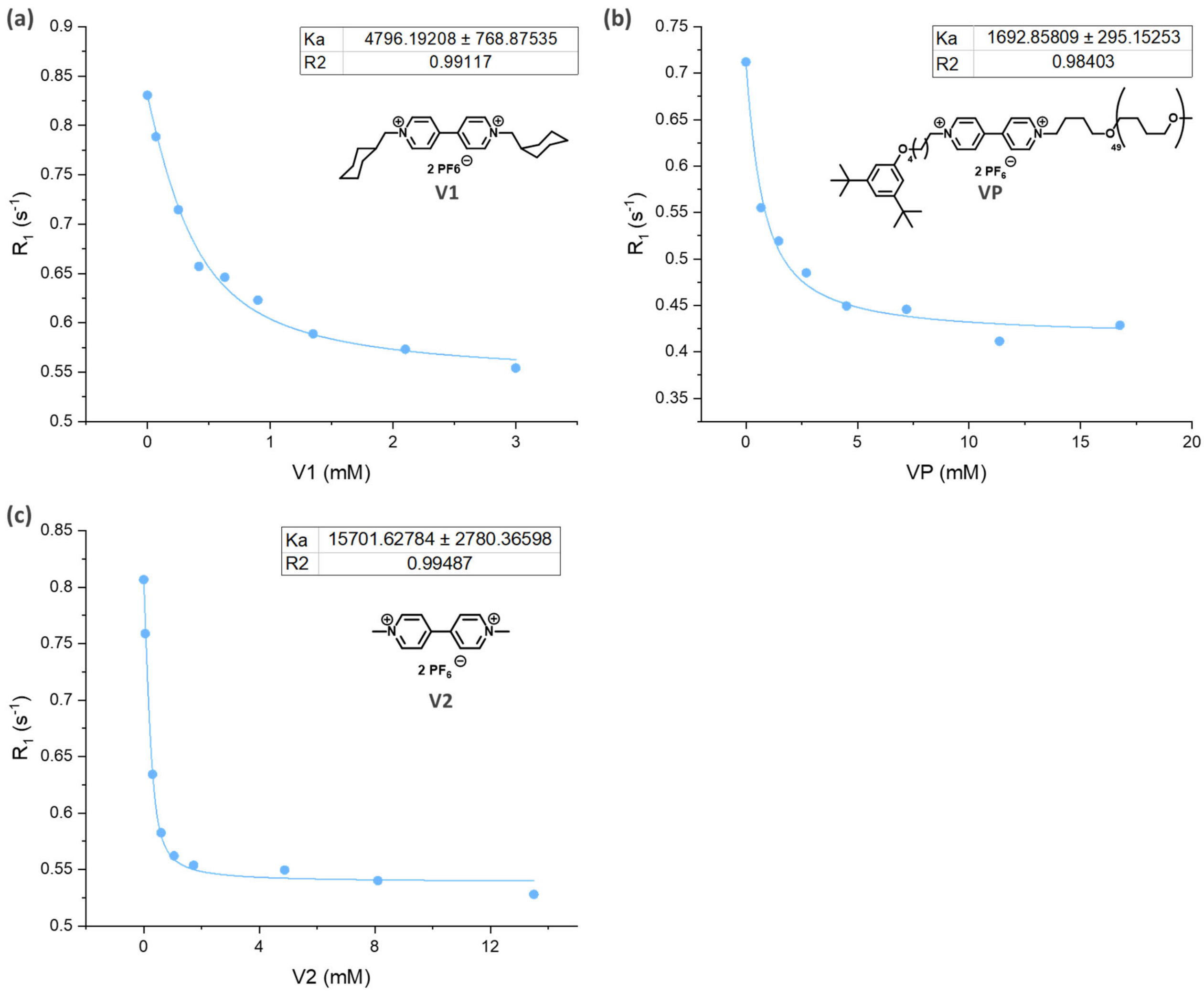

Supplementary Figure 21. Titration curves. a, Titration curve in which R_1 (s^{-1}) is plotted against guest concentration (mM) for the **Mn1**/**V1** complex giving a K_a of 4796.19 ± 768.88 M^{-1}. **b**, Similar titration curve for the **Mn1**/**VP** complex giving a K_a of 1692.86 ± 295.15 M^{-1}. **c**, Similar titration curve for the **Mn1**/**V2** complex giving a K_a of 15701.63 ± 2780.37 M^{-1}.

2.2.2. Temperature dependence

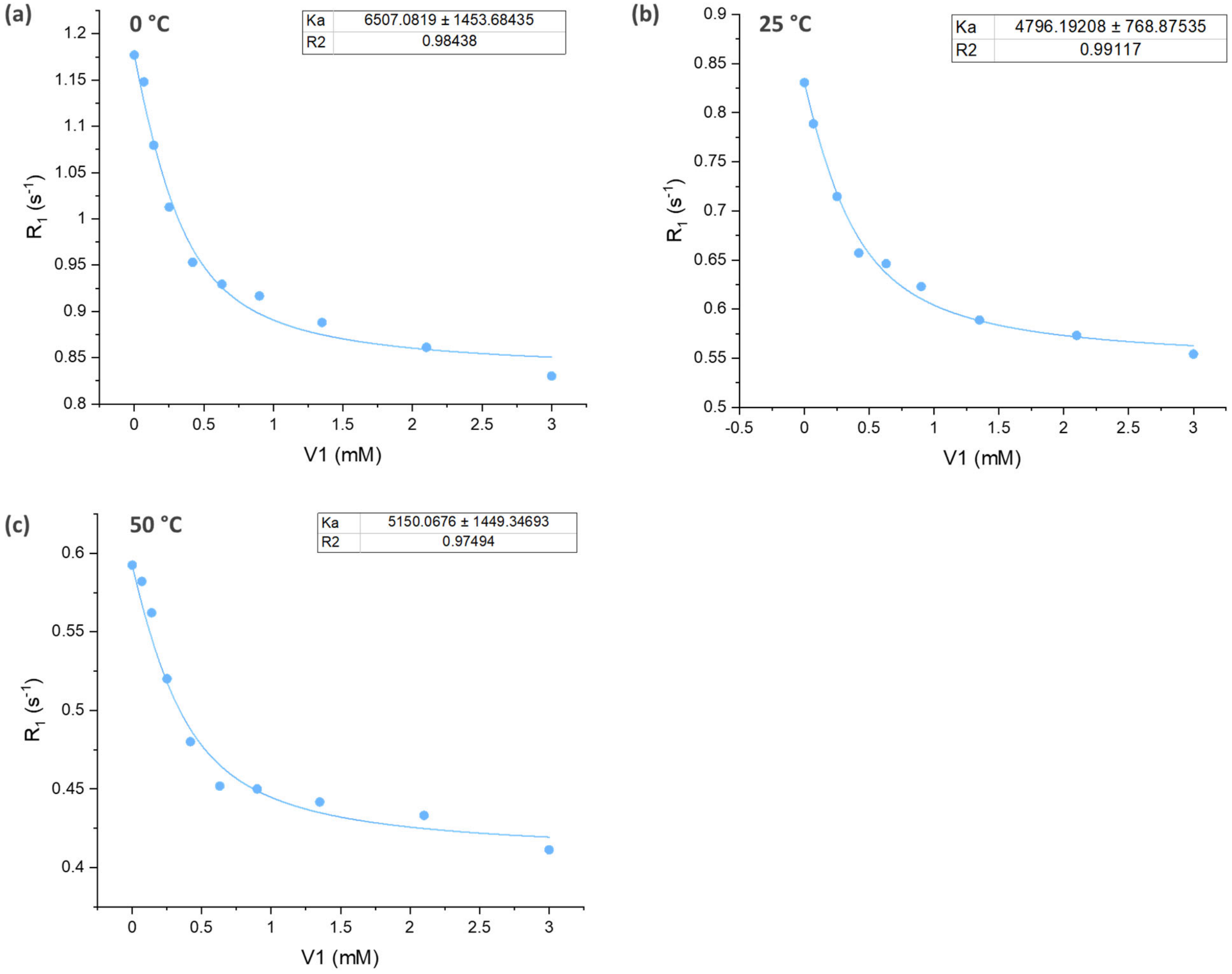

Supplementary Figure 22. Titration curves of Mn1/V1 at different temperatures. R_1 (s^{-1}) is plotted against guest concentration (mM). **a**, 0 °C, K_a = 6507.08 ± 1453.68 M^{-1}. **b**, 25 °C, K_a = 4796.19 ± 768.87 M^{-1}. **c**, 50 °C, 5150.07 ± 1449.35 M^{-1}. From these determined K_a values, it can be concluded there is no temperature dependence on the binding constant and therefore the binding constant of each measured system at room temperature can be used to calculate the concentration of bound host which is needed for the determination of dissociation rate constants in the PRE exchange studies.

2.3. UV-Vis binding titrations (H_21/V1)

Supplementary Table 14. Fitted K_{v1} values, error in the fit of the K_{v1} values and the link to the fits

Measurement	K_{v1} [M^{-1}]	K_{v1} error [%]	Link to the fits
1	16405174	23.7	*http://app.supramolecular.org/bindfit/view/9e28f33d-ad17-4546-8e44-f7b82567e07c*
2	24938513	31.2	*http://app.supramolecular.org/bindfit/view/8b9872ce-742f-44b8-9187-8419bb372b88*

3	17680722	23.9	*http://app.supramolecular.org/bindfit/view/6503c7c2-31c0-4c23-9ae2-504ad5526321*

Supplementary Table 15. measured data for the UV-Vis titration measurement 1 of $\mathbf{H_2 1}$ with **V1**

[H] [M]	[G] [M]	[G]/[H]	429 nm	428 nm	427 nm	426 nm	425 nm	424 nm	423 nm	422 nm	421 nm
1.99E-06	0.00E+00	0.00	0.1513	0.2046	0.2770	0.3363	0.4190	0.4862	0.5733	0.6547	0.7065
1.99E-06	1.99E-07	0.10	0.1565	0.2125	0.2847	0.3543	0.4288	0.4918	0.5853	0.6584	0.7093
1.99E-06	3.97E-07	0.20	0.1638	0.2225	0.2981	0.3581	0.4415	0.5170	0.5895	0.6627	0.7079
1.99E-06	5.95E-07	0.30	0.1691	0.2284	0.3001	0.3756	0.4521	0.5239	0.5981	0.6664	0.7054
1.99E-06	7.92E-07	0.40	0.1787	0.2407	0.3134	0.3861	0.4649	0.5267	0.6029	0.6697	0.7002
1.99E-06	9.89E-07	0.50	0.1842	0.2473	0.3230	0.3943	0.4686	0.5423	0.6153	0.6704	0.6978
1.99E-06	1.19E-06	0.59	0.1961	0.2541	0.3315	0.4027	0.4808	0.5399	0.6162	0.6774	0.6989
1.99E-06	1.38E-06	0.69	0.1920	0.2579	0.3342	0.4075	0.4830	0.5461	0.6187	0.6728	0.6961
1.99E-06	1.58E-06	0.79	0.1964	0.2616	0.3411	0.4170	0.4992	0.5527	0.6255	0.6751	0.6936
1.99E-06	1.77E-06	0.89	0.2025	0.2679	0.3442	0.4214	0.4980	0.5563	0.6258	0.6743	0.6943
1.99E-06	1.97E-06	0.99	0.2001	0.2661	0.3471	0.4169	0.4954	0.5549	0.6308	0.6739	0.6918
1.99E-06	2.35E-06	1.18	0.2012	0.2675	0.3475	0.4214	0.4990	0.5577	0.6356	0.6734	0.6901
1.99E-06	2.74E-06	1.37	0.2056	0.2714	0.3520	0.4324	0.5062	0.5613	0.6303	0.6764	0.6895
1.99E-06	3.12E-06	1.57	0.2097	0.2762	0.3571	0.4321	0.5085	0.5669	0.6325	0.6784	0.6906
1.99E-06	3.50E-06	1.76	0.2068	0.2741	0.3500	0.4327	0.5046	0.5624	0.6322	0.6795	0.6898
1.99E-06	3.88E-06	1.95	0.2135	0.2786	0.3594	0.4315	0.5096	0.5682	0.6352	0.6787	0.6909
1.99E-06	4.26E-06	2.14	0.2108	0.2803	0.3599	0.4301	0.5076	0.5697	0.6363	0.6789	0.6920
1.99E-06	6.15E-06	3.09	0.2097	0.2803	0.3543	0.4296	0.5086	0.5652	0.6382	0.6770	0.6899
1.99E-06	8.03E-06	4.03	0.2137	0.2836	0.3586	0.4376	0.5053	0.5699	0.6328	0.6786	0.6894
1.99E-06	9.92E-06	4.97	0.2143	0.2877	0.3612	0.4384	0.5142	0.5724	0.6387	0.6800	0.6904
1.99E-06	1.37E-05	6.85	0.2152	0.2841	0.3632	0.4358	0.5087	0.5724	0.6379	0.6783	0.6902
1.99E-06	1.74E-05	8.72	0.2135	0.2909	0.3686	0.4354	0.5245	0.5710	0.6345	0.6781	0.6883
1.99E-06	2.11E-05	10.58	0.2222	0.2846	0.3640	0.4406	0.5150	0.5769	0.6383	0.6795	0.6905
1.99E-06	3.03E-05	15.18	0.2157	0.2855	0.3685	0.4431	0.5187	0.5731	0.6397	0.6802	0.6912
1.99E-06	3.93E-05	19.73	0.2149	0.2830	0.3642	0.4449	0.5178	0.5761	0.6388	0.6811	0.6896

Supplementary Table 16. measured data for the UV-Vis titration measurement 2 of $\mathbf{H_2 1}$ with **V1**

[H] [M]	[G] [M]	[G]/[H]	429 nm	428 nm	427 nm	426 nm	425 nm	424 nm	423 nm	422 nm	421 nm
1.99E-06	0.00E+00	0.00	0.1502	0.2088	0.2782	0.3468	0.4218	0.4915	0.5834	0.6616	0.7142
1.99E-06	1.99E-07	0.10	0.1592	0.2188	0.2882	0.3577	0.4352	0.5041	0.5929	0.6696	0.7148
1.99E-06	3.97E-07	0.20	0.1677	0.2324	0.2998	0.3792	0.4514	0.5168	0.5975	0.6709	0.7135
1.99E-06	5.95E-07	0.30	0.1737	0.2342	0.3062	0.3731	0.4579	0.5163	0.6020	0.6659	0.7090
1.99E-06	7.92E-07	0.40	0.1833	0.2475	0.3233	0.3971	0.4699	0.5393	0.6140	0.6734	0.7060
1.99E-06	9.89E-07	0.50	0.1873	0.2479	0.3247	0.3968	0.4752	0.5408	0.6148	0.6746	0.7058
1.99E-06	1.19E-06	0.59	0.1930	0.2587	0.3359	0.4041	0.4836	0.5484	0.6252	0.6737	0.7022
1.99E-06	1.38E-06	0.69	0.1998	0.2649	0.3393	0.4175	0.4921	0.5534	0.6217	0.6808	0.7014
1.99E-06	1.58E-06	0.79	0.2038	0.2719	0.3464	0.4220	0.4989	0.5593	0.6294	0.6825	0.7006
1.99E-06	1.77E-06	0.89	0.2039	0.2723	0.3548	0.4347	0.5039	0.5651	0.6336	0.6829	0.6997
1.99E-06	1.97E-06	0.99	0.2036	0.2740	0.3527	0.4247	0.5006	0.5673	0.6358	0.6820	0.6979
1.99E-06	2.35E-06	1.18	0.2031	0.2728	0.3536	0.4257	0.5046	0.5643	0.6368	0.6830	0.6980
1.99E-06	2.74E-06	1.37	0.2099	0.2770	0.3679	0.4298	0.5129	0.5700	0.6430	0.6827	0.6976
1.99E-06	3.12E-06	1.57	0.2107	0.2730	0.3600	0.4325	0.5069	0.5705	0.6401	0.6847	0.6983
1.99E-06	3.50E-06	1.76	0.2094	0.2762	0.3628	0.4328	0.5136	0.5725	0.6406	0.6857	0.7013
1.99E-06	3.88E-06	1.95	0.2133	0.2825	0.3616	0.4362	0.5138	0.5734	0.6462	0.6843	0.6973
1.99E-06	4.26E-06	2.14	0.2150	0.2832	0.3699	0.4393	0.5184	0.5780	0.6422	0.6850	0.6982
1.99E-06	6.15E-06	3.09	0.2132	0.2824	0.3628	0.4336	0.5165	0.5754	0.6390	0.6841	0.6984
1.99E-06	8.03E-06	4.03	0.2156	0.2813	0.3663	0.4354	0.5129	0.5836	0.6435	0.6849	0.6976
1.99E-06	9.92E-06	4.97	0.2168	0.2849	0.3701	0.4406	0.5186	0.5786	0.6422	0.6845	0.6944
1.99E-06	1.37E-05	6.85	0.2237	0.2918	0.3665	0.4426	0.5256	0.5791	0.6456	0.6863	0.6986
1.99E-06	1.74E-05	8.72	0.2183	0.2851	0.3653	0.4365	0.5189	0.5829	0.6485	0.6896	0.6984
1.99E-06	2.11E-05	10.58	0.2183	0.2974	0.3665	0.4408	0.5166	0.5798	0.6455	0.6911	0.6984
1.99E-06	3.03E-05	15.18	0.2169	0.2825	0.3717	0.4397	0.5168	0.5786	0.6457	0.6892	0.6968
1.99E-06	3.93E-05	19.73	0.2252	0.2824	0.3691	0.4356	0.5215	0.5844	0.6473	0.6912	0.6992

Supplementary Table 17. measured data for the UV-Vis titration measurement 3 of $\mathbf{H_2 1}$ with **V1**

[H] [M]	[G] [M]	[G]/[H]	429 nm	428 nm	427 nm	426 nm	425 nm	424 nm	423 nm	422 nm	421 nm
1.99E-06	0.00E+00	0.00	0.1517	0.2076	0.2734	0.3406	0.4278	0.4816	0.5766	0.6546	0.7119
1.99E-06	1.99E-07	0.10	0.1623	0.2177	0.2894	0.3582	0.4377	0.5034	0.5908	0.6644	0.7081
1.99E-06	3.97E-07	0.20	0.1675	0.2325	0.3022	0.3717	0.4439	0.5111	0.5944	0.6644	0.7088
1.99E-06	5.95E-07	0.30	0.1719	0.2334	0.3038	0.3765	0.4540	0.5151	0.6006	0.6663	0.7075
1.99E-06	7.92E-07	0.40	0.1766	0.2373	0.3154	0.3822	0.4656	0.5255	0.6046	0.6705	0.7053
1.99E-06	9.89E-07	0.50	0.1884	0.2489	0.3239	0.3991	0.4773	0.5380	0.6146	0.6736	0.7007
1.99E-06	1.19E-06	0.59	0.1926	0.2576	0.3410	0.4105	0.4785	0.5438	0.6210	0.6720	0.7003
1.99E-06	1.38E-06	0.69	0.1948	0.2586	0.3336	0.4083	0.4858	0.5466	0.6230	0.6748	0.6969
1.99E-06	1.58E-06	0.79	0.1955	0.2617	0.3396	0.4137	0.4907	0.5509	0.6213	0.6764	0.6966
1.99E-06	1.77E-06	0.89	0.1975	0.2647	0.3497	0.4243	0.4938	0.5560	0.6272	0.6766	0.6946
1.99E-06	1.97E-06	0.99	0.2075	0.2707	0.3508	0.4230	0.4978	0.5621	0.6301	0.6766	0.6915
1.99E-06	2.35E-06	1.18	0.2114	0.2715	0.3519	0.4253	0.5037	0.5672	0.6303	0.6780	0.6932
1.99E-06	2.74E-06	1.37	0.2144	0.2772	0.3581	0.4332	0.5061	0.5617	0.6365	0.6802	0.6912
1.99E-06	3.12E-06	1.57	0.2061	0.2740	0.3562	0.4276	0.4997	0.5654	0.6317	0.6791	0.6904
1.99E-06	3.50E-06	1.76	0.2103	0.2796	0.3593	0.4310	0.5097	0.5660	0.6346	0.6786	0.6910
1.99E-06	3.88E-06	1.95	0.2123	0.2818	0.3631	0.4353	0.5103	0.5677	0.6372	0.6797	0.6916
1.99E-06	4.26E-06	2.14	0.2120	0.2807	0.3651	0.4367	0.5120	0.5709	0.6352	0.6795	0.6915
1.99E-06	6.15E-06	3.09	0.2149	0.2855	0.3632	0.4335	0.5145	0.5714	0.6371	0.6803	0.6905
1.99E-06	8.03E-06	4.03	0.2127	0.2801	0.3627	0.4365	0.5182	0.5703	0.6382	0.6815	0.6916
1.99E-06	9.92E-06	4.97	0.2164	0.2839	0.3645	0.4391	0.5095	0.5750	0.6347	0.6784	0.6898
1.99E-06	1.37E-05	6.85	0.2158	0.2844	0.3639	0.4355	0.5161	0.5738	0.6382	0.6803	0.6908
1.99E-06	1.74E-05	8.72	0.2164	0.2837	0.3617	0.4396	0.5244	0.5797	0.6374	0.6803	0.6908
1.99E-06	2.11E-05	10.58	0.2171	0.2847	0.3690	0.4414	0.5224	0.5750	0.6404	0.6812	0.6910
1.99E-06	3.03E-05	15.18	0.2174	0.2854	0.3697	0.4428	0.5156	0.5752	0.6404	0.6802	0.6909
1.99E-06	3.93E-05	19.73	0.2157	0.2870	0.3673	0.4427	0.5183	0.5816	0.6404	0.6802	0.6910

Supplementary Table 18. fitted data for the UV-Vis titration measurement 1 of $\mathbf{H_2 1}$ with **V1**

[H] [M]	[G] [M]	[G]/[H]	429 nm	428 nm	427 nm	426 nm	425 nm	424 nm	423 nm	422 nm	421 nm
1.99E-06	0.00E+00	0.00	0.1513	0.2046	0.2770	0.3363	0.4190	0.4862	0.5733	0.6547	0.7065
1.99E-06	1.99E-07	0.10	0.1574	0.2122	0.2852	0.3462	0.4283	0.4946	0.5797	0.6571	0.7049

1.99E-06	3.97E-07	0.20	0.1634	0.2197	0.2934	0.3560	0.4374	0.5029	0.5859	0.6595	0.7033
1.99E-06	5.95E-07	0.30	0.1693	0.2271	0.3014	0.3657	0.4464	0.5110	0.5921	0.6619	0.7018
1.99E-06	7.92E-07	0.40	0.1751	0.2344	0.3093	0.3753	0.4553	0.5190	0.5982	0.6643	0.7002
1.99E-06	9.89E-07	0.50	0.1808	0.2415	0.3170	0.3846	0.4640	0.5269	0.6042	0.6665	0.6987
1.99E-06	1.19E-06	0.59	0.1863	0.2484	0.3245	0.3935	0.4723	0.5344	0.6099	0.6687	0.6973
1.99E-06	1.38E-06	0.69	0.1915	0.2549	0.3315	0.4020	0.4802	0.5416	0.6153	0.6708	0.6959
1.99E-06	1.58E-06	0.79	0.1962	0.2609	0.3379	0.4098	0.4874	0.5481	0.6203	0.6727	0.6947
1.99E-06	1.77E-06	0.89	0.2003	0.2660	0.3436	0.4166	0.4937	0.5538	0.6246	0.6744	0.6936
1.99E-06	1.97E-06	0.99	0.2037	0.2702	0.3481	0.4221	0.4988	0.5584	0.6281	0.6757	0.6927
1.99E-06	2.35E-06	1.18	0.2079	0.2756	0.3539	0.4291	0.5053	0.5643	0.6326	0.6775	0.6916
1.99E-06	2.74E-06	1.37	0.2100	0.2782	0.3567	0.4325	0.5086	0.5672	0.6348	0.6783	0.6910
1.99E-06	3.12E-06	1.57	0.2111	0.2796	0.3583	0.4343	0.5102	0.5687	0.6360	0.6788	0.6907
1.99E-06	3.50E-06	1.76	0.2118	0.2804	0.3591	0.4354	0.5112	0.5696	0.6367	0.6790	0.6905
1.99E-06	3.88E-06	1.95	0.2122	0.2810	0.3597	0.4361	0.5119	0.5702	0.6371	0.6792	0.6904
1.99E-06	4.26E-06	2.14	0.2125	0.2813	0.3601	0.4366	0.5123	0.5706	0.6374	0.6793	0.6903
1.99E-06	6.15E-06	3.09	0.2132	0.2822	0.3611	0.4378	0.5134	0.5716	0.6381	0.6796	0.6902
1.99E-06	8.03E-06	4.03	0.2135	0.2826	0.3615	0.4382	0.5138	0.5720	0.6384	0.6797	0.6901
1.99E-06	9.92E-06	4.97	0.2136	0.2828	0.3617	0.4384	0.5141	0.5722	0.6386	0.6798	0.6900
1.99E-06	1.37E-05	6.85	0.2138	0.2829	0.3619	0.4387	0.5143	0.5724	0.6387	0.6798	0.6900
1.99E-06	1.74E-05	8.72	0.2139	0.2830	0.3620	0.4388	0.5144	0.5725	0.6388	0.6799	0.6900
1.99E-06	2.11E-05	10.58	0.2139	0.2831	0.3620	0.4389	0.5145	0.5726	0.6389	0.6799	0.6900
1.99E-06	3.03E-05	15.18	0.2140	0.2832	0.3621	0.4390	0.5146	0.5726	0.6389	0.6799	0.6900
1.99E-06	3.93E-05	19.73	0.2140	0.2832	0.3622	0.4391	0.5146	0.5727	0.6390	0.6799	0.6899

Supplementary Table 19. fitted data for the UV-Vis titration measurement 2 of **$H_2$1** with **V1**

[H] [M]	[G] [M]	[G]/[H]	429 nm	428 nm	427 nm	426 nm	425 nm	424 nm	423 nm	422 nm	421 nm
1.99E-06	0.00E+00	0.00	0.1502	0.2088	0.2782	0.3468	0.4218	0.4915	0.5834	0.6616	0.7142
1.99E-06	1.99E-07	0.10	0.1567	0.2162	0.2869	0.3559	0.4313	0.5001	0.5894	0.6641	0.7125
1.99E-06	3.97E-07	0.20	0.1632	0.2236	0.2956	0.3649	0.4407	0.5086	0.5954	0.6665	0.7109
1.99E-06	5.95E-07	0.30	0.1696	0.2310	0.3042	0.3738	0.4500	0.5171	0.6013	0.6689	0.7093
1.99E-06	7.92E-07	0.40	0.1760	0.2382	0.3127	0.3826	0.4592	0.5254	0.6071	0.6713	0.7077
1.99E-06	9.89E-07	0.50	0.1822	0.2453	0.3211	0.3913	0.4682	0.5336	0.6128	0.6737	0.7061
1.99E-06	1.19E-06	0.59	0.1882	0.2522	0.3292	0.3997	0.4770	0.5416	0.6184	0.6760	0.7046
1.99E-06	1.38E-06	0.69	0.1940	0.2589	0.3370	0.4078	0.4854	0.5492	0.6237	0.6782	0.7031
1.99E-06	1.58E-06	0.79	0.1994	0.2650	0.3442	0.4152	0.4932	0.5563	0.6287	0.6802	0.7018
1.99E-06	1.77E-06	0.89	0.2041	0.2704	0.3505	0.4218	0.5001	0.5625	0.6330	0.6820	0.7006
1.99E-06	1.97E-06	0.99	0.2079	0.2747	0.3555	0.4270	0.5055	0.5674	0.6364	0.6834	0.6997
1.99E-06	2.35E-06	1.18	0.2123	0.2797	0.3614	0.4331	0.5118	0.5732	0.6404	0.6850	0.6986
1.99E-06	2.74E-06	1.37	0.2141	0.2818	0.3639	0.4356	0.5145	0.5756	0.6421	0.6857	0.6981
1.99E-06	3.12E-06	1.57	0.2150	0.2828	0.3651	0.4369	0.5158	0.5768	0.6430	0.6861	0.6979
1.99E-06	3.50E-06	1.76	0.2155	0.2834	0.3657	0.4376	0.5165	0.5774	0.6434	0.6863	0.6978
1.99E-06	3.88E-06	1.95	0.2158	0.2837	0.3662	0.4380	0.5170	0.5779	0.6437	0.6864	0.6977
1.99E-06	4.26E-06	2.14	0.2160	0.2840	0.3665	0.4383	0.5173	0.5781	0.6439	0.6865	0.6976
1.99E-06	6.15E-06	3.09	0.2165	0.2846	0.3671	0.4390	0.5181	0.5788	0.6444	0.6867	0.6975
1.99E-06	8.03E-06	4.03	0.2167	0.2848	0.3674	0.4393	0.5183	0.5791	0.6445	0.6867	0.6974
1.99E-06	9.92E-06	4.97	0.2168	0.2849	0.3675	0.4394	0.5185	0.5792	0.6446	0.6868	0.6974
1.99E-06	1.37E-05	6.85	0.2169	0.2850	0.3677	0.4396	0.5186	0.5794	0.6447	0.6868	0.6974
1.99E-06	1.74E-05	8.72	0.2170	0.2851	0.3678	0.4397	0.5187	0.5794	0.6448	0.6868	0.6974
1.99E-06	2.11E-05	10.58	0.2170	0.2851	0.3678	0.4397	0.5188	0.5795	0.6448	0.6868	0.6974
1.99E-06	3.03E-05	15.18	0.2171	0.2852	0.3679	0.4398	0.5188	0.5795	0.6449	0.6869	0.6974
1.99E-06	3.93E-05	19.73	0.2171	0.2852	0.3679	0.4398	0.5189	0.5796	0.6449	0.6869	0.6973

Supplementary Table 20. fitted data for the UV-Vis titration measurement 3 of **$H_2$1** with **V1**

[H] [M]	[G] [M]	[G]/[H]	429 nm	428 nm	427 nm	426 nm	425 nm	424 nm	423 nm	422 nm	421 nm
1.99E-06	0.00E+00	0.00	0.1517	0.2076	0.2734	0.3406	0.4278	0.4816	0.5766	0.6546	0.7119
1.99E-06	1.99E-07	0.10	0.1579	0.2150	0.2823	0.3502	0.4362	0.4907	0.5827	0.6572	0.7098
1.99E-06	3.97E-07	0.20	0.1641	0.2223	0.2912	0.3598	0.4446	0.4997	0.5887	0.6597	0.7078
1.99E-06	5.95E-07	0.30	0.1701	0.2295	0.3000	0.3692	0.4529	0.5086	0.5947	0.6623	0.7057
1.99E-06	7.92E-07	0.40	0.1761	0.2366	0.3087	0.3784	0.4610	0.5173	0.6005	0.6648	0.7037
1.99E-06	9.89E-07	0.50	0.1819	0.2435	0.3171	0.3875	0.4690	0.5259	0.6062	0.6672	0.7018
1.99E-06	1.19E-06	0.59	0.1875	0.2502	0.3253	0.3962	0.4767	0.5341	0.6118	0.6696	0.6999
1.99E-06	1.38E-06	0.69	0.1928	0.2566	0.3330	0.4045	0.4840	0.5420	0.6170	0.6718	0.6981
1.99E-06	1.58E-06	0.79	0.1977	0.2624	0.3401	0.4121	0.4906	0.5492	0.6218	0.6739	0.6965
1.99E-06	1.77E-06	0.89	0.2020	0.2675	0.3463	0.4187	0.4965	0.5554	0.6260	0.6757	0.6950
1.99E-06	1.97E-06	0.99	0.2054	0.2716	0.3513	0.4241	0.5012	0.5605	0.6294	0.6771	0.6939
1.99E-06	2.35E-06	1.18	0.2097	0.2767	0.3576	0.4308	0.5071	0.5668	0.6336	0.6789	0.6924
1.99E-06	2.74E-06	1.37	0.2118	0.2792	0.3606	0.4340	0.5099	0.5699	0.6356	0.6798	0.6917
1.99E-06	3.12E-06	1.57	0.2129	0.2805	0.3622	0.4357	0.5114	0.5715	0.6367	0.6802	0.6914
1.99E-06	3.50E-06	1.76	0.2135	0.2812	0.3631	0.4367	0.5123	0.5724	0.6373	0.6805	0.6912
1.99E-06	3.88E-06	1.95	0.2139	0.2817	0.3637	0.4373	0.5128	0.5730	0.6377	0.6807	0.6910
1.99E-06	4.26E-06	2.14	0.2142	0.2820	0.3641	0.4377	0.5132	0.5734	0.6380	0.6808	0.6909
1.99E-06	6.15E-06	3.09	0.2149	0.2828	0.3651	0.4388	0.5141	0.5744	0.6387	0.6811	0.6907
1.99E-06	8.03E-06	4.03	0.2151	0.2831	0.3655	0.4392	0.5145	0.5748	0.6389	0.6812	0.6906
1.99E-06	9.92E-06	4.97	0.2153	0.2833	0.3657	0.4394	0.5147	0.5750	0.6391	0.6812	0.6906
1.99E-06	1.37E-05	6.85	0.2154	0.2835	0.3659	0.4396	0.5149	0.5752	0.6392	0.6813	0.6905
1.99E-06	1.74E-05	8.72	0.2155	0.2836	0.3660	0.4397	0.5150	0.5753	0.6393	0.6813	0.6905
1.99E-06	2.11E-05	10.58	0.2155	0.2836	0.3660	0.4398	0.5150	0.5754	0.6393	0.6813	0.6905
1.99E-06	3.03E-05	15.18	0.2156	0.2837	0.3661	0.4399	0.5151	0.5754	0.6394	0.6814	0.6905
1.99E-06	3.93E-05	19.73	0.2156	0.2837	0.3662	0.4400	0.5152	0.5755	0.6394	0.6814	0.6905

2.4. 1D EXSY studies (H_21/V1)

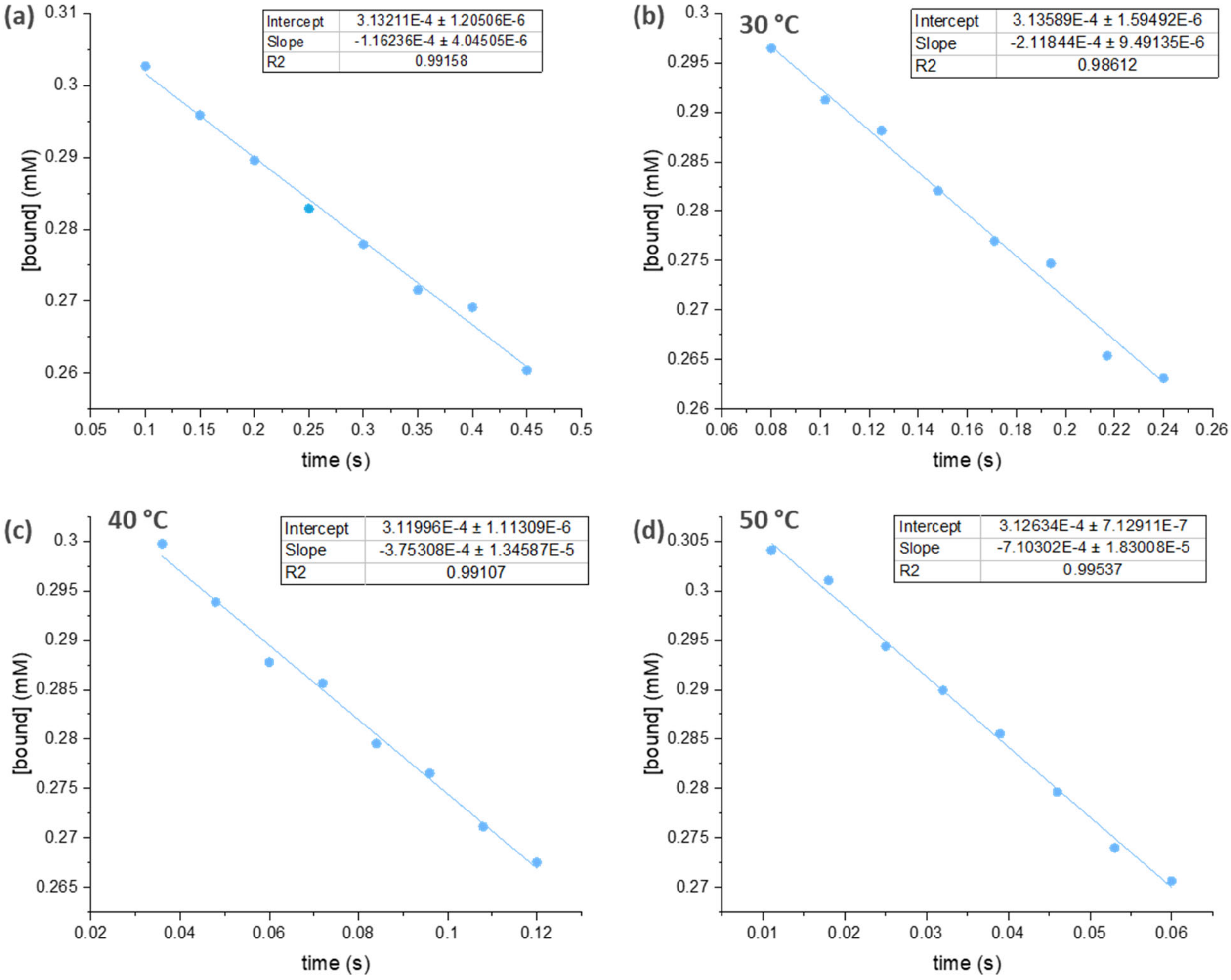

Supplementary Figure 23. 1D EXSY initial rates experiments for H_21/V1. The decay of [bound] guest is depicted over time at different temperatures: **a.** 20 °C; **b.** 30 °C; **c.** 40 °C; **d.** 50 °C. The data is fitted linearly.

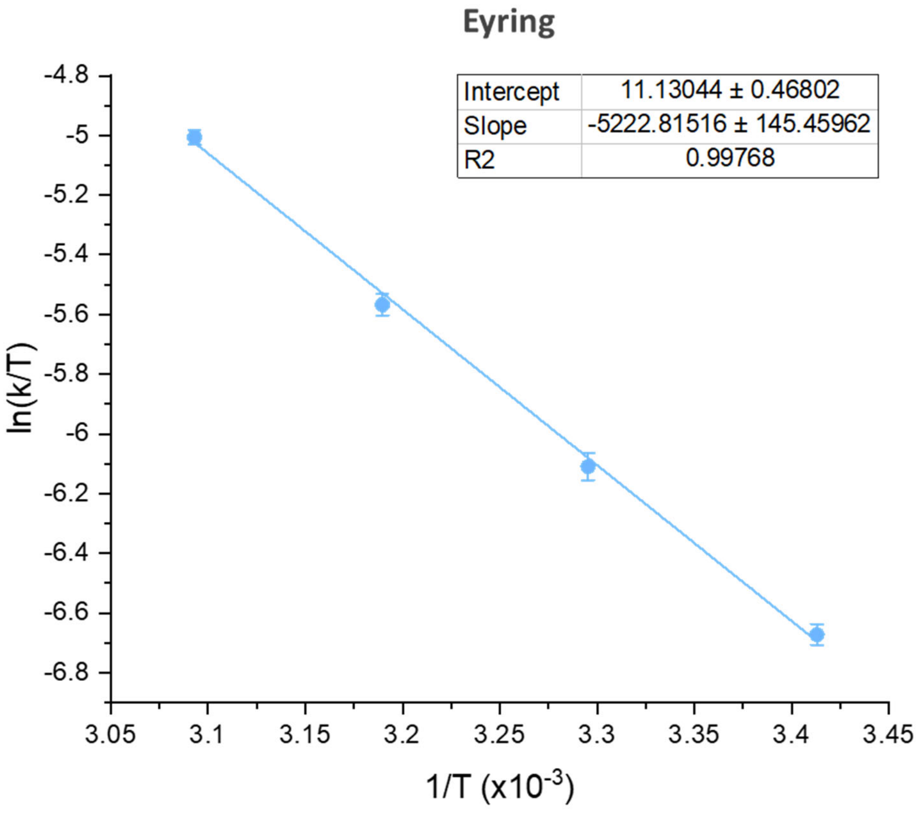

Supplementary Figure 24. Eyring plot for H_21/V1 in which the inverse temperature (1/T) is plotted against ln(k/T).

3. NMR spectra of compounds

3.1. V1

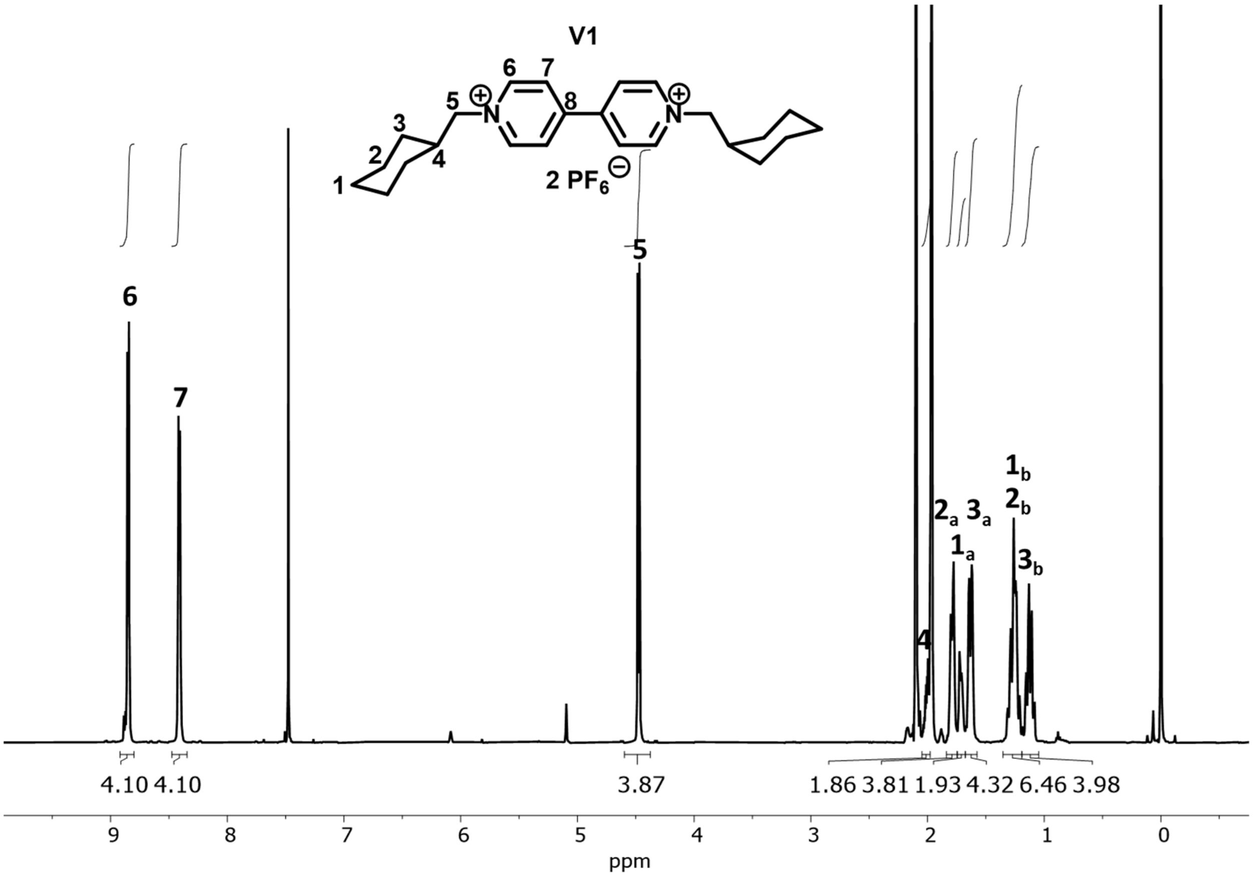

Supplementary Figure 25. ^{1}H **NMR spectrum (500 MHz) of V2 (acetonitrile-*d₃*:chloroform-*d*, 1:1, v/v, 298 K).**

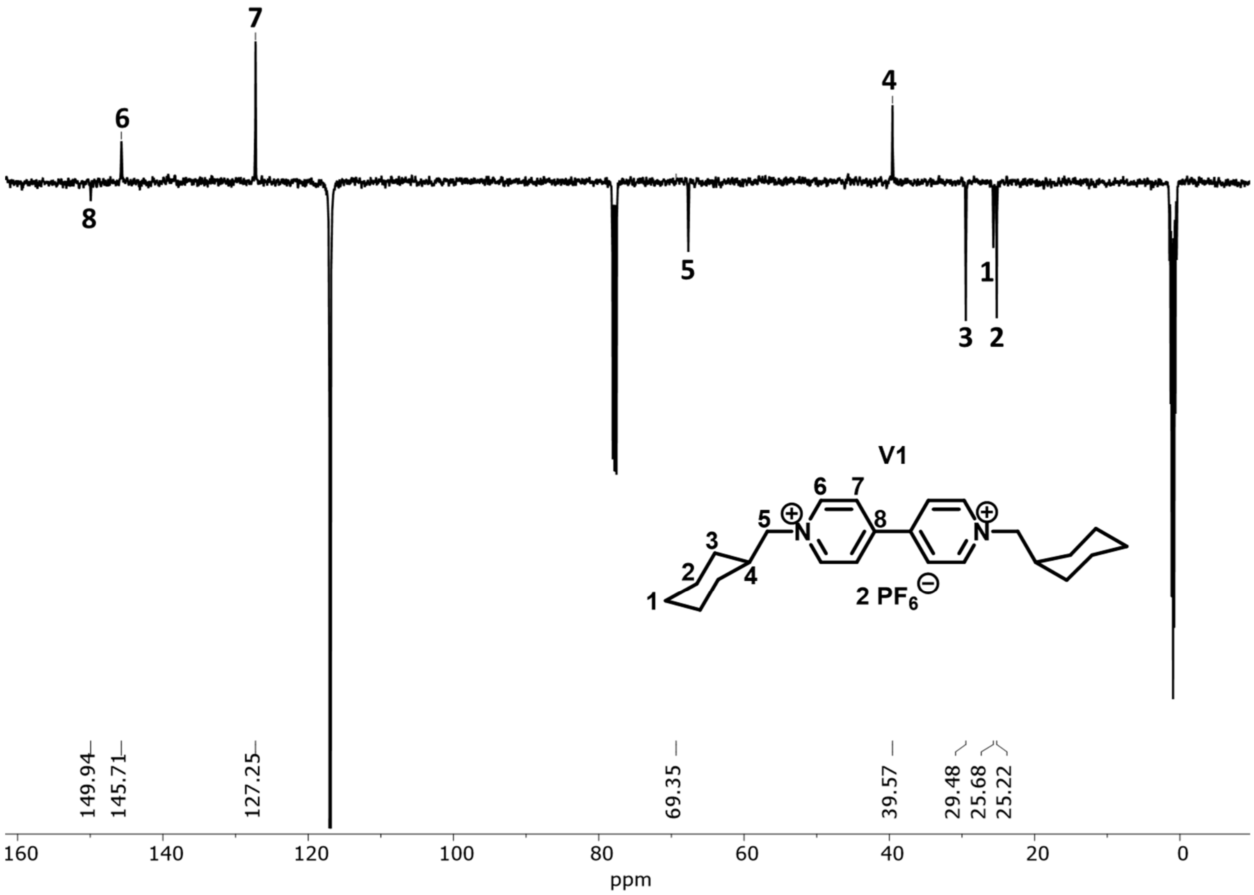

Supplementary Figure 26. ^{13}**C-APT NMR spectrum (125 MHz) of V2 (acetonitrile-*d*$_3$:chloroform-*d*, 1:1, v/v, 298 K).**

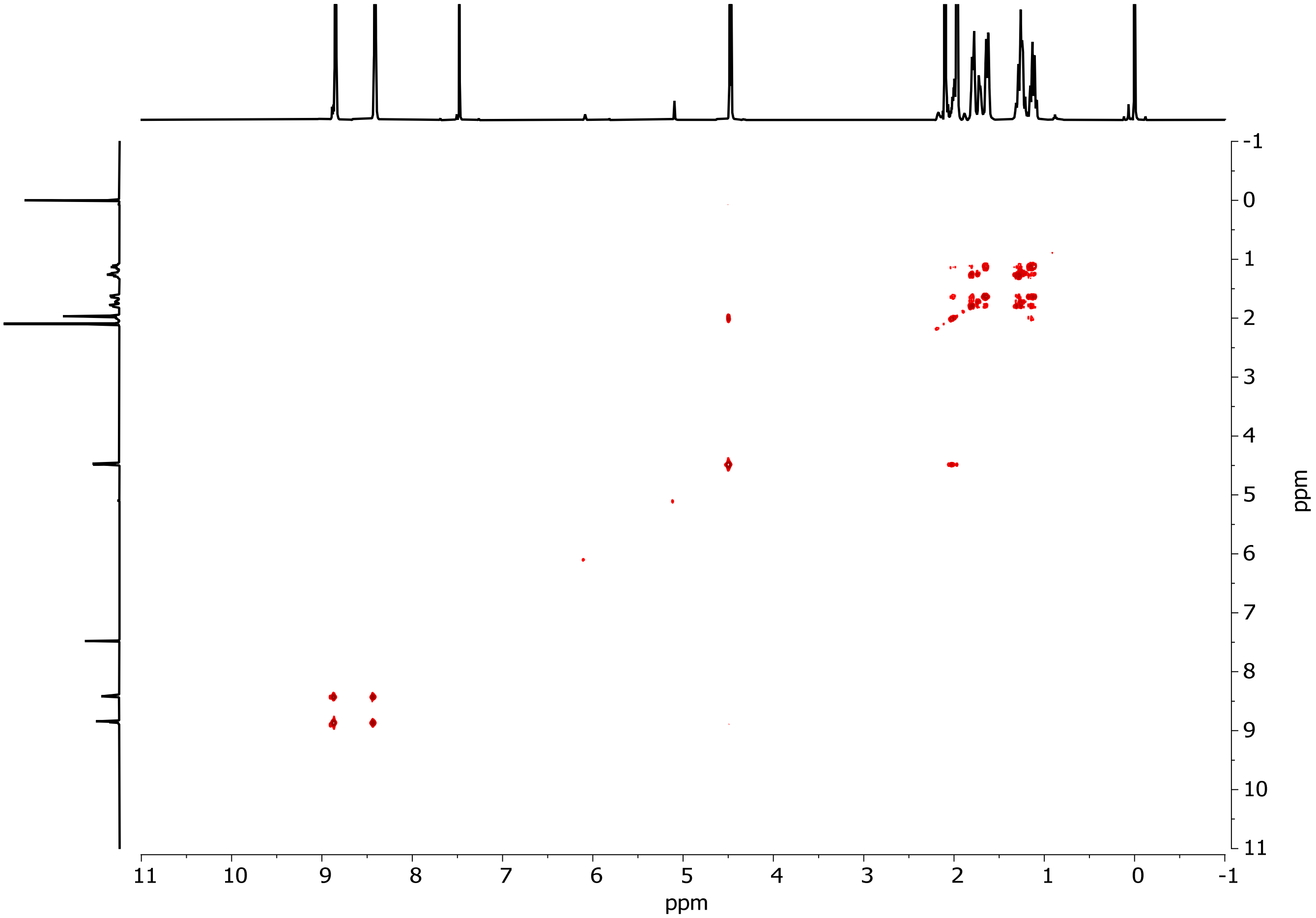

Supplementary Figure 27. ^{1}H-^{1}H COSY NMR spectrum (500 MHz) of V2 (acetonitrile-*d*$_3$:chloroform-*d*, 1:1, v/v, 298 K).

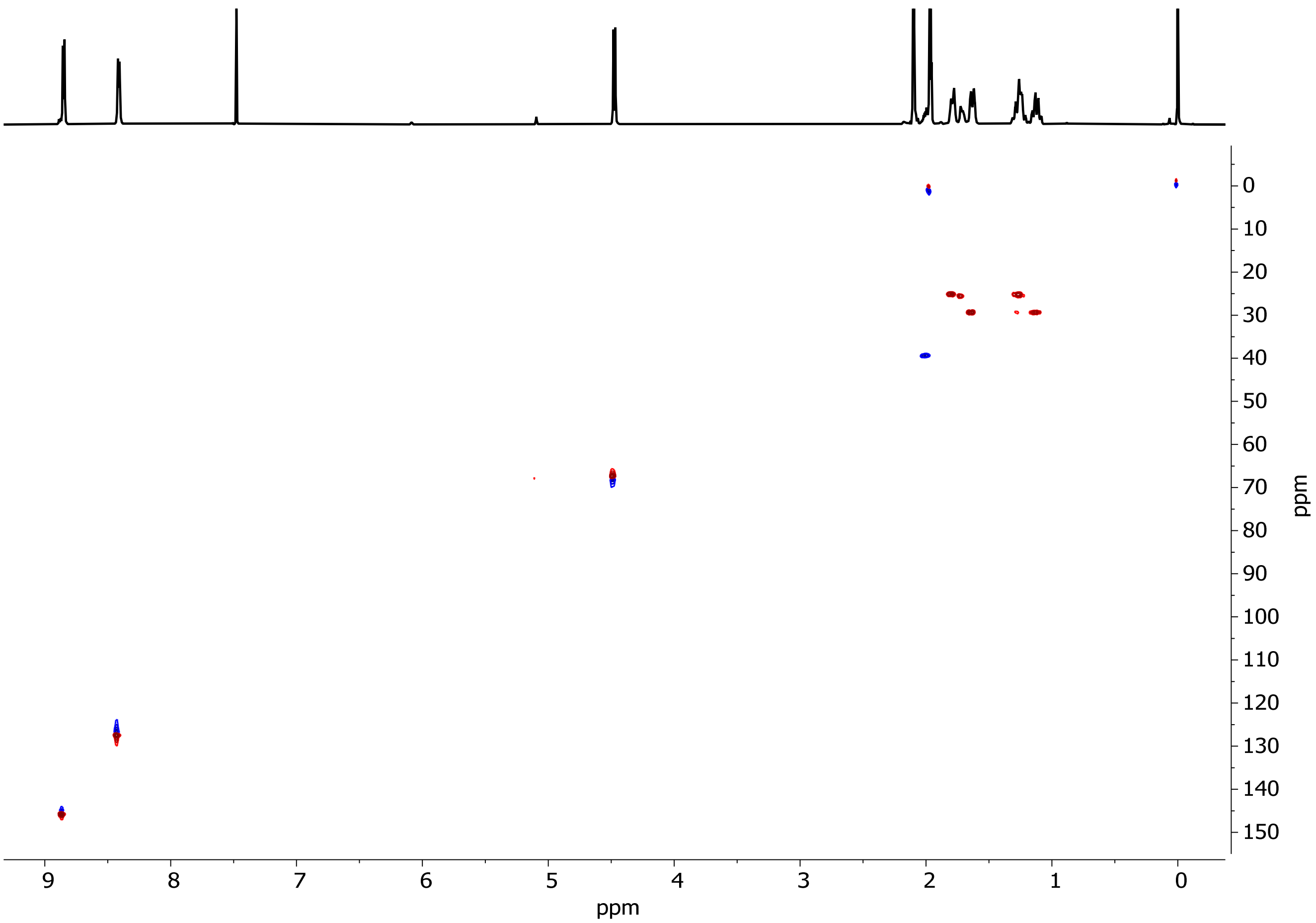

Supplementary Figure 28. ^{13}C-^{1}H **HSQC NMR spectrum (125 MHz) of V2 (acetonitrile-*d*$_3$:chloroform-*d*, 1:1, v/v, 298 K).**

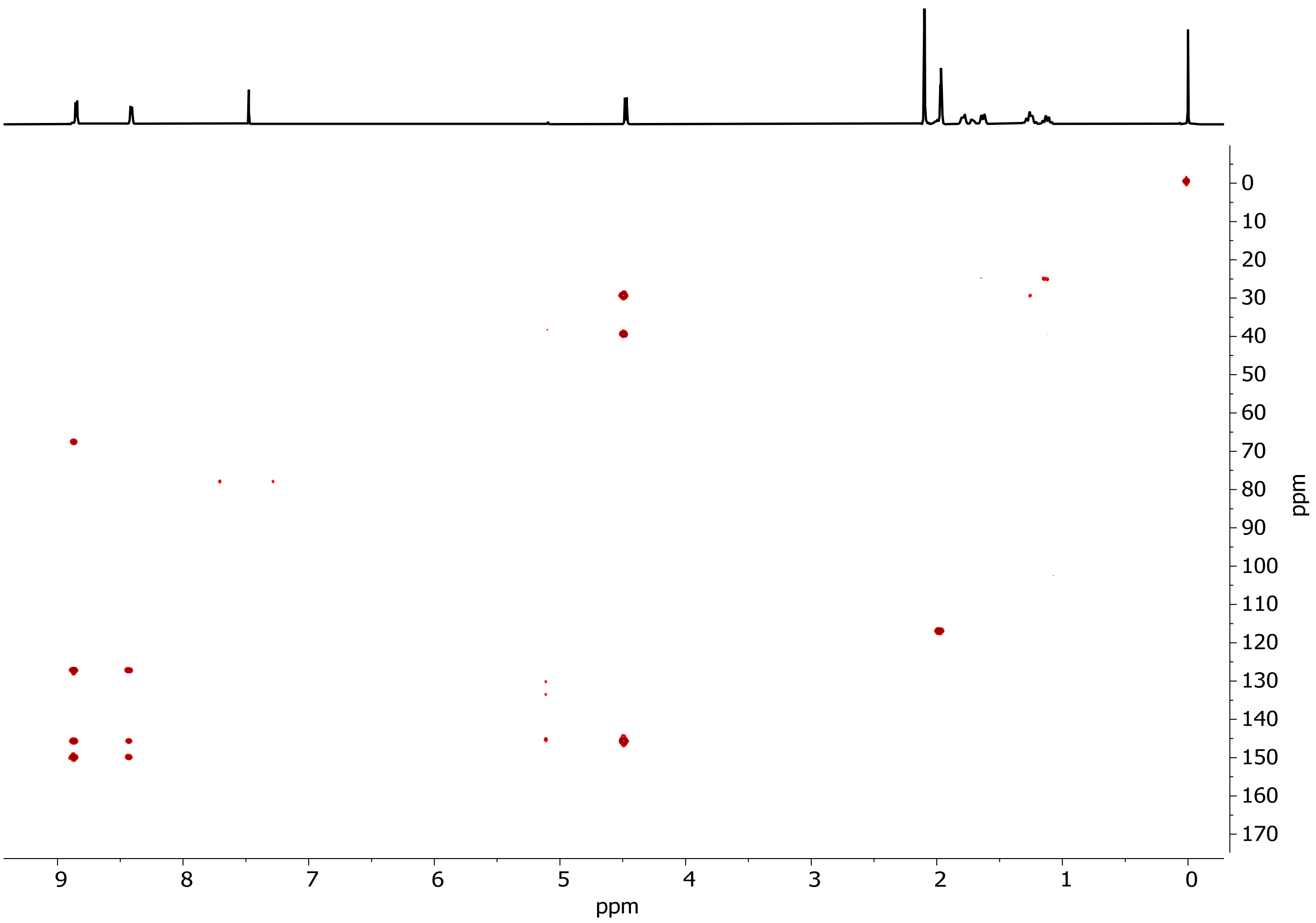

Supplementary Figure 29. ^{13}**C-**^{1}**H HMBC NMR spectrum (125 MHz) of V2 (acetonitrile-*d*$_3$:chloroform-*d*, 1:1, v/v, 298 K).**

3.2. Mn1

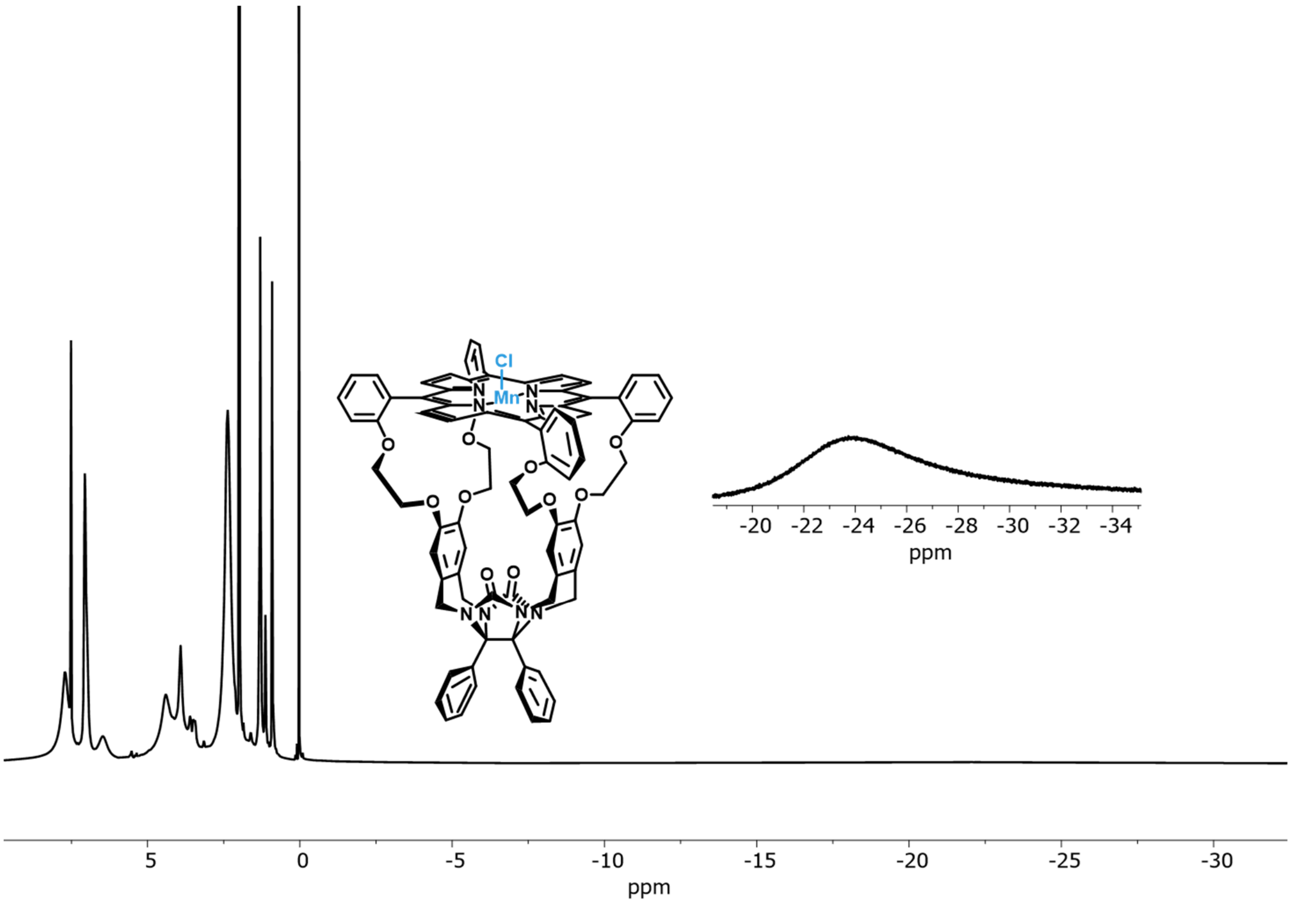

Supplementary Figure 30. ^{1}H NMR spectrum (500 MHz) of Mn1 (acetonitrile-*d$_3$*:chloroform-*d*, 1:1, v/v, 298 K).

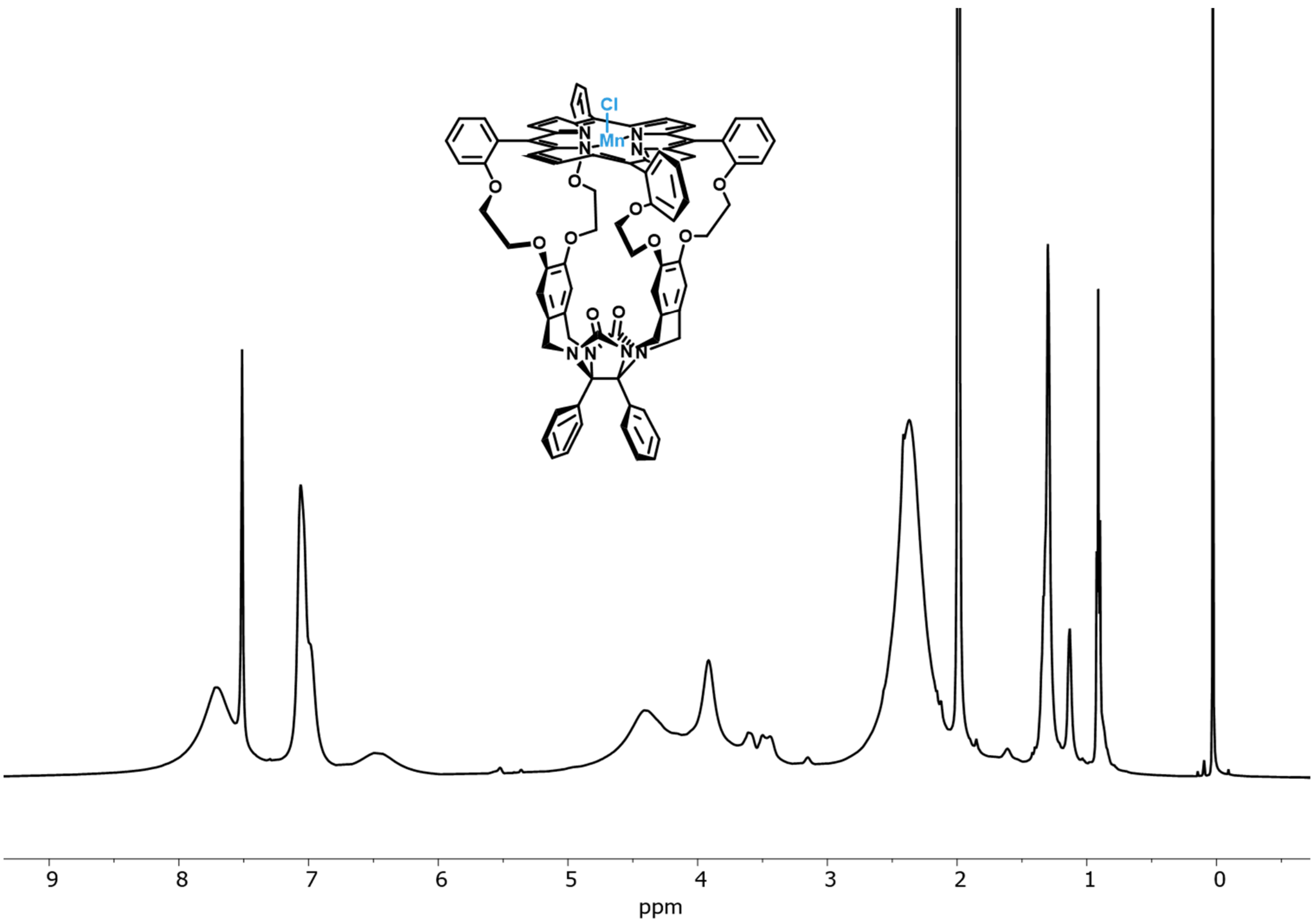

Supplementary Figure 31. ^{1}H NMR spectrum (500 MHz) of Mn1 (acetonitrile-*d_3*:chloroform-*d*, 1:1, v/v, 298 K) zoomed-in in the region 9.5 ppm to −0.7 ppm.

3.3. H_2Rot

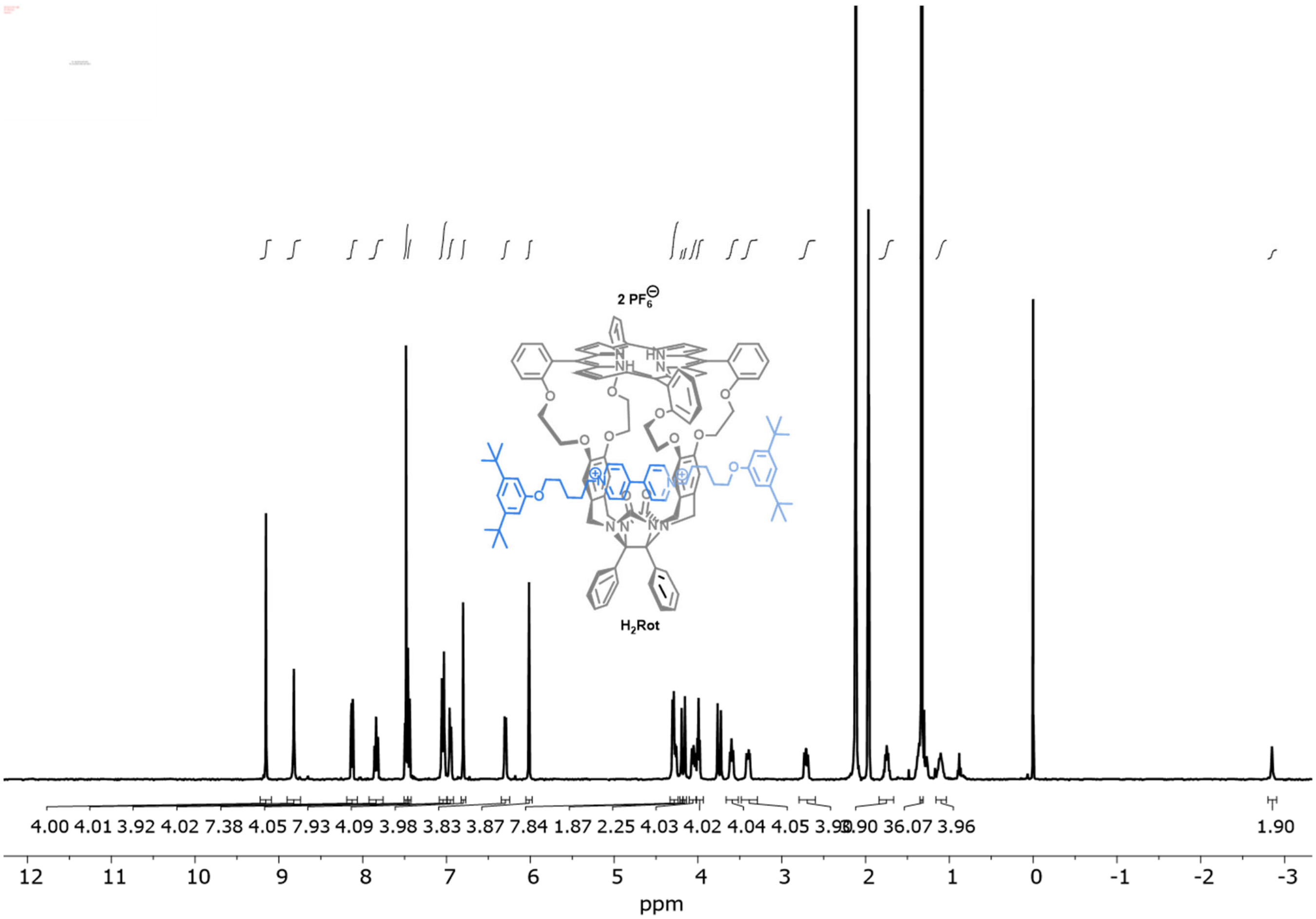

Supplementary Figure 32. ^{1}H NMR spectrum (400 MHz) of H_2Rot (acetonitrile-*d_3*:chloroform-*d*, 1:1, v/v, 298 K).

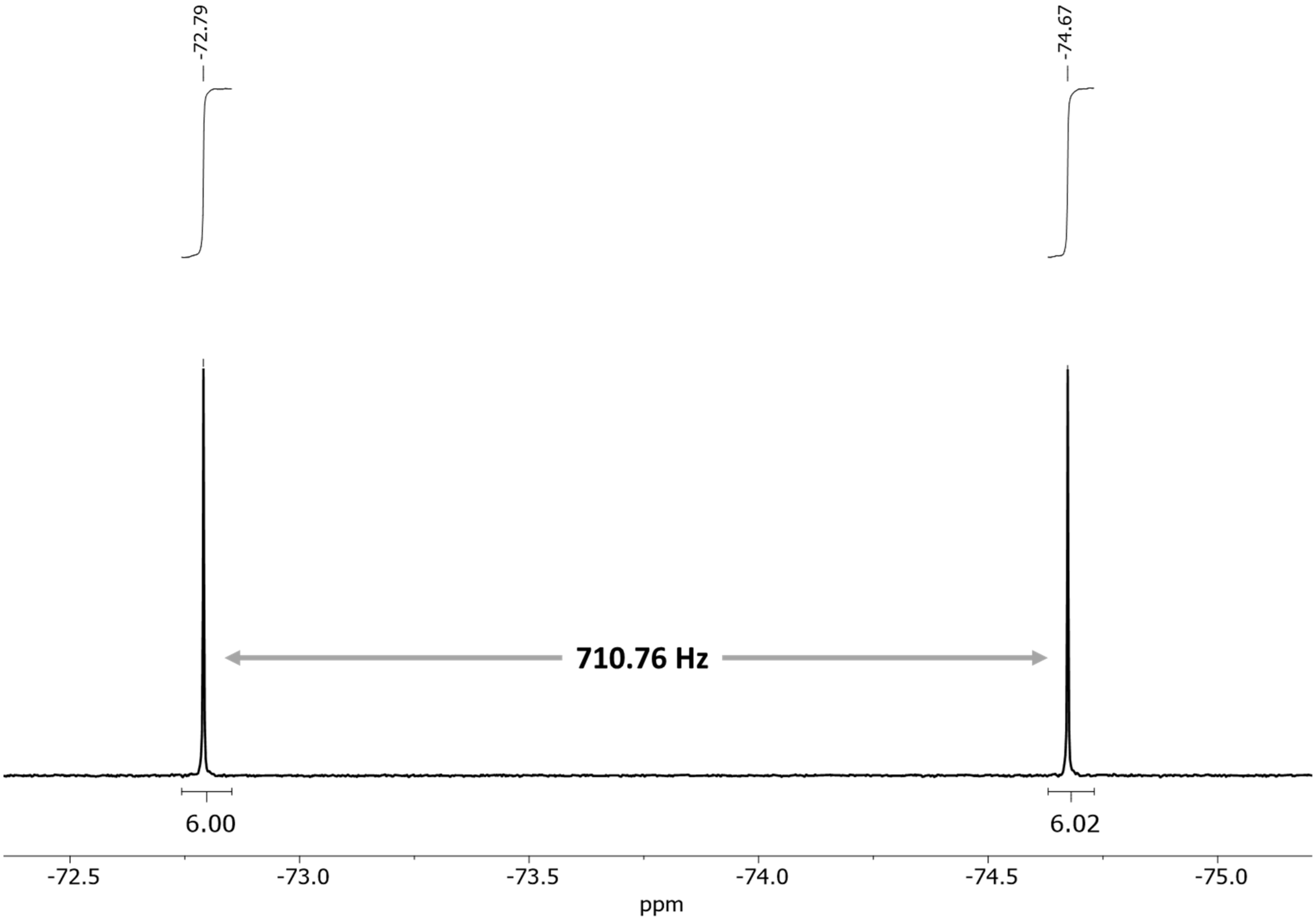

Supplementary Figure 33. ^{19}F NMR spectrum (376 MHz) of H_2Rot (acetonitrile-*d_3*:chloroform-*d*, 1:1, v/v, 298 K).

3.4. MnRot

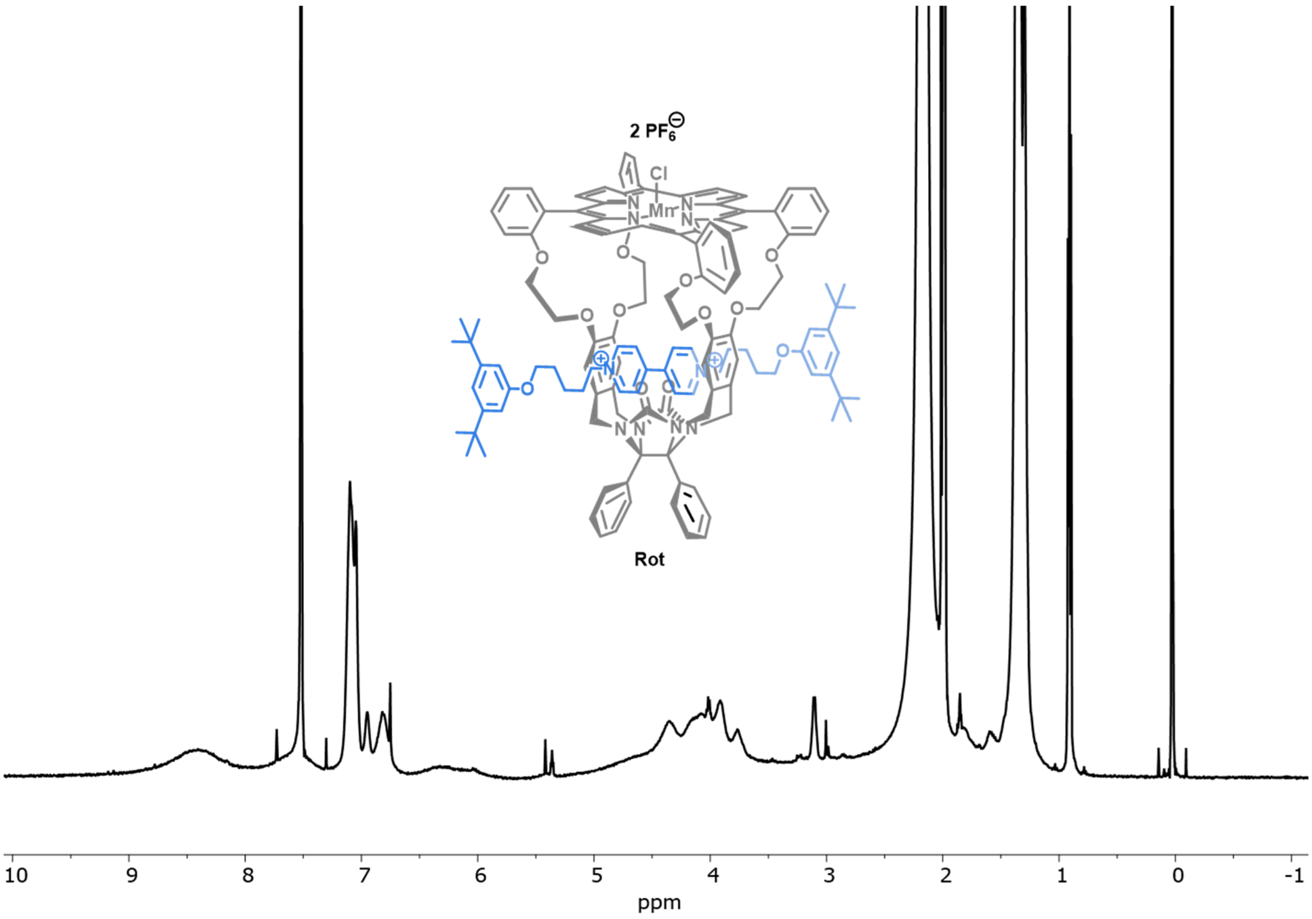

Supplementary Figure 34. **^{1}H NMR spectrum (500 MHz) of MnRot (acetonitrile-d_3:chloroform-d, 1:1, v/v, 298 K).**

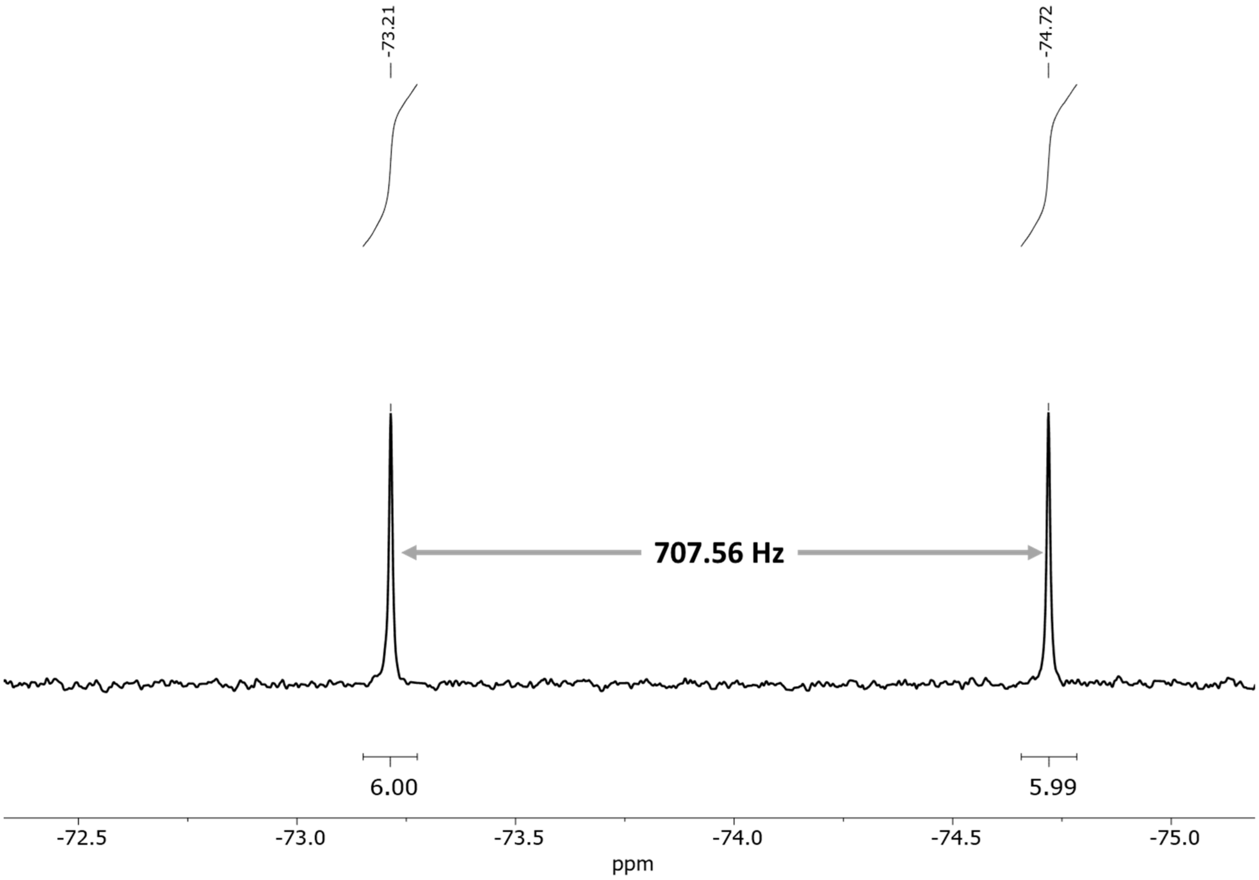

Supplementary Figure 35. ^{19}**F NMR spectrum (470 MHz) of MnRot (acetonitrile-*d*$_3$:chloroform-*d*, 1:1, v/v, 298 K).**

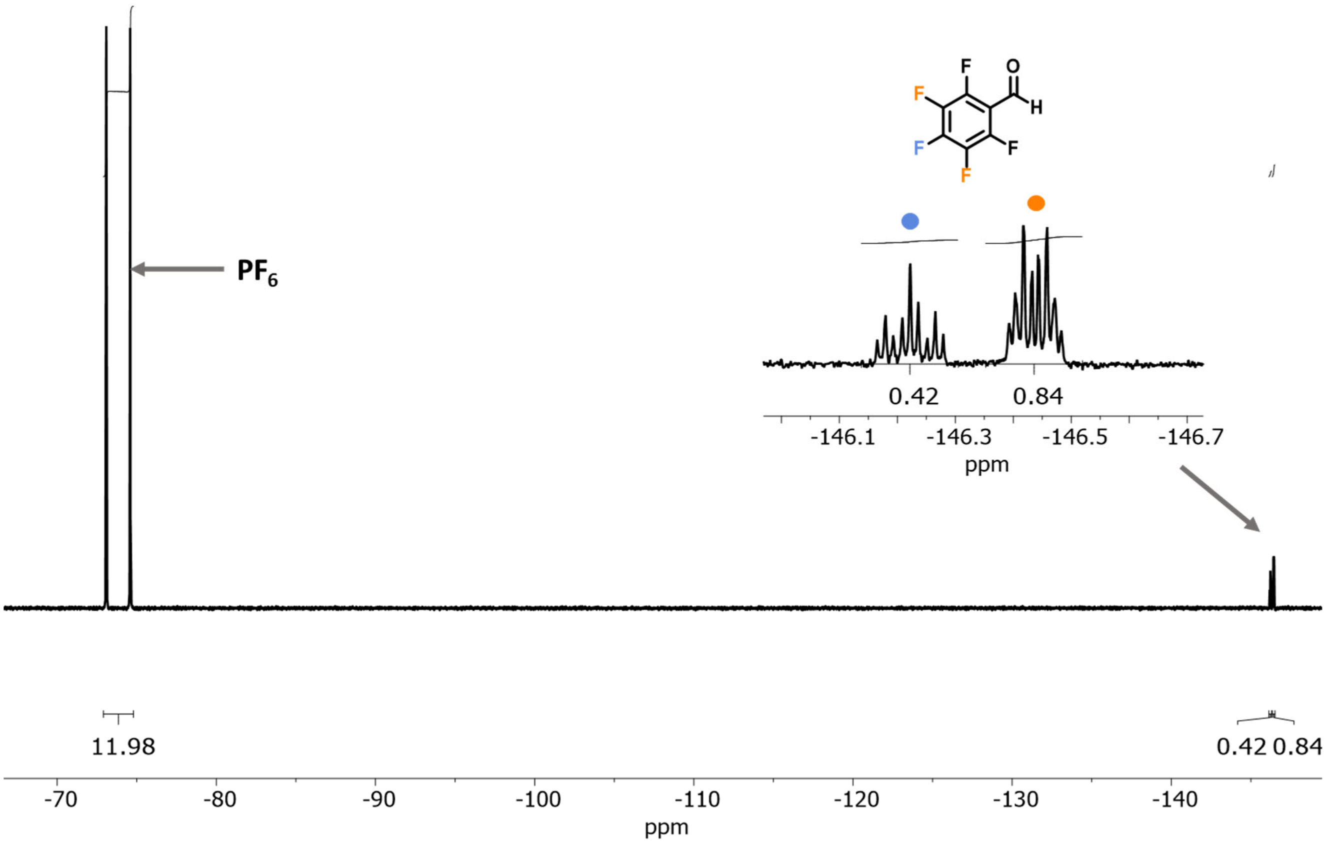

Supplementary Figure 36. ^{19}F NMR spectrum (470 MHz) of MnRot (1 mM) and internal standard: pentafluorobenzaldehyde (0.5 mM) (acetonitrile-*d$_3$*:chloroform-*d*, 1:1, v/v, 298 K). From this spectrum, it was determined that the expected concentration of PF_6 is present in the rotaxane, further confirming the presence of the viologen guest that is trapped inside the cavity of the host molecule.

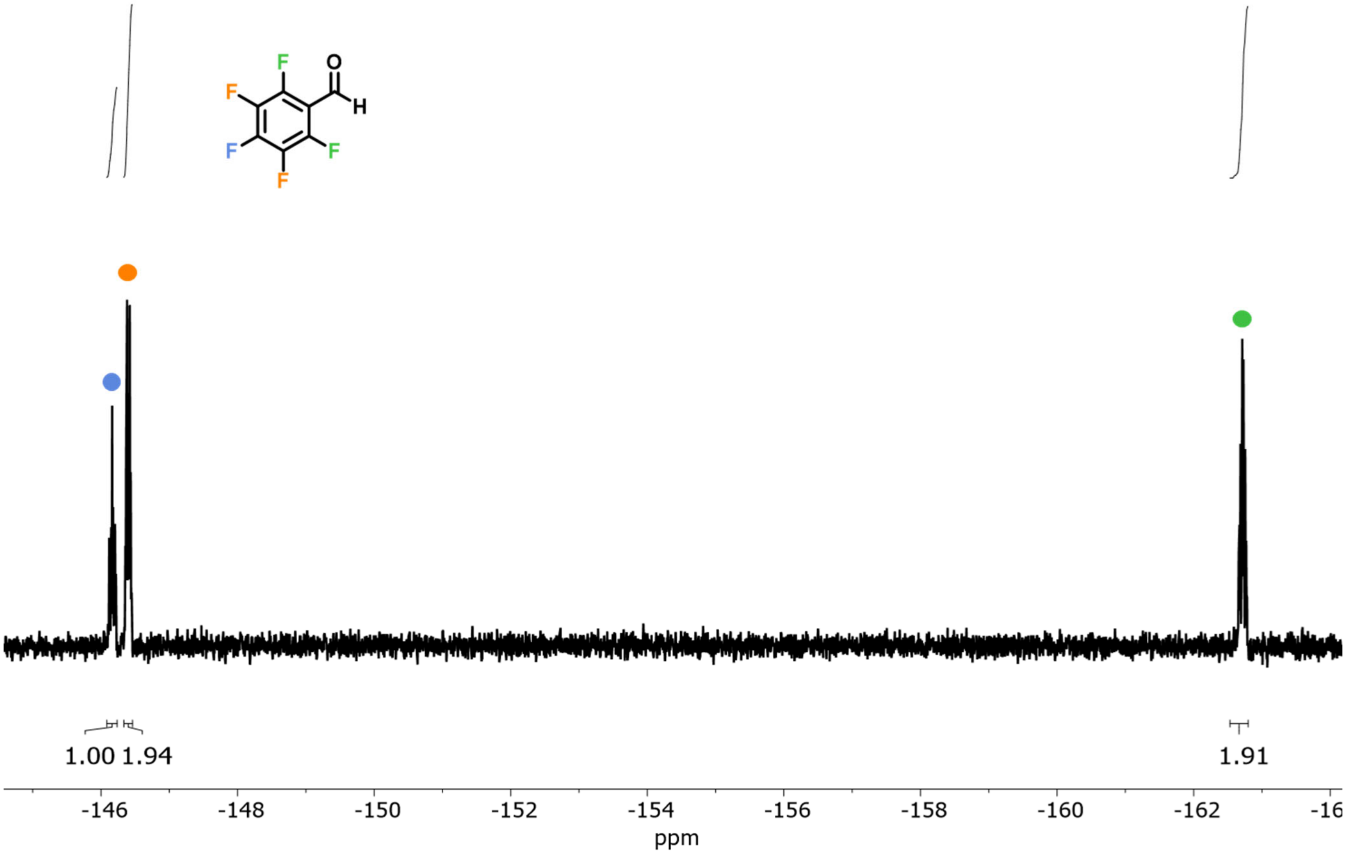

Supplementary Figure 37. **^{19}F NMR spectrum (470 MHz) of the internal standard pentafluorobenzaldehyde (acetonitrile-*d_3*:chloroform-*d*, 1:1, v/v, 298 K).**

3.5. Mn1/V1

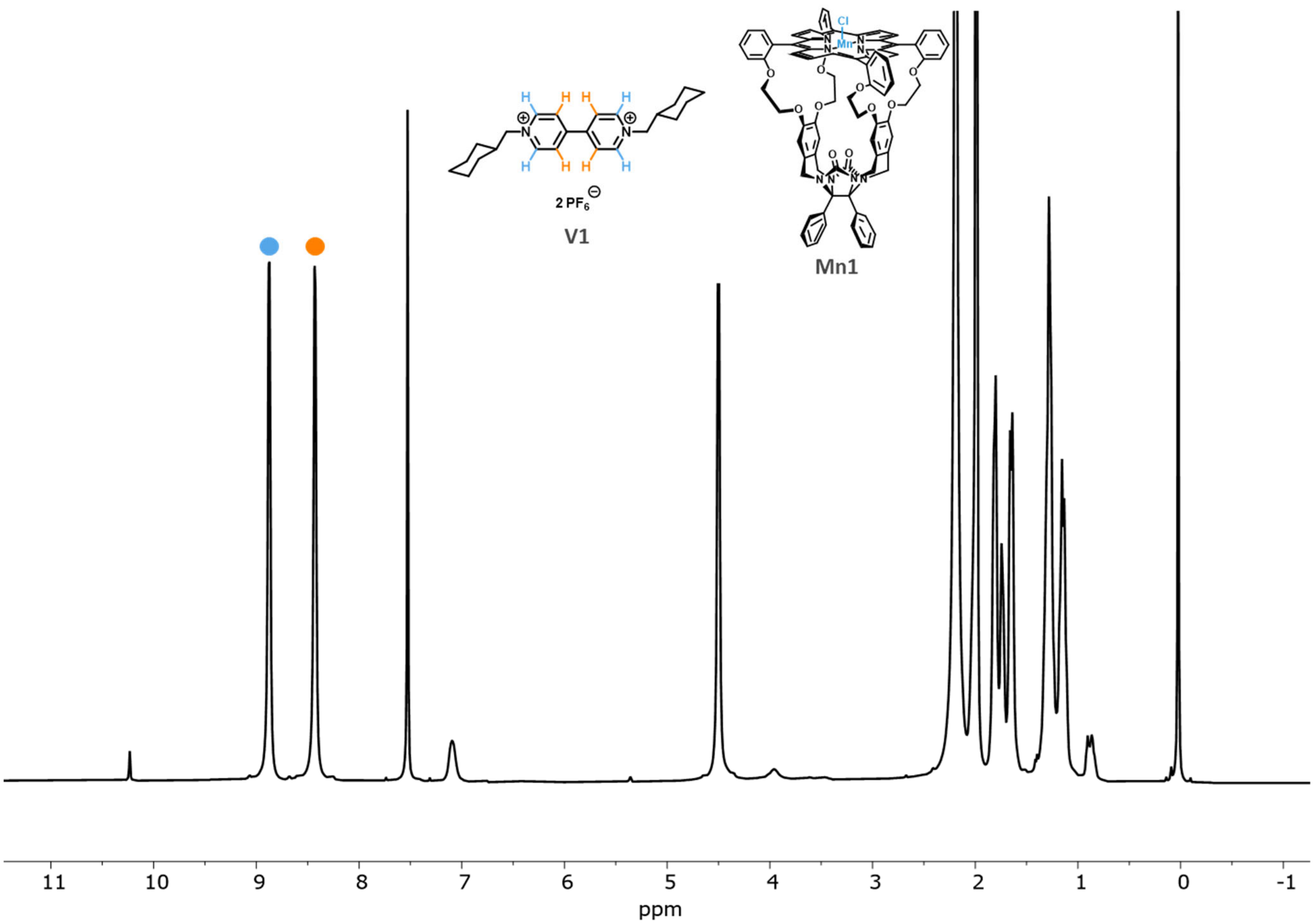

Supplementary Figure 38. ^{1}H NMR spectrum (500 MHz) of Mn1/V1 (0.5 mM/5 mM) (acetonitrile-*d*$_3$:chloroform-*d*, 1:1, v/v, 293 K). The signals of unbound **V1** that are selected for the T_1 and T_2 experiments are depicted in blue (ortho-protons) and orange (meta-protons).

3.6. Mn1/V2

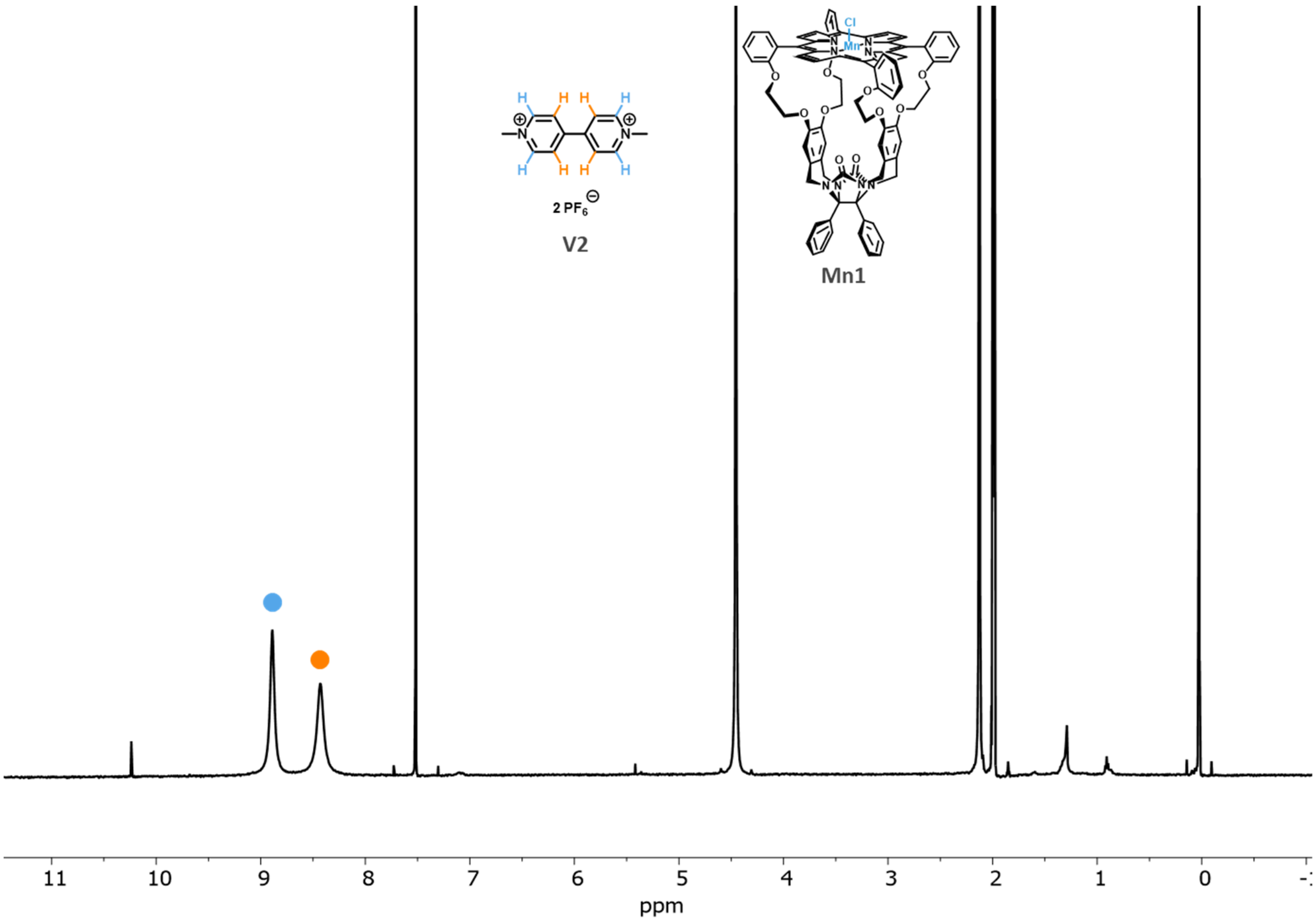

Supplementary Figure 39. ^{1}H NMR spectrum (500 MHz) of Mn1/V2 (0.5 mM/5 mM) (acetonitrile-d_3:chloroform-*d*, 1:1, v/v, 293 K). The signals of unbound **V2** that are selected for the T_1 and T_2 experiments are depicted in blue (ortho-protons) and orange (meta-protons).

3.7. Mn1/VP

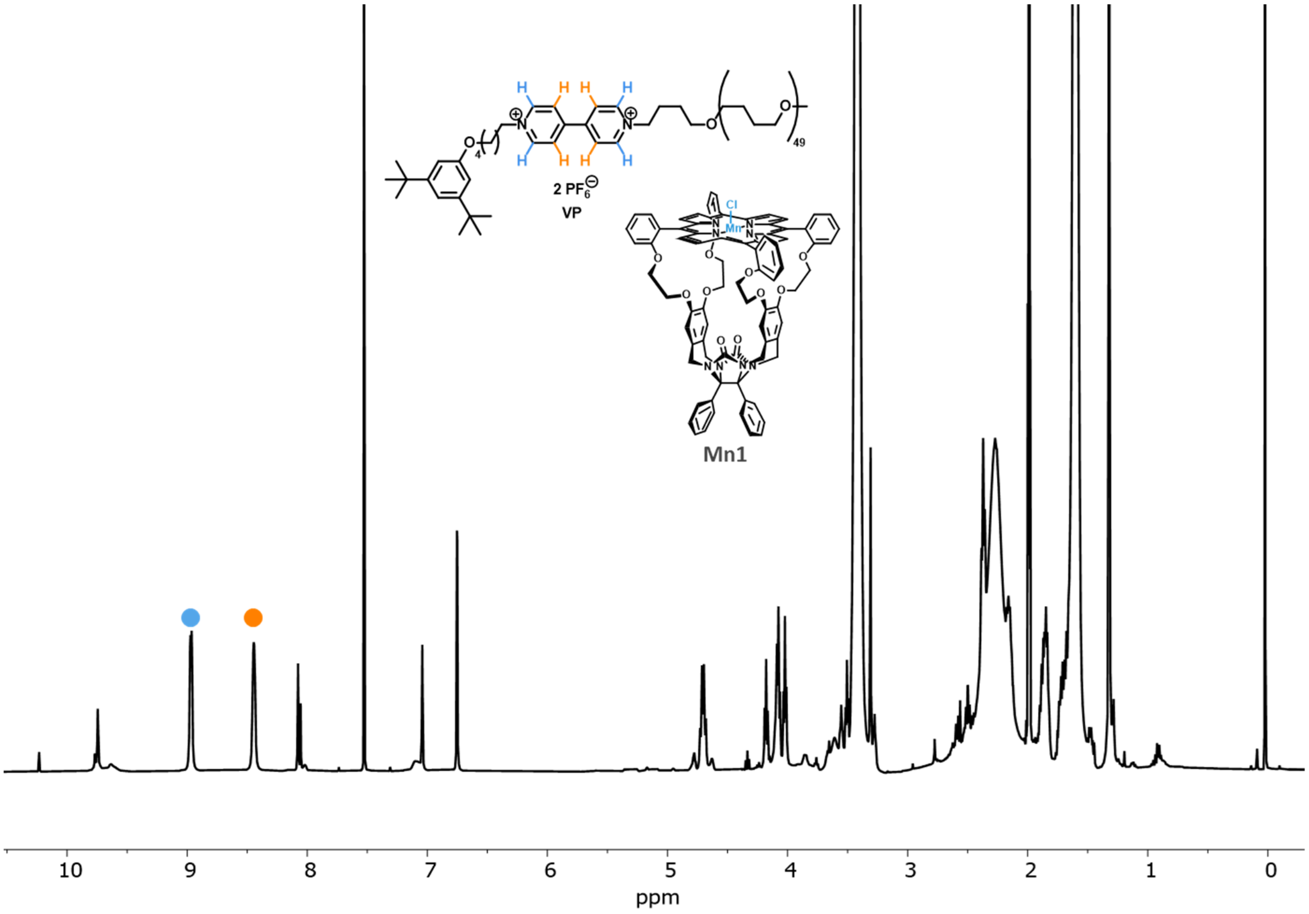

Supplementary Figure 40. ^{1}H NMR spectrum (500 MHz) of Mn1/VP (0.5 mM/5 mM) (acetonitrile-d_3:chloroform-d, 1:1, v/v, 293 K). The signals of unbound **VP** that are selected for the T_1 and T_2 experiments are depicted in blue (ortho-protons) and orange (meta-protons).

3.8. MnRot/V1

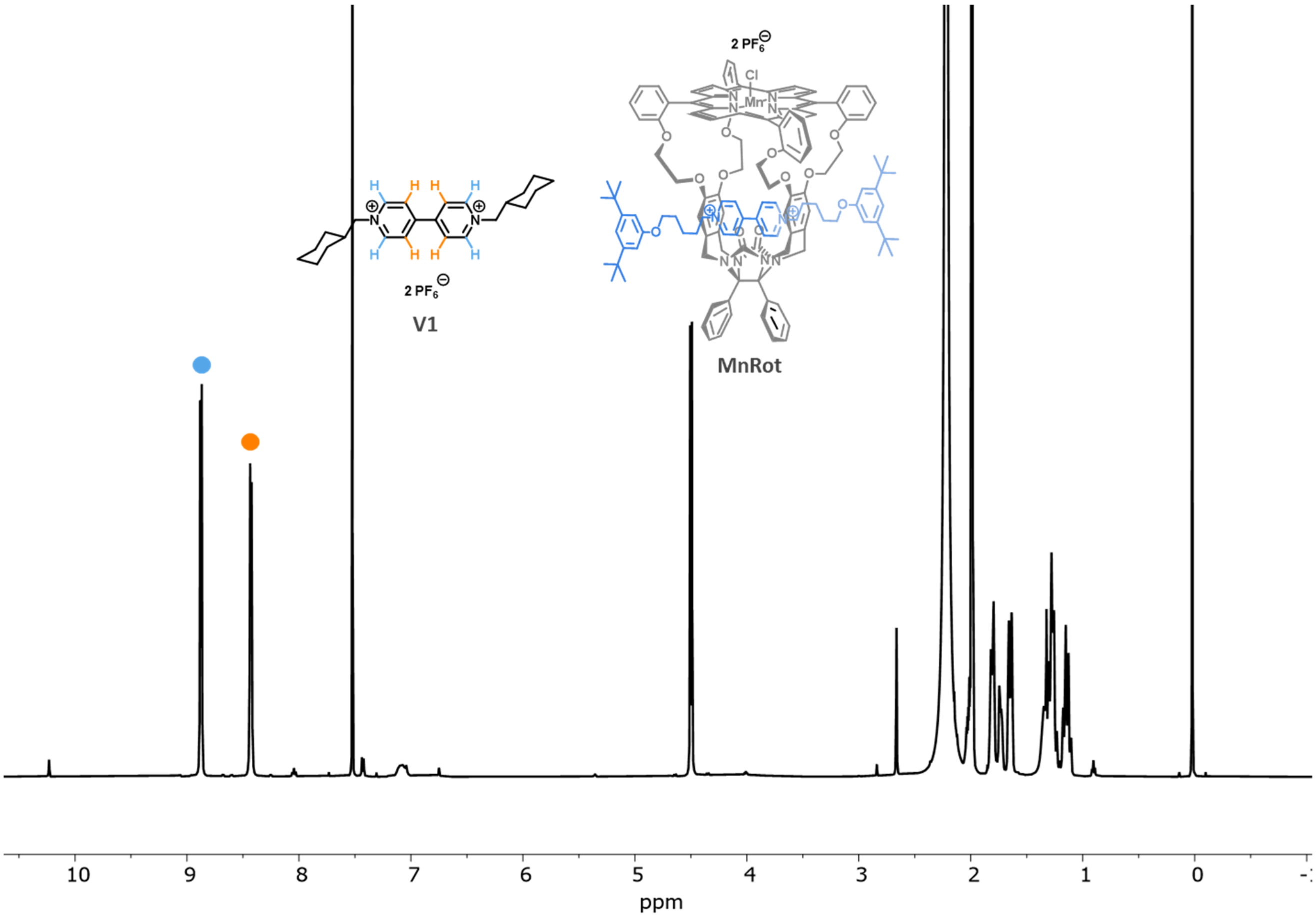

Supplementary Figure 41. ¹H NMR spectrum (500 MHz) of MnRot/V1 (0.5 mM/5 mM) (acetonitrile-d_3:chloroform-*d*, 1:1, v/v, 293 K). The signals of unbound **V1** that are selected for the T_1 and T_2 experiments are depicted in blue (ortho-protons) and orange (meta-protons).

3.9. MnRot/VP

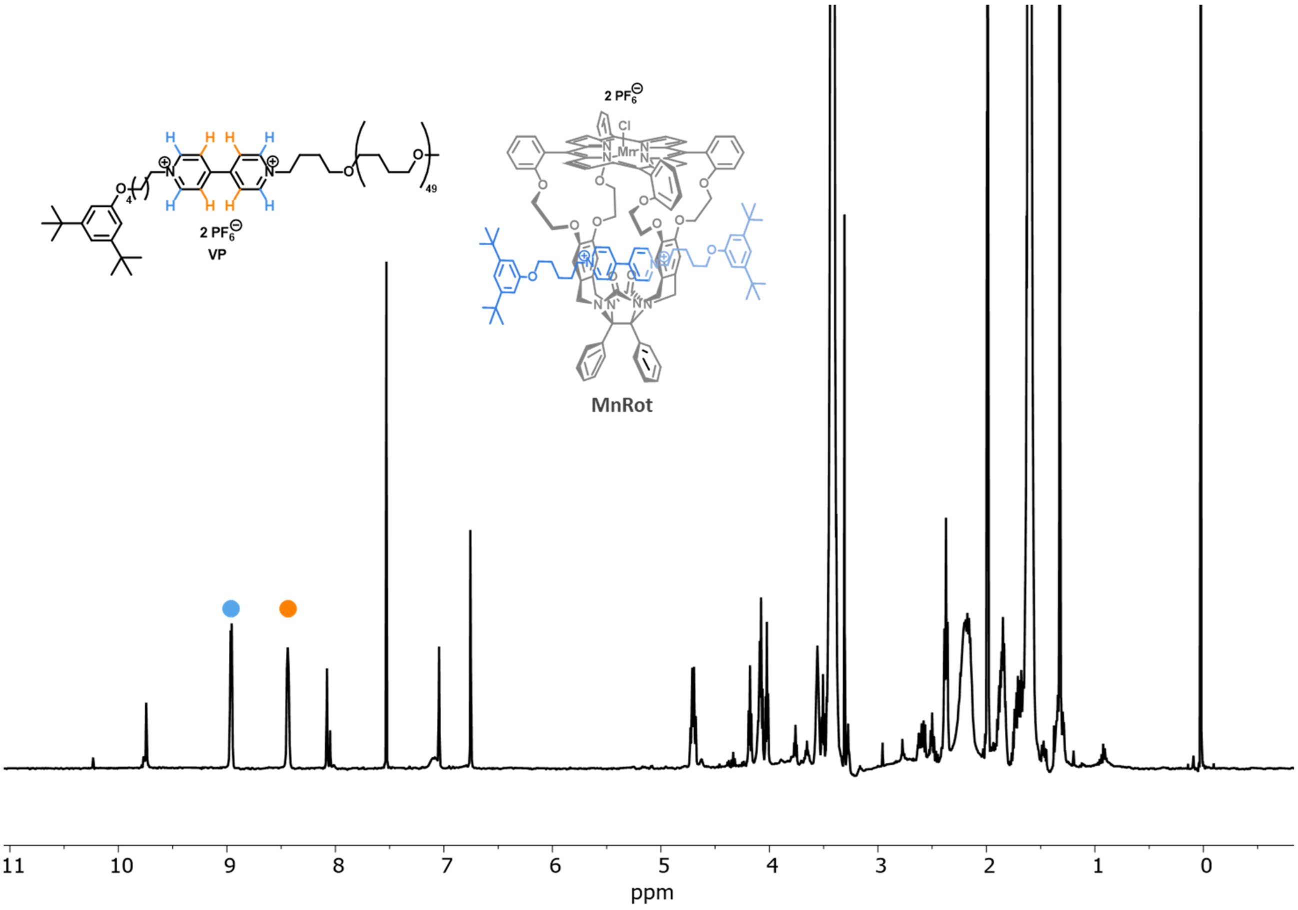

Supplementary Figure 42. ^{1}H NMR spectrum (500 MHz) of MnRot/VP (0.5 mM/5 mM) (acetonitrile-*d_3*:chloroform-*d*, 1:1, v/v, 293 K). The signals of unbound **VP** that are selected for T_1 and T_2 experiments are depicted in blue (ortho-protons) and orange (meta-protons).

4. Exchange: T_1 (inverse recovery) and T_2 (CPMG and PROJECT-CPMG) spectra

4.1. V1 ($T_{1,0}$ and $T_{2,0}$)

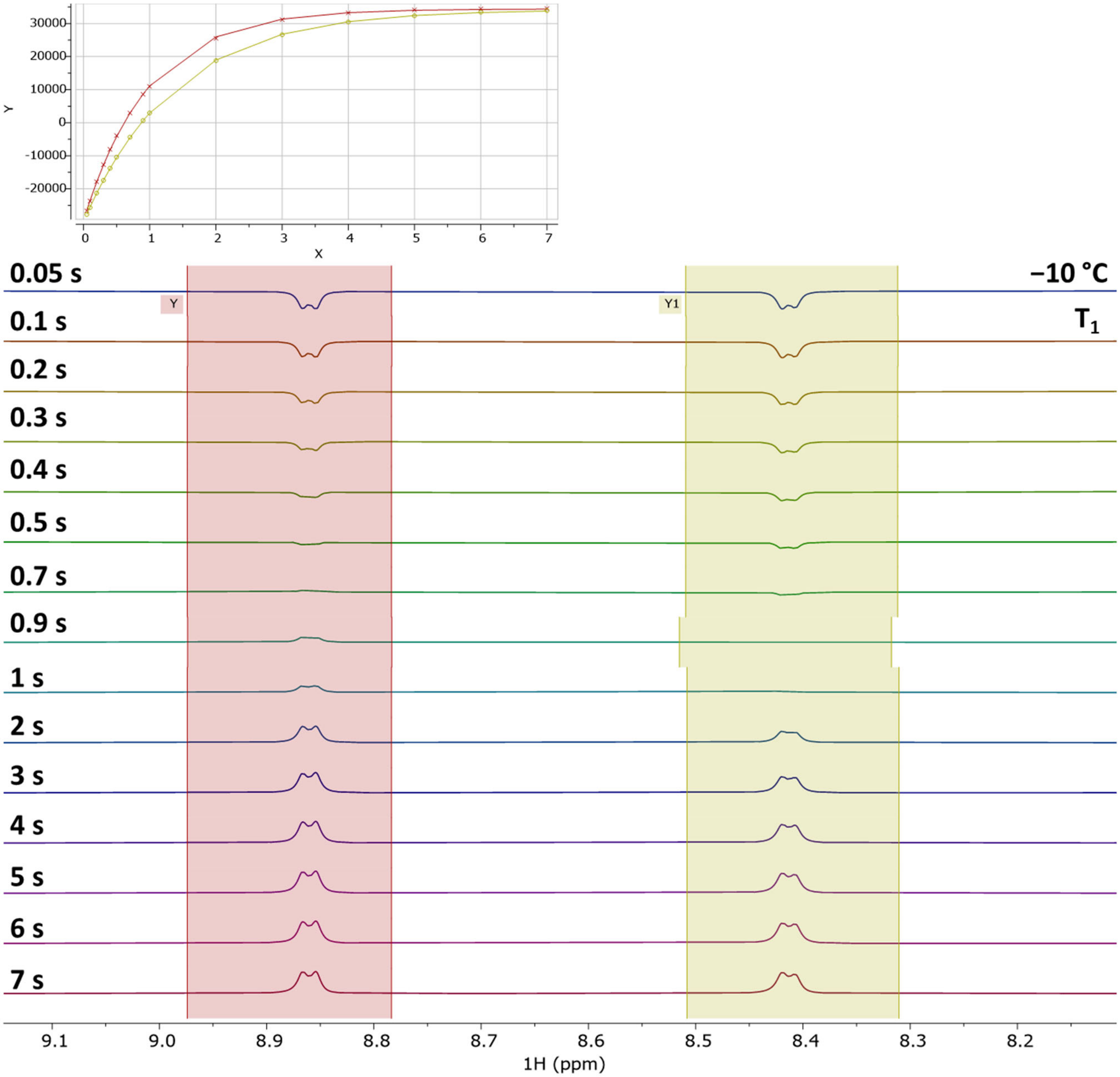

Supplementary Figure 43. T_1 (inverse recovery). Stacked spectra of the inverse recovery experiment to determine the T_1 values of the ortho (Y) and meta (Y1) protons of **V1** in solution, in which the signal intensity (Y) is plotted against the time in seconds (X) (^{1}H, 500 MHz, chloroform-*d* : acetonitrile-d_3, 1:1, v/v, 263 K).

.

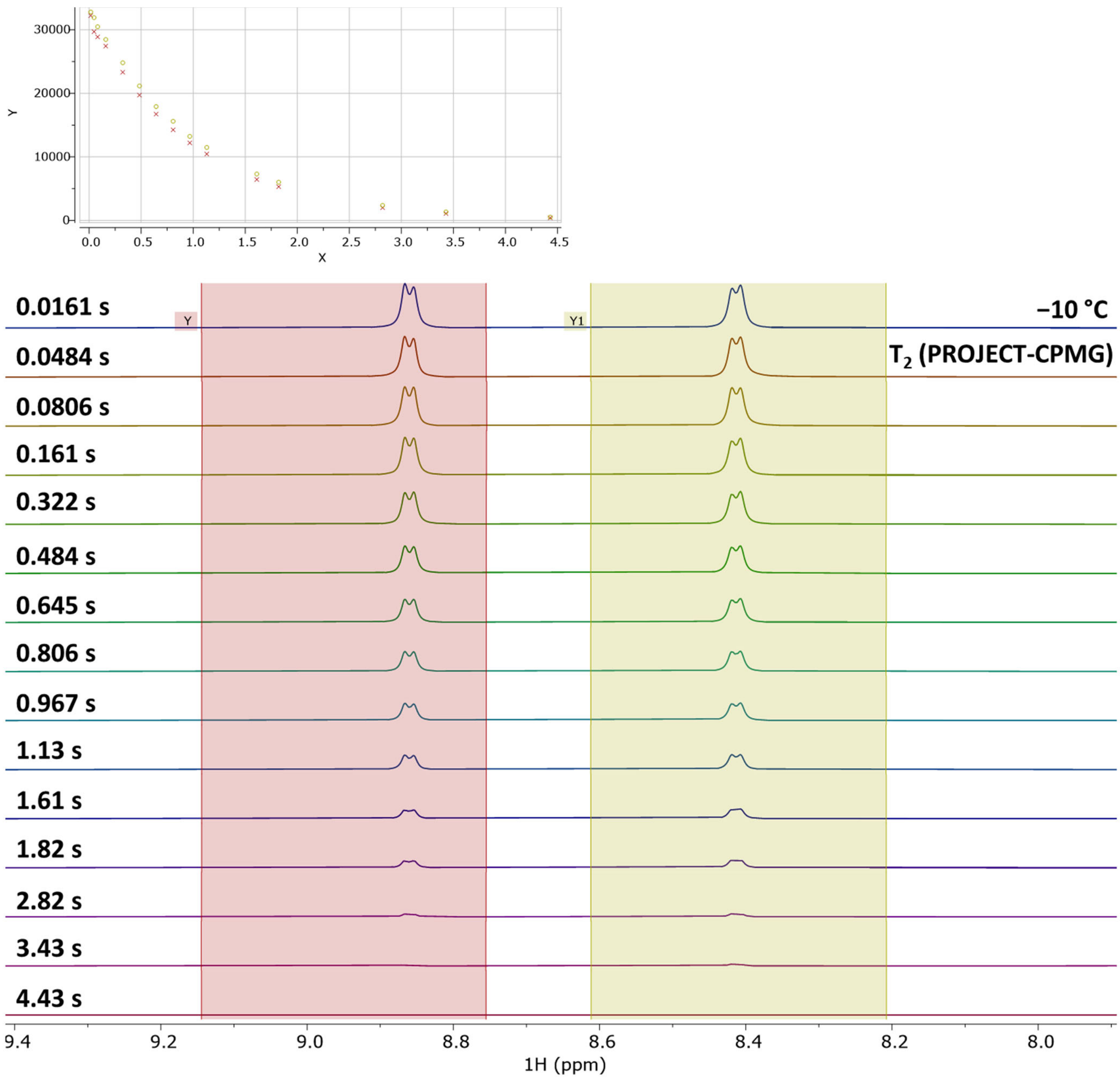

Supplementary Figure 44. T_2 (PROJECT-CPMG). Stacked spectra of the PROJECT-CPMG experiment to determine the T_2 values of the ortho (Y) and meta (Y1) protons of **V1** in solution, in which the signal intensity (Y) is plotted against the time in seconds (X) (^{1}H, 500 MHz, chloroform-*d* : acetonitrile-*d*$_3$, 1:1, v/v, 263 K).

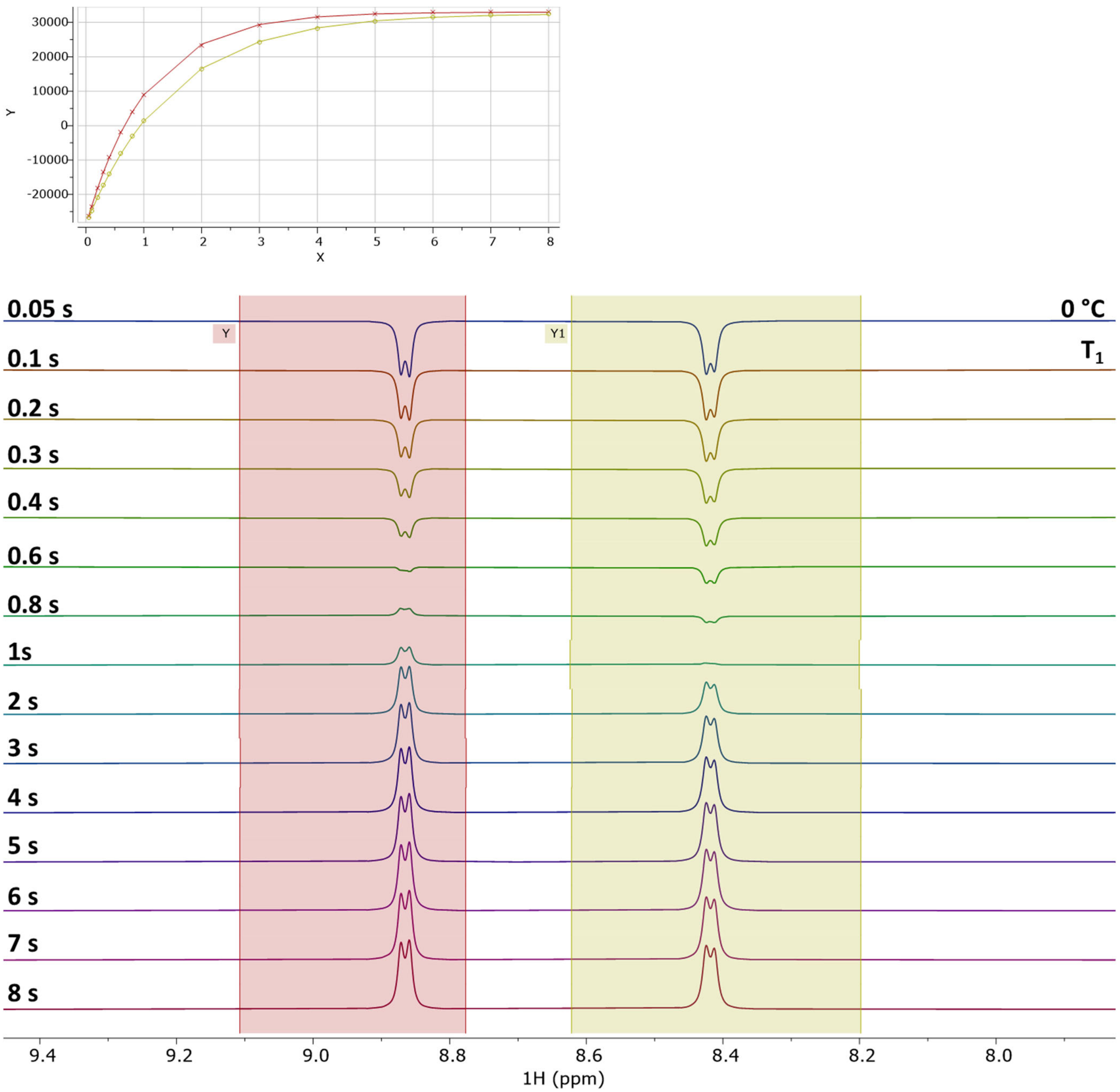

Supplementary Figure 45. T_1 (inverse recovery). Stacked spectra of the inverse recovery experiment to determine the T_1 values of the ortho (Y) and meta (Y1) protons of **V1** in solution, in which the signal intensity (Y) is plotted against the time in seconds (X) (^{1}H, 500 MHz, chloroform-*d* : acetonitrile-*d₃*, 1:1, v/v, 273 K).

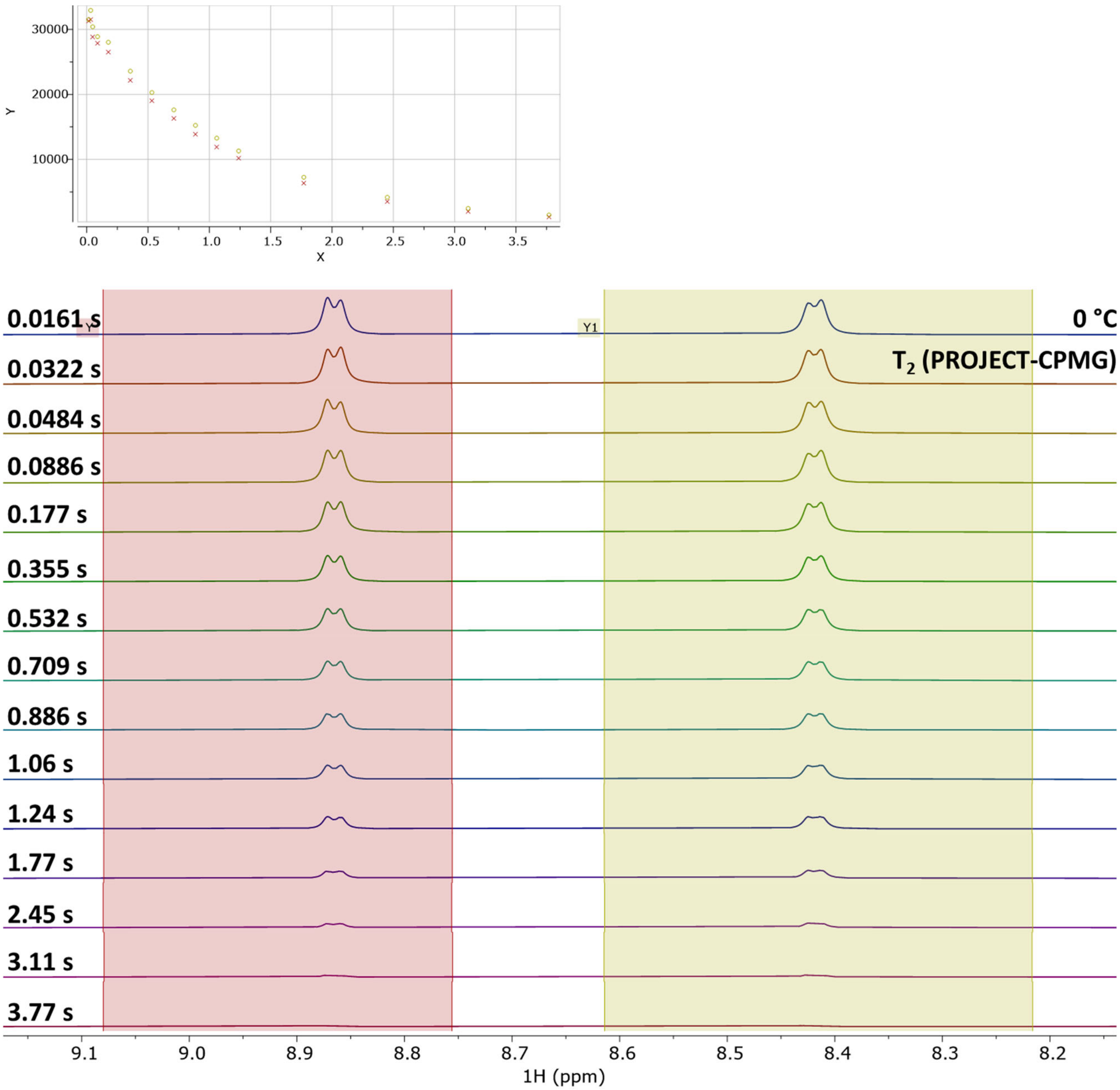

Supplementary Figure 46. T_2 (PROJECT-CPMG). Stacked spectra of the PROJECT-CPMG experiment to determine the T_2 values of the ortho (Y) and meta (Y1) protons of **V1** in solution, in which the signal intensity (Y) is plotted against the time in seconds (X) (^{1}H, 500 MHz, chloroform-*d* : acetonitrile-*d*$_3$, 1:1, v/v, 273 K).

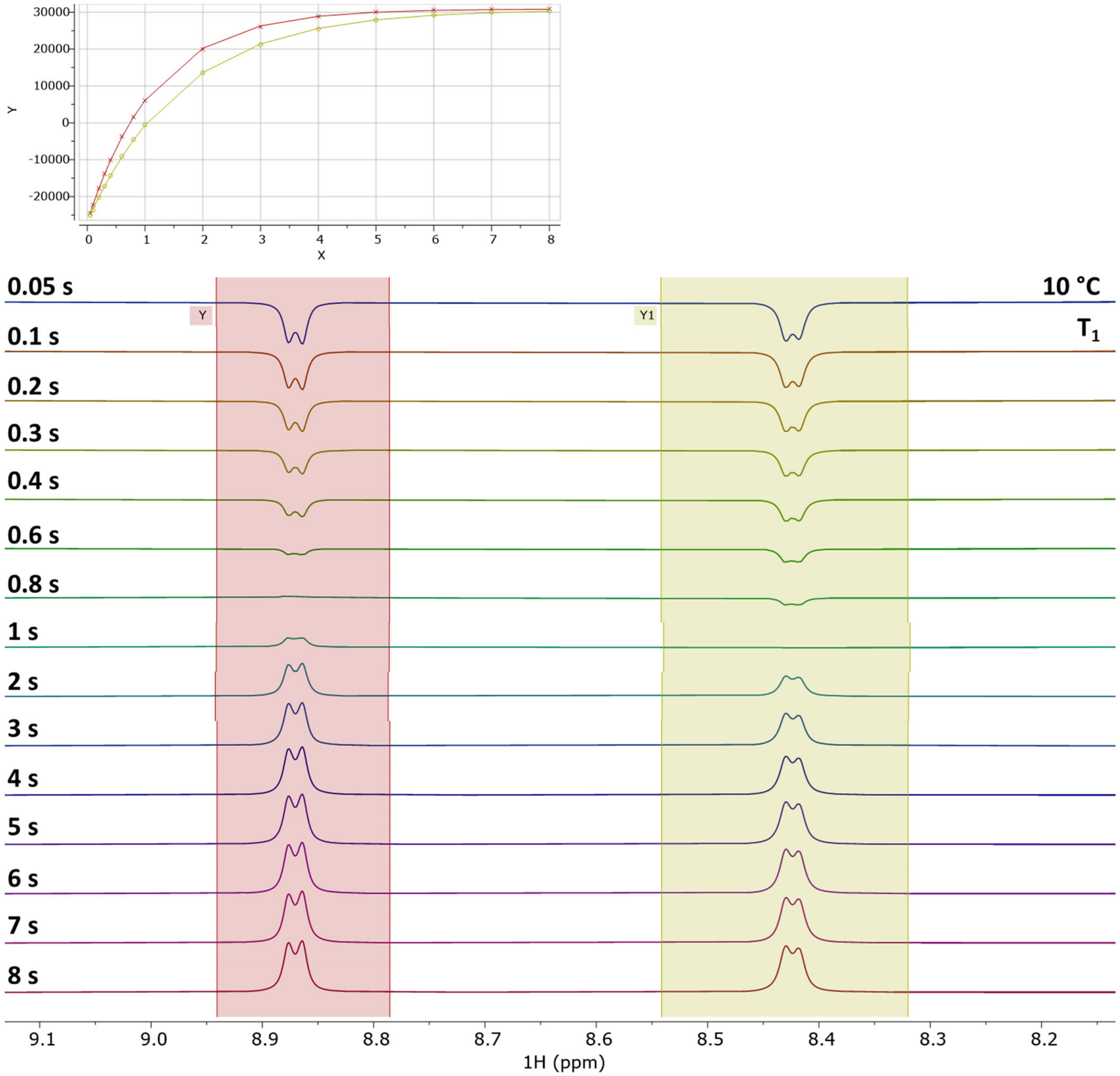

Supplementary Figure 47. T_1 (inverse recovery). Stacked spectra of the inverse recovery experiment to determine the T_1 values of the ortho (Y) and meta (Y1) protons of **V1** in solution, in which the signal intensity (Y) is plotted against the time in seconds (X) (^{1}H, 500 MHz, chloroform-*d* : acetonitrile-*d*$_3$, 1:1, v/v, 283 K).

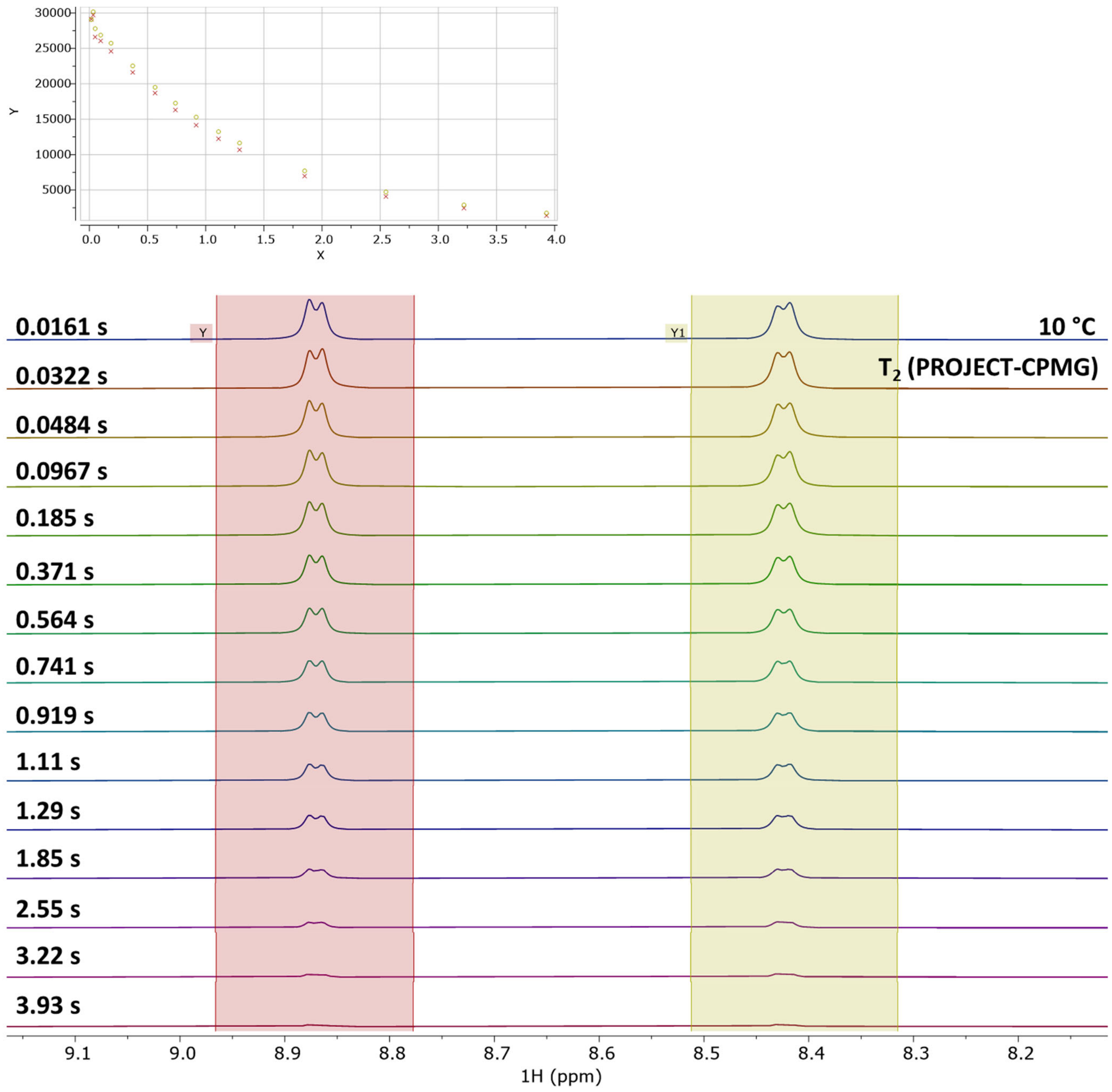

Supplementary Figure 48. T_2 (PROJECT-CPMG). Stacked spectra of the PROJECT-CPMG experiment to determine the T_2 values of the ortho (Y) and meta (Y1) protons of **V1** in solution, in which the signal intensity (Y) is plotted against the time in seconds (X) (^{1}H, 500 MHz, chloroform-*d* : acetonitrile-*d*$_3$, 1:1, v/v, 283 K).

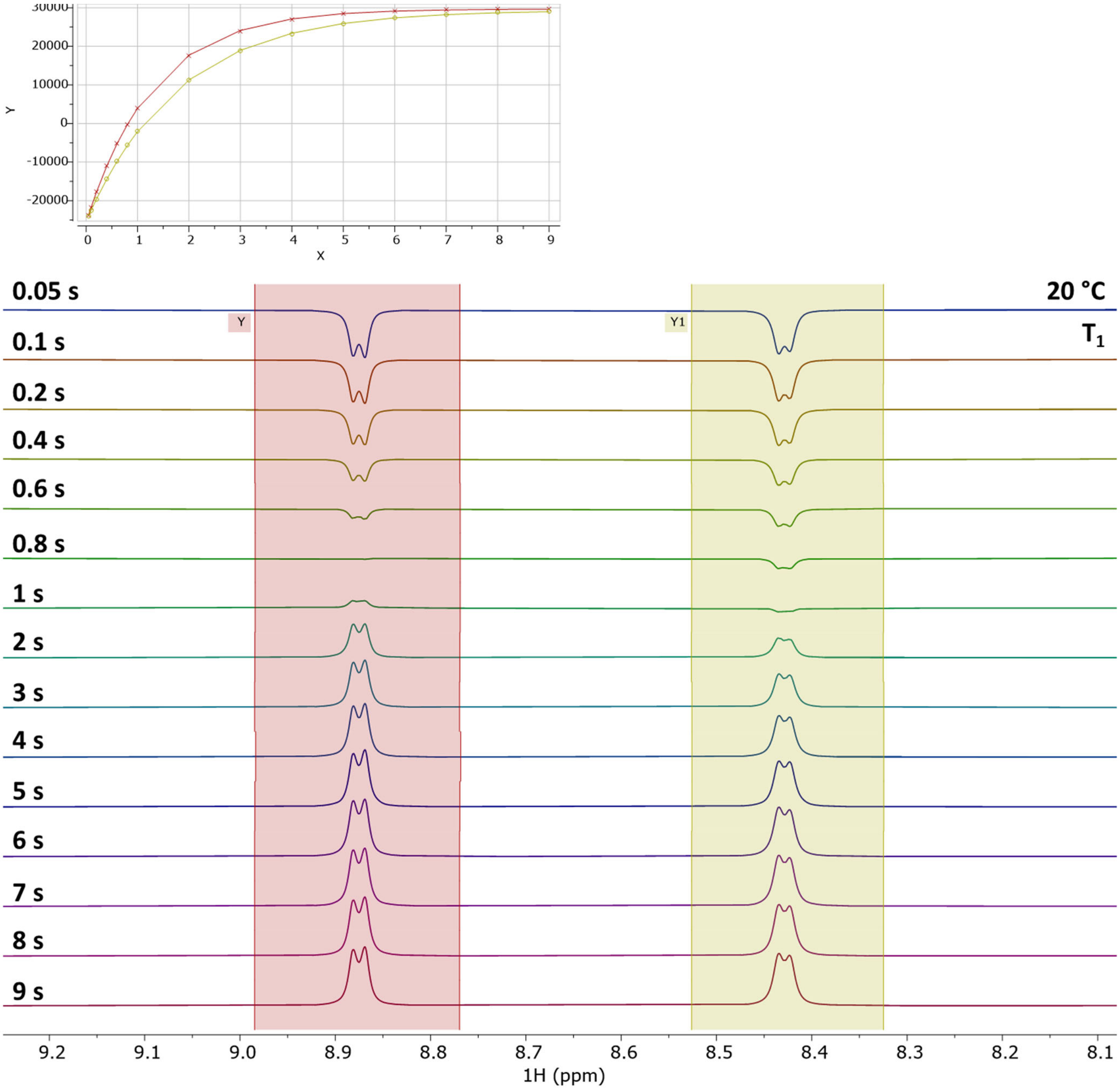

Supplementary Figure 49. T_1 (inverse recovery). Stacked spectra of the inverse recovery experiment to determine the T_1 values of the ortho (Y) and meta (Y1) protons of **V1** in solution, in which the signal intensity (Y) is plotted against the time in seconds (X) (^{1}H, 500 MHz, chloroform-*d* : acetonitrile-*d*$_3$, 1:1, v/v, 293 K).

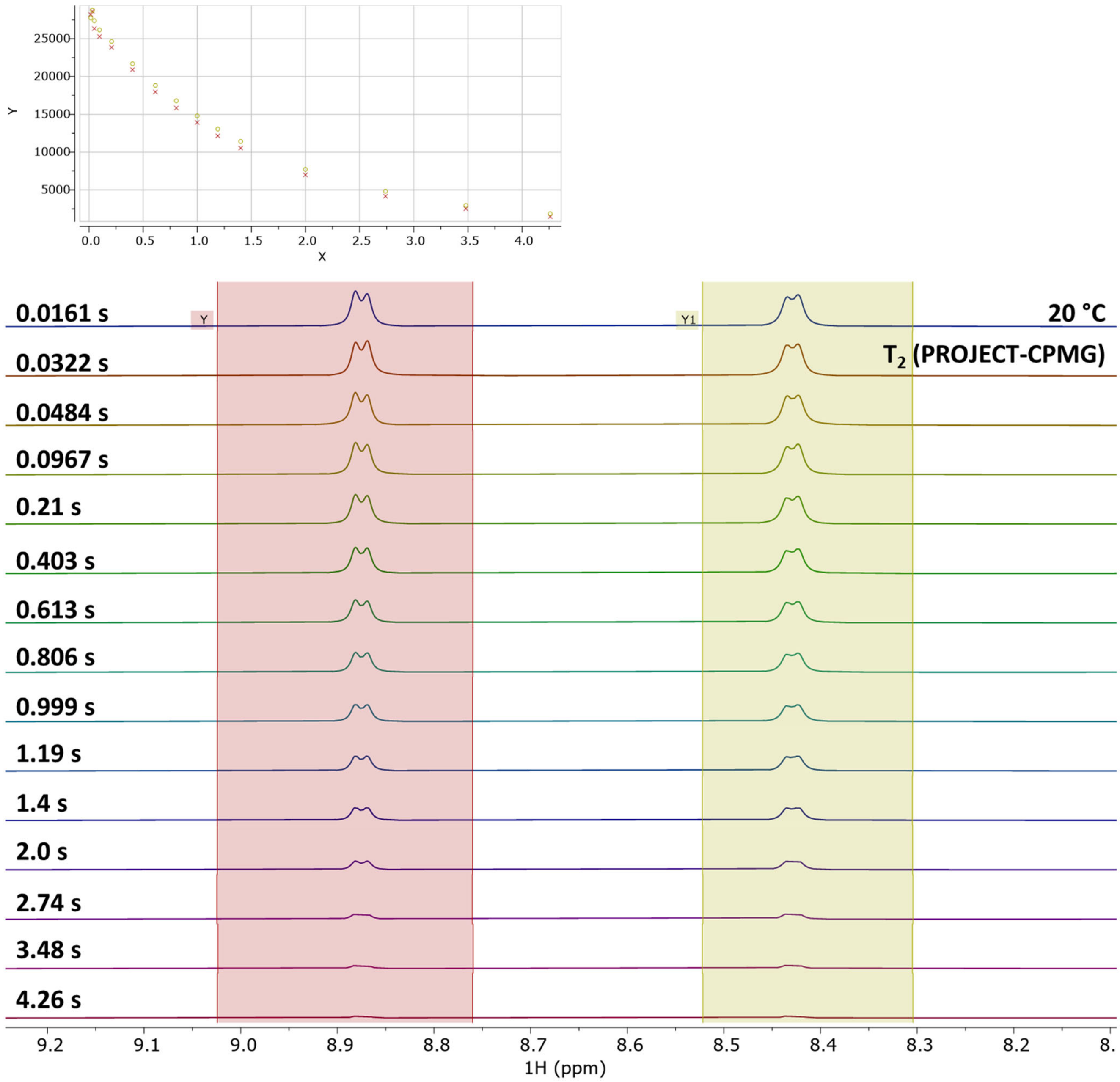

Supplementary Figure 50. T_2 (PROJECT-CPMG). Stacked spectra of the PROJECT-CPMG experiment to determine the T_2 values of the ortho (Y) and meta (Y1) protons of **V1** in solution, in which the signal intensity (Y) is plotted against the time in seconds (X) (^{1}H, 500 MHz, chloroform-*d* : acetonitrile-*d*$_3$, 1:1, v/v, 293 K).

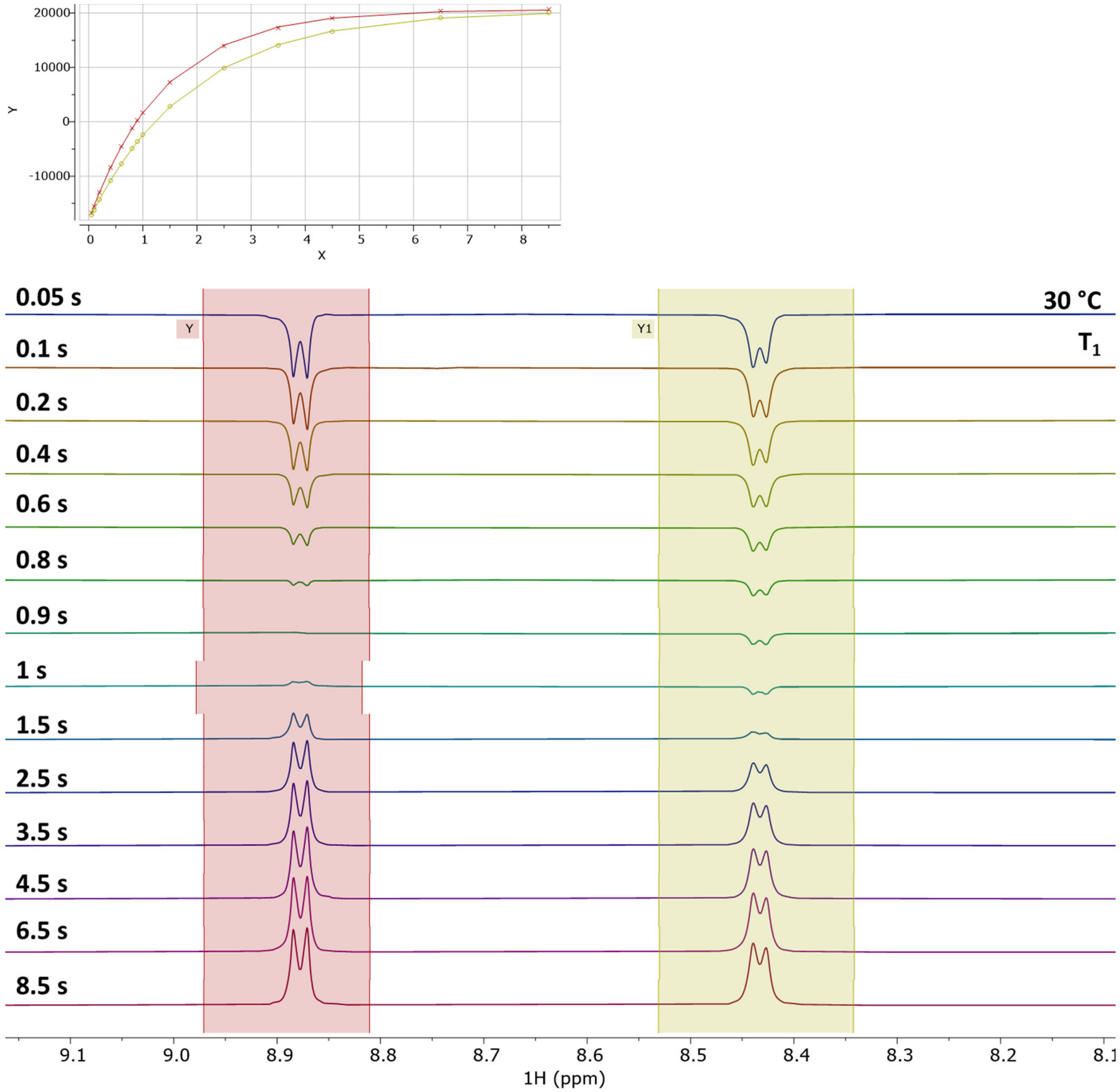

Supplementary Figure 51. T_1 (inverse recovery). Stacked spectra of the inverse recovery experiment to determine the T_1 values of the ortho (Y) and meta (Y1) protons of **V1** in solution, in which the signal intensity (Y) is plotted against the time in seconds (X) (^{1}H, 500 MHz, chloroform-*d* : acetonitrile-d_3, 1:1, v/v, 303 K).

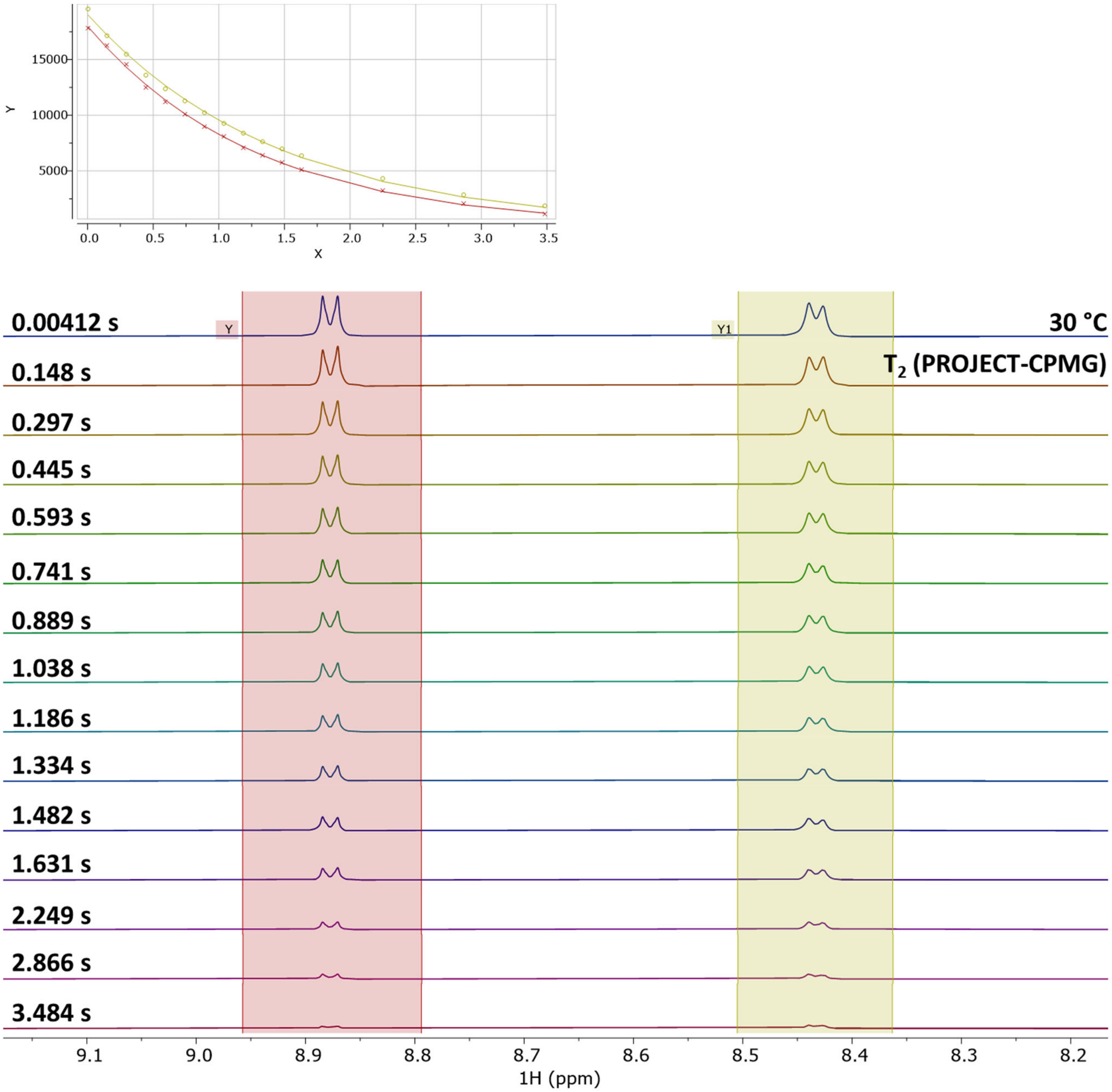

Supplementary Figure 52. T_2 (PROJECT-CPMG). Stacked spectra of the PROJECT-CPMG experiment to determine the T_2 values of the ortho (Y) and meta (Y1) protons of **V1** in solution, in which the signal intensity (Y) is plotted against the time in seconds (X) (^{1}H, 500 MHz, chloroform-*d* : acetonitrile-d_3, 1:1, v/v, 303 K).

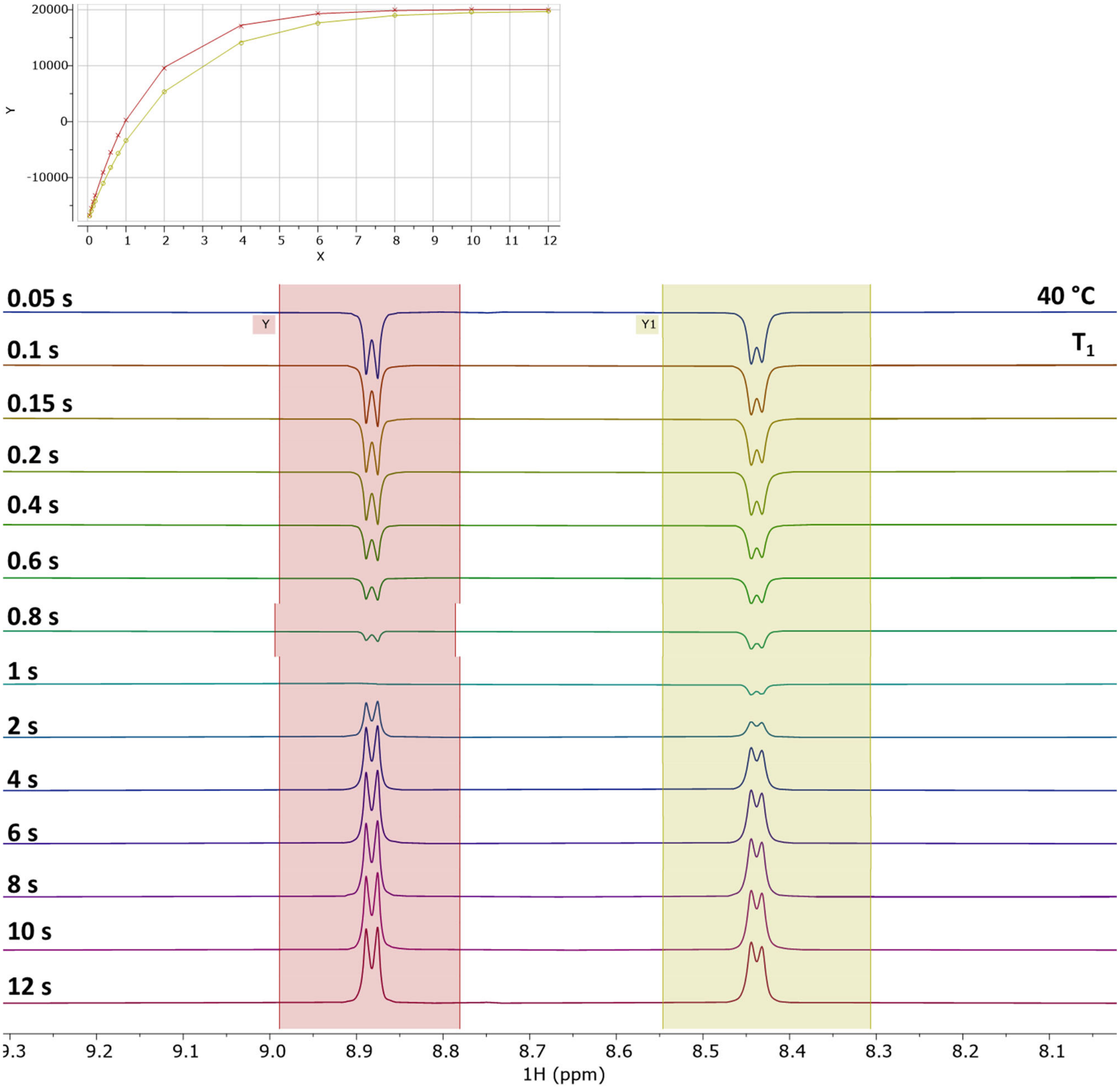

Supplementary Figure 53. T_1 (inverse recovery). Stacked spectra of the inverse recovery experiment to determine the T_1 values of the ortho (Y) and meta (Y1) protons of **V1** in solution, in which the signal intensity (Y) is plotted against the time in seconds (X) (^{1}H, 500 MHz, chloroform-*d* : acetonitrile-d_3, 1:1, v/v, 313 K).

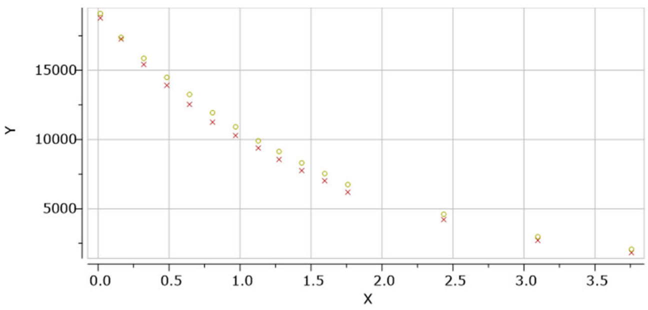

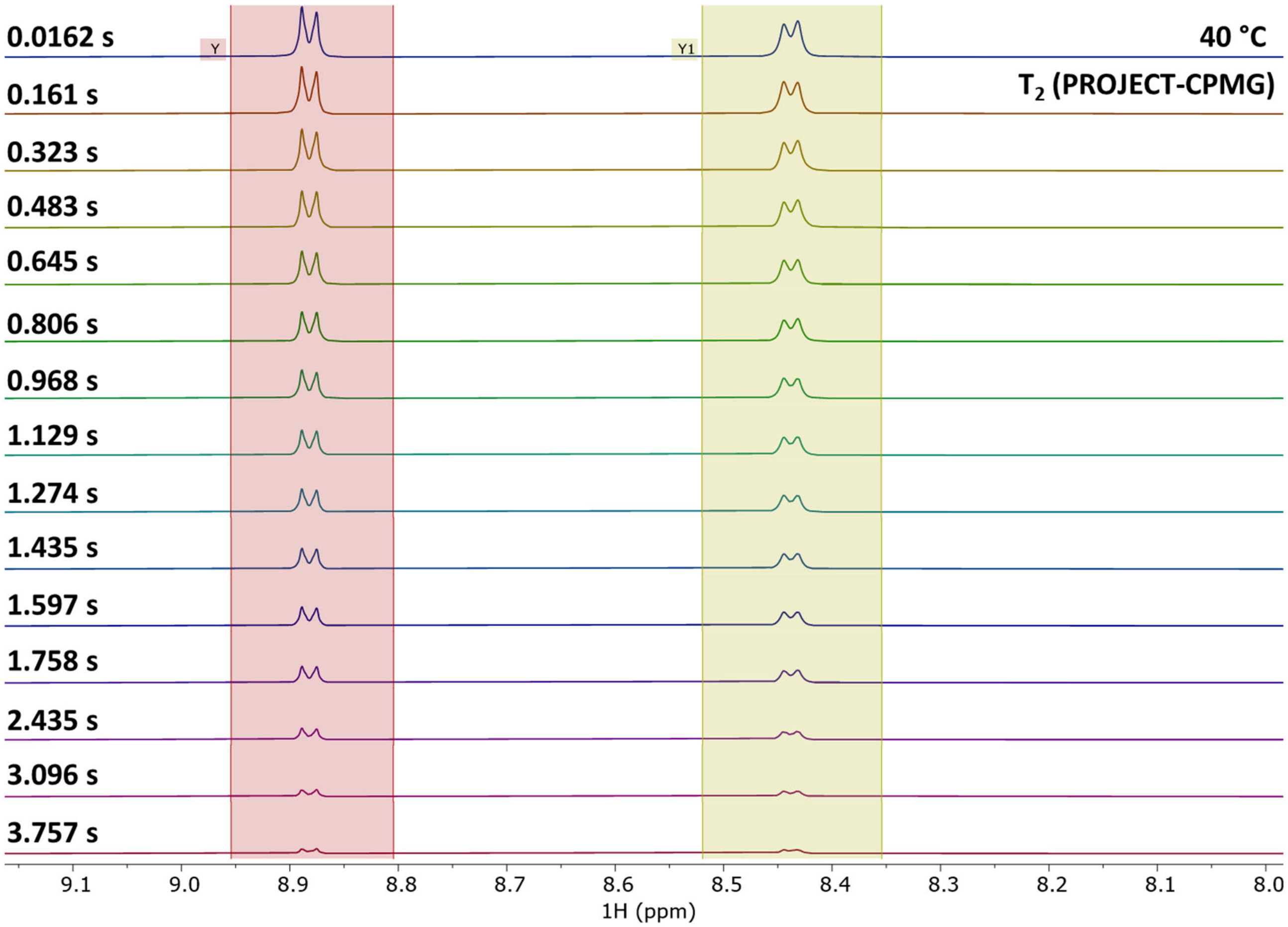

Supplementary Figure 54. T_2 (PROJECT-CPMG). Stacked spectra of the PROJECT-CPMG experiment to determine the T_2 values of the ortho (Y) and meta (Y1) protons of **V1** in solution, in which the signal intensity (Y) is plotted against the time in seconds (X) (^{1}H, 500 MHz, chloroform-*d* : acetonitrile-*d*$_3$, 1:1, v/v, 313 K).

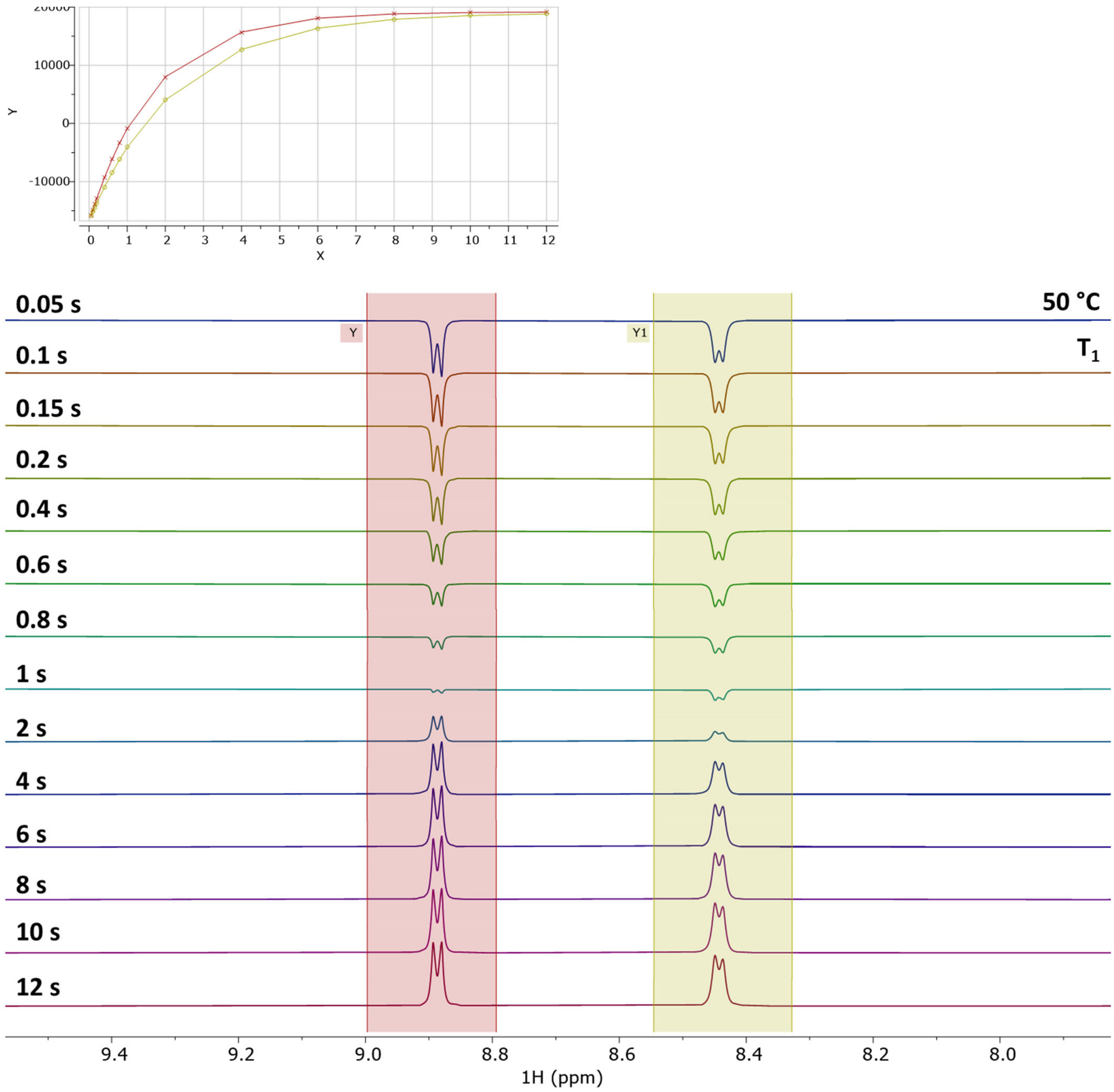

Supplementary Figure 55. T_1 (inverse recovery). Stacked spectra of the inverse recovery experiment to determine the T_1 values of the ortho (Y) and meta (Y1) protons of **V1** in solution, in which the signal intensity (Y) is plotted against the time in seconds (X) (^{1}H, 500 MHz, chloroform-*d* : acetonitrile-*d*$_3$, 1:1, v/v, 323 K).

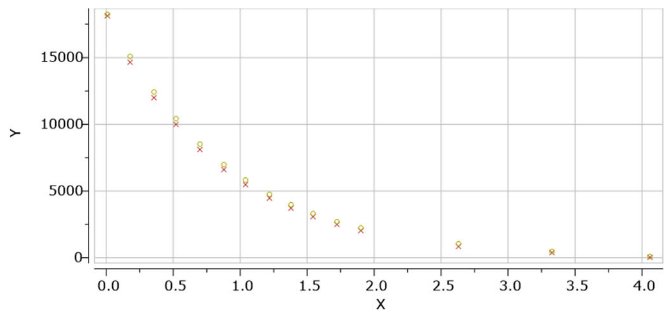

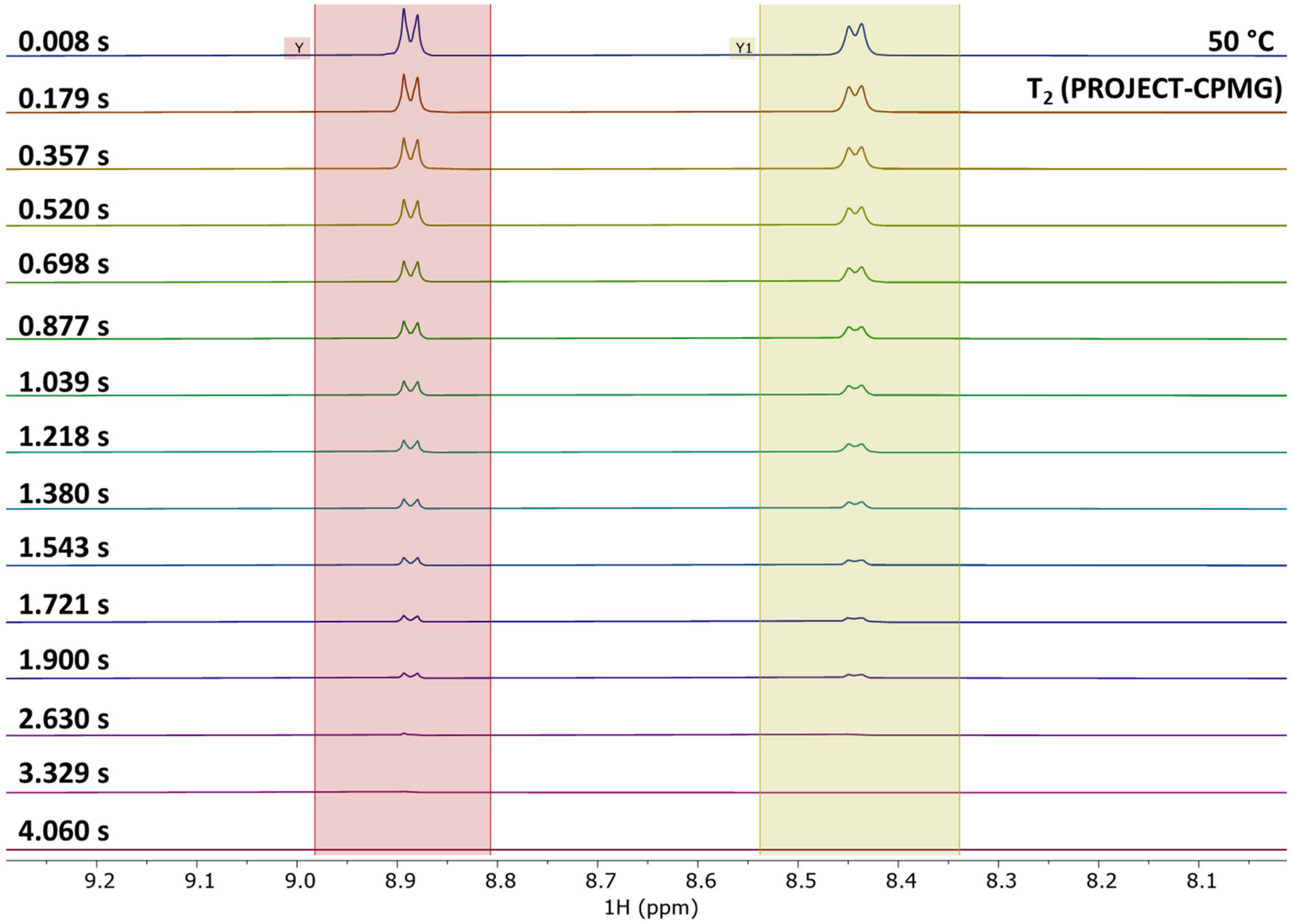

Supplementary Figure 56. T_2 (PROJECT-CPMG). Stacked spectra of the PROJECT-CPMG experiment to determine the T_2 values of the ortho (Y) and meta (Y1) protons of **V1** in solution, in which the signal intensity (Y) is plotted against the time in seconds (X) (^{1}H, 500 MHz, chloroform-*d* : acetonitrile-d_3, 1:1, v/v, 323 K).

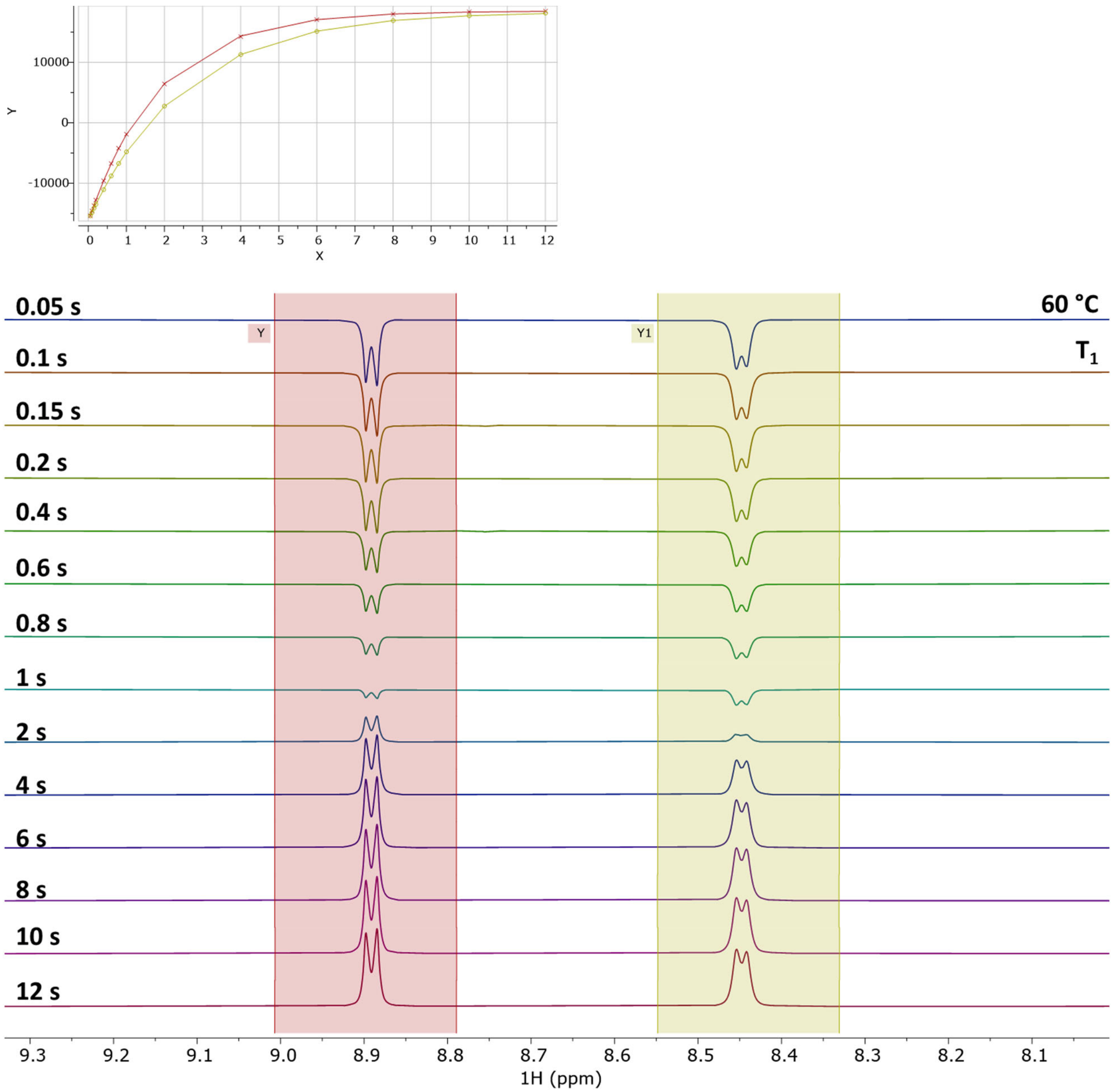

Supplementary Figure 57. T_1 (inverse recovery). Stacked spectra of the inverse recovery experiment to determine the T_1 values of the ortho (Y) and meta (Y1) protons of **V1** in solution, in which the signal intensity (Y) is plotted against the time in seconds (X) (^{1}H, 500 MHz, chloroform-*d* : acetonitrile-d_3, 1:1, v/v, 333 K).

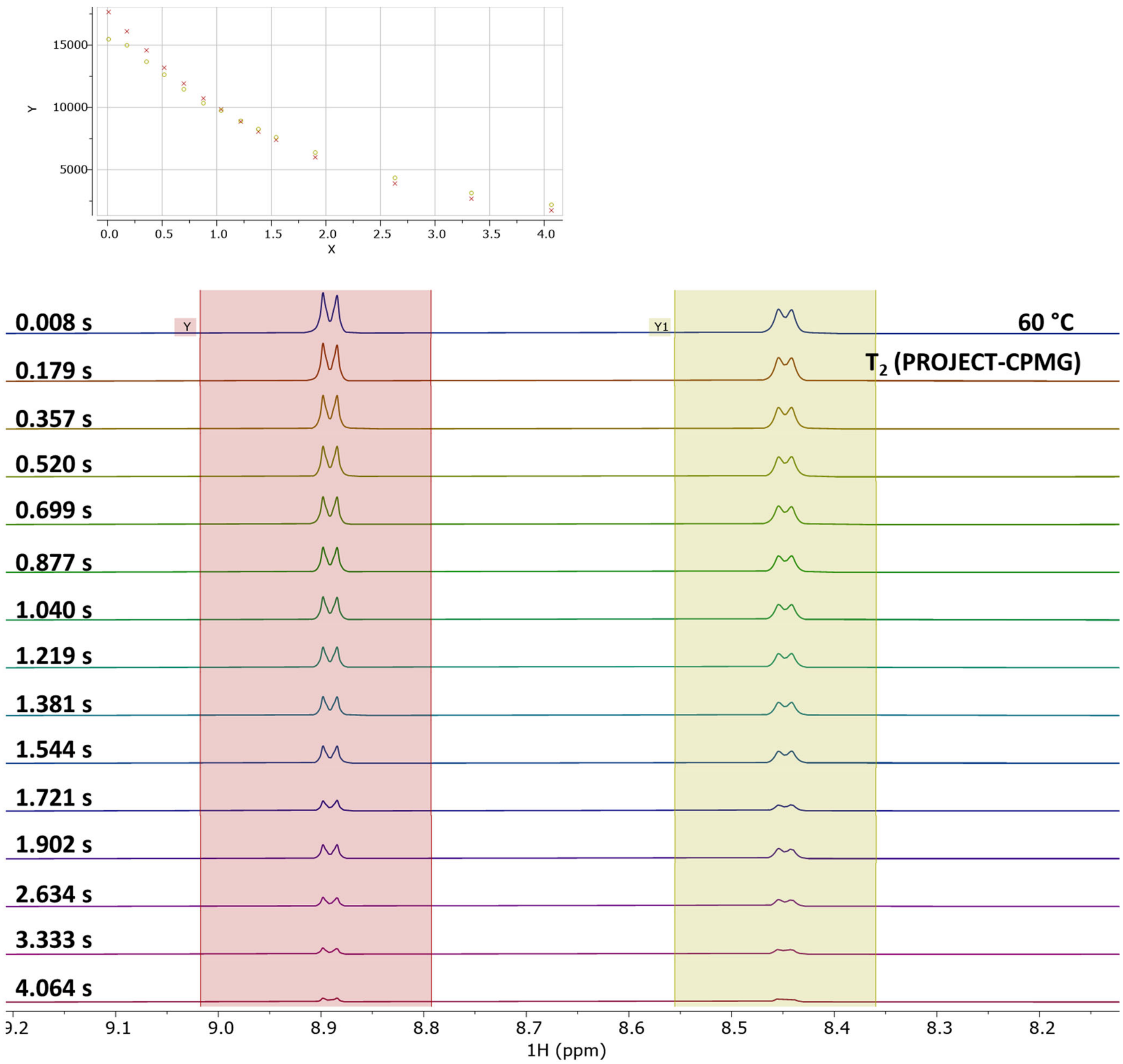

Supplementary Figure 58. T_2 (PROJECT-CPMG). Stacked spectra of the PROJECT-CPMG experiment to determine the T_2 values of the ortho (Y) and meta (Y1) protons of **V1** in solution, in which the signal intensity (Y) is plotted against the time in seconds (X) (^{1}H, 500 MHz, chloroform-*d* : acetonitrile-*d*$_3$, 1:1, v/v, 333 K).

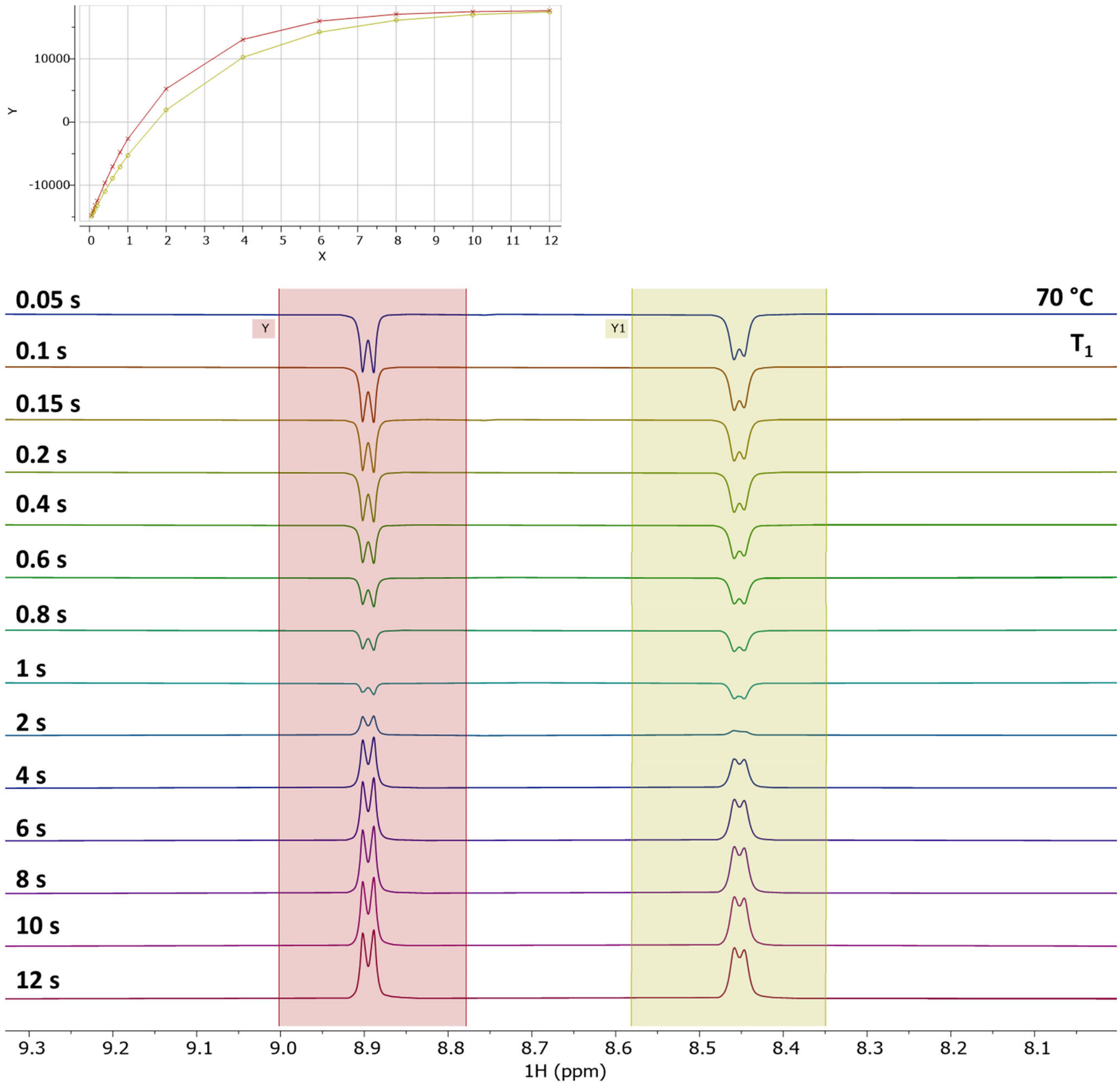

Supplementary Figure 59. T_1 (inverse recovery). Stacked spectra of the inverse recovery experiment to determine the T_1 values of the ortho (Y) and meta (Y1) protons of **V1** in solution, in which the signal intensity (Y) is plotted against the time in seconds (X) (^{1}H, 500 MHz, chloroform-*d* : acetonitrile-d_3, 1:1, v/v, 343 K).

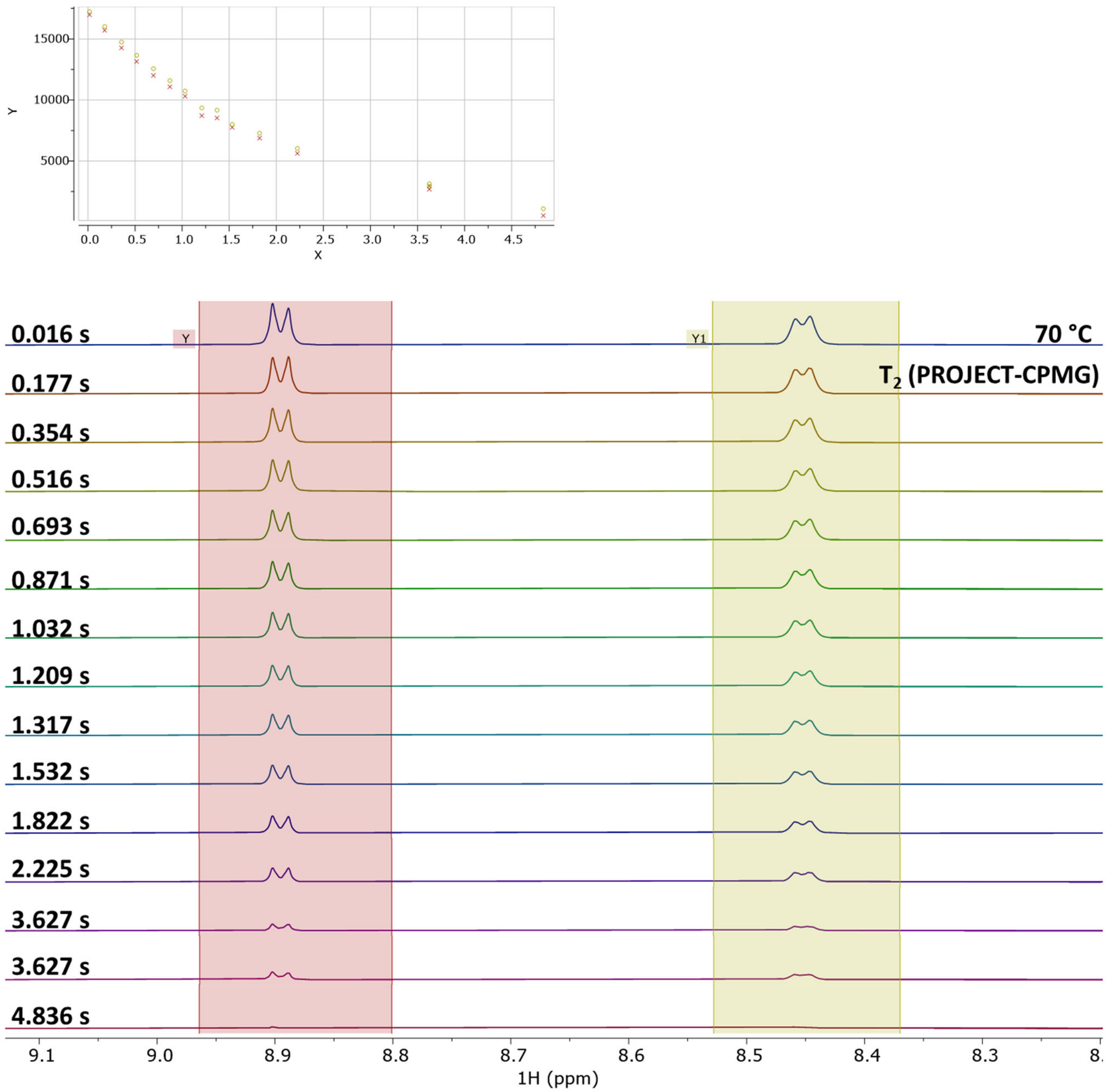

Supplementary Figure 60. T_2 (PROJECT-CPMG). Stacked spectra of the PROJECT-CPMG experiment to determine the T_2 values of the ortho (Y) and meta (Y1) protons of **V1** in solution, in which the signal intensity (Y) is plotted against the time in seconds (X) (^{1}H, 500 MHz, chloroform-*d* : acetonitrile-*d*$_3$, 1:1, v/v, 343 K).

4.2. Mn1/V1 ($T_{1,obs}$ and $T_{2,obs}$)

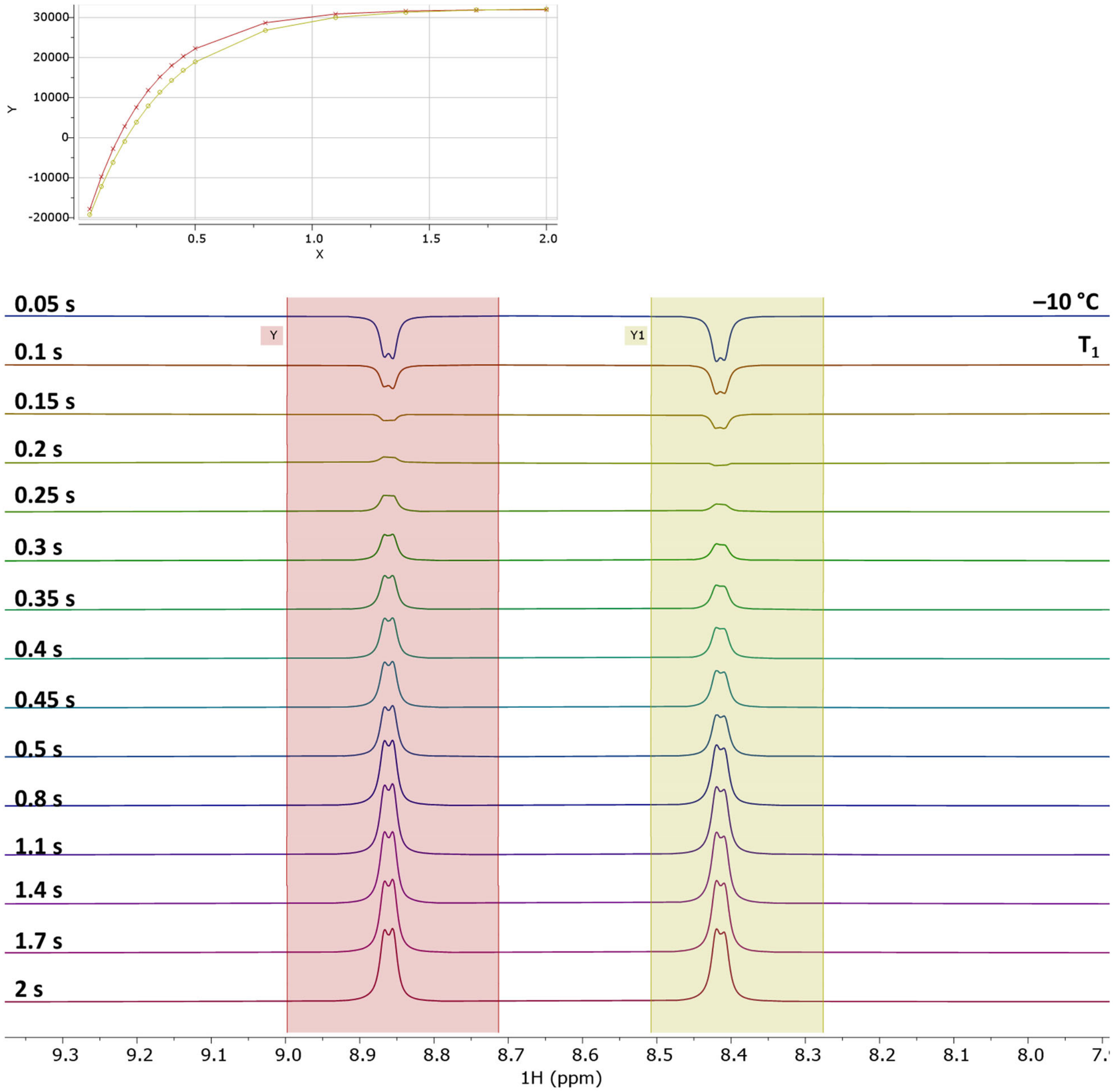

Supplementary Figure 61. T_1 (inverse recovery). Stacked spectra of the inverse recovery experiment to determine the T_1 values of the ortho (Y) and meta (Y1) protons of **Mn1** (0.5 mM) and **V1** (5 mM) in solution, in which the signal intensity (Y) is plotted against the time in seconds (X) (^{1}H, 500 MHz, chloroform-*d* : acetonitrile-*d₃*, 1:1, v/v, 263 K).

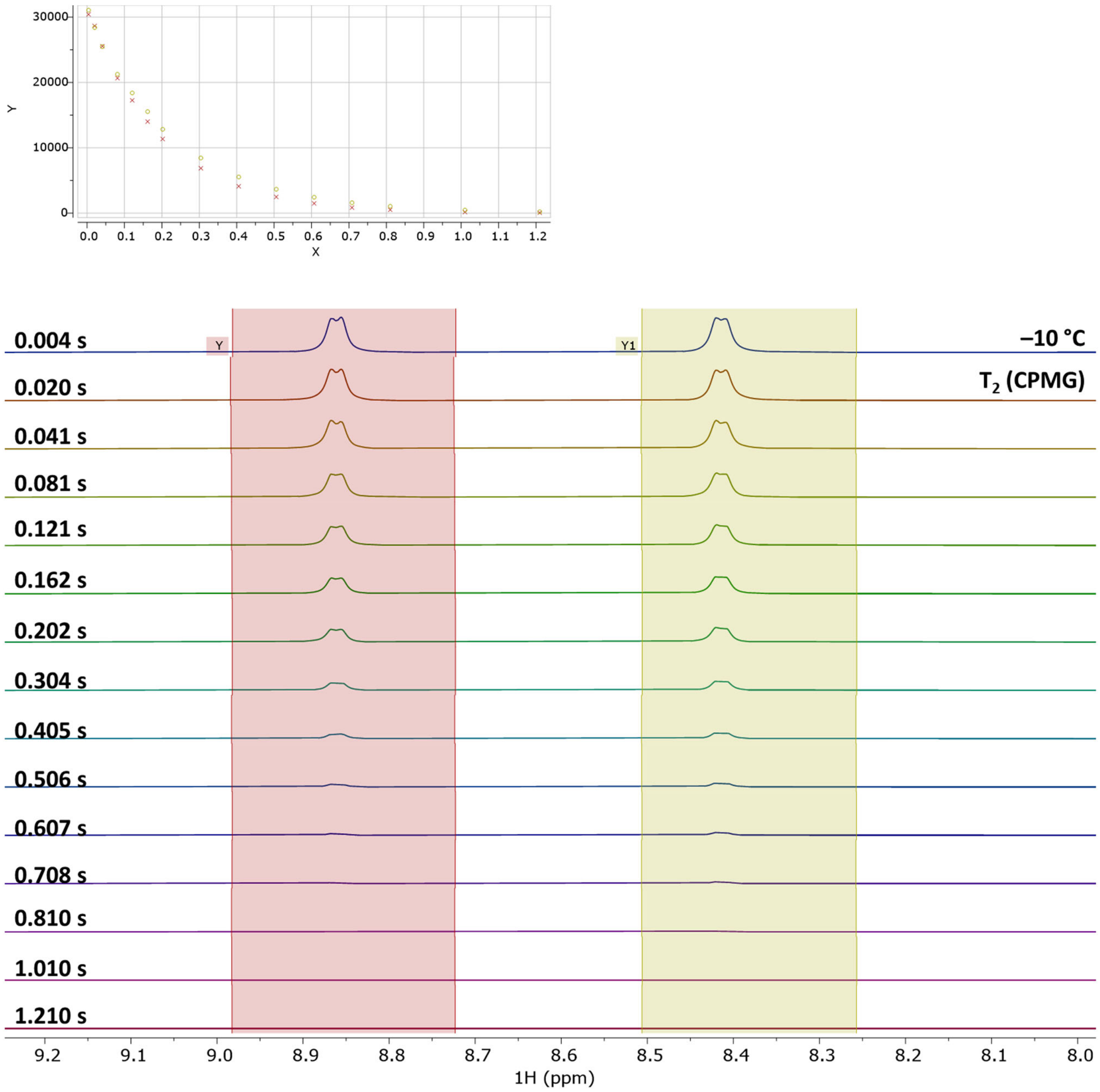

Supplementary Figure 62. T_2 (CPMG). Stacked spectra of the CPMG experiment to determine the T_2 values of the ortho (Y) and meta (Y1) protons of **Mn1** (0.5 mM) and **V1** (5 mM) in solution, in which the signal intensity (Y) is plotted against the time in seconds (X) (^{1}H, 500 MHz, chloroform-*d* : acetonitrile-*d*$_3$, 1:1, v/v, 263 K).

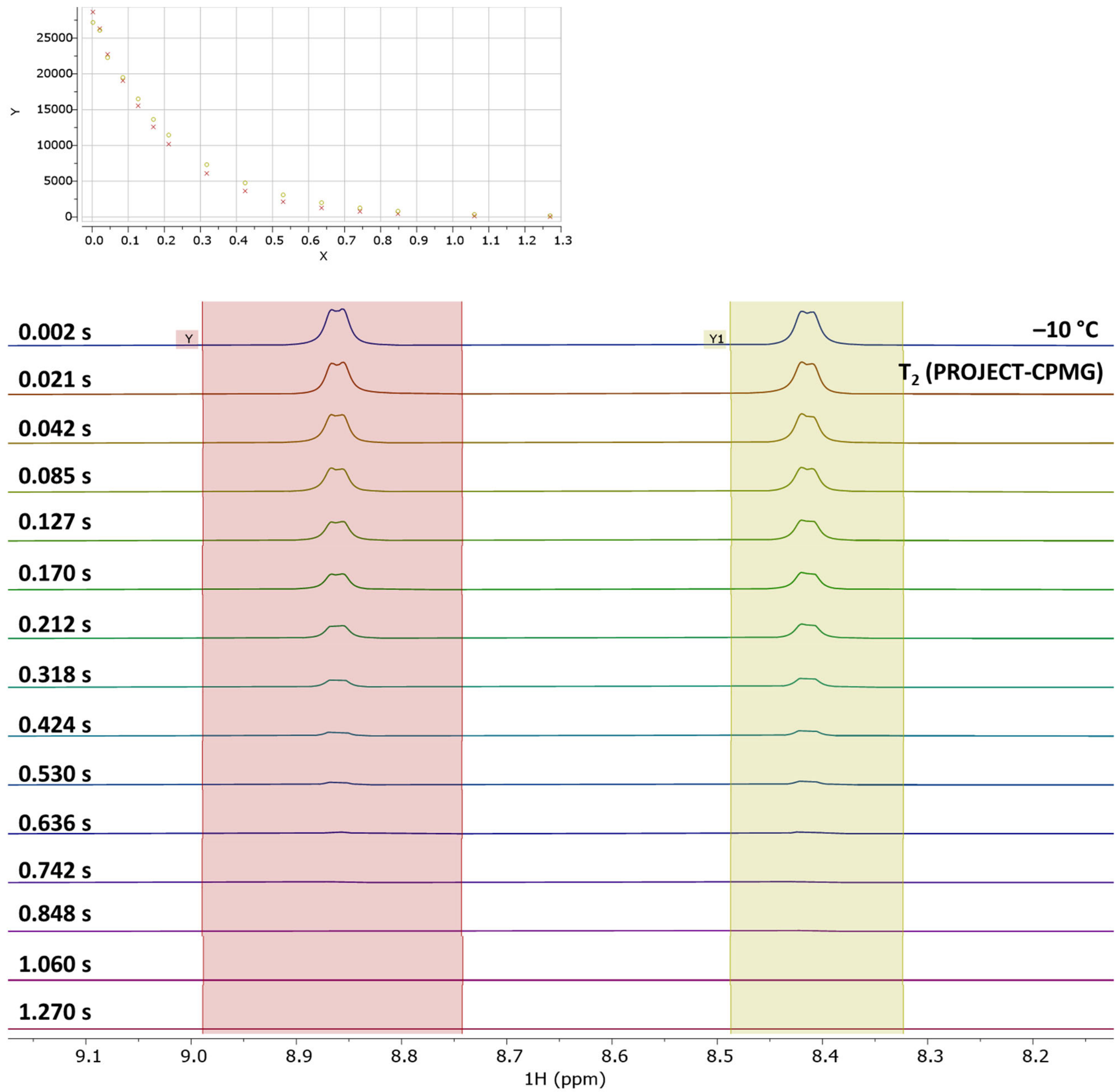

Supplementary Figure 63. T_2 (PROJECT-CPMG). Stacked spectra of the PROJECT-CPMG experiment to determine the T_2 values of the ortho (Y) and meta (Y1) protons of **Mn1** (0.5 mM) and **V1** (5 mM) in solution, in which the signal intensity (Y) is plotted against the time in seconds (X) (^{1}H, 500 MHz, chloroform-*d* : acetonitrile-*d*$_3$, 1:1, v/v, 263 K).

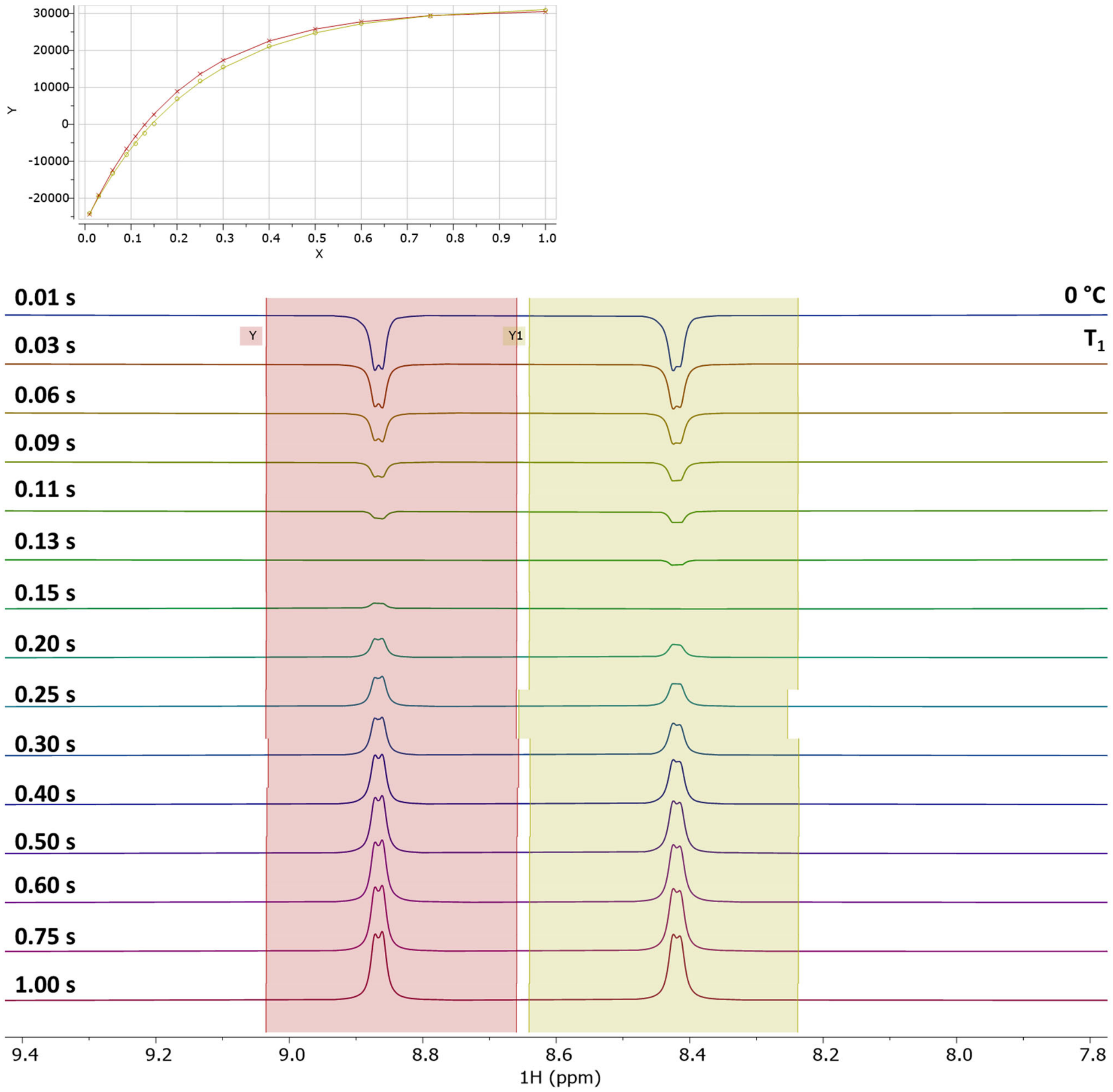

Supplementary Figure 64. T_1 (inverse recovery). Stacked spectra of the inverse recovery experiment to determine the T_1 values of the ortho (Y) and meta (Y1) protons of **Mn1** (0.5 mM) and **V1** (5 mM) in solution, in which the signal intensity (Y) is plotted against the time in seconds (X) (^{1}H, 500 MHz, chloroform-*d* : acetonitrile-*d*$_3$, 1:1, v/v, 273 K).

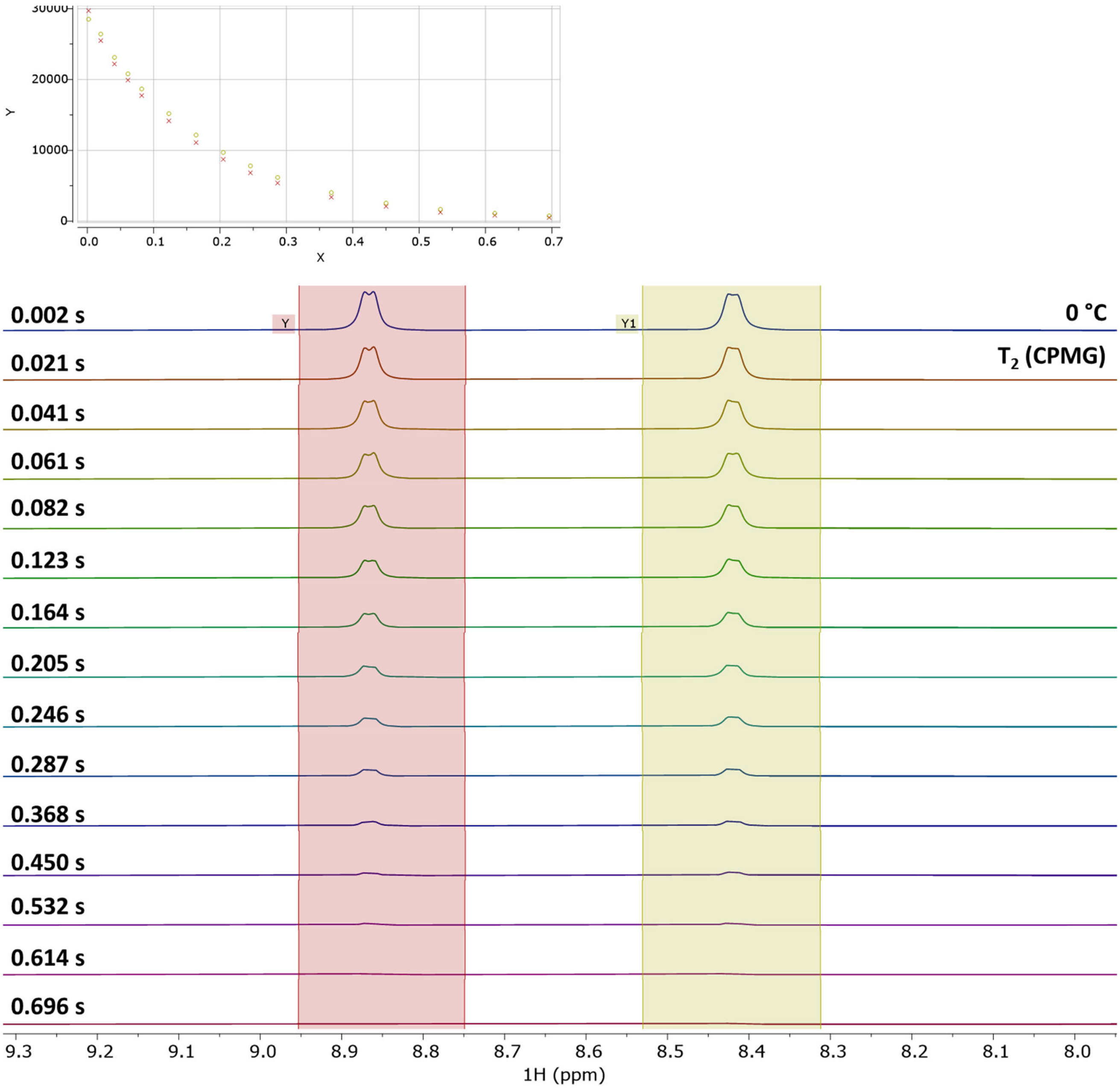

Supplementary Figure 65. T_2 (CPMG). Stacked spectra of the CPMG experiment to determine the T_2 values of the ortho (Y) and meta (Y1) protons of **Mn1** (0.5 mM) and **V1** (5 mM) in solution, in which the signal intensity (Y) is plotted against the time in seconds (X) (^{1}H, 500 MHz, chloroform-*d* : acetonitrile-*d*$_3$, 1:1, v/v, 273 K).

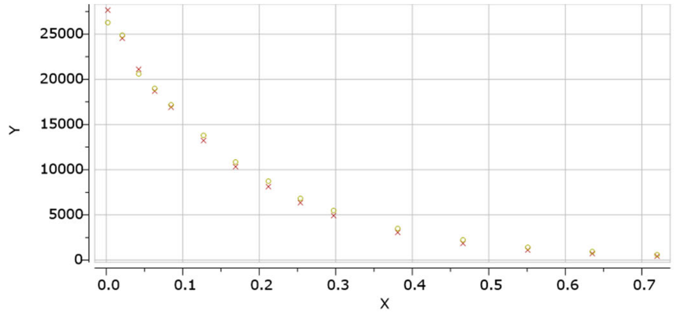

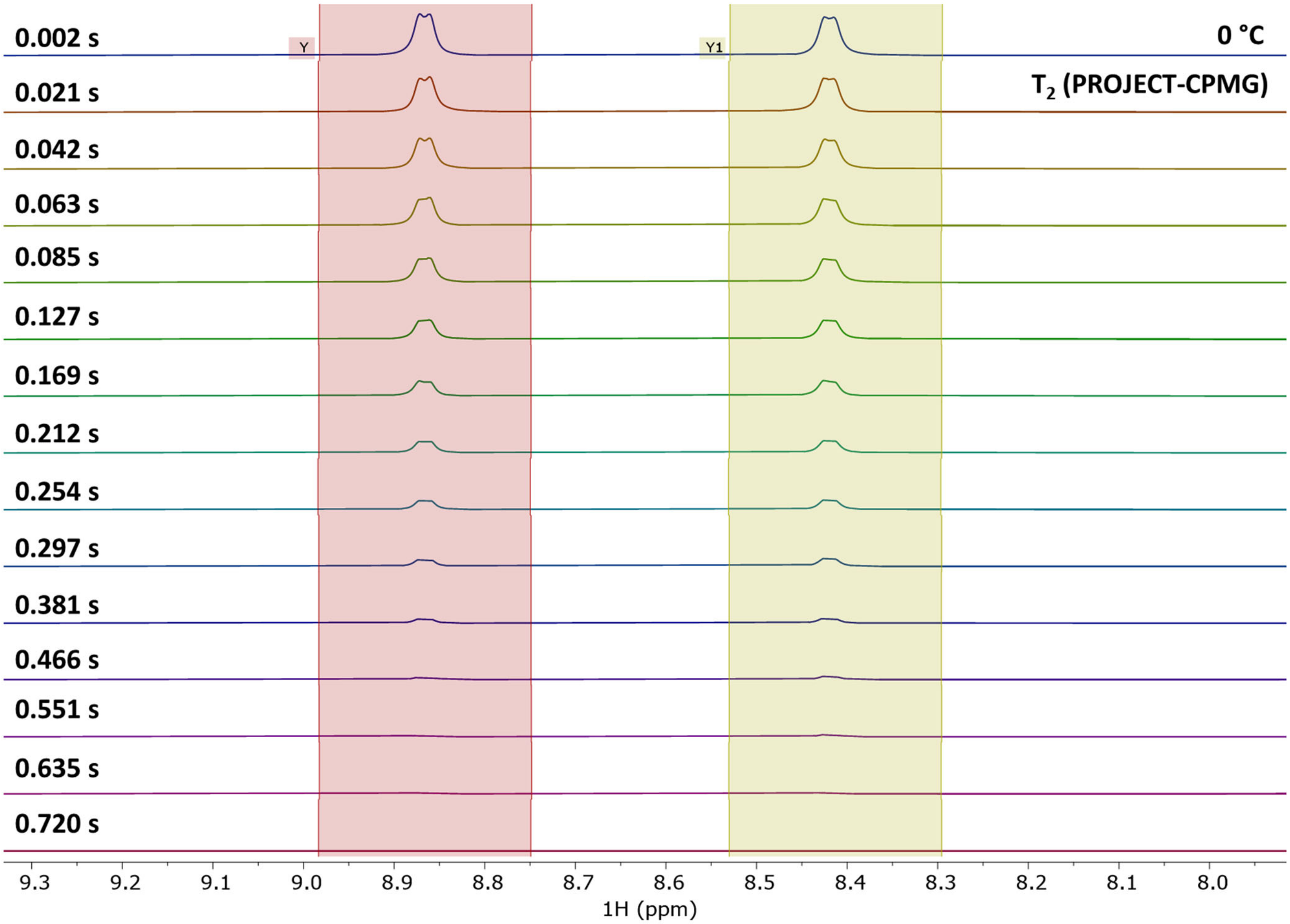

Supplementary Figure 66. T_2 (PROJECT-CPMG). Stacked spectra of the PROJECT-CPMG experiment to determine the T_2 values of the ortho (Y) and meta (Y1) protons of **Mn1** (0.5 mM) and **V1** (5 mM) in solution, in which the signal intensity (Y) is plotted against the time in seconds (X) (^{1}H, 500 MHz, chloroform-*d* : acetonitrile-*d*$_3$, 1:1, v/v, 273 K).

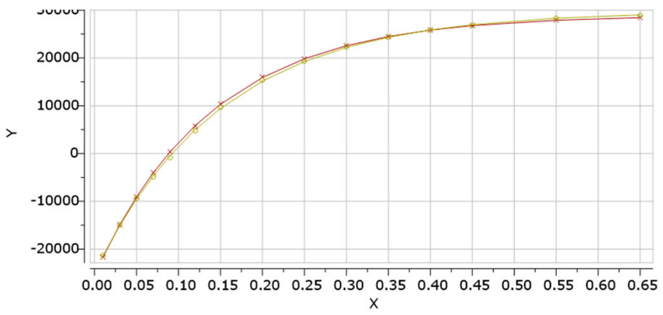

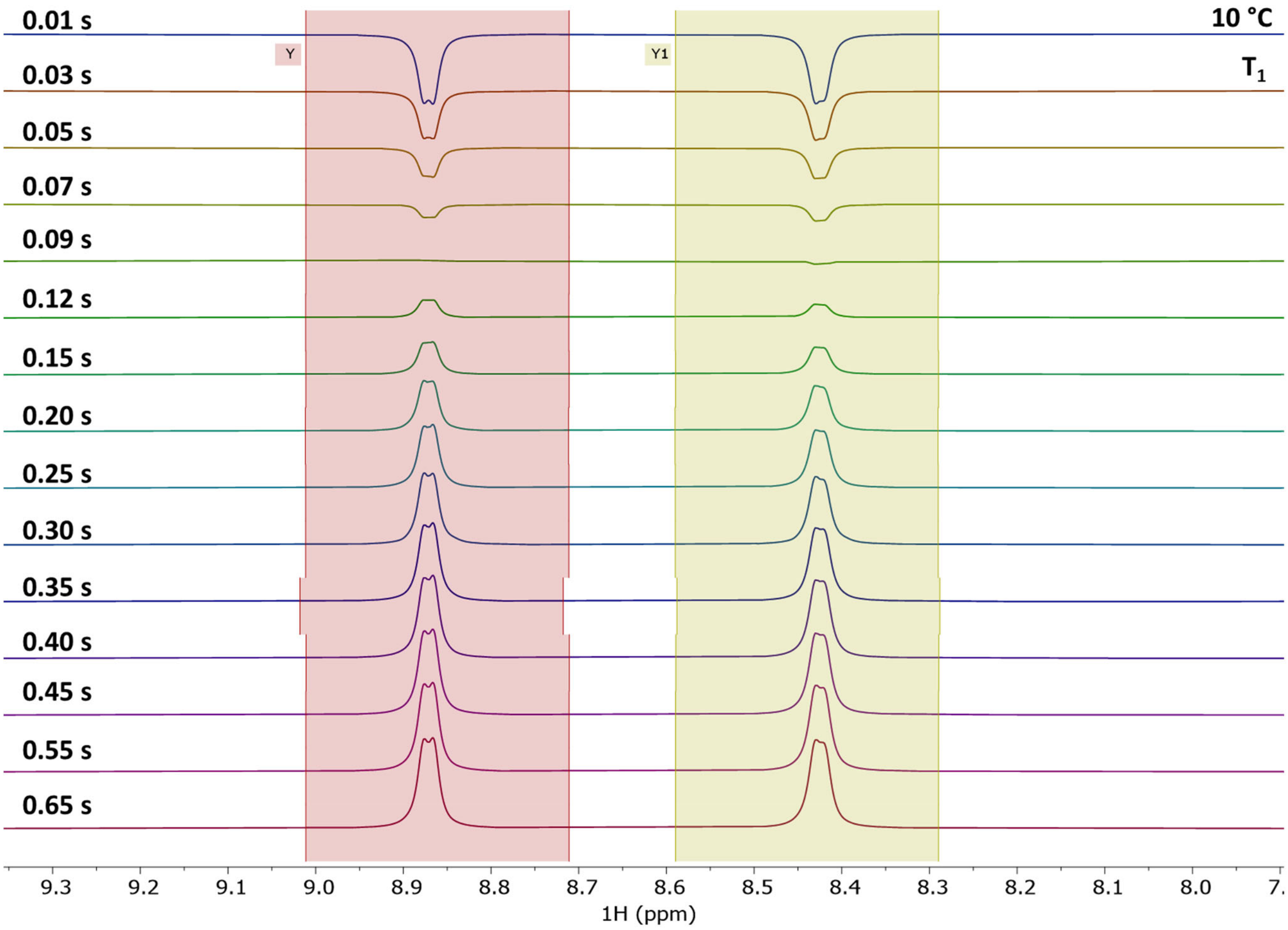

Supplementary Figure 67. T_1 (inverse recovery). Stacked spectra of the inverse recovery experiment to determine the T_1 values of the ortho (Y) and meta (Y1) protons of **Mn1** (0.5 mM) and **V1** (5 mM) in solution, in which the signal intensity (Y) is plotted against the time in seconds (X) (^{1}H, 500 MHz, chloroform-*d* : acetonitrile-*d*$_3$, 1:1, v/v, 283 K).

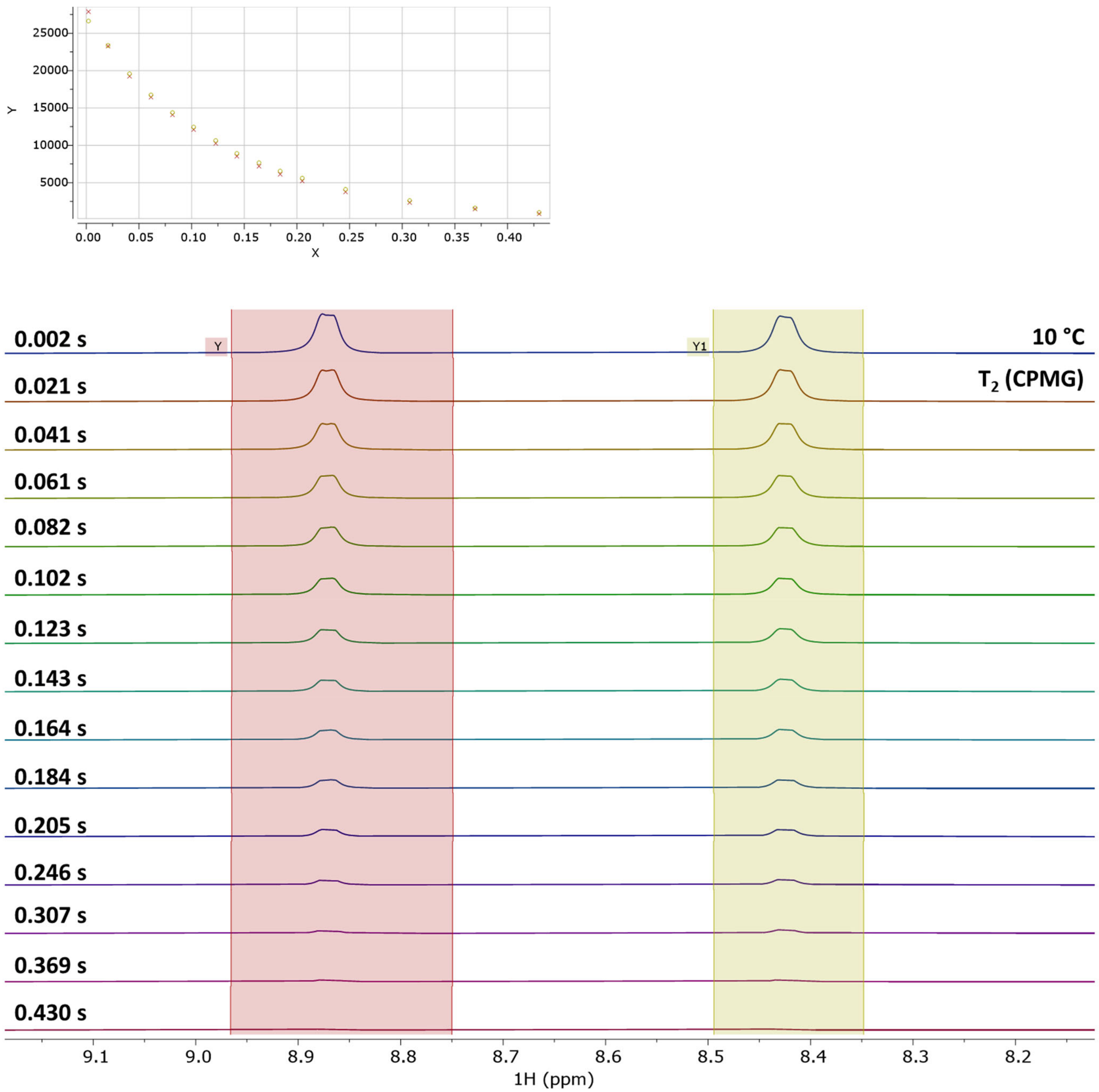

Supplementary Figure 68. T_2 (CPMG). Stacked spectra of the CPMG experiment to determine the T_2 values of the ortho (Y) and meta (Y1) protons of **Mn1** (0.5 mM) and **V1** (5 mM) in solution, in which the signal intensity (Y) is plotted against the time in seconds (X) (^{1}H, 500 MHz, chloroform-*d* : acetonitrile-*d*$_3$, 1:1, v/v, 283 K).

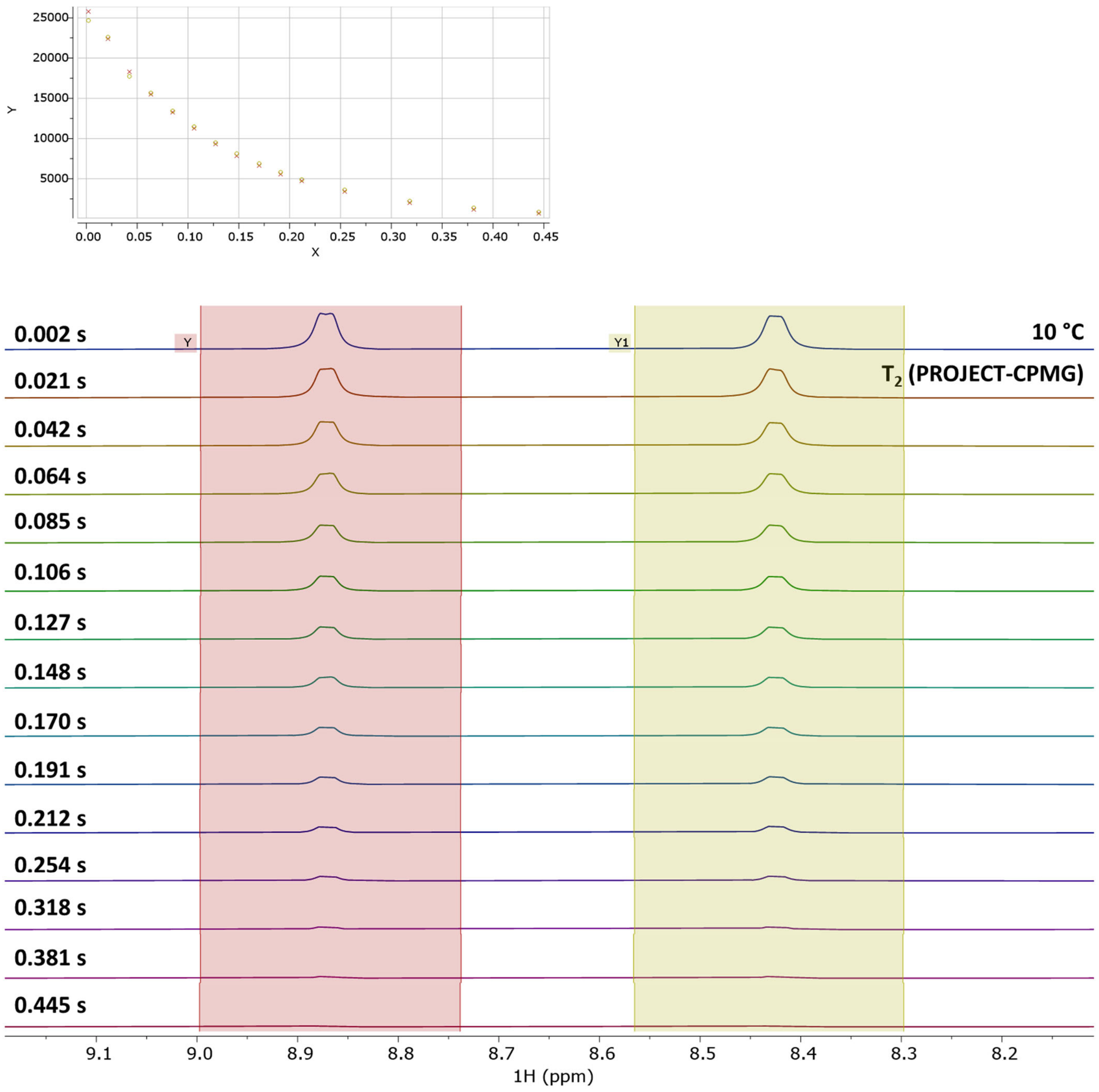

Supplementary Figure 69. T_2 (PROJECT-CPMG). Stacked spectra of the PROJECT-CPMG experiment to determine the T_2 values of the ortho (Y) and meta (Y1) protons of **Mn1** (0.5 mM) and **V1** (5 mM) in solution, in which the signal intensity (Y) is plotted against the time in seconds (X) (^{1}H, 500 MHz, chloroform-*d* : acetonitrile-d_3, 1:1, v/v, 283 K).

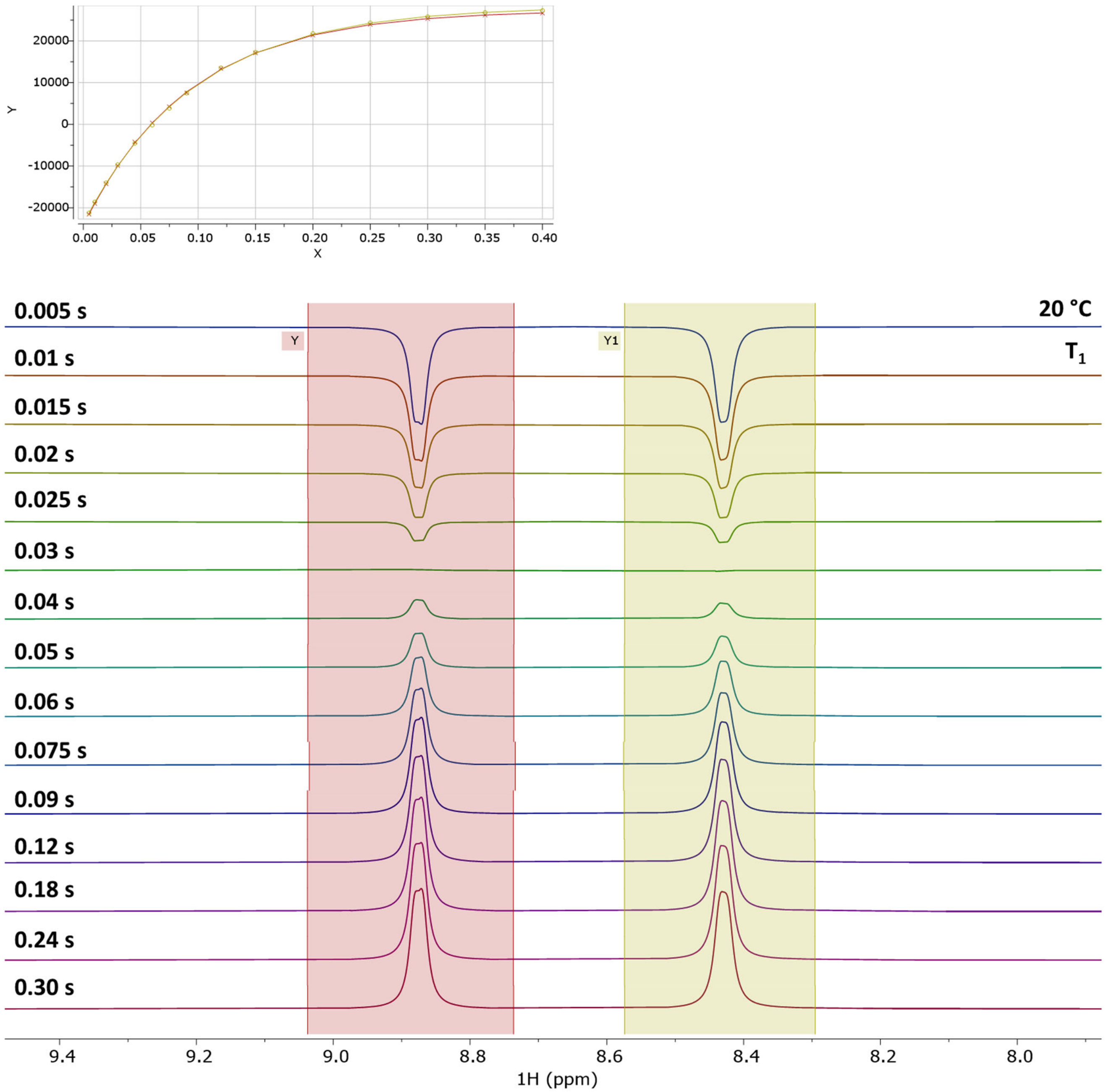

Supplementary Figure 70. T_1 (inverse recovery). Stacked spectra of the inverse recovery experiment to determine the T_1 values of the ortho (Y) and meta (Y1) protons of **Mn1** (0.5 mM) and **V1** (5 mM) in solution, in which the signal intensity (Y) is plotted against the time in seconds (X) (^{1}H, 500 MHz, chloroform-*d* : acetonitrile-*d*$_3$, 1:1, v/v, 293 K).

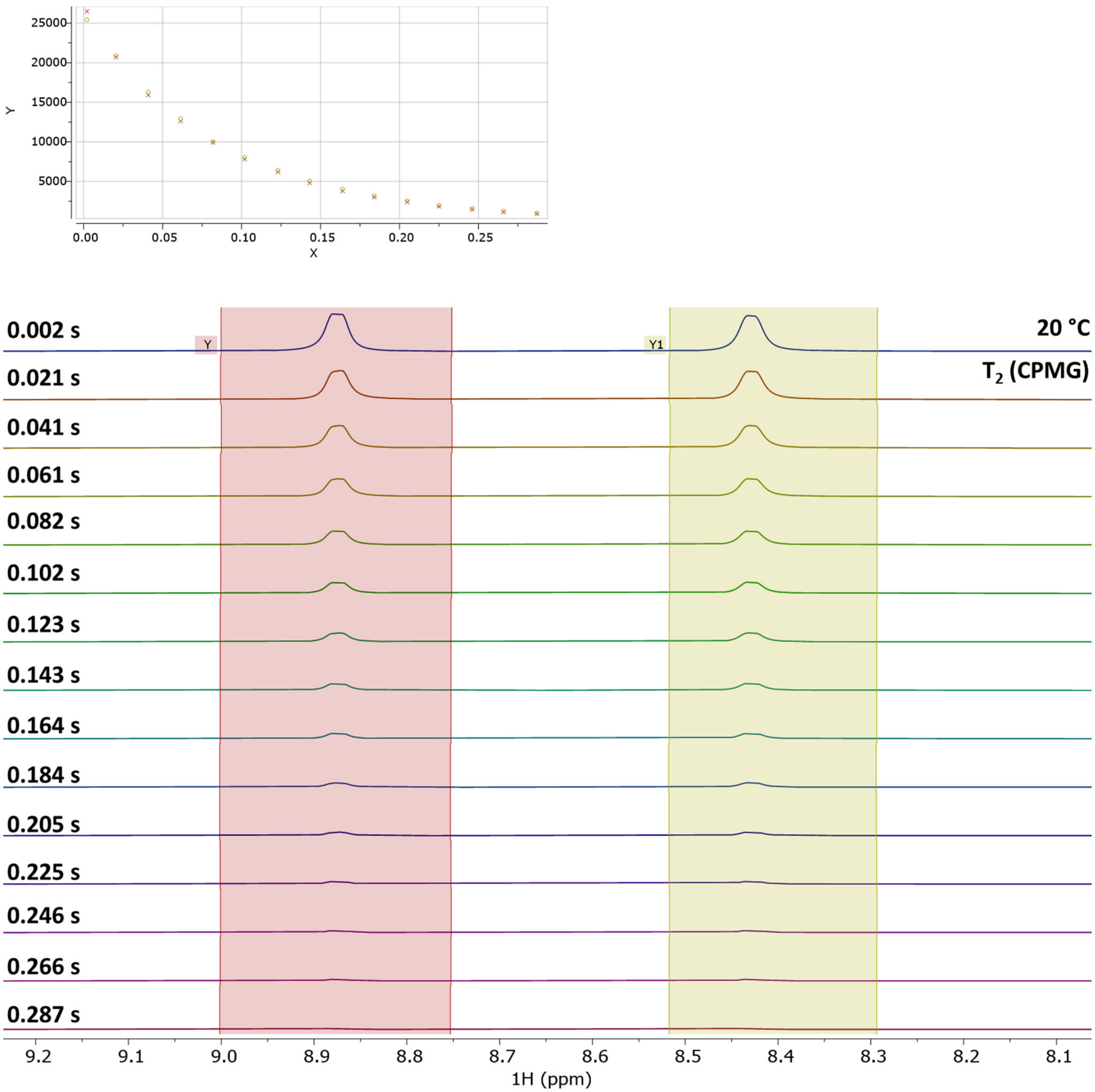

Supplementary Figure 71. T_2 (CPMG). Stacked spectra of the CPMG experiment to determine the T_2 values of the ortho (Y) and meta (Y1) protons of **Mn1** (0.5 mM) and **V1** (5 mM) in solution, in which the signal intensity (Y) is plotted against the time in seconds (X) (^{1}H, 500 MHz, chloroform-*d* : acetonitrile-*d*$_3$, 1:1, v/v, 293 K).

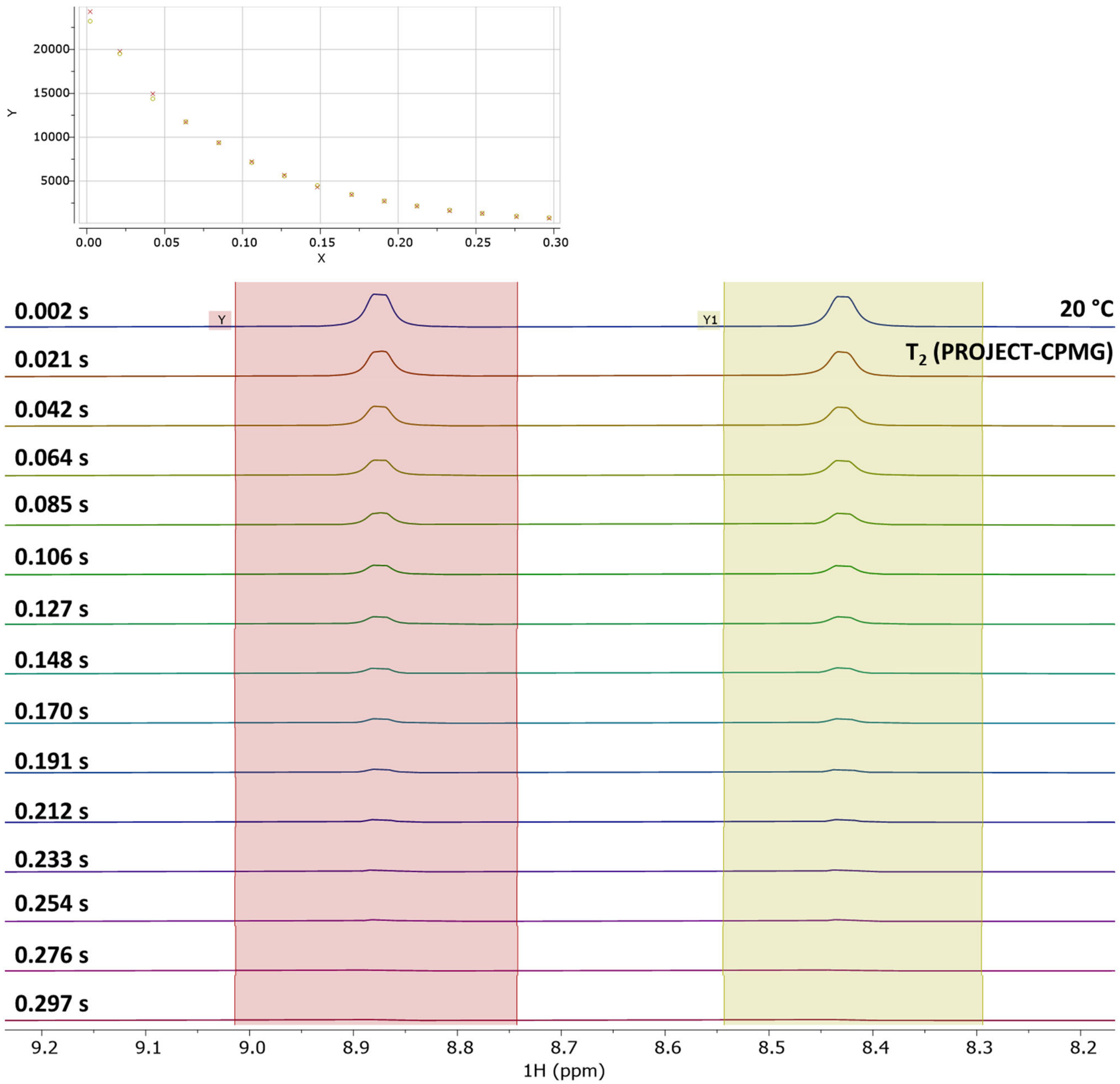

Supplementary Figure 72. T_2 (PROJECT-CPMG). Stacked spectra of the PROJECT-CPMG experiment to determine the T_2 values of the ortho (Y) and meta (Y1) protons of **Mn1** (0.5 mM) and **V1** (5 mM) in solution, in which the signal intensity (Y) is plotted against the time in seconds (X) (^{1}H, 500 MHz, chloroform-*d* : acetonitrile-d_3, 1:1, v/v, 293 K).

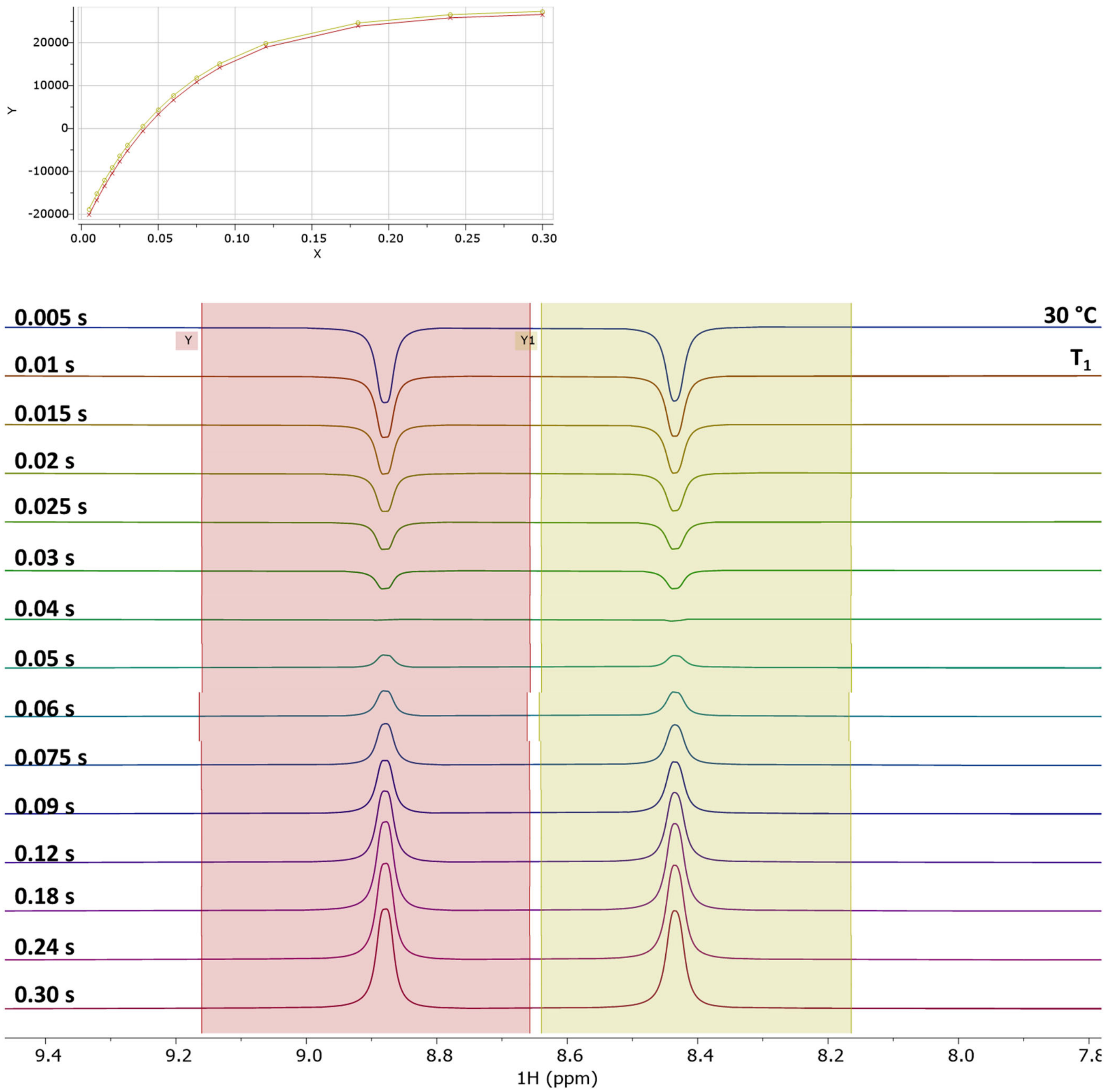

Supplementary Figure 73. T_1 (inverse recovery). Stacked spectra of the inverse recovery experiment to determine the T_1 values of the ortho (Y) and meta (Y1) protons of **Mn1** (0.5 mM) and **V1** (5 mM) in solution, in which the signal intensity (Y) is plotted against the time in seconds (X) (^{1}H, 500 MHz, chloroform-*d* : acetonitrile-*d*$_3$, 1:1, v/v, 303 K).

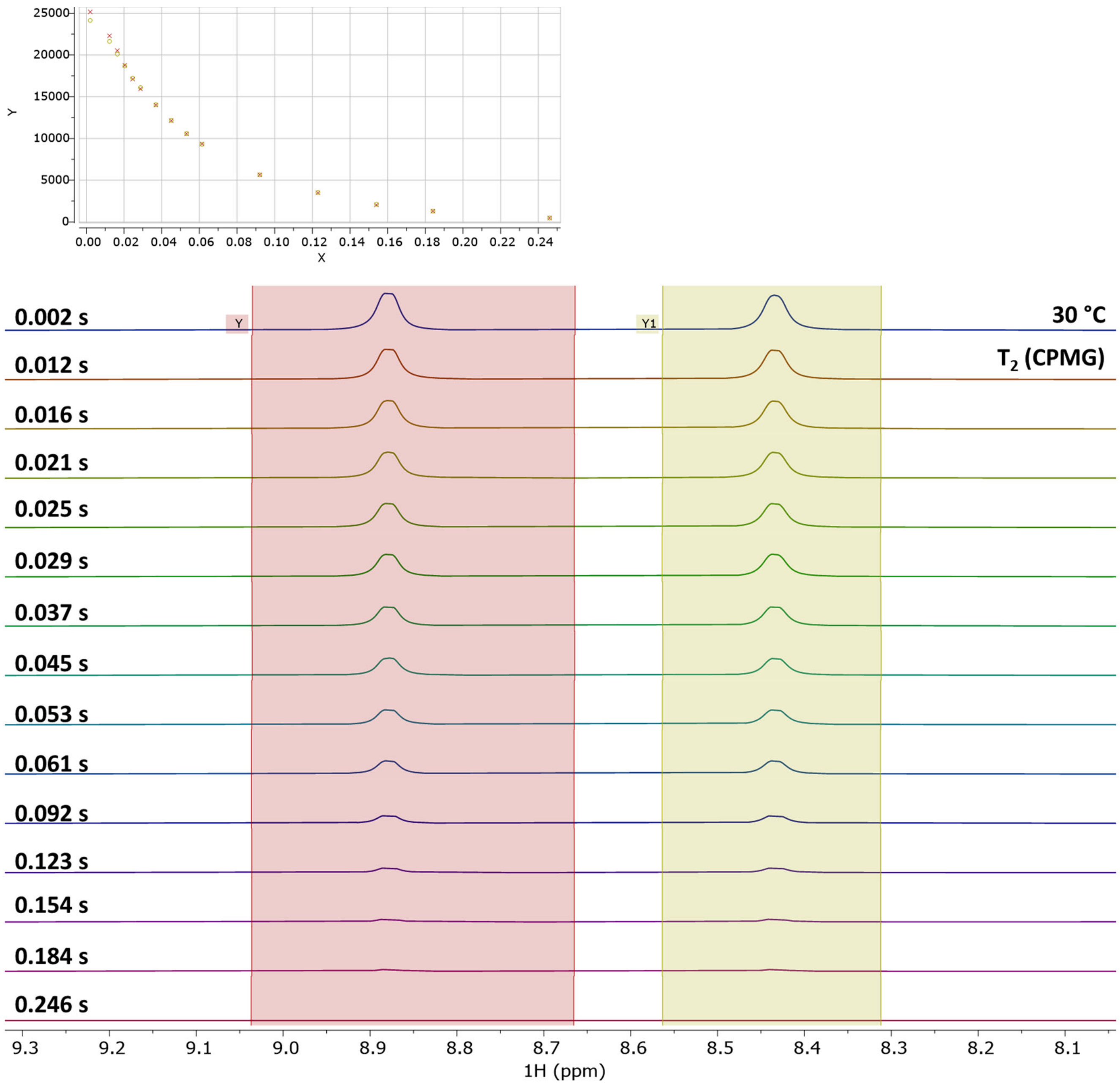

Supplementary Figure 74. T_2 (CPMG). Stacked spectra of the CPMG experiment to determine the T_2 values of the ortho (Y) and meta (Y1) protons of **Mn1** (0.5 mM) and **V1** (5 mM) in solution, in which the signal intensity (Y) is plotted against the time in seconds (X) (^{1}H, 500 MHz, chloroform-*d* : acetonitrile-*d$_3$*, 1:1, v/v, 303 K).

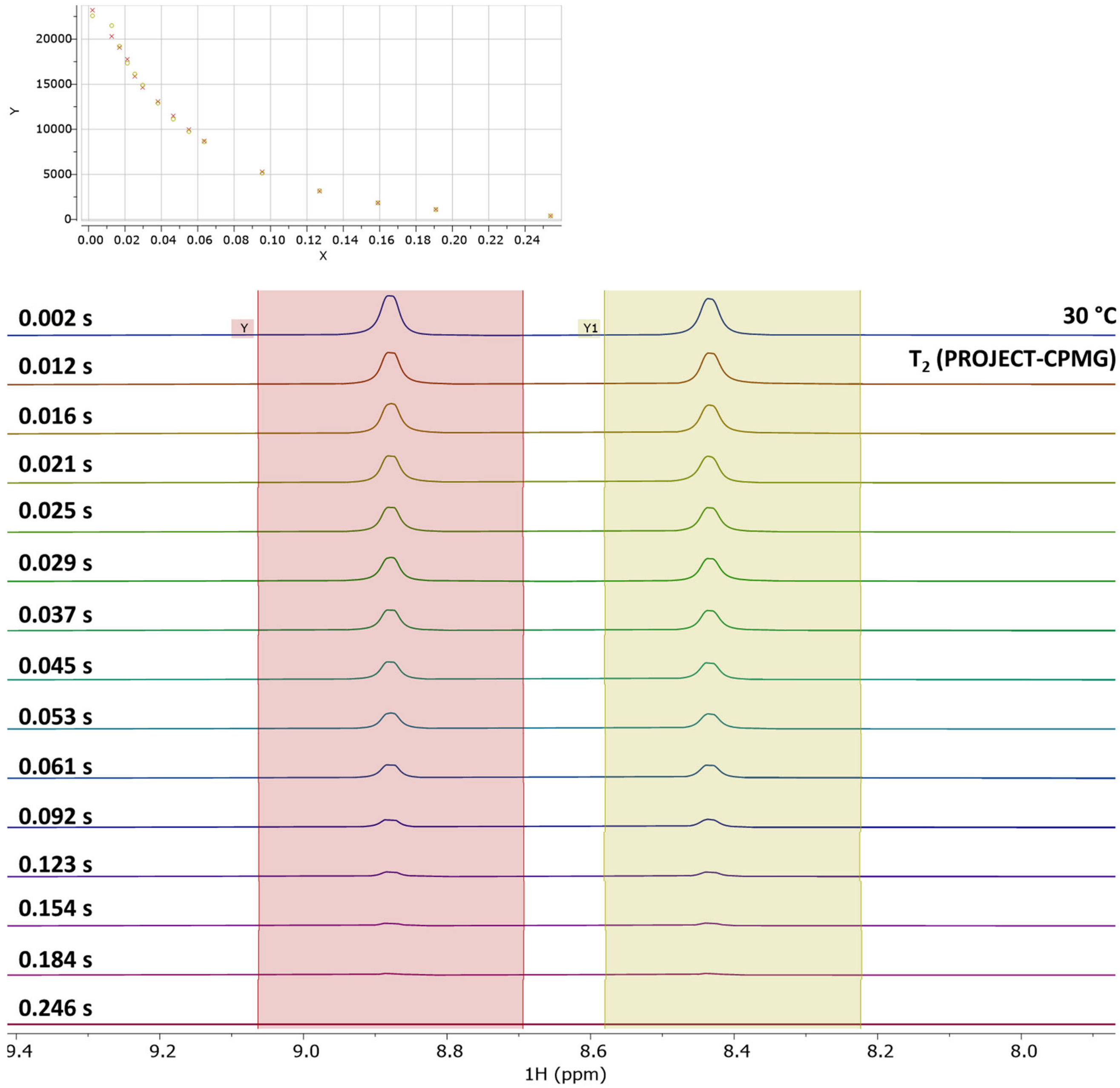

Supplementary Figure 75. T_2 (PROJECT-CPMG). Stacked spectra of the PROJECT-CPMG experiment to determine the T_2 values of the ortho (Y) and meta (Y1) protons of **Mn1** (0.5 mM) and **V1** (5 mM) in solution, in which the signal intensity (Y) is plotted against the time in seconds (X) (^{1}H, 500 MHz, chloroform-*d* : acetonitrile-d_3, 1:1, v/v, 303 K).

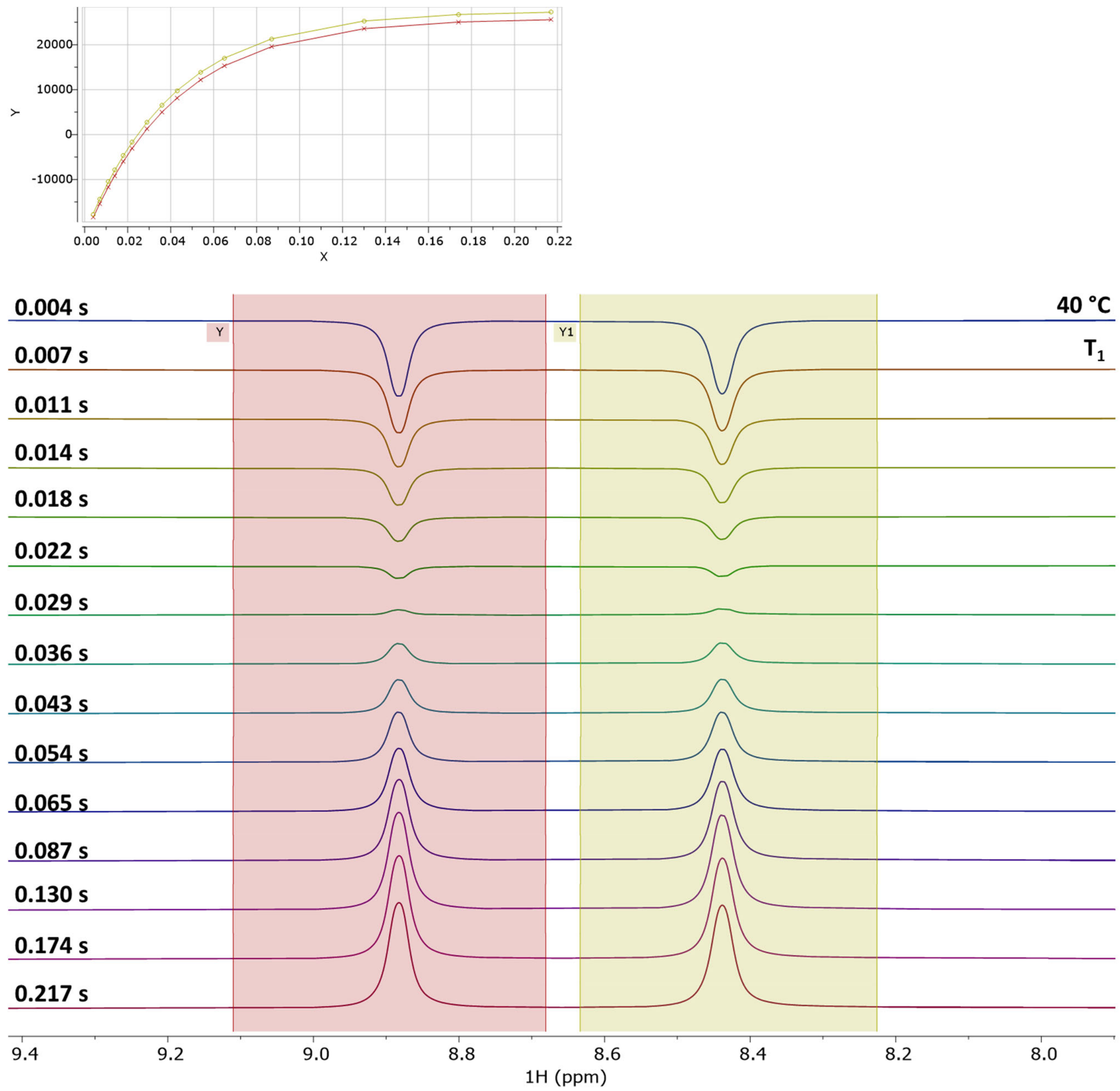

Supplementary Figure 76. T_1 (inverse recovery). Stacked spectra of the inverse recovery experiment to determine the T_1 values of the ortho (Y) and meta (Y1) protons of **Mn1** (0.5 mM) and **V1** (5 mM) in solution, in which the signal intensity (Y) is plotted against the time in seconds (X) (^{1}H, 500 MHz, chloroform-*d* : acetonitrile-*d*$_3$, 1:1, v/v, 313 K).

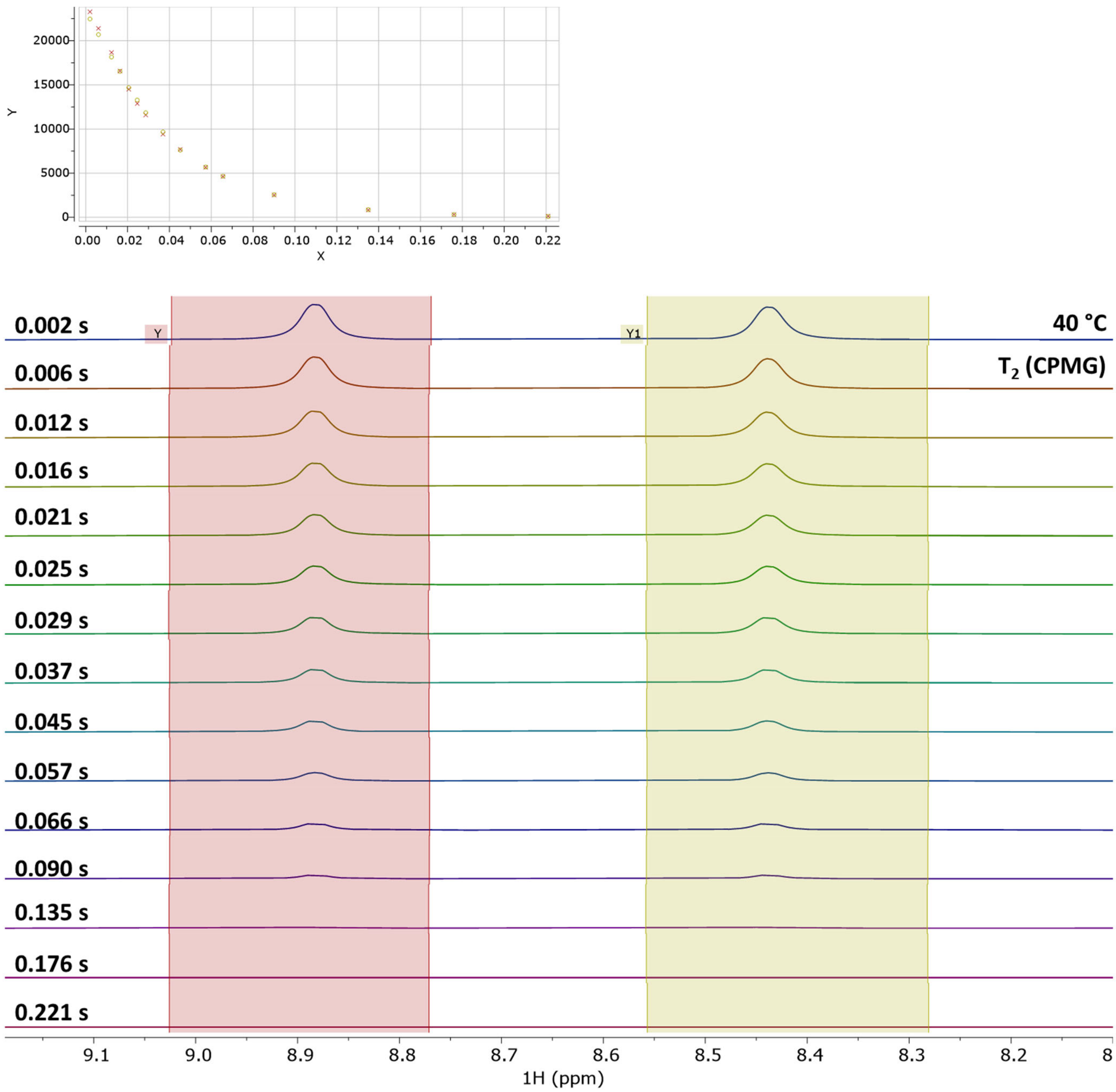

Supplementary Figure 77. T_2 (CPMG). Stacked spectra of the CPMG experiment to determine the T_2 values of the ortho (Y) and meta (Y1) protons of **Mn1** (0.5 mM) and **V1** (5 mM) in solution, in which the signal intensity (Y) is plotted against the time in seconds (X) (^{1}H, 500 MHz, chloroform-*d* : acetonitrile-*d*$_3$, 1:1, v/v, 313 K).

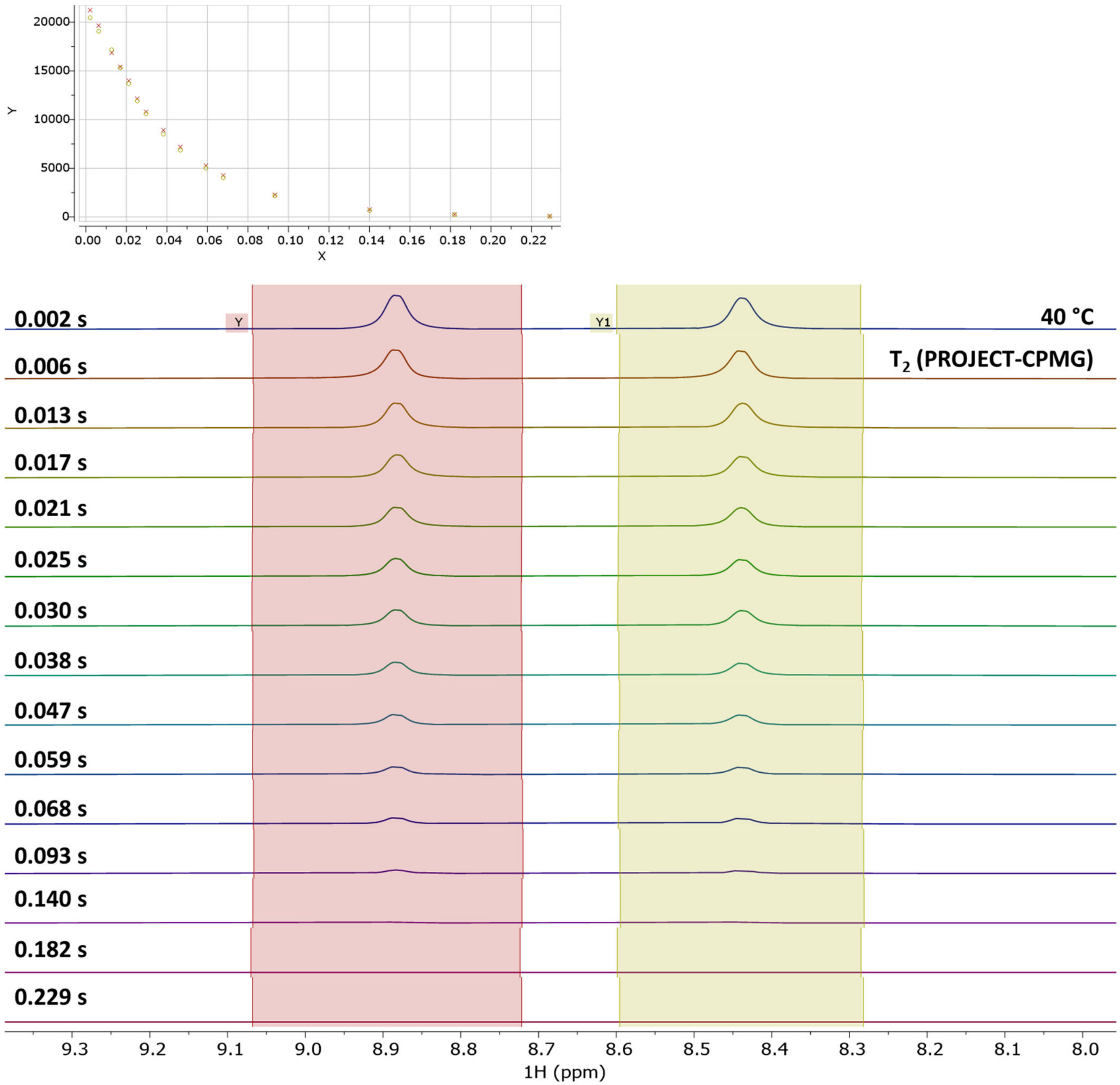

Supplementary Figure 78. T_2 (PROJECT-CPMG). Stacked spectra of the PROJECT-CPMG experiment to determine the T_2 values of the ortho (Y) and meta (Y1) protons of **Mn1** (0.5 mM) and **V1** (5 mM) in solution, in which the signal intensity (Y) is plotted against the time in seconds (X) (^{1}H, 500 MHz, chloroform-*d* : acetonitrile-*d*$_3$, 1:1, v/v, 313 K).

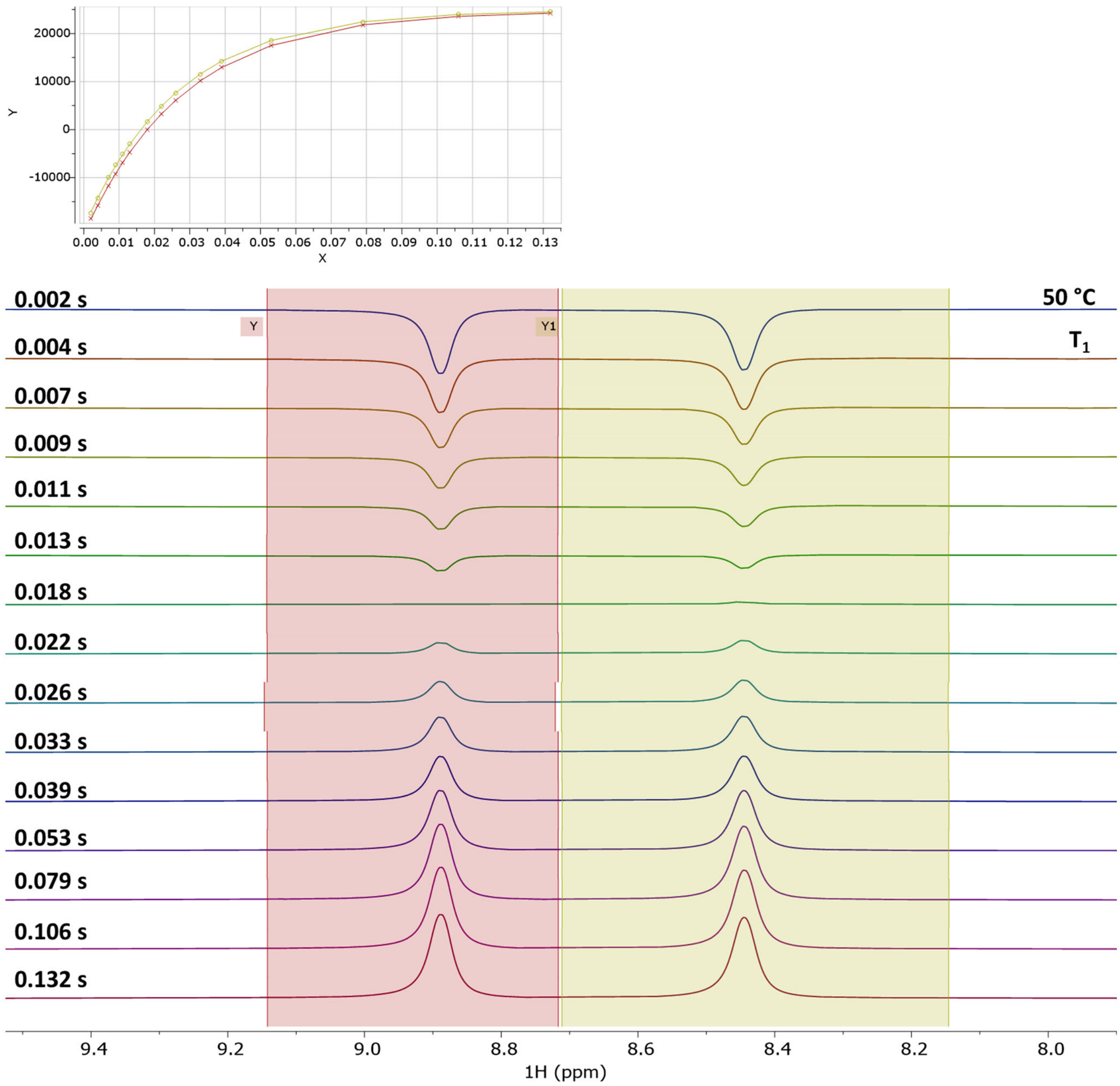

Supplementary Figure 79. T_1 (inverse recovery). Stacked spectra of the inverse recovery experiment to determine the T_1 values of the ortho (Y) and meta (Y1) protons of **Mn1** (0.5 mM) and **V1** (5 mM) in solution, in which the signal intensity (Y) is plotted against the time in seconds (X) (^{1}H, 500 MHz, chloroform-*d* : acetonitrile-*d*$_3$, 1:1, v/v, 323 K).

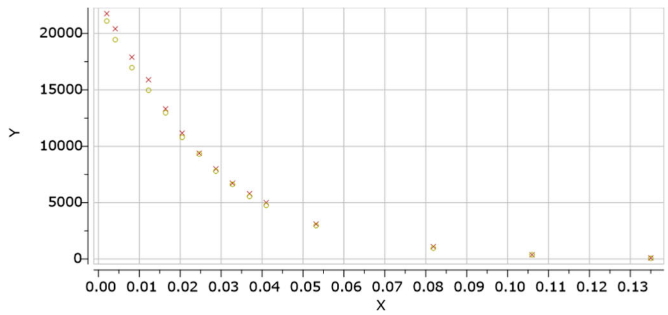

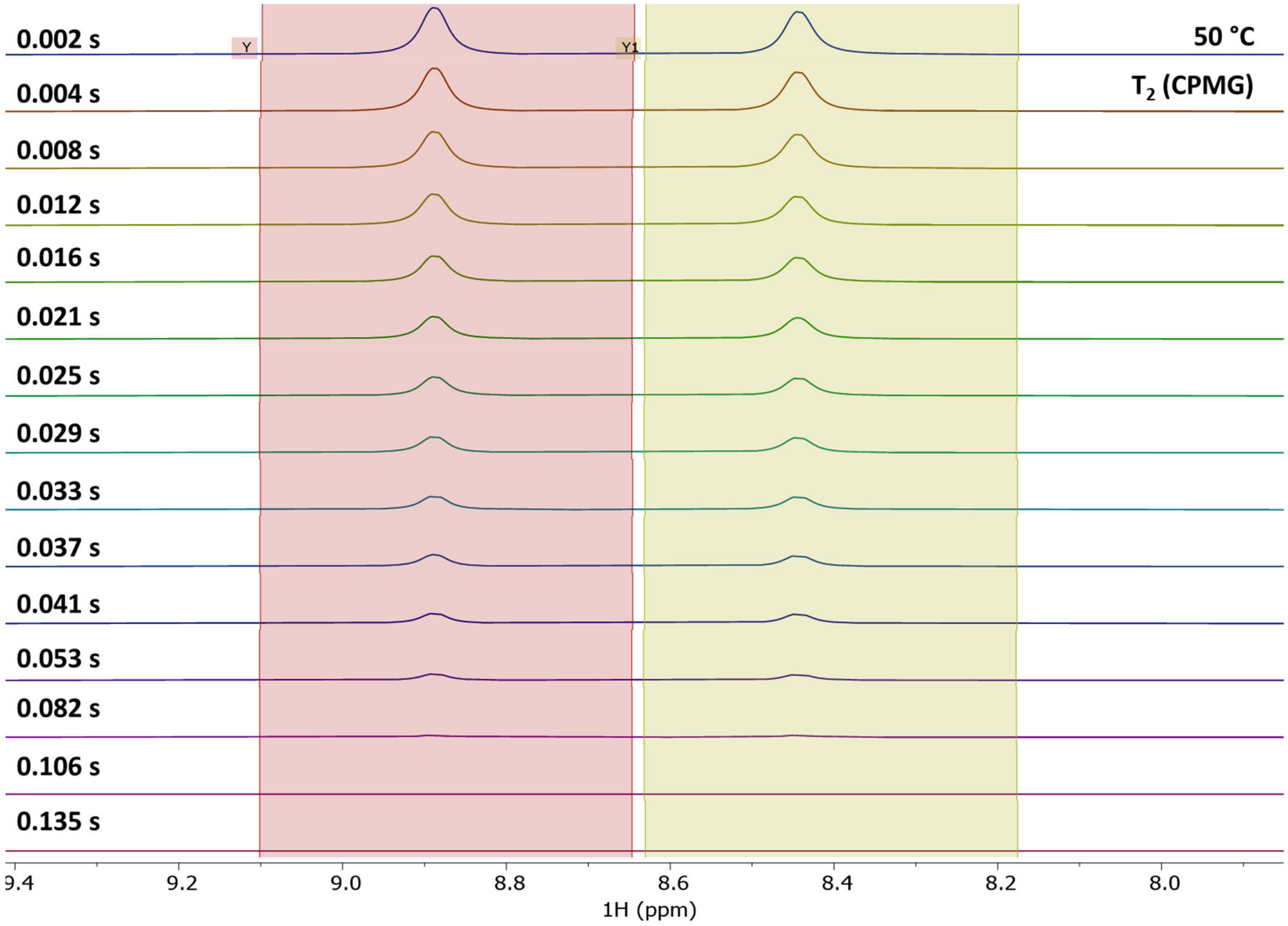

Supplementary Figure 80. T_2 (CPMG). Stacked spectra of the CPMG experiment to determine the T_2 values of the ortho (Y) and meta (Y1) protons of **Mn1** (0.5 mM) and **V1** (5 mM) in solution, in which the signal intensity (Y) is plotted against the time in seconds (X) (^{1}H, 500 MHz, chloroform-*d* : acetonitrile-*d*$_3$, 1:1, v/v, 323 K).

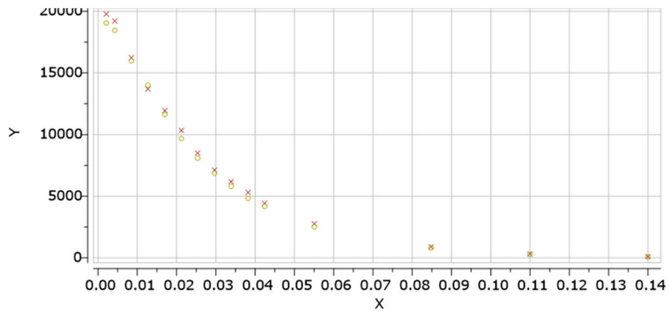

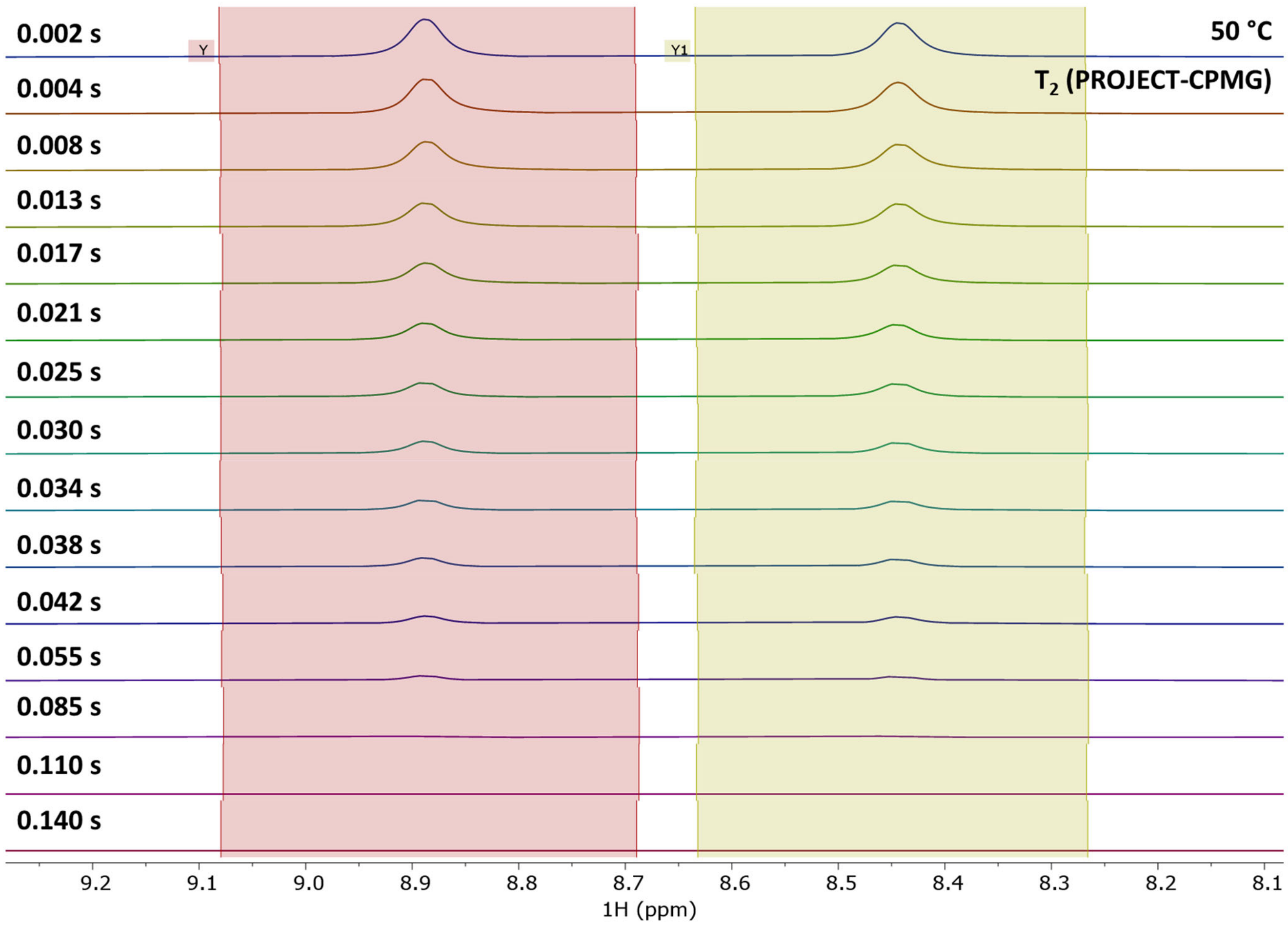

Supplementary Figure 81. T_2 (PROJECT-CPMG). Stacked spectra of the PROJECT-CPMG experiment to determine the T_2 values of the ortho (Y) and meta (Y1) protons of **Mn1** (0.5 mM) and **V1** (5 mM) in solution, in which the signal intensity (Y) is plotted against the time in seconds (X) (^{1}H, 500 MHz, chloroform-*d* : acetonitrile-*d*$_3$, 1:1, v/v, 323 K).

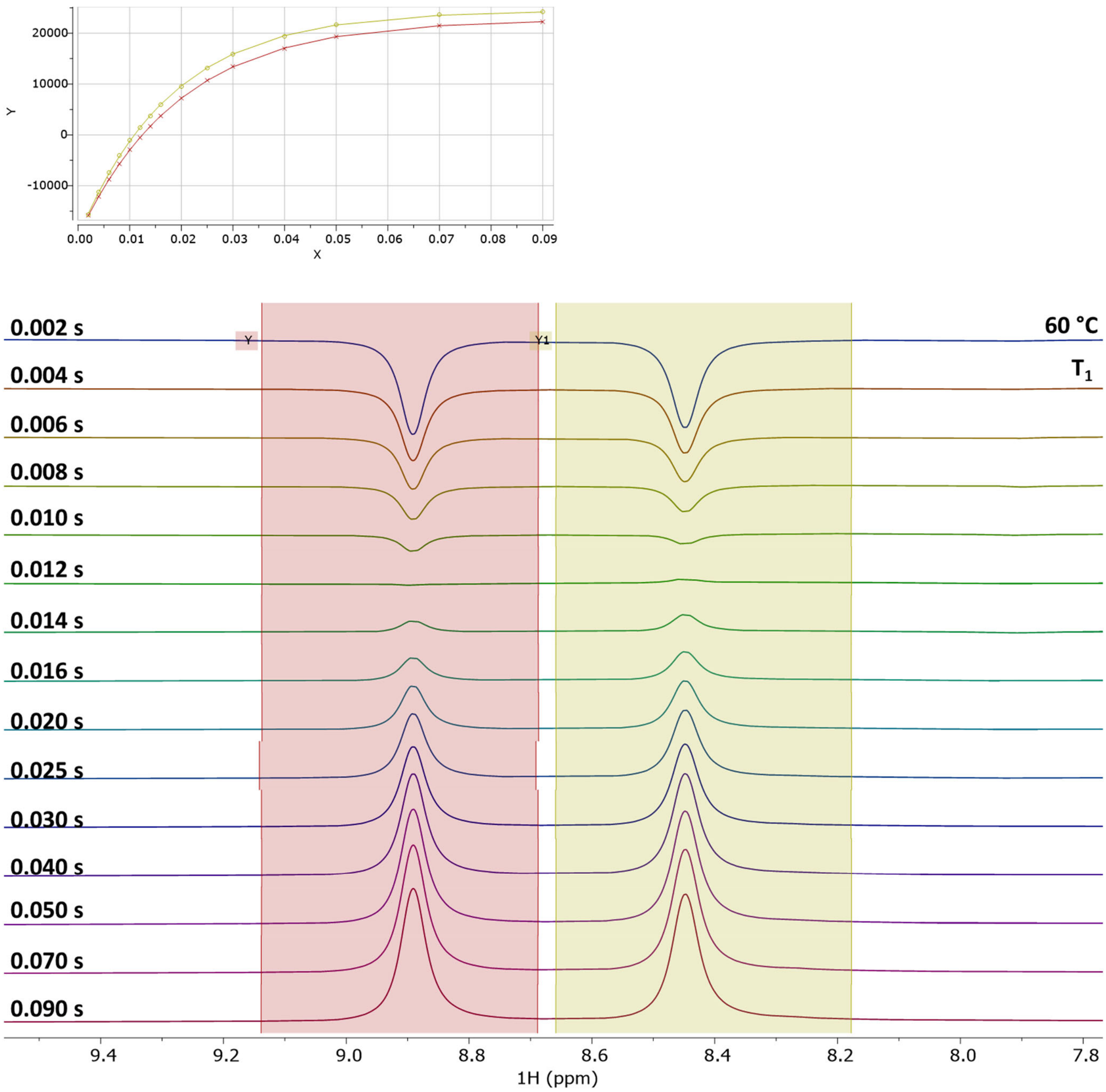

Supplementary Figure 82. T_1 (inverse recovery). Stacked spectra of the inverse recovery experiment to determine the T_1 values of the ortho (Y) and meta (Y1) protons of **Mn1** (0.5 mM) and **V1** (5 mM) in solution, in which the signal intensity (Y) is plotted against the time in seconds (X) (^{1}H, 500 MHz, chloroform-*d* : acetonitrile-*d*$_3$, 1:1, v/v, 333 K).

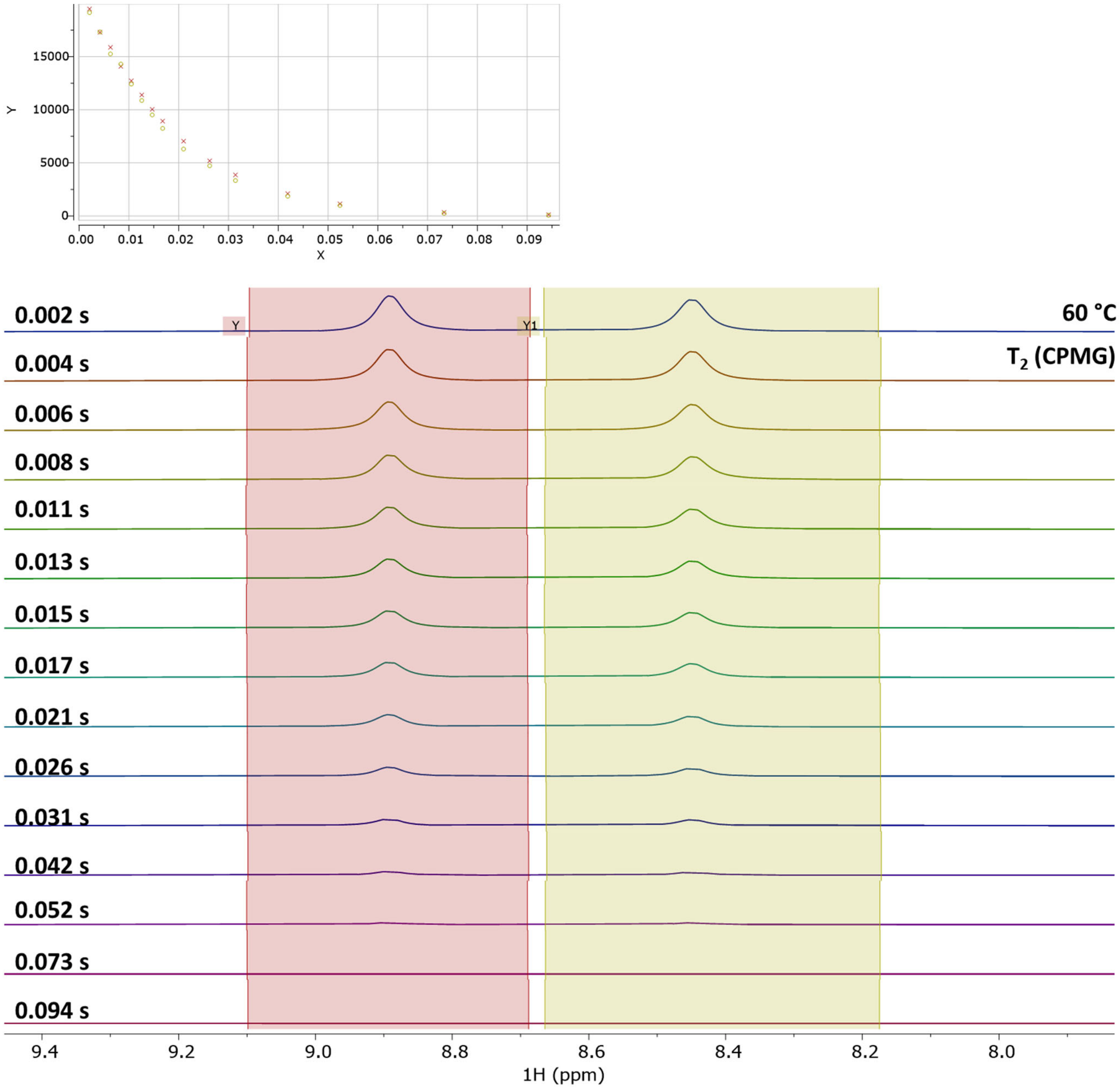

Supplementary Figure 83. T_2 (CPMG). Stacked spectra of the CPMG experiment to determine the T_2 values of the ortho (Y) and meta (Y1) protons of **Mn1** (0.5 mM) and **V1** (5 mM) in solution, in which the signal intensity (Y) is plotted against the time in seconds (X) (^{1}H, 500 MHz, chloroform-*d* : acetonitrile-*d*$_3$, 1:1, v/v, 333 K).

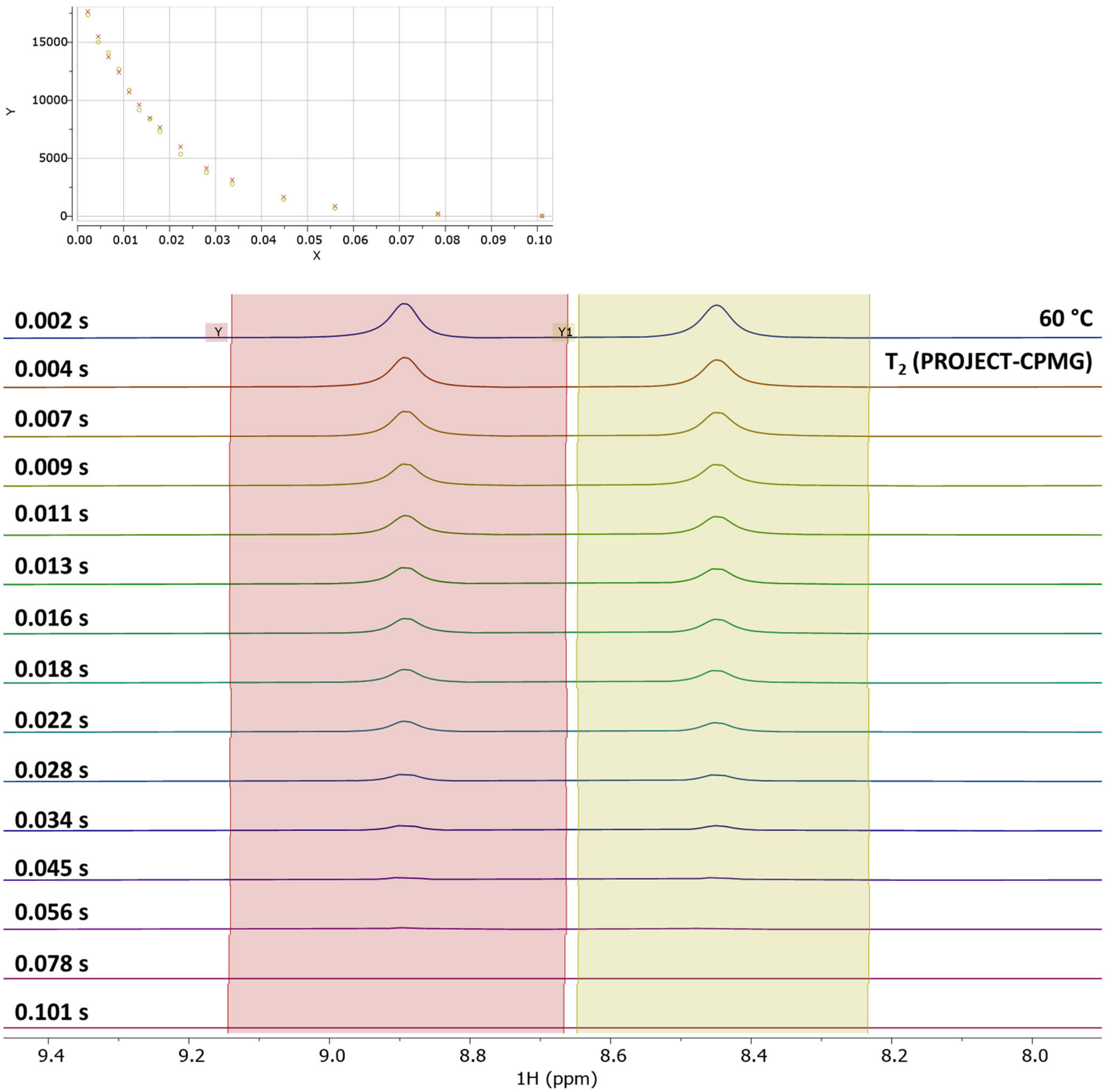

Supplementary Figure 84. T_2 (PROJECT-CPMG). Stacked spectra of the PROJECT-CPMG experiment to determine the T_2 values of the ortho (Y) and meta (Y1) protons of **Mn1** (0.5 mM) and **V1** (5 mM) in solution, in which the signal intensity (Y) is plotted against the time in seconds (X) (^{1}H, 500 MHz, chloroform-*d* : acetonitrile-d_3, 1:1, v/v, 333 K).

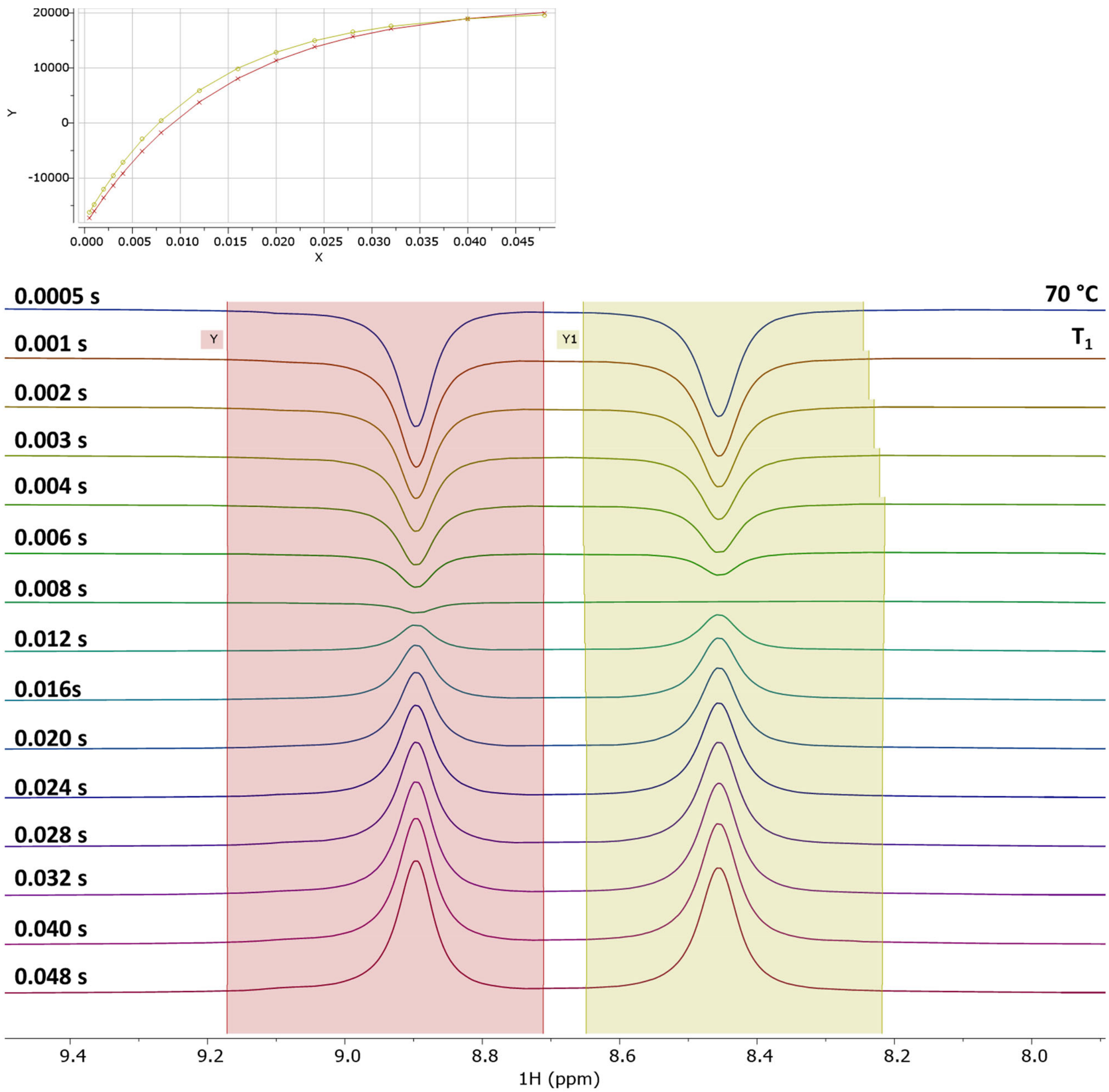

Supplementary Figure 85. T_1 (inverse recovery). Stacked spectra of the inverse recovery experiment to determine the T_1 values of the ortho (Y) and meta (Y1) protons of **Mn1** (0.5 mM) and **V1** (5 mM) in solution, in which the signal intensity (Y) is plotted against the time in seconds (X) (^{1}H, 500 MHz, chloroform-*d* : acetonitrile-*d*$_3$, 1:1, v/v, 343 K).

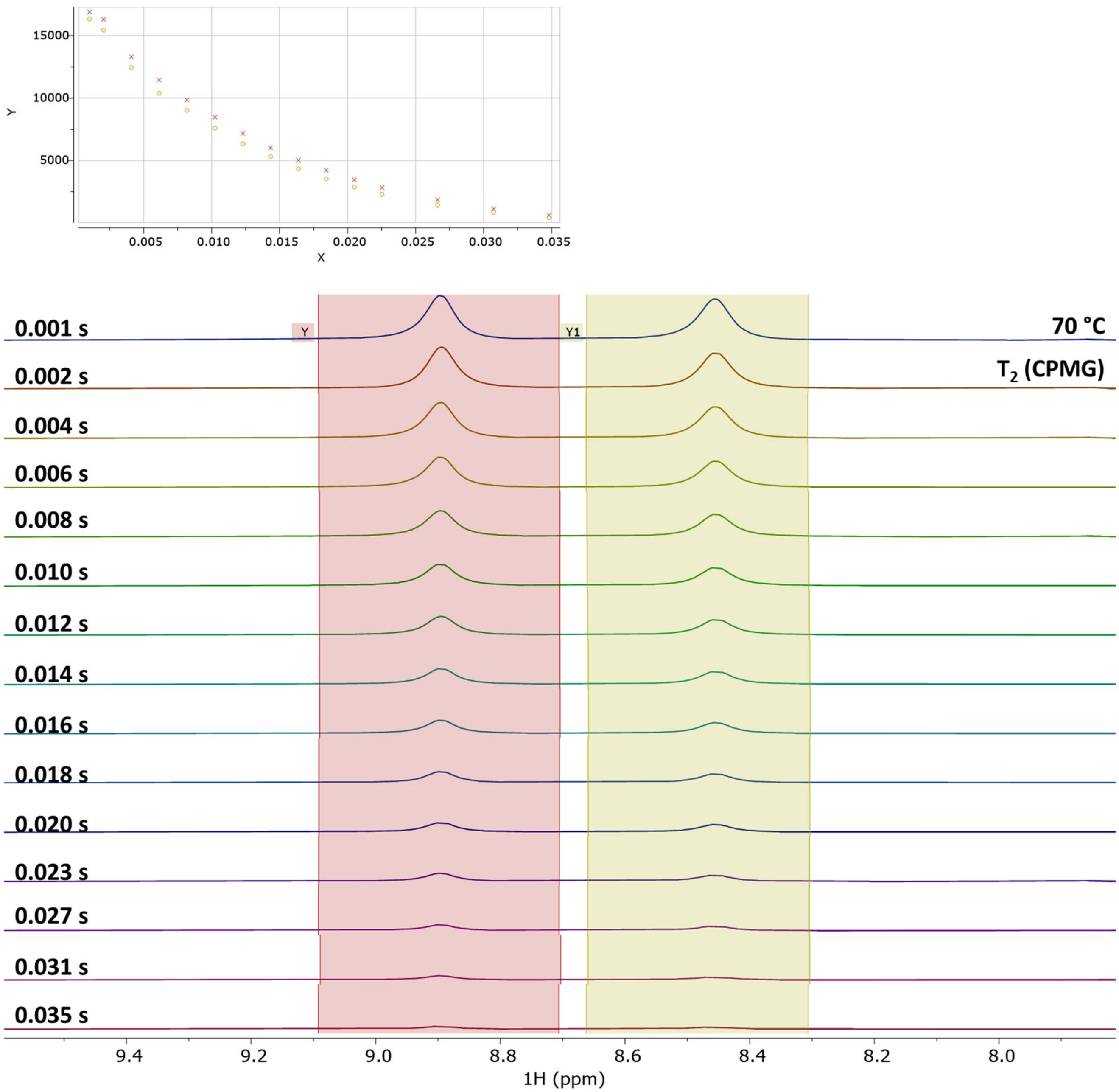

Supplementary Figure 86. T_2 (CPMG). Stacked spectra of the CPMG experiment to determine the T_2 values of the ortho (Y) and meta (Y1) protons of **Mn1** (0.5 mM) and **V1** (5 mM) in solution, in which the signal intensity (Y) is plotted against the time in seconds (X) (^{1}H, 500 MHz, chloroform-*d* : acetonitrile-*d*$_3$, 1:1, v/v, 343 K).

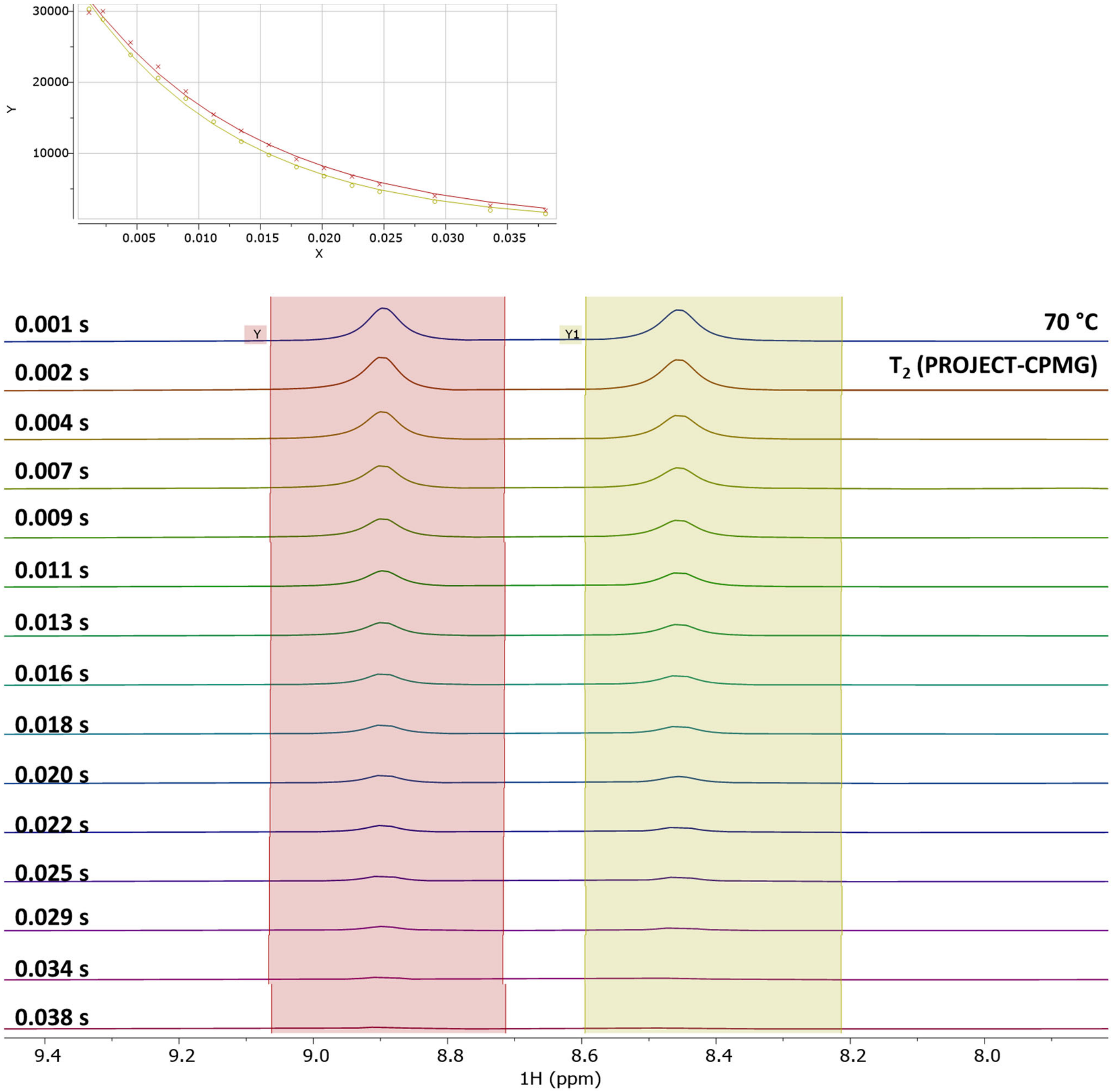

Supplementary Figure 87. T_2 (PROJECT-CPMG). Stacked spectra of the PROJECT-CPMG experiment to determine the T_2 values of the ortho (Y) and meta (Y1) protons of **Mn1** (0.5 mM) and **V1** (5 mM) in solution, in which the signal intensity (Y) is plotted against the time in seconds (X) (^{1}H, 500 MHz, chloroform-*d* : acetonitrile-*d*$_3$, 1:1, v/v, 343 K).

4.3. MnRot/V1 ($T_{1,os}$ and $T_{2,os}$)

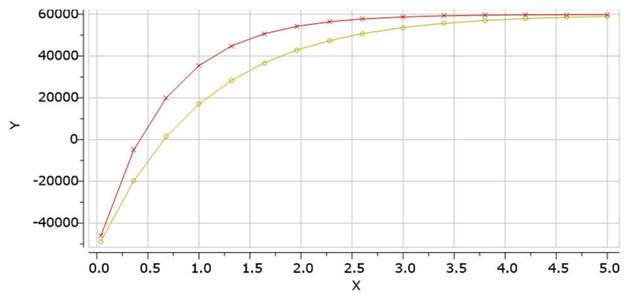

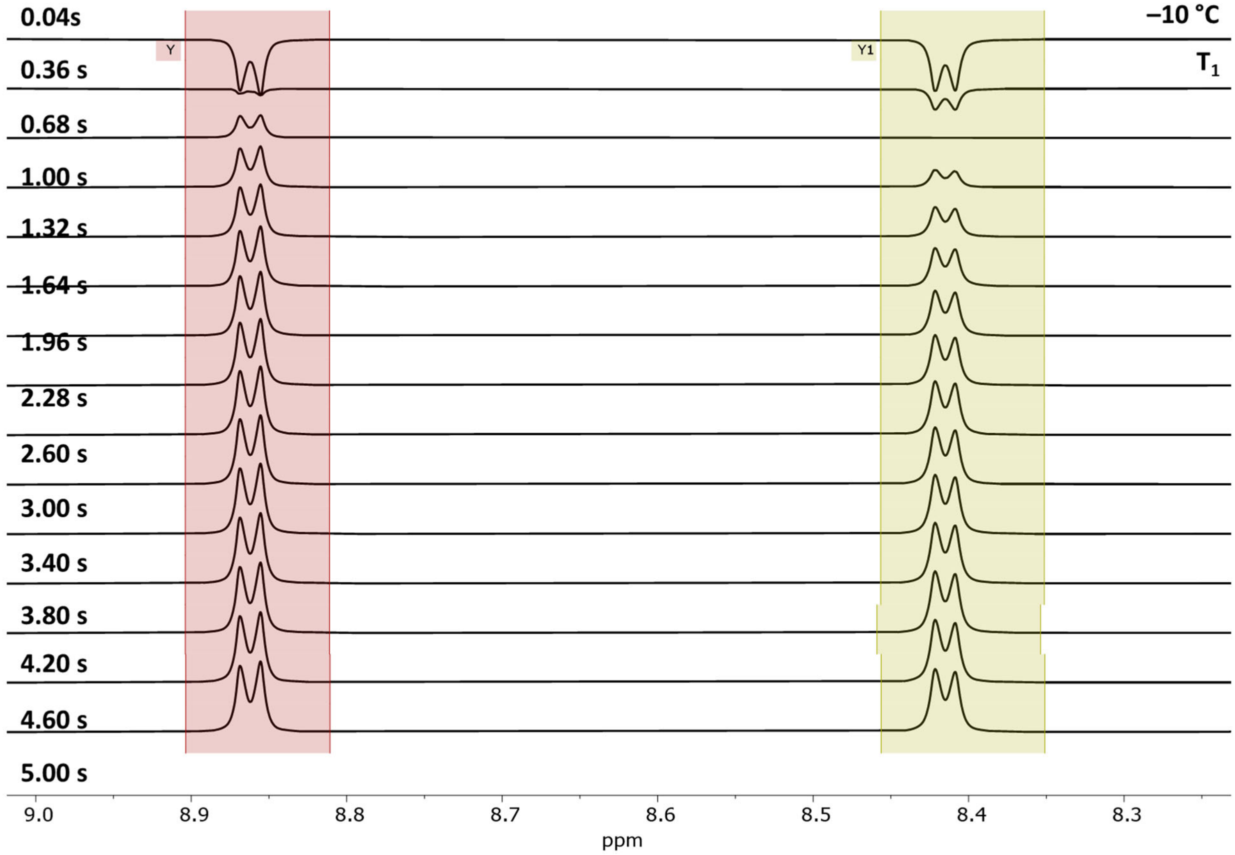

Supplementary Figure 88. T_1 (inverse recovery). Stacked spectra of the inverse recovery experiment to determine the T_1 values of the ortho (Y) and meta (Y1) protons of **MnRot** (0.5 mM) and **V1** (5 mM) in solution, in which the signal intensity (Y) is plotted against the time in seconds (X) (^1H, 500 MHz, chloroform-*d* : acetonitrile-*d*$_3$, 1:1, v/v, 263 K).

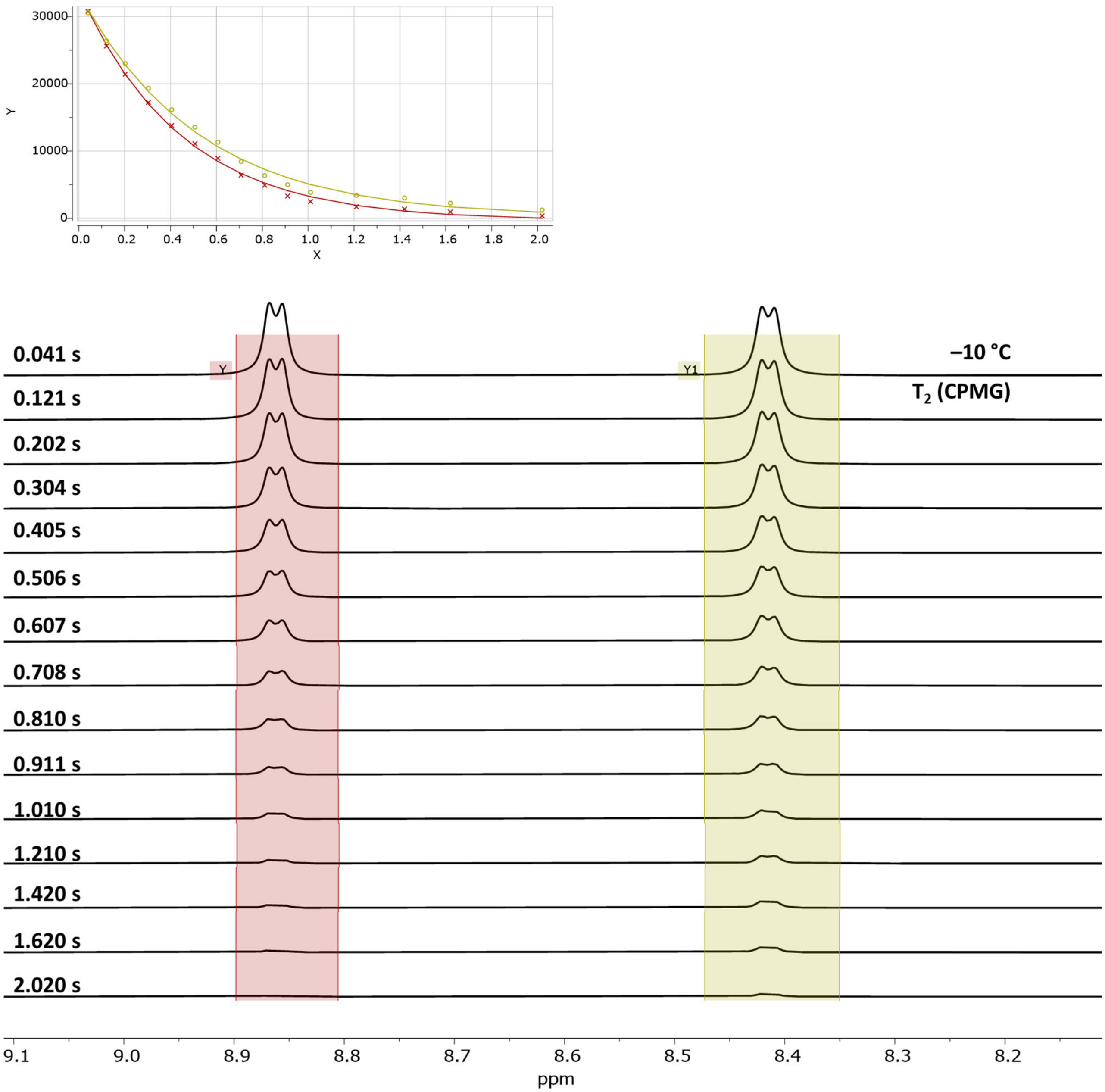

Supplementary Figure 89. T_2 (CPMG). Stacked spectra of the CPMG experiment to determine the T_2 values of the ortho (Y) and meta (Y1) protons of **MnRot** (0.5 mM) and **V1** (5 mM) in solution, in which the signal intensity (Y) is plotted against the time in seconds (X) (^{1}H, 500 MHz, chloroform-*d* : acetonitrile-*d*$_3$, 1:1, v/v, 263 K).

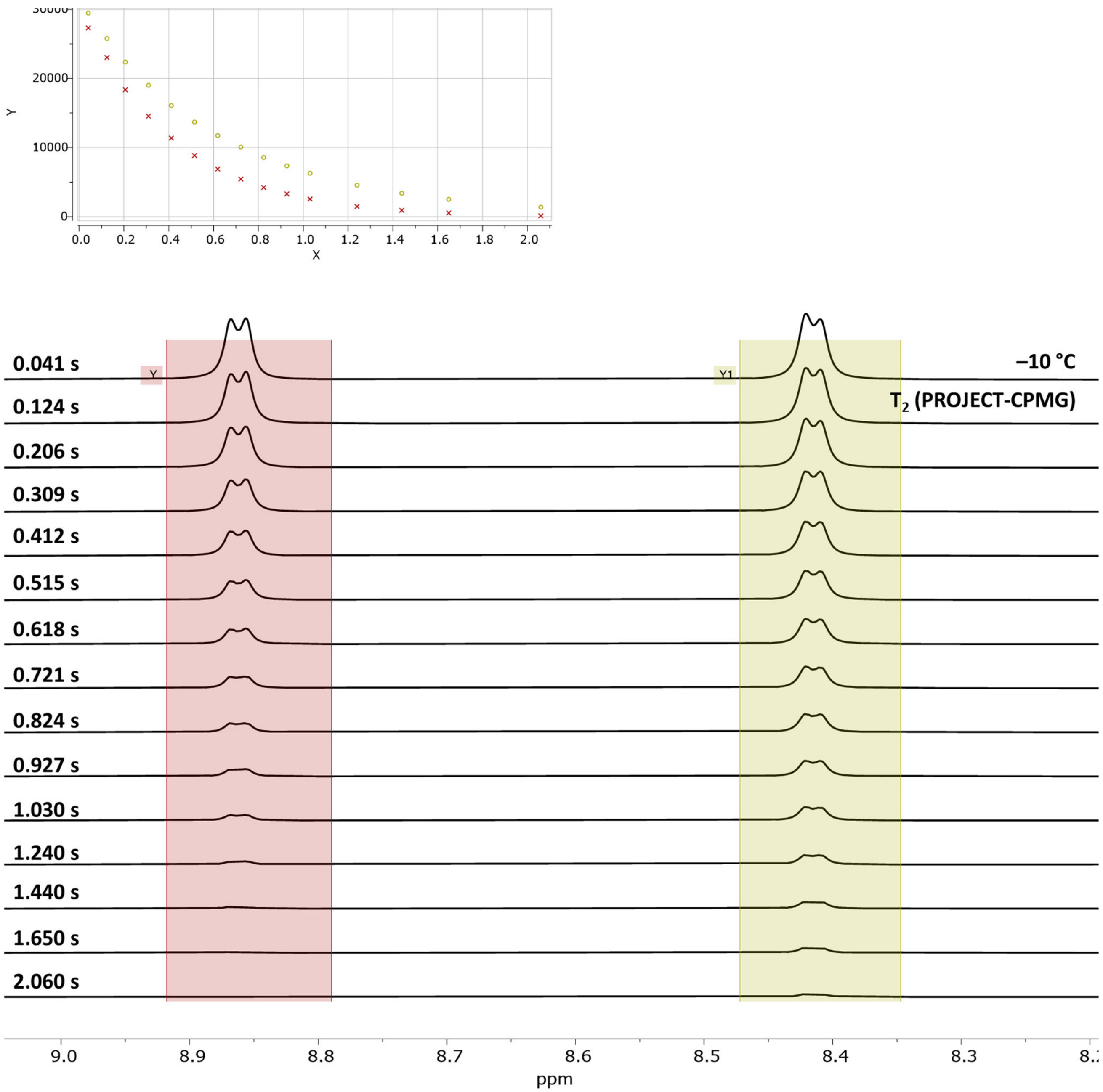

Supplementary Figure 90. T_2 (PROJECT-CPMG). Stacked spectra of the PROJECT-CPMG experiment to determine the T_2 values of the ortho (Y) and meta (Y1) protons of **MnRot** (0.5 mM) and **V1** (5 mM) in solution, in which the signal intensity (Y) is plotted against the time in seconds (X) (^{1}H, 500 MHz, chloroform-*d* : acetonitrile-*d*$_3$, 1:1, v/v, 263 K).

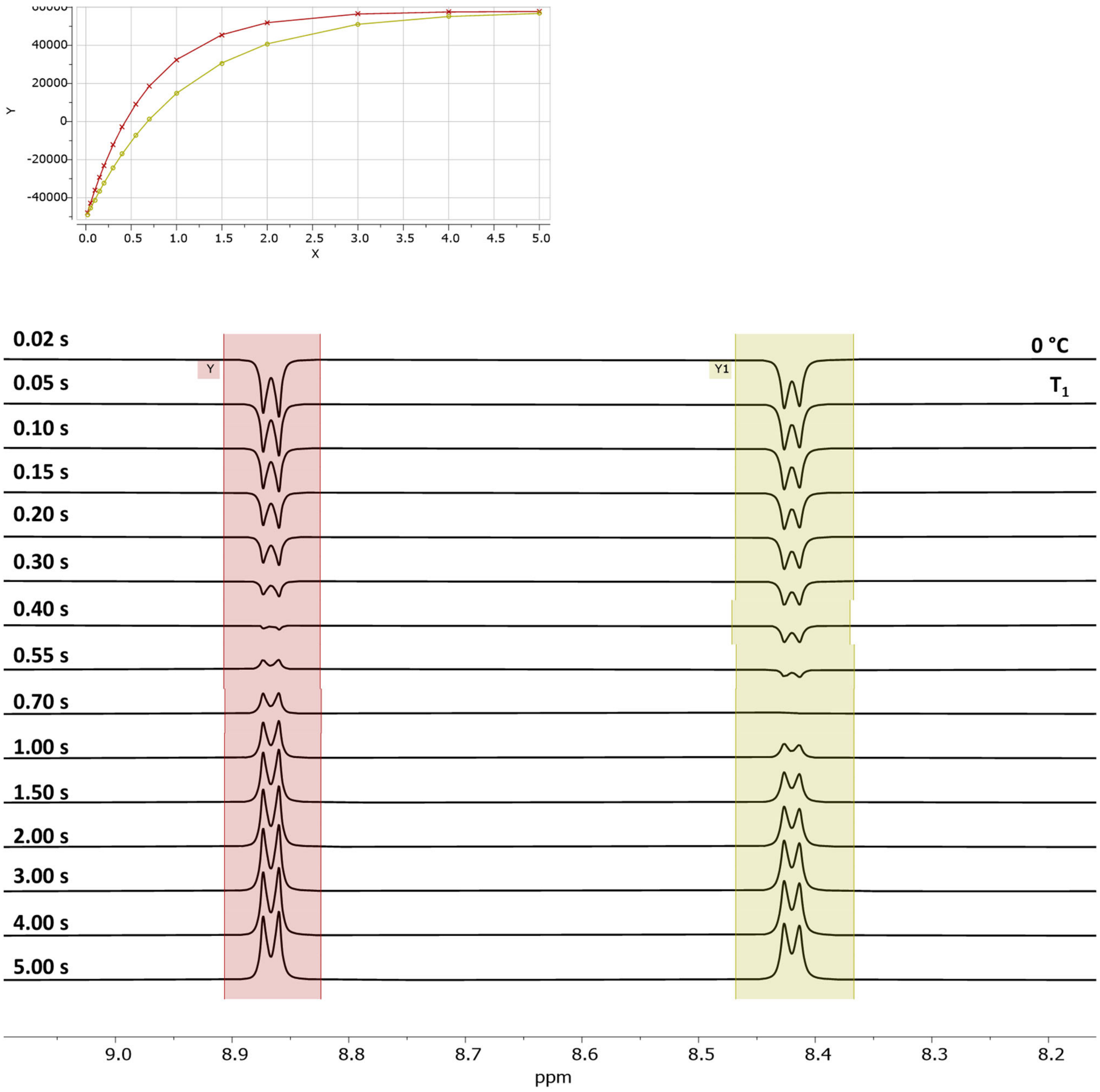

Supplementary Figure 91. T_1 (inverse recovery). Stacked spectra of the inverse recovery experiment to determine the T_1 values of the ortho (Y) and meta (Y1) protons of **MnRot** (0.5 mM) and **V1** (5 mM) in solution, in which the signal intensity (Y) is plotted against the time in seconds (X) (^{1}H, 500 MHz, chloroform-*d* : acetonitrile-*d*$_3$, 1:1, v/v, 273 K).

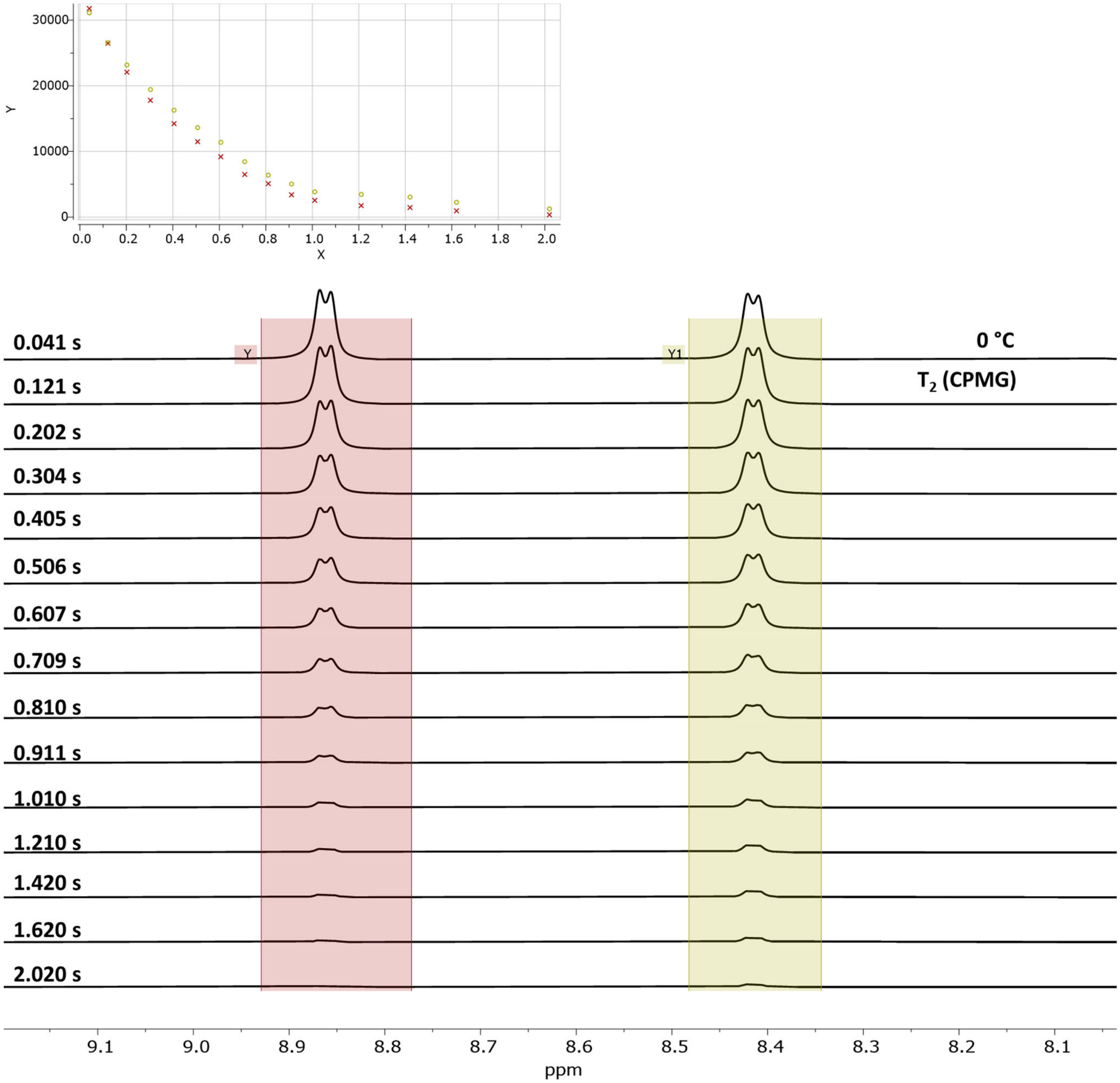

Supplementary Figure 92. T_2 (CPMG). Stacked spectra of the CPMG experiment to determine the T_2 values of the ortho (Y) and meta (Y1) protons of **MnRot** (0.5 mM) and **V1** (5 mM) in solution, in which the signal intensity (Y) is plotted against the time in seconds (X) (^{1}H, 500 MHz, chloroform-*d* : acetonitrile-*d*$_3$, 1:1, v/v, 273 K).

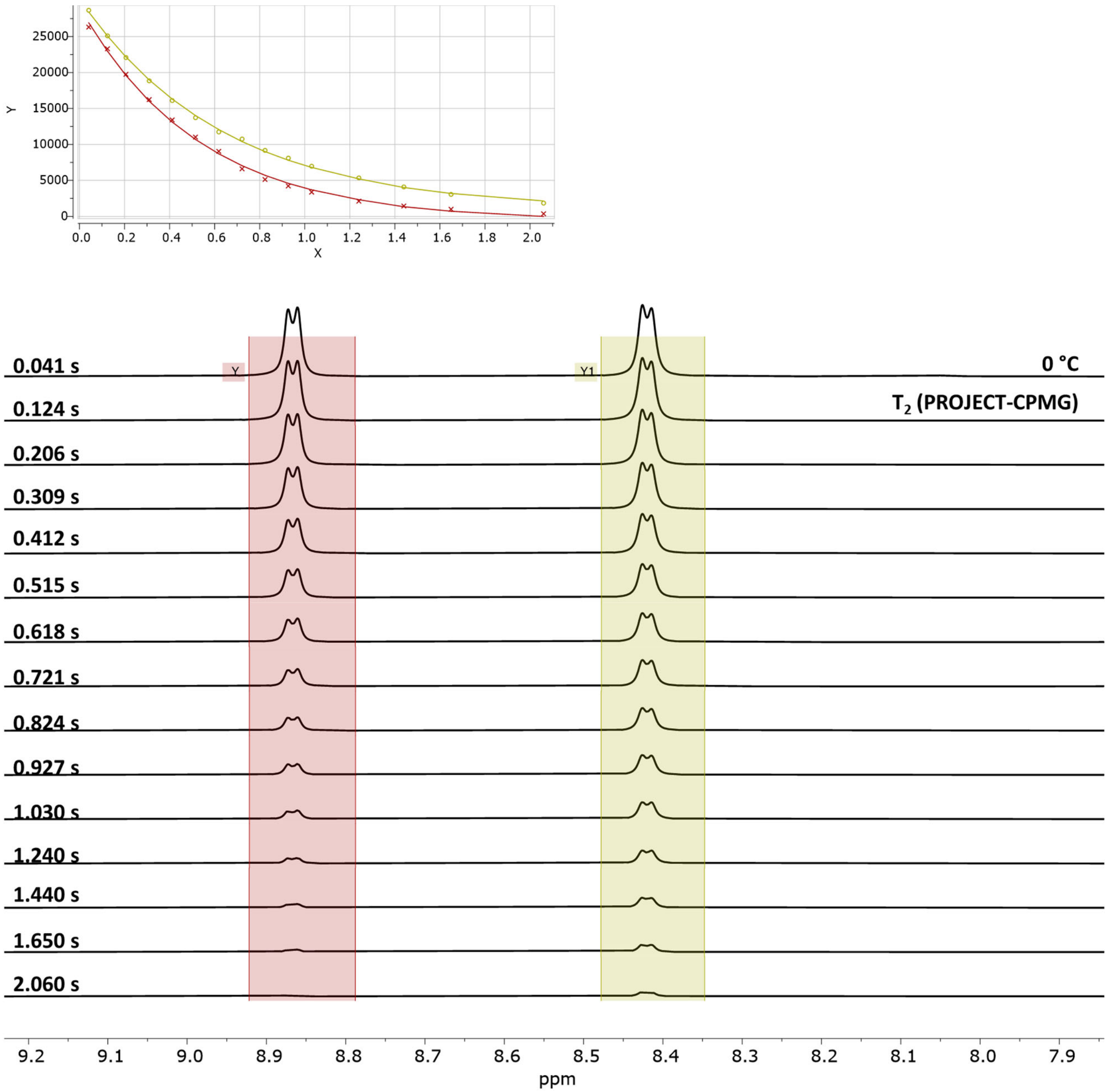

Supplementary Figure 93. T_2 (PROJECT-CPMG). Stacked spectra of the PROJECT-CPMG experiment to determine the T_2 values of the ortho (Y) and meta (Y1) protons of **MnRot** (0.5 mM) and **V1** (5 mM) in solution, in which the signal intensity (Y) is plotted against the time in seconds (X) (^{1}H, 500 MHz, chloroform-*d* : acetonitrile-*d*$_3$, 1:1, v/v, 273 K).

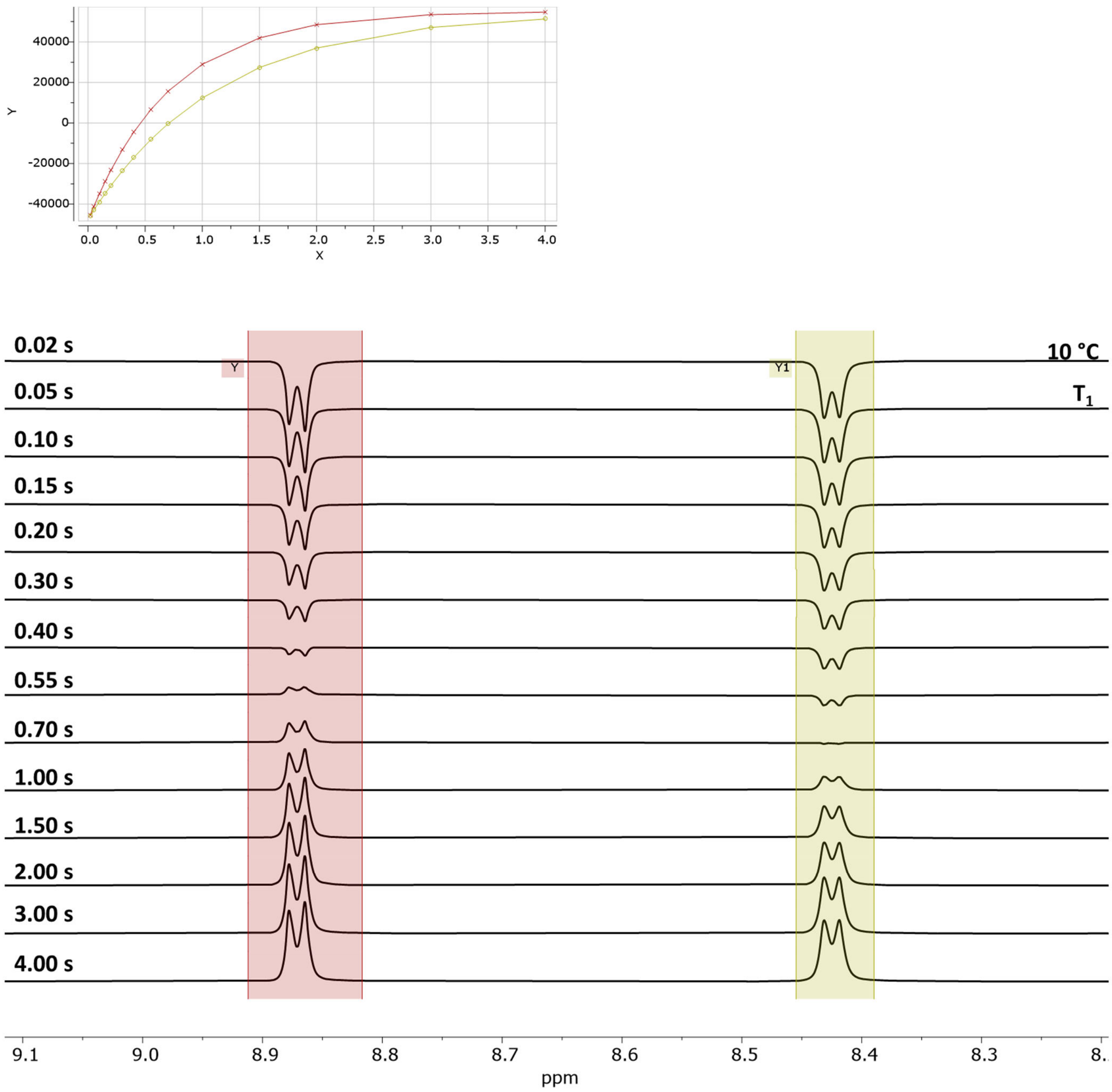

Supplementary Figure 94. T_1 (inverse recovery). Stacked spectra of the inverse recovery experiment to determine the T_1 values of the ortho (Y) and meta (Y1) protons of **MnRot** (0.5 mM) and **V1** (5 mM) in solution, in which the signal intensity (Y) is plotted against the time in seconds (X) (^1H, 500 MHz, chloroform-*d* : acetonitrile-*d*$_3$, 1:1, v/v, 283 K).

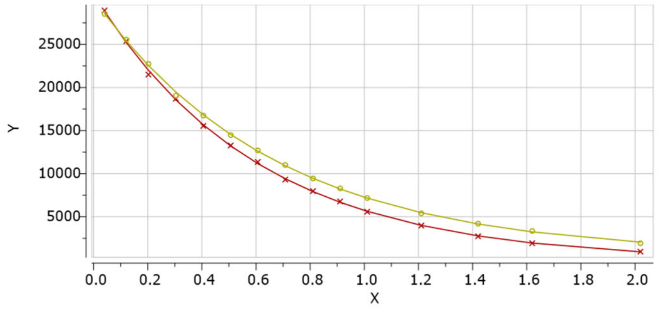

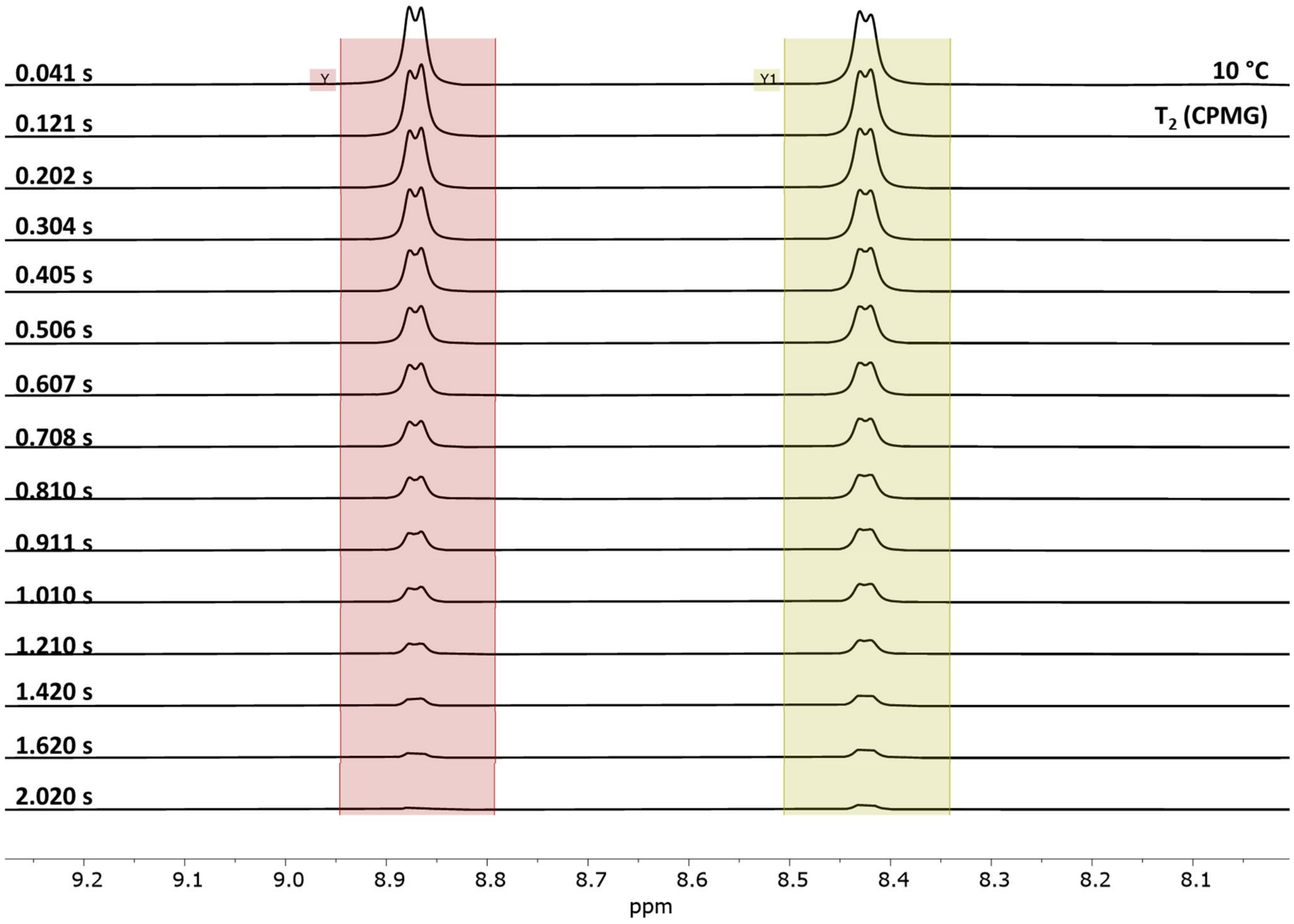

Supplementary Figure 95. T_2 (CPMG). Stacked spectra of the CPMG experiment to determine the T_2 values of the ortho (Y) and meta (Y1) protons of **MnRot** (0.5 mM) and **V1** (5 mM) in solution, in which the signal intensity (Y) is plotted against the time in seconds (X) (^{1}H, 500 MHz, chloroform-*d* : acetonitrile-d_3, 1:1, v/v, 283 K).

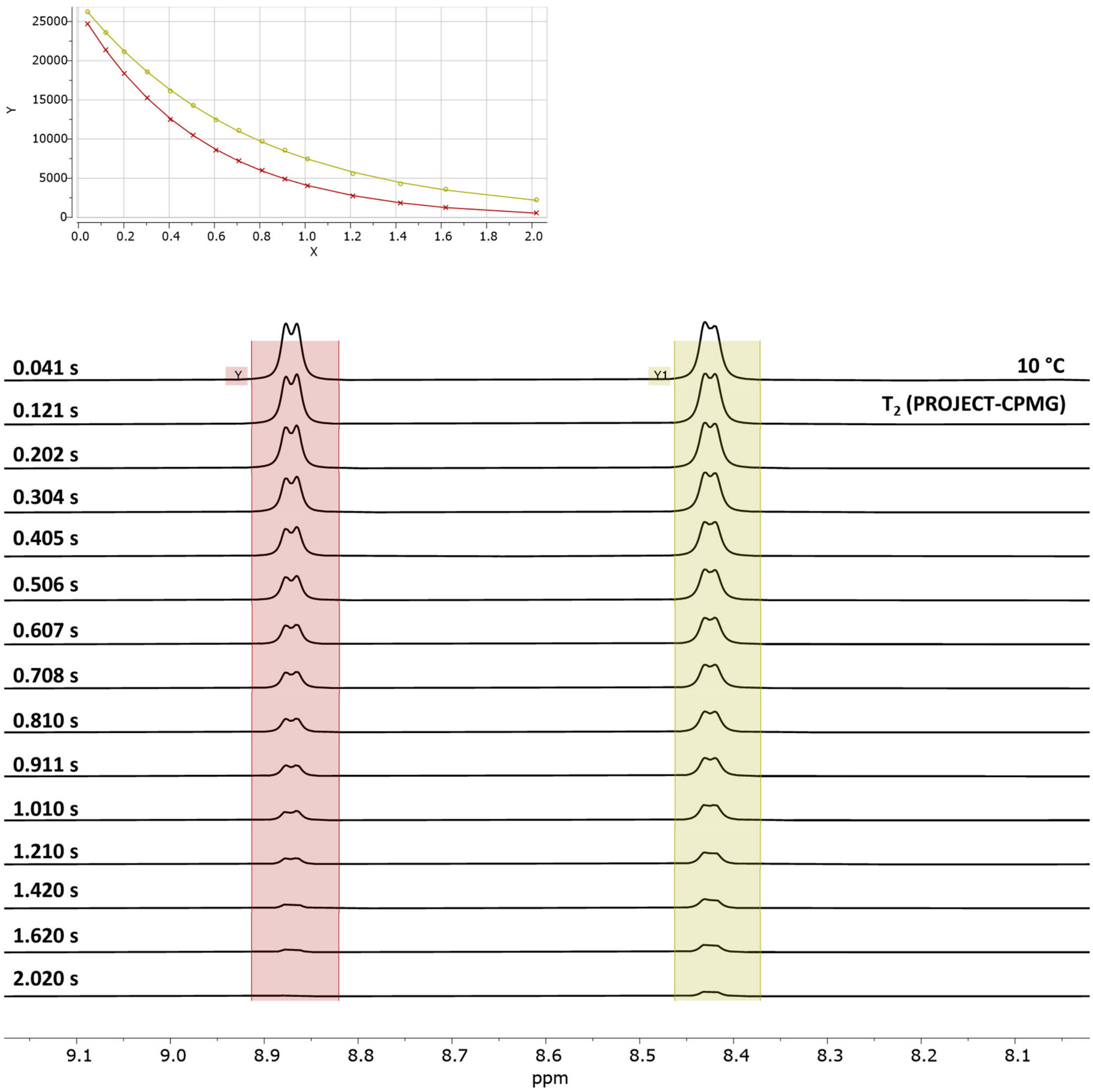

Supplementary Figure 96. T_2 (PROJECT-CPMG). Stacked spectra of the PROJECT-CPMG experiment to determine the T_2 values of the ortho (Y) and meta (Y1) protons of **MnRot** (0.5 mM) and **V1** (5 mM) in solution, in which the signal intensity (Y) is plotted against the time in seconds (X) (^{1}H, 500 MHz, chloroform-*d* : acetonitrile-*d*$_3$, 1:1, v/v, 283 K).

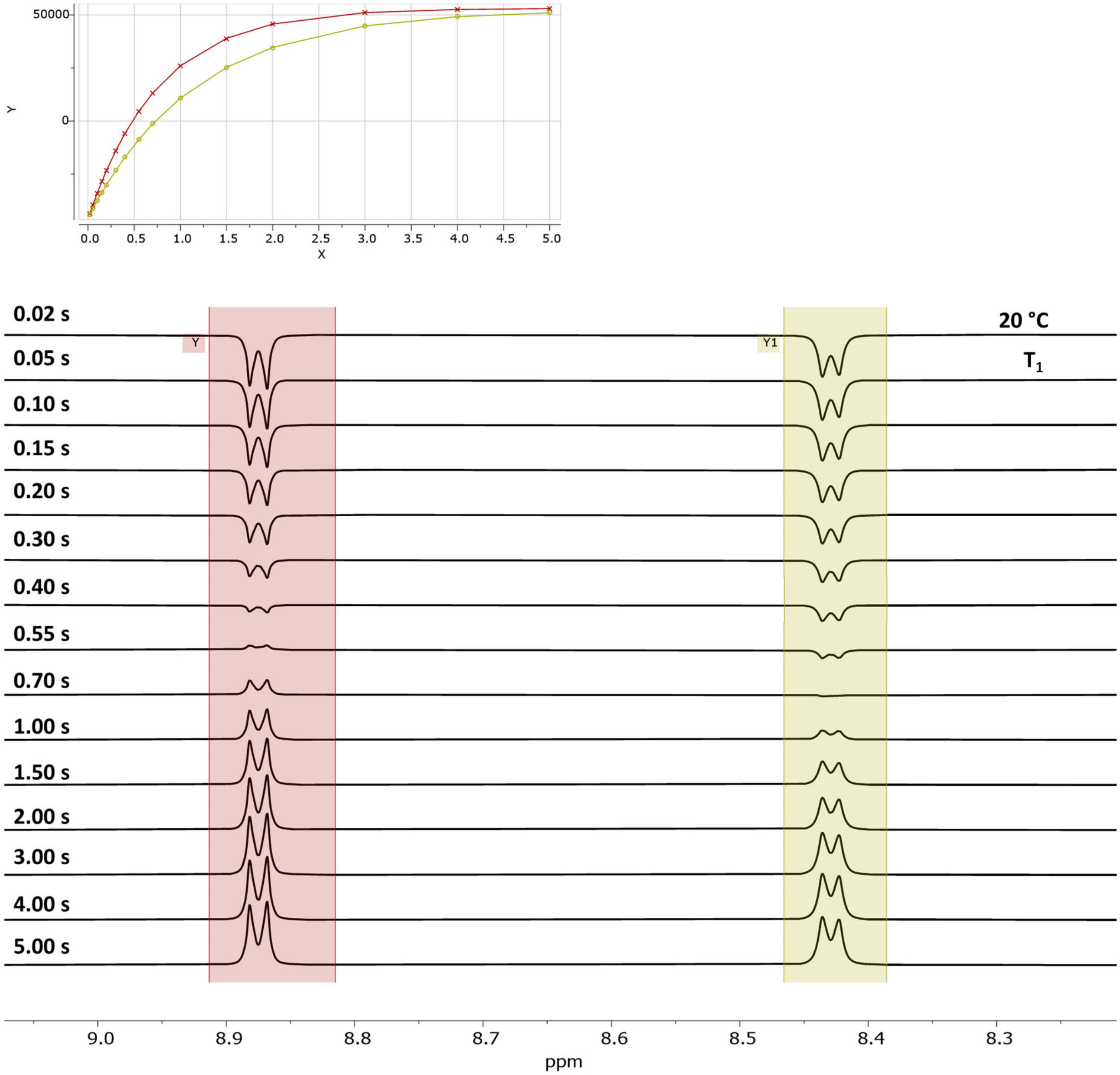

Supplementary Figure 97. T_1 (inverse recovery). Stacked spectra of the inverse recovery experiment to determine the T_1 values of the ortho (Y) and meta (Y1) protons of **MnRot** (0.5 mM) and **V1** (5 mM) in solution, in which the signal intensity (Y) is plotted against the time in seconds (X) (^{1}H, 500 MHz, chloroform-*d* : acetonitrile-*d*$_3$, 1:1, v/v, 293 K).

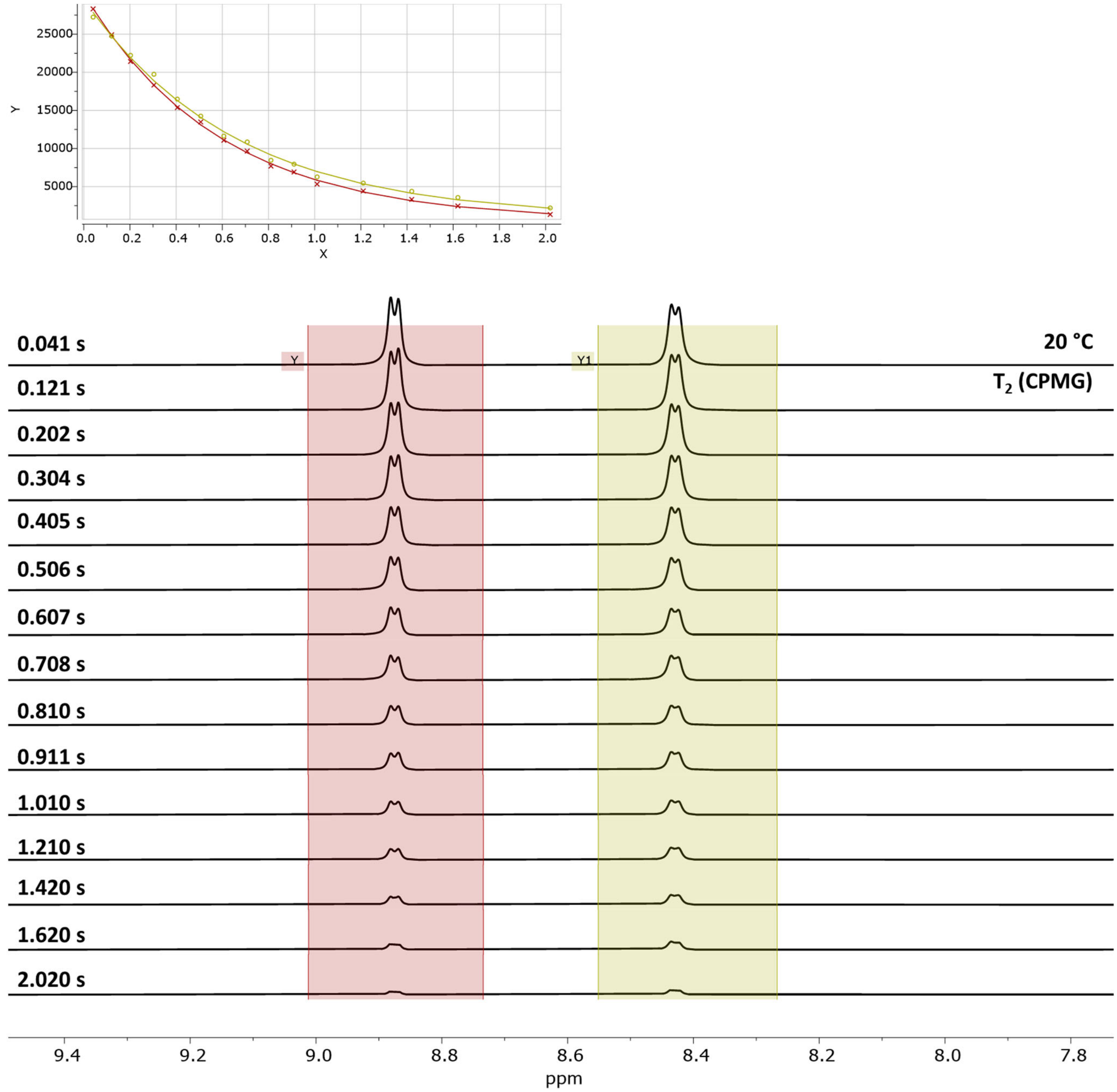

Supplementary Figure 98. T_2 (CPMG). Stacked spectra of the CPMG experiment to determine the T_2 values of the ortho (Y) and meta (Y1) protons of **MnRot** (0.5 mM) and **V1** (5 mM) in solution, in which the signal intensity (Y) is plotted against the time in seconds (X) (^{1}H, 500 MHz, chloroform-*d* : acetonitrile-*d*$_3$, 1:1, v/v, 293 K).

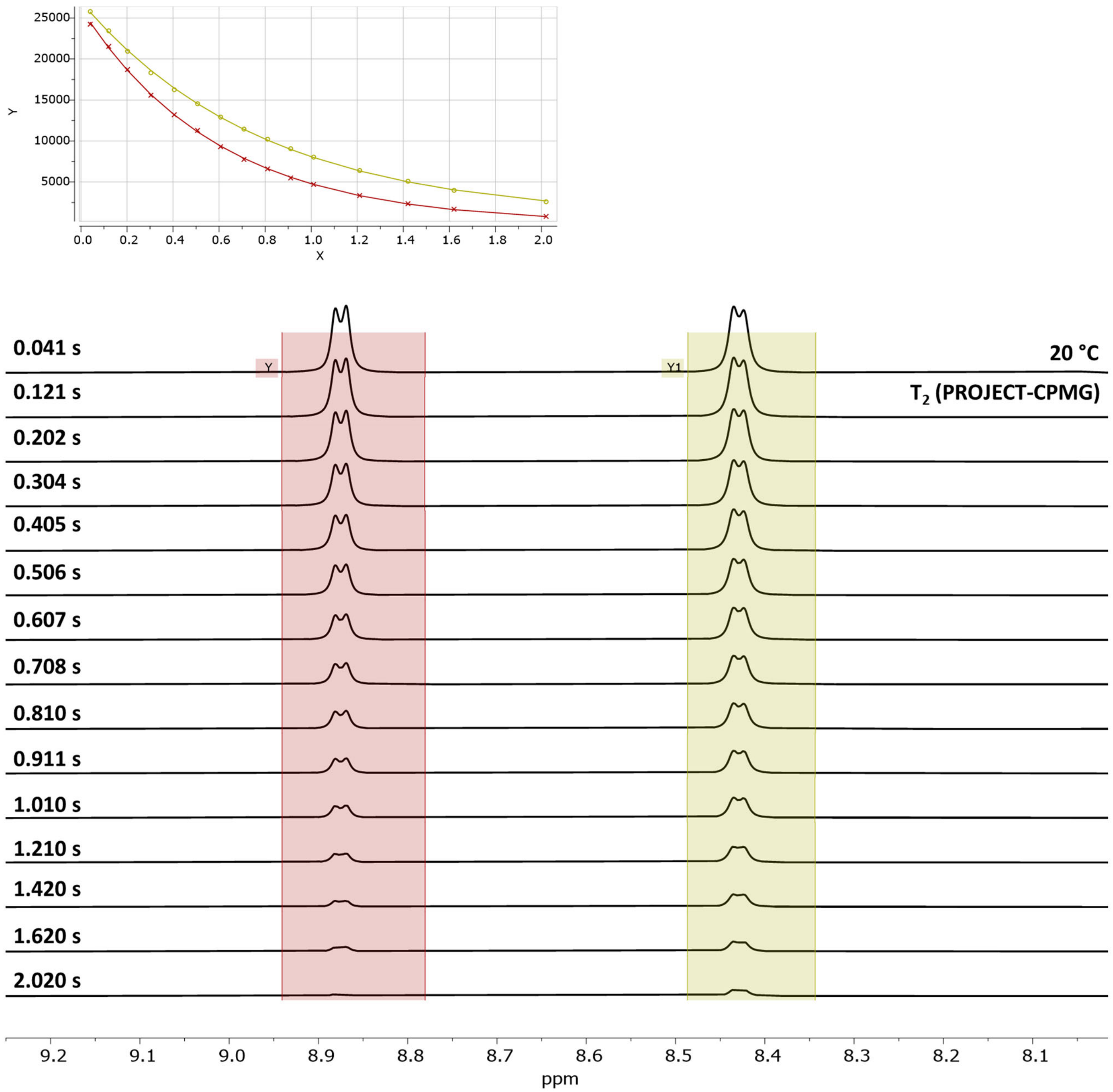

Supplementary Figure 99. T_2 (PROJECT-CPMG). Stacked spectra of the PROJECT-CPMG experiment to determine the T_2 values of the ortho (Y) and meta (Y1) protons of **MnRot** (0.5 mM) and **V1** (5 mM) in solution, in which the signal intensity (Y) is plotted against the time in seconds (X) (^{1}H, 500 MHz, chloroform-*d* : acetonitrile-*d*$_3$, 1:1, v/v, 293 K).

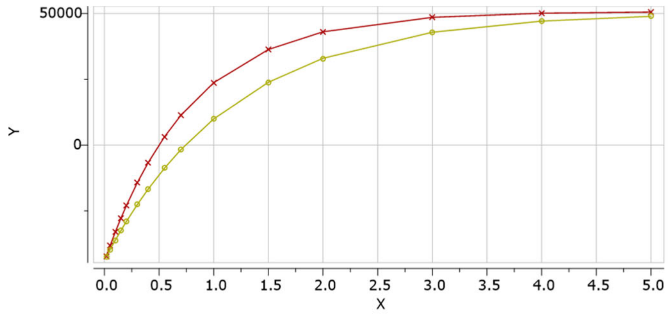

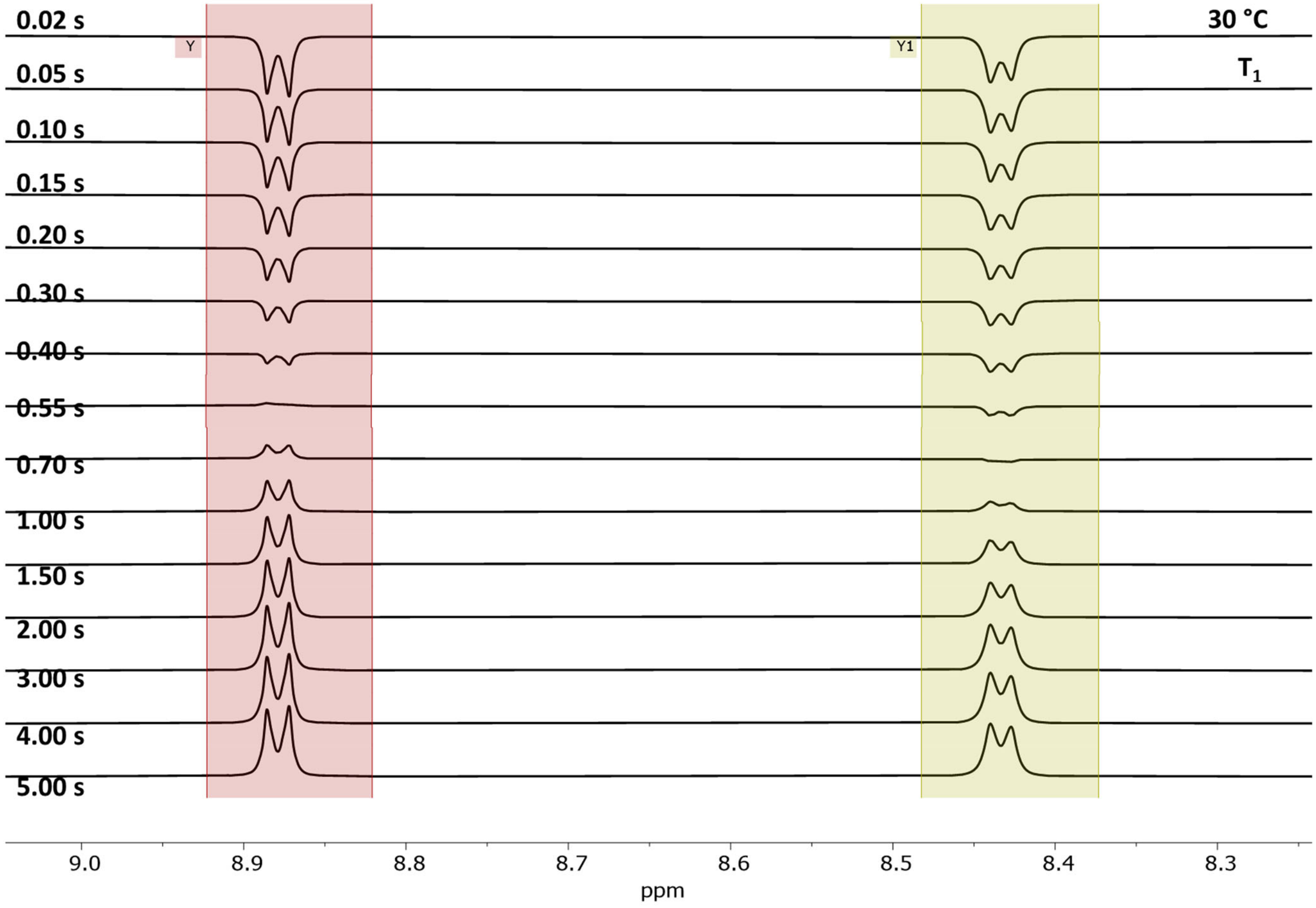

Supplementary Figure 100. T_1 (inverse recovery). Stacked spectra of the inverse recovery experiment to determine the T_1 values of the ortho (Y) and meta (Y1) protons of **MnRot** (0.5 mM) and **V1** (5 mM) in solution, in which the signal intensity (Y) is plotted against the time in seconds (X) (^{1}H, 500 MHz, chloroform-*d* : acetonitrile-d_3, 1:1, v/v, 303 K).

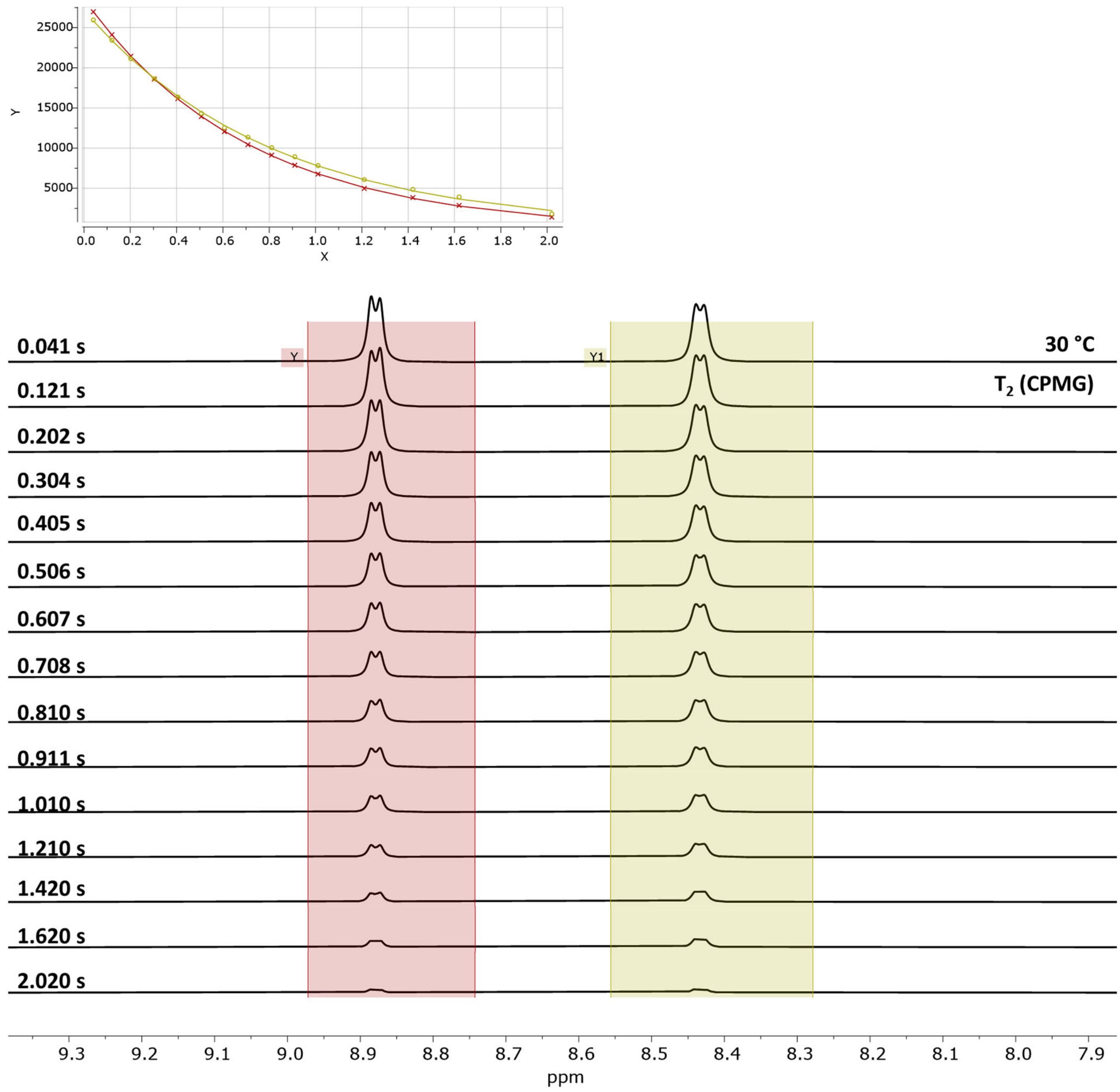

Supplementary Figure 101. T_2 (CPMG). Stacked spectra of the CPMG experiment to determine the T_2 values of the ortho (Y) and meta (Y1) protons of **MnRot** (0.5 mM) and **V1** (5 mM) in solution, in which the signal intensity (Y) is plotted against the time in seconds (X) (^{1}H, 500 MHz, chloroform-*d* : acetonitrile-d_3, 1:1, v/v, 303 K).

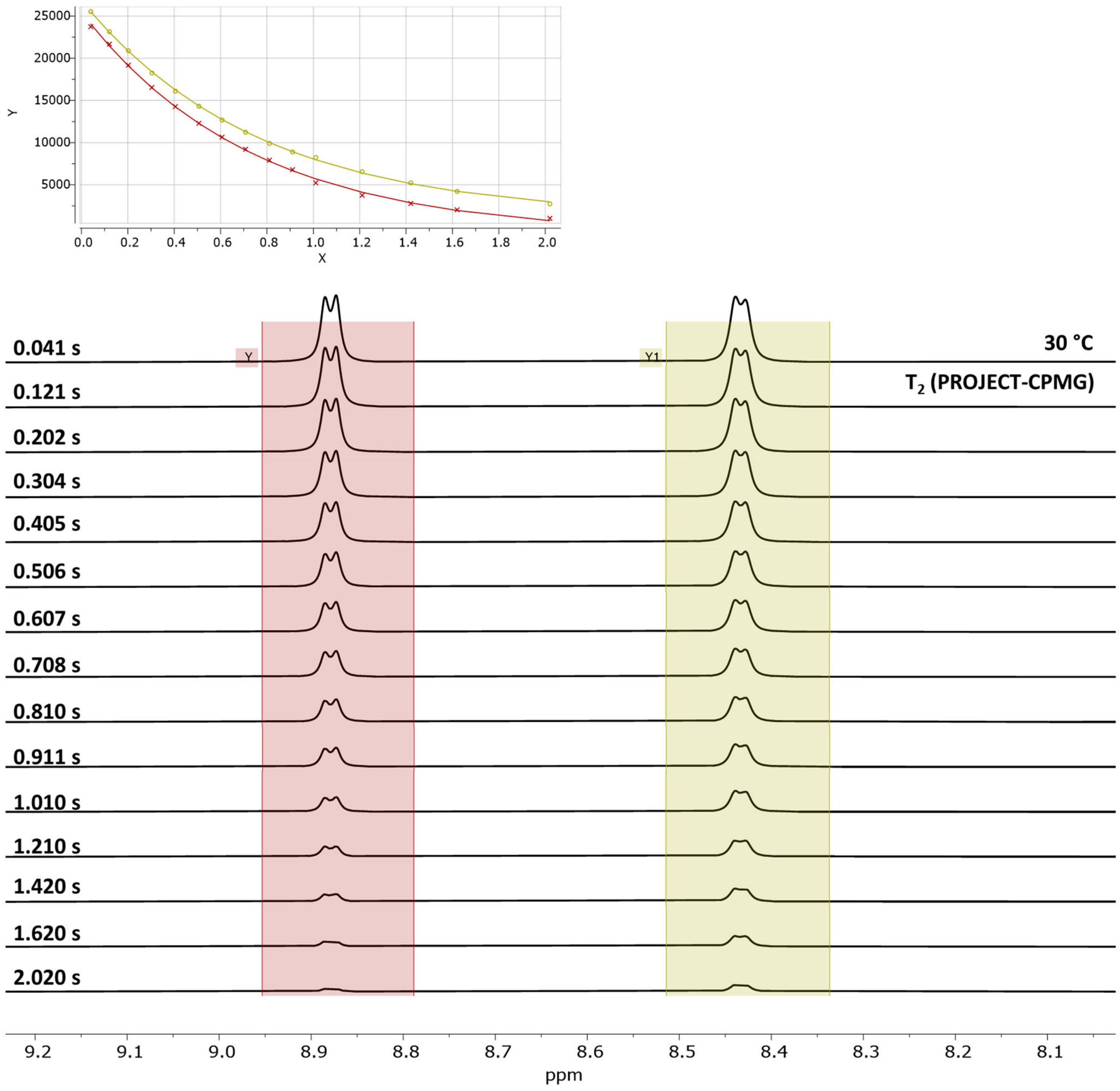

Supplementary Figure 102. T_2 (PROJECT-CPMG). Stacked spectra of the PROJECT-CPMG experiment to determine the T_2 values of the ortho (Y) and meta (Y1) protons of **MnRot** (0.5 mM) and **V1** (5 mM) in solution, in which the signal intensity (Y) is plotted against the time in seconds (X) (^{1}H, 500 MHz, chloroform-*d* : acetonitrile-*d*$_3$, 1:1, v/v, 303 K).

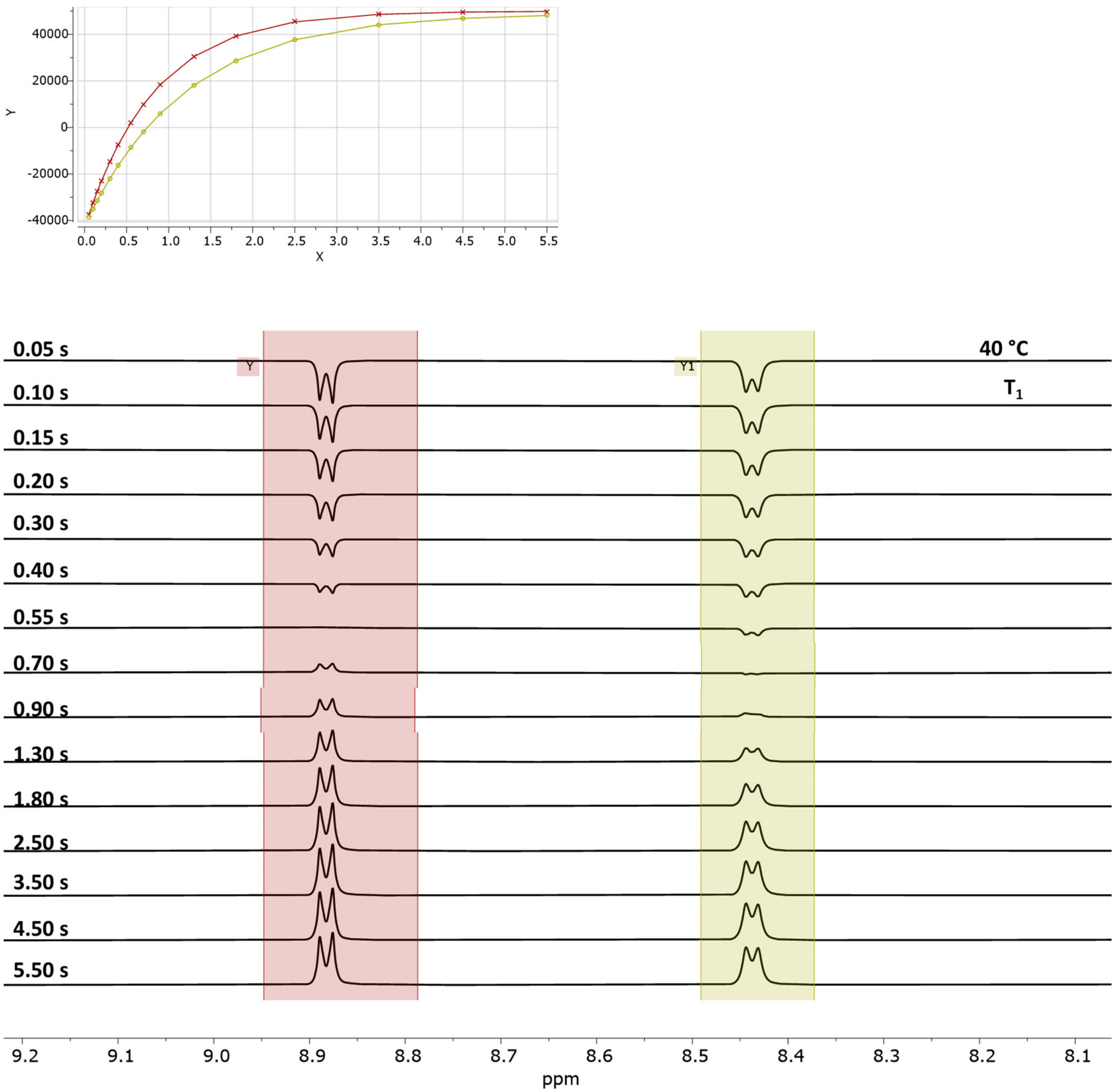

Supplementary Figure 103. T_1 (inverse recovery). Stacked spectra of the inverse recovery experiment to determine the T_1 values of the ortho (Y) and meta (Y1) protons of **MnRot** (0.5 mM) and **V1** (5 mM) in solution, in which the signal intensity (Y) is plotted against the time in seconds (X) (^{1}H, 500 MHz, chloroform-*d* : acetonitrile-*d*$_3$, 1:1, v/v, 313 K).

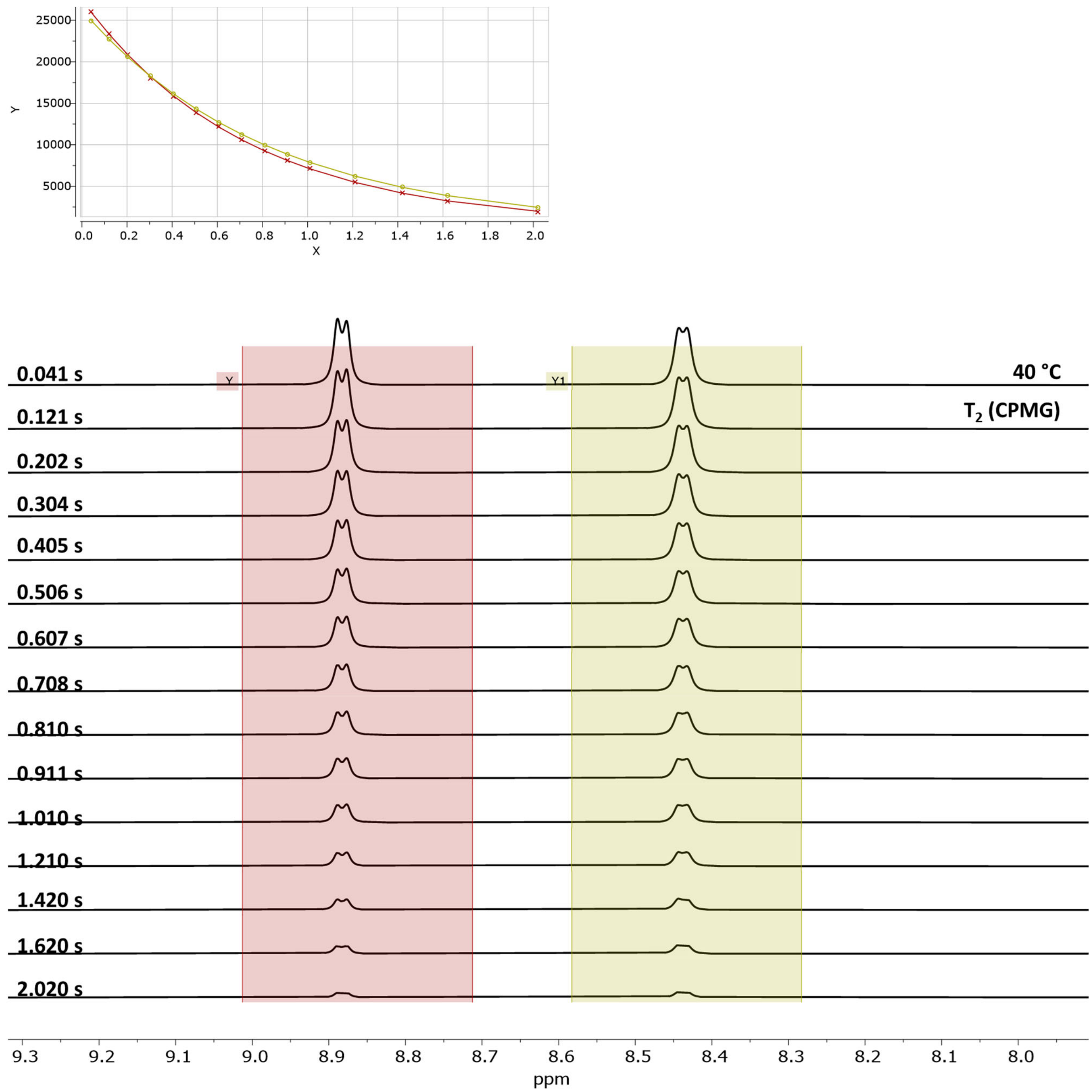

Supplementary Figure 104. T_2 (CPMG). Stacked spectra of the CPMG experiment to determine the T_2 values of the ortho (Y) and meta (Y1) protons of **MnRot** (0.5 mM) and **V1** (5 mM) in solution, in which the signal intensity (Y) is plotted against the time in seconds (X) (^{1}H, 500 MHz, chloroform-*d* : acetonitrile-*d*$_3$, 1:1, v/v, 313 K).

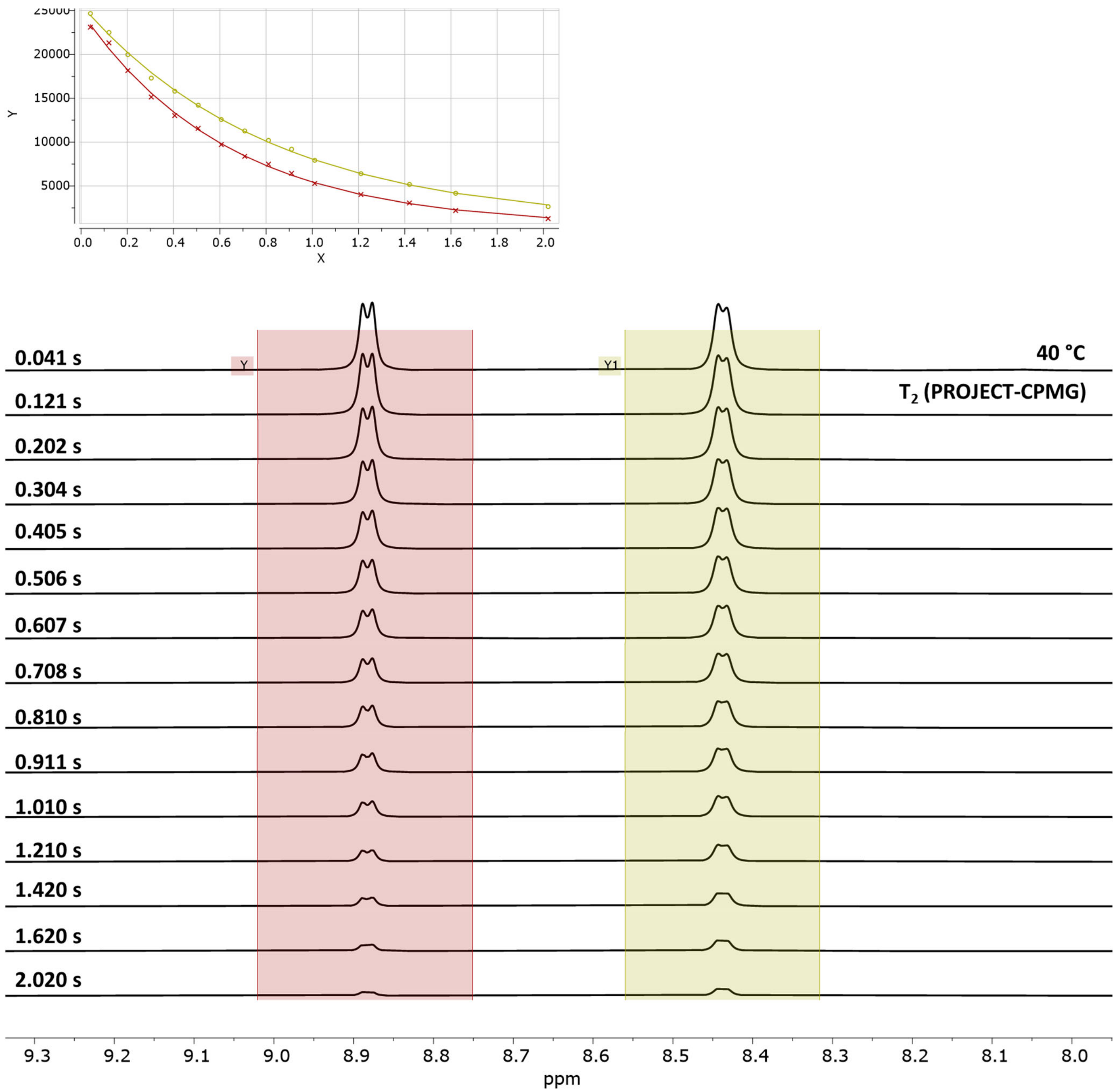

Supplementary Figure 105. T_2 (PROJECT-CPMG). Stacked spectra of the PROJECT-CPMG experiment to determine the T_2 values of the ortho (Y) and meta (Y1) protons of **MnRot** (0.5 mM) and **V1** (5 mM) in solution, in which the signal intensity (Y) is plotted against the time in seconds (X) (^{1}H, 500 MHz, chloroform-*d* : acetonitrile-*d₃*, 1:1, v/v, 313 K).

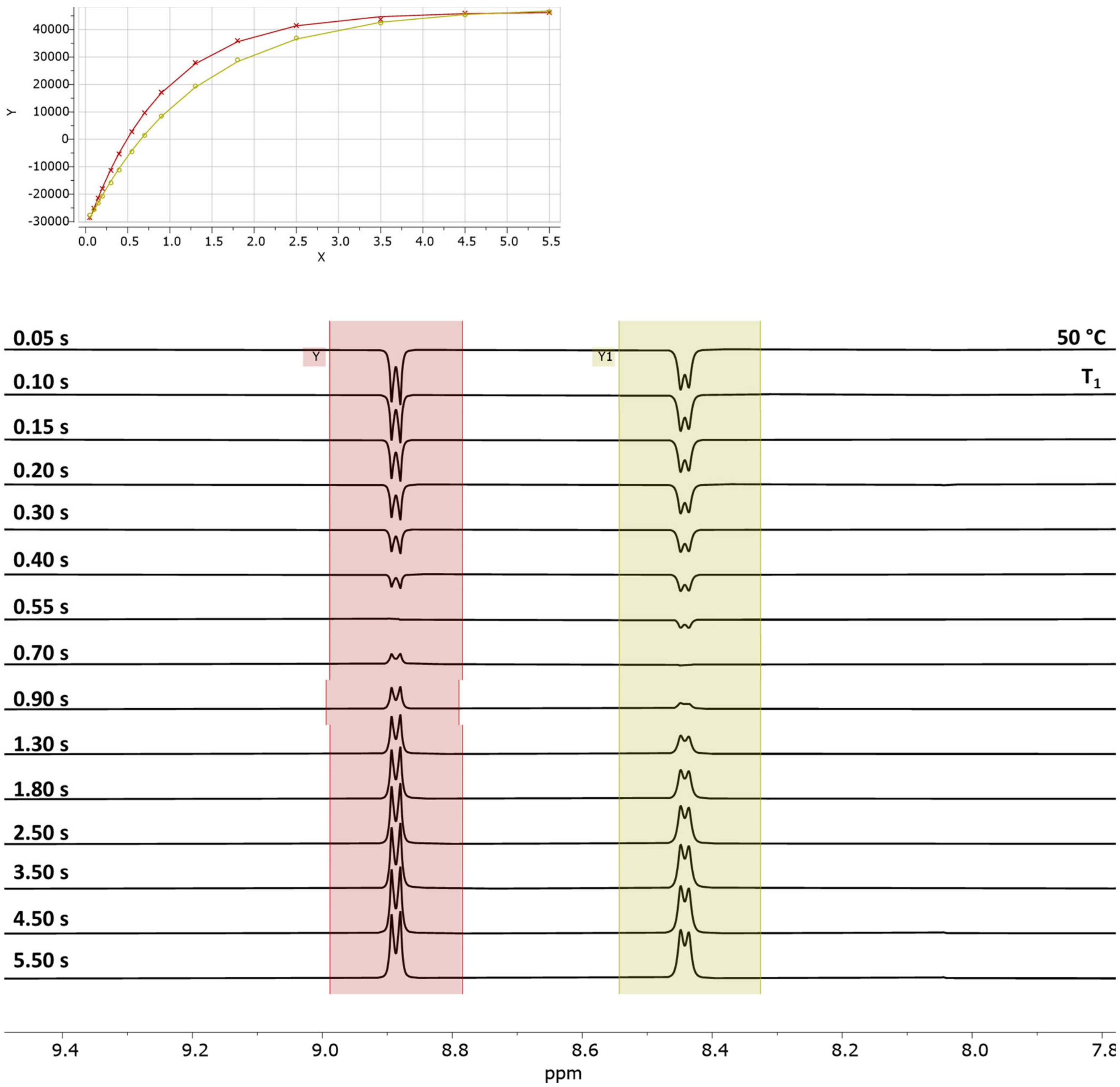

Supplementary Figure 106. T_1 (inverse recovery). Stacked spectra of the inverse recovery experiment to determine the T_1 values of the ortho (Y) and meta (Y1) protons of **MnRot** (0.5 mM) and **V1** (5 mM) in solution, in which the signal intensity (Y) is plotted against the time in seconds (X) (^{1}H, 500 MHz, chloroform-*d* : acetonitrile-*d$_3$*, 1:1, v/v, 323 K).

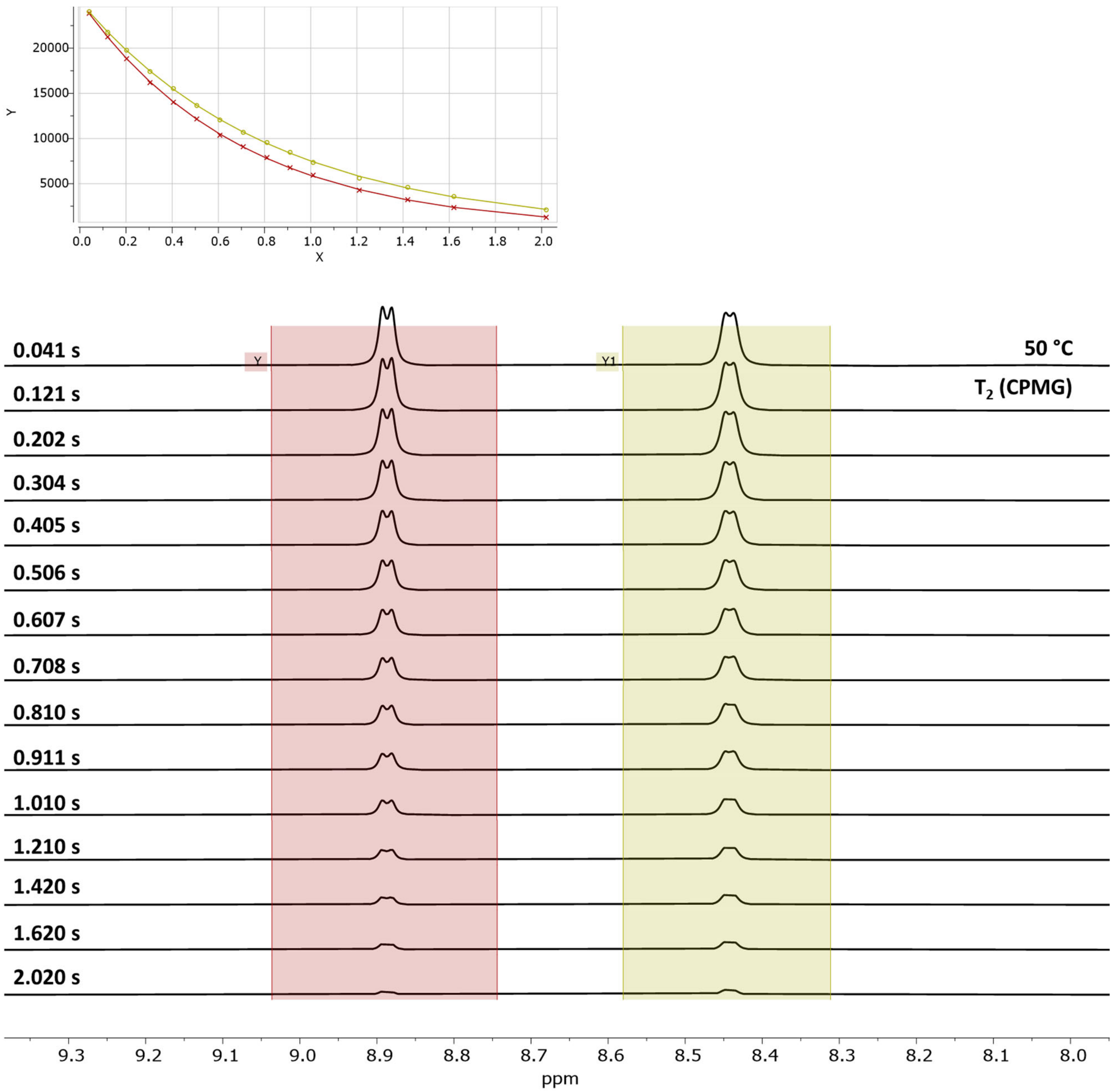

Supplementary Figure 107. T_2 (CPMG). Stacked spectra of the CPMG experiment to determine the T_2 values of the ortho (Y) and meta (Y1) protons of **MnRot** (0.5 mM) and **V1** (5 mM) in solution, in which the signal intensity (Y) is plotted against the time in seconds (X) (^{1}H, 500 MHz, chloroform-*d* : acetonitrile-d_3, 1:1, v/v, 323 K).

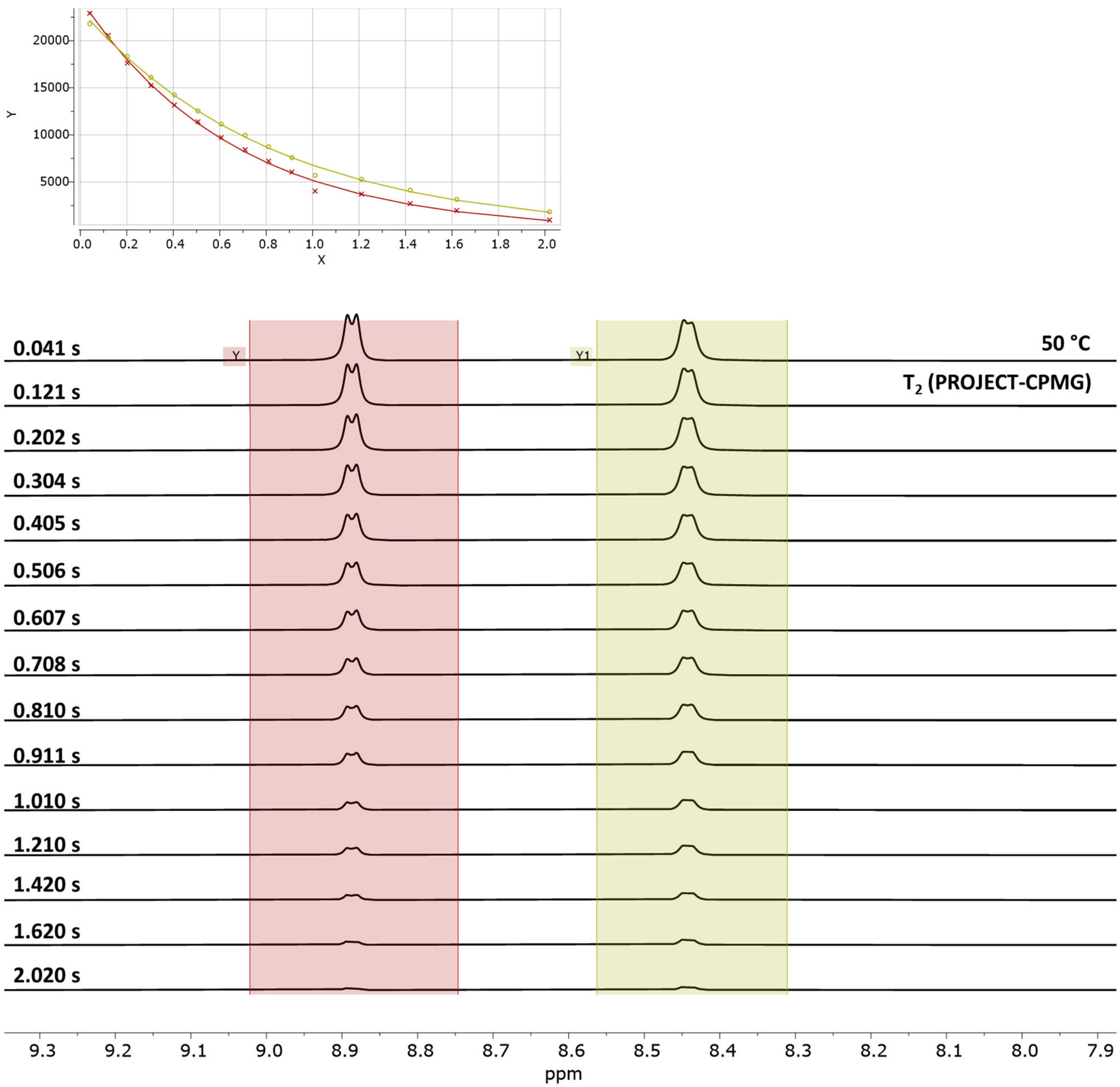

Supplementary Figure 108. T_2 (PROJECT-CPMG). Stacked spectra of the PROJECT-CPMG experiment to determine the T_2 values of the ortho (Y) and meta (Y1) protons of **MnRot** (0.5 mM) and **V1** (5 mM) in solution, in which the signal intensity (Y) is plotted against the time in seconds (X) (^{1}H, 500 MHz, chloroform-*d* : acetonitrile-*d*$_3$, 1:1, v/v, 323 K).

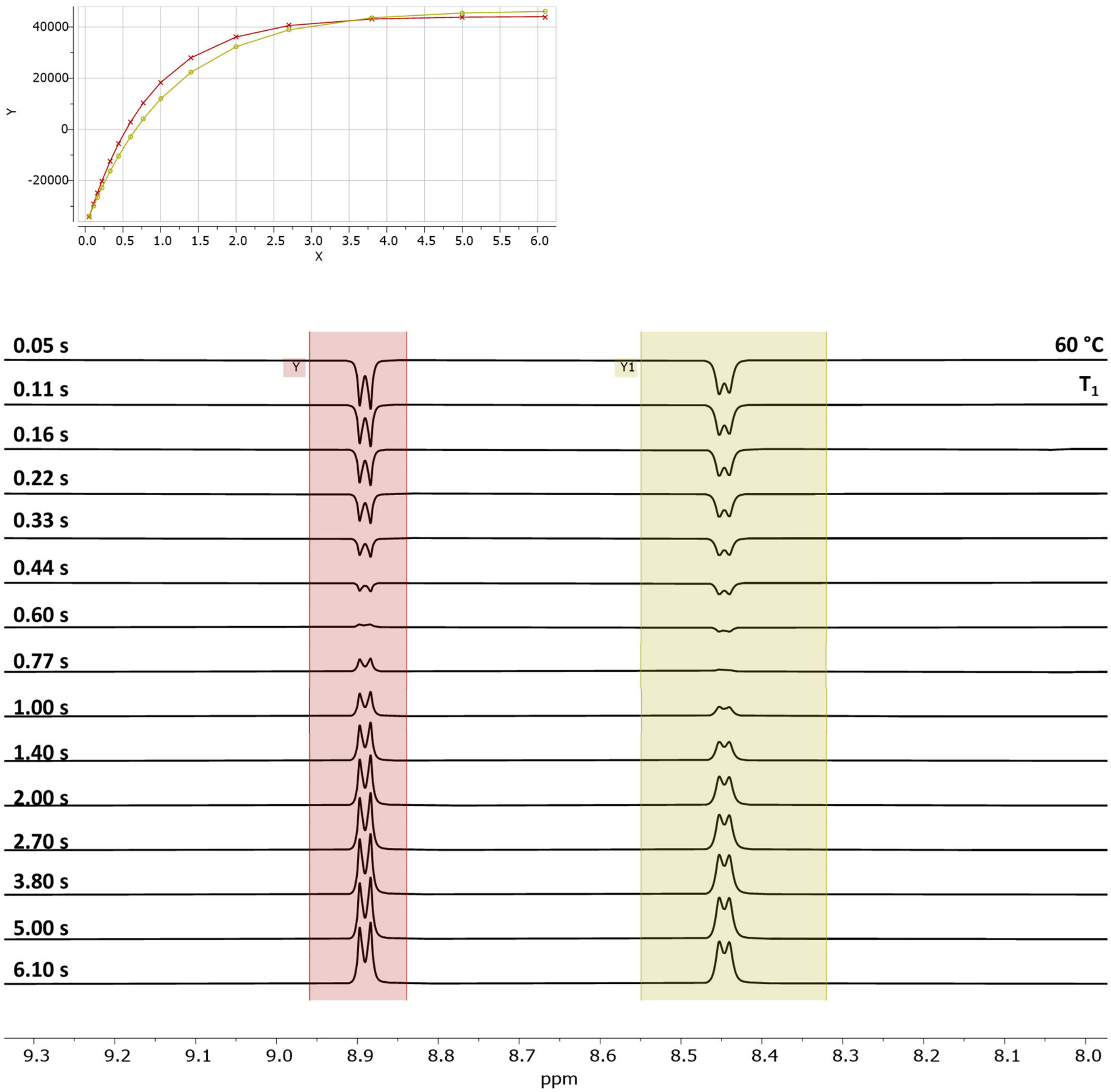

Supplementary Figure 109. T_1 (inverse recovery). Stacked spectra of the inverse recovery experiment to determine the T_1 values of the ortho (Y) and meta (Y1) protons of **MnRot** (0.5 mM) and **V1** (5 mM) in solution, in which the signal intensity (Y) is plotted against the time in seconds (X) (^{1}H, 500 MHz, chloroform-*d* : acetonitrile-*d*$_3$, 1:1, v/v, 333 K).

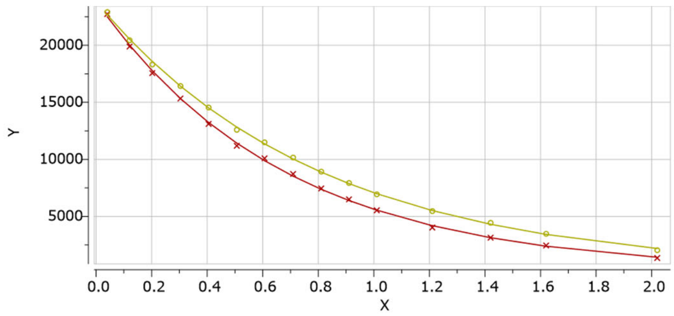

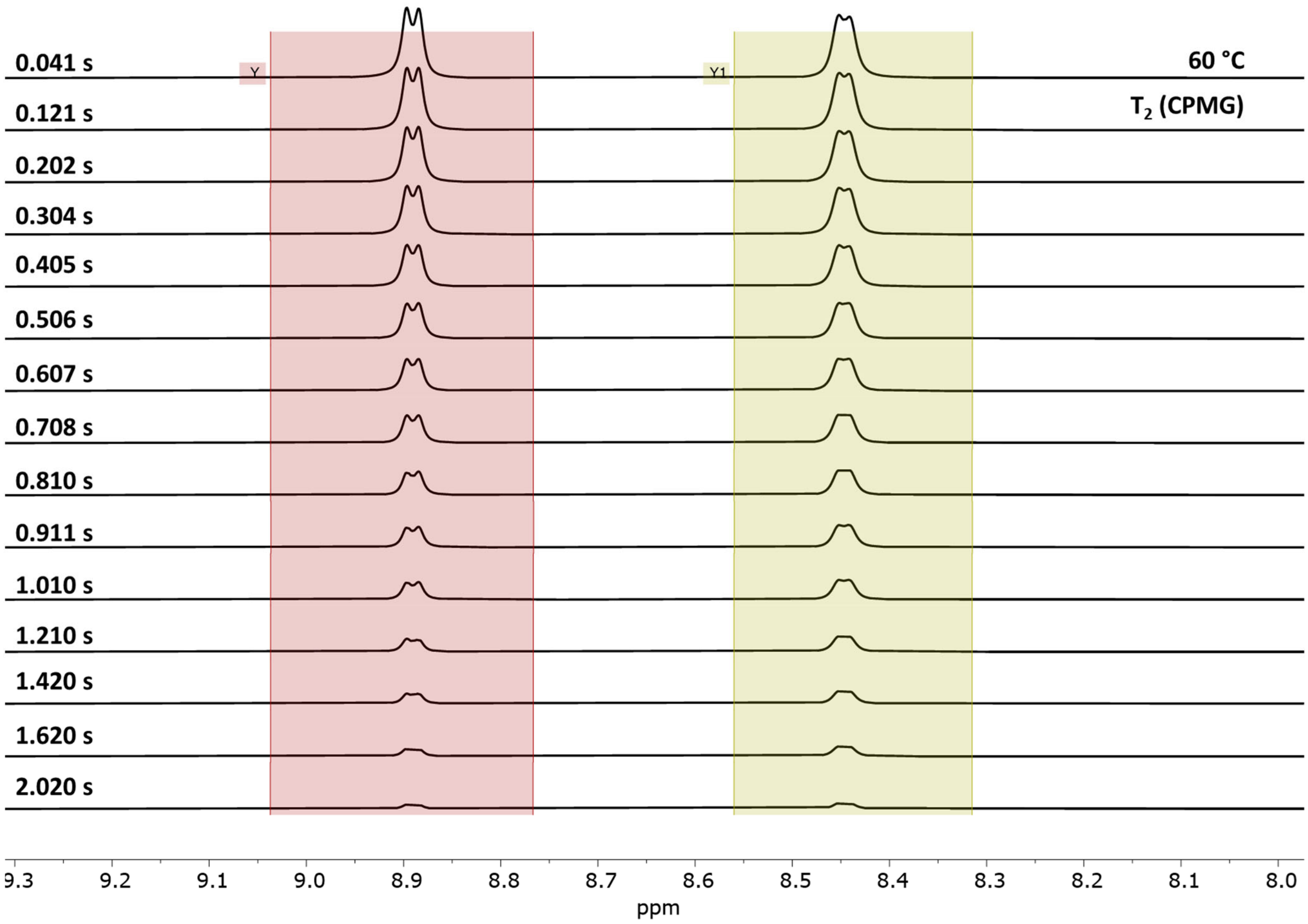

Supplementary Figure 110. T_2 (CPMG). Stacked spectra of the CPMG experiment to determine the T_2 values of the ortho (Y) and meta (Y1) protons of **MnRot** (0.5 mM) and **V1** (5 mM) in solution, in which the signal intensity (Y) is plotted against the time in seconds (X) (^{1}H, 500 MHz, chloroform-*d* : acetonitrile-*d*$_3$, 1:1, v/v, 333 K).

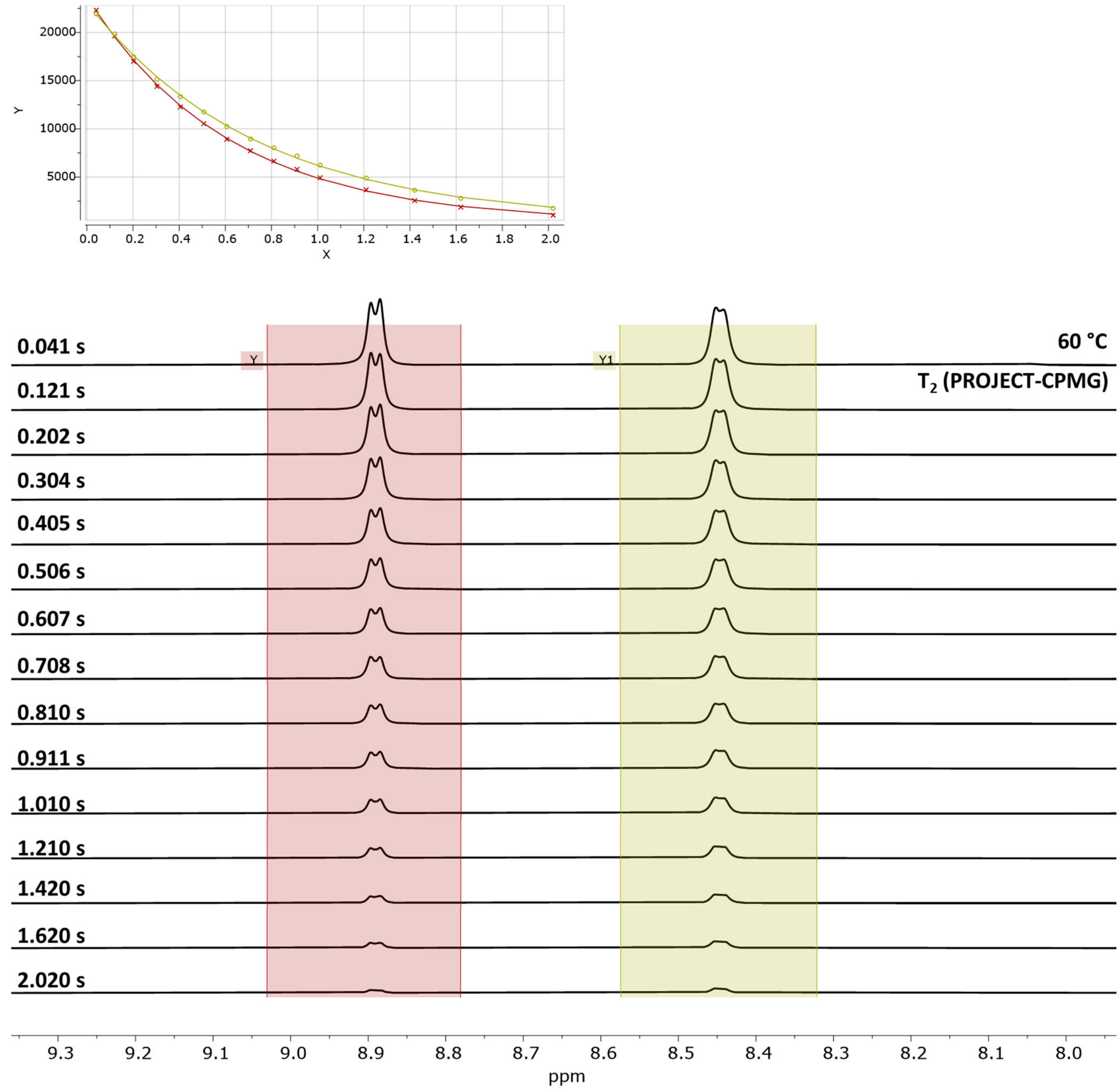

Supplementary Figure 111. T_2 (PROJECT-CPMG). Stacked spectra of the PROJECT-CPMG experiment to determine the T_2 values of the ortho (Y) and meta (Y1) protons of **MnRot** (0.5 mM) and **V1** (5 mM) in solution, in which the signal intensity (Y) is plotted against the time in seconds (X) (^{1}H, 500 MHz, chloroform-*d* : acetonitrile-*d*$_3$, 1:1, v/v, 333 K).

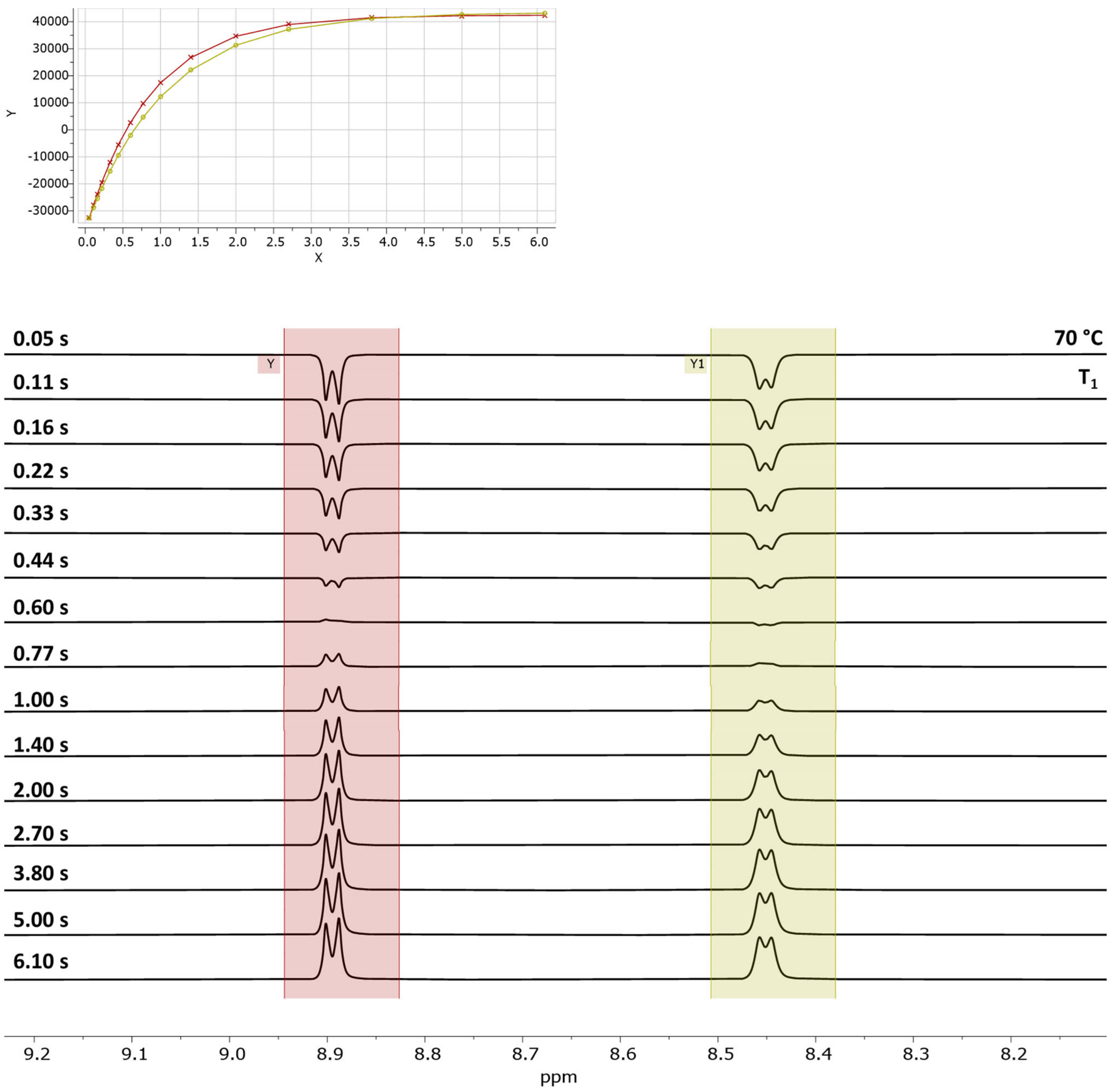

Supplementary Figure 112. T_1 (inverse recovery). Stacked spectra of the inverse recovery experiment to determine the T_1 values of the ortho (Y) and meta (Y1) protons of **MnRot** (0.5 mM) and **V1** (5 mM) in solution, in which the signal intensity (Y) is plotted against the time in seconds (X) (^{1}H, 500 MHz, chloroform-*d* : acetonitrile-d_3, 1:1, v/v, 343 K).

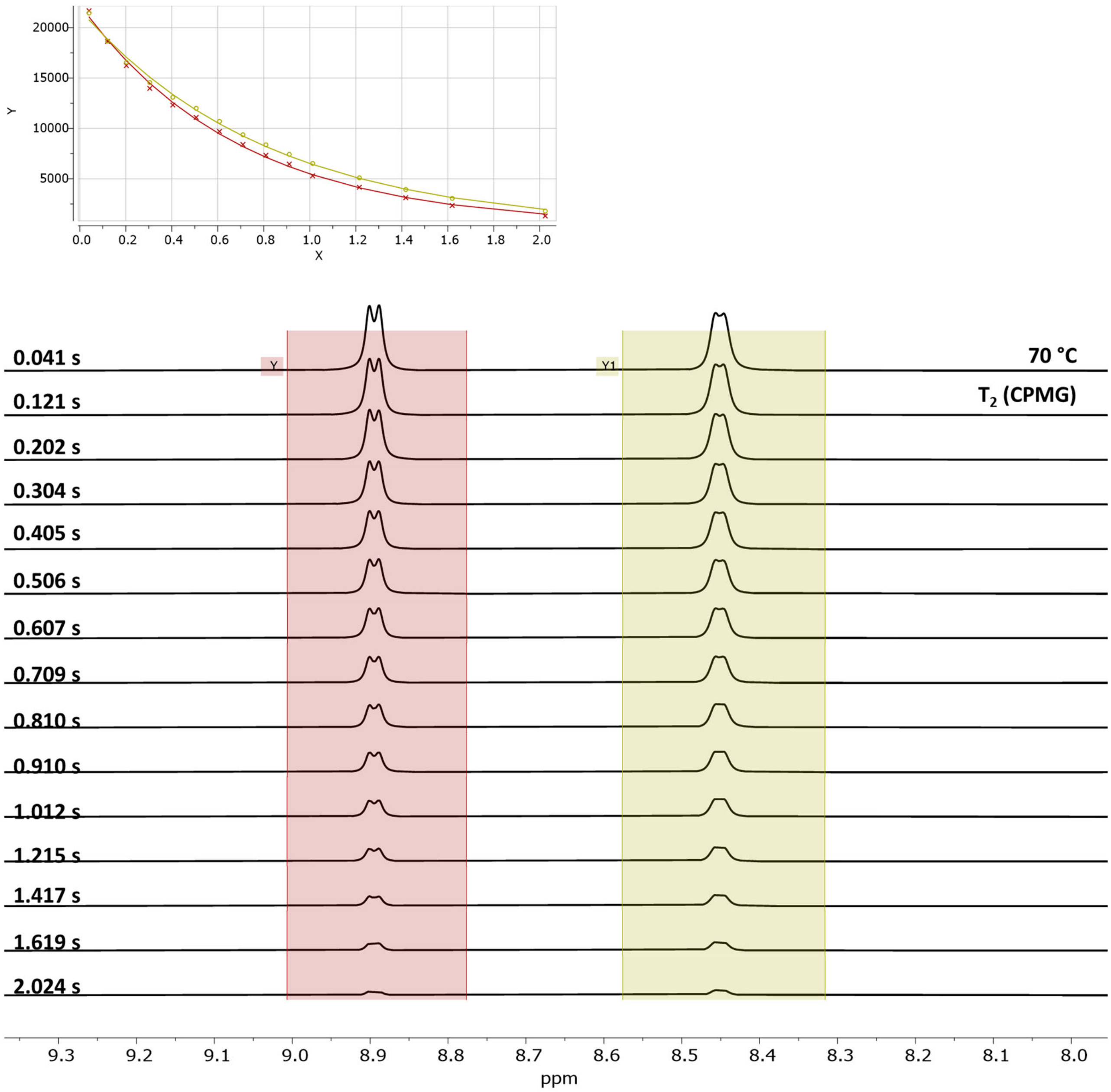

Supplementary Figure 113. T_2 (CPMG). Stacked spectra of the CPMG experiment to determine the T_2 values of the ortho (Y) and meta (Y1) protons of **MnRot** (0.5 mM) and **V1** (5 mM) in solution, in which the signal intensity (Y) is plotted against the time in seconds (X) (^{1}H, 500 MHz, chloroform-*d* : acetonitrile-*d*$_3$, 1:1, v/v, 343 K).

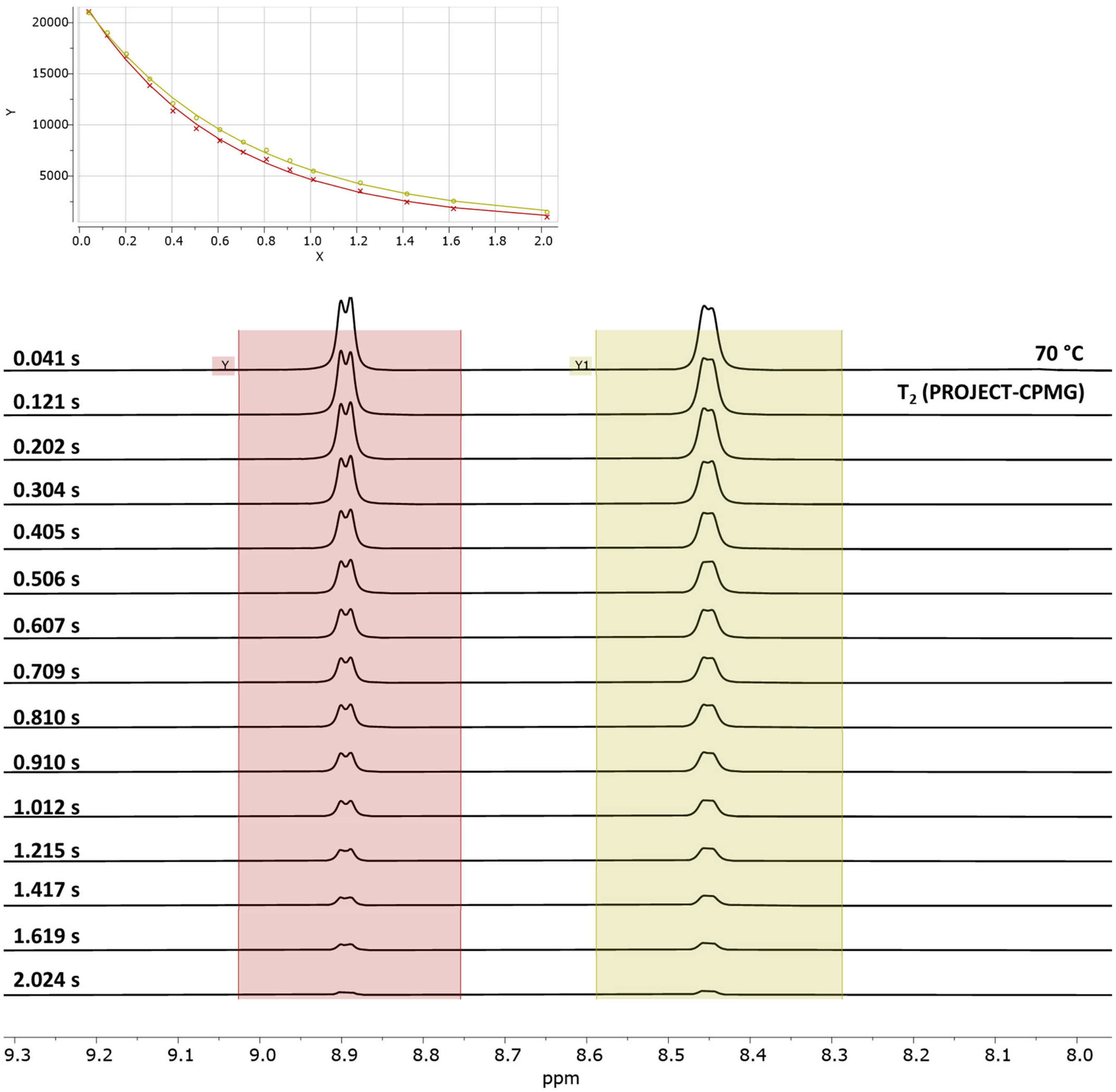

Supplementary Figure 114. T_2 (PROJECT-CPMG). Stacked spectra of the PROJECT-CPMG experiment to determine the T_2 values of the ortho (Y) and meta (Y1) protons of **MnRot** (0.5 mM) and **V1** (5 mM) in solution, in which the signal intensity (Y) is plotted against the time in seconds (X) (^{1}H, 500 MHz, chloroform-*d* : acetonitrile-*d*$_3$, 343 K).

4.4. VP ($T_{1,0}$ and $T_{2,0}$)

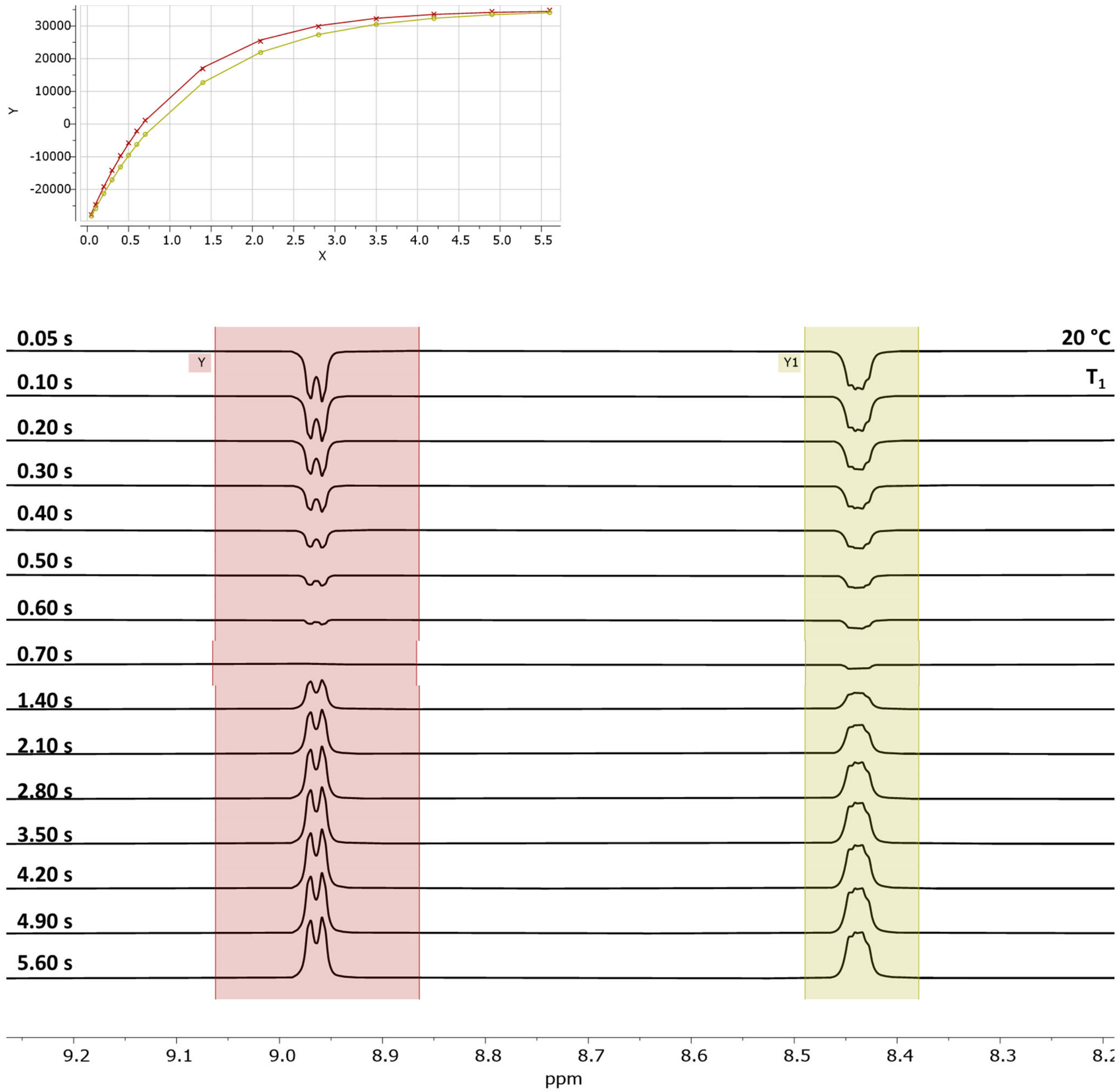

Supplementary Figure 115. T_1 (inverse recovery). Stacked spectra of the inverse recovery experiment to determine the T_1 values of the ortho (Y) and meta (Y1) protons of **VP** in solution, in which the signal intensity (Y) is plotted against the time in seconds (X) (^{1}H, 500 MHz, chloroform-*d* : acetonitrile-*d$_3$*, 293 K).

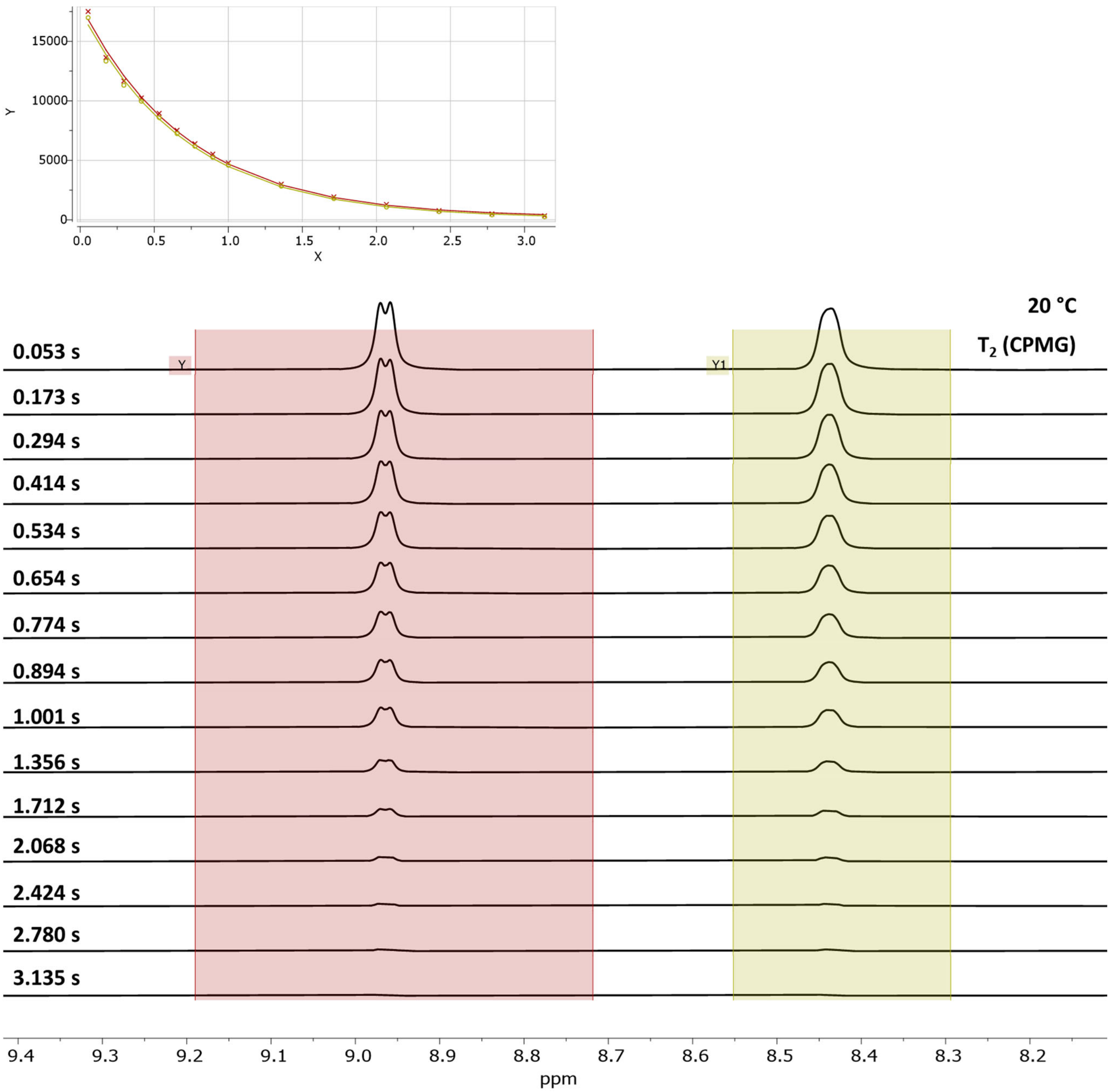

Supplementary Figure 116. T_2 (CPMG). Stacked spectra of the CPMG experiment to determine the T_2 values of the ortho (Y) and meta (Y1) protons of **VP** in solution, in which the signal intensity (Y) is plotted against the time in seconds (X) (^{1}H, 500 MHz, chloroform-*d* : acetonitrile-*d*$_3$, 293 K).

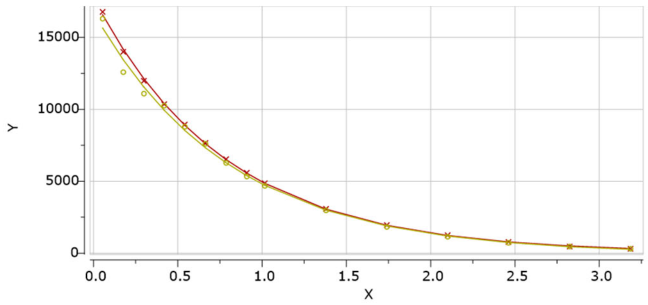

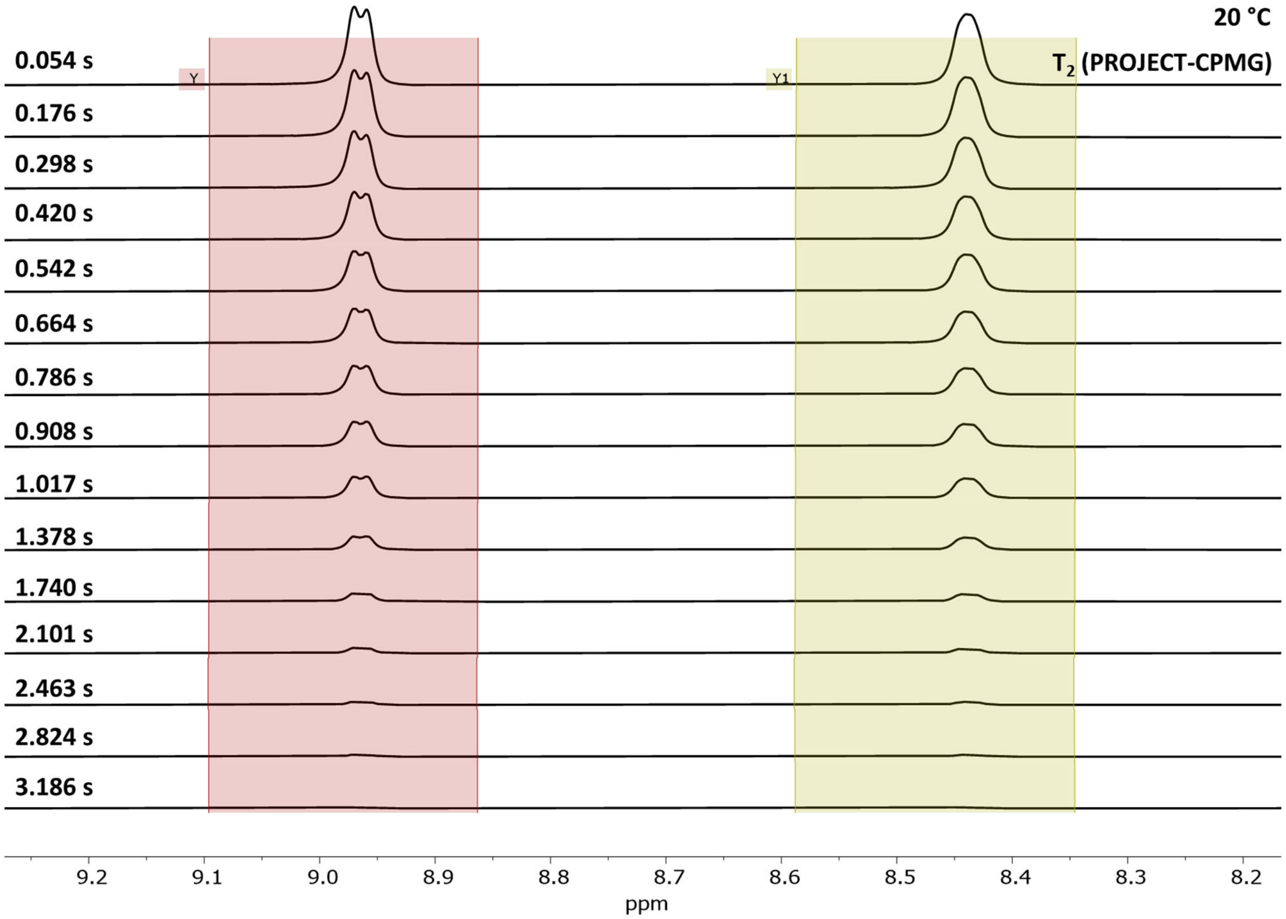

Supplementary Figure 117. T_2 (PROJECT-CPMG). Stacked spectra of the PROJECT-CPMG experiment to determine the T_2 values of the ortho (Y) and meta (Y1) protons of **VP** in solution, in which the signal intensity (Y) is plotted against the time in seconds (X) (^{1}H, 500 MHz, chloroform-*d* : acetonitrile-d_3, 293 K).

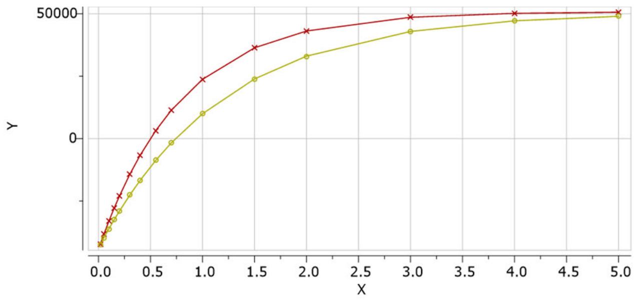

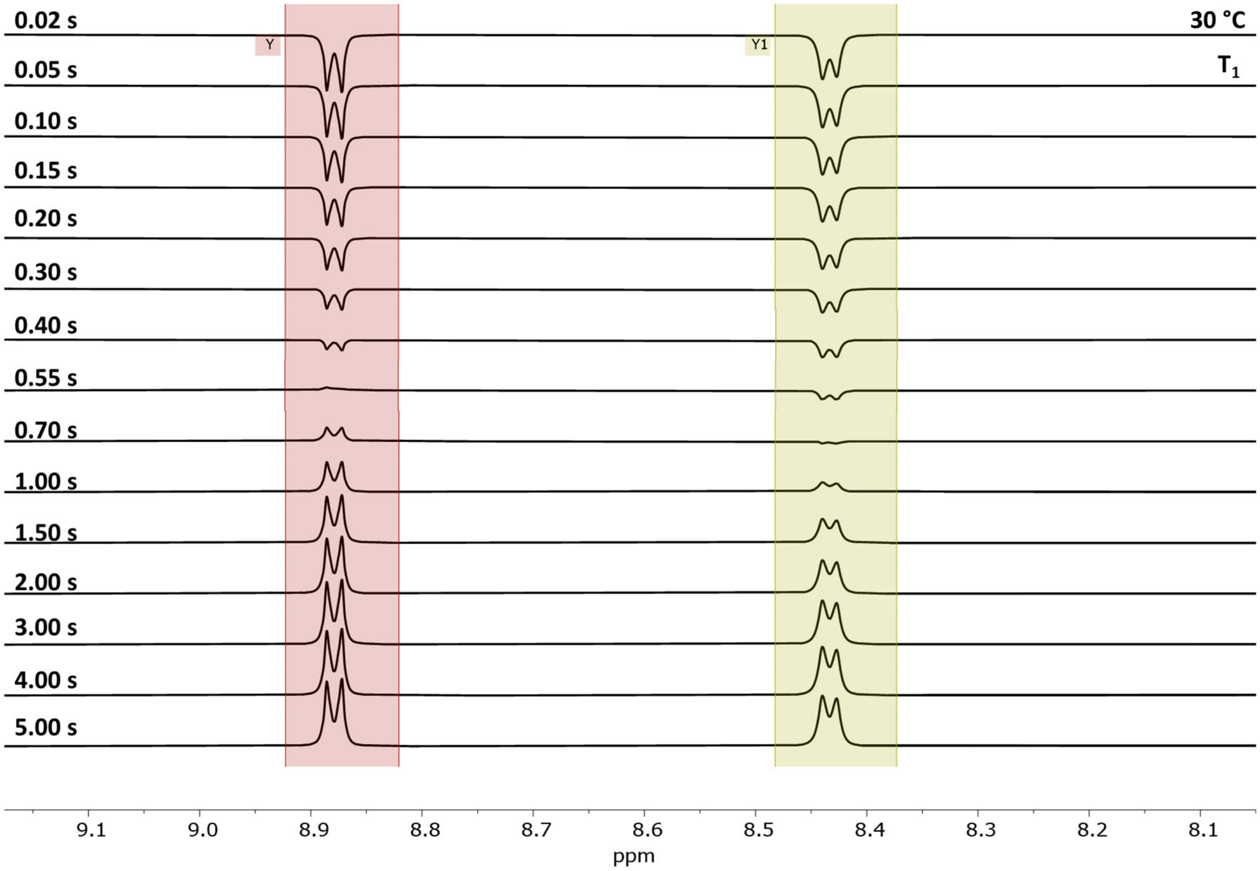

Supplementary Figure 118. T_1 (inverse recovery). Stacked spectra of the inverse recovery experiment to determine the T_1 values of the ortho (Y) and meta (Y1) protons of **VP** in solution, in which the signal intensity (Y) is plotted against the time in seconds (X) (^{1}H, 500 MHz, chloroform-*d* : acetonitrile-*d*$_3$, 303 K).

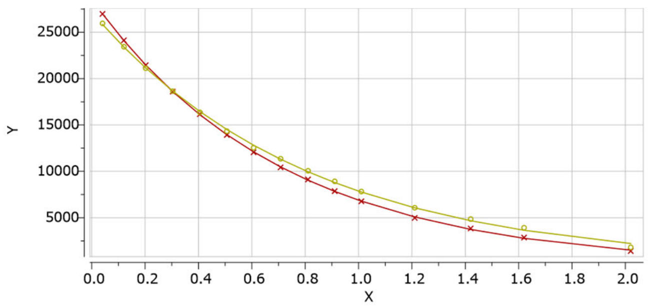

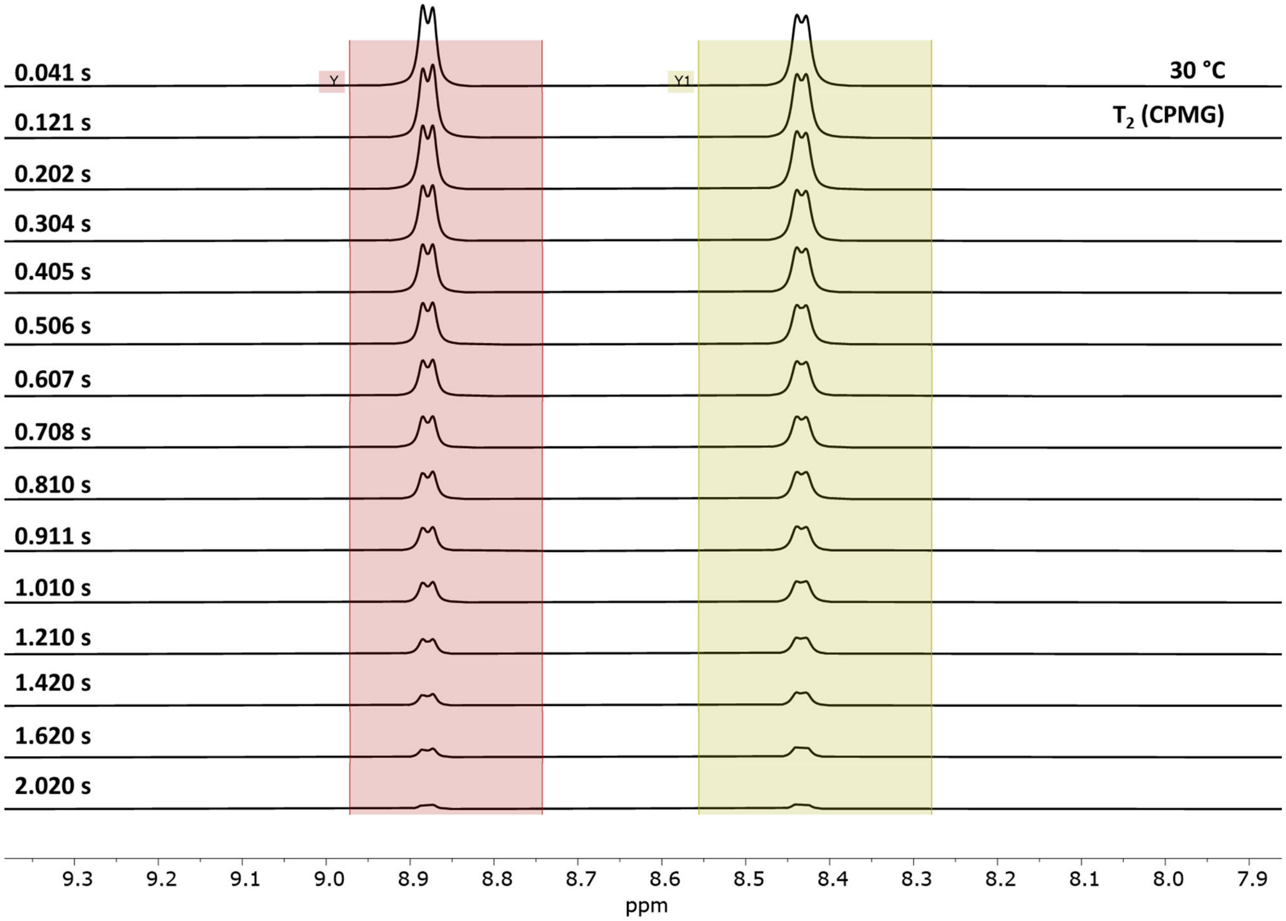

Supplementary Figure 119. T_2 (CPMG). Stacked spectra of the CPMG experiment to determine the T_2 values of the ortho (Y) and meta (Y1) protons of **VP** in solution, in which the signal intensity (Y) is plotted against the time in seconds (X) (^1H, 500 MHz, chloroform-*d* : acetonitrile-*d*$_3$, 303 K).

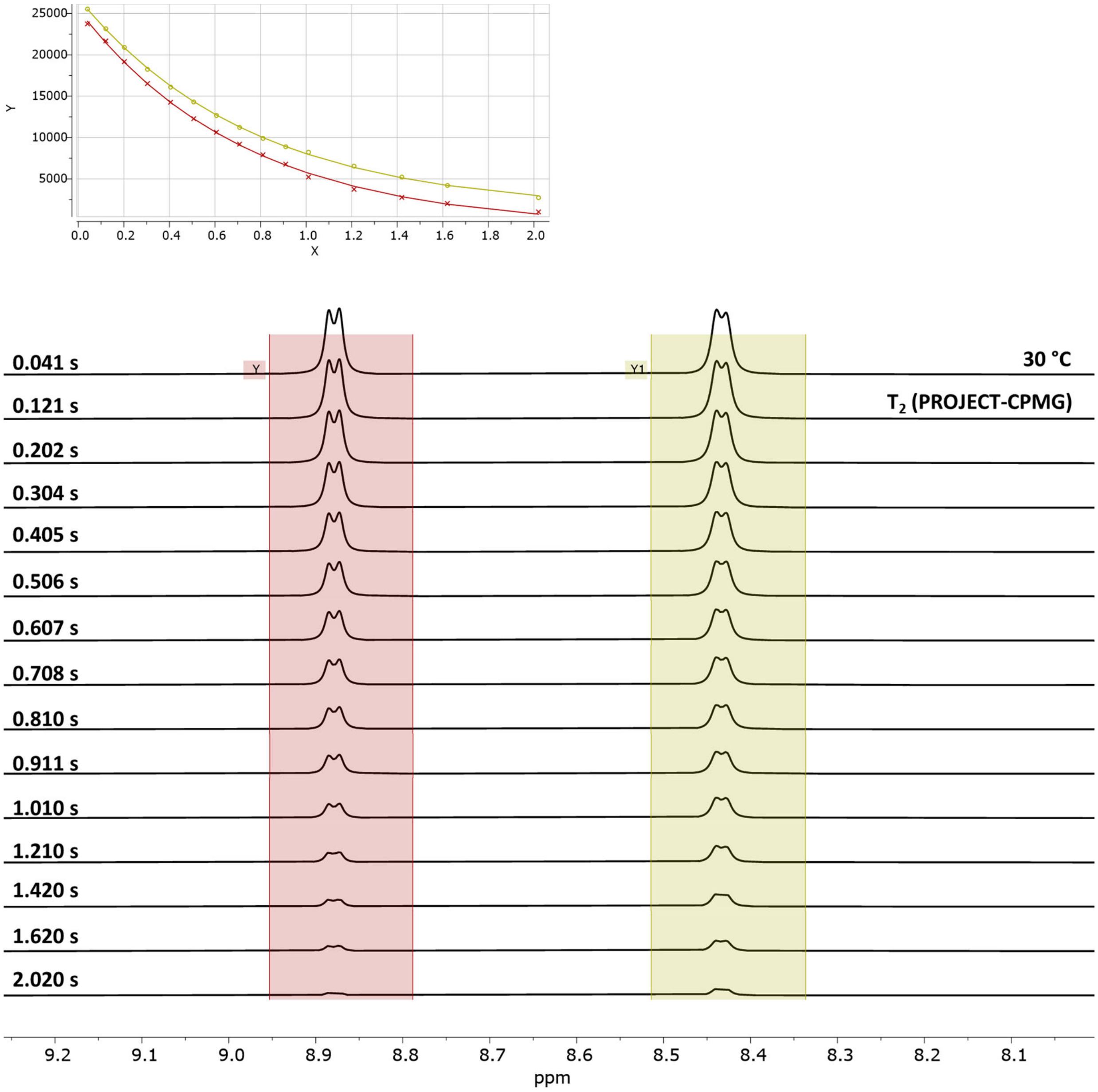

Supplementary Figure 120. T_2 (PROJECT-CPMG). Stacked spectra of the PROJECT-CPMG experiment to determine the T_2 values of the ortho (Y) and meta (Y1) protons of **VP** in solution, in which the signal intensity (Y) is plotted against the time in seconds (X) (^{1}H, 500 MHz, chloroform-*d* : acetonitrile-*d*$_3$, 303 K).

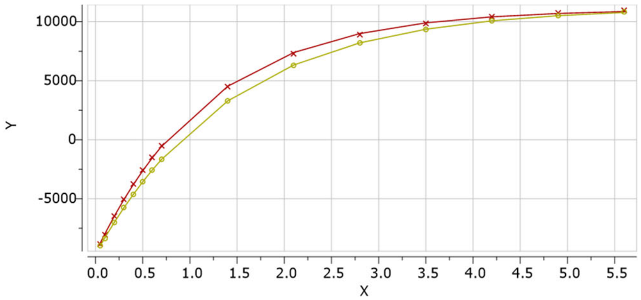

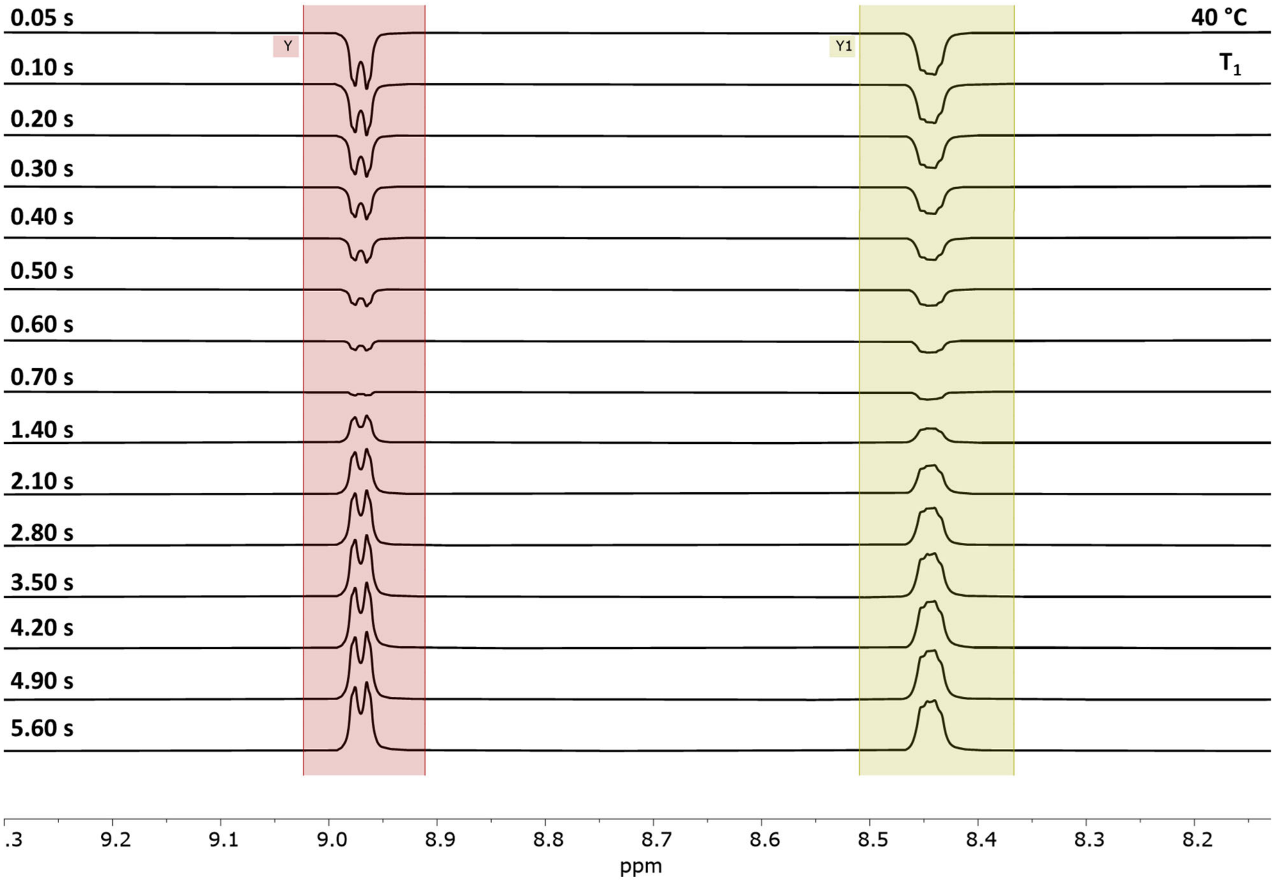

Supplementary Figure 121. T_1 (inverse recovery). Stacked spectra of the inverse recovery experiment to determine the T_1 values of the ortho (Y) and meta (Y1) protons of **VP** in solution, in which the signal intensity (Y) is plotted against the time in seconds (X) (^1H, 500 MHz, chloroform-*d* : acetonitrile-*d*$_3$, 313 K).

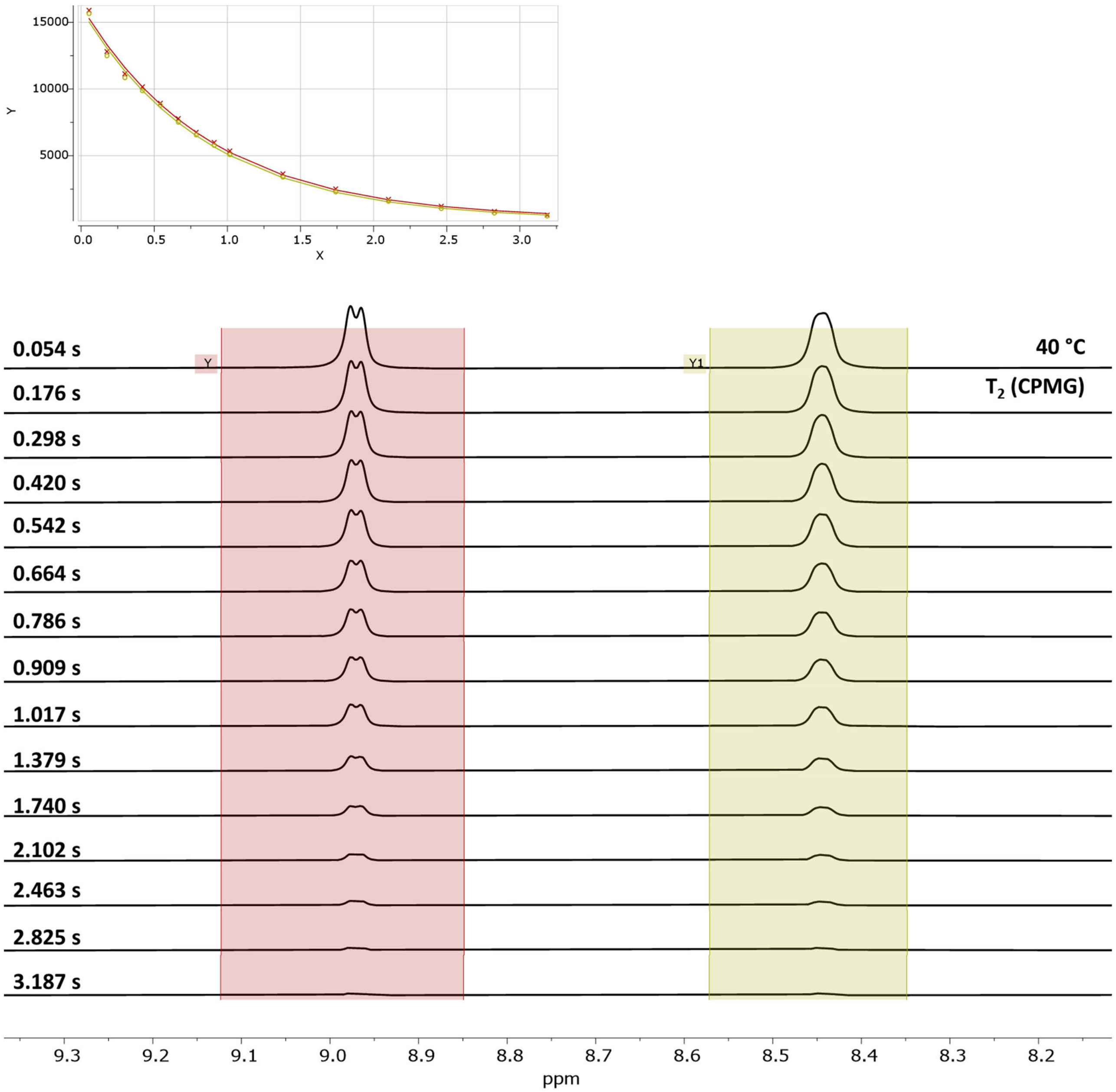

Supplementary Figure 122. T_2 (CPMG). Stacked spectra of the CPMG experiment to determine the T_2 values of the ortho (Y) and meta (Y1) protons of **VP** in solution, in which the signal intensity (Y) is plotted against the time in seconds (X) (^{1}H, 500 MHz, chloroform-*d* : acetonitrile-*d$_3$*, 313 K).

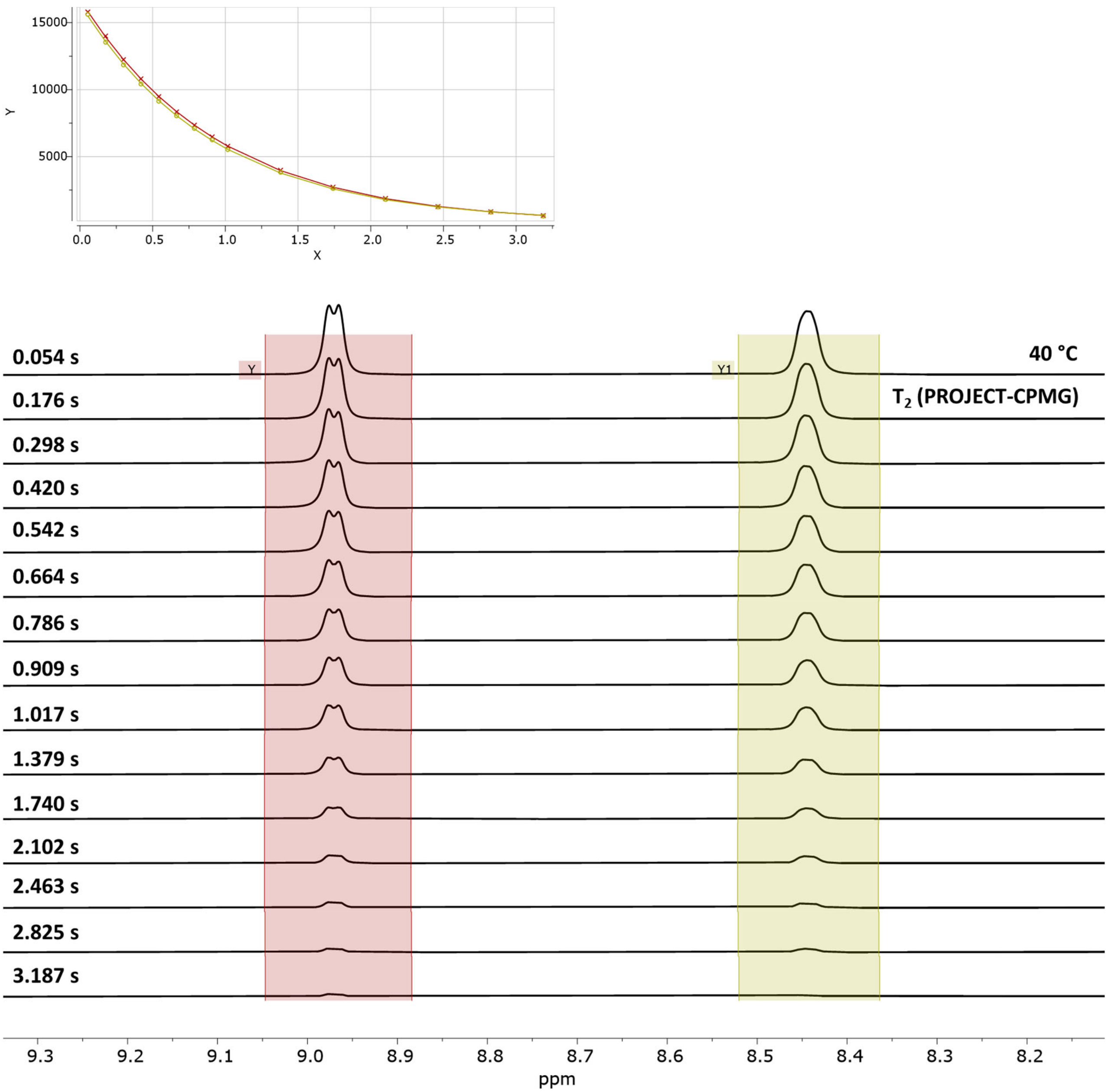

Supplementary Figure 123. T_2 (PROJECT-CPMG). Stacked spectra of the PROJECT-CPMG experiment to determine the T_2 values of the ortho (Y) and meta (Y1) protons of **VP** in solution, in which the signal intensity (Y) is plotted against the time in seconds (X) (^{1}H, 500 MHz, chloroform-*d* : acetonitrile-*d*$_3$, 313 K).

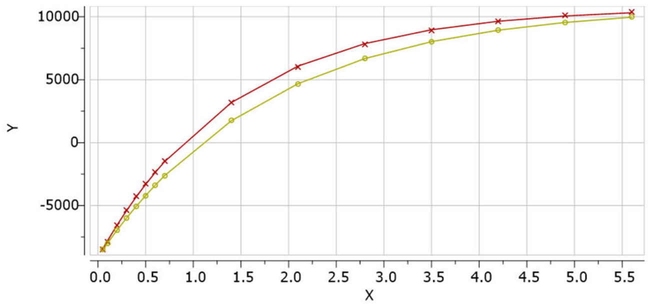

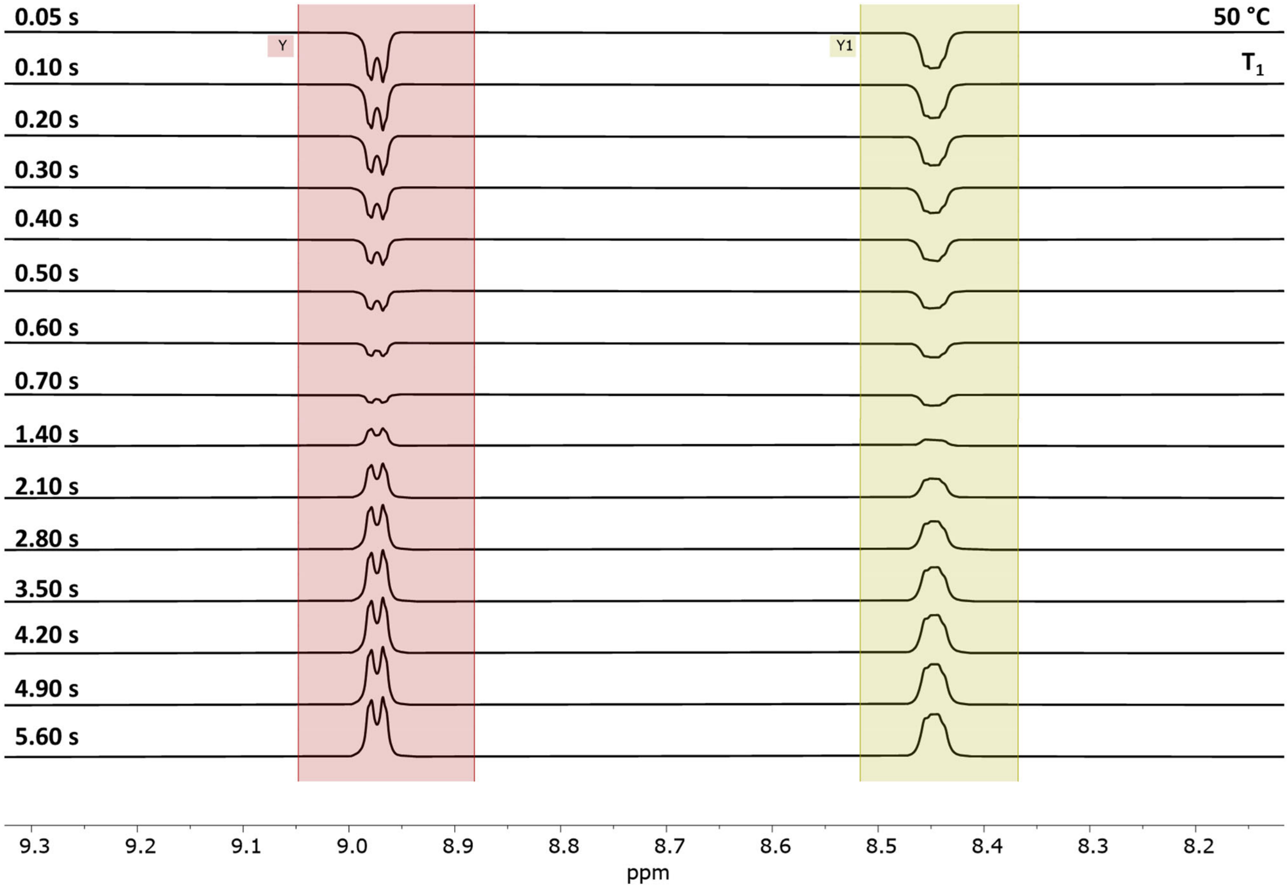

Supplementary Figure 124. T_1 (inverse recovery). Stacked spectra of the inverse recovery experiment to determine the T_1 values of the ortho (Y) and meta (Y1) protons of **VP** in solution, in which the signal intensity (Y) is plotted against the time in seconds (X) (^{1}H, 500 MHz, chloroform-*d* : acetonitrile-*d*$_3$, 323 K).

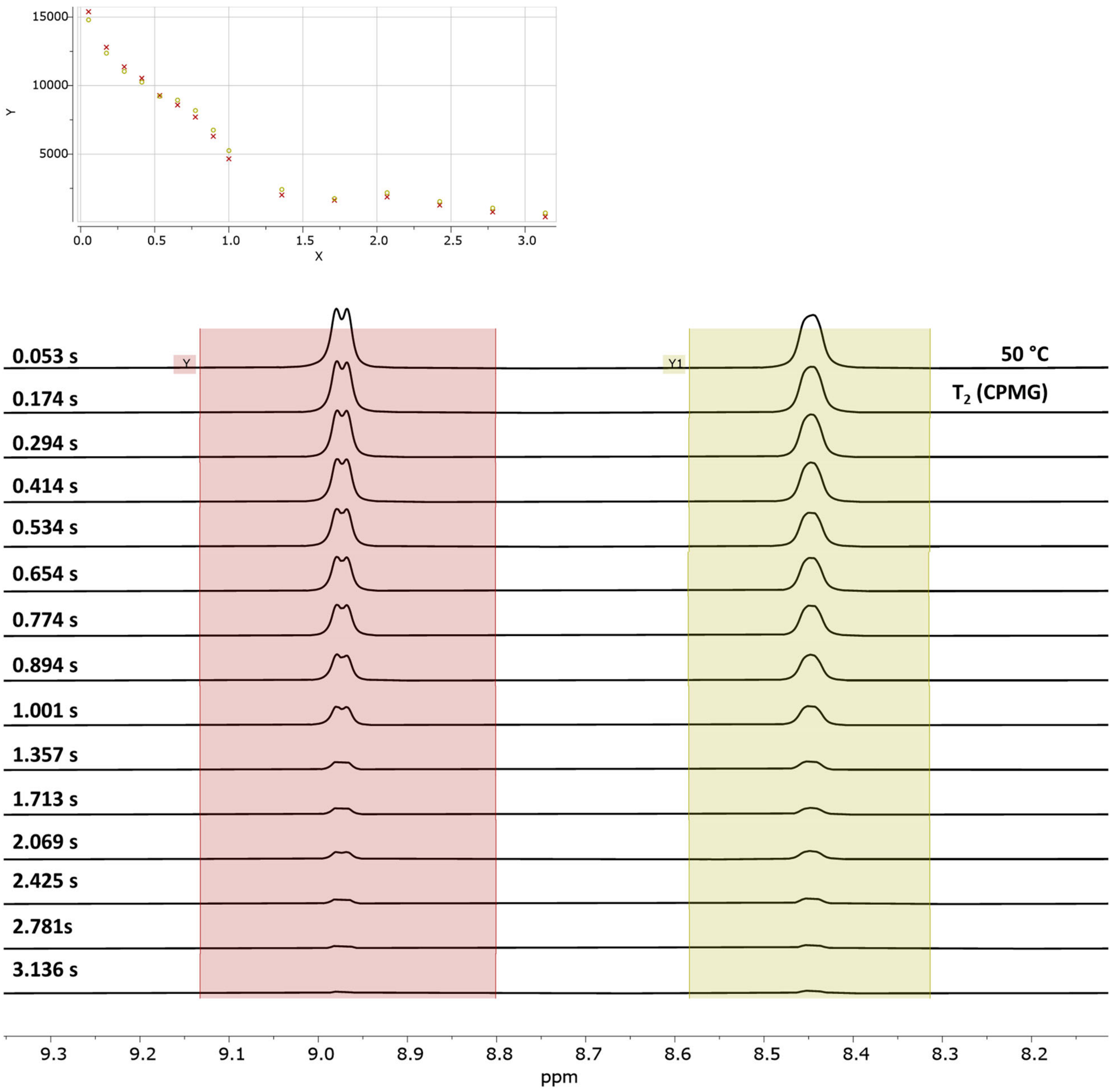

Supplementary Figure 125. T_2 (CPMG). Stacked spectra of the CPMG experiment to determine the T_2 values of the ortho (Y) and meta (Y1) protons of **VP** in solution, in which the signal intensity (Y) is plotted against the time in seconds (X) (^1H, 500 MHz, chloroform-*d* : acetonitrile-*d*$_3$, 323 K).

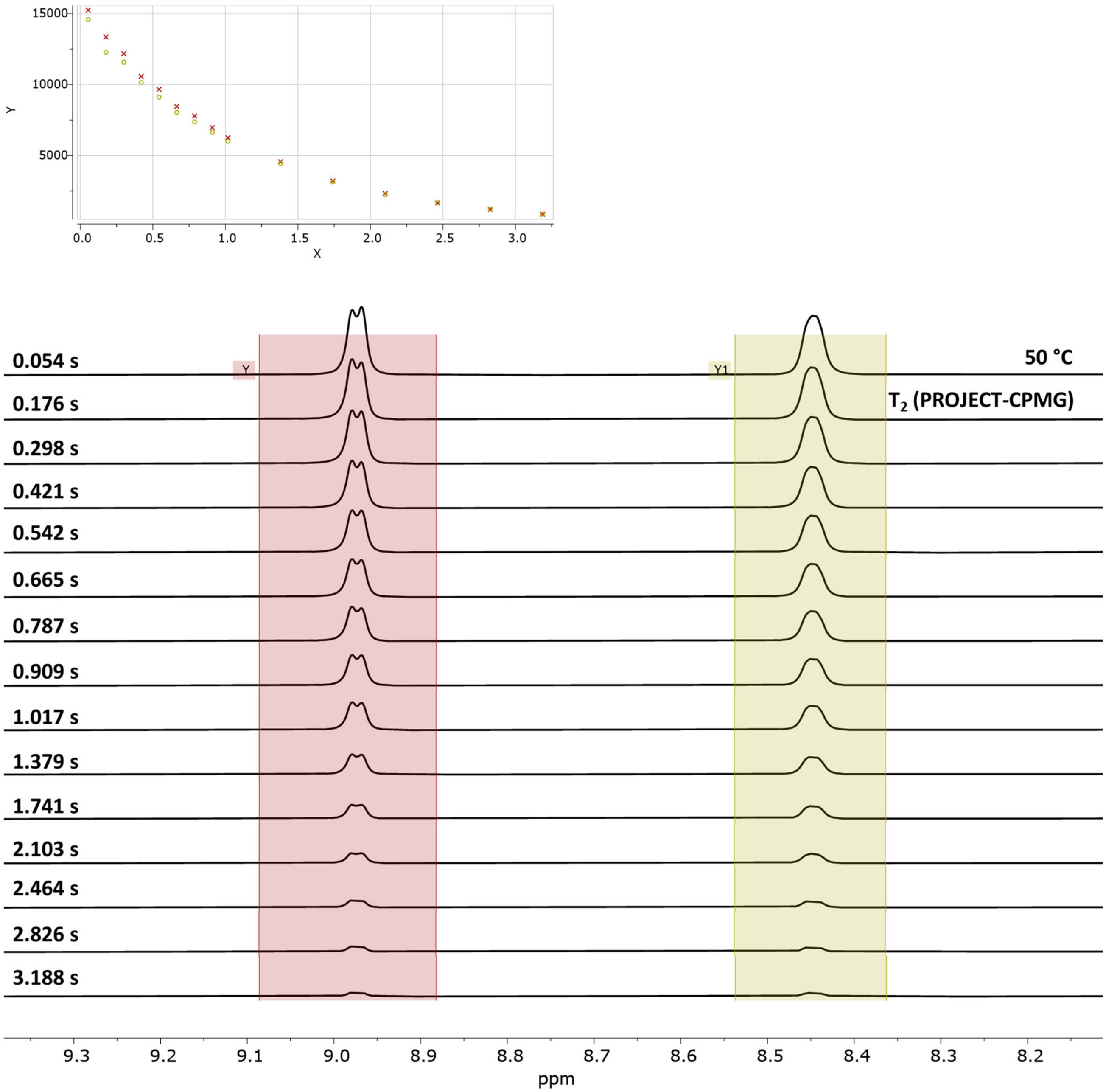

Supplementary Figure 126. T_2 (PROJECT-CPMG). Stacked spectra of the PROJECT-CPMG experiment to determine the T_2 values of the ortho (Y) and meta (Y1) protons of **VP** in solution, in which the signal intensity (Y) is plotted against the time in seconds (X) (^{1}H, 500 MHz, chloroform-*d* : acetonitrile-*d$_3$*, 323 K).

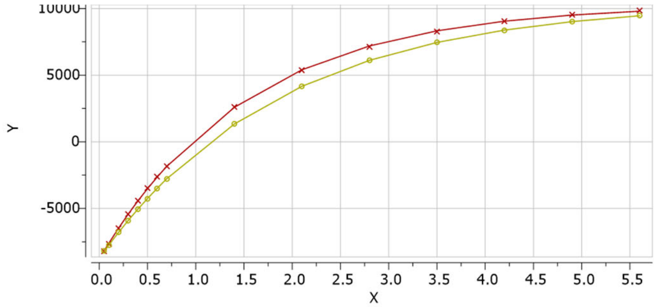

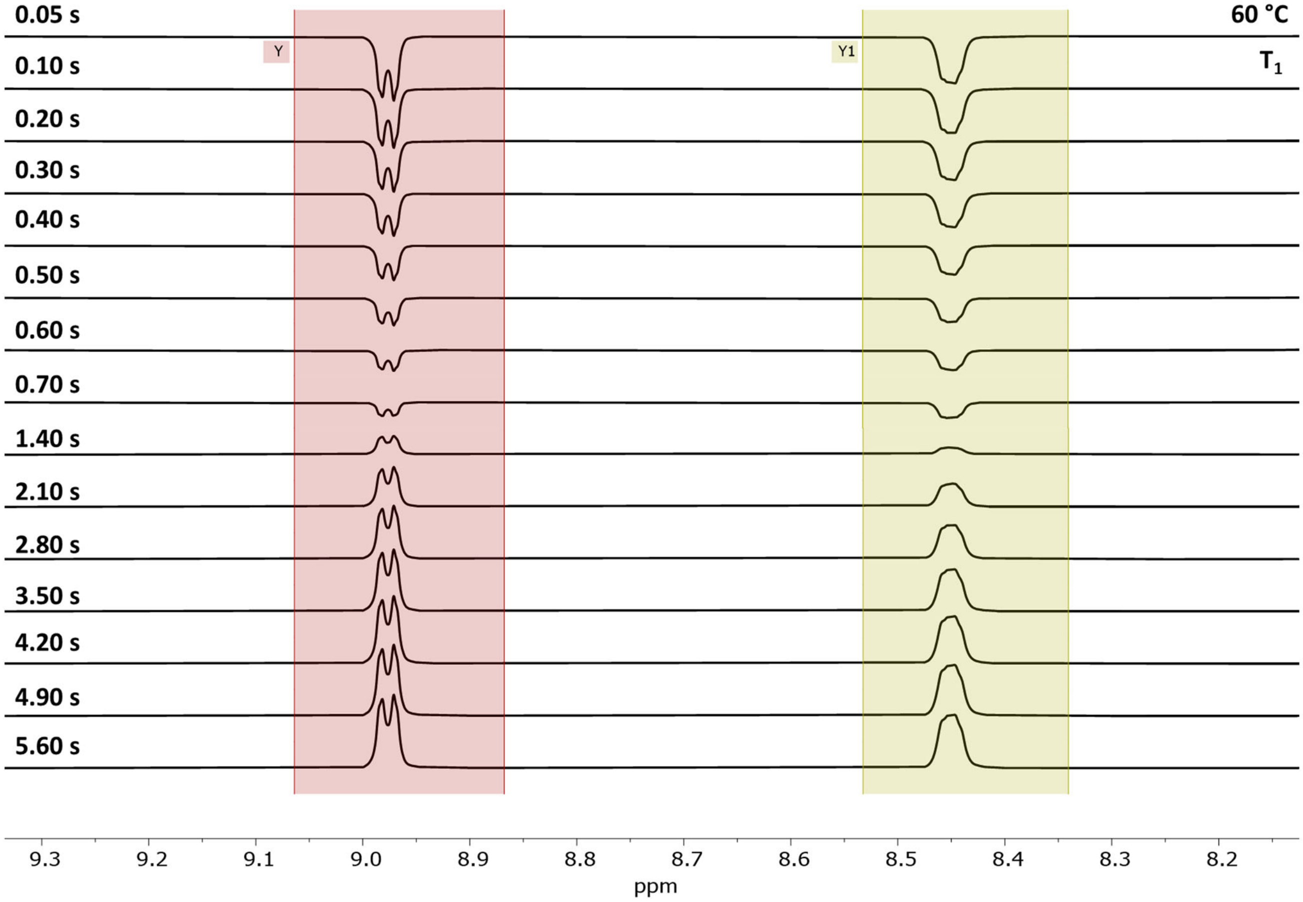

Supplementary Figure 127. T_1 (inverse recovery). Stacked spectra of the inverse recovery experiment to determine the T_1 values of the ortho (Y) and meta (Y1) protons of **VP** in solution, in which the signal intensity (Y) is plotted against the time in seconds (X) (^{1}H, 500 MHz, chloroform-*d* : acetonitrile-*d$_3$*, 333 K).

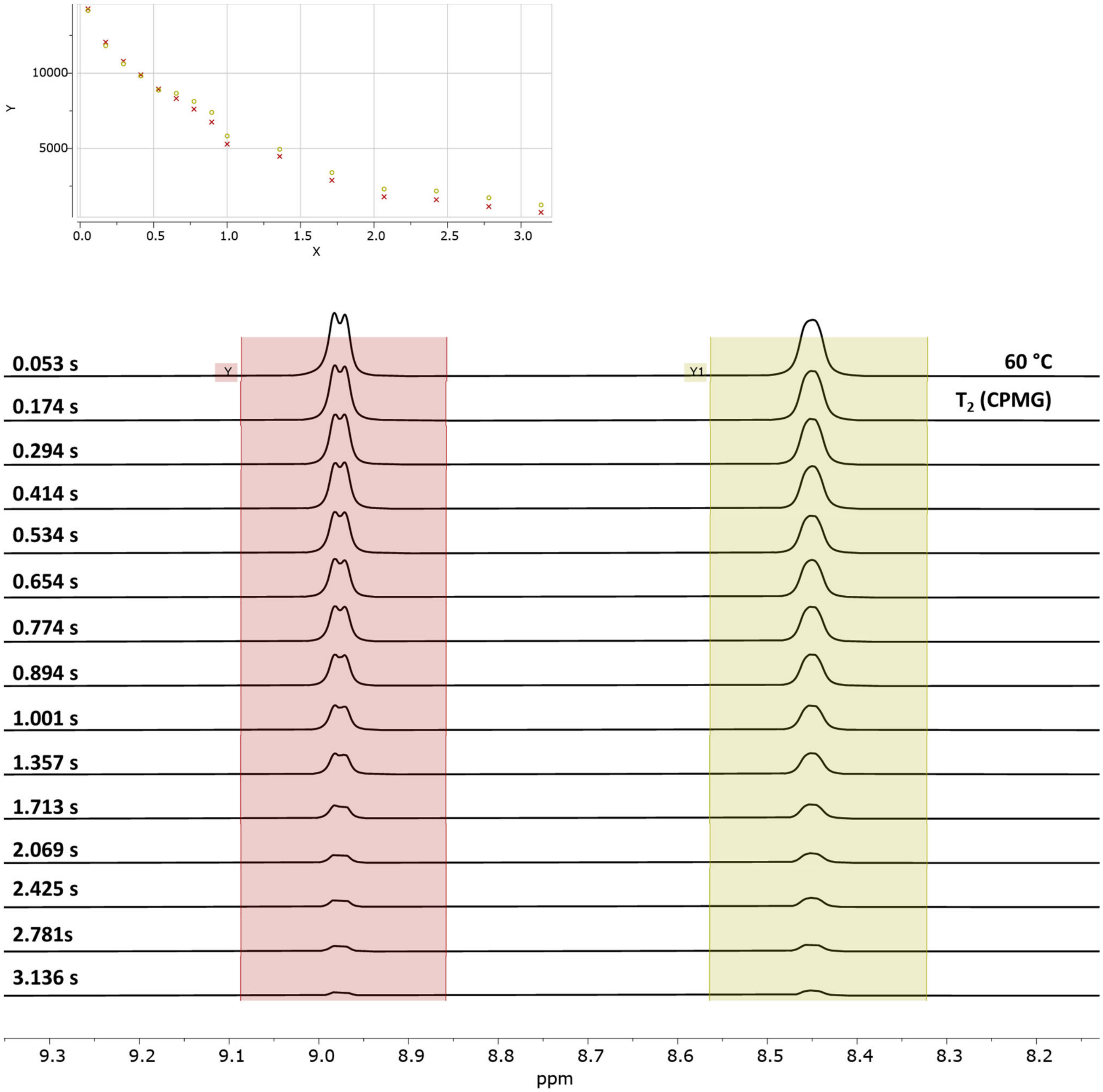

Supplementary Figure 128. T_2 (CPMG). Stacked spectra of the CPMG experiment to determine the T_2 values of the ortho (Y) and meta (Y1) protons of **VP** in solution, in which the signal intensity (Y) is plotted against the time in seconds (X) (^{1}H, 500 MHz, chloroform-*d* : acetonitrile-*d*$_3$, 333 K).

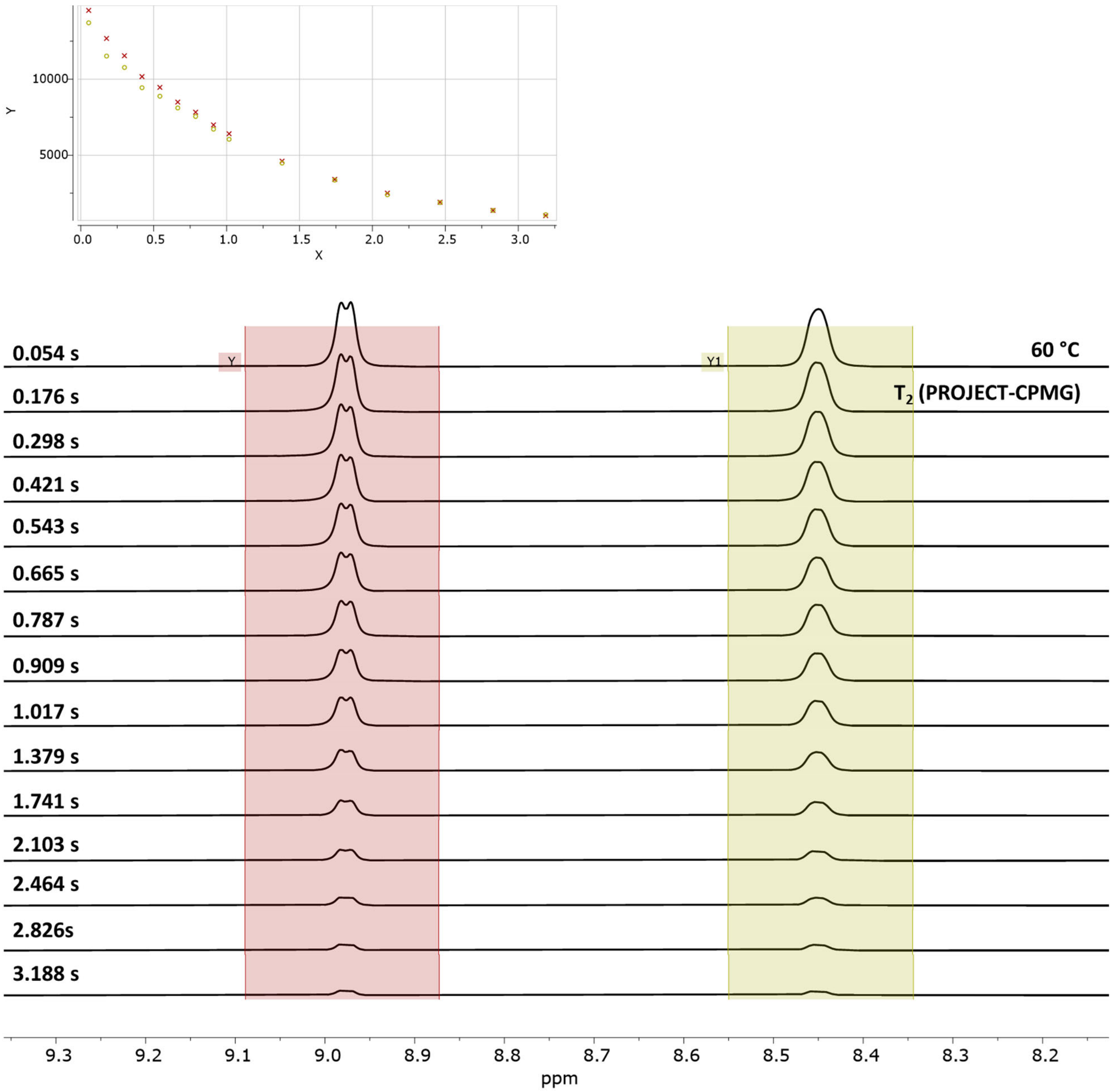

Supplementary Figure 129. T_2 (PROJECT-CPMG). Stacked spectra of the PROJECT-CPMG experiment to determine the T_2 values of the ortho (Y) and meta (Y1) protons of **VP** in solution, in which the signal intensity (Y) is plotted against the time in seconds (X) (^{1}H, 500 MHz, chloroform-*d* : acetonitrile-*d*$_3$, 333 K).

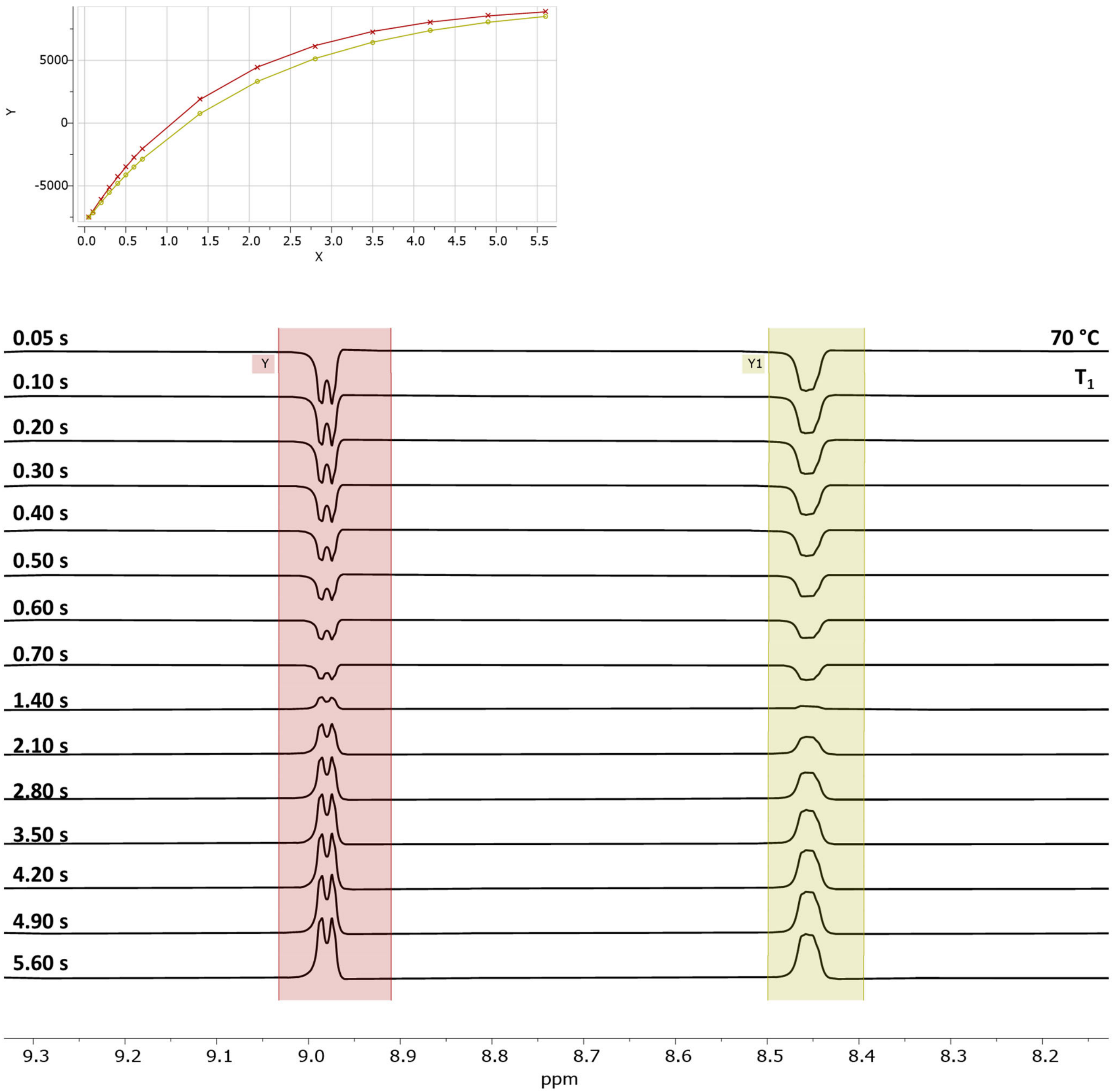

Supplementary Figure 130. T_1 (inverse recovery). Stacked spectra of the inverse recovery experiment to determine the T_1 values of the ortho (Y) and meta (Y1) protons of **VP** in solution, in which the signal intensity (Y) is plotted against the time in seconds (X) (^{1}H, 500 MHz, chloroform-*d* : acetonitrile-d_3, 343 K).

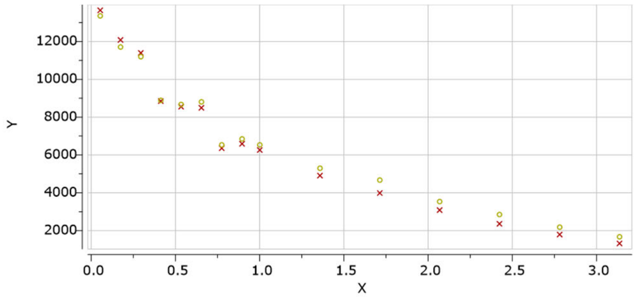

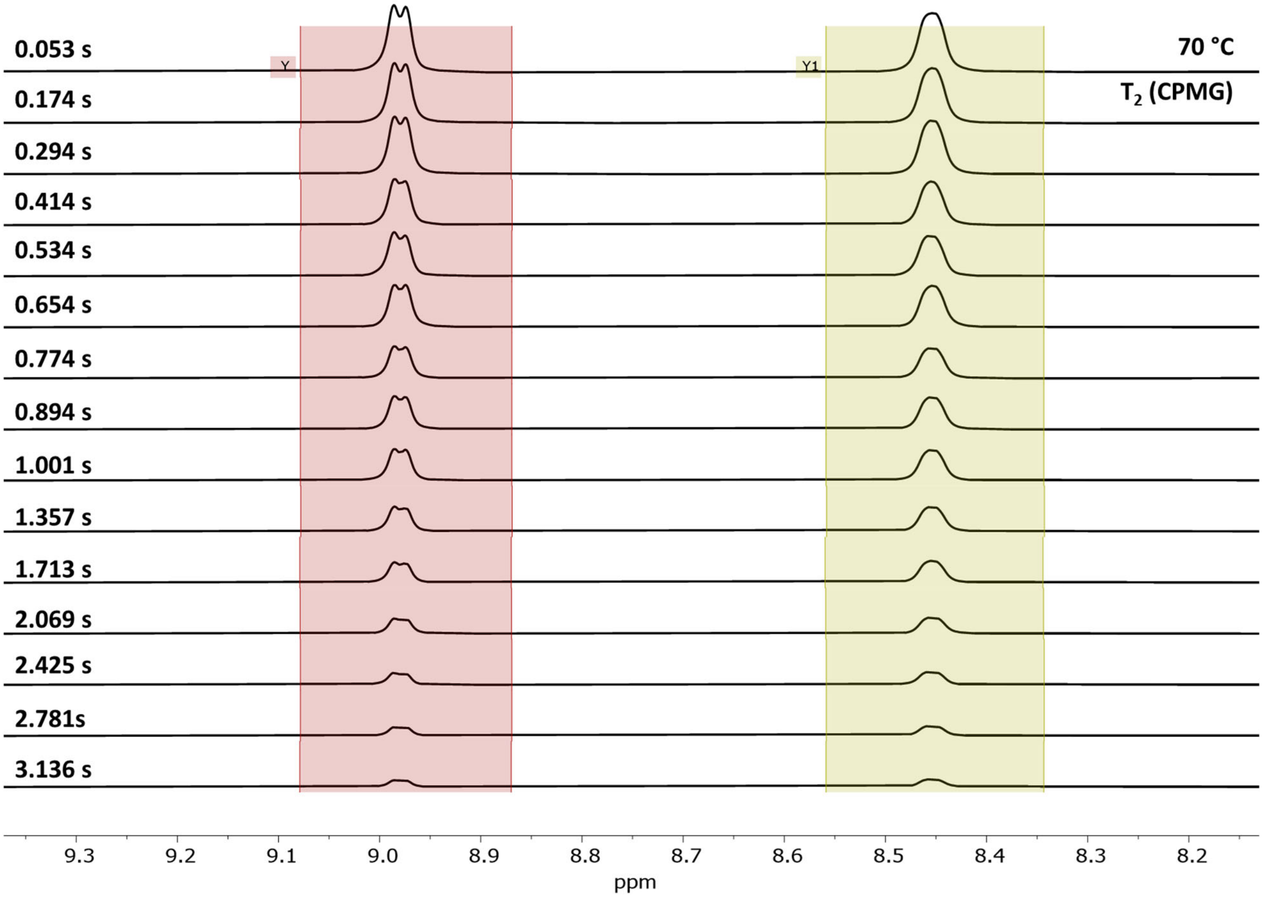

Supplementary Figure 131. T_2 (CPMG). Stacked spectra of the CPMG experiment to determine the T_2 values of the ortho (Y) and meta (Y1) protons of **VP** in solution, in which the signal intensity (Y) is plotted against the time in seconds (X) (^{1}H, 500 MHz, chloroform-*d* : acetonitrile-*d₃*, 343 K).

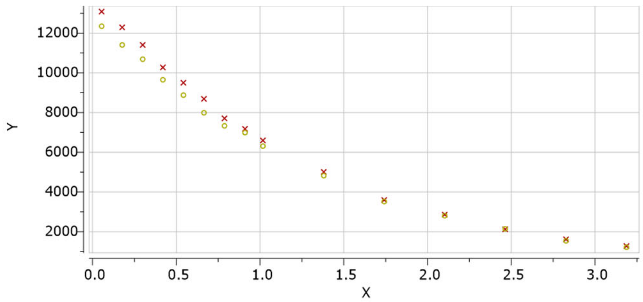

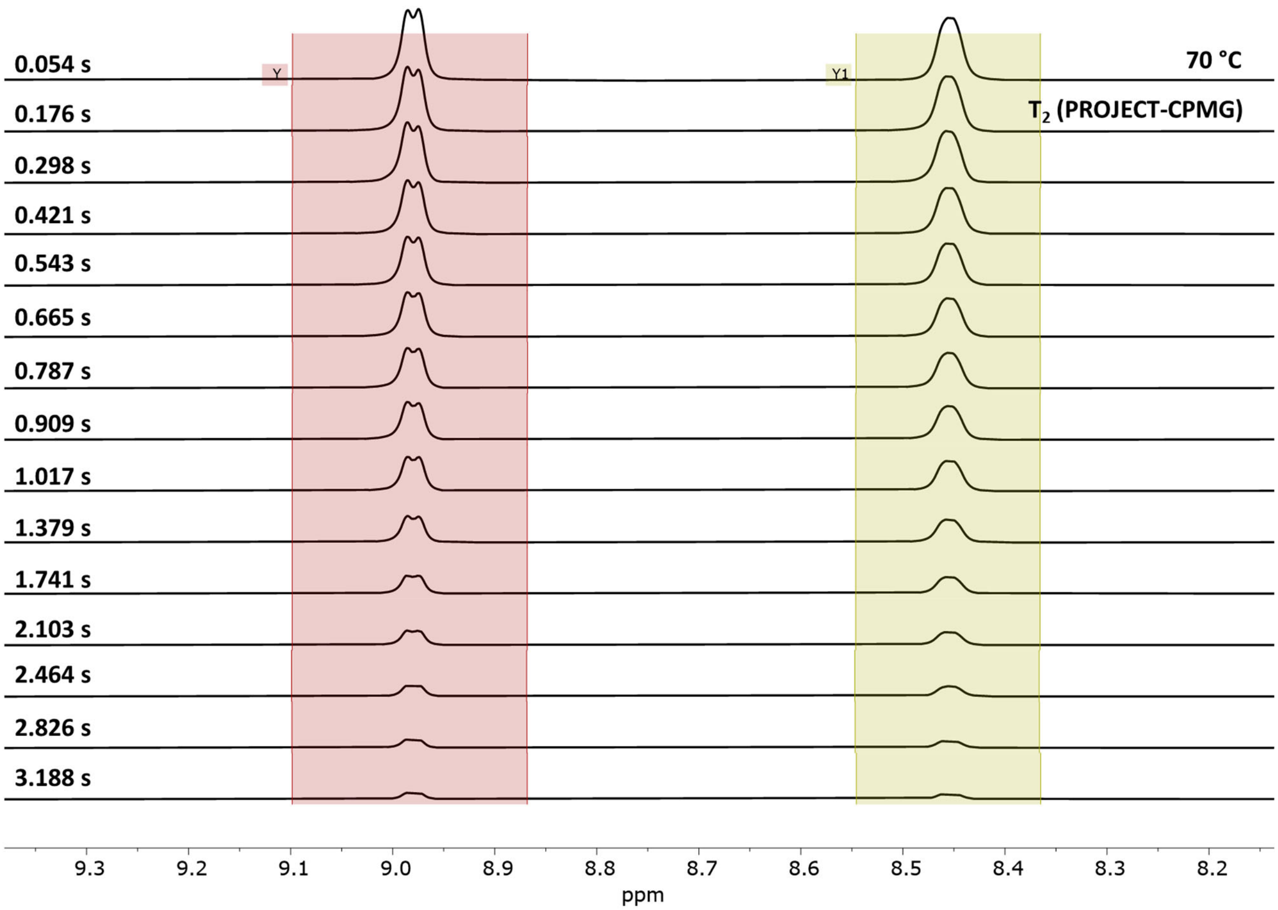

Supplementary Figure 132. T_2 (PROJECT-CPMG). Stacked spectra of the PROJECT-CPMG experiment to determine the T_2 values of the ortho (Y) and meta (Y1) protons of **VP** in solution, in which the signal intensity (Y) is plotted against the time in seconds (X) (^{1}H, 500 MHz, chloroform-*d* : acetonitrile-*d*$_3$, 343 K).

4.5. Mn1/VP ($T_{1,obs}$ and $T_{2,obs}$)

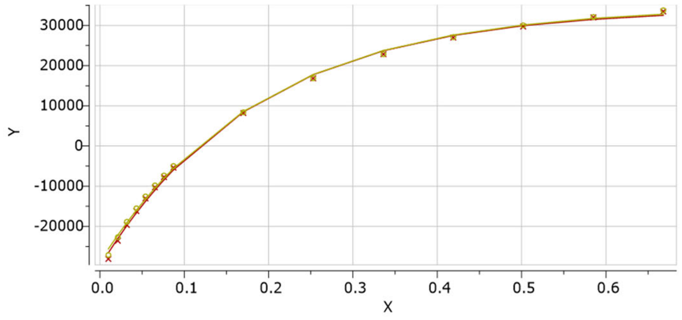

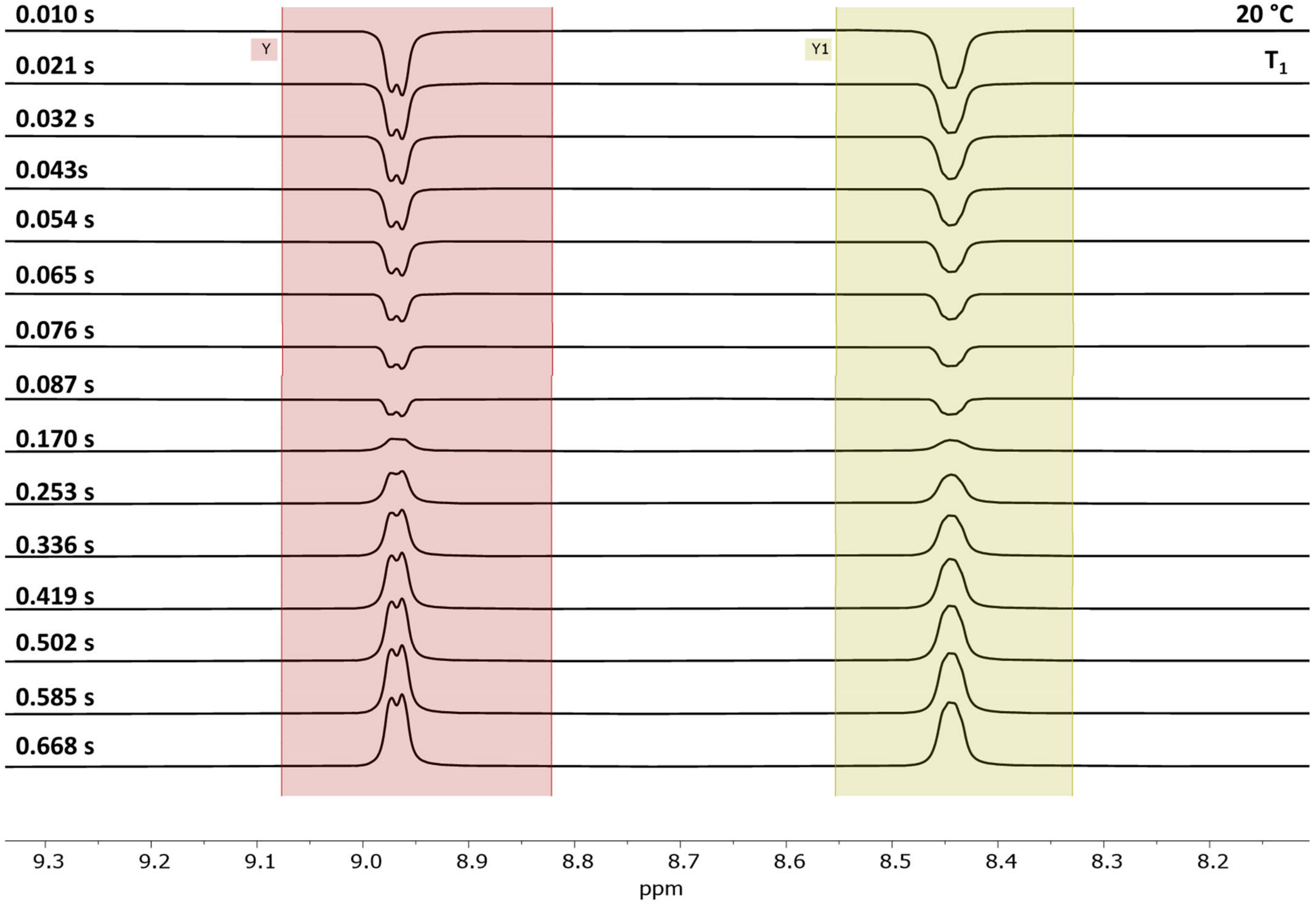

Supplementary Figure 133. T_1 (inverse recovery). Stacked spectra of the inverse recovery experiment to determine the T_1 values of the ortho (Y) and meta (Y1) protons of **Mn1** (0.5 mM) and **VP** (5 mM) in solution, in which the signal intensity (Y) is plotted against the time in seconds (X) (^1H, 500 MHz, chloroform-*d* : acetonitrile-*d*$_3$, 293 K).

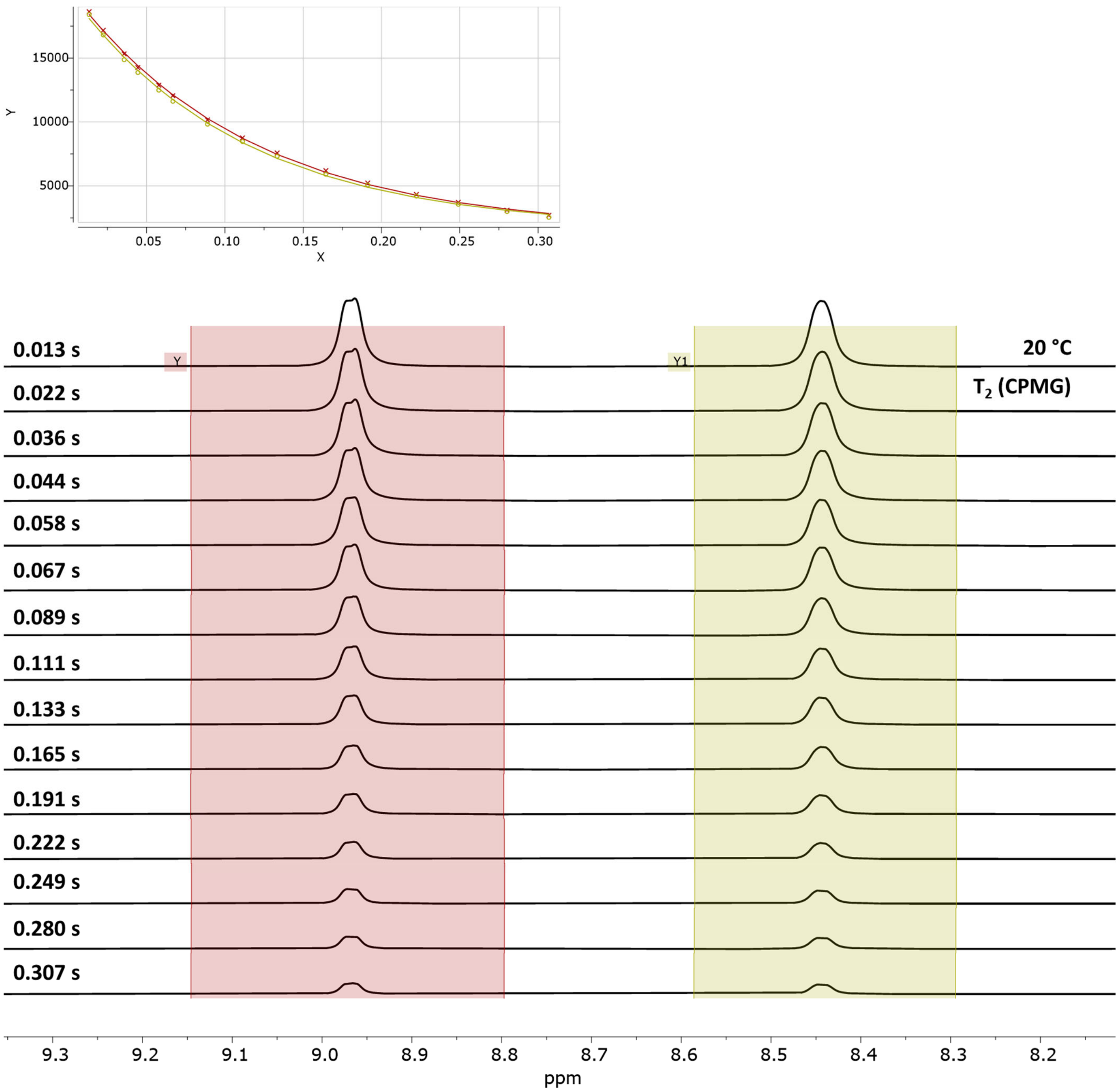

Supplementary Figure 134. T_2 (CPMG). Stacked spectra of the CPMG experiment to determine the T_2 values of the ortho (Y) and meta (Y1) protons of **Mn1** (0.5 mM) and **VP** (5 mM) in solution, in which the signal intensity (Y) is plotted against the time in seconds (X) (^{1}H, 500 MHz, chloroform-*d* : acetonitrile-*d*$_3$, 293 K).

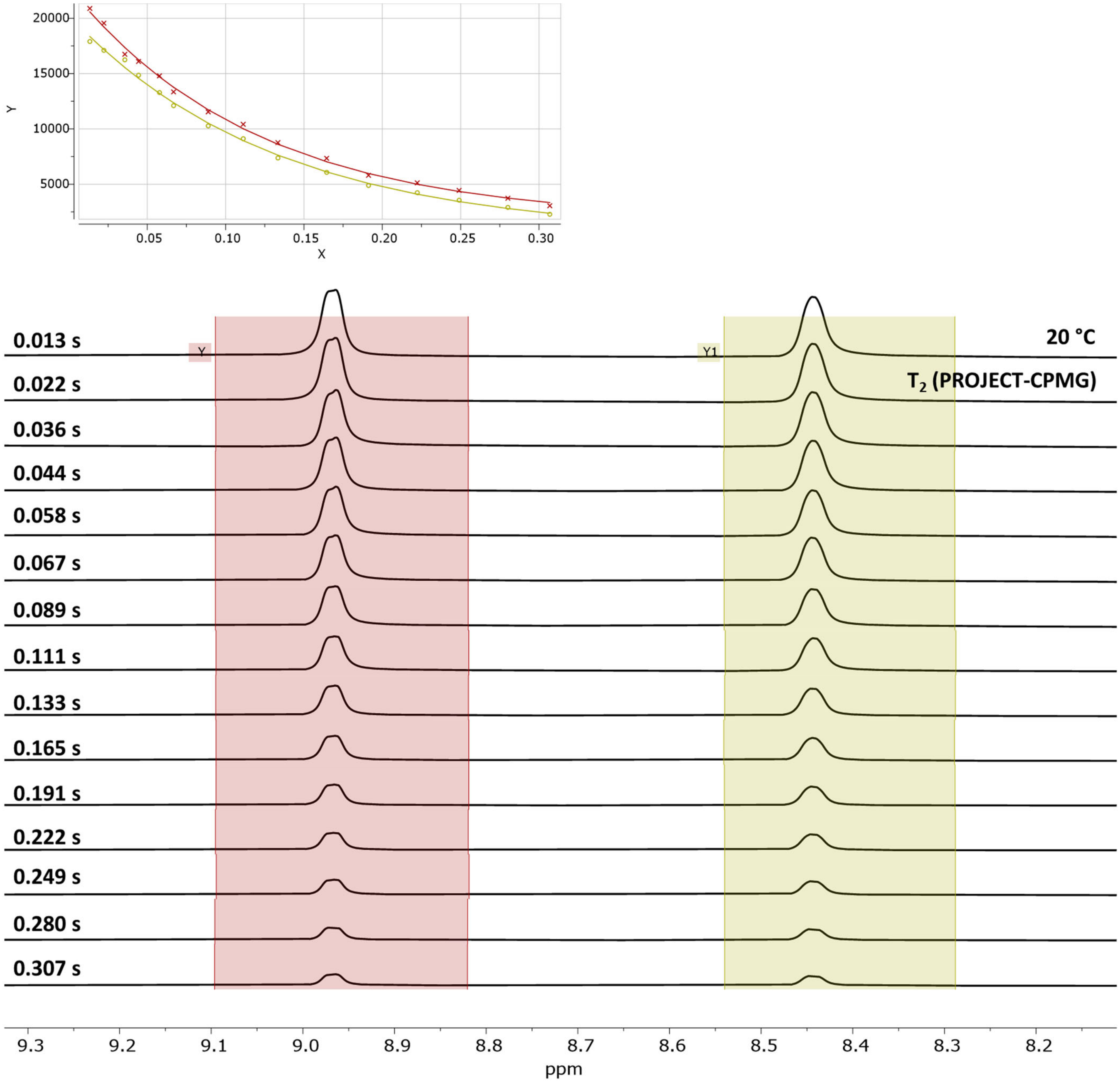

Supplementary Figure 135. T_2 (PROJECT-CPMG). Stacked spectra of the PROJECT-CPMG experiment to determine the T_2 values of the ortho (Y) and meta (Y1) protons of **Mn1** (0.5 mM) and **VP** (5 mM) in solution, in which the signal intensity (Y) is plotted against the time in seconds (X) (^{1}H, 500 MHz, chloroform-*d* : acetonitrile-*d*$_3$, 293 K).

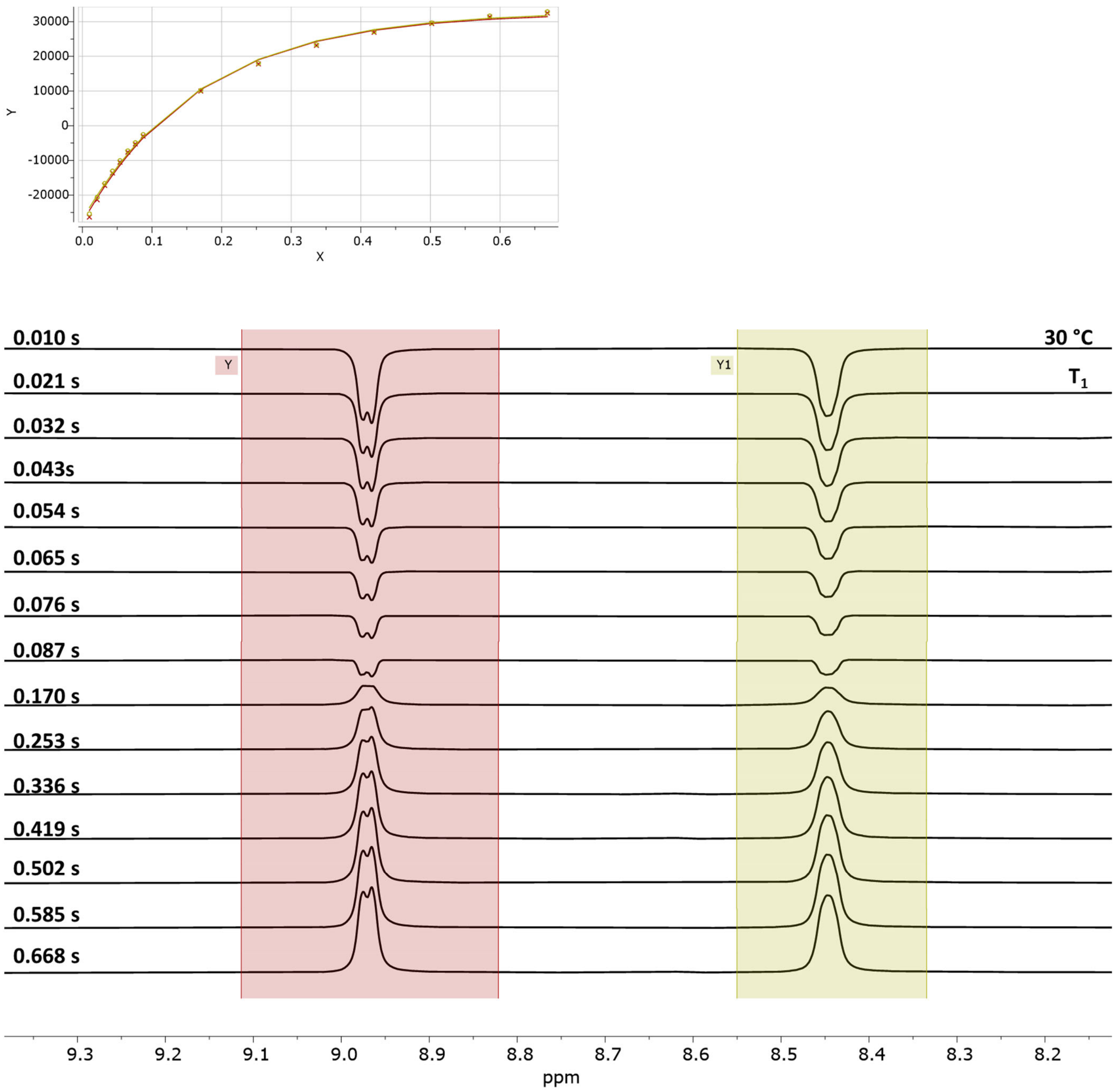

Supplementary Figure 136. T_1 (inverse recovery). Stacked spectra of the inverse recovery experiment to determine the T_1 values of the ortho (Y) and meta (Y1) protons of **Mn1** (0.5 mM) and **VP** (5 mM) in solution, in which the signal intensity (Y) is plotted against the time in seconds (X) (^1H, 500 MHz, chloroform-*d* : acetonitrile-d_3, 303 K).

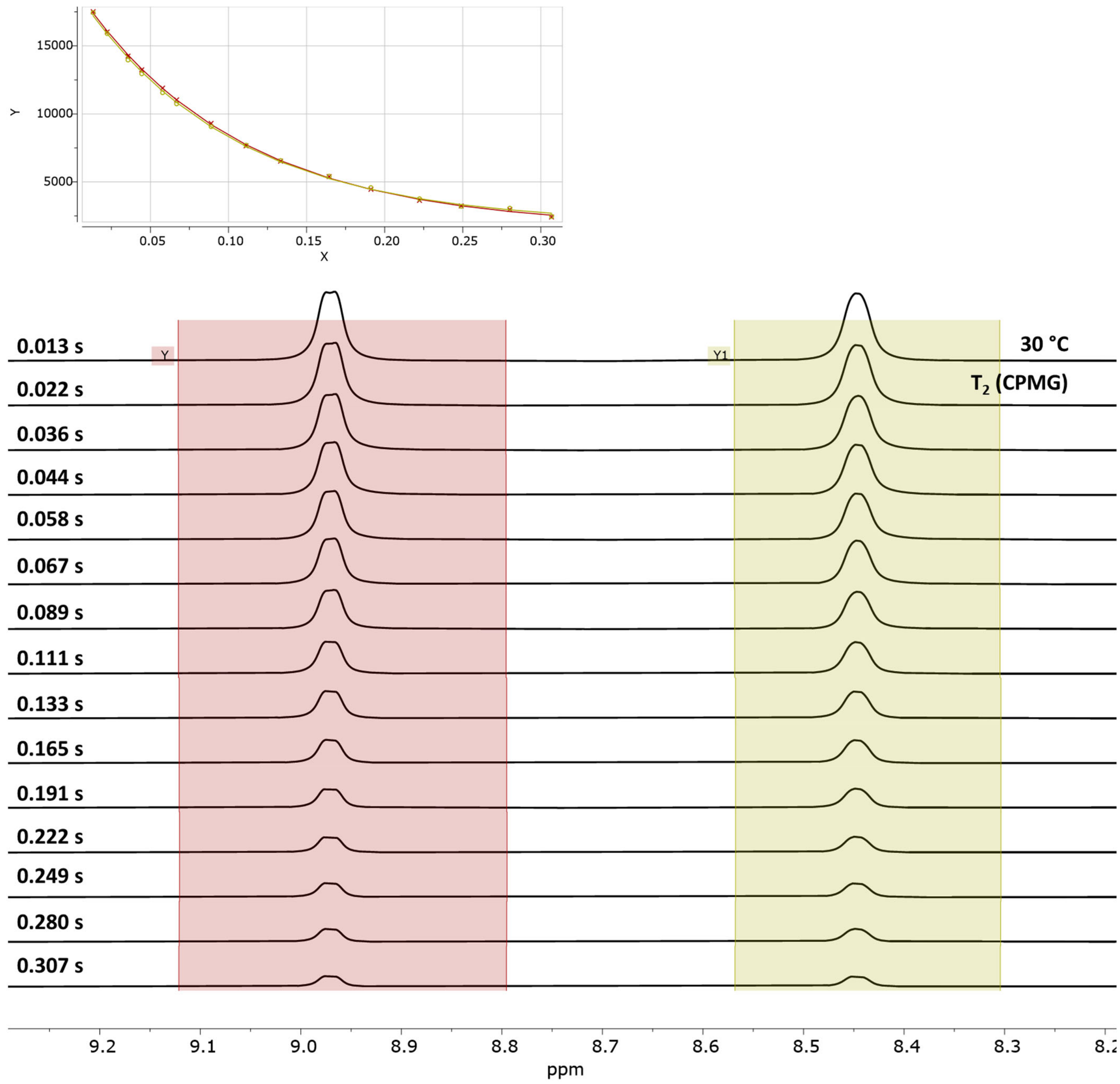

Supplementary Figure 137. T_2 (CPMG). Stacked spectra of the CPMG experiment to determine the T_2 values of the ortho (Y) and meta (Y1) protons of **Mn1** (0.5 mM) and **VP** (5 mM) in solution, in which the signal intensity (Y) is plotted against the time in seconds (X) (^{1}H, 500 MHz, chloroform-*d* : acetonitrile-*d*$_3$, 303 K).

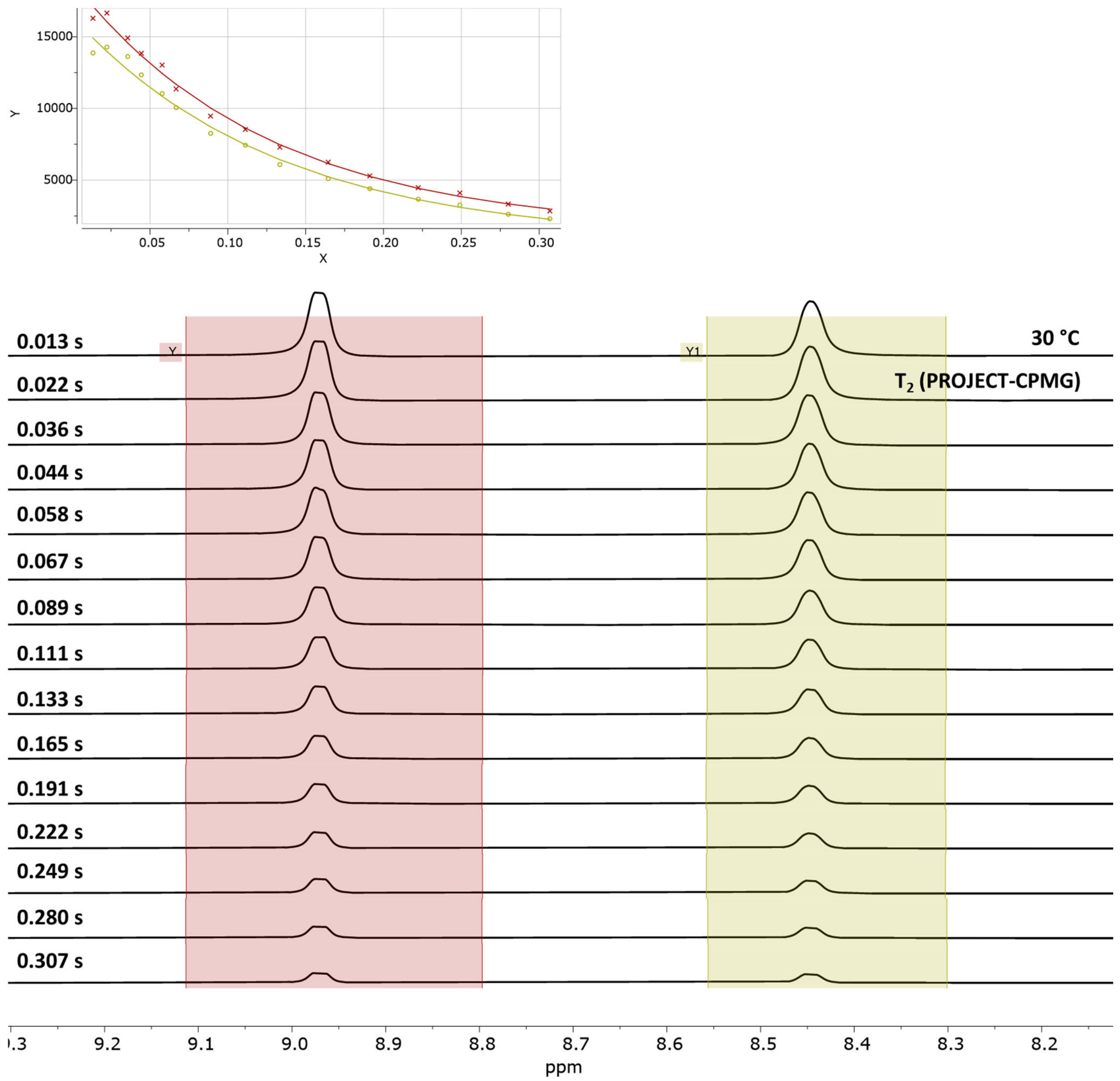

Supplementary Figure 138. T_2 (PROJECT-CPMG). Stacked spectra of the PROJECT-CPMG experiment to determine the T_2 values of the ortho (Y) and meta (Y1) protons of **Mn1** (0.5 mM) and **VP** (5 mM) in solution, in which the signal intensity (Y) is plotted against the time in seconds (X) (^{1}H, 500 MHz, chloroform-*d* : acetonitrile-*d*$_3$, 303 K).

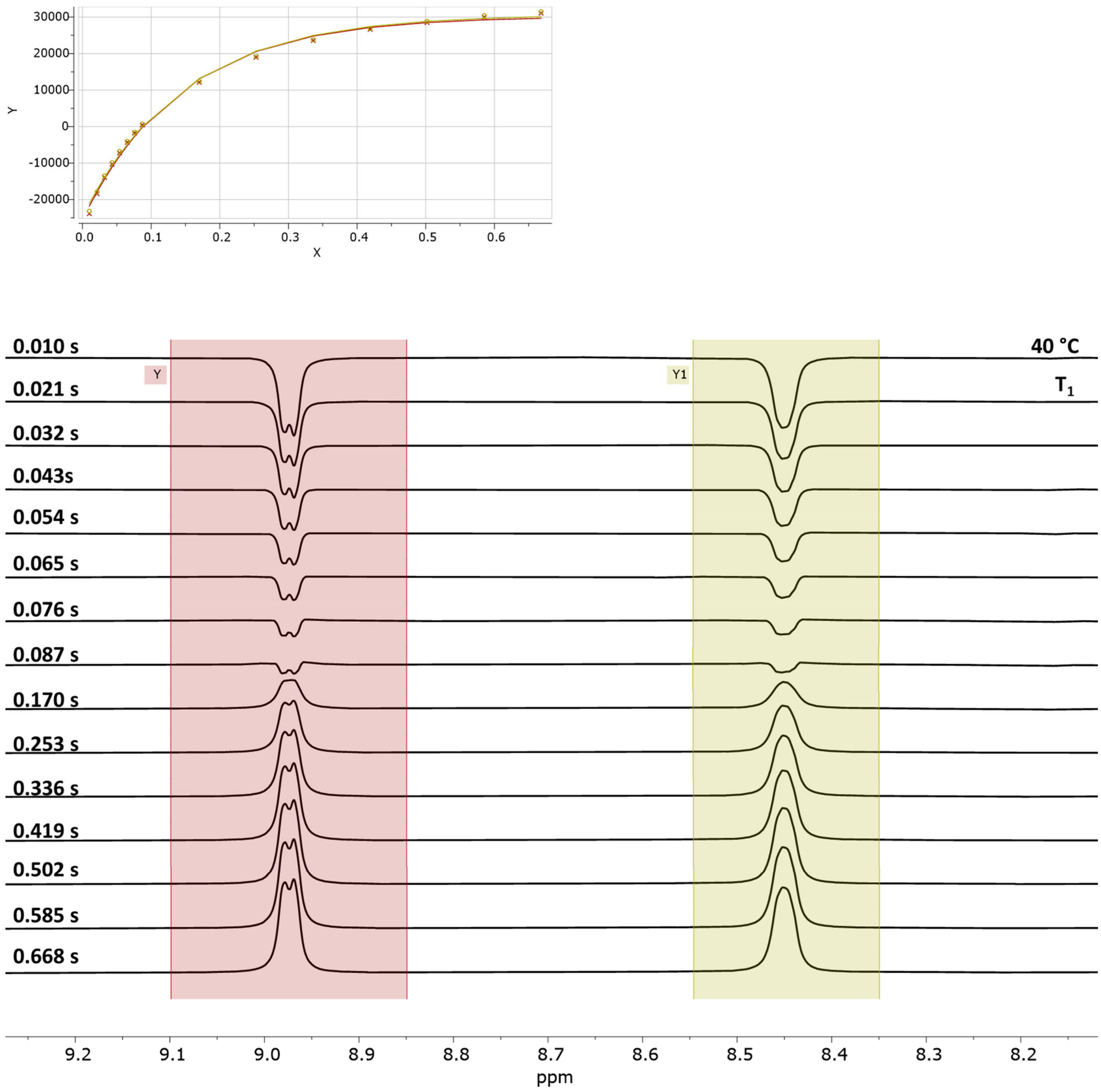

Supplementary Figure 139. T_1 (inverse recovery). Stacked spectra of the inverse recovery experiment to determine the T_1 values of the ortho (Y) and meta (Y1) protons of **Mn1** (0.5 mM) and **VP** (5 mM) in solution, in which the signal intensity (Y) is plotted against the time in seconds (X) (^{1}H, 500 MHz, chloroform-*d* : acetonitrile-*d*$_3$, 313 K).

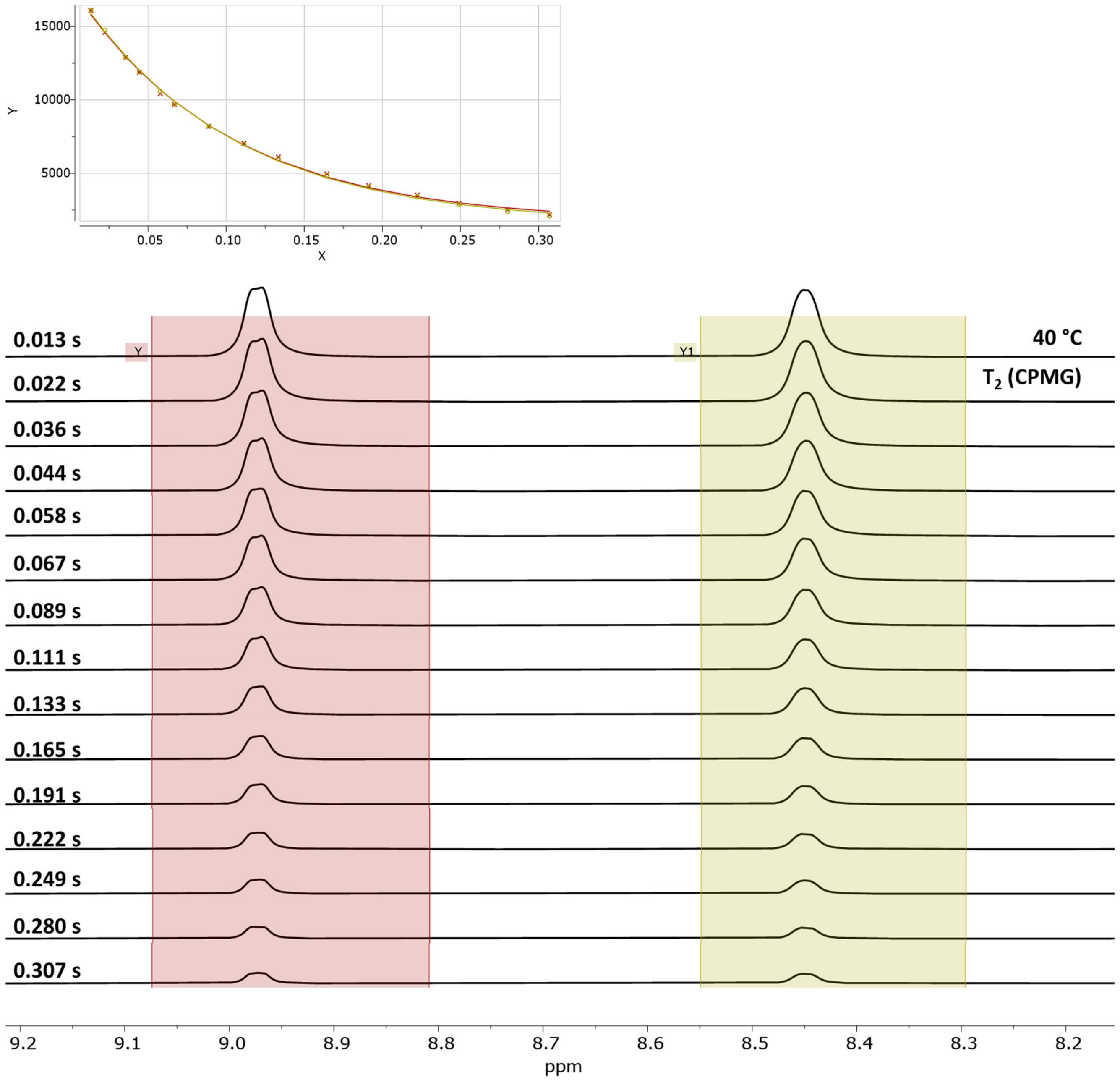

Supplementary Figure 140. T_2 (CPMG). Stacked spectra of the CPMG experiment to determine the T_2 values of the ortho (Y) and meta (Y1) protons of **Mn1** (0.5 mM) and **VP** (5 mM) in solution, in which the signal intensity (Y) is plotted against the time in seconds (X) (^{1}H, 500 MHz, chloroform-*d* : acetonitrile-*d*$_3$, 313 K).

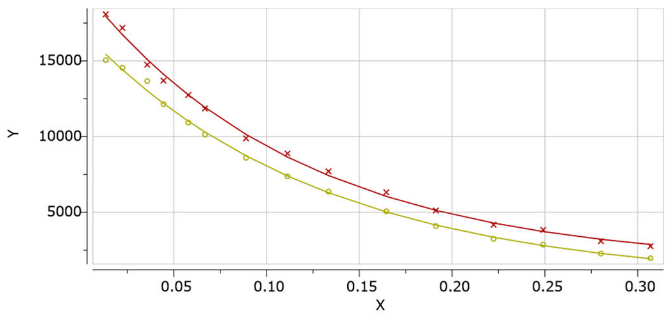

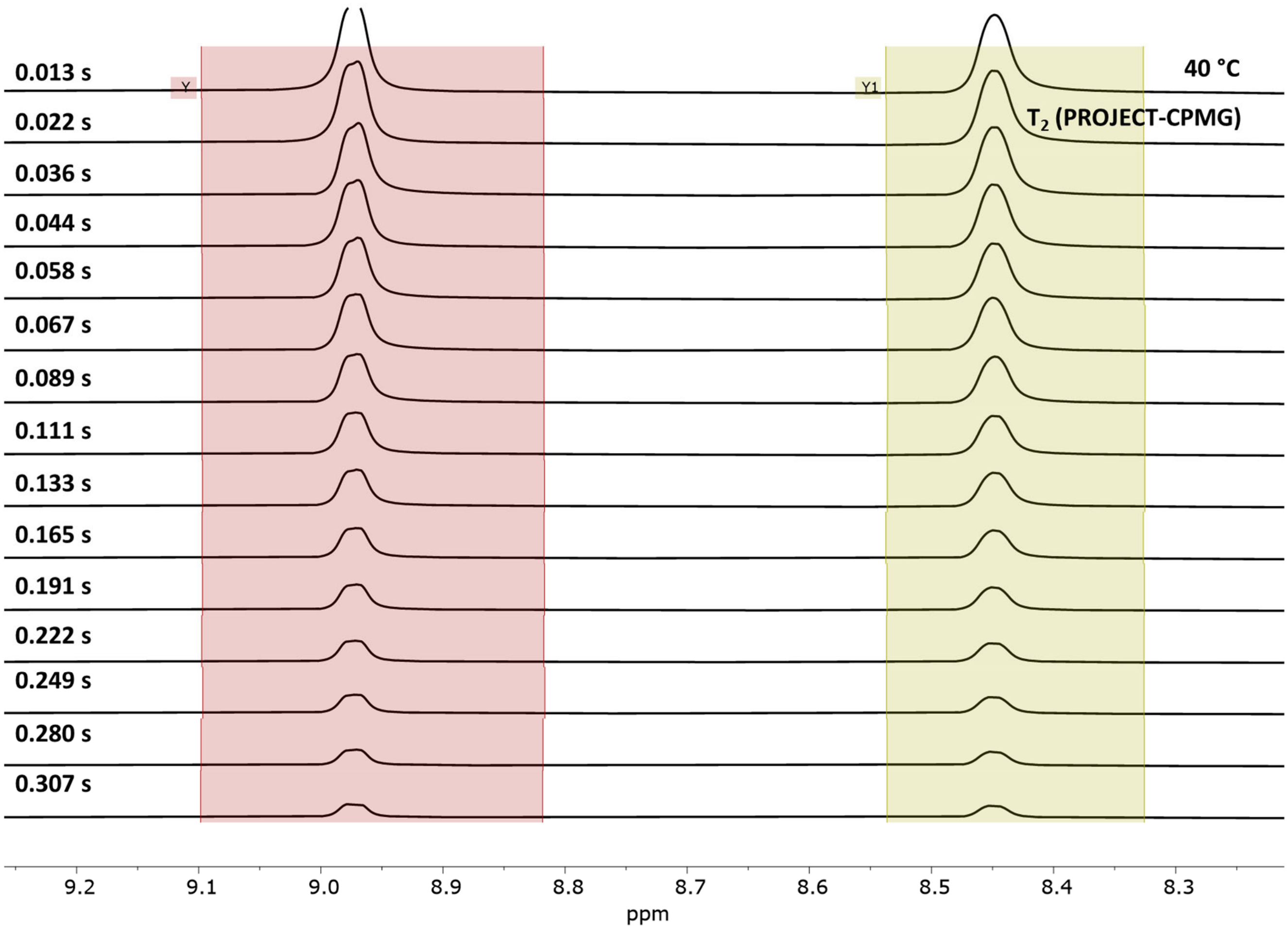

Supplementary Figure 141. T_2 (PROJECT-CPMG). Stacked spectra of the PROJECT-CPMG experiment to determine the T_2 values of the ortho (Y) and meta (Y1) protons of **Mn1** (0.5 mM) and **VP** (5 mM) in solution, in which the signal intensity (Y) is plotted against the time in seconds (X) (^{1}H, 500 MHz, chloroform-*d* : acetonitrile-d_3, 313 K).

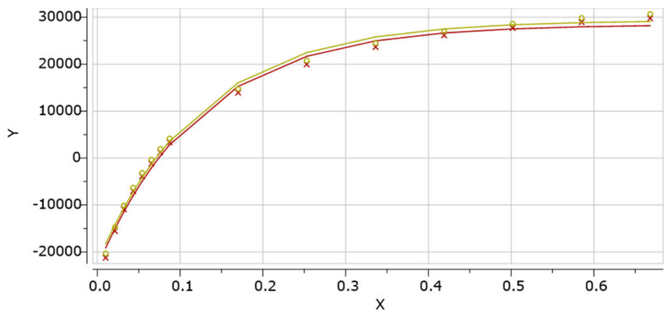

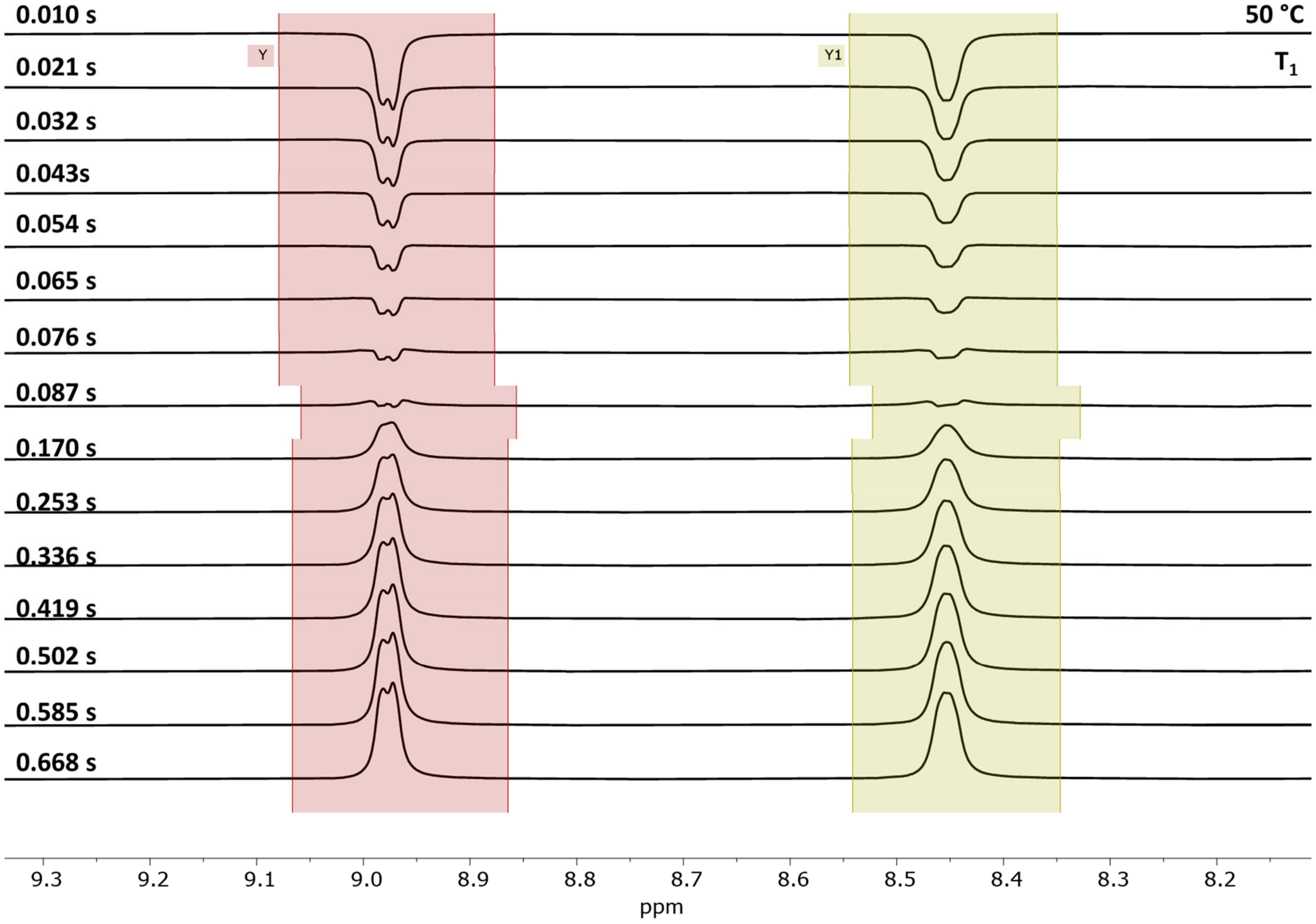

Supplementary Figure 142. T_1 (inverse recovery). Stacked spectra of the inverse recovery experiment to determine the T_1 values of the ortho (Y) and meta (Y1) protons of **Mn1** (0.5 mM) and **VP** (5 mM) in solution, in which the signal intensity (Y) is plotted against the time in seconds (X) (^{1}H, 500 MHz, chloroform-*d* : acetonitrile-*d*$_3$, 323 K).

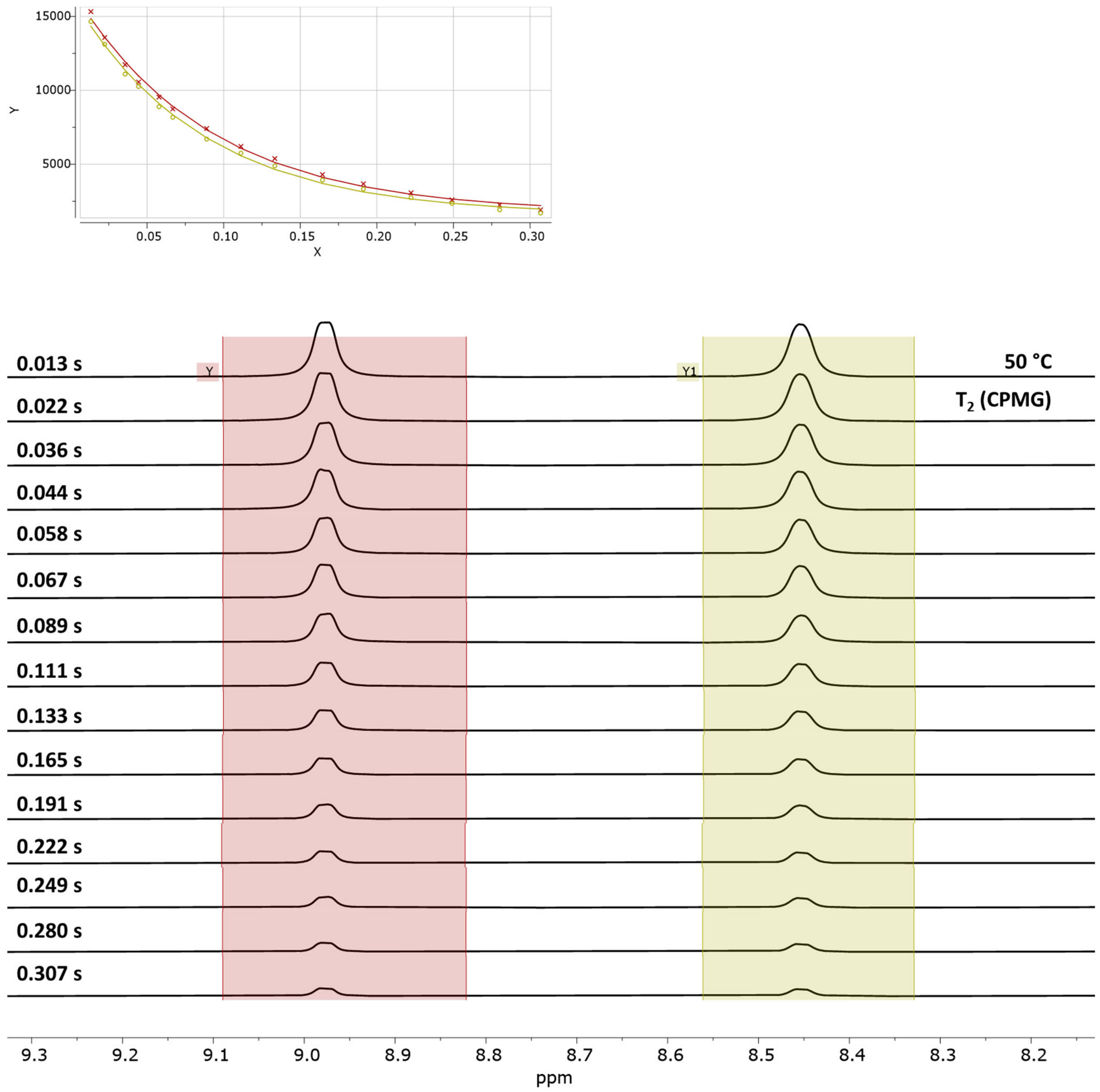

Supplementary Figure 143. T_2 (CPMG). Stacked spectra of the CPMG experiment to determine the T_2 values of the ortho (Y) and meta (Y1) protons of **Mn1** (0.5 mM) and **VP** (5 mM) in solution, in which the signal intensity (Y) is plotted against the time in seconds (X) (^{1}H, 500 MHz, chloroform-*d* : acetonitrile-*d*$_3$, 323 K).

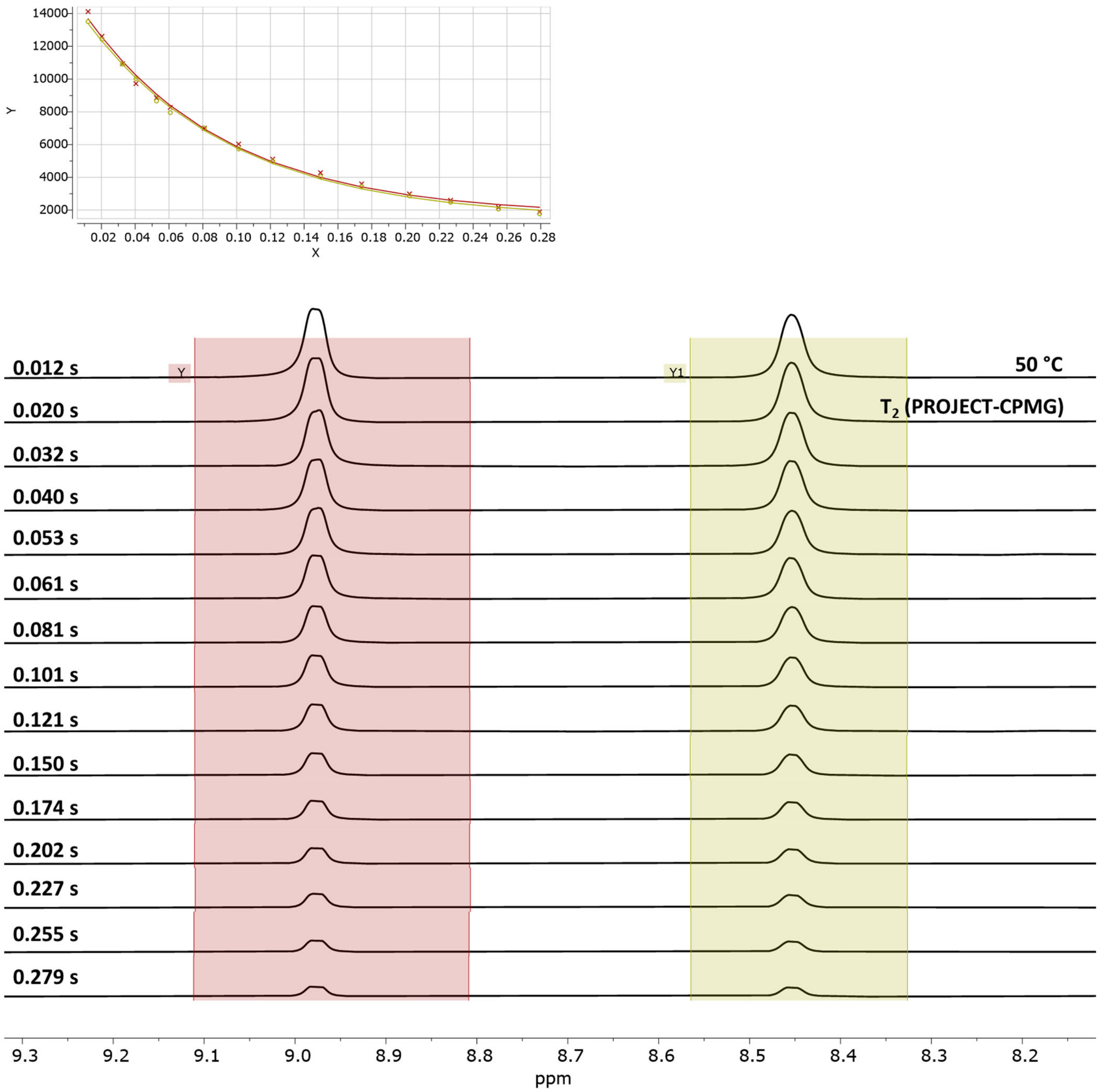

Supplementary Figure 144. T_2 (PROJECT-CPMG). Stacked spectra of the PROJECT-CPMG experiment to determine the T_2 values of the ortho (Y) and meta (Y1) protons of **Mn1** (0.5 mM) and **VP** (5 mM) in solution, in which the signal intensity (Y) is plotted against the time in seconds (X) (^{1}H, 500 MHz, chloroform-*d* : acetonitrile-*d*$_3$, 323 K).

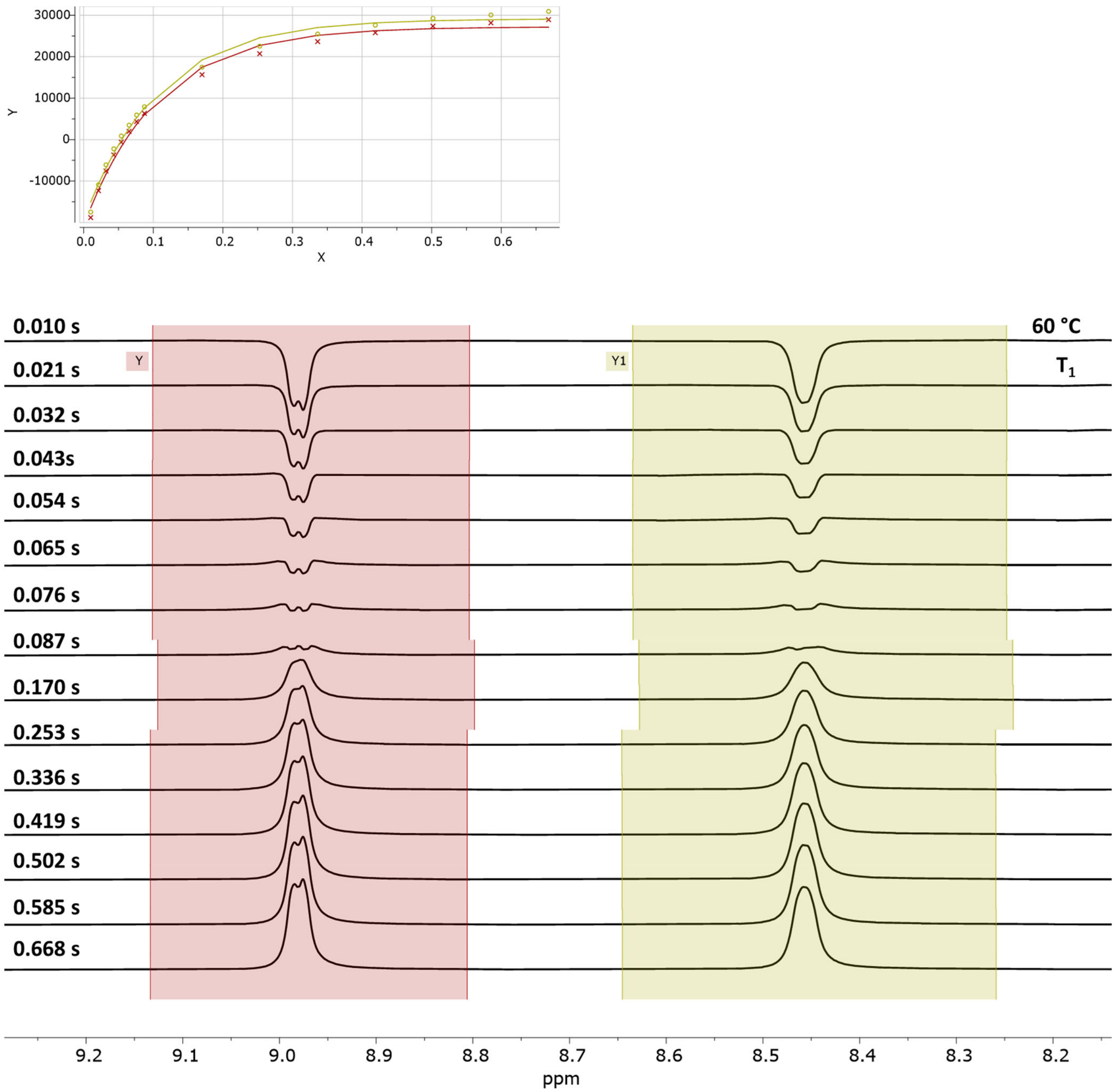

Supplementary Figure 145. T_1 (inverse recovery). Stacked spectra of the inverse recovery experiment to determine the T_1 values of the ortho (Y) and meta (Y1) protons of **Mn1** (0.5 mM) and **VP** (5 mM) in solution, in which the signal intensity (Y) is plotted against the time in seconds (X) (^{1}H, 500 MHz, chloroform-*d* : acetonitrile-d_3, 333 K).

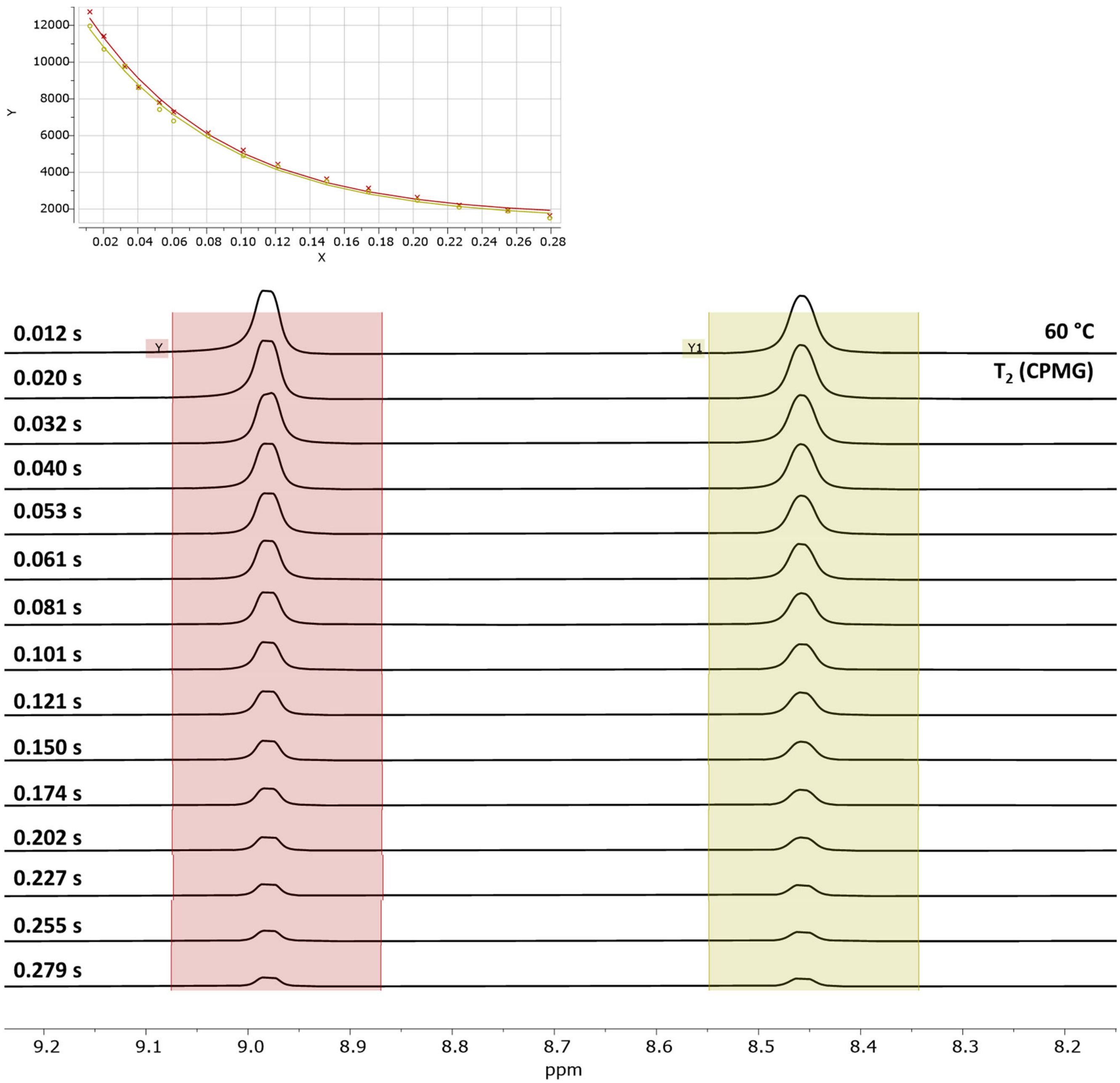

Supplementary Figure 146. T_2 (CPMG). Stacked spectra of the CPMG experiment to determine the T_2 values of the ortho (Y) and meta (Y1) protons of **Mn1** (0.5 mM) and **VP** (5 mM) in solution, in which the signal intensity (Y) is plotted against the time in seconds (X) (^{1}H, 500 MHz, chloroform-*d* : acetonitrile-*d*$_3$, 333 K).

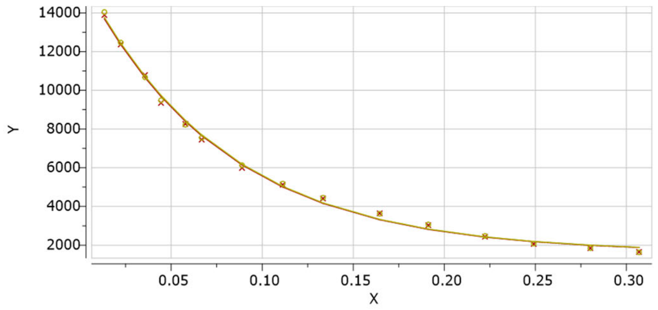

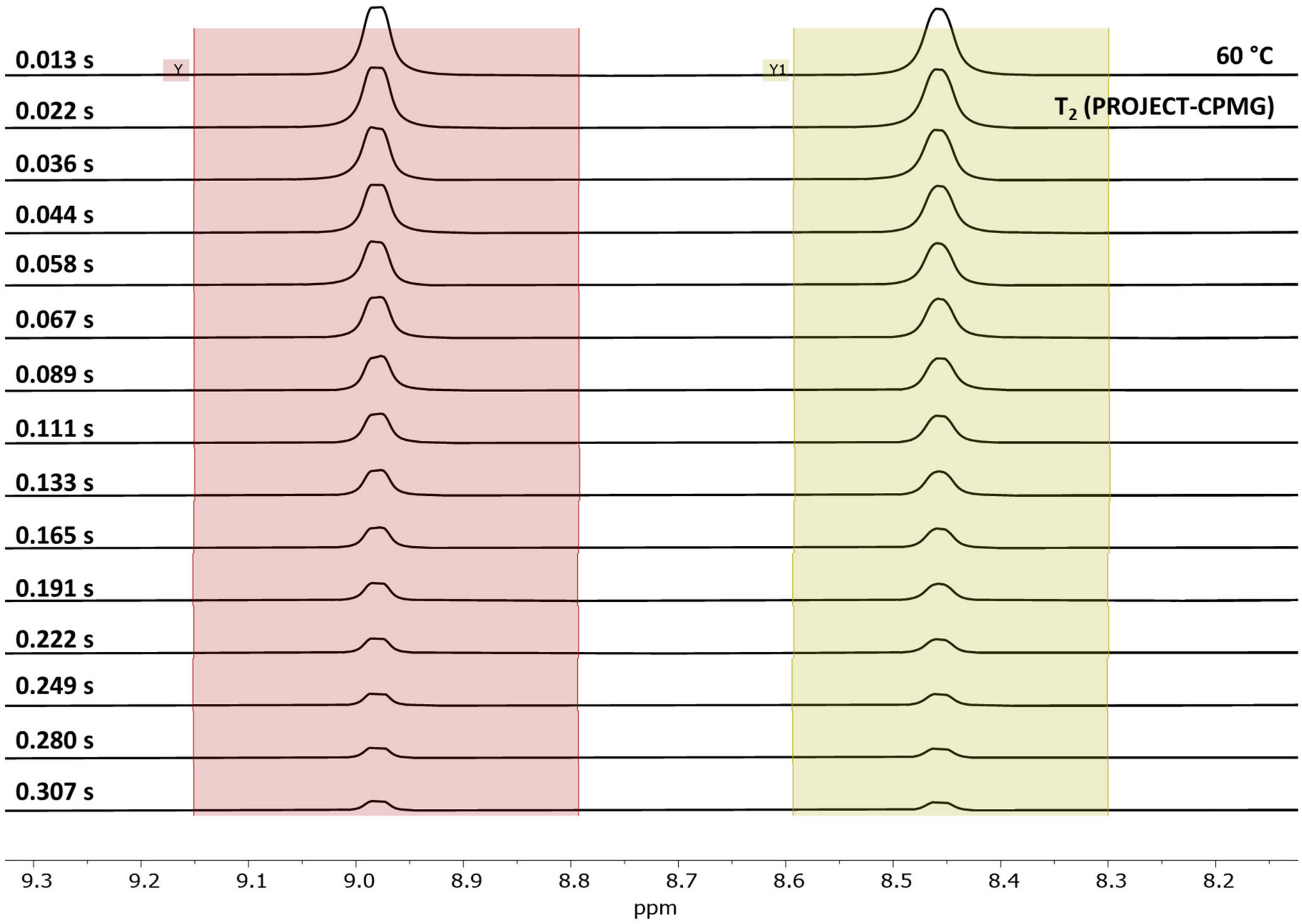

Supplementary Figure 147. T_2 (PROJECT-CPMG). Stacked spectra of the PROJECT-CPMG experiment to determine the T_2 values of the ortho (Y) and meta (Y1) protons of **Mn1** (0.5 mM) and **VP** (5 mM) in solution, in which the signal intensity (Y) is plotted against the time in seconds (X) (^{1}H, 500 MHz, chloroform-*d* : acetonitrile-*d$_3$*, 333 K).

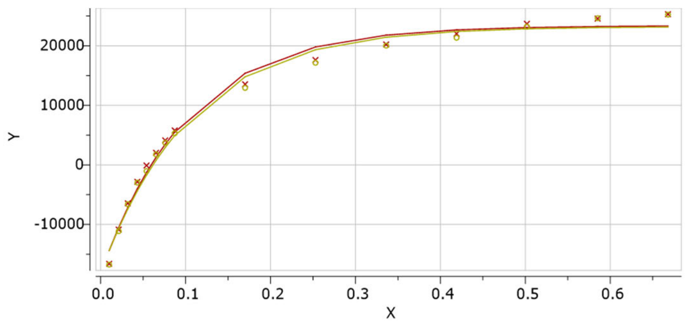

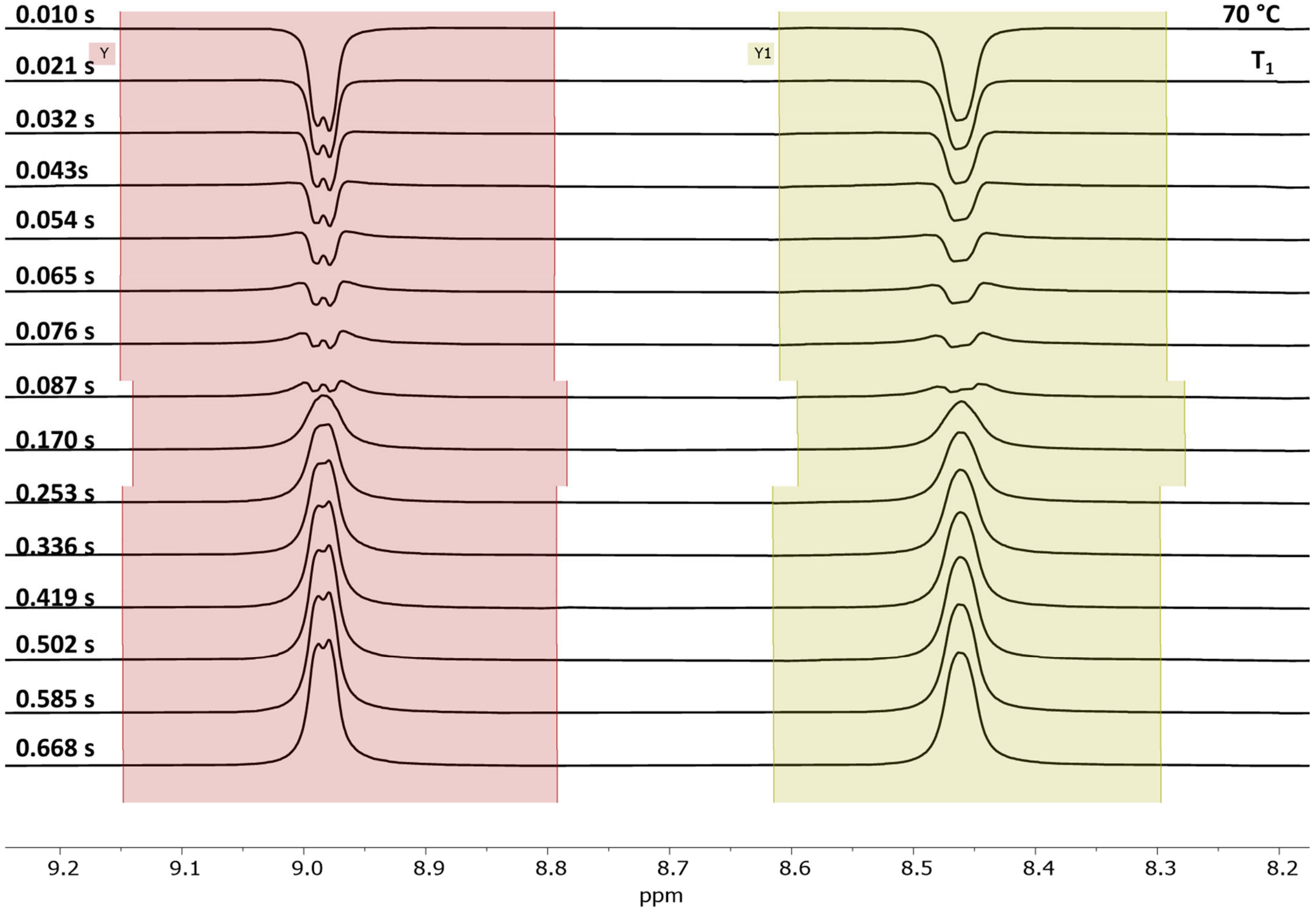

Supplementary Figure 148. T_1 (inverse recovery). Stacked spectra of the inverse recovery experiment to determine the T_1 values of the ortho (Y) and meta (Y1) protons of **Mn1** (0.5 mM) and **VP** (5 mM) in solution, in which the signal intensity (Y) is plotted against the time in seconds (X) (^{1}H, 500 MHz, chloroform-*d* : acetonitrile-*d*$_3$, 343 K).

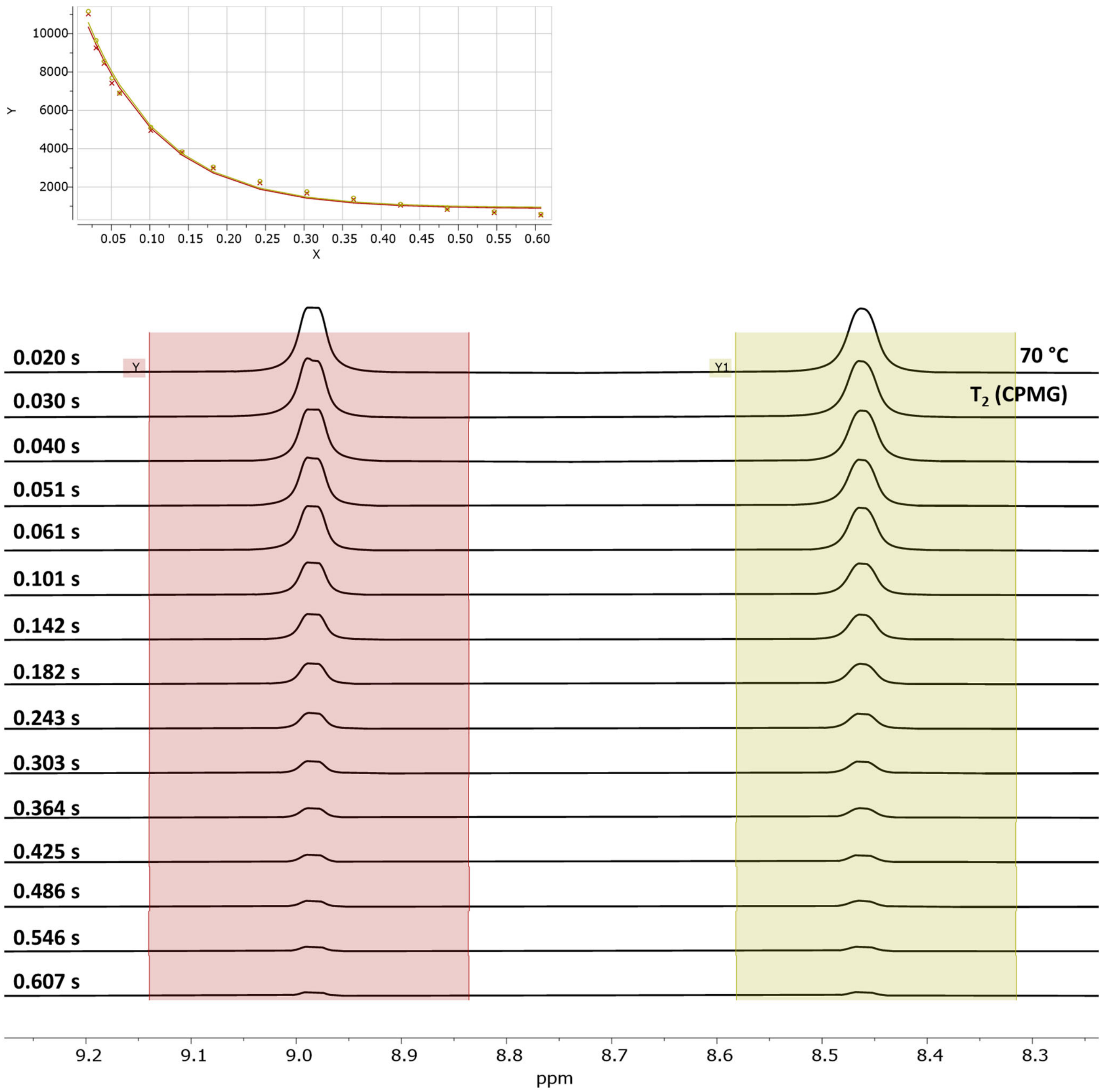

Supplementary Figure 149. T_2 (CPMG). Stacked spectra of the CPMG experiment to determine the T_2 values of the ortho (Y) and meta (Y1) protons of **Mn1** (0.5 mM) and **VP** (5 mM) in solution, in which the signal intensity (Y) is plotted against the time in seconds (X) (^{1}H, 500 MHz, chloroform-*d* : acetonitrile-*d$_3$*, 343 K).

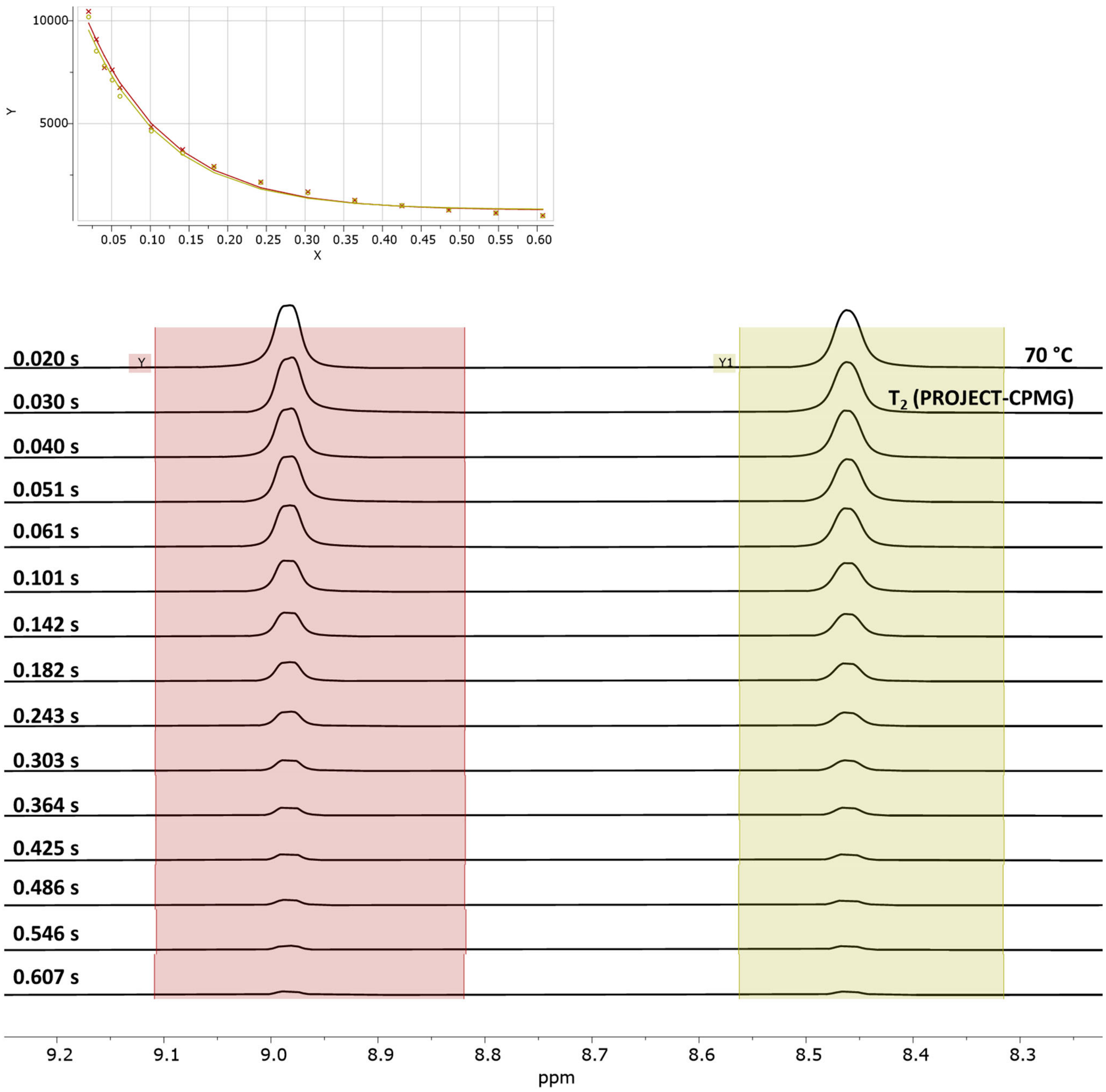

Supplementary Figure 150. T_2 (PROJECT-CPMG). Stacked spectra of the PROJECT-CPMG experiment to determine the T_2 values of the ortho (Y) and meta (Y1) protons of **Mn1** (0.5 mM) and **VP** (5 mM) in solution, in which the signal intensity (Y) is plotted against the time in seconds (X) (^{1}H, 500 MHz, chloroform-*d* : acetonitrile-*d*$_3$, 343 K).

4.6. MnRot/VP ($T_{1,os}$ and $T_{2,os}$)

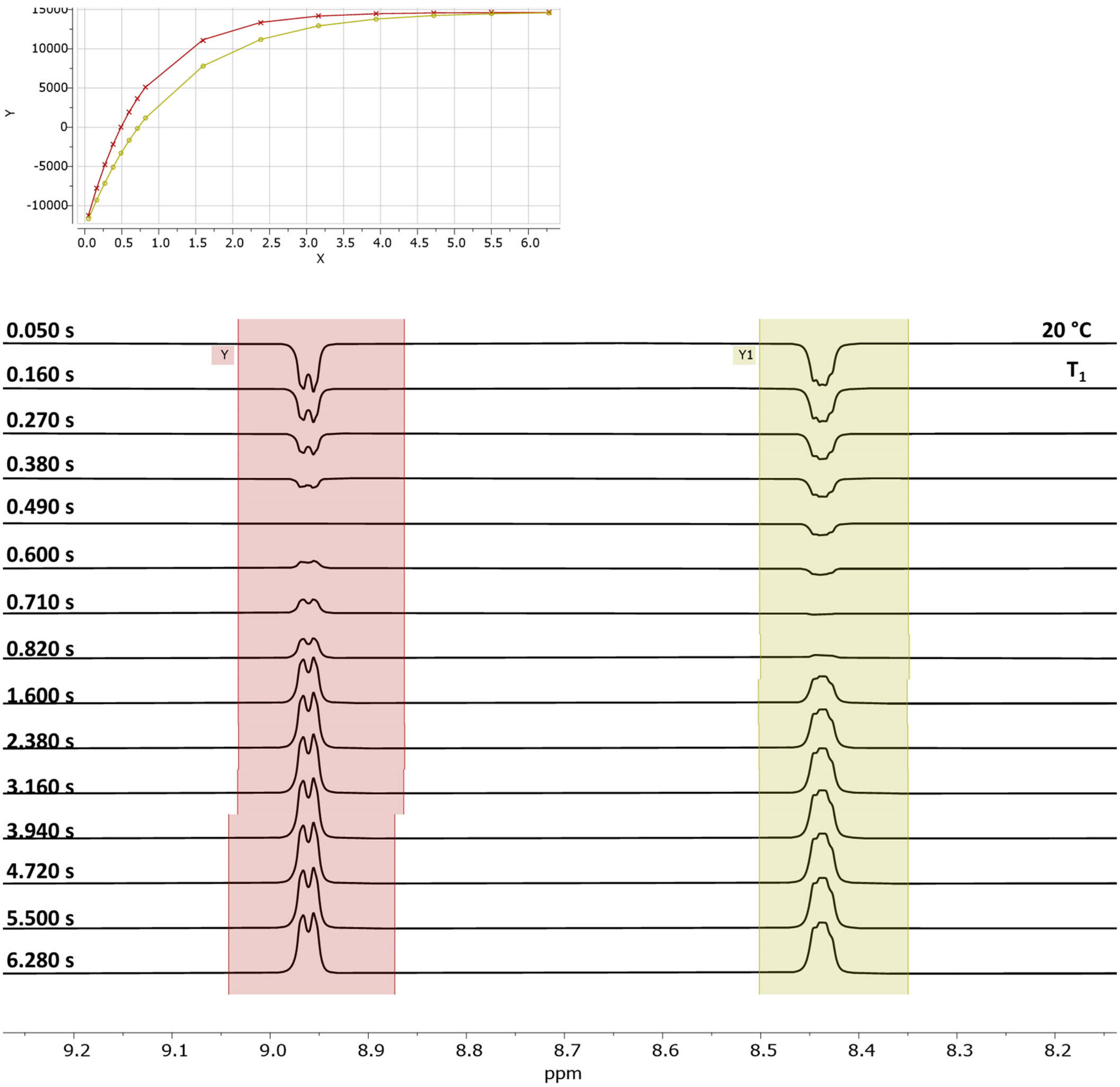

Supplementary Figure 151. T_1 (inverse recovery). Stacked spectra of the inverse recovery experiment to determine the T_1 values of the ortho (Y) and meta (Y1) protons of **MnRot** (0.5 mM) and **VP** (5 mM) in solution, in which the signal intensity (Y) is plotted against the time in seconds (X) (^{1}H, 500 MHz, chloroform-*d* : acetonitrile-d_3, 293 K).

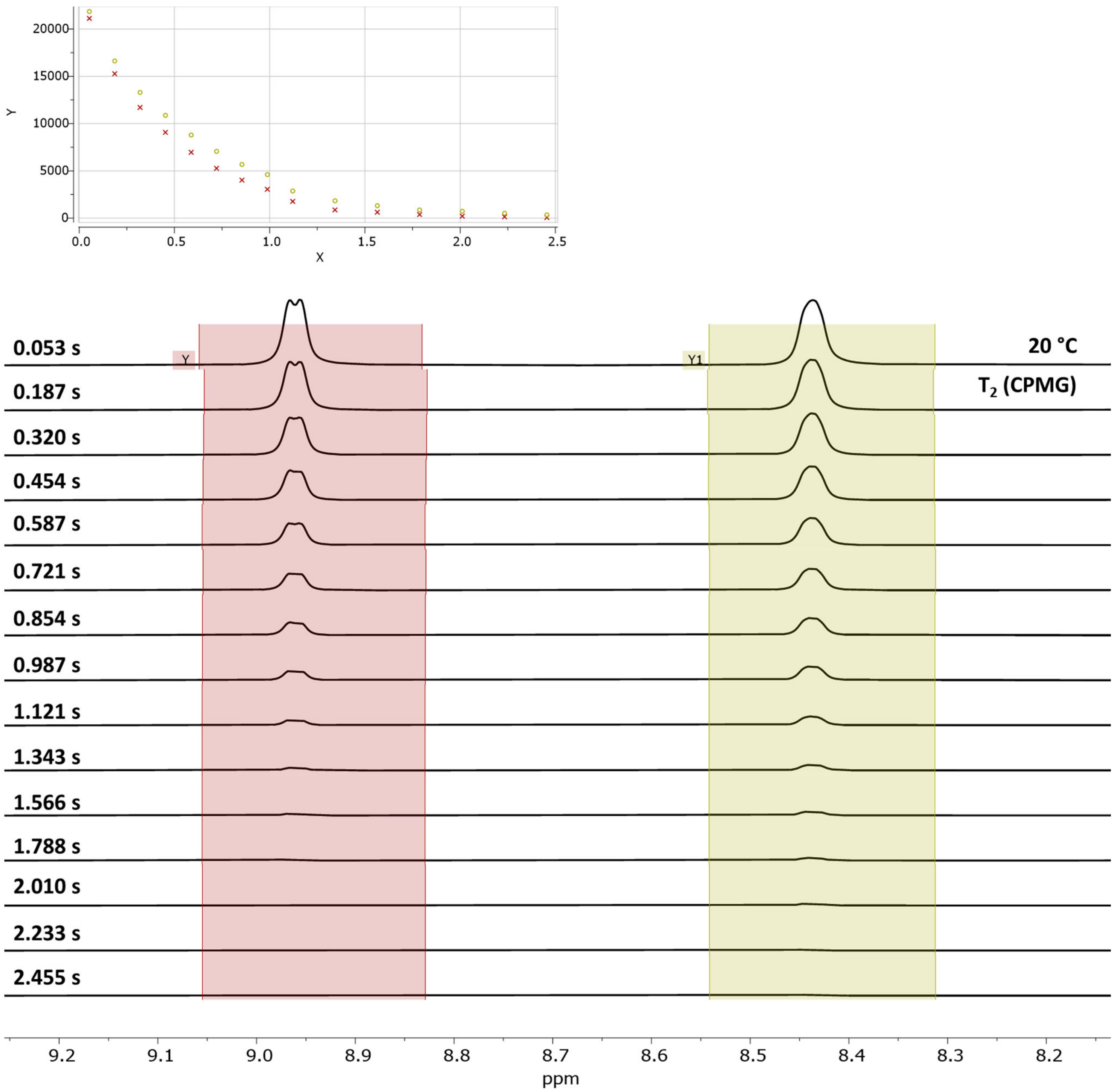

Supplementary Figure 152. T_2 (CPMG). Stacked spectra of the CPMG experiment to determine the T_2 values of the ortho (Y) and meta (Y1) protons of **MnRot** (0.5 mM) and **VP** (5 mM) in solution, in which the signal intensity (Y) is plotted against the time in seconds (X) (^{1}H, 500 MHz, chloroform-*d* : acetonitrile-*d*$_3$, 293 K).

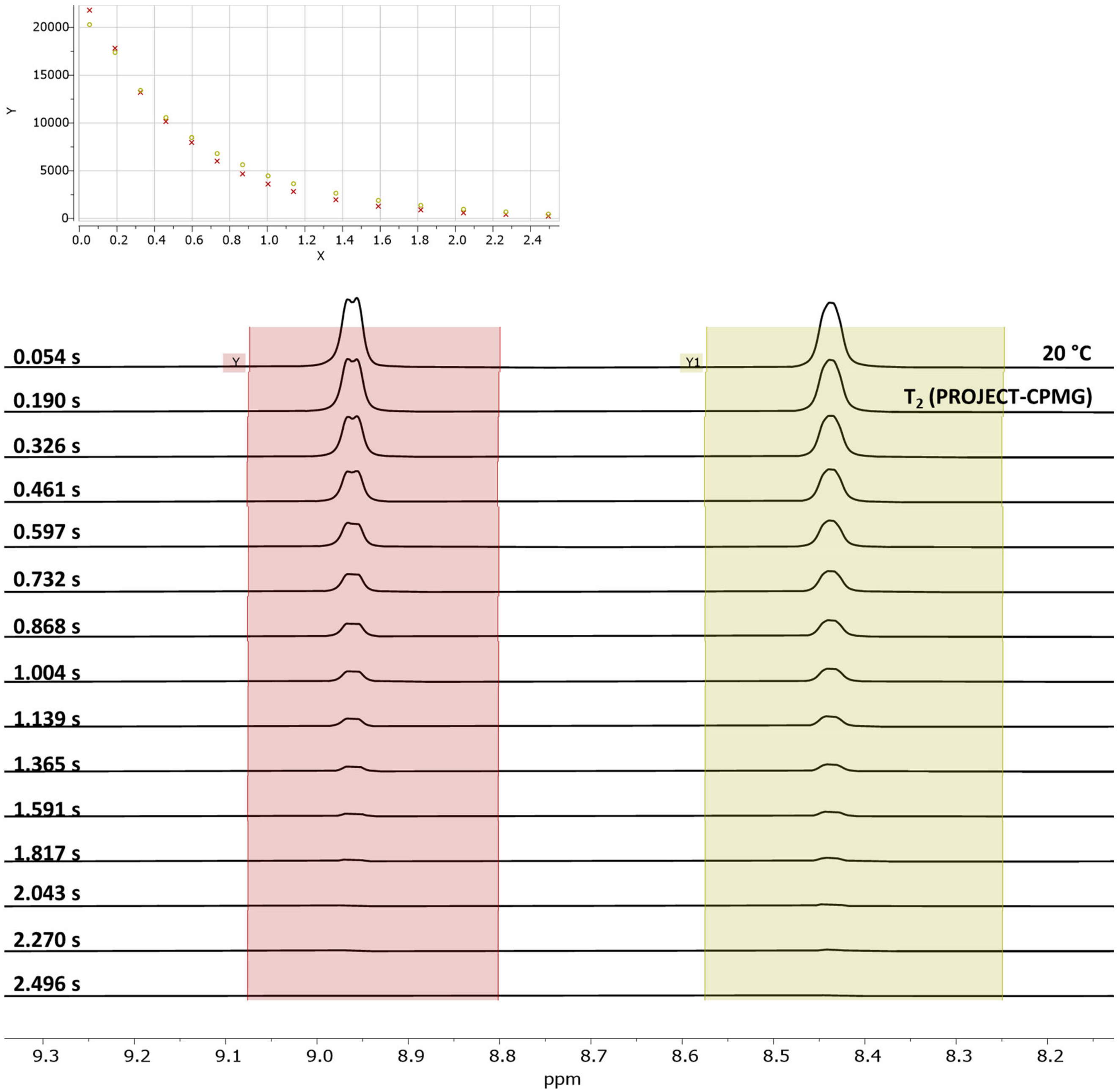

Supplementary Figure 153. T_2 (PROJECT-CPMG). Stacked spectra of the PROJECT-CPMG experiment to determine the T_2 values of the ortho (Y) and meta (Y1) protons of **MnRot** (0.5 mM) and **VP** (5 mM) in solution, in which the signal intensity (Y) is plotted against the time in seconds (X) (^{1}H, 500 MHz, chloroform-*d* : acetonitrile-*d*$_3$, 293 K).

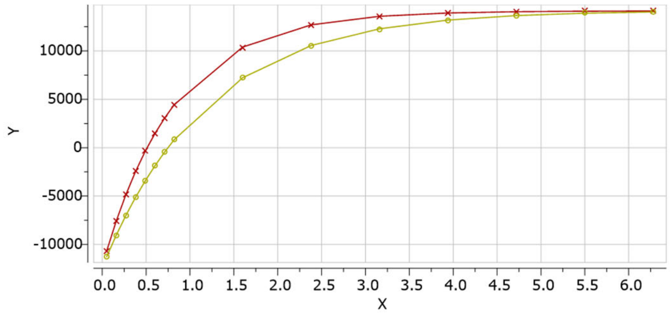

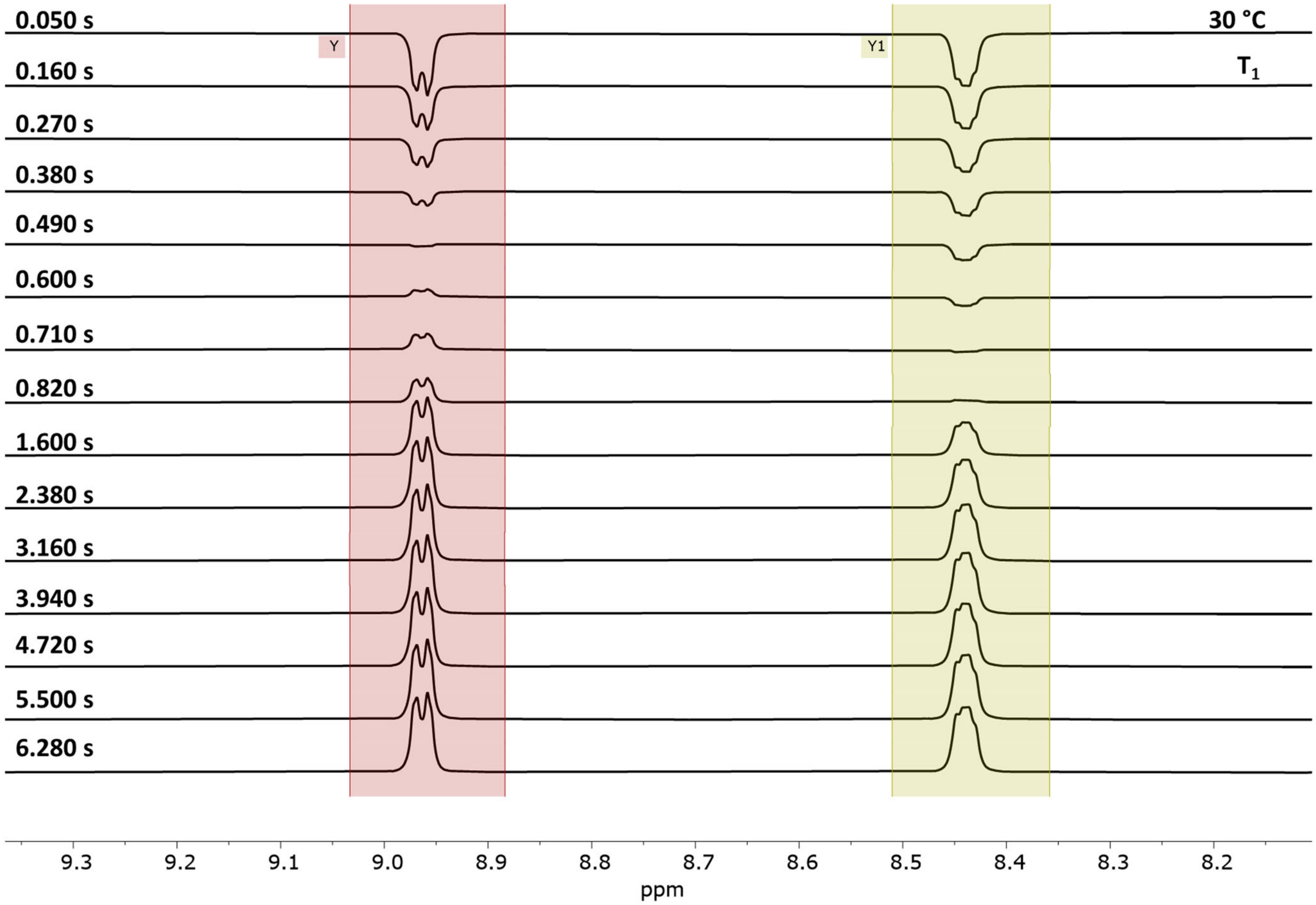

Supplementary Figure 154. T_1 (inverse recovery). Stacked spectra of the inverse recovery experiment to determine the T_1 values of the ortho (Y) and meta (Y1) protons of **MnRot** (0.5 mM) and **VP** (5 mM) in solution, in which the signal intensity (Y) is plotted against the time in seconds (X) (^{1}H, 500 MHz, chloroform-*d* : acetonitrile-*d*$_3$, 303 K).

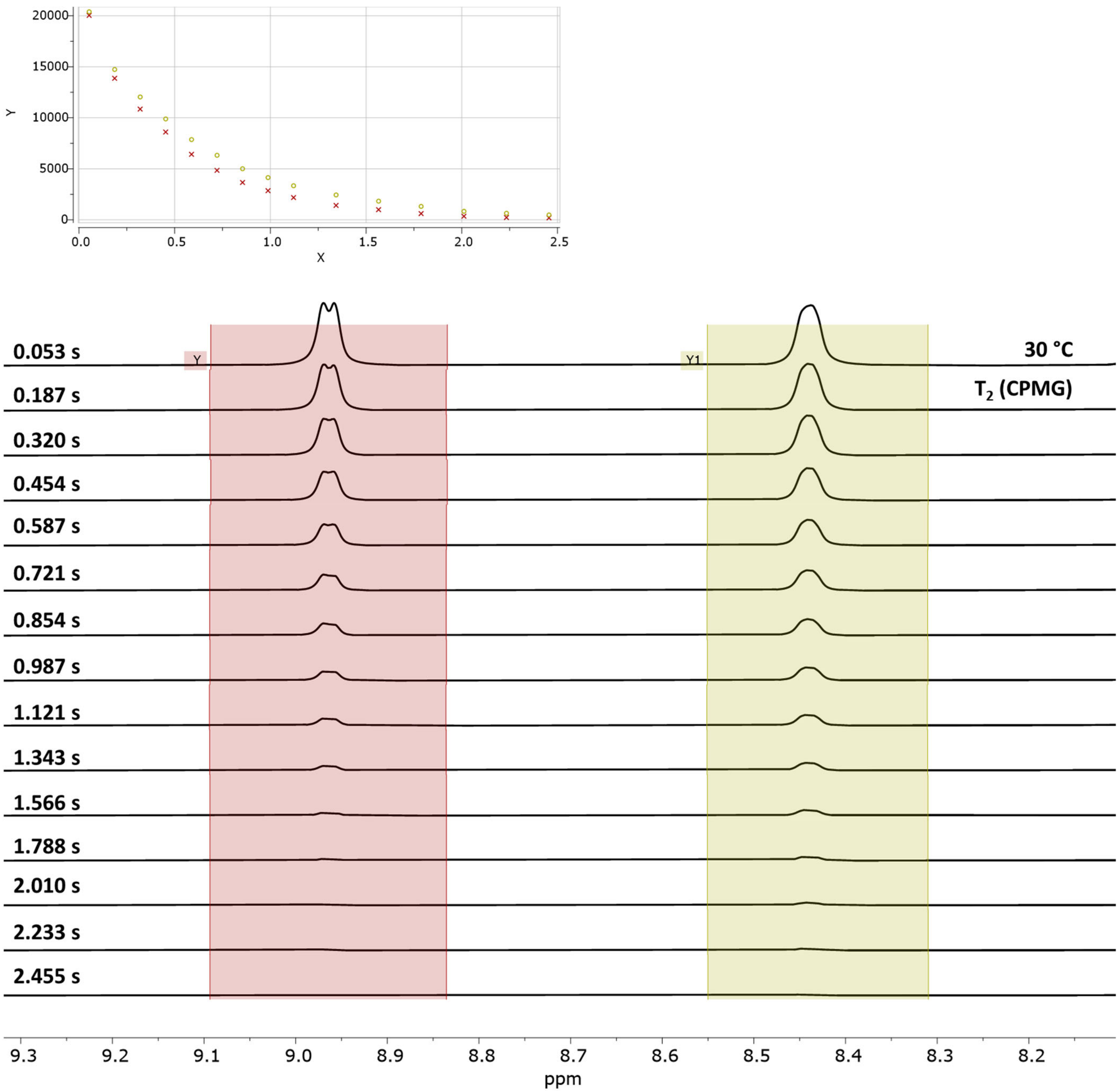

Supplementary Figure 155. T_2 (CPMG). Stacked spectra of the CPMG experiment to determine the T_2 values of the ortho (Y) and meta (Y1) protons of **MnRot** (0.5 mM) and **VP** (5 mM) in solution, in which the signal intensity (Y) is plotted against the time in seconds (X) (^{1}H, 500 MHz, chloroform-*d* : acetonitrile-*d*$_3$, 303 K).

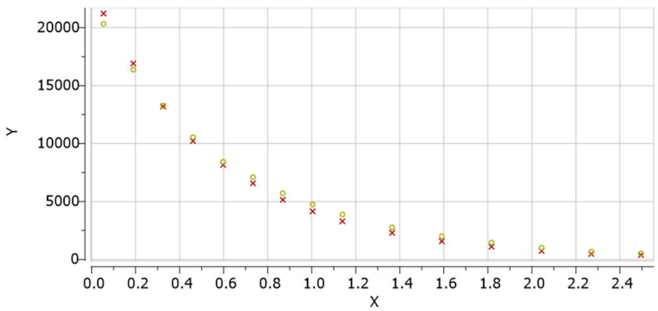

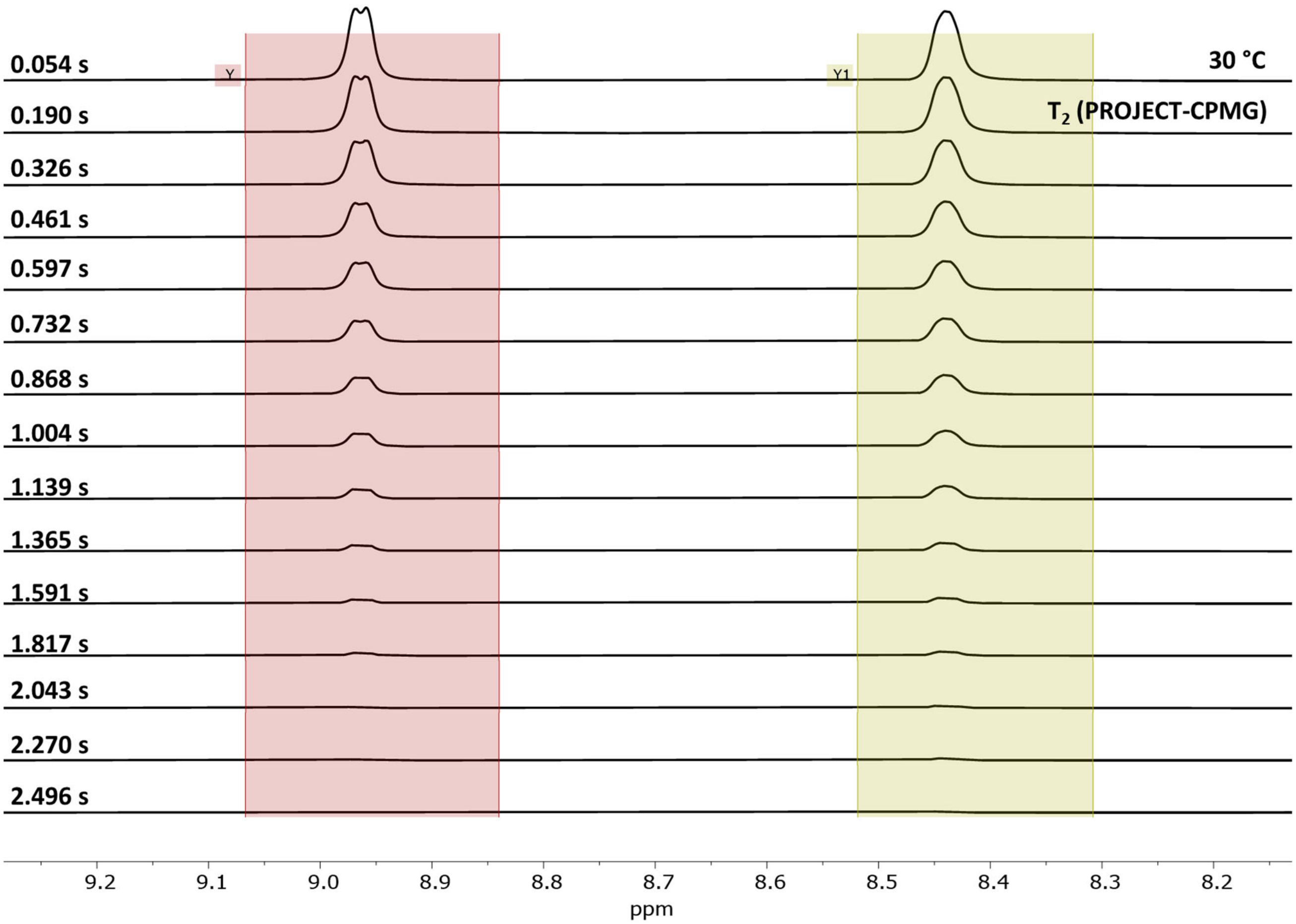

Supplementary Figure 156. T_2 (PROJECT-CPMG). Stacked spectra of the PROJECT-CPMG experiment to determine the T_2 values of the ortho (Y) and meta (Y1) protons of **MnRot** (0.5 mM) and **VP** (5 mM) in solution, in which the signal intensity (Y) is plotted against the time in seconds (X) (^{1}H, 500 MHz, chloroform-*d* : acetonitrile-*d*$_3$, 303 K).

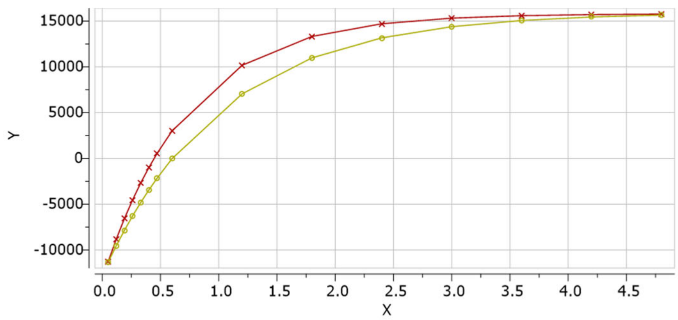

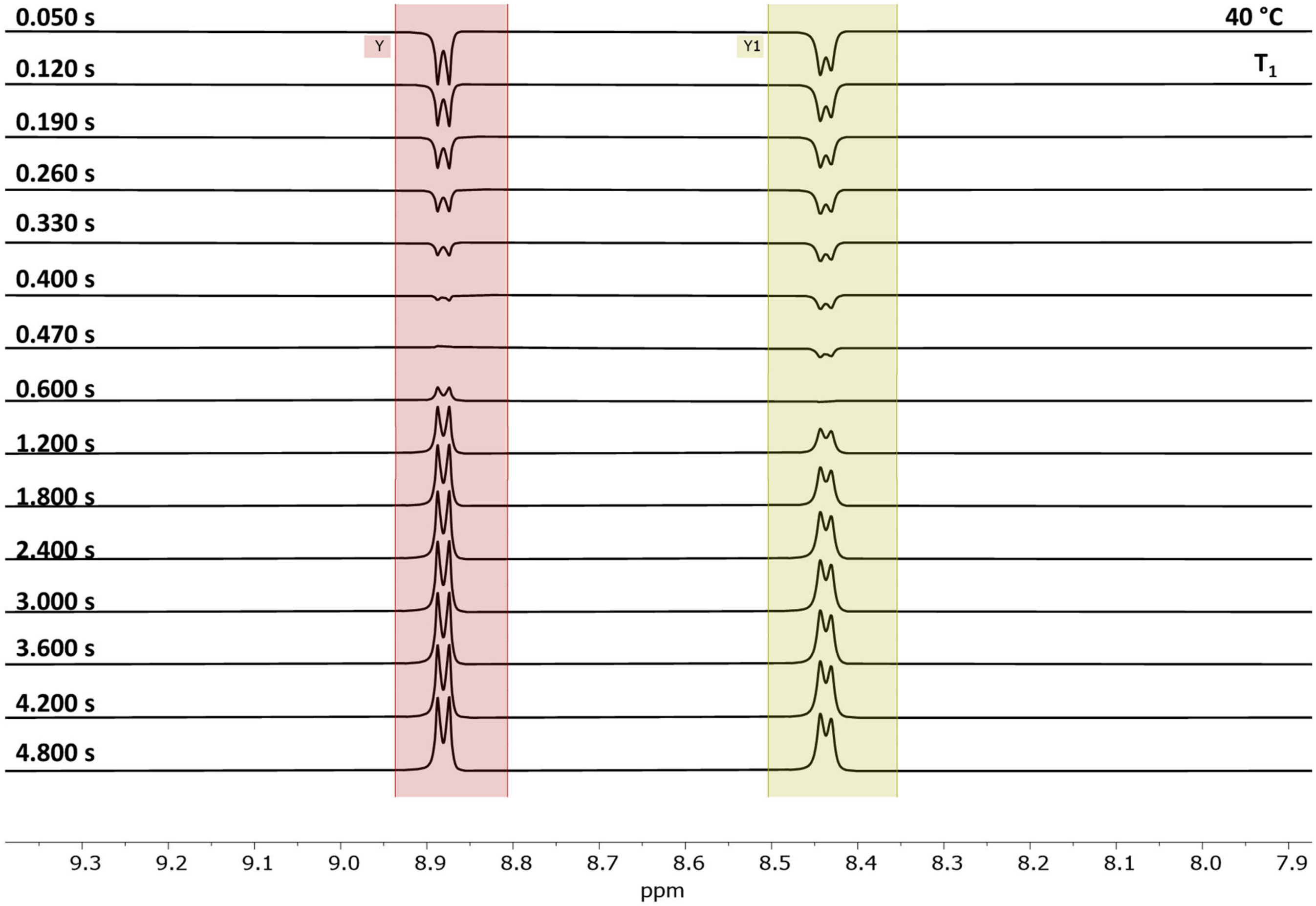

Supplementary Figure 157. T_1 (inverse recovery). Stacked spectra of the inverse recovery experiment to determine the T_1 values of the ortho (Y) and meta (Y1) protons of **MnRot** (0.5 mM) and **VP** (5 mM) in solution, in which the signal intensity (Y) is plotted against the time in seconds (X) (^{1}H, 500 MHz, chloroform-*d* : acetonitrile-*d*$_3$, 313 K).

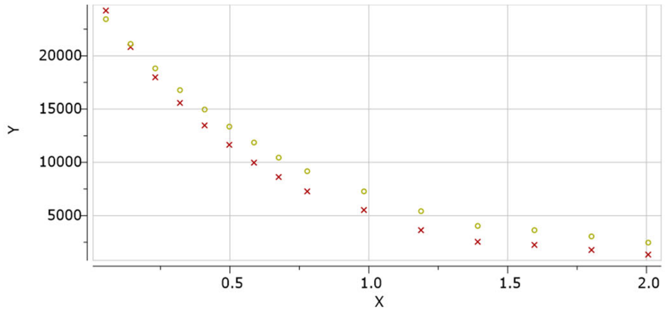

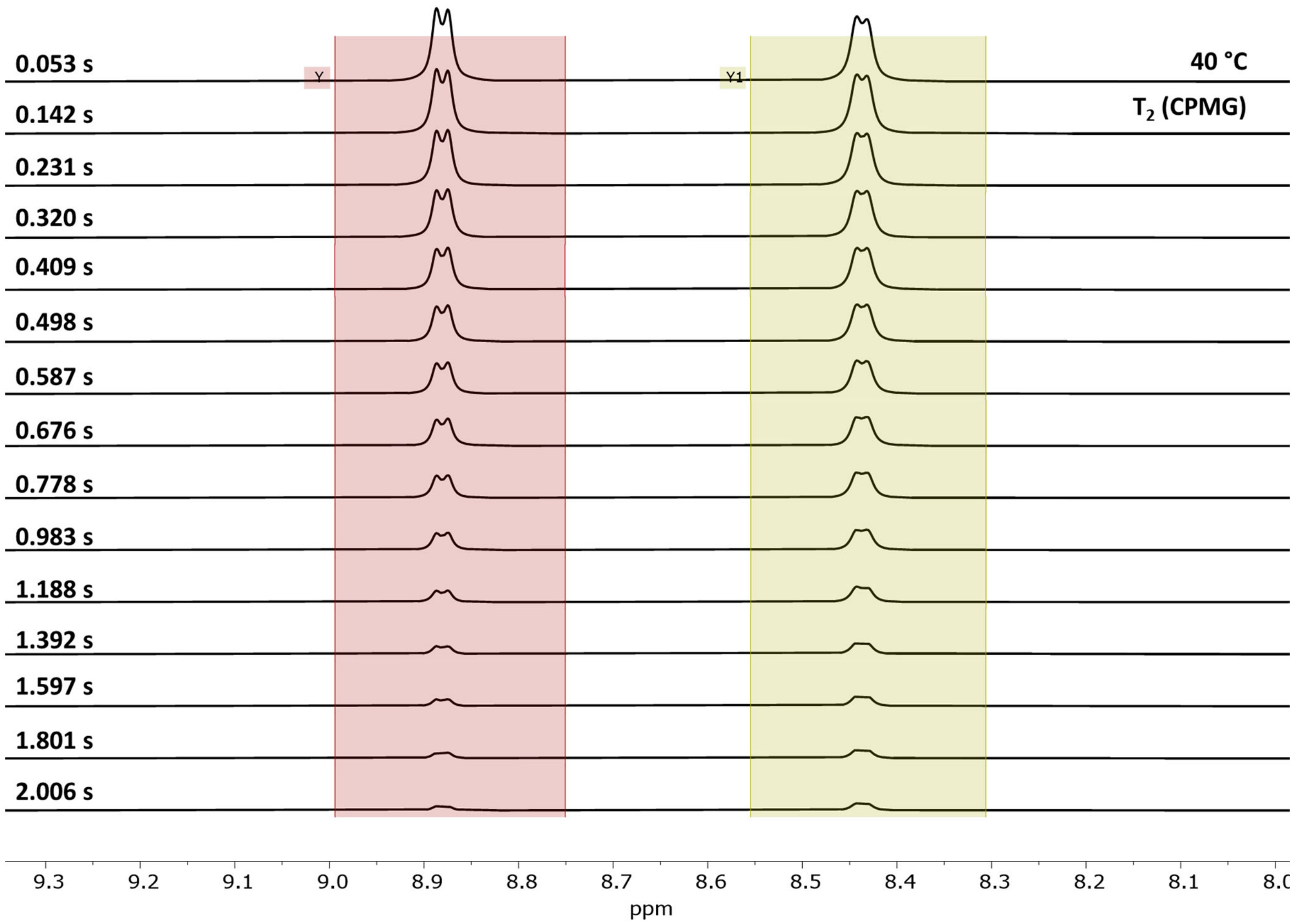

Supplementary Figure 158. T_2 (CPMG). Stacked spectra of the CPMG experiment to determine the T_2 values of the ortho (Y) and meta (Y1) protons of **MnRot** (0.5 mM) and **VP** (5 mM) in solution, in which the signal intensity (Y) is plotted against the time in seconds (X) (^{1}H, 500 MHz, chloroform-*d* : acetonitrile-*d*$_3$, 313 K).

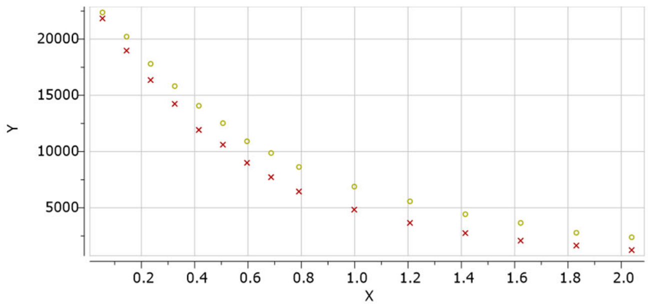

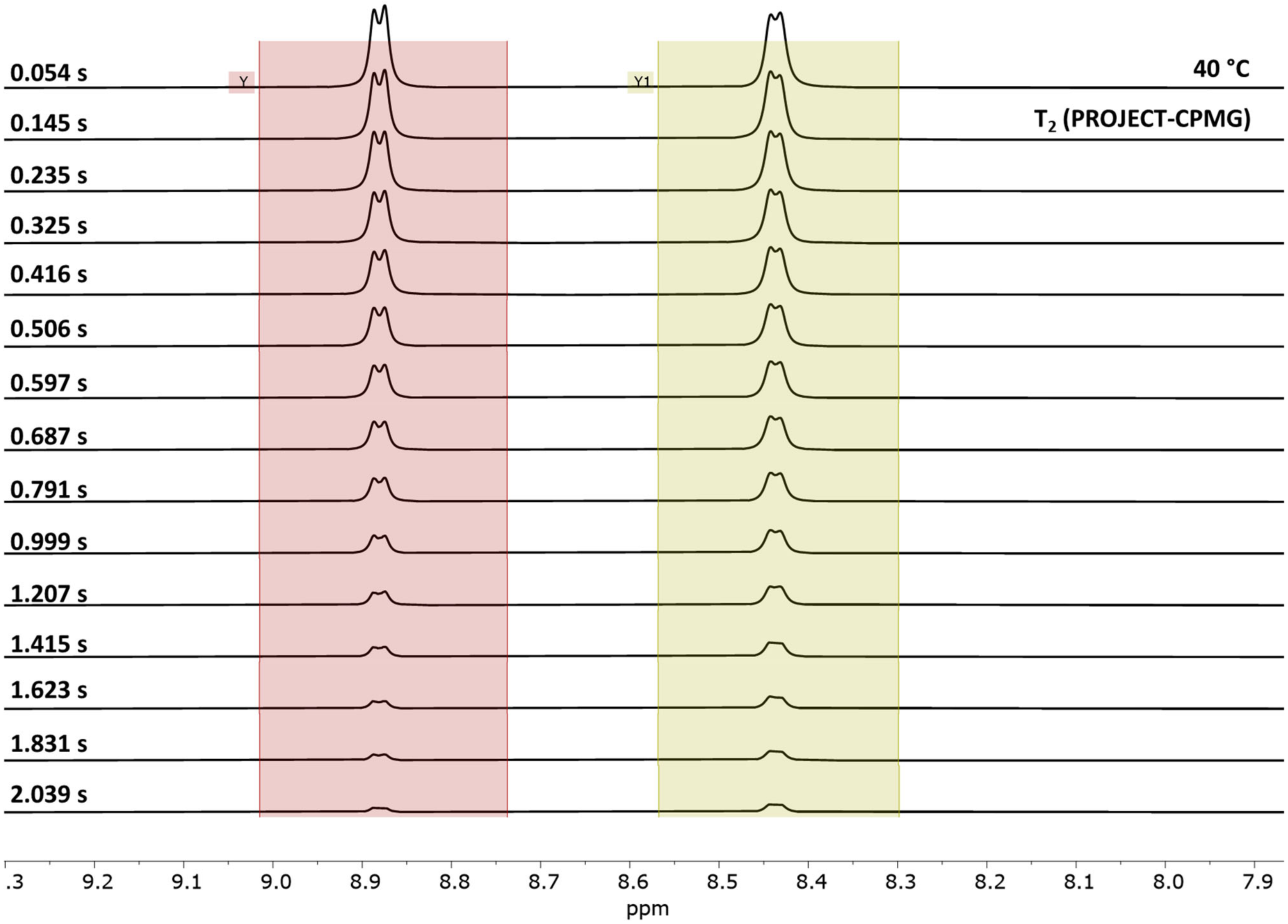

Supplementary Figure 159. T_2 (PROJECT-CPMG). Stacked spectra of the PROJECT-CPMG experiment to determine the T_2 values of the ortho (Y) and meta (Y1) protons of **MnRot** (0.5 mM) and **VP** (5 mM) in solution, in which the signal intensity (Y) is plotted against the time in seconds (X) (^{1}H, 500 MHz, chloroform-*d* : acetonitrile-*d*$_3$, 313 K).

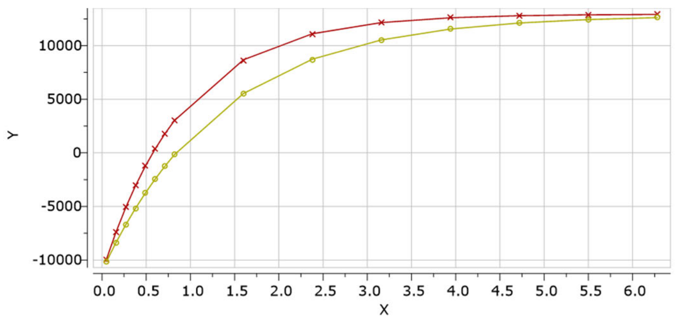

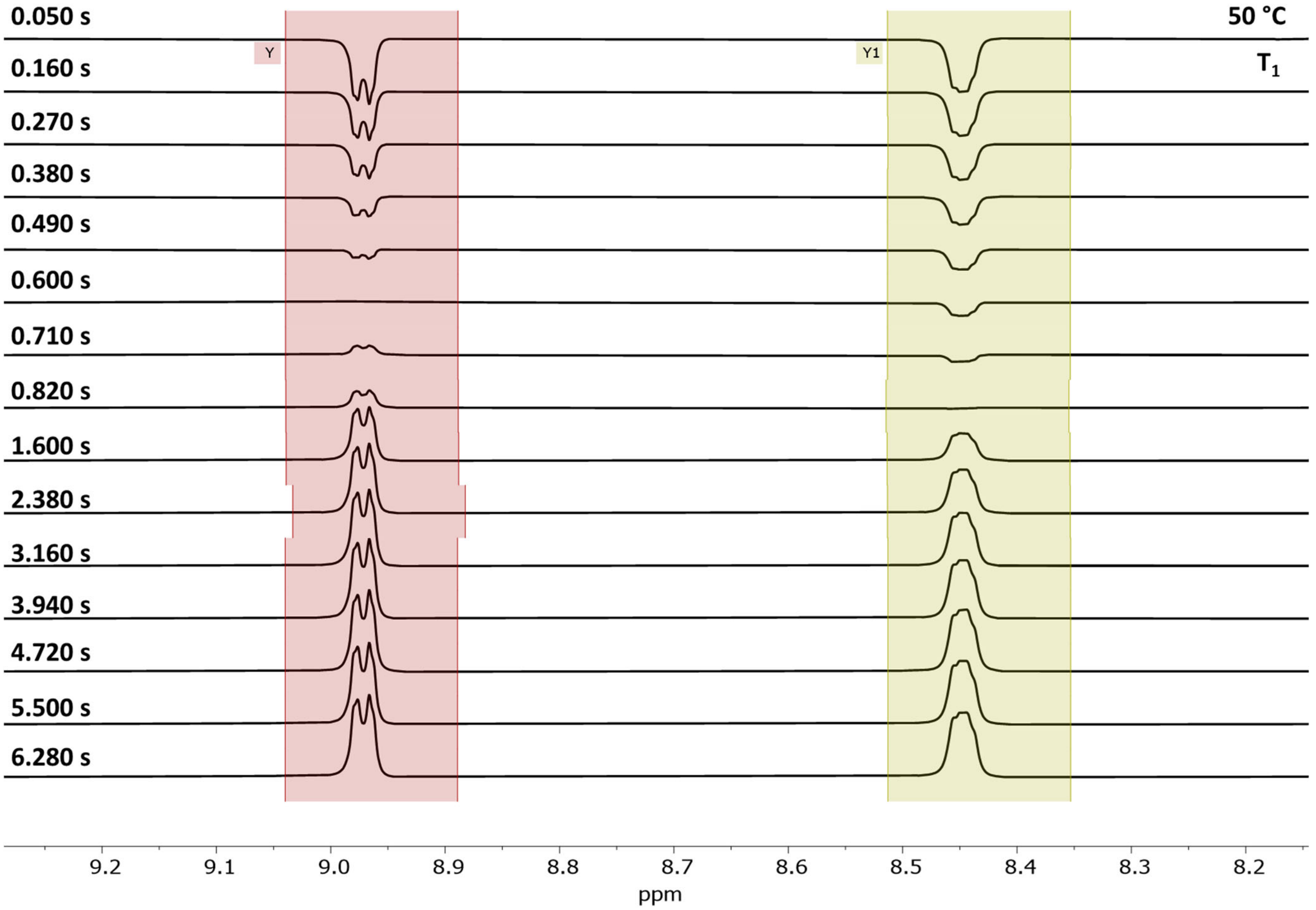

Supplementary Figure 160. T_1 (inverse recovery). Stacked spectra of the inverse recovery experiment to determine the T_1 values of the ortho (Y) and meta (Y1) protons of **MnRot** (0.5 mM) and **VP** (5 mM) in solution, in which the signal intensity (Y) is plotted against the time in seconds (X) (^{1}H, 500 MHz, chloroform-*d* : acetonitrile-*d*$_3$, 323 K).

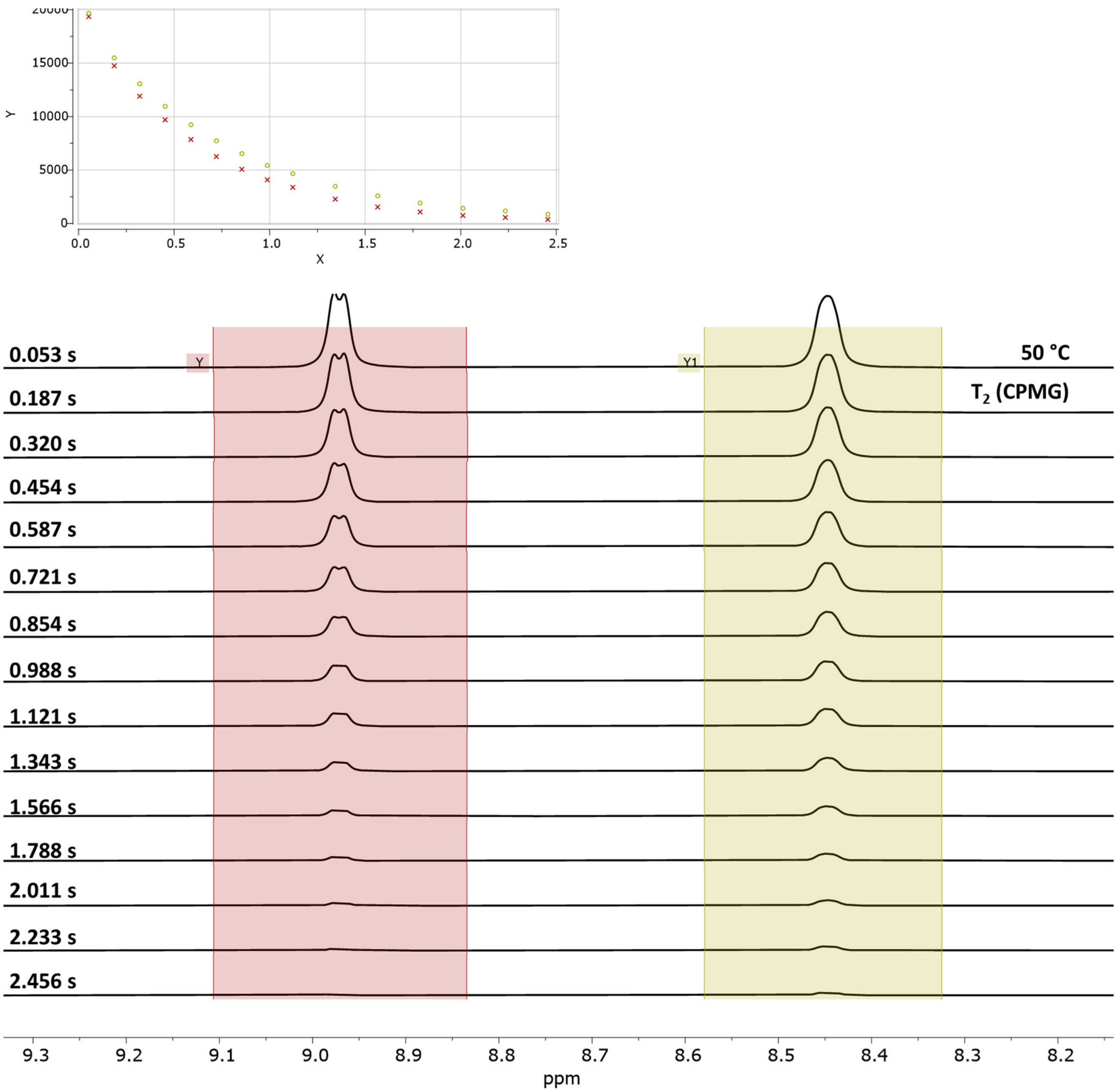

Supplementary Figure 161. T_2 (CPMG). Stacked spectra of the CPMG experiment to determine the T_2 values of the ortho (Y) and meta (Y1) protons of **MnRot** (0.5 mM) and **VP** (5 mM) in solution, in which the signal intensity (Y) is plotted against the time in seconds (X) (^{1}H, 500 MHz, chloroform-*d* : acetonitrile-*d*$_3$, 323 K).

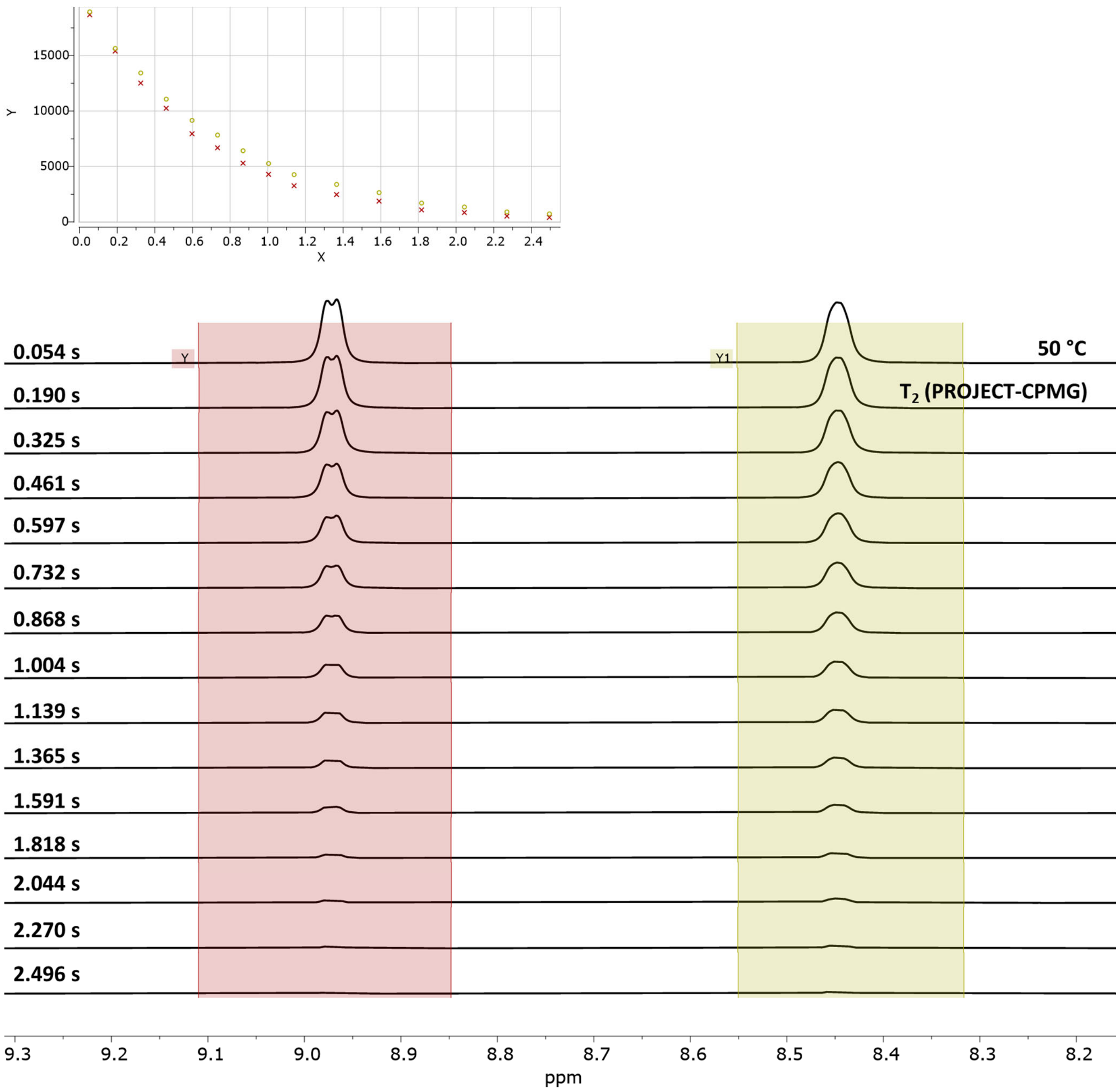

Supplementary Figure 162. T_2 (PROJECT-CPMG). Stacked spectra of the PROJECT-CPMG experiment to determine the T_2 values of the ortho (Y) and meta (Y1) protons of **MnRot** (0.5 mM) and **VP** (5 mM) in solution, in which the signal intensity (Y) is plotted against the time in seconds (X) (^{1}H, 500 MHz, chloroform-*d* : acetonitrile-*d*$_3$, 323 K).

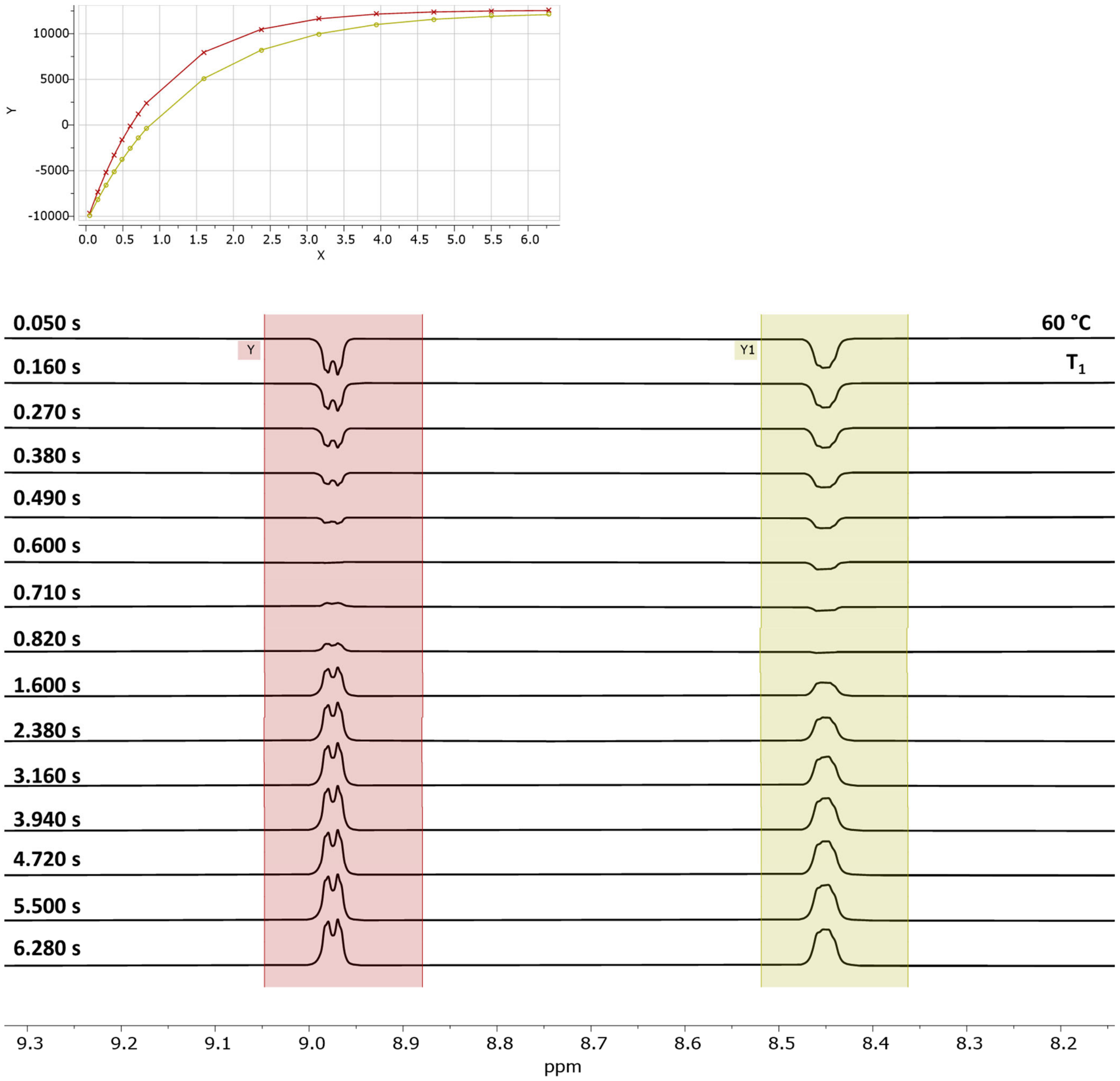

Supplementary Figure 163. T_1 (inverse recovery). Stacked spectra of the inverse recovery experiment to determine the T_1 values of the ortho (Y) and meta (Y1) protons of **MnRot** (0.5 mM) and **VP** (5 mM) in solution, in which the signal intensity (Y) is plotted against the time in seconds (X) (^{1}H, 500 MHz, chloroform-*d* : acetonitrile-*d*$_3$, 333 K).

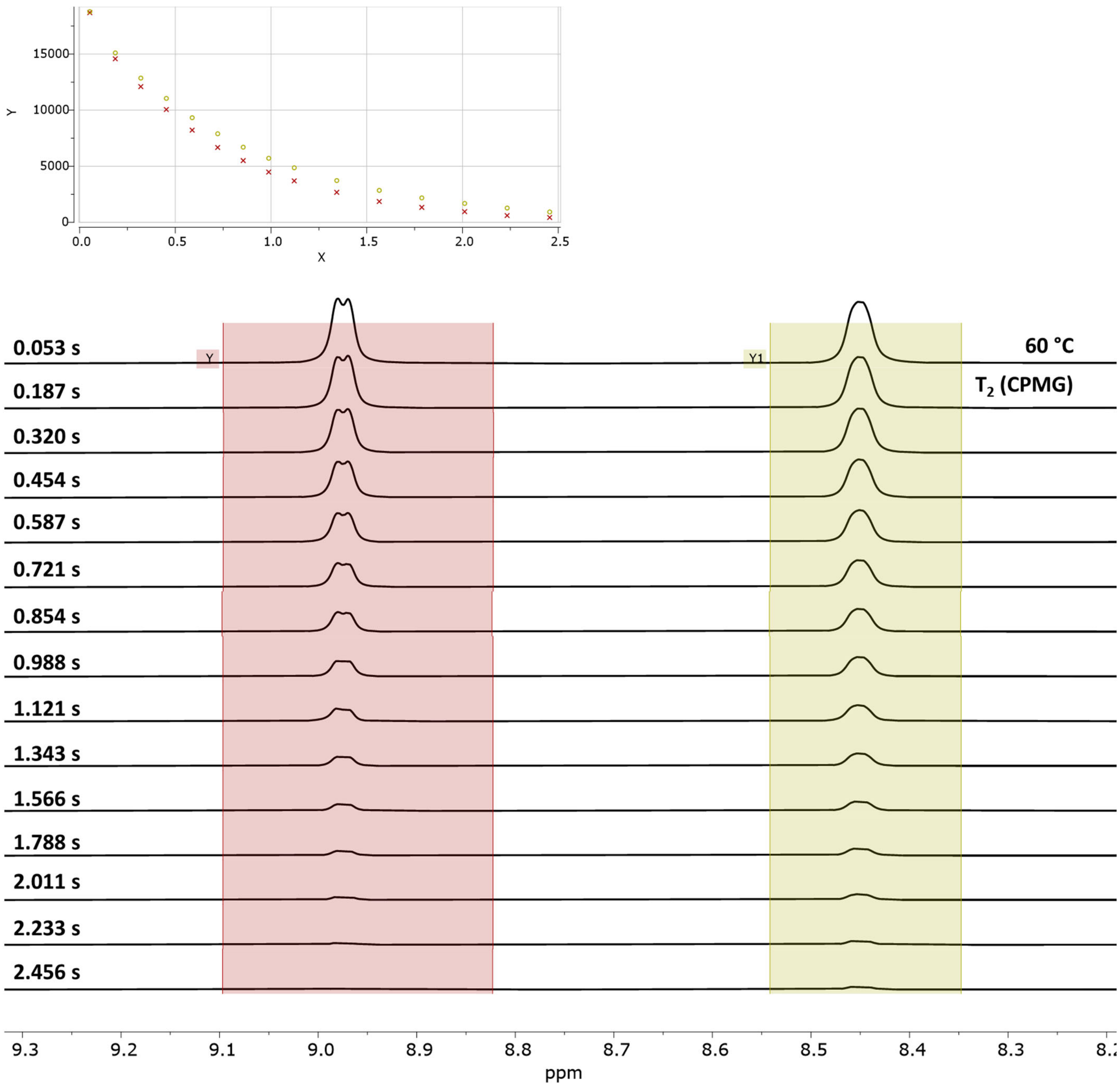

Supplementary Figure 164. T_2 (CPMG). Stacked spectra of the CPMG experiment to determine the T_2 values of the ortho (Y) and meta (Y1) protons of **MnRot** (0.5 mM) and **VP** (5 mM) in solution, in which the signal intensity (Y) is plotted against the time in seconds (X) (^{1}H, 500 MHz, chloroform-*d* : acetonitrile-*d*$_3$, 333 K).

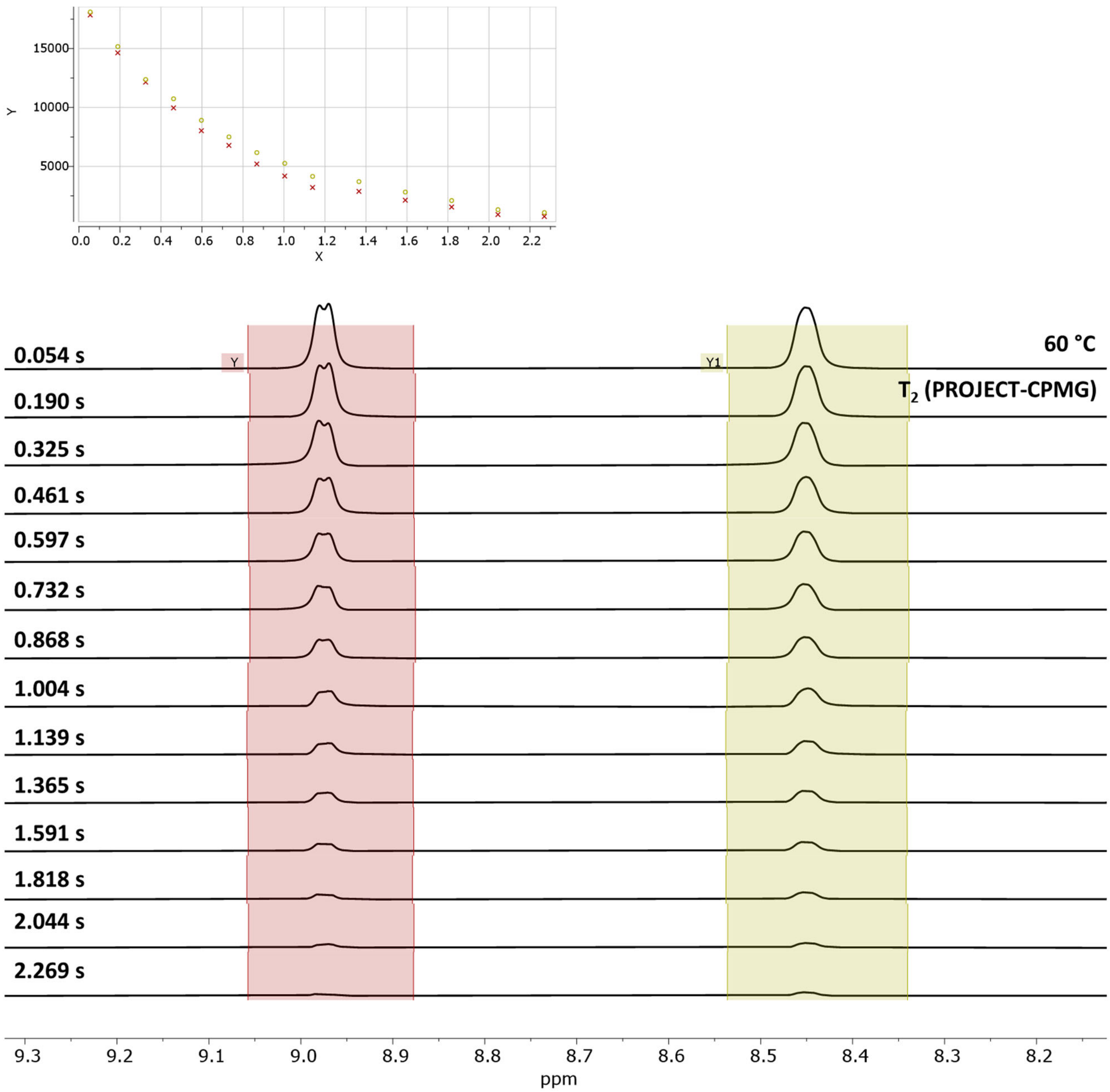

Supplementary Figure 165. T_2 (PROJECT-CPMG). Stacked spectra of the PROJECT-CPMG experiment to determine the T_2 values of the ortho (Y) and meta (Y1) protons of **MnRot** (0.5 mM) and **VP** (5 mM) in solution, in which the signal intensity (Y) is plotted against the time in seconds (X) (^{1}H, 500 MHz, chloroform-*d* : acetonitrile-*d*$_3$, 333 K).

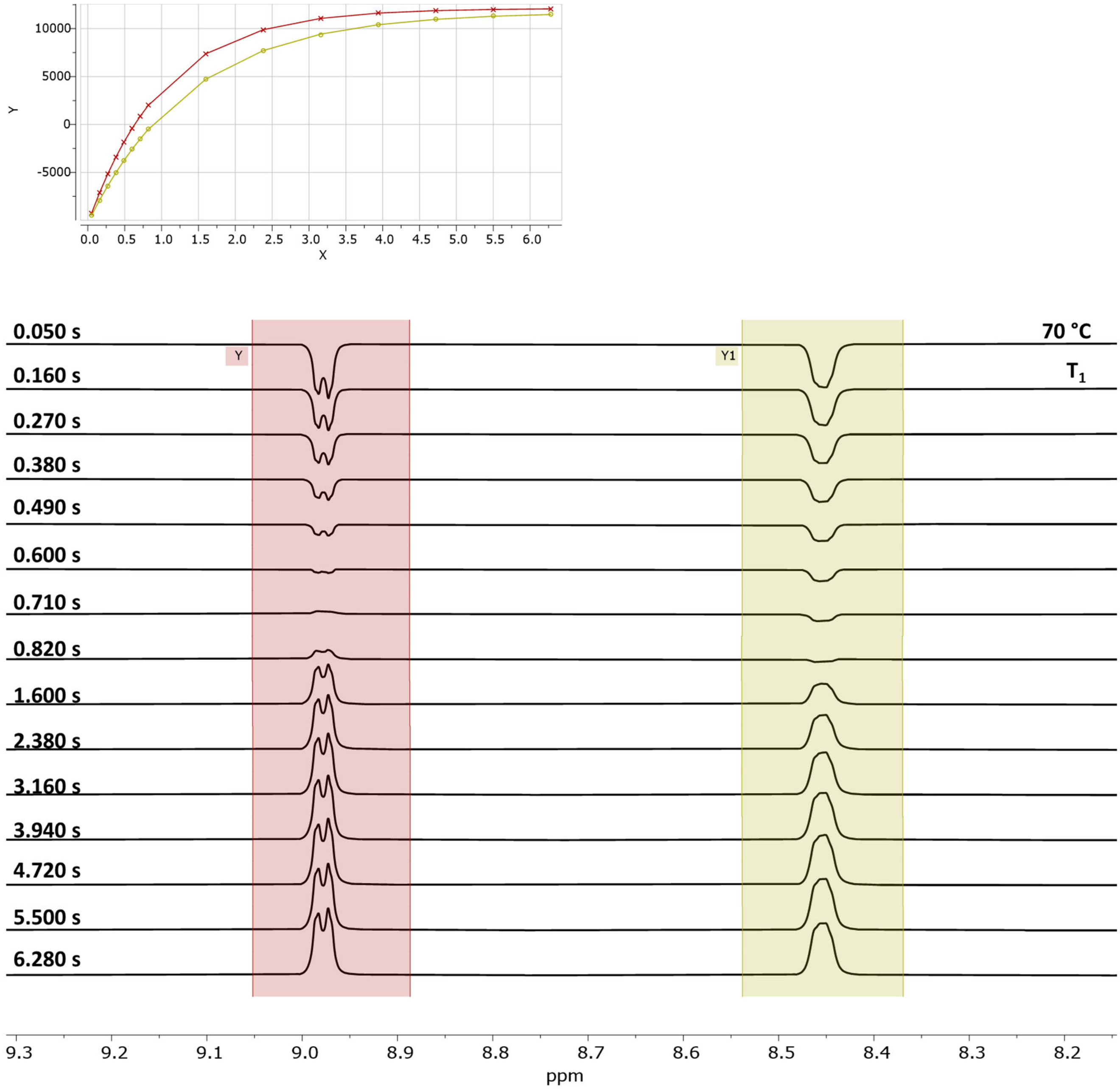

Supplementary Figure 166. T_1 (inverse recovery). Stacked spectra of the inverse recovery experiment to determine the T_1 values of the ortho (Y) and meta (Y1) protons of **MnRot** (0.5 mM) and **VP** (5 mM) in solution, in which the signal intensity (Y) is plotted against the time in seconds (X) (^{1}H, 500 MHz, chloroform-*d* : acetonitrile-*d$_3$*, 343 K).

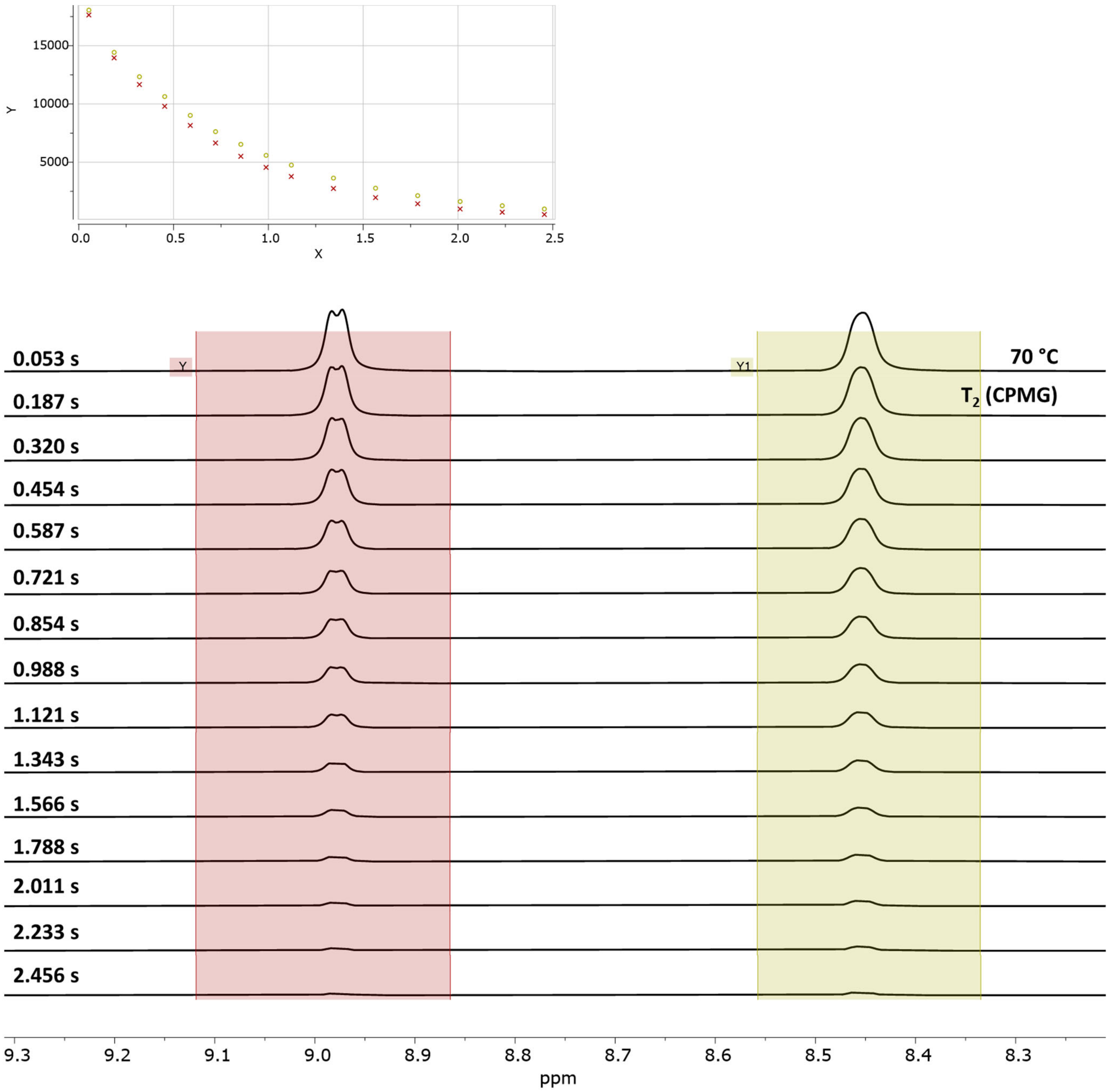

Supplementary Figure 167. T_2 (CPMG). Stacked spectra of the CPMG experiment to determine the T_2 values of the ortho (Y) and meta (Y1) protons of **MnRot** (0.5 mM) and **VP** (5 mM) in solution, in which the signal intensity (Y) is plotted against the time in seconds (X) (^{1}H, 500 MHz, chloroform-*d* : acetonitrile-*d*$_3$, 343 K).

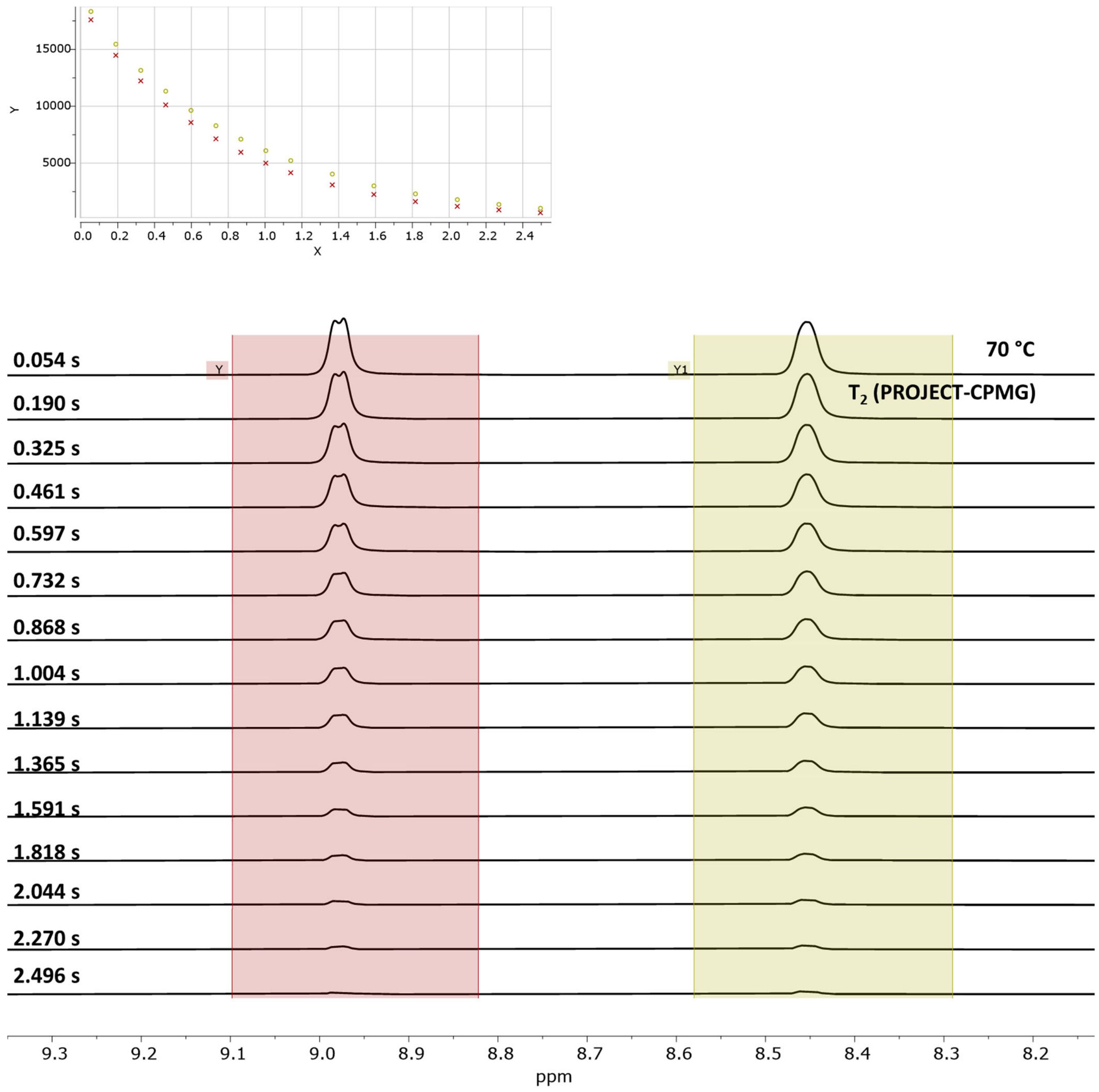

Supplementary Figure 168. T_2 (PROJECT-CPMG). Stacked spectra of the PROJECT-CPMG experiment to determine the T_2 values of the ortho (Y) and meta (Y1) protons of **MnRot** (0.5 mM) and **VP** (5 mM) in solution, in which the signal intensity (Y) is plotted against the time in seconds (X) (^{1}H, 500 MHz, chloroform-*d* : acetonitrile-*d*$_3$, 343 K).

4.7. V2 ($T_{1,0}$)

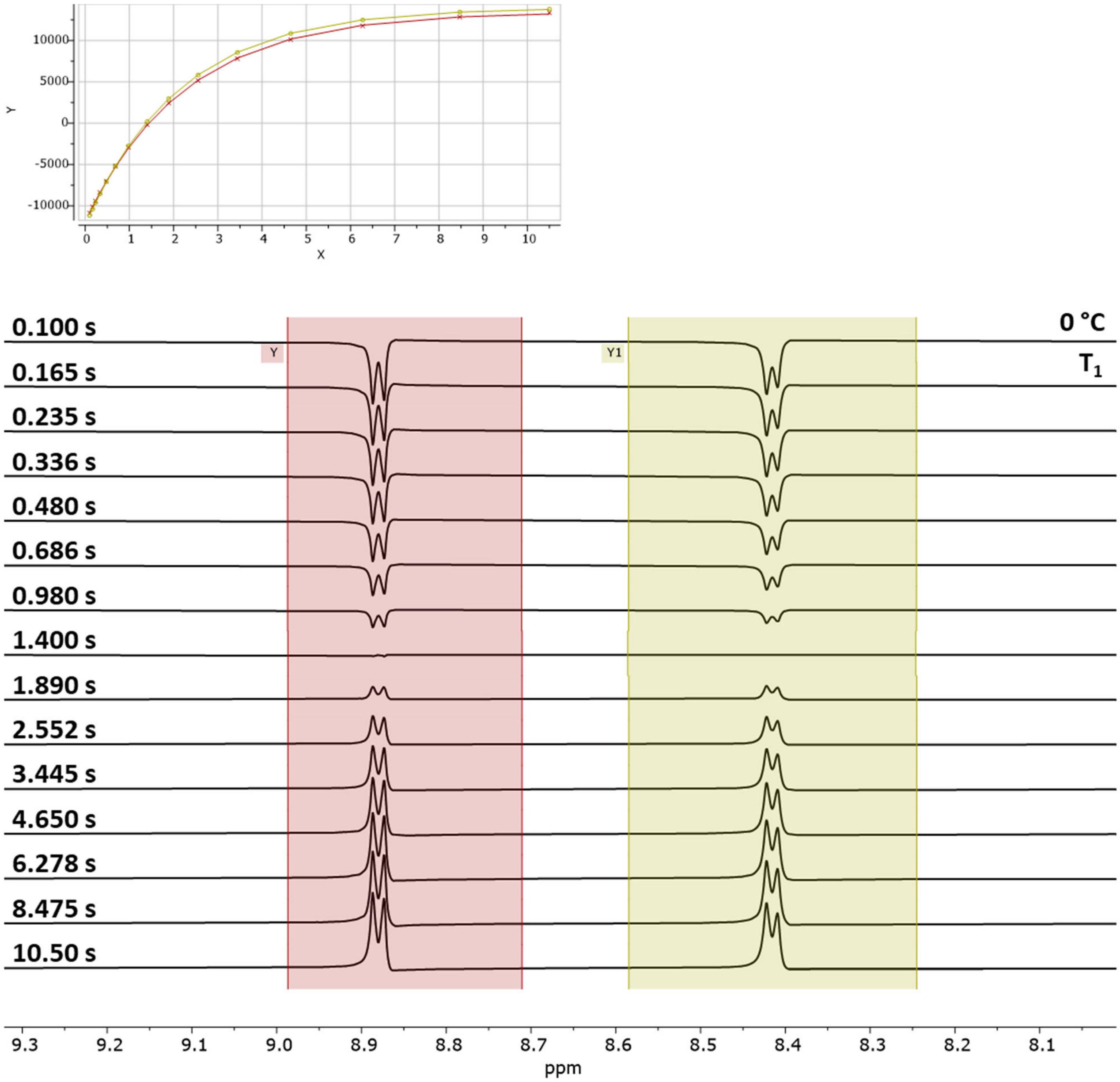

Supplementary Figure 169. T_1 (inverse recovery). Stacked spectra of the inverse recovery experiment to determine the T_1 values of the ortho (Y) and meta (Y1) protons of **V2** (5 mM) in solution, in which the signal intensity (Y) is plotted against the time in seconds (X) (^{1}H, 500 MHz, chloroform-*d* : acetonitrile-*d*$_3$, v/v/v, 273 K).

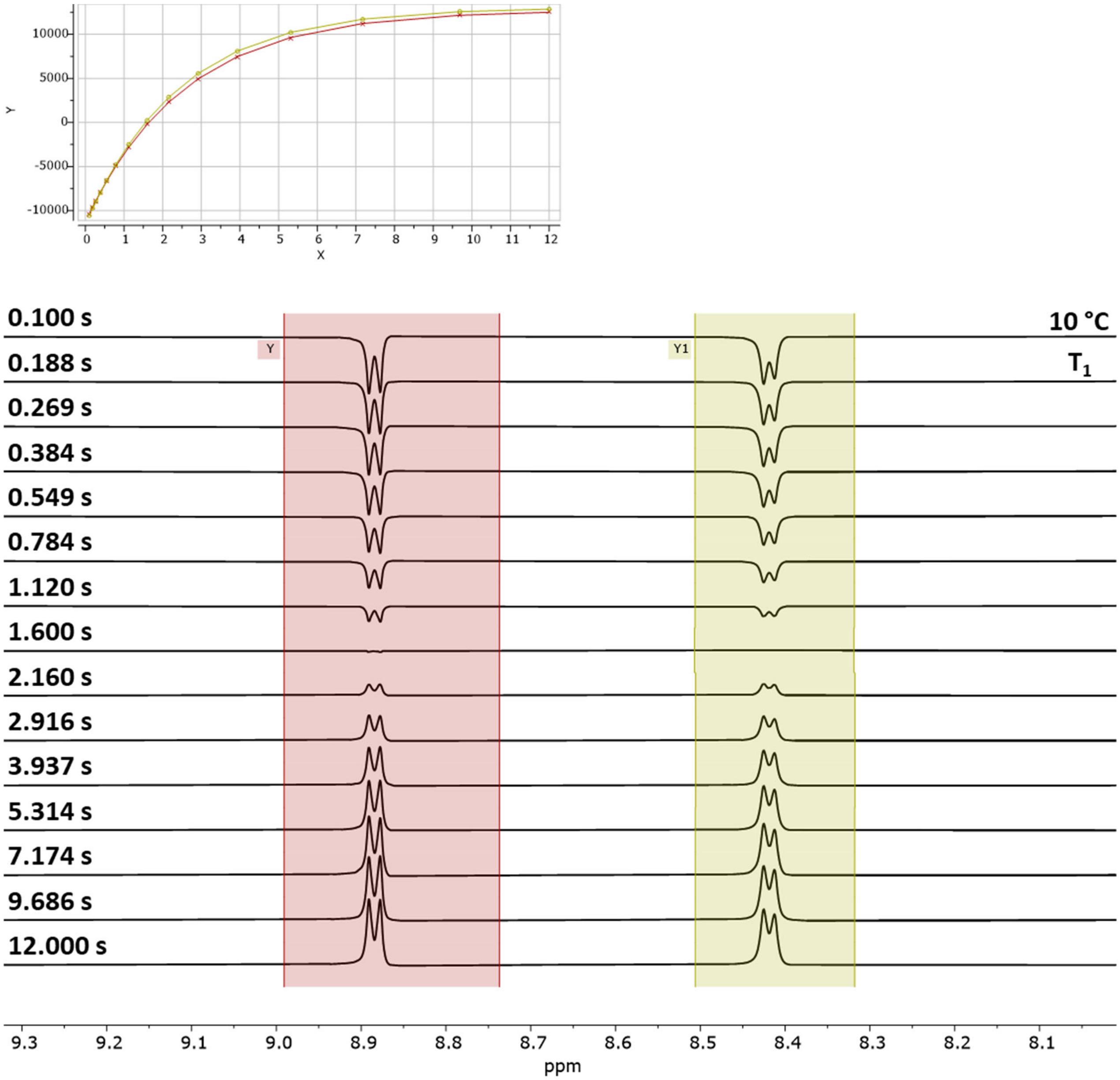

Supplementary Figure 170. T_1 (inverse recovery). Stacked spectra of the inverse recovery experiment to determine the T_1 values of the ortho (Y) and meta (Y1) protons of **V2** (5 mM) in solution, in which the signal intensity (Y) is plotted against the time in seconds (X) (^{1}H, 500 MHz, chloroform-*d* : acetonitrile-*d*$_3$, v/v/v, 283 K).

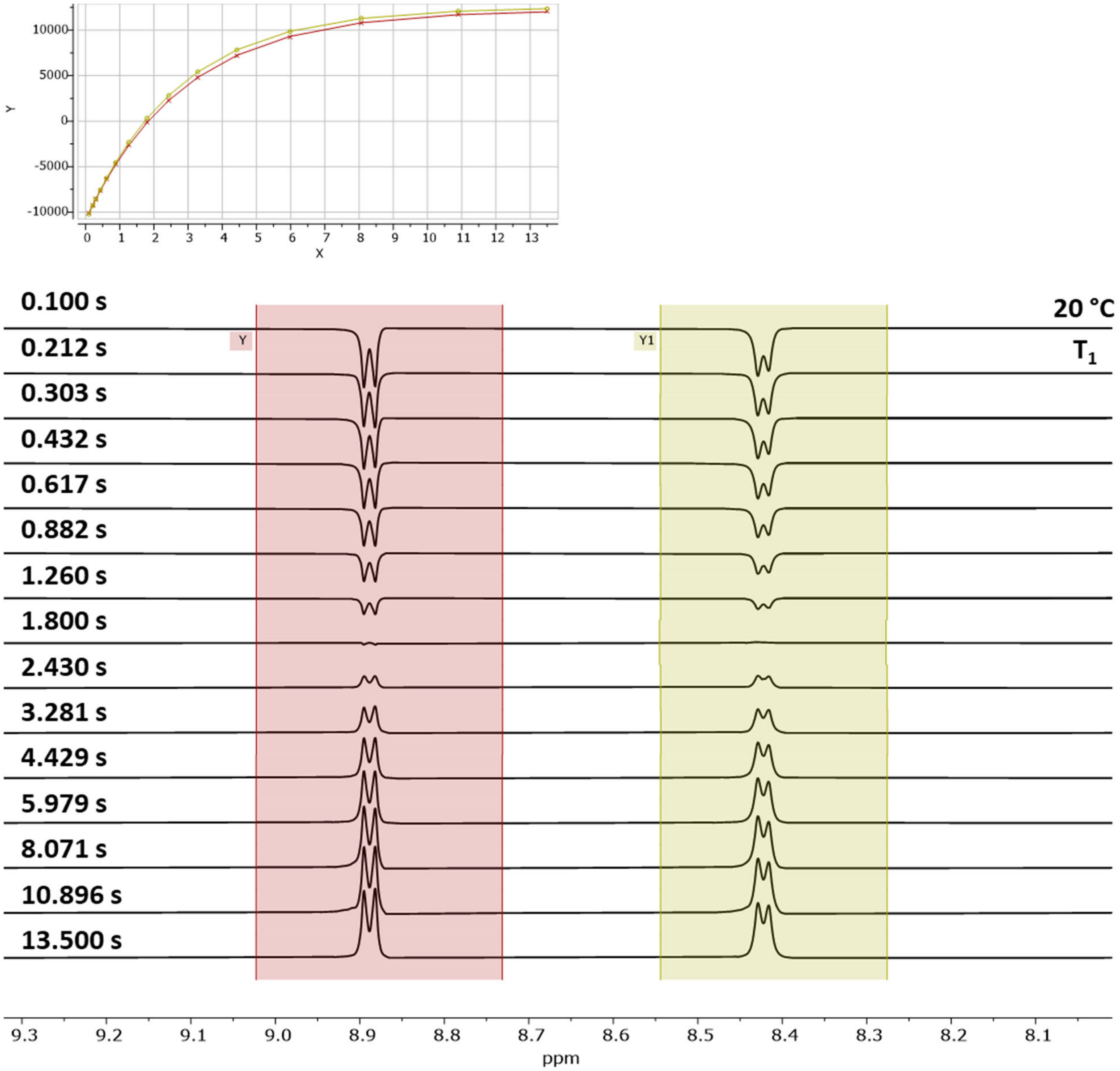

Supplementary Figure 171. T_1 (inverse recovery). Stacked spectra of the inverse recovery experiment to determine the T_1 values of the ortho (Y) and meta (Y1) protons of **V2** (5 mM) in solution, in which the signal intensity (Y) is plotted against the time in seconds (X) (^{1}H, 500 MHz, chloroform-*d* : acetonitrile-*d*$_3$, v/v/v, 293 K).

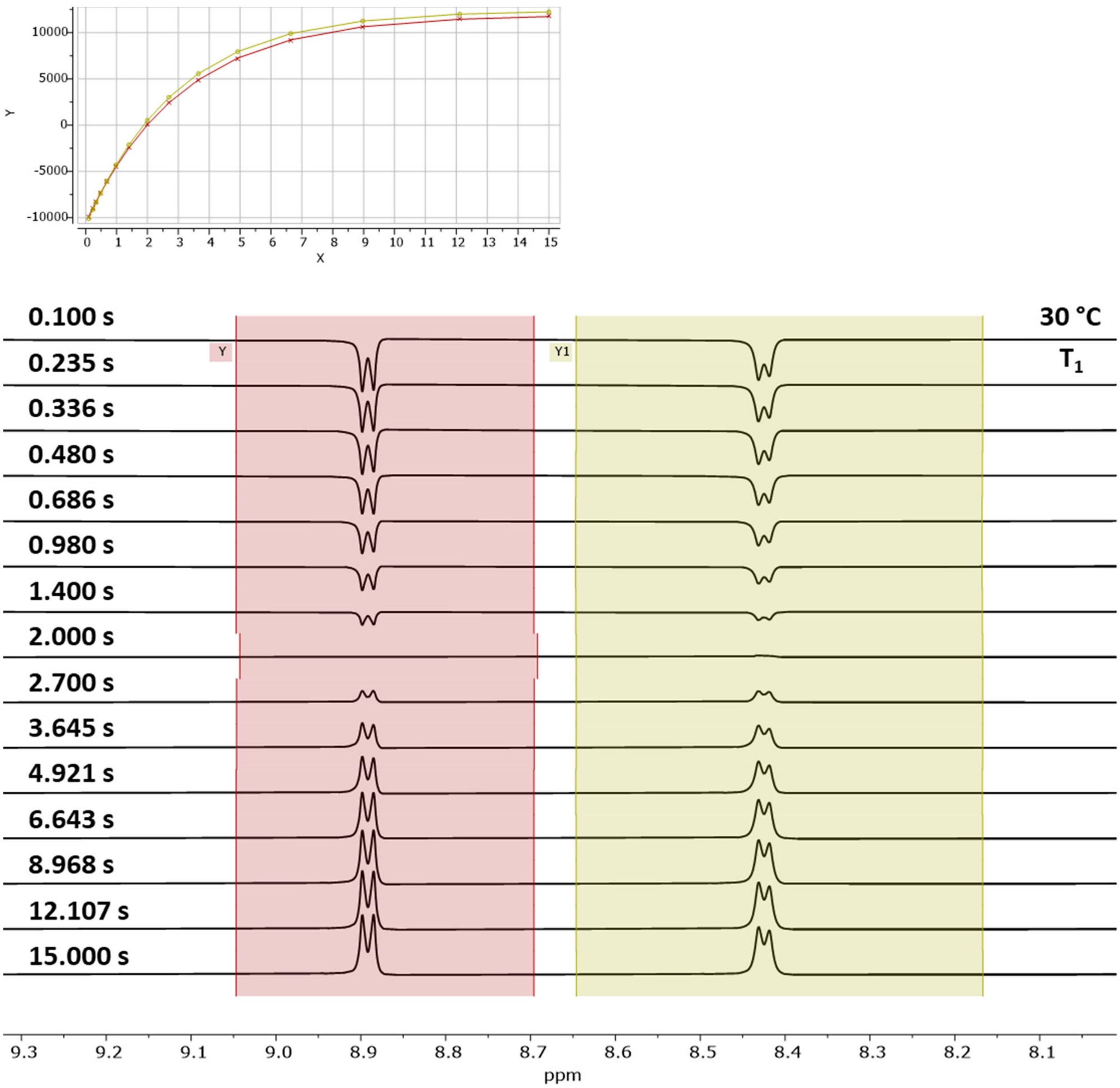

Supplementary Figure 172. T_1 (inverse recovery). Stacked spectra of the inverse recovery experiment to determine the T_1 values of the ortho (Y) and meta (Y1) protons of **V2** (5 mM) in solution, in which the signal intensity (Y) is plotted against the time in seconds (X) (^{1}H, 500 MHz, chloroform-*d* : acetonitrile-d_3, v/v/v, 303 K).

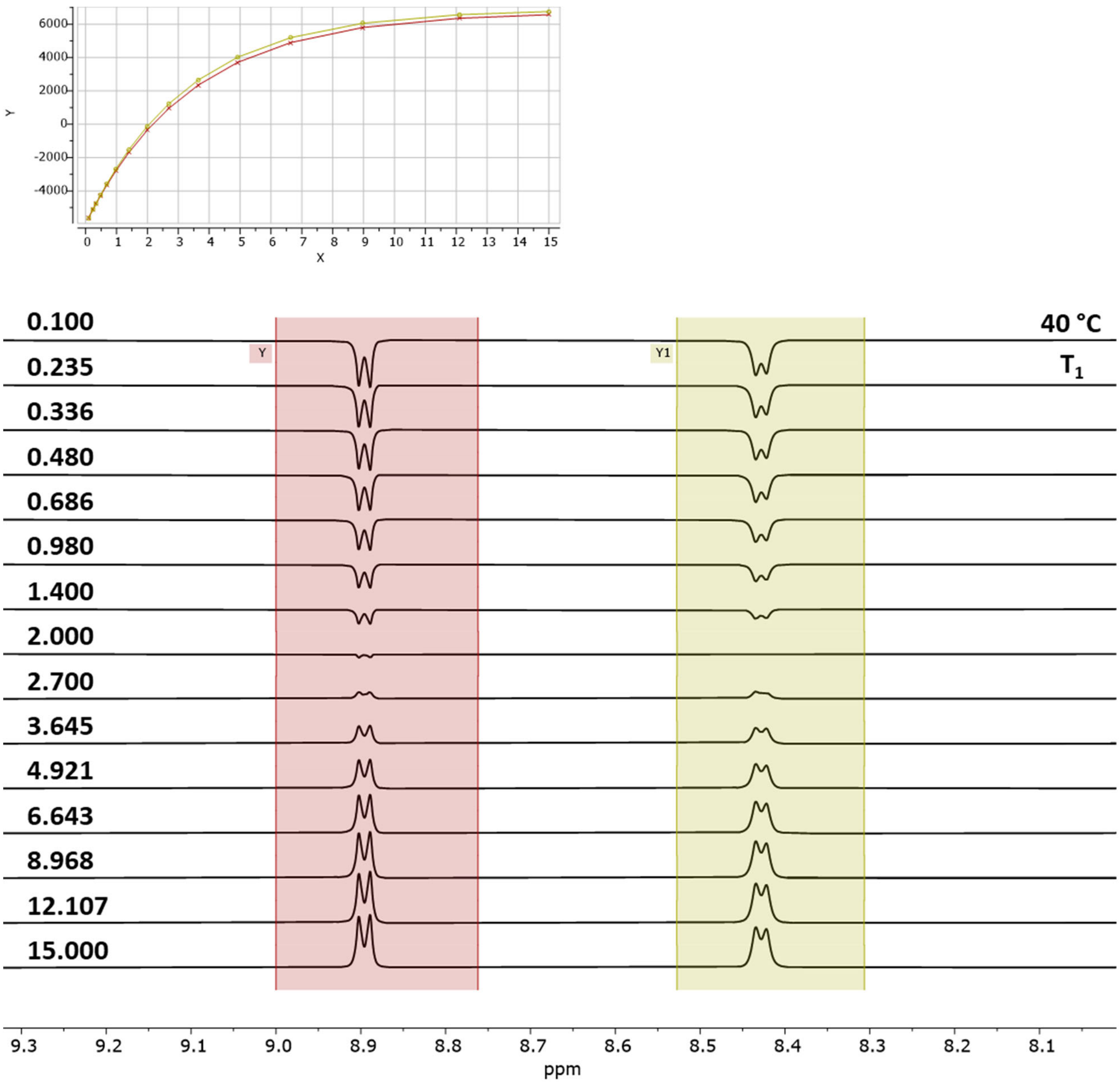

Supplementary Figure 173. T_1 (inverse recovery). Stacked spectra of the inverse recovery experiment to determine the T_1 values of the ortho (Y) and meta (Y1) protons of **V2** (5 mM) in solution, in which the signal intensity (Y) is plotted against the time in seconds (X) (^{1}H, 500 MHz, chloroform-*d* : acetonitrile-*d*$_3$, v/v/v, 313 K).

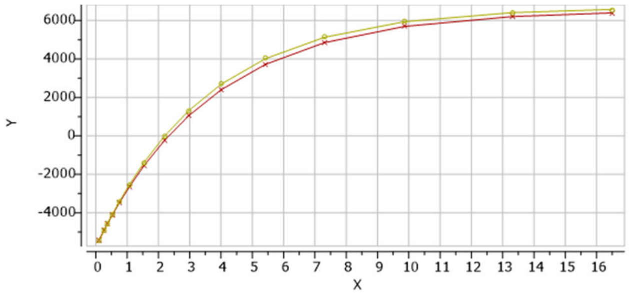

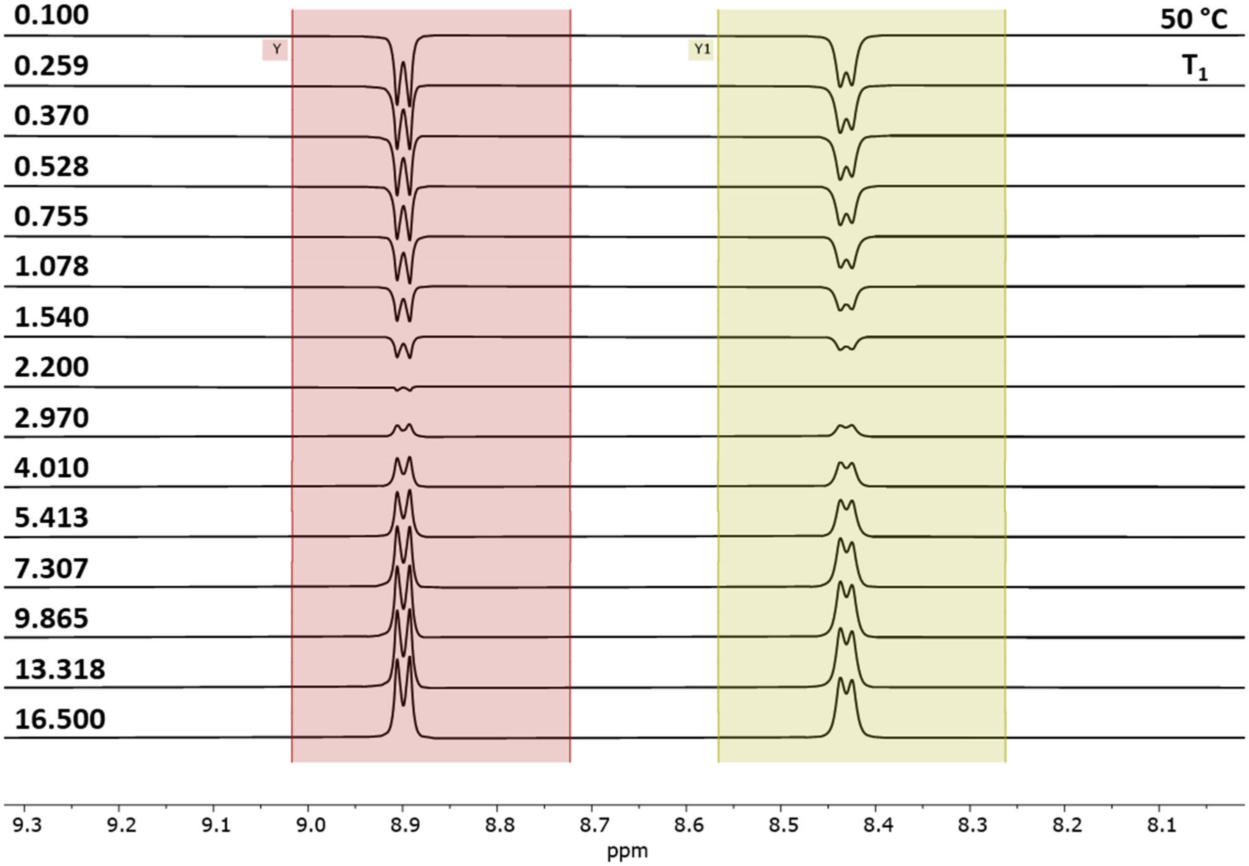

Supplementary Figure 174. T_1 (inverse recovery). Stacked spectra of the inverse recovery experiment to determine the T_1 values of the ortho (Y) and meta (Y1) protons of **V2** (5 mM) in solution, in which the signal intensity (Y) is plotted against the time in seconds (X) (^{1}H, 500 MHz, chloroform-*d* : acetonitrile-*d*$_3$, v/v/v, 323 K).

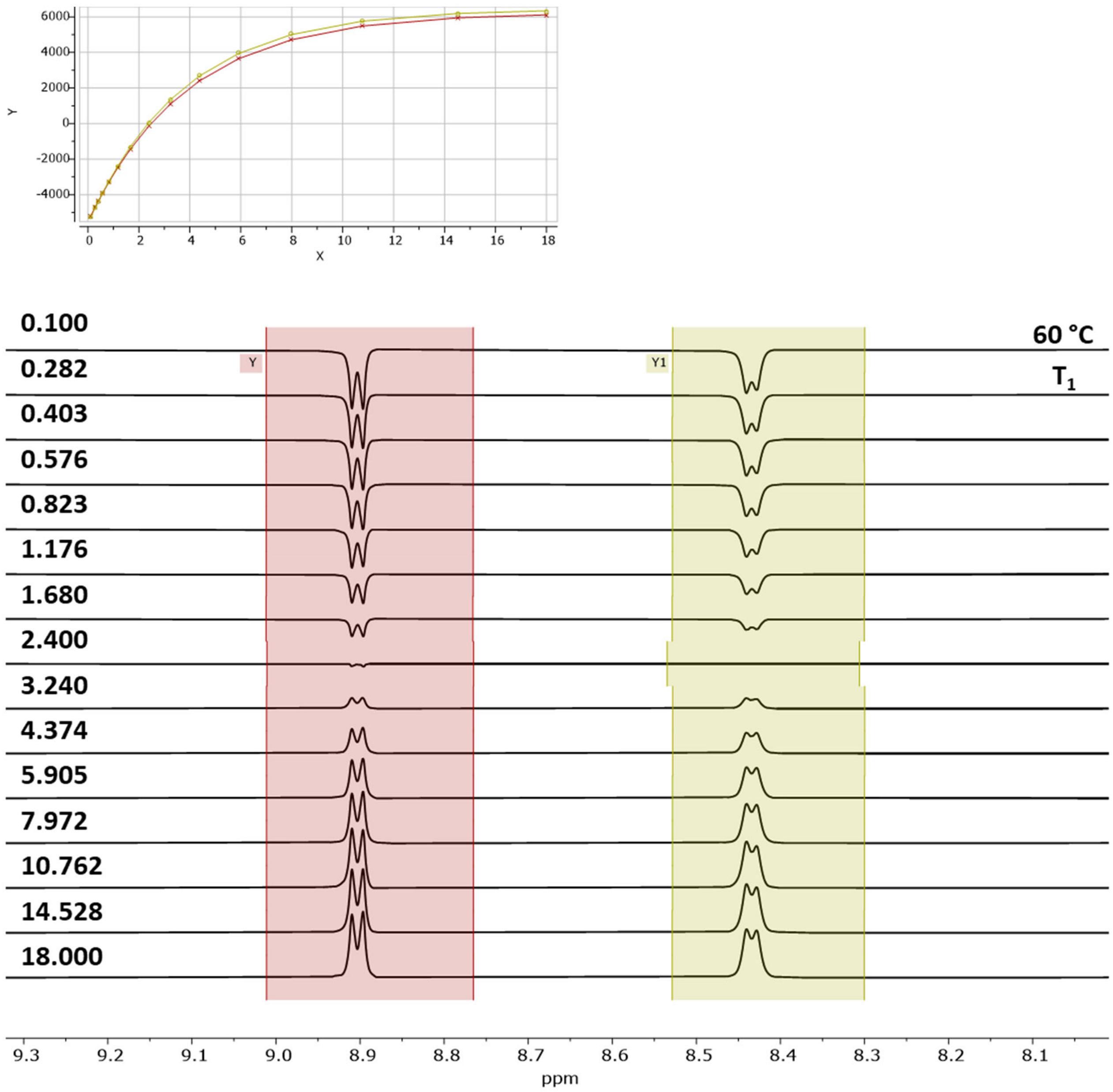

Supplementary Figure 175. T_1 (inverse recovery). Stacked spectra of the inverse recovery experiment to determine the T_1 values of the ortho (Y) and meta (Y1) protons of **V2** (5 mM) in solution, in which the signal intensity (Y) is plotted against the time in seconds (X) (^{1}H, 500 MHz, chloroform-*d* : acetonitrile-*d*$_3$, v/v/v, 333 K).

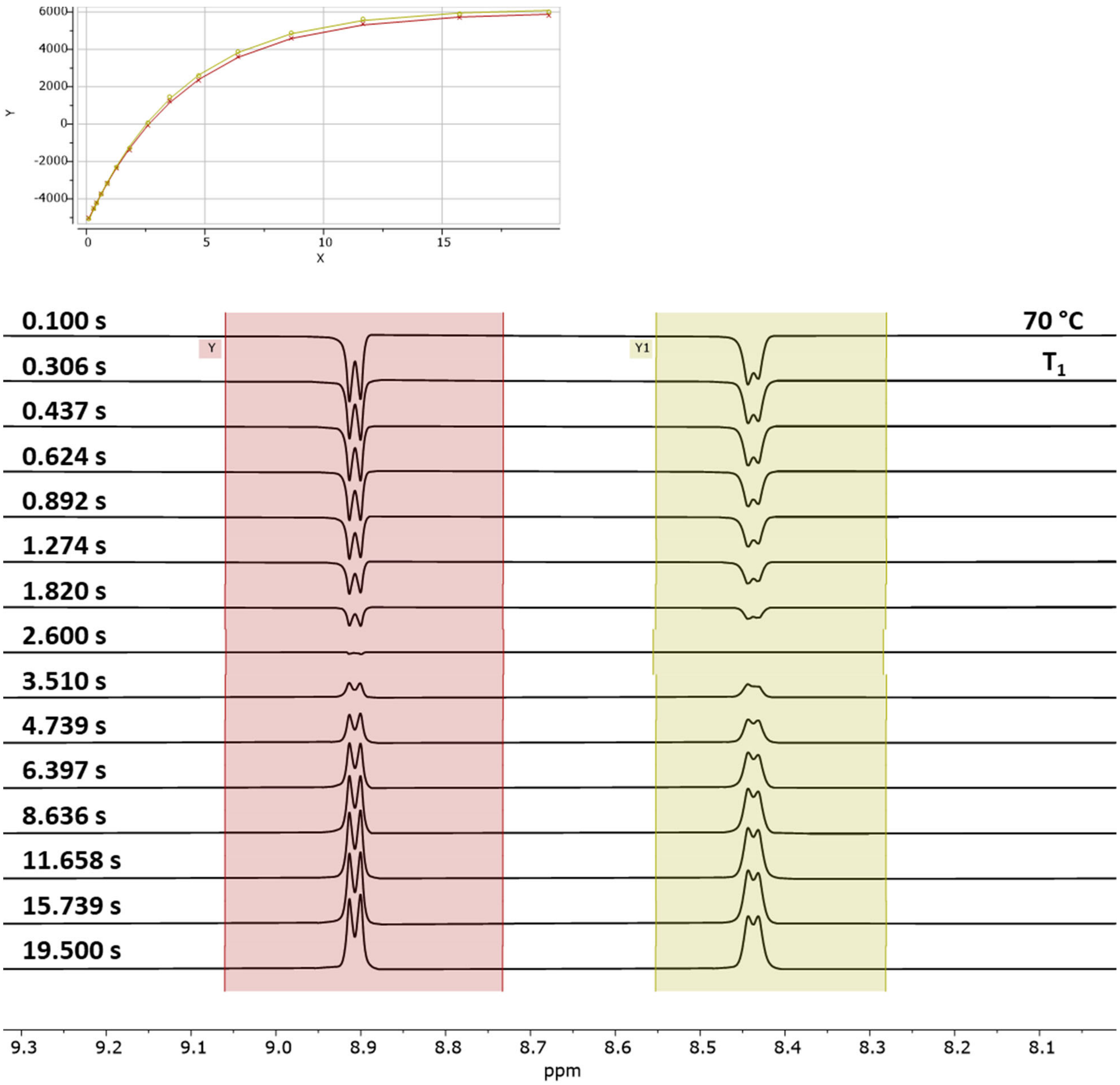

Supplementary Figure 176. T_1 (inverse recovery). Stacked spectra of the inverse recovery experiment to determine the T_1 values of the ortho (Y) and meta (Y1) protons of **V2** (5 mM) in solution, in which the signal intensity (Y) is plotted against the time in seconds (X) (^{1}H, 500 MHz, chloroform-*d* : acetonitrile-*d*$_3$, v/v/v, 343 K).

4.8. Mn1/V2 ($T_{1,obs}$)

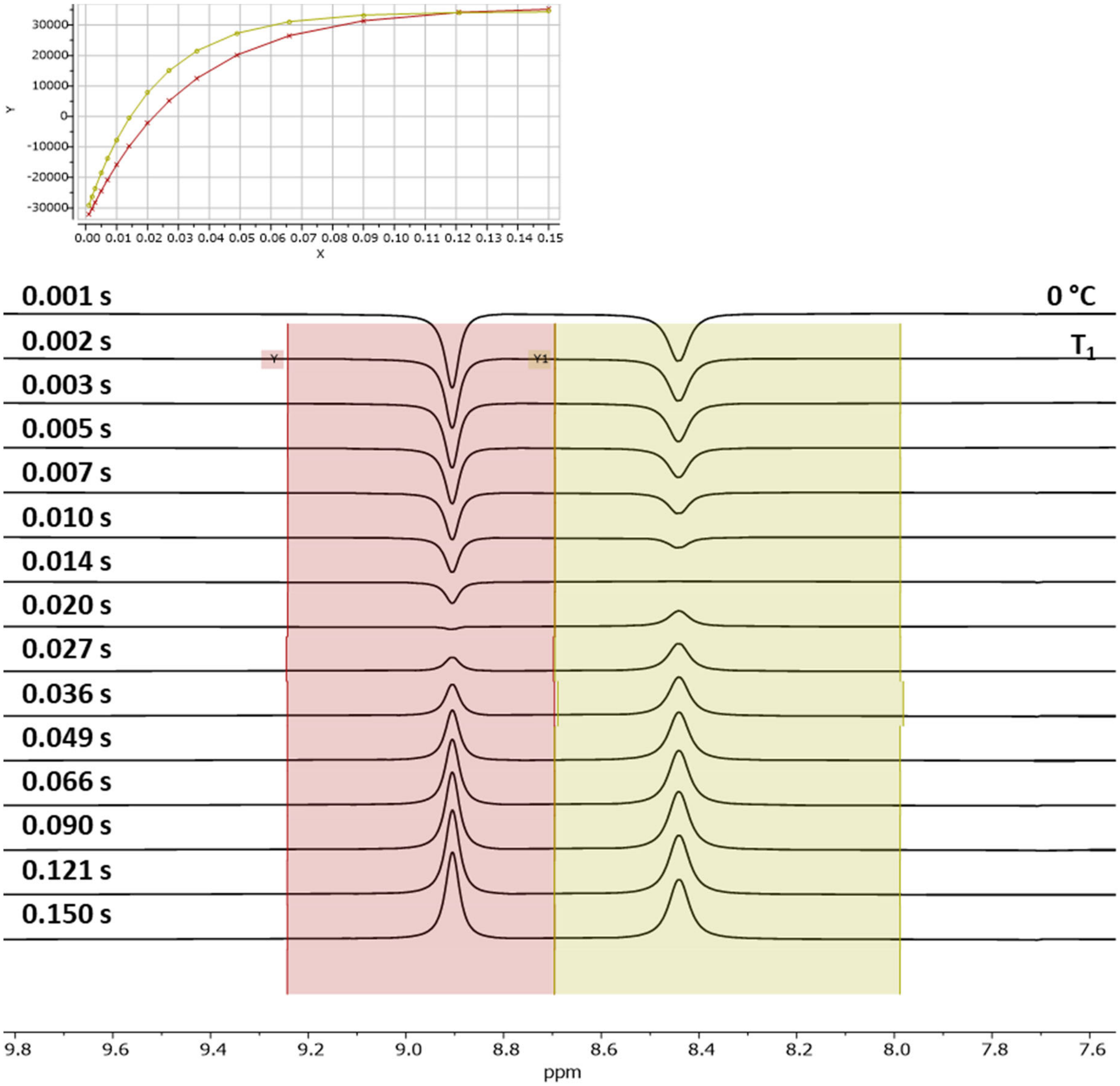

Supplementary Figure 177. T_1 (inverse recovery). Stacked spectra of the inverse recovery experiment to determine the T_1 values of the ortho (Y) and meta (Y1) protons of **Mn1** (0.05) **V2** (5 mM) in solution, in which the signal intensity (Y) is plotted against the time in seconds (X) (^{1}H, 500 MHz, chloroform-*d* : acetonitrile-d_3, v/v/v, 273 K).

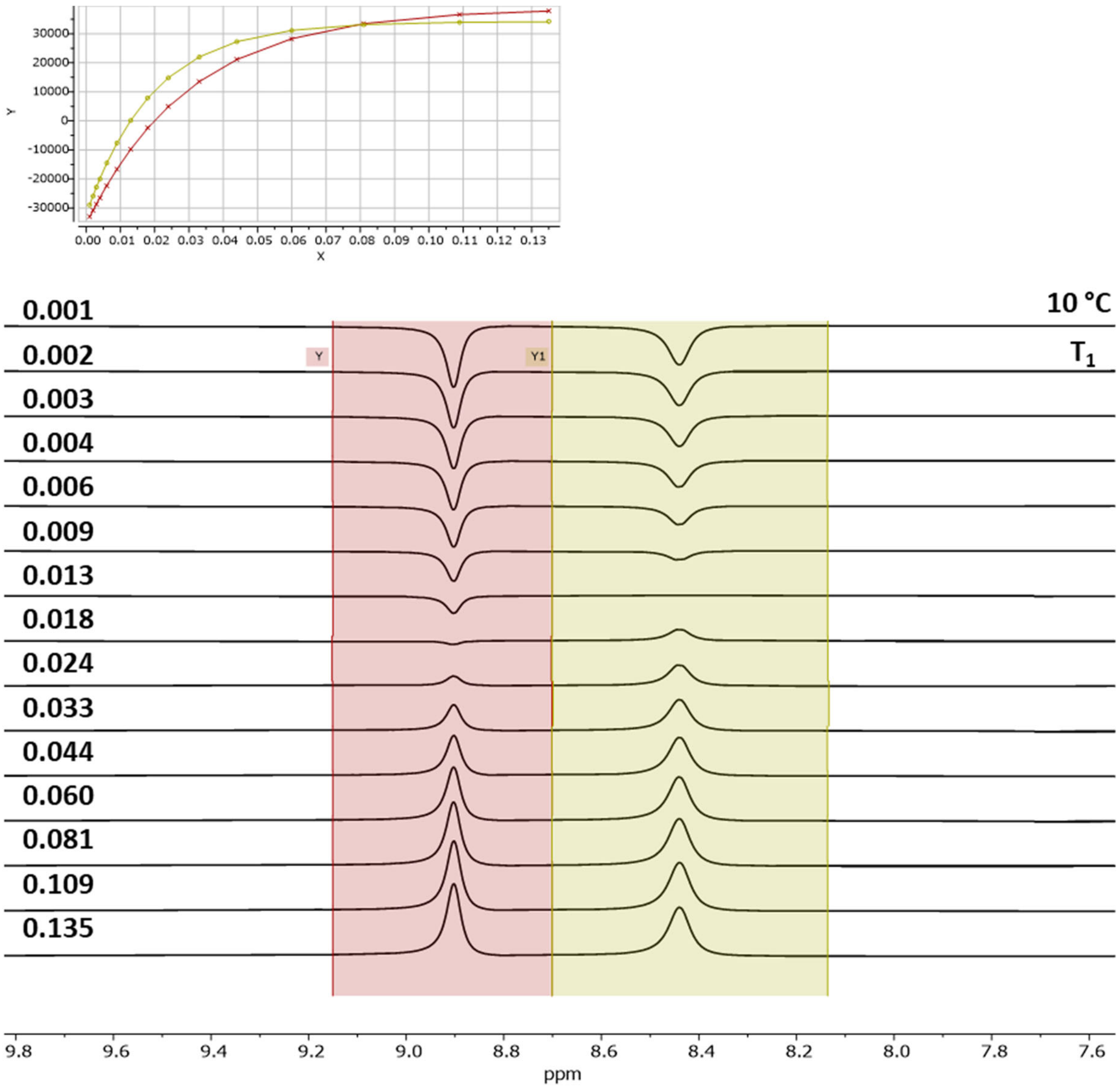

Supplementary Figure 178. T_1 (inverse recovery). Stacked spectra of the inverse recovery experiment to determine the T_1 values of the ortho (Y) and meta (Y1) protons of **Mn1** (0.05) **V2** (5 mM) in solution, in which the signal intensity (Y) is plotted against the time in seconds (X) (^{1}H, 500 MHz, chloroform-*d* : acetonitrile-d_3, v/v/v, 283 K).

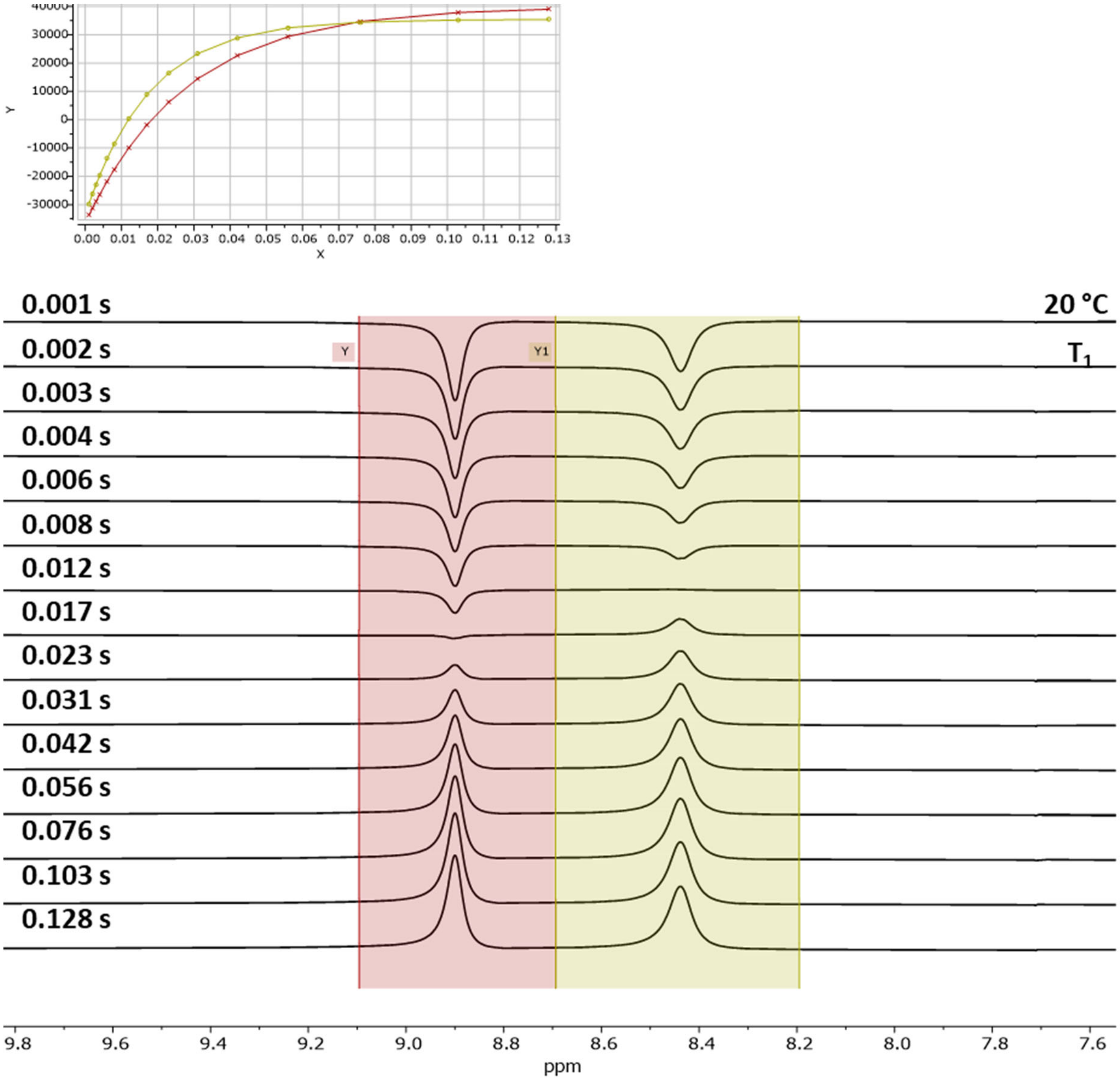

Supplementary Figure 179. T_1 (inverse recovery). Stacked spectra of the inverse recovery experiment to determine the T_1 values of the ortho (Y) and meta (Y1) protons of **Mn1** (0.05) **V2** (5 mM) in solution, in which the signal intensity (Y) is plotted against the time in seconds (X) (^{1}H, 500 MHz, chloroform-*d* : acetonitrile-*d*$_3$, v/v/v, 293 K).

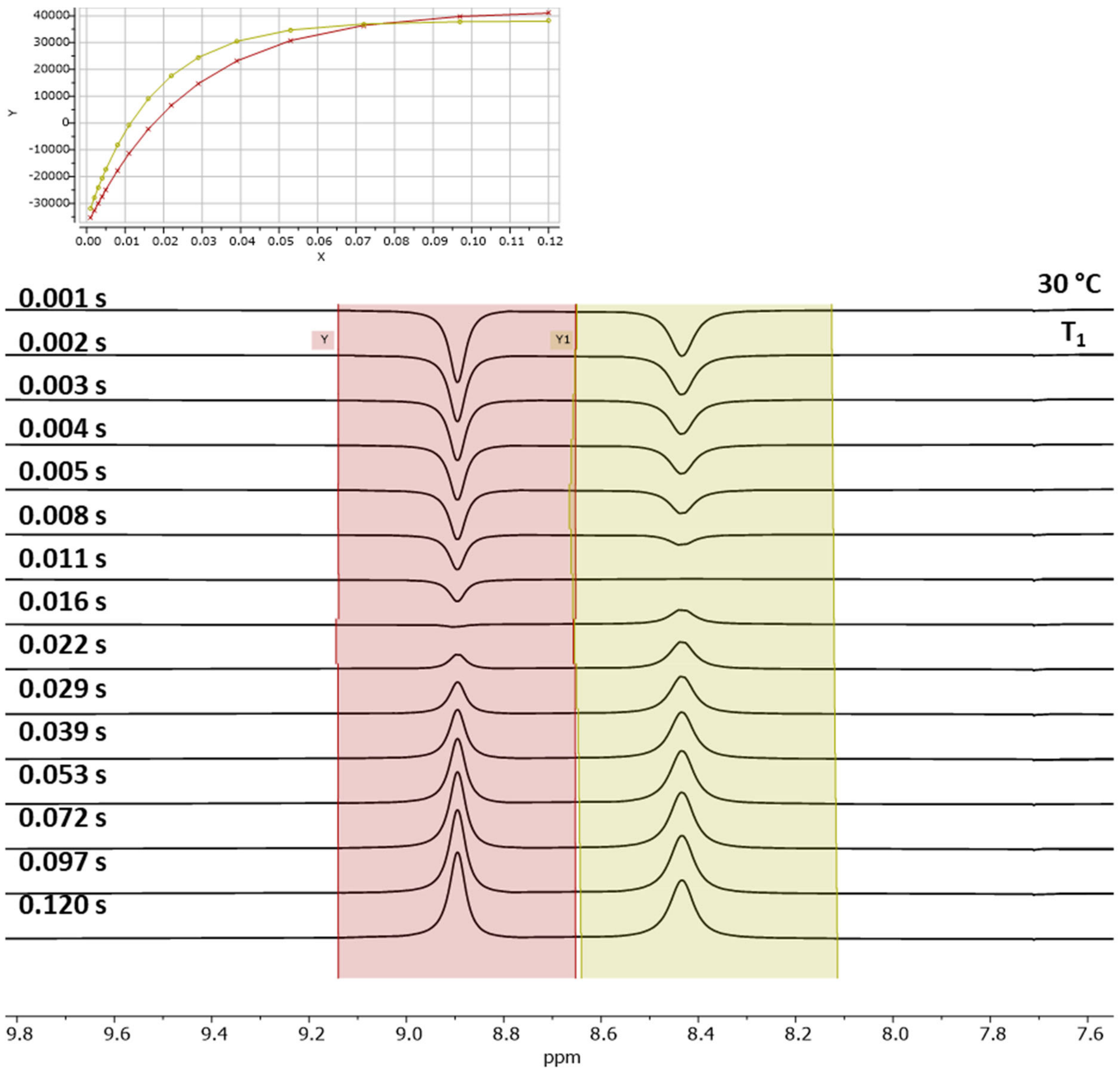

Supplementary Figure 180. T_1 (inverse recovery). Stacked spectra of the inverse recovery experiment to determine the T_1 values of the ortho (Y) and meta (Y1) protons of **Mn1** (0.05) **V2** (5 mM) in solution, in which the signal intensity (Y) is plotted against the time in seconds (X) (^{1}H, 500 MHz, chloroform-*d* : acetonitrile-*d₃*, v/v/v, 303 K).

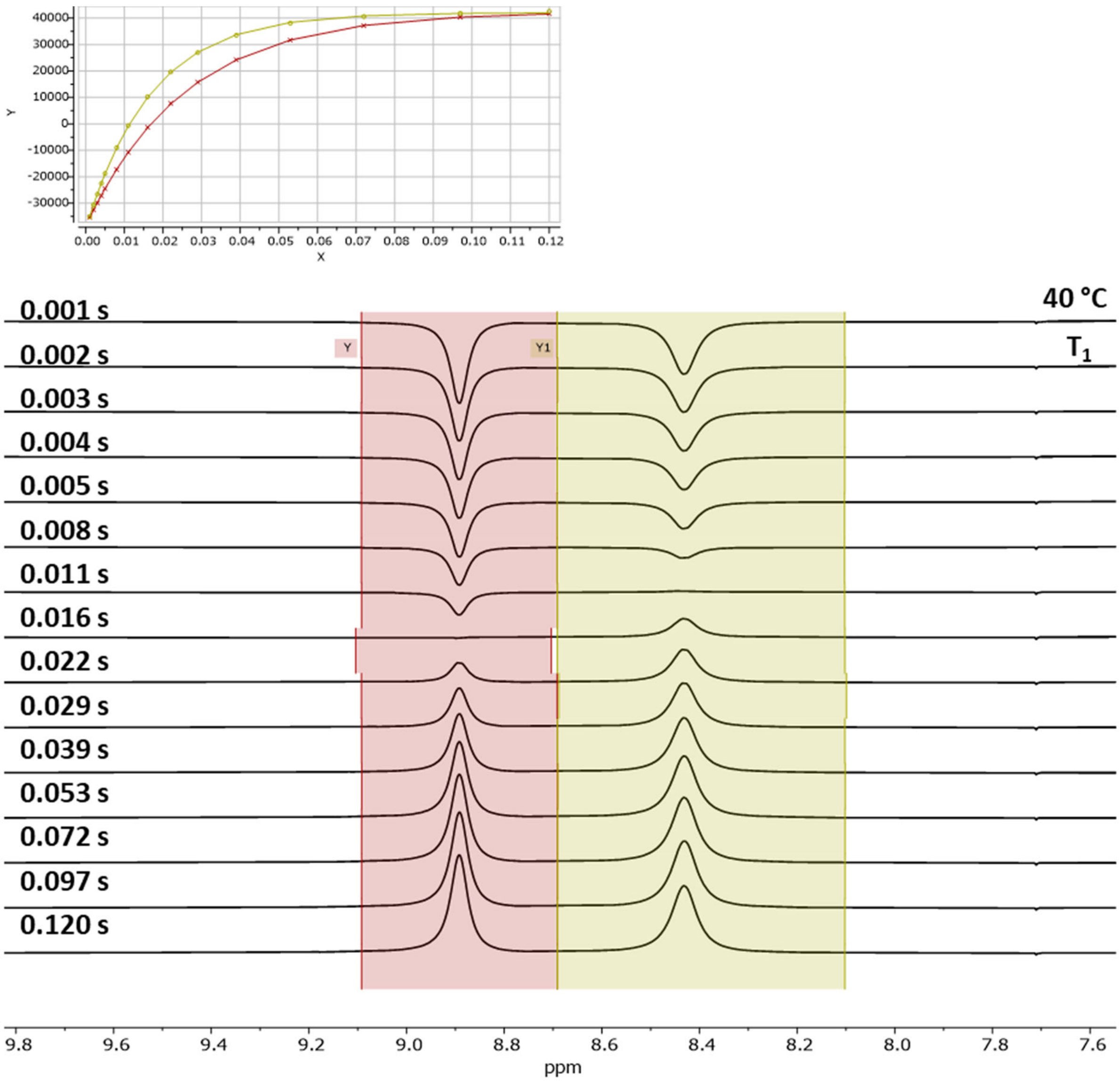

Supplementary Figure 181. T_1 (inverse recovery). Stacked spectra of the inverse recovery experiment to determine the T_1 values of the ortho (Y) and meta (Y1) protons of **Mn1** (0.05) **V2** (5 mM) in solution, in which the signal intensity (Y) is plotted against the time in seconds (X) (^{1}H, 500 MHz, chloroform-*d* : acetonitrile-d_3, v/v/v, 313 K).

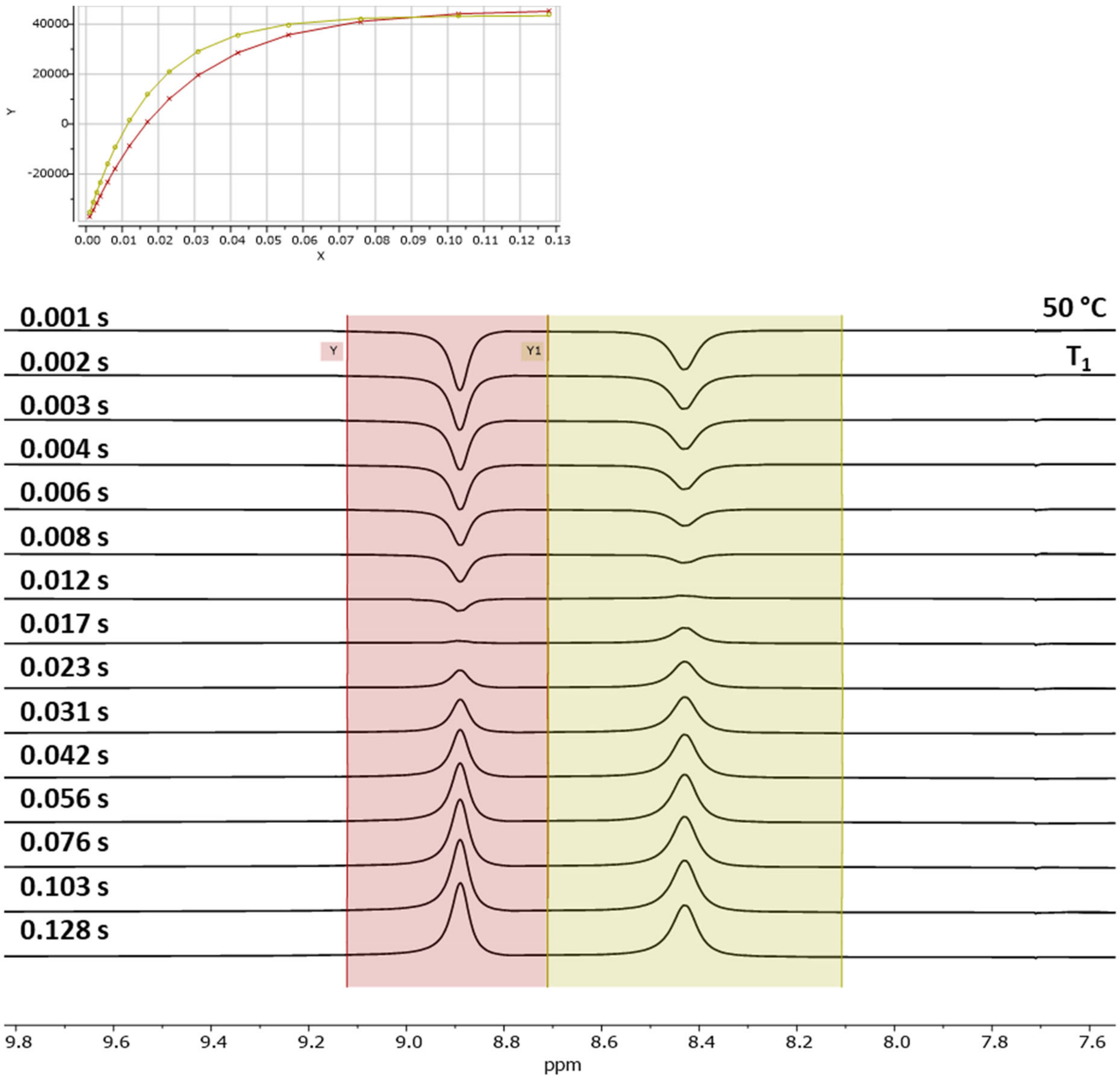

Supplementary Figure 182. T_1 (inverse recovery). Stacked spectra of the inverse recovery experiment to determine the T_1 values of the ortho (Y) and meta (Y1) protons of **Mn1** (0.05) **V2** (5 mM) in solution, in which the signal intensity (Y) is plotted against the time in seconds (X) (^{1}H, 500 MHz, chloroform-*d* : acetonitrile-*d*$_3$, v/v/v, 323 K).

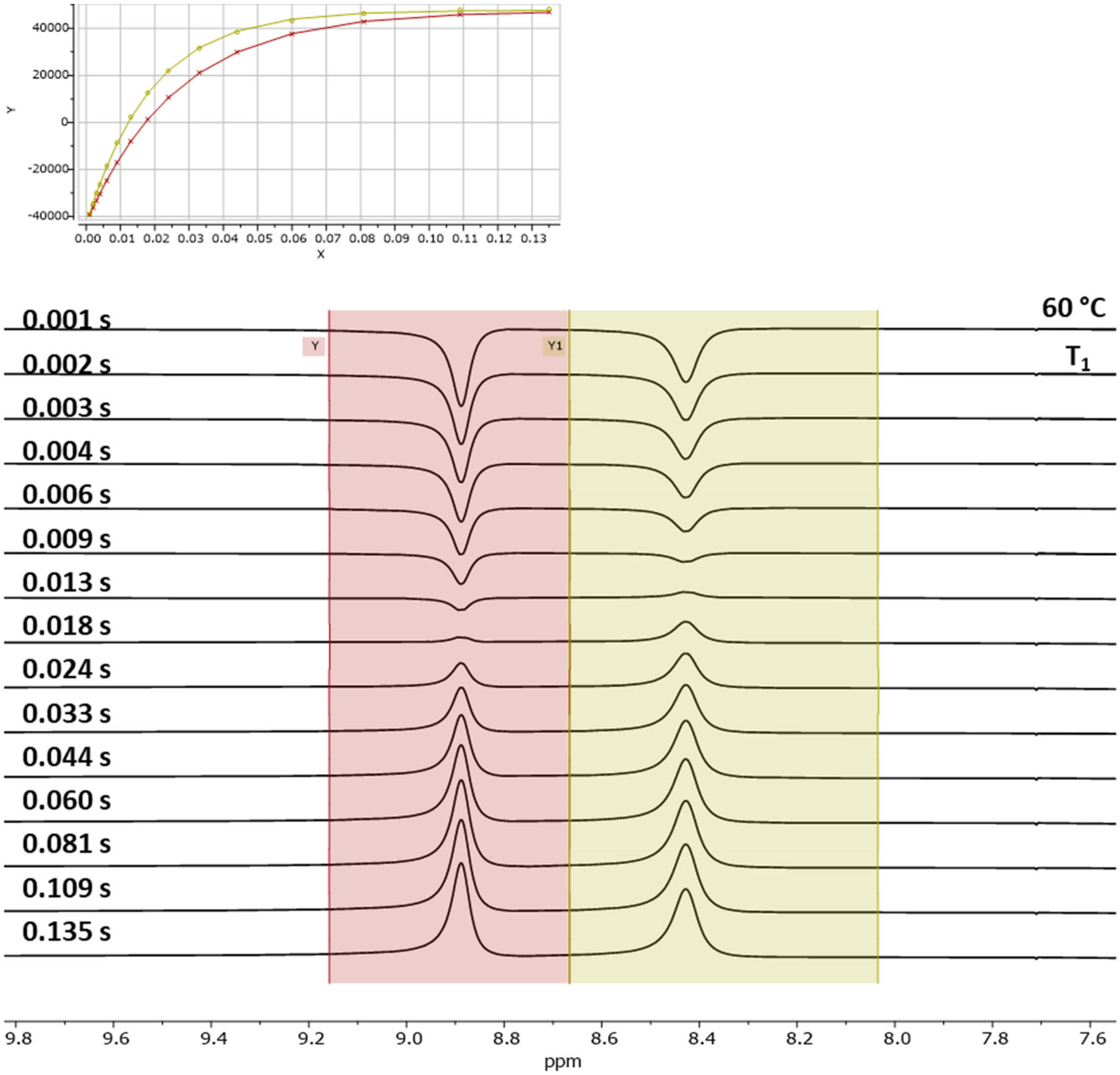

Supplementary Figure 183. T_1 (inverse recovery). Stacked spectra of the inverse recovery experiment to determine the T_1 values of the ortho (Y) and meta (Y1) protons of **Mn1** (0.05) **V2** (5 mM) in solution, in which the signal intensity (Y) is plotted against the time in seconds (X) (^{1}H, 500 MHz, chloroform-*d* : acetonitrile-*d$_3$*, v/v/v, 333 K).

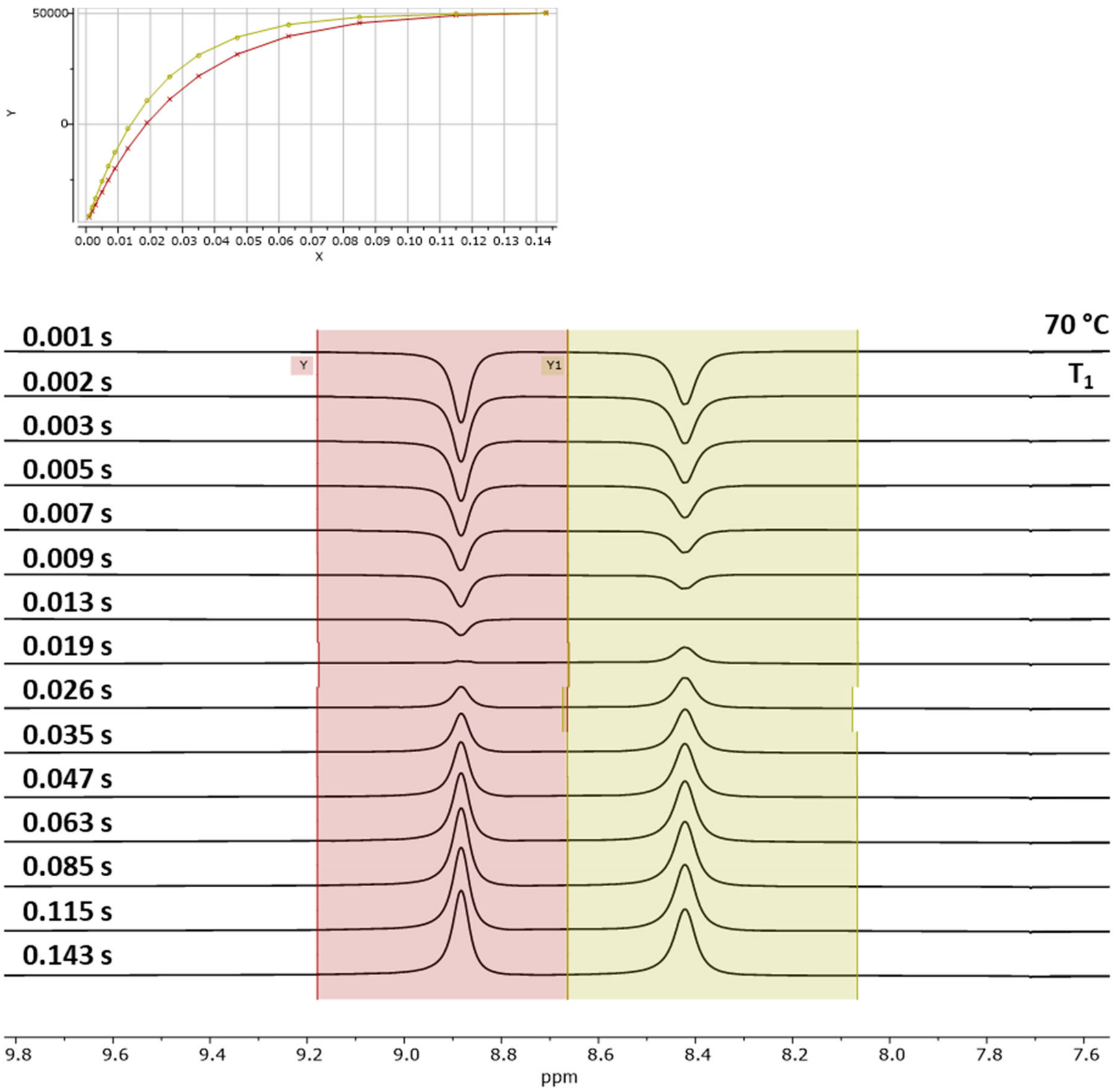

Supplementary Figure 184. T_1 (inverse recovery). Stacked spectra of the inverse recovery experiment to determine the T_1 values of the ortho (Y) and meta (Y1) protons of **Mn1** (0.05) **V2** (5 mM) in solution, in which the signal intensity (Y) is plotted against the time in seconds (X) (^{1}H, 500 MHz, chloroform-*d* : acetonitrile-*d*$_3$, v/v/v, 343 K).

5. Binding: T_1 (inverse recovery) spectra

5.1. Mn1/V1

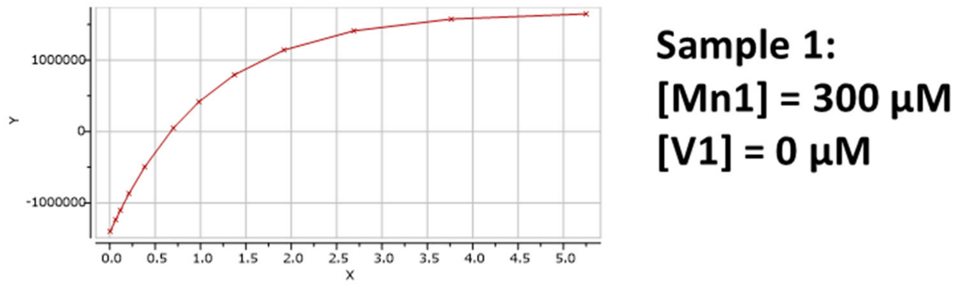

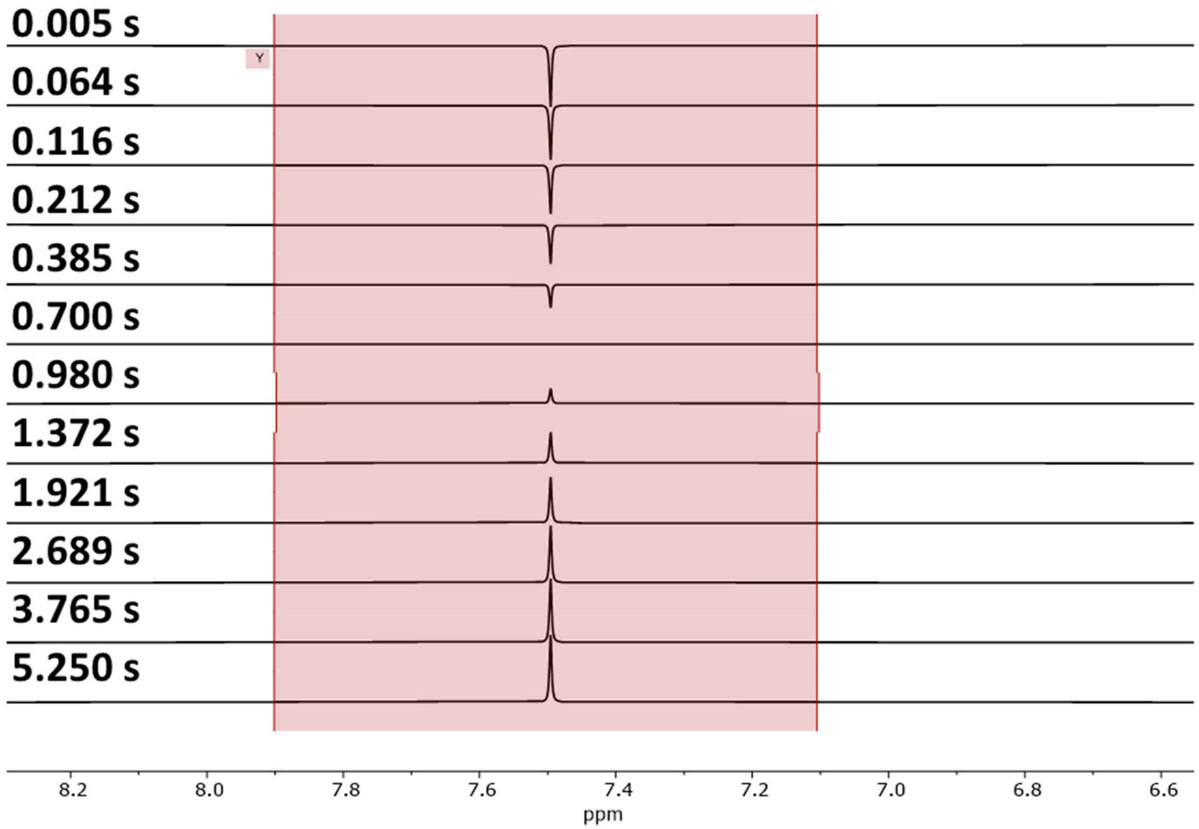

Supplementary Figure 185. T_1 (inverse recovery). Stacked spectra of the inverse recovery experiment to determine the T_1 values of the chloroform signal, in which the signal intensity (Y) is plotted against the time in seconds (X) ([**Mn1**] = 300 µM; [**V1**] = 0 µM; ^{1}H, 300 MHz, chloroform : chloroform-*d* : acetonitrile-*d*$_3$, 1 : 4 : 5, v/v/v, 299 K).

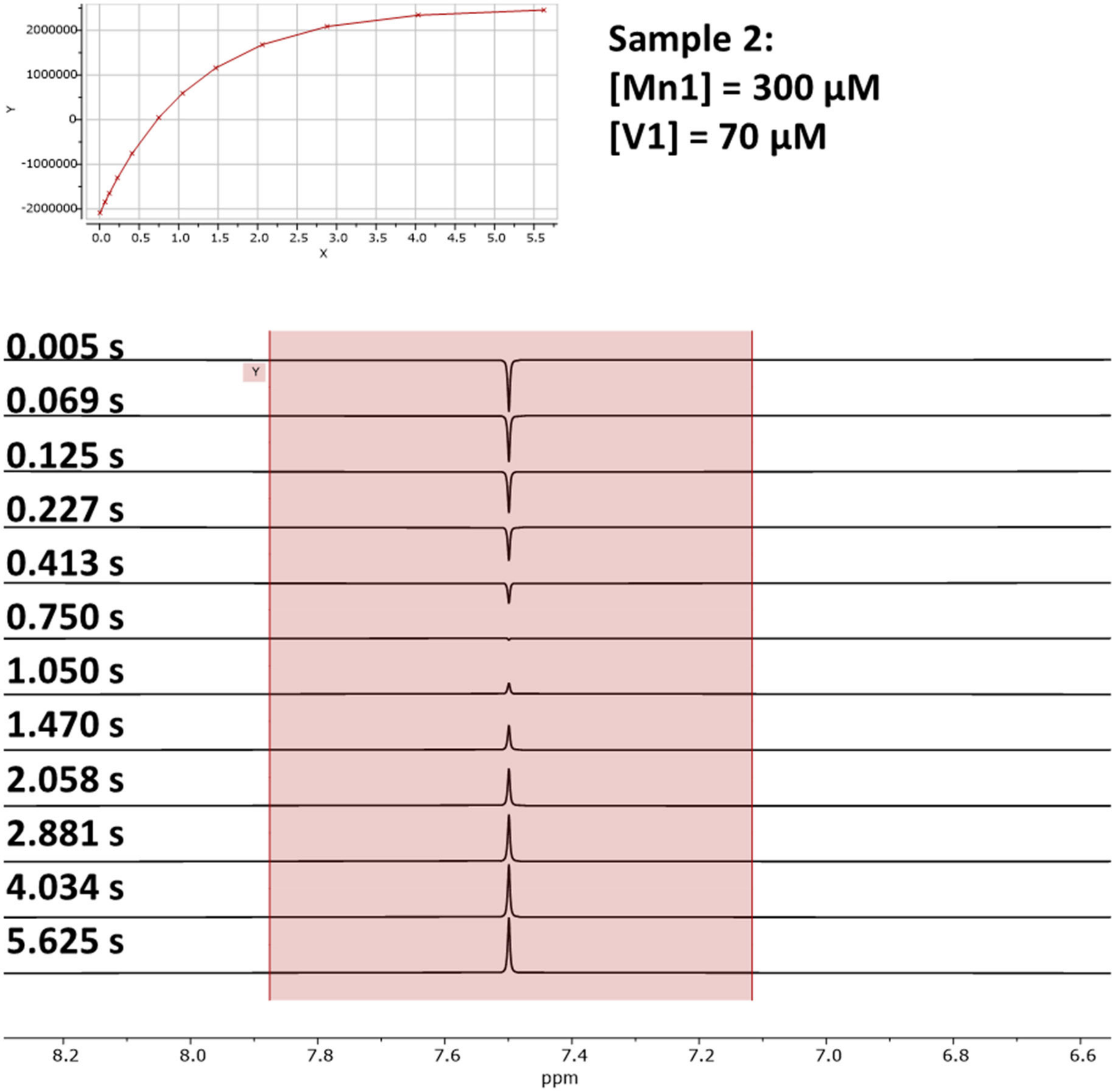

Supplementary Figure 186. T_1 (inverse recovery). Stacked spectra of the inverse recovery experiment to determine the T_1 values of the chloroform signal, in which the signal intensity (Y) is plotted against the time in seconds (X) ([**Mn1**] = 300 µM; [**V1**] = 70 µM; ^{1}H, 300 MHz, chloroform : chloroform-*d* : acetonitrile-d_3, 1 : 4 : 5, v/v/v, 299 K).

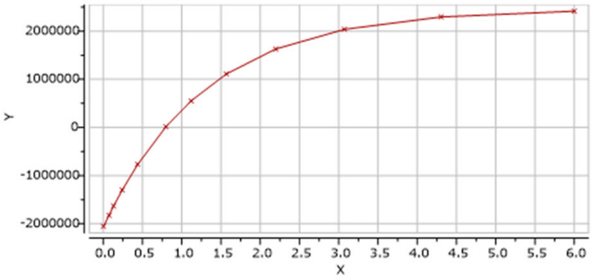

Sample 3:
[Mn1] = 300 µM
[V1] = 140 µM

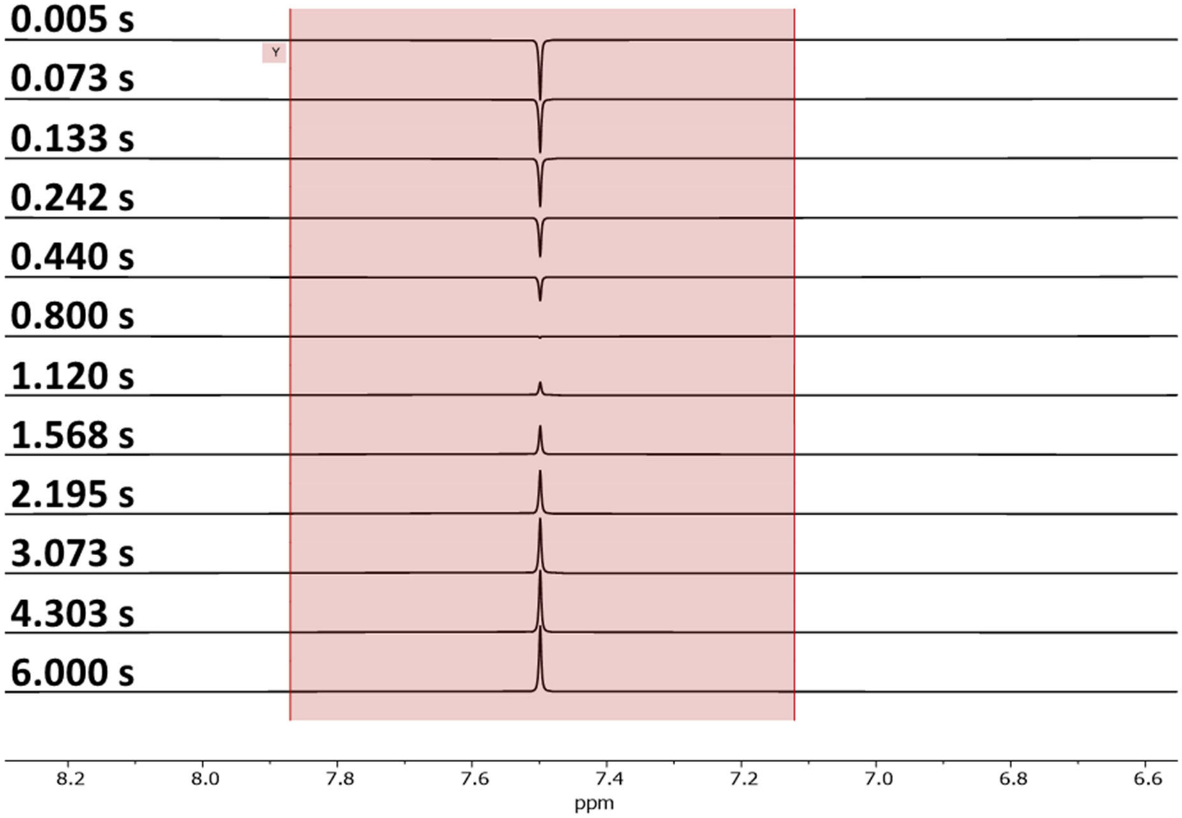

Supplementary Figure 187. T_1 (inverse recovery). Stacked spectra of the inverse recovery experiment to determine the T_1 values of the chloroform signal, in which the signal intensity (Y) is plotted against the time in seconds (X) ([**Mn1**] = 300 µM; [**V1**] = 140 µM; ^{1}H, 300 MHz, chloroform : chloroform-*d* : acetonitrile-d_3, 1 : 4 : 5, v/v/v, 299 K).

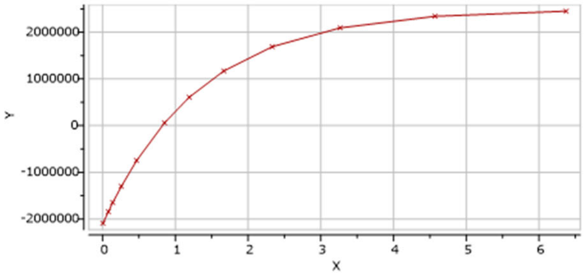

Sample 4:
[Mn1] = 300 µM
[V1] = 252 µM

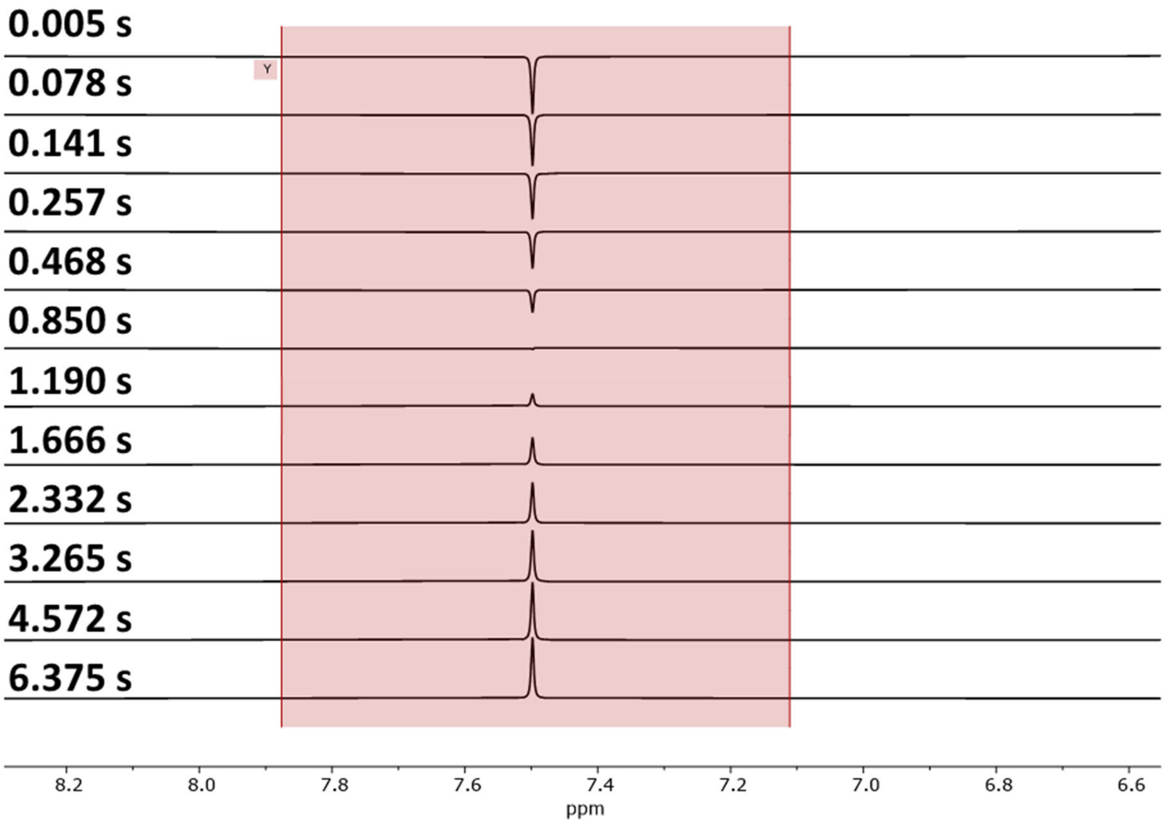

Supplementary Figure 188. T_1 (inverse recovery). Stacked spectra of the inverse recovery experiment to determine the T_1 values of the chloroform signal, in which the signal intensity (Y) is plotted against the time in seconds (X) ([**Mn1**] = 300 µM; [**V1**] = 252 µM; ^{1}H, 300 MHz, chloroform : chloroform-*d* : acetonitrile-d_3, 1 : 4 : 5, v/v/v, 299 K).

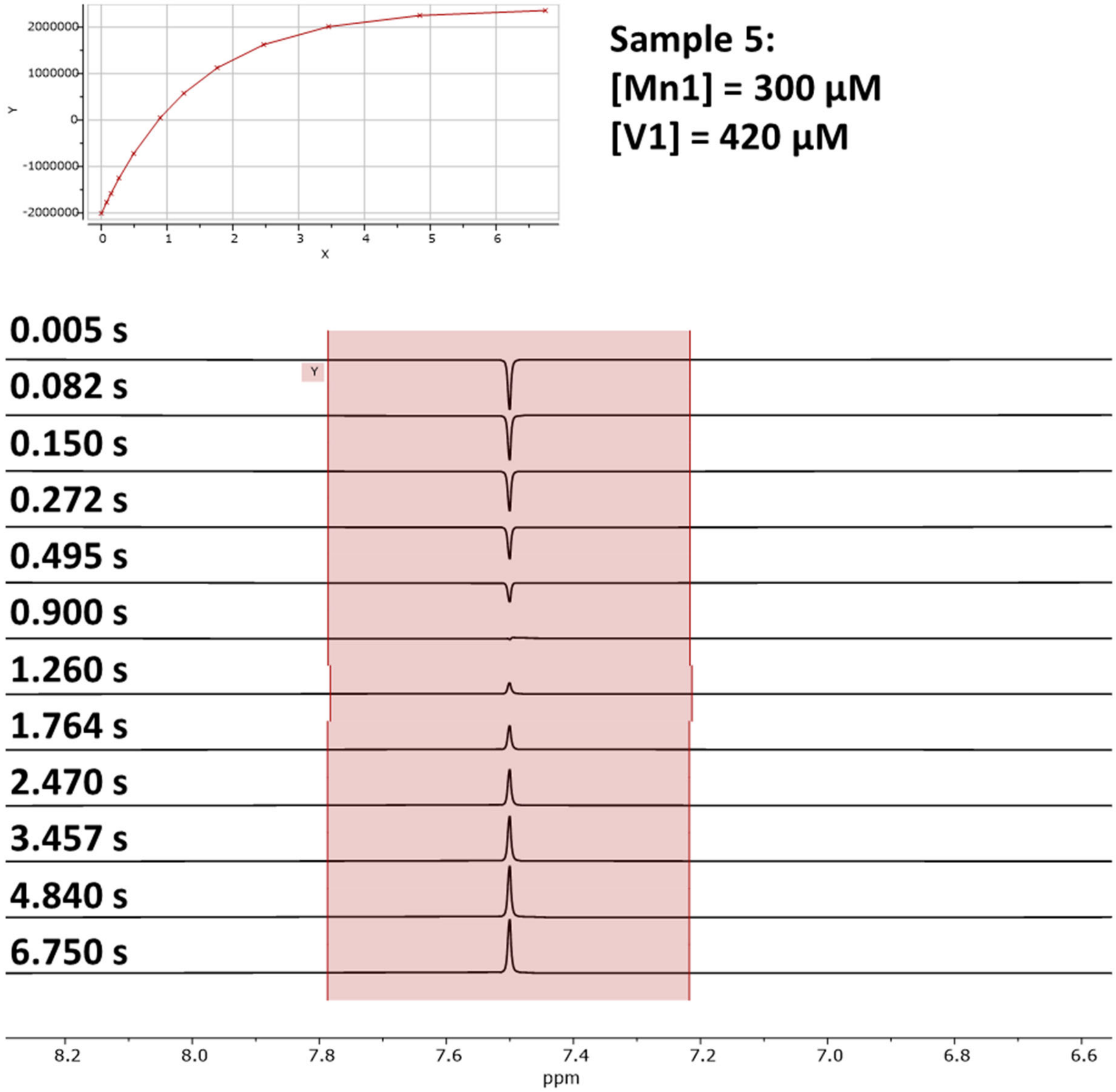

Supplementary Figure 189. T_1 (inverse recovery). Stacked spectra of the inverse recovery experiment to determine the T_1 values of the chloroform signal, in which the signal intensity (Y) is plotted against the time in seconds (X) ([**Mn1**] = 300 µM; [**V1**] = 420 µM; ^{1}H, 300 MHz, chloroform : chloroform-*d* : acetonitrile-*d*$_3$, 1 : 4 : 5, v/v/v, 299 K).

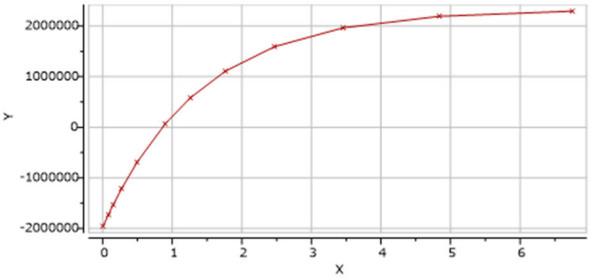

Sample 6:
[Mn1] = 300 µM
[V1] = 630 µM

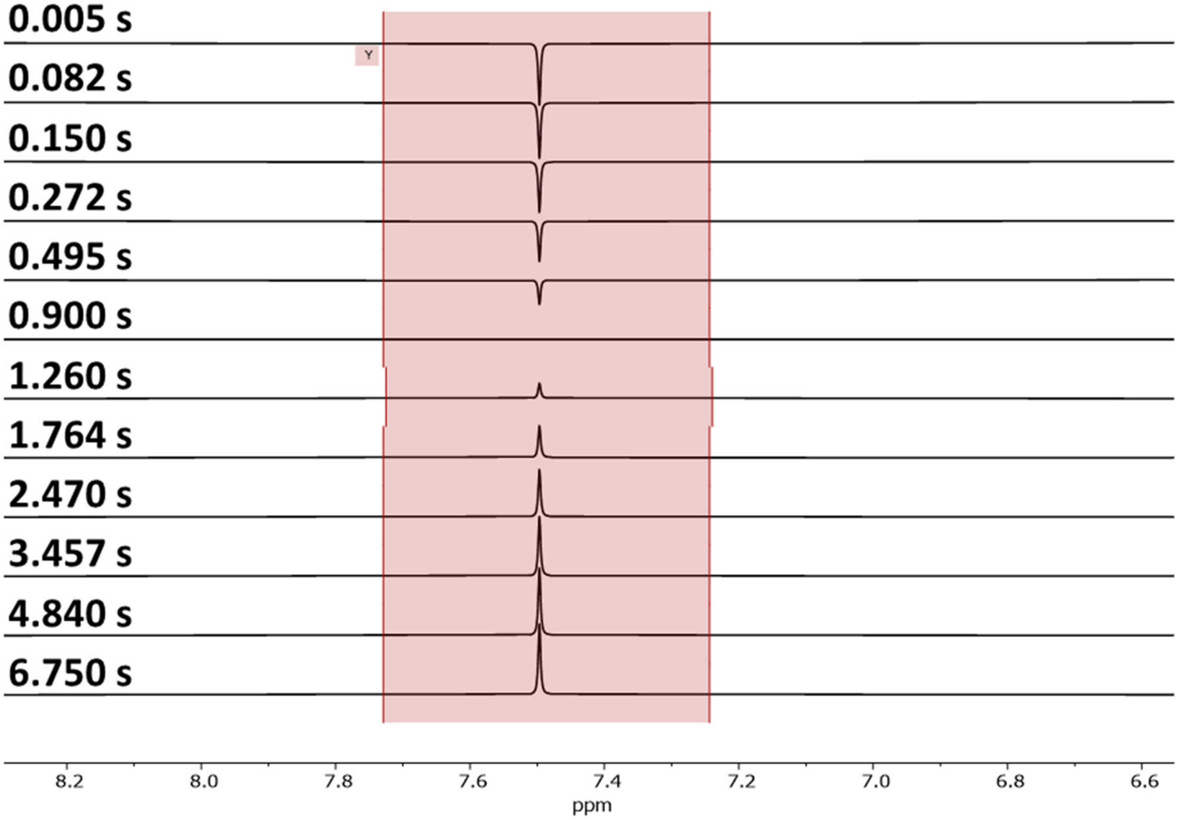

Supplementary Figure 190. T_1 (inverse recovery). Stacked spectra of the inverse recovery experiment to determine the T_1 values of the chloroform signal, in which the signal intensity (Y) is plotted against the time in seconds (X) ([**Mn1**] = 300 µM; [**V1**] = 630 µM; ^{1}H, 300 MHz, chloroform : chloroform-*d* : acetonitrile-*d$_3$*, 1 : 4 : 5, v/v/v, 299 K).

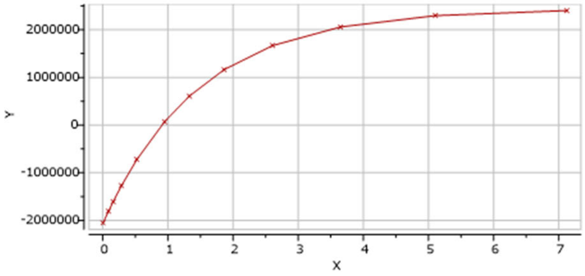

Sample 7:
[Mn1] = 300 µM
[V1] = 900 µM

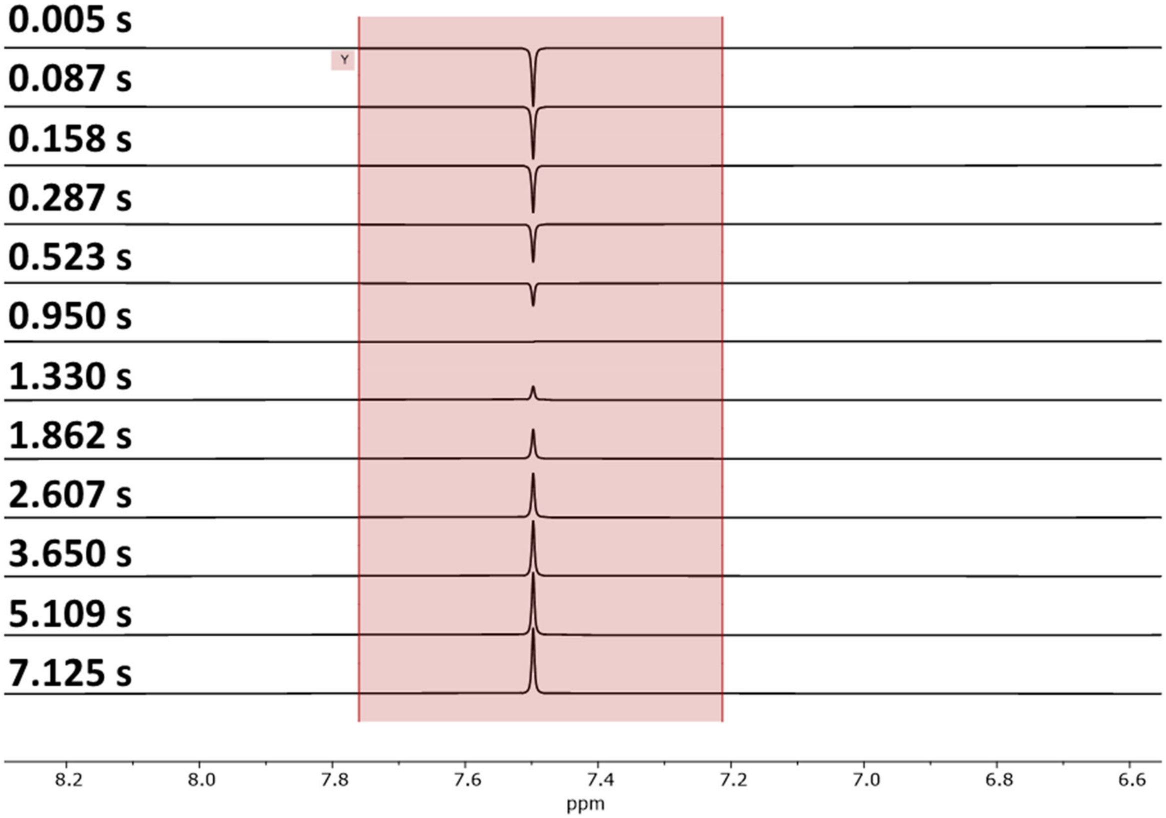

Supplementary Figure 191. T_1 (inverse recovery). Stacked spectra of the inverse recovery experiment to determine the T_1 values of the chloroform signal, in which the signal intensity (Y) is plotted against the time in seconds (X) ([**Mn1**] = 300 µM; [**V1**] = 900 µM; ^{1}H, 300 MHz, chloroform : chloroform-*d* : acetonitrile-d_3, 1 : 4 : 5, v/v/v, 299 K).

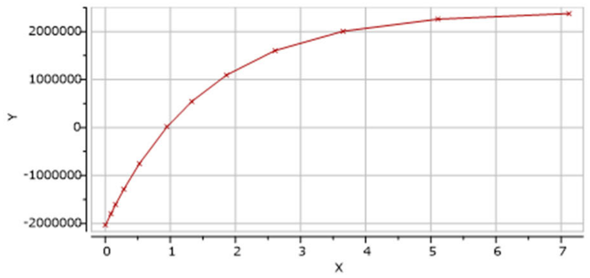

Sample 8:
[Mn1] = 300 µM
[V1] = 1350 µM

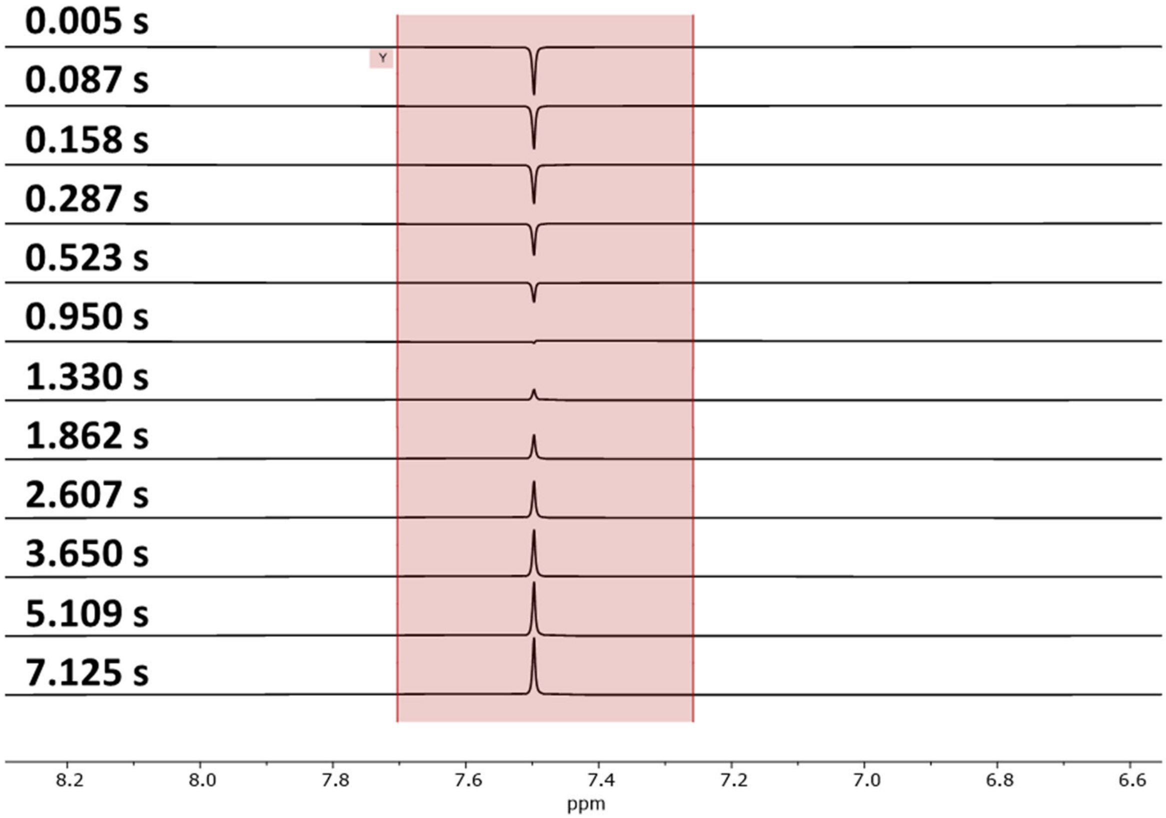

Supplementary Figure 192. T_1 (inverse recovery). Stacked spectra of the inverse recovery experiment to determine the T_1 values of the chloroform signal, in which the signal intensity (Y) is plotted against the time in seconds (X) ([**Mn1**] = 300 µM; [**V1**] = 1350 µM; ^{1}H, 300 MHz, chloroform : chloroform-*d* : acetonitrile-d_3, 1 : 4 : 5, v/v/v, 299 K).

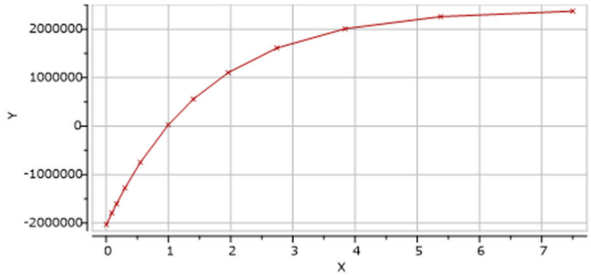

Sample 9:
[Mn1] = 300 µM
[V1] = 2100 µM

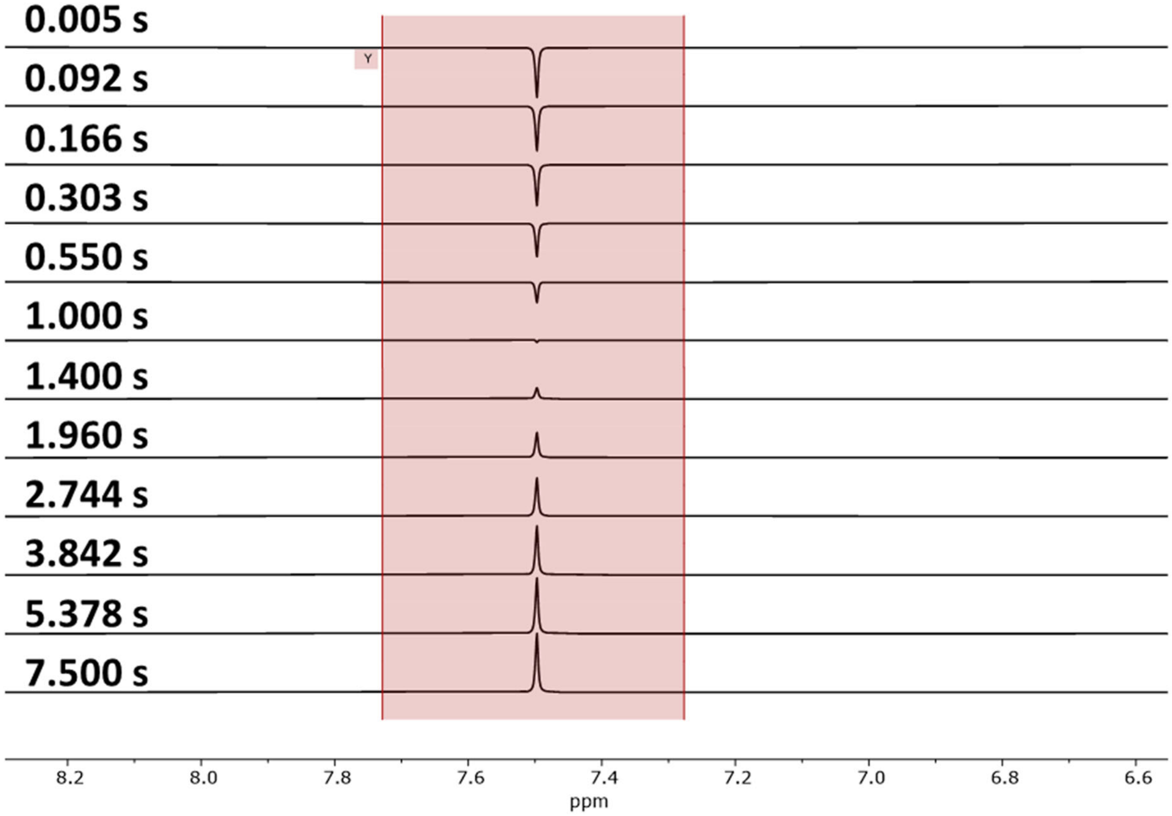

Supplementary Figure 193. T_1 (inverse recovery). Stacked spectra of the inverse recovery experiment to determine the T_1 values of the chloroform signal, in which the signal intensity (Y) is plotted against the time in seconds (X) ([**Mn1**] = 300 µM; [**V1**] = 2100 µM; ^{1}H, 300 MHz, chloroform : chloroform-*d* : acetonitrile-d_3, 1 : 4 : 5, v/v/v, 299 K).

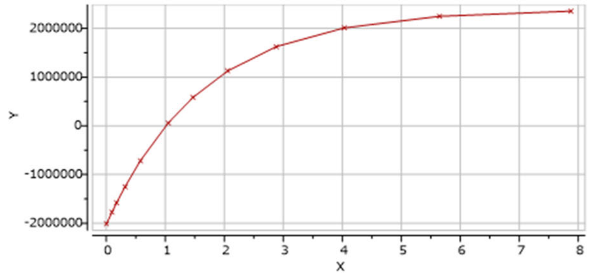

Sample 10:
[Mn1] = 300 µM
[V1] = 3000 µM

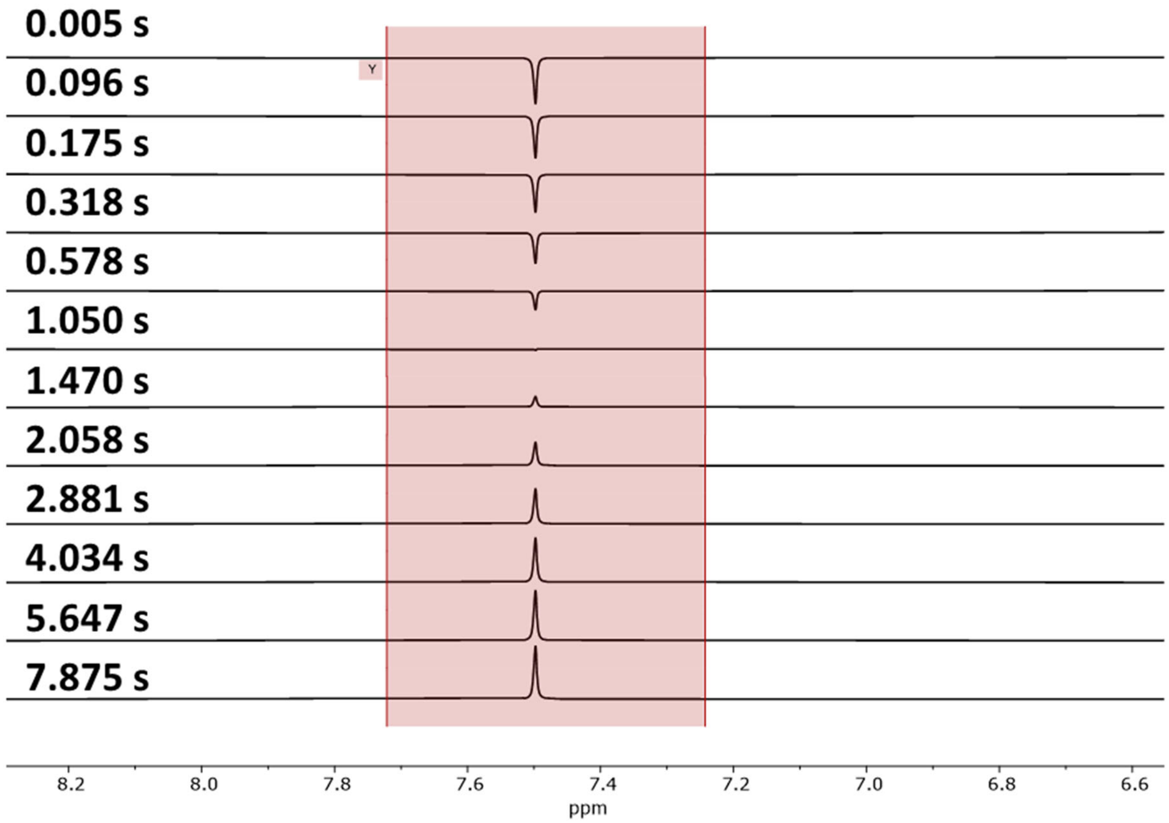

Supplementary Figure 194. T_1 (inverse recovery). Stacked spectra of the inverse recovery experiment to determine the T_1 values of the chloroform signal, in which the signal intensity (Y) is plotted against the time in seconds (X) ([**Mn1**] = 300 µM; [**V1**] = 3000 µM; ^{1}H, 300 MHz, chloroform : chloroform-*d* : acetonitrile-d_3, 1 : 4 : 5, v/v/v, 299 K).

5.2. Mn1/VP

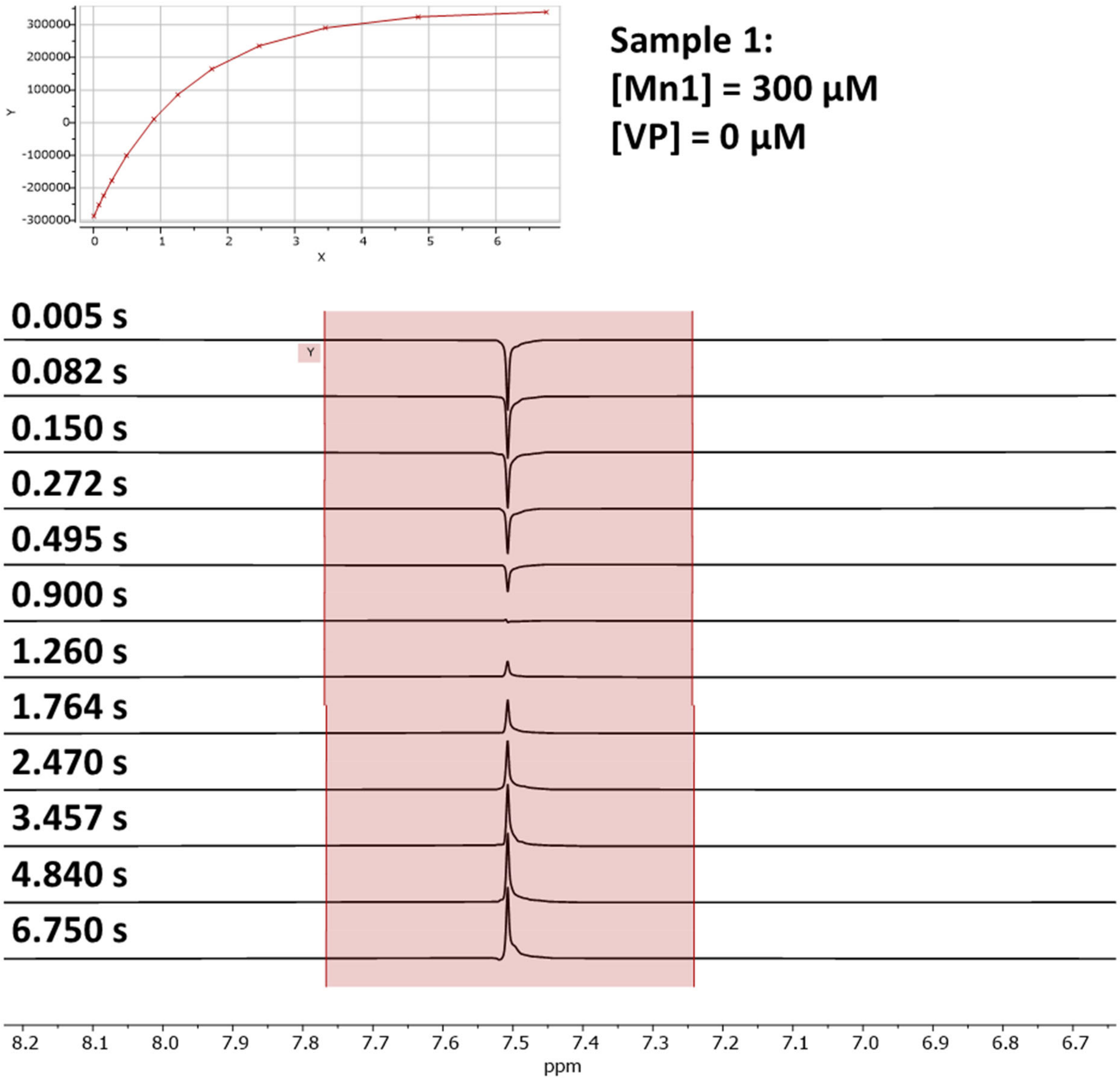

Supplementary Figure 195. T_1 (inverse recovery). Stacked spectra of the inverse recovery experiment to determine the T_1 values of the chloroform signal, in which the signal intensity (Y) is plotted against the time in seconds (X) ([**Mn1**] = 300 µM; [**VP**] = 0 µM; ^{1}H, 300 MHz, chloroform : chloroform-*d* : acetonitrile-*d$_3$*, 1 : 4 : 5, v/v/v, 299 K).

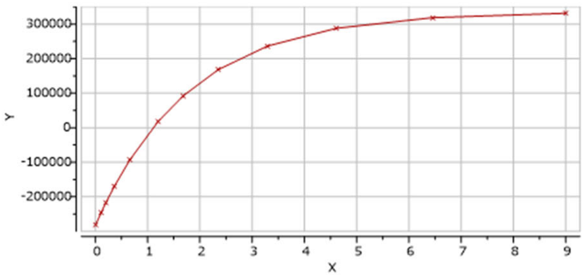

Sample 2:
[Mn1] = 300 µM
[VP] = 680 µM

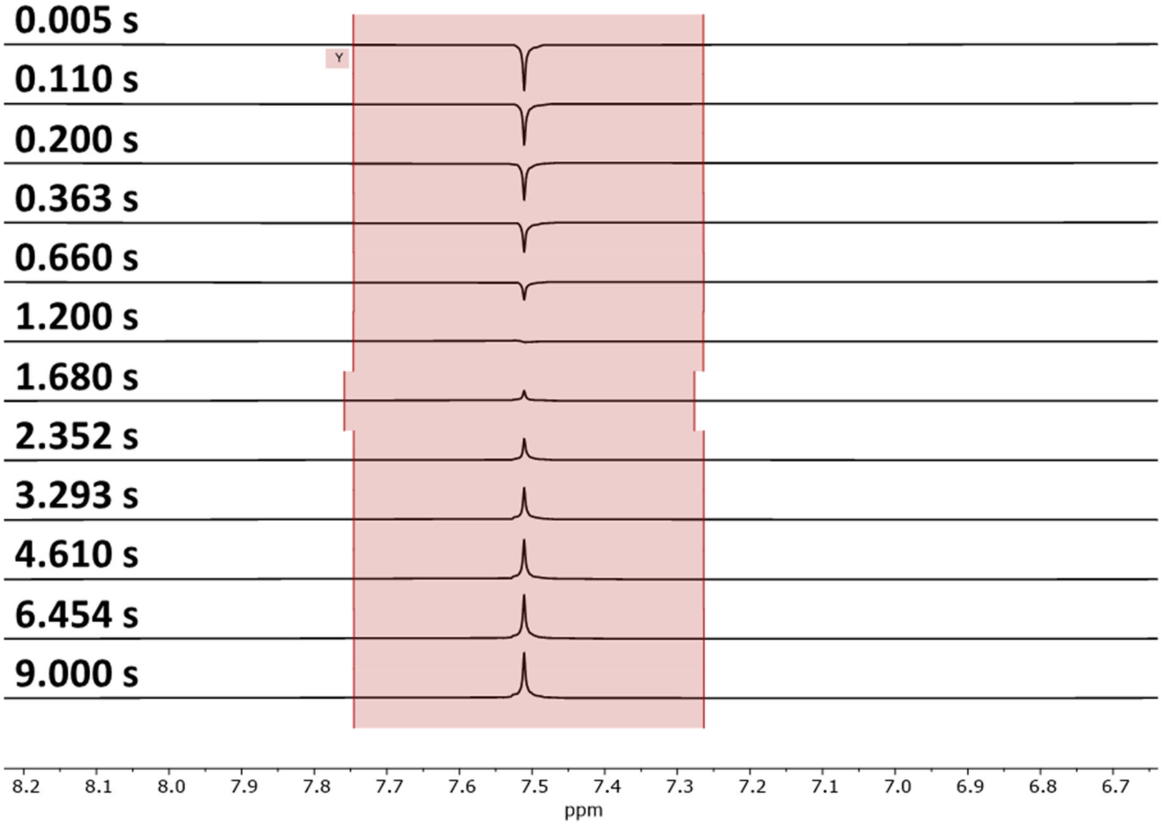

Supplementary Figure 196. T_1 (inverse recovery). Stacked spectra of the inverse recovery experiment to determine the T_1 values of the chloroform signal, in which the signal intensity (Y) is plotted against the time in seconds (X) ([**Mn1**] = 300 µM; [**VP**] = 680 µM; ^{1}H, 300 MHz, chloroform : chloroform-*d* : acetonitrile-*d*$_3$, 1 : 4 : 5, v/v/v, 299 K).

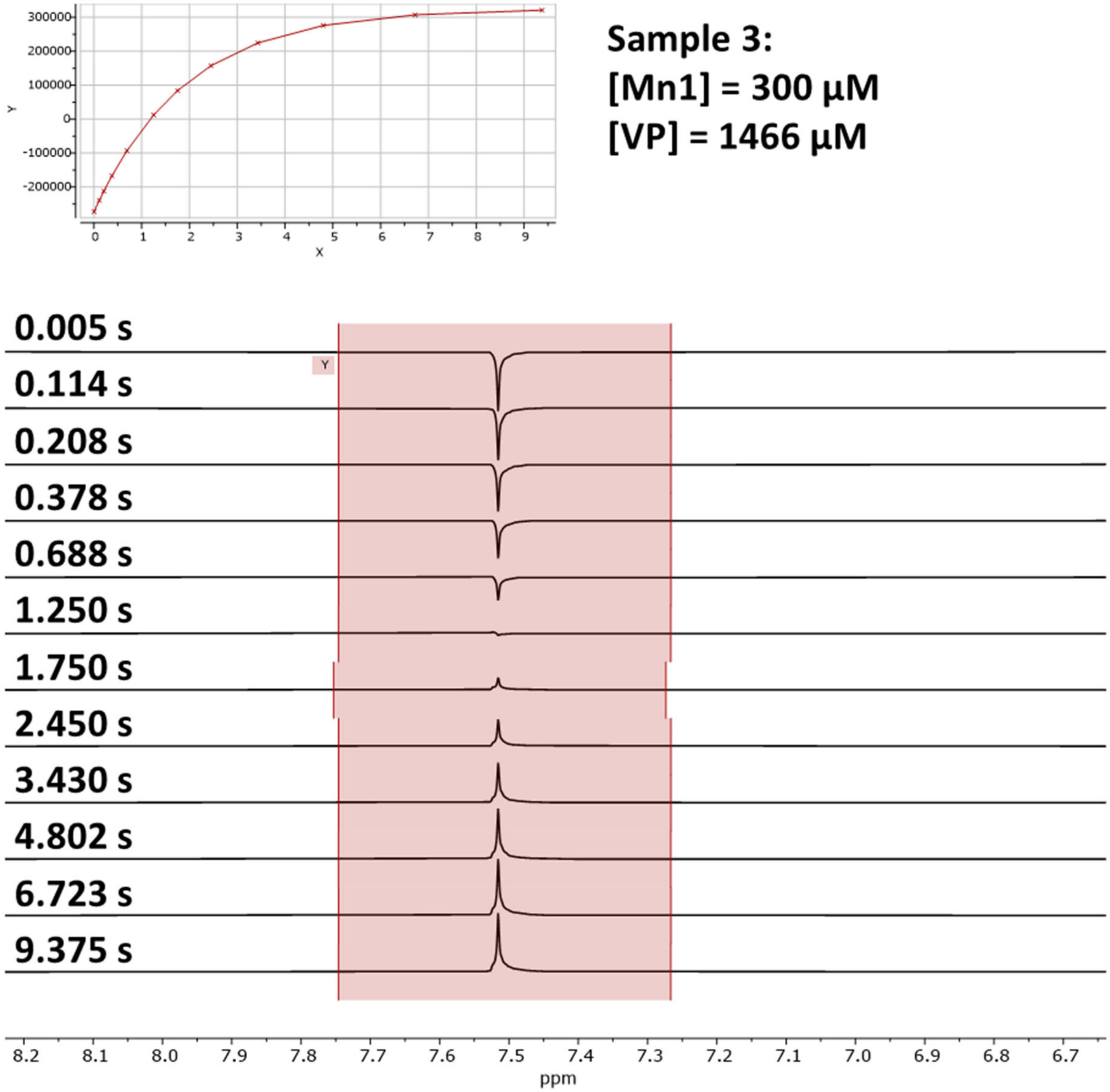

Supplementary Figure 197. T_1 (inverse recovery). Stacked spectra of the inverse recovery experiment to determine the T_1 values of the chloroform signal, in which the signal intensity (Y) is plotted against the time in seconds (X) ([**Mn1**] = 300 µM; [**VP**] = 1466 µM; ^{1}H, 300 MHz, chloroform : chloroform-*d* : acetonitrile-d_3, 1 : 4 : 5, v/v/v, 299 K).

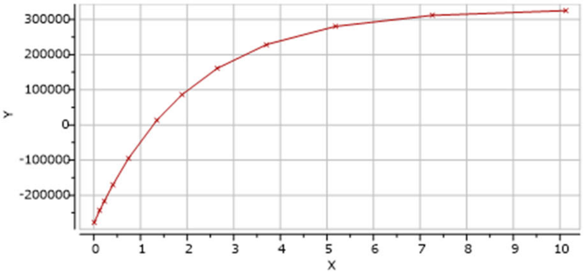

Sample 4:
[Mn1] = 300 µM
[VP] = 2706 µM

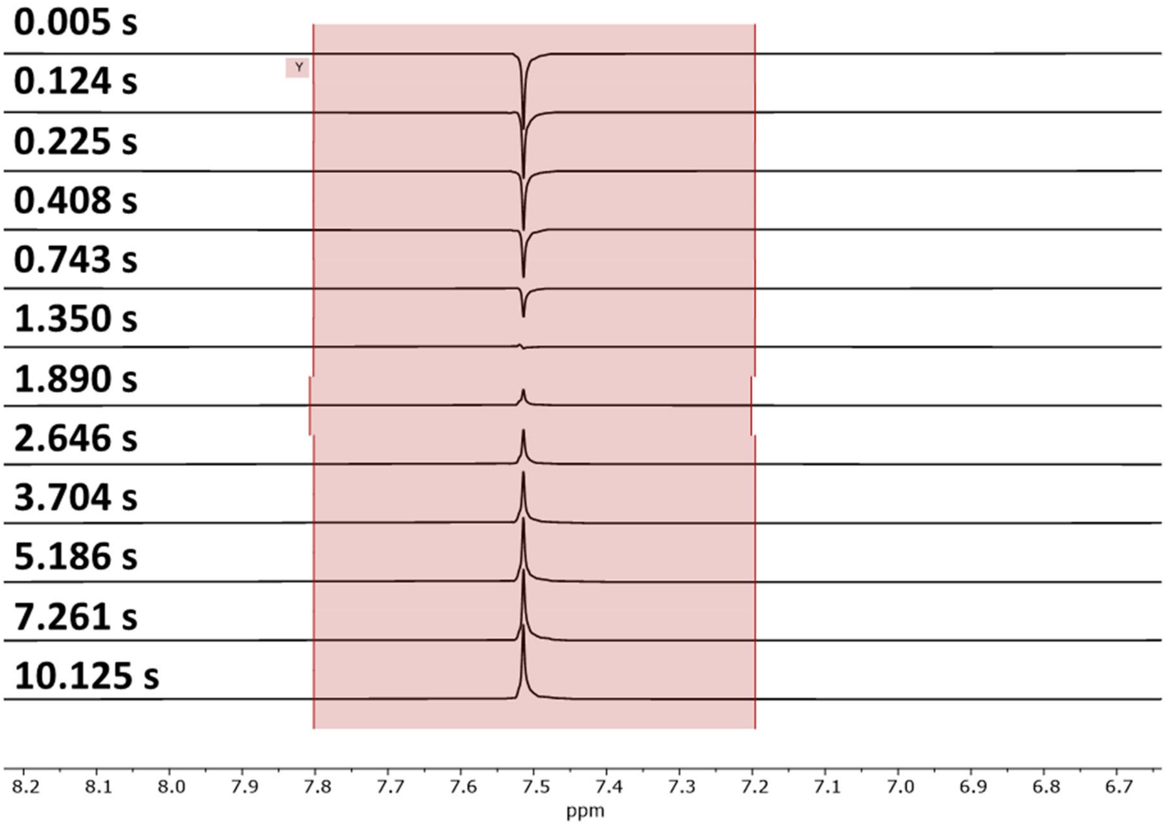

Supplementary Figure 198. T_1 (inverse recovery). Stacked spectra of the inverse recovery experiment to determine the T_1 values of the chloroform signal, in which the signal intensity (Y) is plotted against the time in seconds (X) ([**Mn1**] = 300 µM; [**VP**] = 2706 µM; ^{1}H, 300 MHz, chloroform : chloroform-*d* : acetonitrile-d_3, 1 : 4 : 5, v/v/v, 299 K).

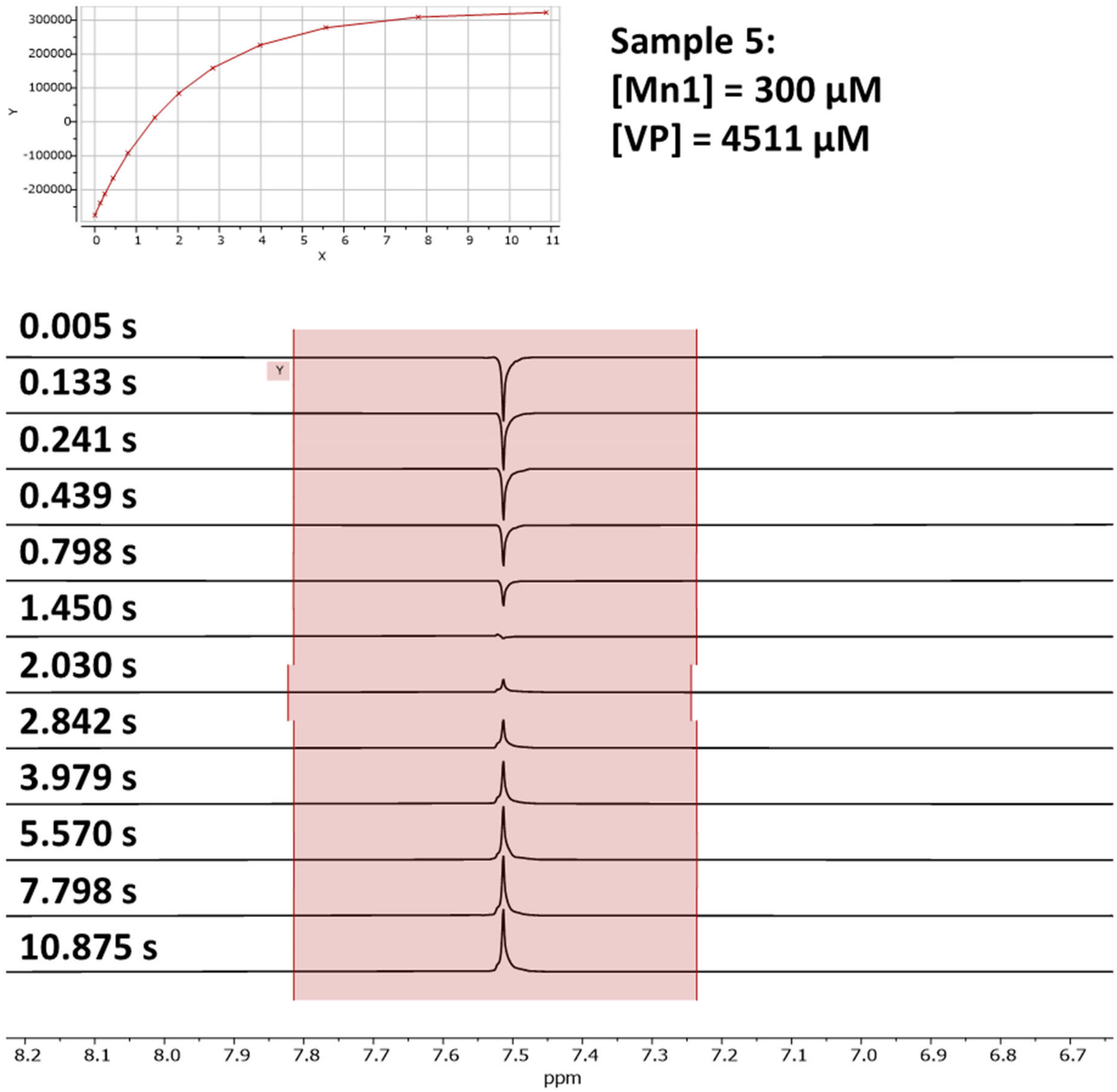

Supplementary Figure 199. T_1 (inverse recovery). Stacked spectra of the inverse recovery experiment to determine the T_1 values of the chloroform signal, in which the signal intensity (Y) is plotted against the time in seconds (X) ([**Mn1**] = 300 µM; [**VP**] = 4511 µM; ^{1}H, 300 MHz, chloroform : chloroform-*d* : acetonitrile-d_3, 1 : 4 : 5, v/v/v, 299 K).

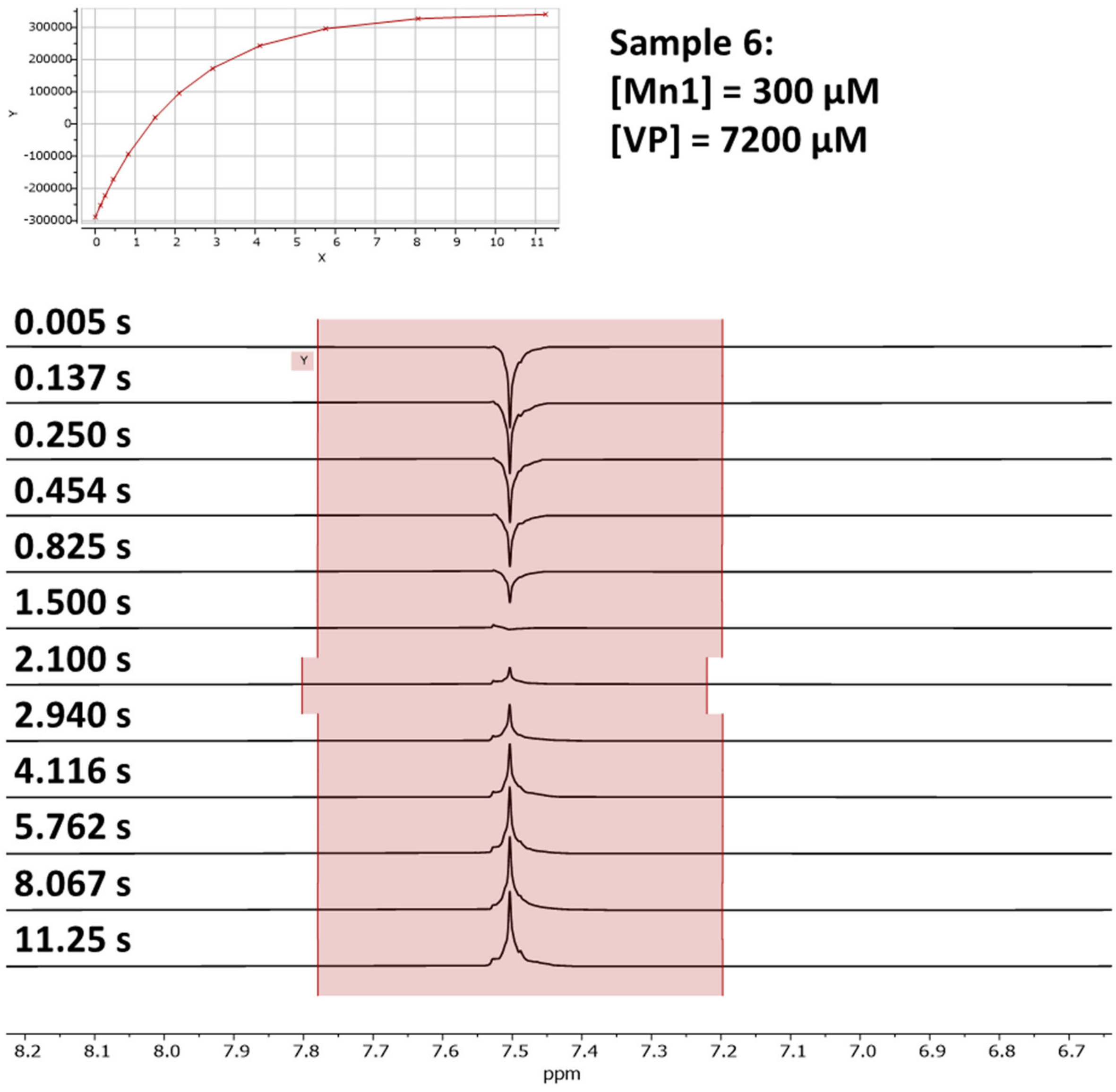

Supplementary Figure 200. T_1 (inverse recovery). Stacked spectra of the inverse recovery experiment to determine the T_1 values of the chloroform signal, in which the signal intensity (Y) is plotted against the time in seconds (X) ([**Mn1**] = 300 µM; [**VP**] = 7200 µM; ^{1}H, 300 MHz, chloroform : chloroform-*d* : acetonitrile-d_3, 1 : 4 : 5, v/v/v, 299 K).

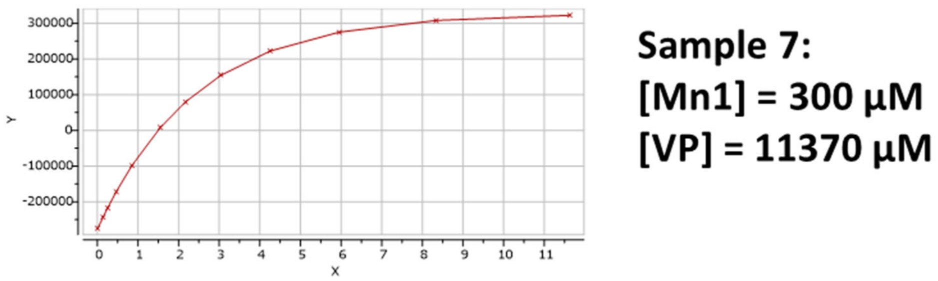

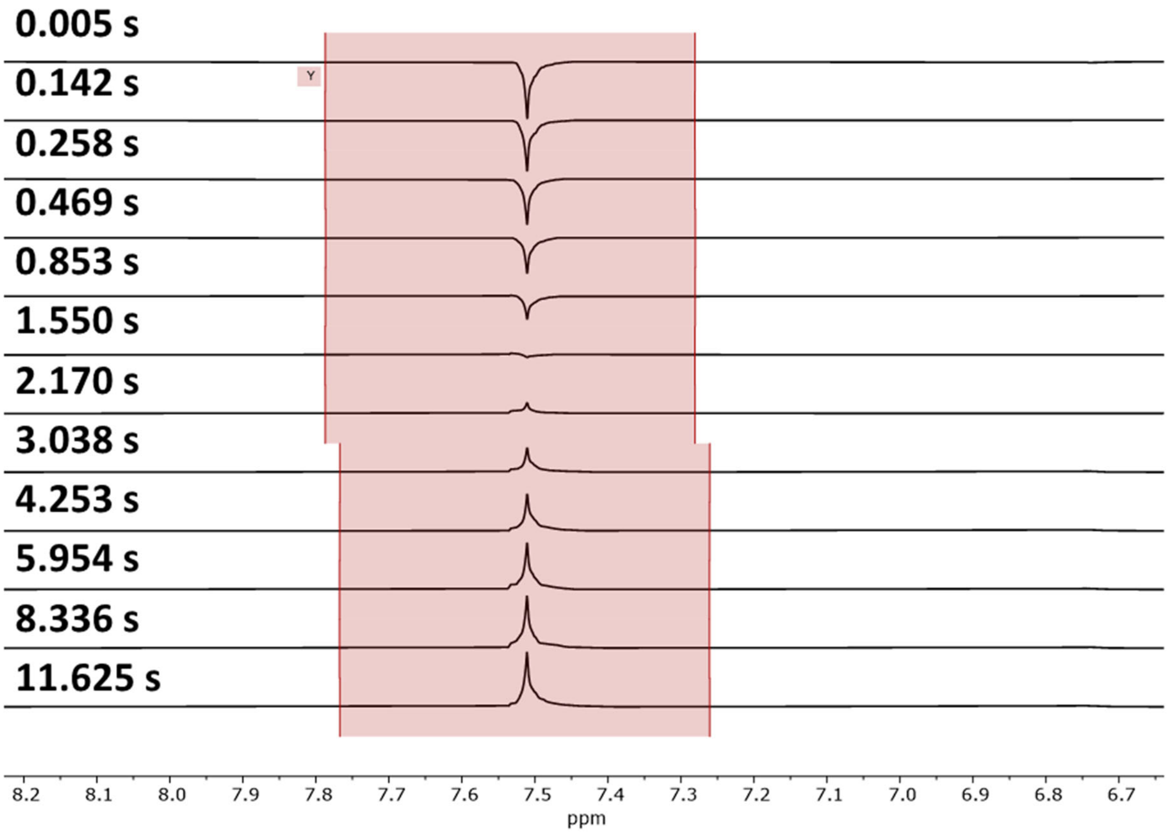

Supplementary Figure 201. T_1 (inverse recovery). Stacked spectra of the inverse recovery experiment to determine the T_1 values of the chloroform signal, in which the signal intensity (Y) is plotted against the time in seconds (X) ([**Mn1**] = 300 µM; [**VP**] = 11370 µM; ^{1}H, 300 MHz, chloroform : chloroform-*d* : acetonitrile-d_3, 1 : 4 : 5, v/v/v, 299 K).

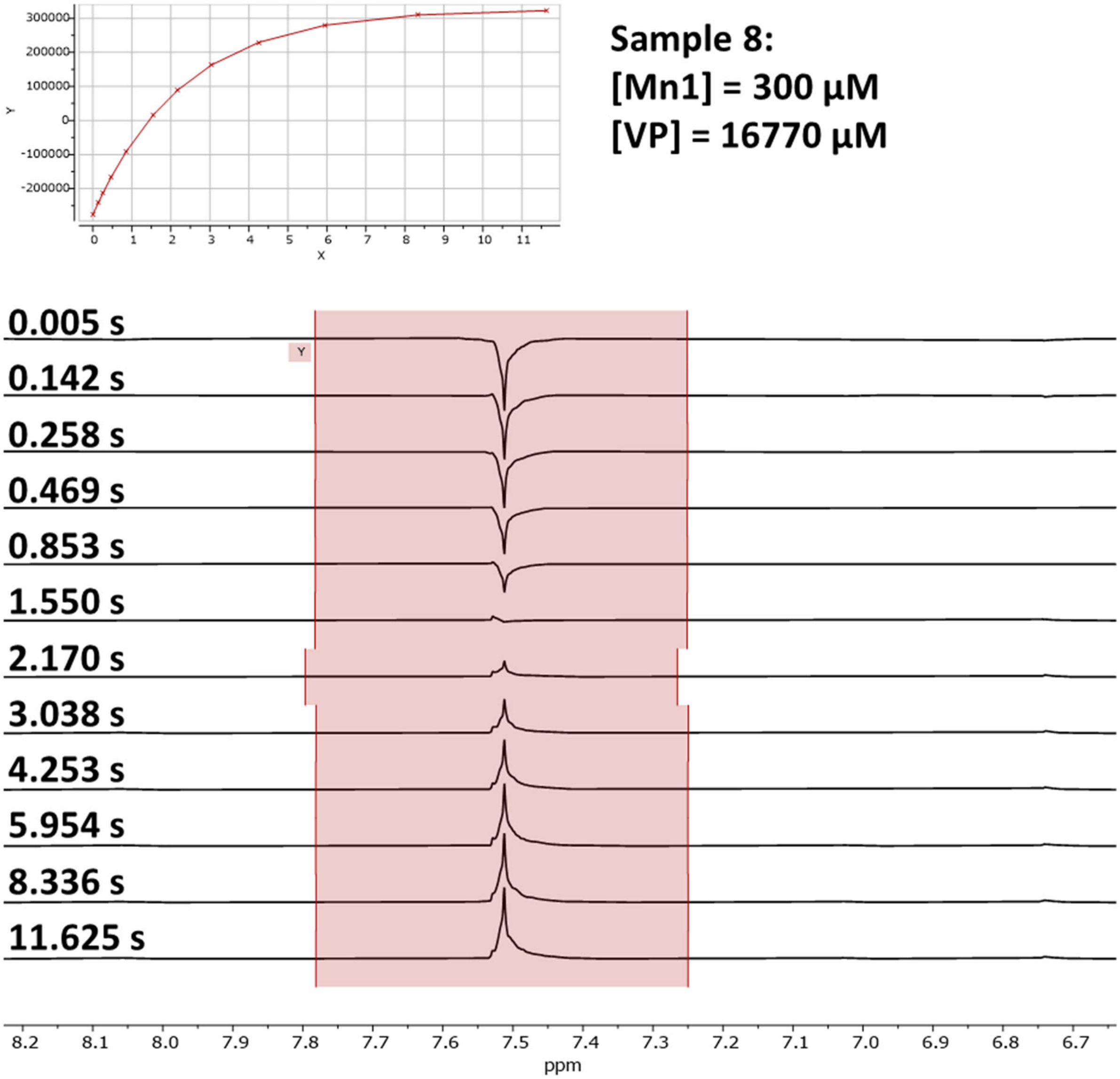

Supplementary Figure 202. T_1 (inverse recovery). Stacked spectra of the inverse recovery experiment to determine the T_1 values of the chloroform signal, in which the signal intensity (Y) is plotted against the time in seconds (X) ([**Mn1**] = 300 µM; [**VP**] = 16770 µM; ^{1}H, 300 MHz, chloroform : chloroform-*d* : acetonitrile-*d*$_3$, 1 : 4 : 5, v/v/v, 299 K).

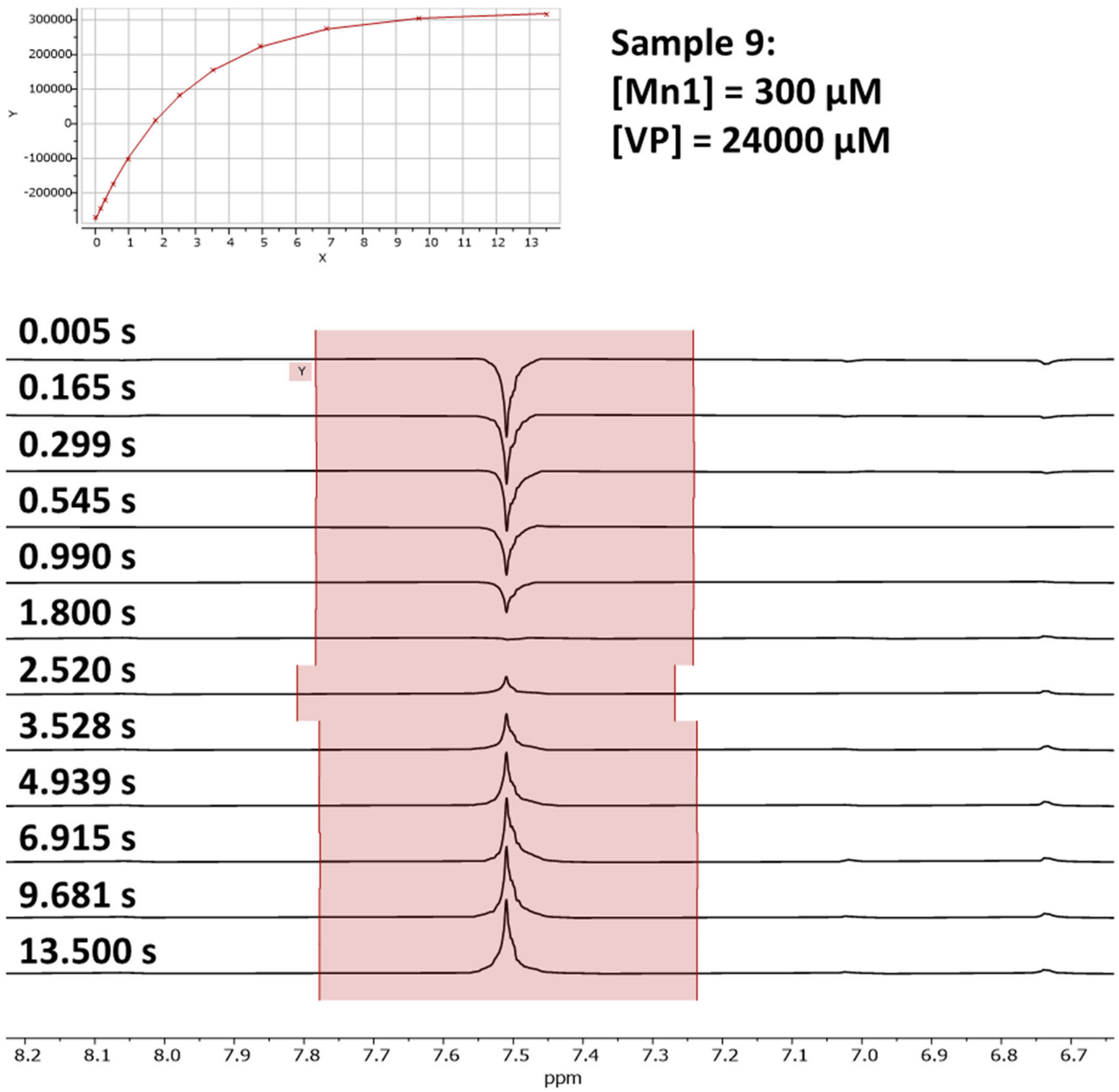

Supplementary Figure 203. T_1 (inverse recovery). Stacked spectra of the inverse recovery experiment to determine the T_1 values of the chloroform signal, in which the signal intensity (Y) is plotted against the time in seconds (X) ([**Mn1**] = 300 µM; [**VP**] = 24000 µM; ^{1}H, 300 MHz, chloroform : chloroform-*d* : acetonitrile-*d*$_3$, 1 : 4 : 5, v/v/v, 299 K).

5.3. Mn1/V2

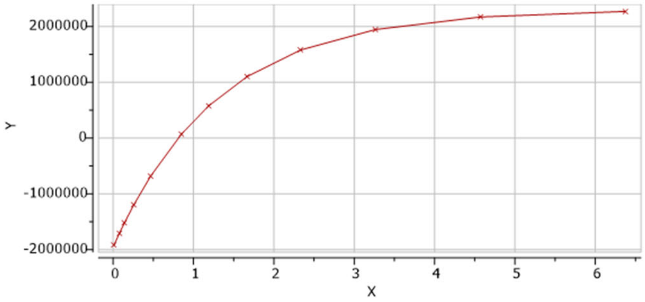

Sample 1:
[Mn1] = 300 µM
[V2] = 0 µM

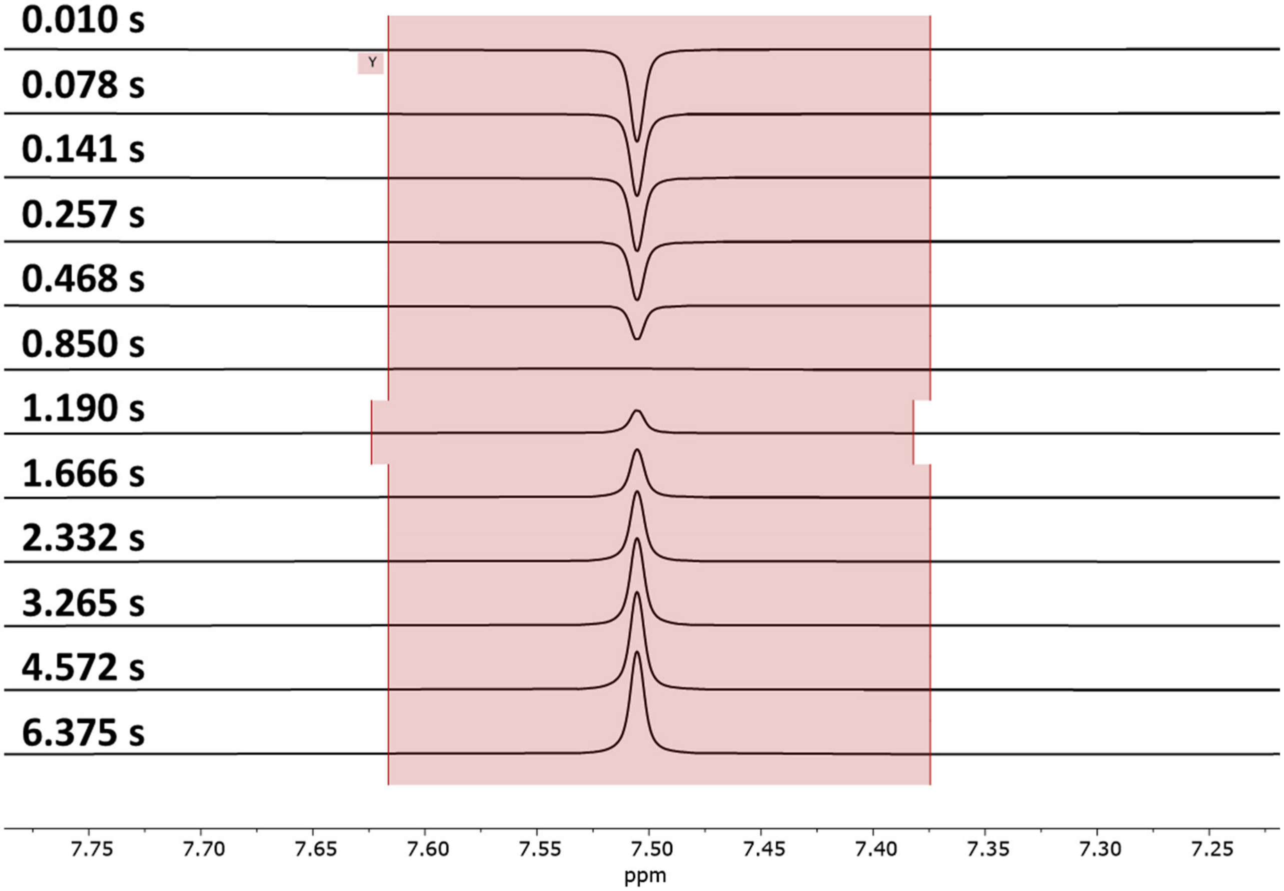

Supplementary Figure 204. T_1 (inverse recovery). Stacked spectra of the inverse recovery experiment to determine the T_1 values of the chloroform signal, in which the signal intensity (Y) is plotted against the time in seconds (X) ([**Mn1**] = 300 µM; [**V2**] = 0 µM; ^{1}H, 300 MHz, chloroform : chloroform-*d* : acetonitrile-d_3, 1 : 4 : 5, v/v/v, 299 K).

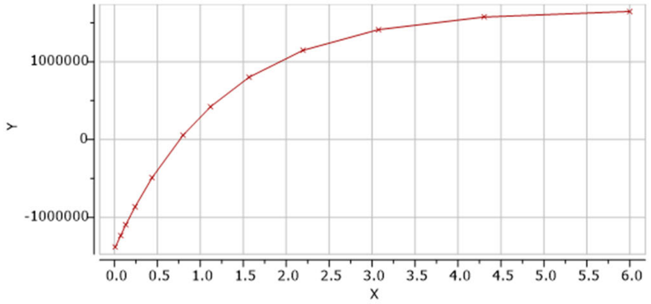

Sample 2:
[Mn1] = 300 µM
[V2] = 150 µM

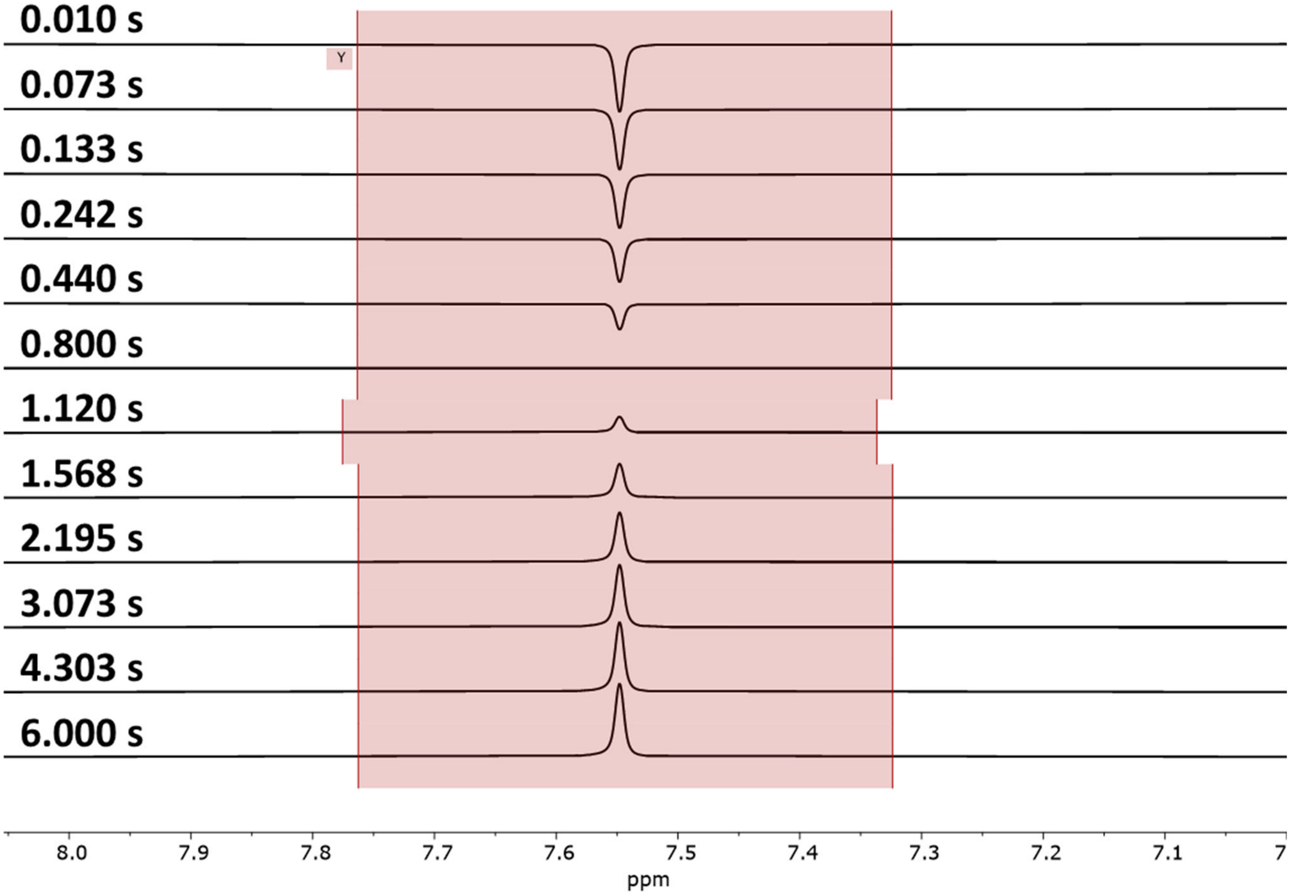

Supplementary Figure 205. T_1 (inverse recovery). Stacked spectra of the inverse recovery experiment to determine the T_1 values of the chloroform signal, in which the signal intensity (Y) is plotted against the time in seconds (X) ([**Mn1**] = 300 µM; [**V2**] = 150 µM; ^{1}H, 300 MHz, chloroform : chloroform-*d* : acetonitrile-d_3, 1 : 4 : 5, v/v/v, 299 K).

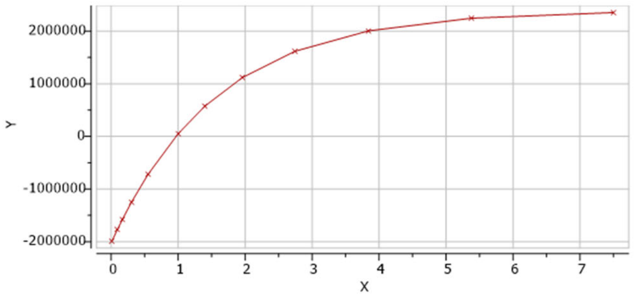

Sample 3:
[Mn1] = 300 µM
[V2] = 300 µM

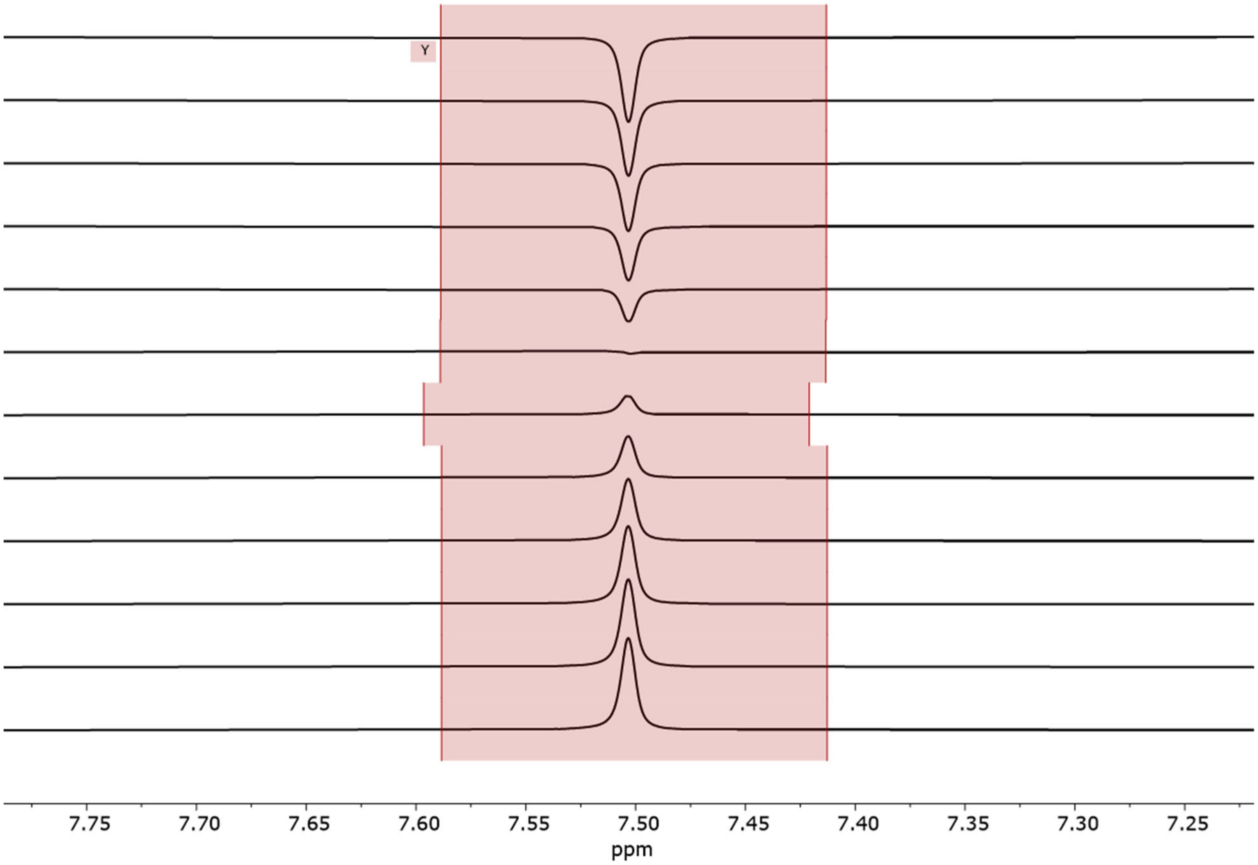

Supplementary Figure 206. T_1 (inverse recovery). Stacked spectra of the inverse recovery experiment to determine the T_1 values of the chloroform signal, in which the signal intensity (Y) is plotted against the time in seconds (X) ([**Mn1**] = 300 µM; [**V2**] = 300 µM; ^{1}H, 300 MHz, chloroform : chloroform-*d* : acetonitrile-d_3, 1 : 4 : 5, v/v/v, 299 K).

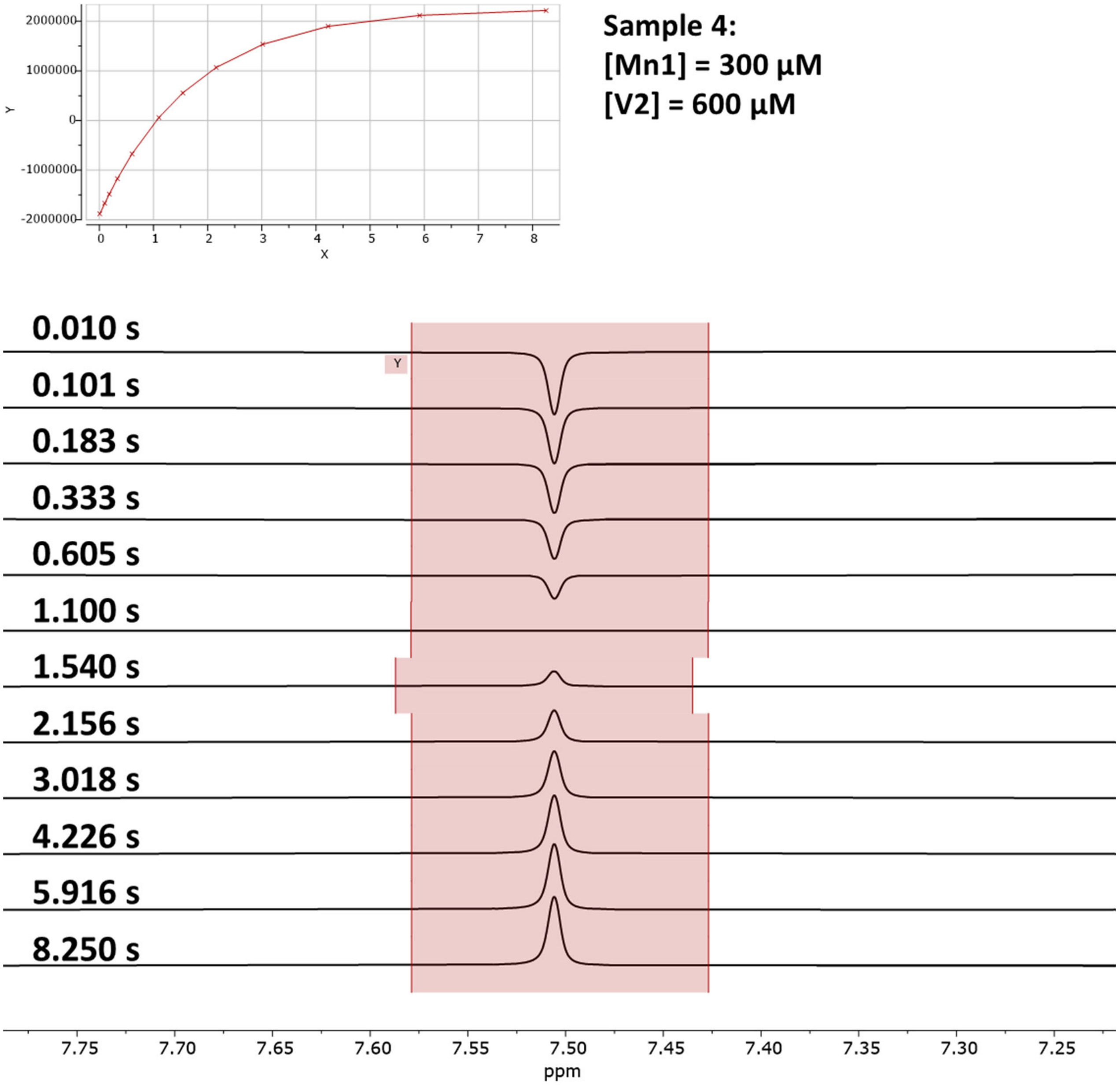

Supplementary Figure 207. T_1 (inverse recovery). Stacked spectra of the inverse recovery experiment to determine the T_1 values of the chloroform signal, in which the signal intensity (Y) is plotted against the time in seconds (X) ([**Mn1**] = 300 µM; [**V2**] = 600 µM; ^{1}H, 300 MHz, chloroform : chloroform-*d* : acetonitrile-d_3, 1 : 4 : 5, v/v/v, 299 K).

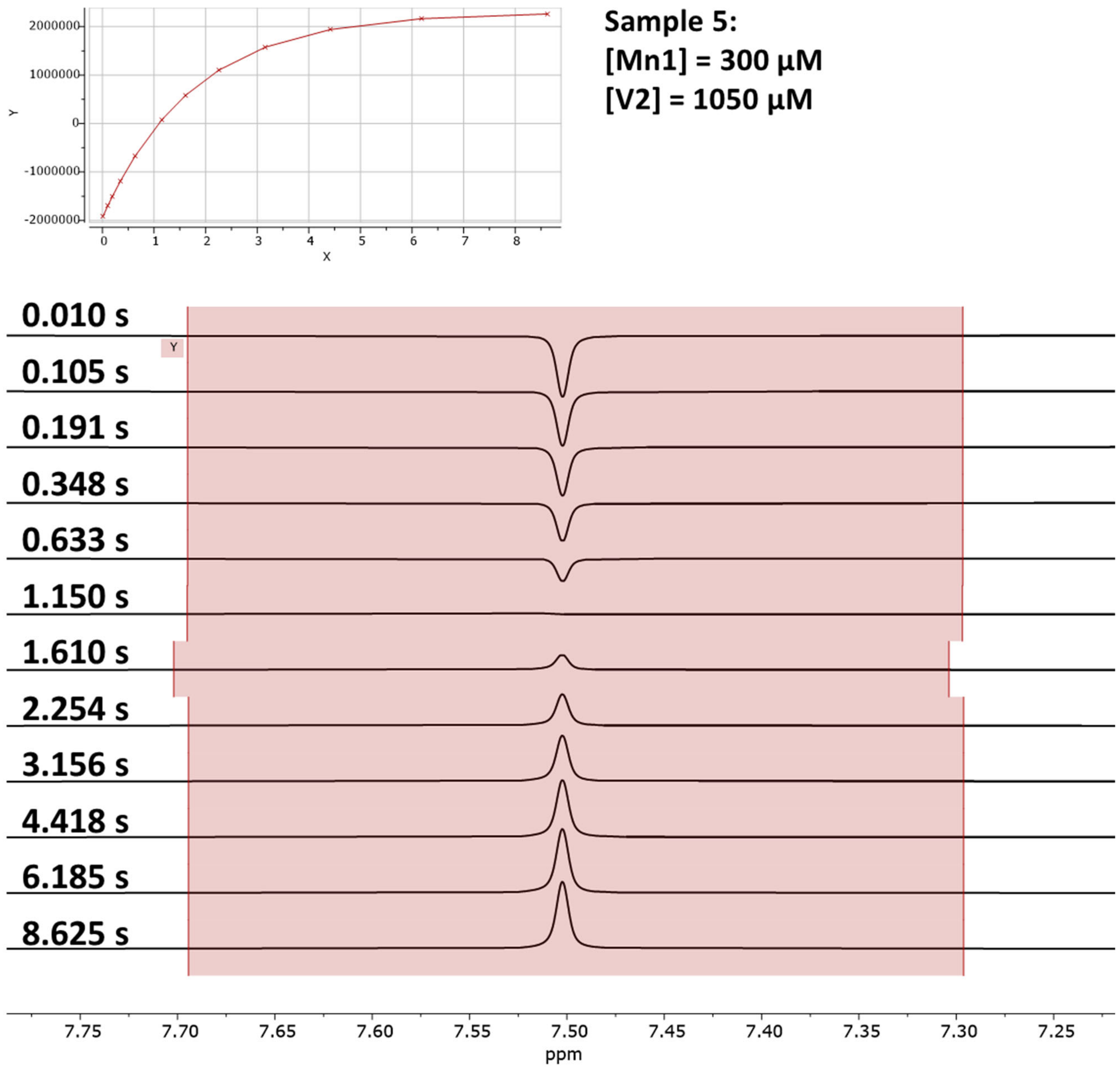

Supplementary Figure 208. T_1 (inverse recovery). Stacked spectra of the inverse recovery experiment to determine the T_1 values of the chloroform signal, in which the signal intensity (Y) is plotted against the time in seconds (X) ([**Mn1**] = 300 µM; [**V2**] = 1050 µM; ^{1}H, 300 MHz, chloroform : chloroform-*d* : acetonitrile-d_3, 1 : 4 : 5, v/v/v, 299 K).

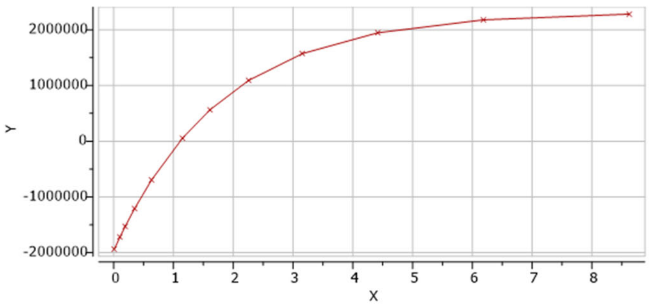

Sample 6:
[Mn1] = 300 µM
[V2] = 1725 µM

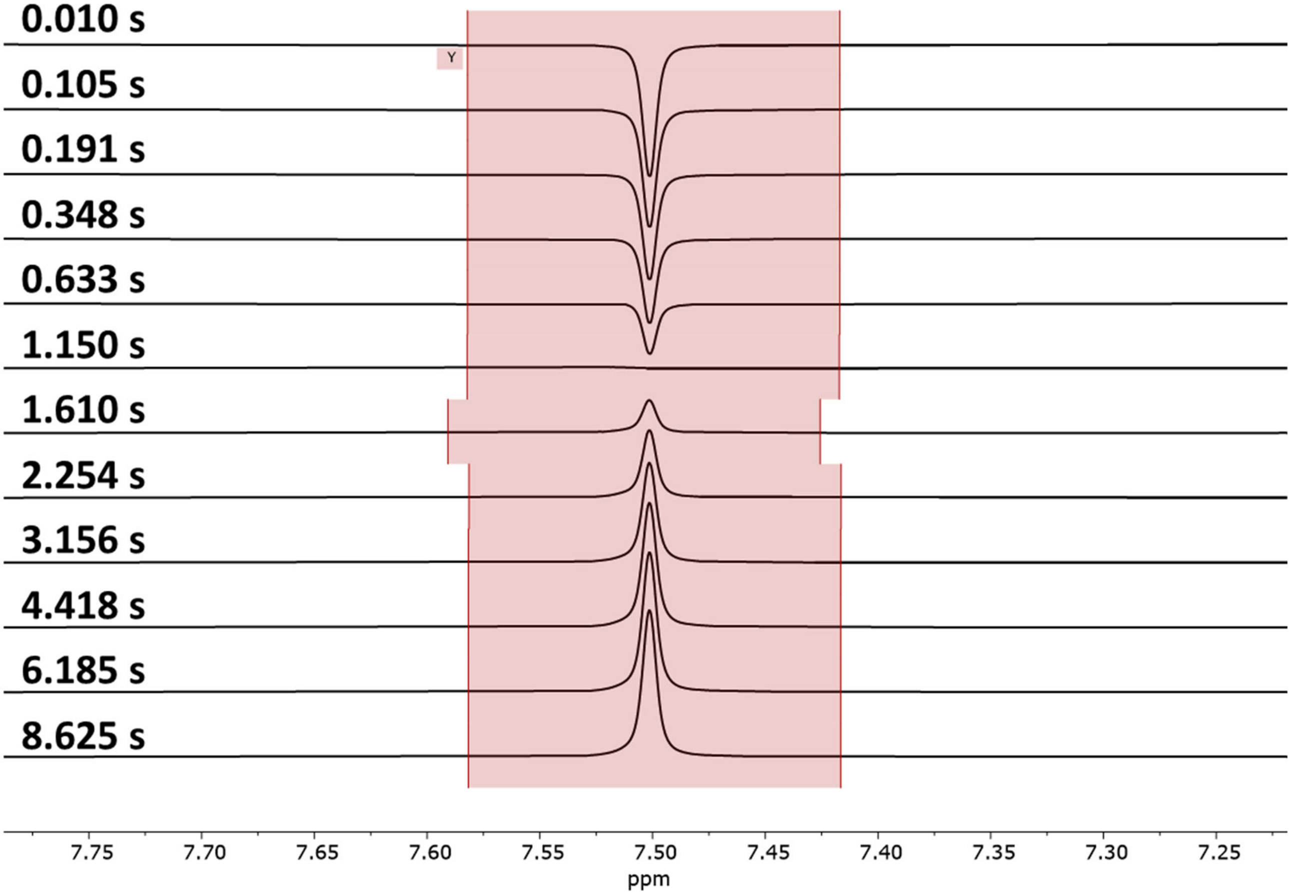

Supplementary Figure 209. T_1 (inverse recovery). Stacked spectra of the inverse recovery experiment to determine the T_1 values of the chloroform signal, in which the signal intensity (Y) is plotted against the time in seconds (X) ([**Mn1**] = 300 µM; [**V2**] = 1725 µM; ^{1}H, 300 MHz, chloroform : chloroform-*d* : acetonitrile-*d*$_3$, 1 : 4 : 5, v/v/v, 299 K).

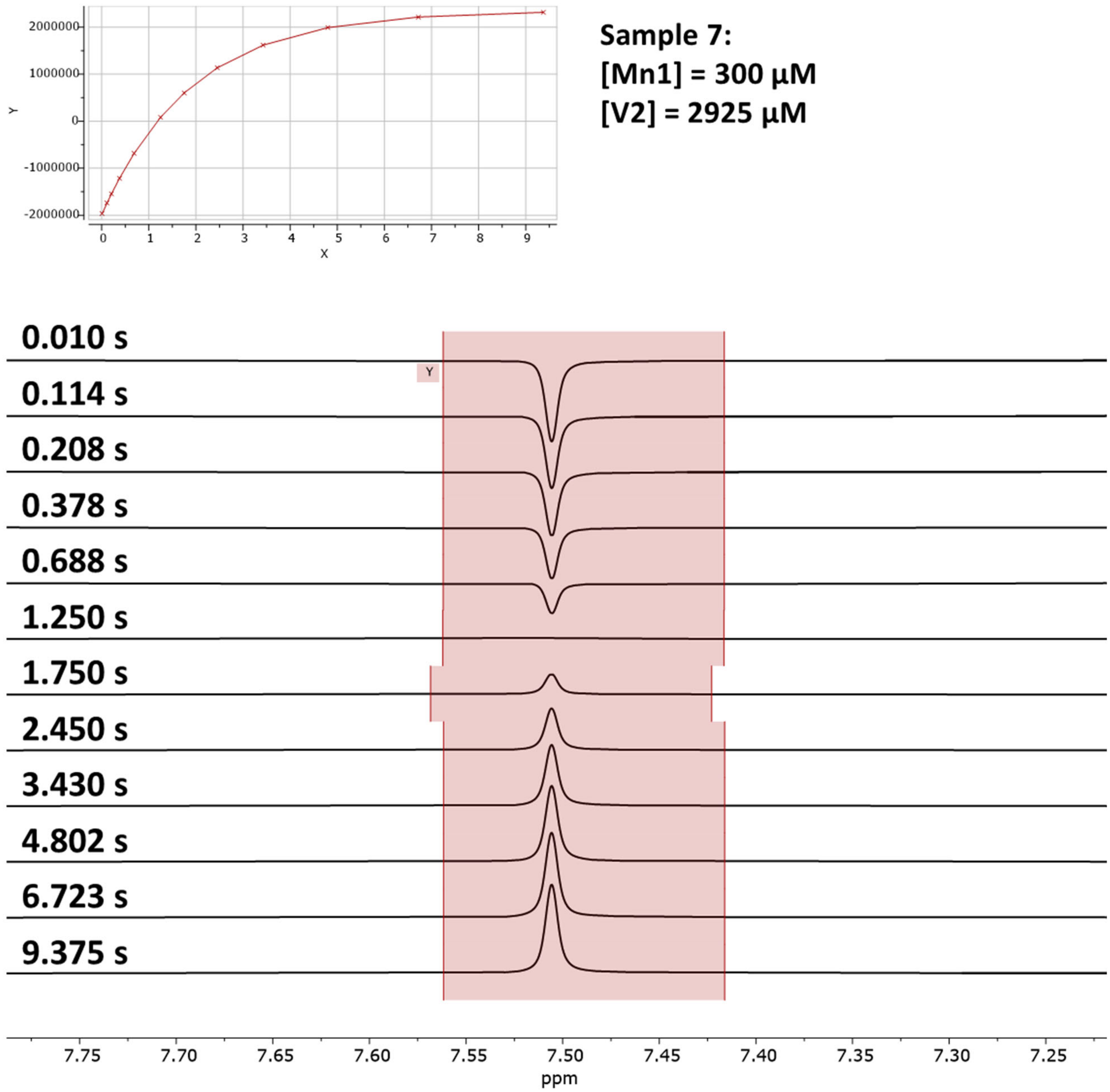

Supplementary Figure 210. T_1 (inverse recovery). Stacked spectra of the inverse recovery experiment to determine the T_1 values of the chloroform signal, in which the signal intensity (Y) is plotted against the time in seconds (X) ([**Mn1**] = 300 µM; [**V2**] = 2925 µM; ^{1}H, 300 MHz, chloroform : chloroform-*d* : acetonitrile-d_3, 1 : 4 : 5, v/v/v, 299 K).

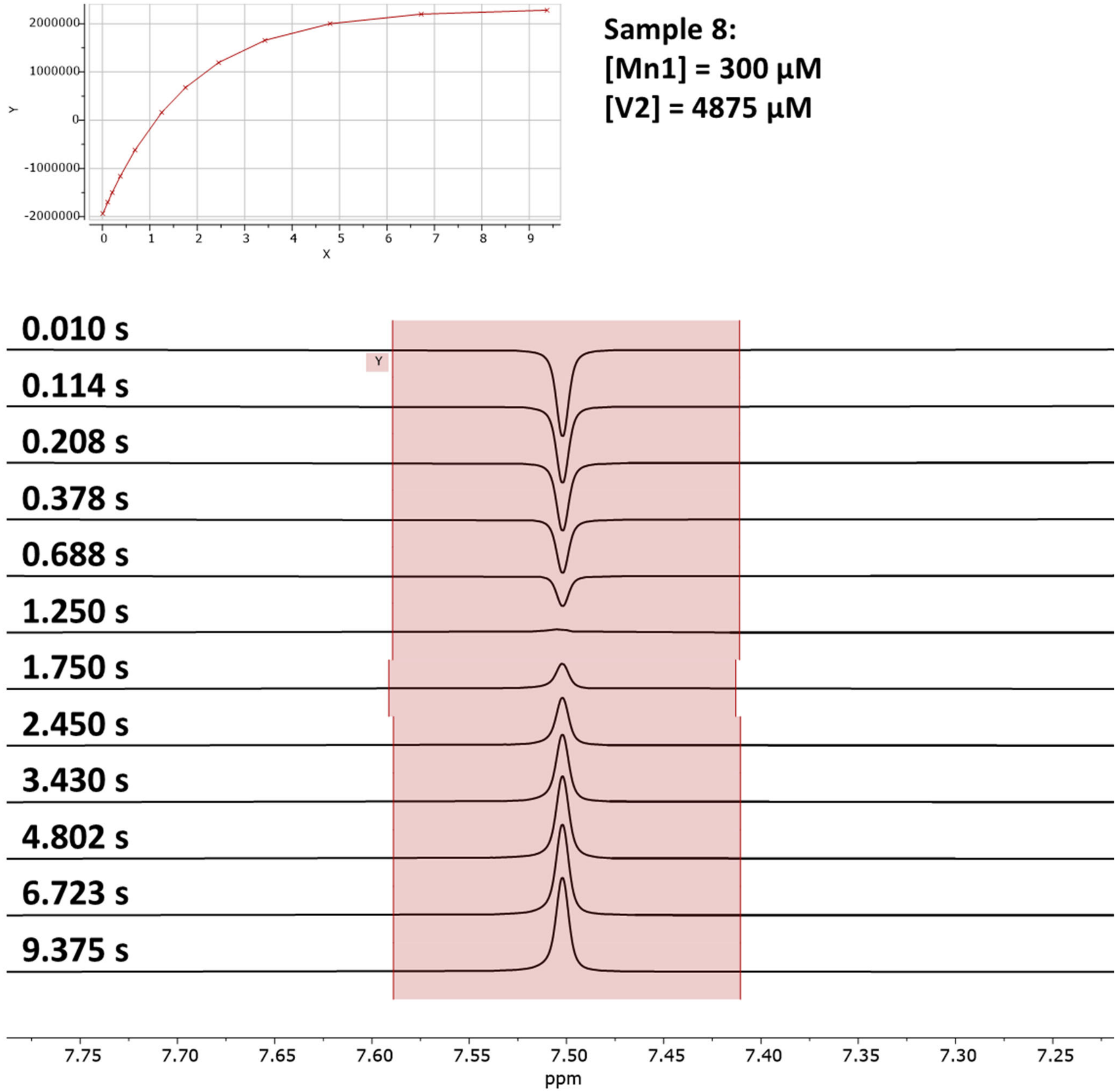

Supplementary Figure 211. T_1 (inverse recovery). Stacked spectra of the inverse recovery experiment to determine the T_1 values of the chloroform signal, in which the signal intensity (Y) is plotted against the time in seconds (X) ([**Mn1**] = 300 µM; [**V2**] = 4875 µM; ^{1}H, 300 MHz, chloroform : chloroform-*d* : acetonitrile-*d₃*, 1 : 4 : 5, v/v/v, 299 K).

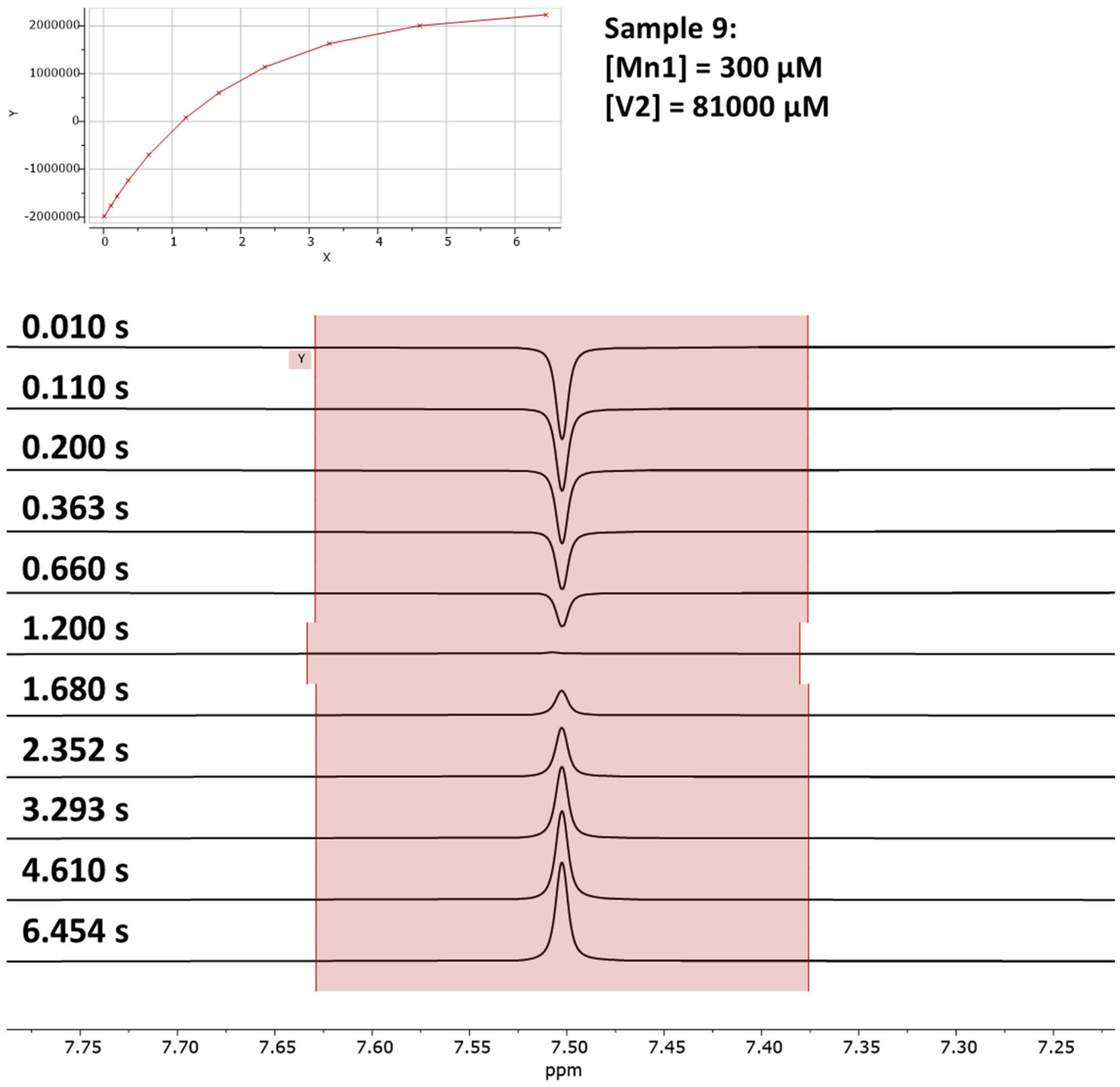

Supplementary Figure 212. T_1 (inverse recovery). Stacked spectra of the inverse recovery experiment to determine the T_1 values of the chloroform signal, in which the signal intensity (Y) is plotted against the time in seconds (X) ([**Mn1**] = 300 µM; [**V2**] = 81000 µM; ^{1}H, 300 MHz, chloroform : chloroform-*d* : acetonitrile-d_3, 1 : 4 : 5, v/v/v, 299 K).

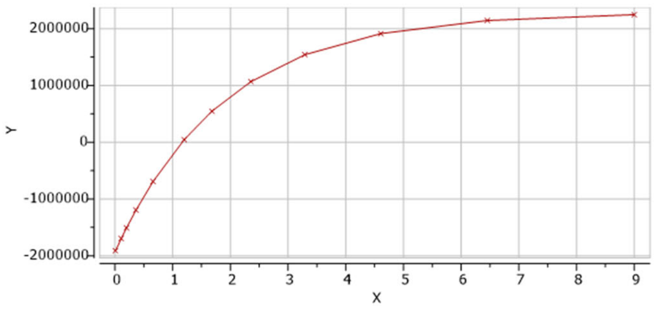

Sample 10:
[Mn1] = 300 µM
[V2] = 135000 µM

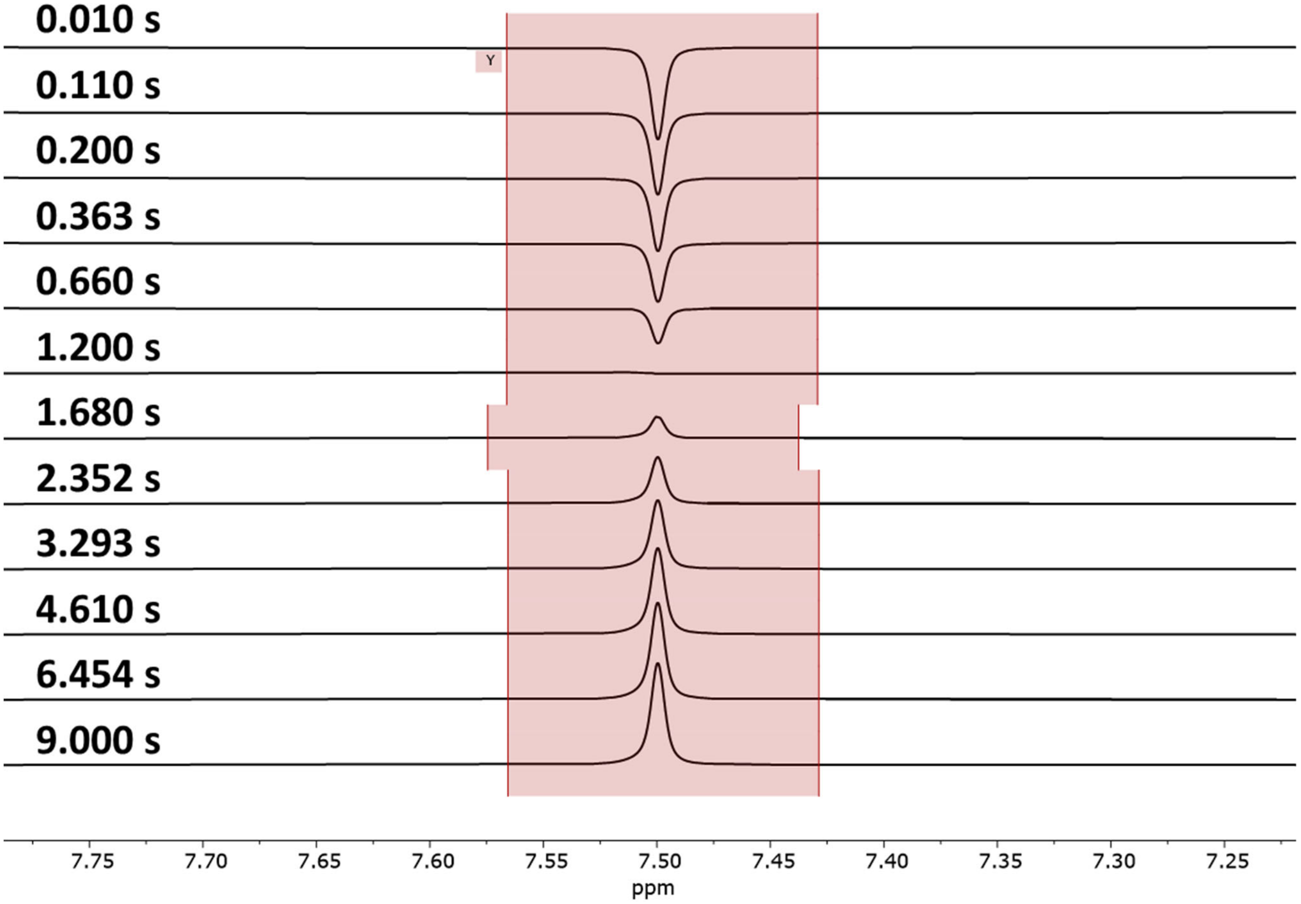

Supplementary Figure 213. T_1 (inverse recovery). Stacked spectra of the inverse recovery experiment to determine the T_1 values of the chloroform signal, in which the signal intensity (Y) is plotted against the time in seconds (X) ([**Mn1**] = 300 µM; [**V2**] = 135000 µM; ^{1}H, 300 MHz, chloroform : chloroform-*d* : acetonitrile-d_3, 1 : 4 : 5, v/v/v, 299 K).

5. How-to guide for utilizing PRE relaxometry studies to determine association and dissociation constants in paramagnetic host-guest complexes

Determination of association constants using relaxometry in PRE NMR

To determine the association constant of a host-guest complex of choice, a reporter solvent needs to be chosen. The reporter solvent needs to interact with the host of choice in the form of (weak) binding, and exchange is required to occur. Additionally, the guest of choice should have a stronger binding constant than the reporter solvent. To determine the suitable host concentration in these studies, a test run should be performed first. With an estimate of the association constant in mind, at least three (but more if desired) samples should be prepared. Sample 1 should contain only diamagnetic guest (determines $R_{1,0}$), sample 2 should contain host and no guest (determines the maximum $R_{1,obs}$), and sample 3 should contain host and the concentration of guest that is estimated to give complete association of the host (determines the minimum $R_{1,obs}$). See below for K_a estimation calculations.

During the test run, the T_1 relaxation time constant of the reporter solvent is measured via the inversion recovery pulse sequence of all samples. The relaxation rate for sample 1 ($R_{1,0}$) is used to calculate $R_{1,p}$ for samples 2 and 3 using Supplementary Equation 28. Between samples 2 and 3, a sufficient difference in $T_{1,p}$ should be observed (~0.3 s minimum or a minimum difference in $R_{1,p}$ of ~0.75, see Supplementary Figure 214) for the results to be reliable.

$$\mathrm{R_{1,obs}} = \mathrm{R_{1,0}} + \mathrm{R_{1,p}} \qquad \text{Supplementary Equation 28}$$

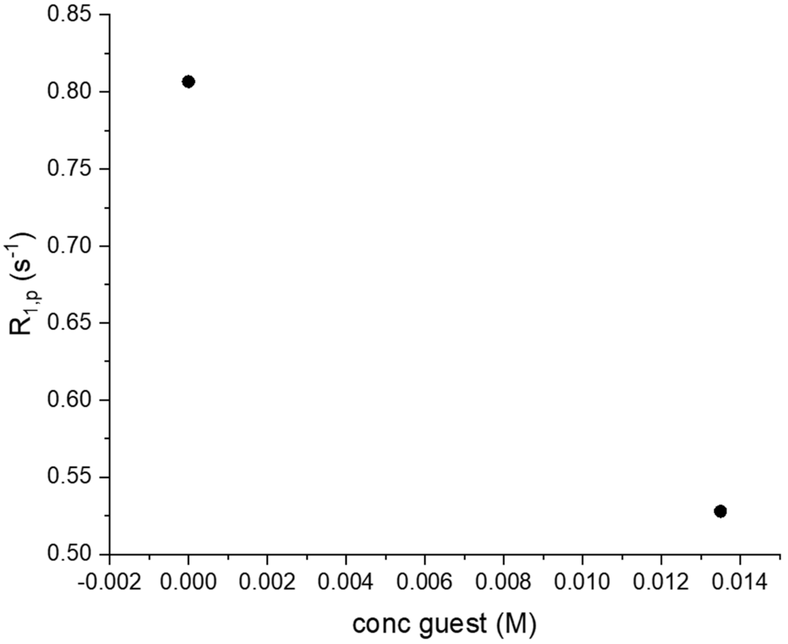

Supplementary Figure 214. Example of a test-run of Mn1/V2 (25 °C, 300 MHz, reporter solvent: chloroform) using 0.3 mM of host and 0 mM of guest for sample 2 (R_1 = 0.81 s^{-1}) and 13.5 mM of V2 for sample 3 (R_1 = 0.53 s^{-1}).

When the difference in $T_{1,p}$ between samples 2 and 3 is insufficient, a couple of parameters may improve the results of these studies: a higher concentration of host, varying the temperature, or measuring at a lower magnetic field.

After a successful test run, the samples can be prepared separately, varying in guest concentration, or the guest can be added in between measurements. The latter method is preferred if a limited amount of compound is available but is not desirable if it is known that the guest requires a longer time to form the host-guest complex.

After measuring the T_1-values of the sample(s), the $R_{1,p}$ ($1/T_{1,p}$) is plotted against the added concentration of guest to give a typical binding curve (Supplementary Figure 215).

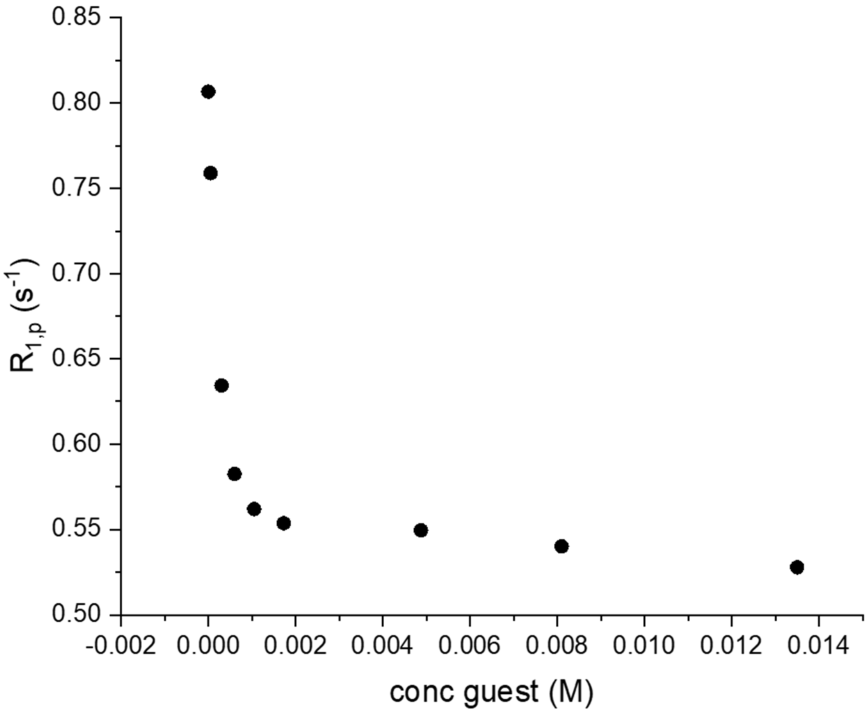

Supplementary Figure 215. Example of a titration curve of Mn1/V2 (25 °C, 300 MHz, reporter solvent: chloroform) using 0.3 mM of host and up to 13.5 mM of V2.

Supplementary Table 21. Plotted values of concentrations guest (V2) and $R_{1,p}$ values used in Supplementary Figure 215.

Conc. guest (M)	$R_{1,p}$ (s^{-1})
0	0.806634
0.00005	0.758904
0.0003	0.634244
0.0006	0.58252
0.00105	0.562054
0.001725	0.553691
0.004875	0.549571
0.0081	0.54017
0.0135	0.527958

The obtained R_1 values can be used to calculate the K_a, using OriginLabs (or any similar software), using the following custom fitting equations:

```
Function type in OriginPro 2022: LabTalk Equations
Independent variables: Gt
Dependent variables: Robs
Parameters: Ka, Rb
Constants: Ht, Rf

Function body (Dependent variables: Robs):
a = Ka;
b = 1 - Ka*Gt + Ka*Ht;
c = -Gt;
G = (-b + sqrt(b^2 - 4*a*c))/(2*a);
Robs = ((Rb - Rf)*(Gt - G))/Ht + Rf;

Constants (for this specific fit):
Ht = 0.0003
Rf = 0.806634
```

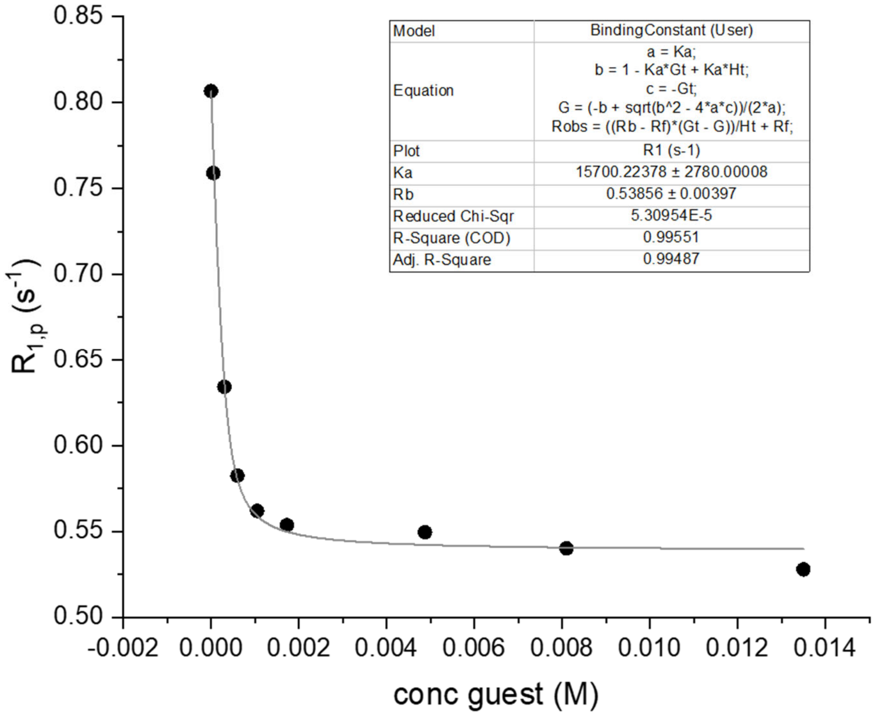

Supplementary Figure 216. Example of a titration curve of Mn1/V2 (25 °C, 300 MHz, reporter solvent: chloroform) using 0.3 mM of host and up to 13.5 mM of V2 after fitting.

In the equations Ka = the association constant (M^{-1}), Gt = $[G]_{total}$ = the total guest concentration (M), Ht = $[H]_{total}$ = the total host concentration (M), Rb = R_{bound} = the relaxation rate of the bound species (s^{-1}), Rf = R_{free} = the relaxation rate of the unbound species (s^{-1}). For further information and earlier studies on enzymatic systems, see Bertini et al.[10].

K_a calculations

To calculate the expected percentage of bound host, the desired host concentration ($[H]_{tot}$, M), the ligand concentration ($[G]_{tot}$, M), and the association constant estimation (K_a, M^{-1}) are used. The concentration of bound host ($[HG]$, M) is calculated by the quadratic Supplementary Equation 29.

$$[HG] = \frac{-b-\sqrt{b^2-4ac}}{2a} \qquad \text{Supplementary Equation 29}$$

in which

$a = 1, b = -\left([G]_{tot} + [H]_{tot} + \frac{1}{K_a}\right)$, and $c = [H]_{tot} \cdot [G]_{tot}$

It is converted to the percentage of bound host with Supplementary Equation 30:

$$\frac{[HG]}{[H]_{tot}} \cdot 100 = \%\text{bound host}$$ Supplementary Equation 30

Determination of dissociation rate constants using relaxometry in PRE NMR

To determine the dissociation constants using this method, a set of requirements needs to be met before reliable results can be obtained.

A sample should be prepared containing the paramagnetic host and the diamagnetic guest (sample 1). Subsequently, the T_1 relaxation time constant at a given temperature is measured by the inversion recovery pulse sequence and the T_2 by either the CPMG or PROJECT-CPMG pulse sequence. Since these observed R_1 and R_2 values are the sums of the paramagnetic and the diamagnetic contributions (Supplementary Equation 31), another sample should be prepared containing only the diamagnetic guest (sample 2) of which the R_1 and R_2 values are measured (R_0). From the obtained values of samples 1 and 2, the paramagnetic contribution to the relaxation rates (R_p) can be calculated.

$$R_{1_{obs}} = R_{1,0} + R_{1,p}$$ Supplementary Equation 31

The obtained values for $R_{1,p}$ and $R_{2,p}$ are then used to determine whether the following requirements are met:

1. R_p should increase at higher temperatures: $\frac{\partial R_p}{\partial T} > 0$
2. R_p is independent of the field strength: $\frac{\partial R_p}{\partial \omega} = 0$
3. $R_{1,p}$ should be approximately equal to $R_{2,p}$: $\frac{R_{2,p}}{R_{1,p}} \approx 1$

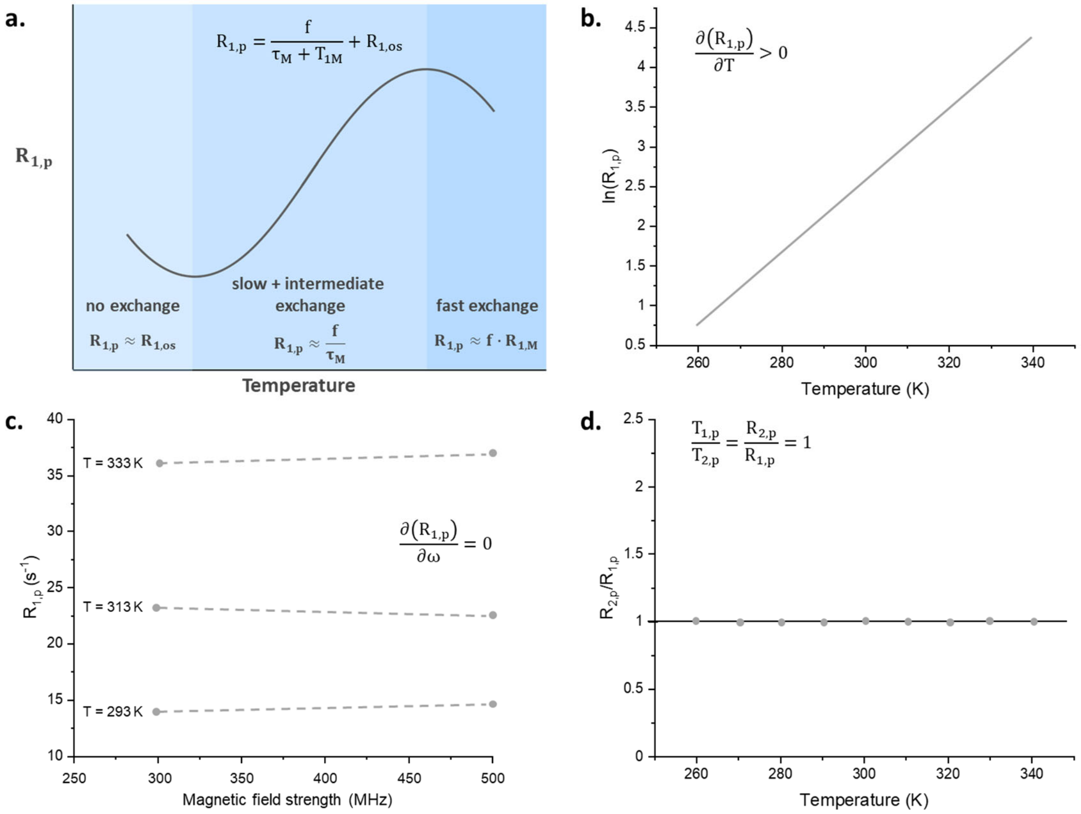

Supplementary Figure 217. Results obtained if the three listed requirements are met. **a**. Indication of the different dominating factors governing exchange at different temperatures (low T is left, high T is right). At low temperatures where there is no exchange, the outer-sphere interactions are dominating; at higher temperatures the slow and intermediate exchange regime holds, and the exchange is dominating; and in the fast exchange regime the inner-sphere interactions dominate. **b**. Graph of $\ln(R_{1,p})$ against the temperature, displaying a positive slope with increasing temperature. **c**. $R_{1,p}$ values plotted against temperature comparing 300 MHz and 500 MHz experiments. **d**. Ratio of $R_{2,p}$ and $R_{1,p}$ plotted against temperature.

Testing the validity of each requirement

Requirement 1: $\frac{\partial R_p}{\partial T} > 0$

a) Choose two different temperatures and measure the T_1 and T_2 time constants at each temperature point for samples 1 and 2. Ideally, the difference should be >20-30 K.

b) Calculate and plot R_p versus temperature. A positive slope indicates that exchange occurs in an appropriate regime for extracting rate constants. A negative slope could indicate a fast-exchange regime (inner-sphere contributions dominate) or a very slow-exchange regime (outer-sphere contributions

dominate) but is overall unsuitable for extracting rate constants. See Mildvan et al.[4] for an elaboration on the theory.

Requirement 2: $\frac{\partial R_p}{\partial \omega} = 0$

a) R_p should be determined using NMR spectrometers with different magnetic field strengths. A 300 MHz and a 500 MHz spectrometer were used for the present studies.

b) Calculate and plot R_p versus field strength (ω). If a non-zero slope is observed, it is evident that nuclear relaxation rather than chemical exchange is dominating $R_{p,}$ and extraction of rate constants becomes challenging.

Requirement 3: $\frac{R_{2,p}}{R_{1,p}} \approx 1$

a) Use the data acquired in *Requirements 1* and *2* to calculate $R_{2,p}/R_{1,p}$. If the ratio is not ~1 then either inner-sphere or outer-sphere interactions are dominant.

If the ratio between $R_{2,p}$ and $R_{1,p}$ starts to increasingly deviate from 1 upon lowering the temperature, an extra sample can be prepared to account for the outer-sphere interactions (sample 3). These interactions are non-bonding interactions between the free diamagnetic guest and the paramagnetic host and arise solely from the presence of a paramagnetic species. Therefore, a new host should be synthesized which resembles the original host as much as possible, but in which the binding pocket is blocked or in which exchange, and therefore association, is prevented. After correcting $R_{1,p}$ and $R_{2,p}$ for the outer-sphere contribution, *Requirement 3* should now be met.

Extracting Rate Constants Through Mathematical Simplification

In the case that all requirements are met, Supplementary Equation 32 can be simplified to Supplementary Equation 33. This is possible as the above requirements indicate that the residence time of the guest near or on the metal-center (τ_M, s) is much greater than T_M (inner-sphere relaxation constant) and outer-sphere effects (R_{os}). The observed dissociation rate constant (k_{obs}) can be calculated from the inverse of τ_M.

$$R_p = \frac{f}{\tau_M + T_M} + R_{os} \qquad \text{Supplementary Equation 32}$$

$$R_p = \frac{f}{\tau_M} + R_{os} \quad ; \tau_M^{-1} = k_{obs} \qquad \text{Supplementary Equation 33}$$

in which ''f'' is the mole-fraction of the ligand bound to the host. Under saturation conditions this is simply the total host to total guest ratio. However, the fraction of the host bound by guest must be determined for any given ligand/host sample, using the determined association constant (K_a) for the system.

When *Requirement 3* is met under all measured temperatures and sample 3 is not required, then the R_{os} term can be ignored and the equation is further simplified to equation Supplementary Equation 34.

$$R_p = \frac{f}{\tau_M} \qquad \text{Supplementary Equation 34}$$

When there is no ligand dependence on the exchange process, k_{obs} is the true dissociation rate constant k_d. However, when a ligand dependence is observed, k_{obs} must be corrected for the order of the dependence. Therefore, the ligand dependence should be tested for studied systems by determining the τ_M values at different ligand concentrations.

From the obtained k_d at various temperature points, an Eyring plot can be constructed, and the dissociation parameters $\Delta H^‡$, $\Delta S^‡$, $\Delta G^‡$ can be determined.

Fast Exchange Regime

If the system is in the fast-exchange regime, the inner-sphere relaxation dominates over the exchange, and it is not viable to extract rate constants under these conditions. However, when the behaviour is observed to be in the intermediate exchange regime, where it transitions from slow to fast (Supplementary Figure 217), the rate constant can still be extracted provided sufficient fast-exchange temperature points can be acquired. The value of R_M can be calculated using Supplementary Equation 35.

$$R_{obs} = \chi_{HG} \cdot R_M + \chi_G \cdot R_0 \quad ; \quad R_M = T_M^{-1} \qquad \text{Supplementary Equation 35}$$

in which χ_{HG} and χ_G are the mol fractions of bound and free guest, respectively. After measuring R_M at several temperatures, $\ln(R_M)$ is plotted vs temperature and R_M (and thus T_M) is extrapolated for temperatures where R_M is not dominating (in which $\frac{\partial R_p}{\partial T} > 0$). The calculated values for T_M can then be used to calculate τ_M using Supplementary Equation 32.

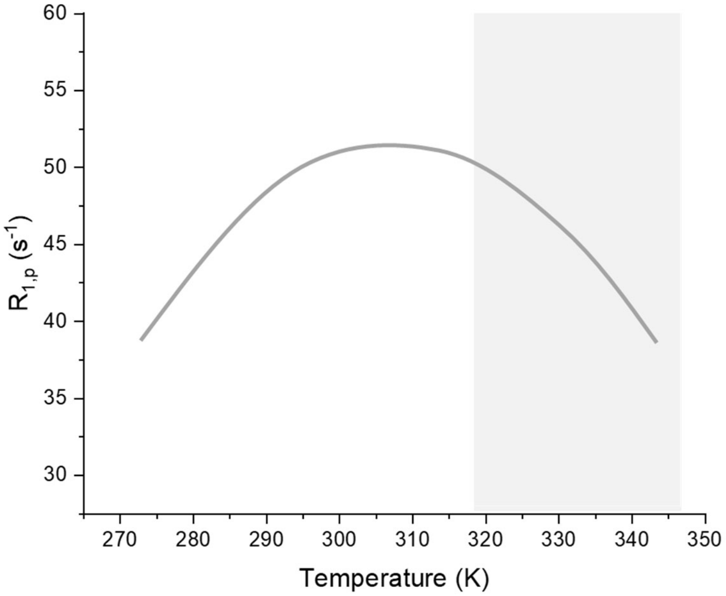

Supplementary Figure 218. Schematic depiction of the transition between the intermediate-exchange rate (positive slope) and the fast-exchange rate (negative slope). The $R_{1,p}$ (s^{-1}) is plotted against the temperature (K). The temperature range which yields a negative slope for the change in $R_{1,p}$ is indicated in grey. This range can be used to calculate R_M.

6. Supplementary References

1. Coumans, R. G. E., Elemans, J. A. A. W., Rowan, A. E. & Nolte, R. J. M. Interlocked porphyrin switches. *Chem. – Eur. J.* **19**, 7758–7770 (2013).
2. Gilissen, P. J. *et al.* Rapid and scalable synthesis of chiral porphyrin cage compounds. *Tetrahedron* **75**, 4640–4647 (2019).
3. Swartjes, A., White, P. B., Lammertink, M., Elemans, J. A. A. W. & Nolte, R. J. M. Host–guest exchange of viologen guests in porphyrin cage compounds as studied by selective exchange spectroscopy (1D EXSY) NMR. *Angew. Chem. Int. Ed.* **60**, 1254–1262 (2021).
4. Mildvan, A. S. & Cohn, M. Aspects of enzyme mechanisms studied by nuclear spin relaxation induced by paramagnetic probes. in *Advances in Enzymology and Related Areas of Molecular Biology* 1–70 (John Wiley & Sons, Ltd, 1970). doi:10.1002/9780470122785.ch1.

5. Carr, H. Y. & Purcell, E. M. Effects of diffusion on free precession in nuclear magnetic resonance experiments. *Phys. Rev.* **94**, 630–638 (1954).
6. Aguilar, J. A., Nilsson, M., Bodenhausen, G. & Morris, G. A. Spin echo NMR spectra without J modulation. *Chem. Commun.* **48**, 811–813 (2011).
7. Supramolecular.org - Binding constant calculators | Supramolecular. http://supramolecular.org/.
8. Hibbert, D. B. & Thordarson, P. The death of the Job plot, transparency, open science and online tools, uncertainty estimation methods and other developments in supramolecular chemistry data analysis. *Chem. Commun.* **52**, 12792–12805 (2016).
9. Thordarson, P. Determining association constants from titration experiments in supramolecular chemistry. *Chem Soc Rev* **40**, 1305–1323 (2011).
10. Bertini, I., Fragai, M., Luchinat, C. & Talluri, E. Water-Based Ligand Screening for Paramagnetic Metalloproteins. *Angew. Chem. Int. Ed.* **47**, 4533–4537 (2008).
